# Review of Grain Fortification Legislation, Standards, and Monitoring Documents

**DOI:** 10.9745/GHSP-D-17-00427

**Published:** 2018-06-27

**Authors:** Kristin J. Marks, Corey L. Luthringer, Laird J. Ruth, Laura A. Rowe, Noor A. Khan, Luz María De-Regil, Ximena López, Helena Pachón

**Affiliations:** a Food Fortification Initiative, Atlanta, GA, USA.; b Emory University, Atlanta, GA, USA.; c Global Alliance for Improved Nutrition, Geneva, Switzerland.; d U.S. Centers for Disease Control and Prevention, Atlanta, GA, USA.; e Project Healthy Children, Cambridge, MA, USA.; f Nutrition International, Ottawa, Canada.; g Granotec, Santiago, Chile.

## Abstract

**Objective:**

Analyze the content of documents used to guide mandatory fortification programs for cereal grains.

**Methods:**

Legislation, standards, and monitoring documents, which are used to mandate, provide specifications for, and confirm fortification, respectively, were collected from countries with mandatory wheat flour (n=80), maize flour (n=11), and/or rice (n=6) fortification as of January 31, 2015, yielding 97 possible country-grain combinations (e.g., Philippines-wheat flour, Philippines-rice) for the analysis. After excluding countries with limited or no documentation, 72 reviews were completed, representing 84 country-grain combinations. Based on best practices, a criteria checklist was created with 44 items that should be included in fortification documents. Two reviewers independently scored each available document set for a given country and food vehicle (a country-grain combination) using the checklist, and then reached consensus on the scoring. We calculated the percentage of country-grain combinations containing each checklist item and examined differences in scores by grain, region, and income level.

**Results:**

Of the 72 country-grain combinations, the majority of documentation came from countries in the Americas (46%) and Africa (32%), and most were from upper and lower middle-income countries (73%). The majority of country-grain combinations had documentation stating the food vehicle(s) to be fortified (97%) and the micronutrients (e.g., iron) (100%), fortificants (e.g., ferrous fumarate) (88%), and fortification levels required (96%). Most (78%) stated that labeling is required to indicate a product is fortified. Many country-grain combinations described systems for external (64%) monitoring, and stated that industry is required to follow quality assurance/quality control (64%), though detailed protocols (33%) and roles and responsibilities (45%) were frequently not described.

**Conclusions:**

Most country-grain combinations have systems in place for internal, external, and import monitoring. However, documentation of other important items that would influence product compliance to national standard, such as roles and responsibilities between agencies, the cost of regulating fortification, and enforcement strategies, are often lacking. Countries with existing mandatory fortification can improve upon these items in revisions to their documentation while countries that are beginning fortification can use the checklist to assist in developing new policies and programs.

## Abstract (precis)

The majority of countries with mandatory grain fortification requirements document the technical specifications for grain fortification, such as allowable food vehicles and fortification levels required. Most document systems for monitoring. However, detailed protocols, descriptions of roles and responsibilities, means to support the cost of regulation, enforcement strategies, and methods for reporting monitoring results to stakeholders are generally lacking.

## INTRODUCTION

Large-scale fortification of staple foods is a cost-effective and sustainable strategy for substantially reducing micronutrient malnutrition.^1^ The fortification of cereal grains with folic acid, iron, zinc, vitamin B12, niacin, riboflavin, thiamin, vitamin A, and other micronutrients has gained global traction as a strategy to improve human health. Fortification has led to reduced incidence of neural tube defects^2^^,^^3^ and nutritional anemia,^4^ among other health outcomes. According to the Food Fortification Initiative, in 2016, 34% of industrially milled wheat flour, 57% of industrially milled maize flour, and 1% of industrially milled rice was fortified (71%, 29%, and 45% of wheat flour, maize flour, and rice, respectively, was industrially milled), and 87 countries mandated the fortification of at least one of these cereal grains.^5^

For governments to ensure effective food fortification, enactment of laws and regulations provide legal authority and a regulatory framework.^1^ Mandatory fortification, as compared to voluntary, is more likely to achieve and sustain the desired health benefits of fortification.^3^^,^^6^ The regulatory framework specific to food fortification provides the basis for ensuring the quality and safety of products and for meeting public health nutrition objectives.^7^^,^^8^ Practical implementation of fortification is challenging though, as evidenced by global insufficient compliance against fortification standards.^7^ Therefore, periodic government monitoring will help determine whether program objectives are being met.

Given the challenges of implementing large-scale, cereal-grain fortification, effective legislation, standards, and monitoring documents can provide clear guidance on key program decisions, activities, and milestones such as the micronutrients/premix required; financial responsibility of implementing and monitoring and enforcing fortification; labeling of fortified products; internal, external, commercial, and import monitoring procedures; incentives and penalties; laboratory methods; and reporting guidelines.^1^^,^^8^^–^^23^ The objectives of this review are to assess the content of legislation, standards, and monitoring documents used to guide mandatory cereal grain fortification programs in countries and to identify areas of strength and areas needing improvement. To the best of our knowledge, there has never been a review of cereal-grain legislation, standards, and monitoring documents' content conducted at global scale. Previous work has looked at individual regions or a handful of countries, has been limited to one food vehicle, and has rarely included monitoring documents.^8^^,^^10^^,^^21^^,^^24^

The purpose of this article is to assess cereal-grain legislation, standards, and monitoring documents among countries with mandatory grain fortification programs.

## METHODS

### Document Inclusion Criteria

Any country that had mandatory fortification of wheat flour, maize flour, or rice as of January 31, 2015, was included in this review. We defined mandatory fortification of cereal grains as “country has legislation that has the effect of mandating fortification of one or more types of wheat or maize flour or rice with at least iron or folic acid.”^5^ Under this definition, as of January 2015, 80 countries mandated wheat flour fortification, 11 mandated maize flour fortification, and 6 mandated rice fortification. This yielded a maximum of 97 possible country-grain combinations for the analysis (e.g., Philippines-wheat flour was one combination and Philippines-rice was another).

As of January 2015, 80 countries mandated wheat flour fortification, 11 mandated maize flour fortification, and 6 mandated rice fortification.

Documents included in our study consist of legislation and statutory instruments, standards, technical regulations and specifications, and monitoring guidelines (Box). Legislation and statutory instruments typically mandate national or regional fortification of the specified cereal grain and include initial legislation such as the food act (may also be known as the food and drug act or food control act)^1^^,^^17^; hereafter, we refer to these as legislation documents. Standards, technical regulations, and specifications typically provide any implementing rules, regulations, or guidelines, such as dictating which vitamins and minerals to include in fortification and the levels of each nutrient to be added, as well as packaging and labeling requirements^1^^,^^17^; hereafter, we refer to these as standards documents. Monitoring guidelines ensure that quality control measures are followed routinely and problems are corrected so that fortified products consistently abide by relevant standards and fortification achieves its maximum health impact^1^^,^^17^; hereafter, we refer to these as monitoring guidelines.

BOX **Description of Legislation, Standards, and Monitoring Documents^a^**

**Legislation:** establishes the legal framework and broad principles for fortification

Examples include:

- Statutory instruments
- Food law

**Standards:** mandate the specific legal requirements for food fortification

Examples include:

- Technical regulations
- Specifications

**Monitoring documents:** provide instructions to track the operational performance of a fortification program

Examples include:

- Manuals
- Guidelines
- Procedures

^a^ Adapted from Allen et al. (2006)^1^ and Nathan (1999).^17^

Four monitoring categories were included in this analysis: internal, external, commercial, and import level.^1^ As part of internal monitoring, food processors use quality assurance and quality control procedures to ensure consistent production of quality fortified food.^11^ In external and import monitoring, government authorities periodically inspect and audit processes and test products at production and import sites, respectively, to ensure that fortification meets the country's specifications.^1^ As part of commercial monitoring, food safety inspectors check retail outlets to be sure the fortified product is in the marketplace and complies with regulations on packaging and labeling.^21^

In addition to the 3 main types of documents included (legislation, standards, and monitoring guidelines), we included the following document types when applicable: updates or amendments to legislation or standards; documents that are referenced by the legislation, standards, or monitoring guidelines; and reports of monitoring results. We recognize that countries have different legal systems, resulting in laws and regulations taking different forms; therefore, this review examines legislation, standards, and monitoring documents collectively and uses broad inclusion criteria for documents.^17^

**FIGURE 1:**
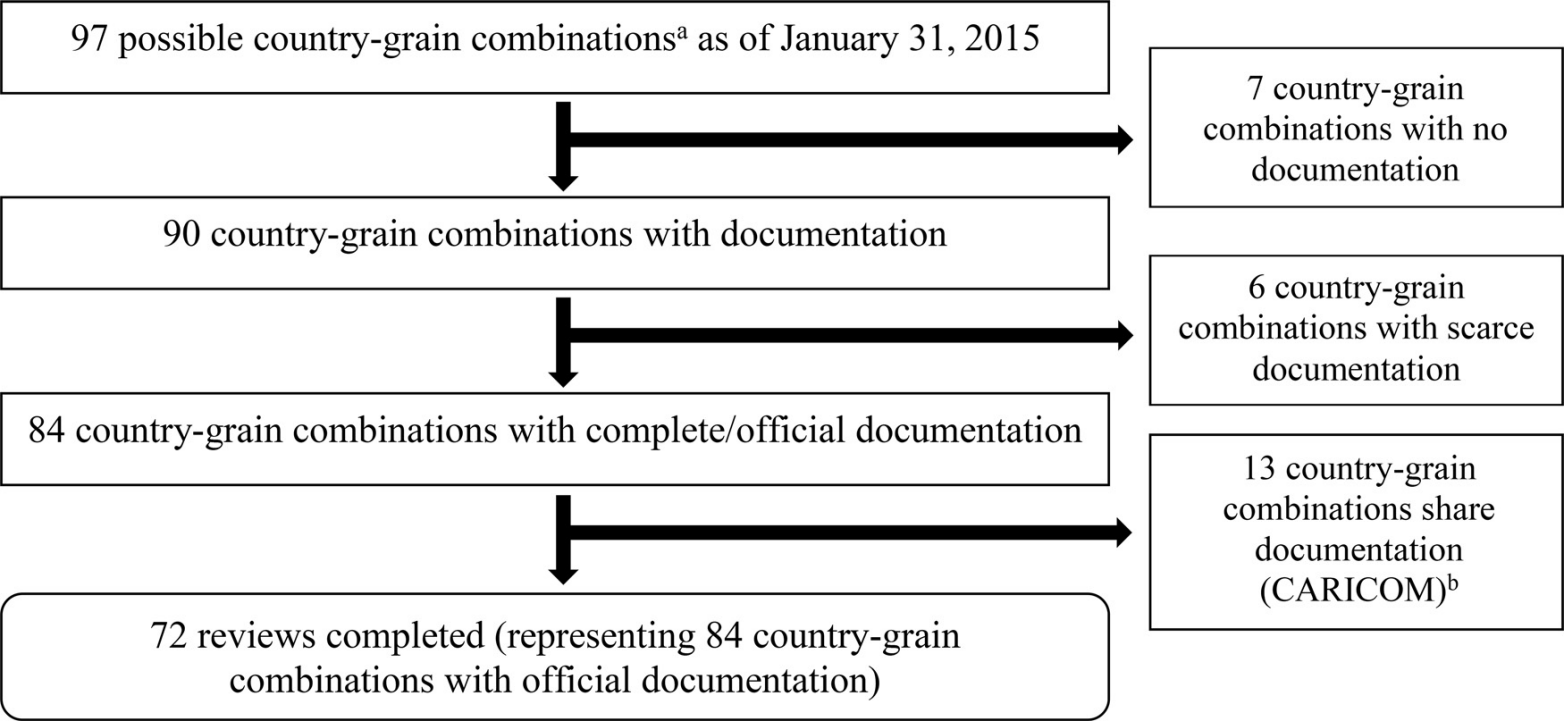
Flowchart of Country-Grain Combination^a^ Exclusions ^a^ Country-grain combination refers to the unit of analysis; countries that mandate the fortification of multiple cereal grains will contribute more than one country-grain combination (e.g., Philippines-wheat and Philippines-rice). ^b^ Thirteen Caribbean countries follow the Caribbean Community and Common Market (CARICOM) standard (Caribbean Community Secretariat, 1995): Antigua and Barbuda, Bahamas, Barbados, Dominica, Grenada, Guyana, Haiti, Jamaica, Saint Kitts and Nevis, Saint Lucia, Saint Vincent and the Grenadines, Suriname, and Trinidad and Tobago.

Of the 97 possible country-grain combinations, there were 7 countries with no documentation available (Benin, Guinea, Iran, Mali, Mauritania, Niger, and Saudi Arabia) (Figure 1). In 6 countries (Bahrain, Iraq, Jordan, Nepal, Oman, and Yemen), very limited documents were available so they were also excluded. Thirteen Caribbean countries follow the Caribbean Community and Common Market (CARICOM) standard^25^ and have no additional documentation; therefore, we reviewed these countries as a whole (Antigua and Barbuda, Bahamas, Barbados, Dominica, Grenada, Guyana, Haiti, Jamaica, Saint Kitts and Nevis, Saint Lucia, Saint Vincent and the Grenadines, Suriname, and Trinidad and Tobago). In summary, we completed 72 reviews representing 84 country-grain combinations (Supplement Table 1).

We completed 72 reviews representing 84 country-grain combinations.

### Document Collection

Documents included in the analysis were primary sources collected in a variety of ways. First, we gathered documents from internal databases within partner agencies, such as the Food Fortification Initiative (FFI), the Global Alliance for Improved Nutrition (GAIN), Project Healthy Children (PHC), and Nutrition International, that support countries with their fortification programs. Second, we sent requests for documents to contacts for all countries mandating grain fortification in September 2014 and September 2015 to fill any known gaps.^4^^,^^26^ We also emailed and called Ministries of Health and other relevant agencies within countries to procure documents. If documents were in a language other than English or Spanish, they were translated to English.

### Framework Development

To evaluate the contents of a set of documents, where a set is the legislation, standards, and/or monitoring guidelines for a given country-grain combination, we developed a checklist of items that should be ideally included in a set of fortification documents.^1^^,^^8^^–^^23^ We determined the checklist items through a literature review conducted in February and March 2015 using PubMed and Google Scholar (keywords included combinations of food fortification, legislation, standards, monitoring, evaluation, framework). The checklist was then revised by content experts, who also suggested pertinent studies for consideration. As part of the literature review, studies of legislation surrounding mandatory or voluntary fortification of any food vehicle were included, specifically studies of legislation for industrial fortification (as opposed to home-based fortification, which employs the use of a supplement, not fortified food). Legislative frameworks for fortification and case studies of legislation development or monitoring procedures were also included. Manuals for legislation, standards, and enforcement of food law and fortification, as well as manuals developed for implementing components of a fortification program, were also included in the literature review. Lastly, we included any studies that contained suggestions for model laws or any studies on monitoring of fortification programs.

We piloted the criteria checklist with a subset of 11 country-grain combinations. Following the pilot, we pared down the checklist and focused on items relevant to fortification (as opposed to general food control measures). The final version of the criteria checklist contained 44 items categorized as general, micronutrients/premix, costing, labeling, internal monitoring, external monitoring, commercial monitoring, import monitoring, enforcement/penalties, laboratory testing, and reporting (Table 1).

**TABLE 1.** Checklist of Key Items^a^ and All Possible Scoring Options in Fortification Legislation, Standards, and Monitoring Documents

Item	Scoring Options	References
**General**		
1. States that legislation applies to at least one food vehicle fit for human consumption (types/grades to be fortified)	(2) States at least one type fit for human consumption (0) Does not state	^10, 12, 17, 22^
2. States the public health objective; purpose and scope of legislation	(2) States the public health objective or general purpose of legislation (0) Does not state	^10, 13, 17, 22^
3. References latest available science or accepted international norms and recommendations, particularly for items that may not be covered in the country's documents	(2) States the documents referenced (0) Does not state	^12, 17, 21^
4. Provides definitions that include terms that are specific to fortification (e.g., fortified food, premix, fortificant, food vehicle)	(2) States at least one term related to fortification (0) Does not state	^17, 22^
5. Provides repeals (if there is at least one prior document about fortification)	(2) States repeals (0) Does not state (N/A) No prior documents about fortification	^12, 17^
6. Provides effective date or gives grace period for when fortification is to begin (e.g., effective 6 months from signing)	(2) States effective date or grace period for when fortification is to begin (e.g., effective 6 months from signing) (0) Does not state	^12, 17^
**Micronutrients/Premix**		
7. States nutrients required	(2) States nutrients (0) Does not state	^8, 10, 12, 13, 21^
8. States fortificants (chemical compounds) to be used (including fortificants that are allowable as options)	(2) States fortificants for at least one nutrient (0) Does not state	^8, 10, 12, 13, 21^
9. States fortification levels	(2) States a range or number with +/- (1) States one number only (0) Does not state	^8, 10, 13, 21^
10. States consideration of bioavailability/biological activity of fortificants	(2) States some consideration of bioavailability (mentions these or related terms) (0) Does not state any consideration	^9^
11. States consideration of nutrient stability	(2) States consideration of nutrient stability (0) Does not state any consideration	^11^
**Costing**		
12. States that the cost of fortification is regulated through cost-sharing schemes (between government, industry, consumers) or tax measures (to assist industry)	(2) States consideration of either cost regulation method (0) Does not state any consideration	^10, 12, 13^
13. States consideration of the financial responsibility (of the government) of monitoring and enforcing fortification (schedule of fees, budget)	(2) Shows consideration that monitoring costs money (0) Does not state any consideration	^10, 13, 19, 21, 22^
**Labeling**		
14. Includes some sort of statement/label/logo that makes it clear that the product is fortified	(2) Includes a statement, label, or logo (0) Does not include statement, label, or logo	^8, 10, 12, 13, 18^
15. Provides guidance on health claims that can be made for this product (specific to micronutrients added through fortification)	(2) Provides guidance on health claims specific to micronutrients added through fortification (0) Does not provide	^12, 15, 20^
**Internal Monitoring (Conducted by Industry)**		
16. States requirement for sampling as part of internal monitoring (e.g., describing number of samples, amount, frequency, individual vs. composite, where samples are taken in the process, and percent considered passing)	(2) States that samples should be taken as part of internal monitoring (1) States that samples should be taken (generally) (0) States that samples should not be taken (N/A) Does not describe the sampling process	^1, 8, 12, 22, 23^
17. States that industry is required to follow quality assurance/quality control in regards to fortification	(2) States requirement of quality assurance/quality control for fortification (0) Does not state requirement	^1, 8, 12, 22, 23^
18. States applicability of using qualitative testing (e.g., spot tests, iChecks) to determine the presence or absence of a vitamin or mineral	(2) States applicability of spot test to determine presence/absence of vitamin or mineral specific to internal monitoring (1) States applicability of spot test to determine presence/absence of vitamin or mineral generally (0) Does not state	^11, 21^
**External Monitoring (Conducted by Government)**		
19. States requirement for external monitoring at the production site to assure compliance with standards and regulations	(2) States requirement for external monitoring or the need for audits/inspections (0) Does not state requirement	^1, 9, 10, 12, 15, 17, 18, 21, 22, 23^
20. Describes protocols and systems for regulatory monitoring	(2) Includes checklists or provides detailed description of regulatory monitoring procedures (1) Does not explicitly describe, but references protocols and systems for regulatory monitoring (0) Does not describe	^1, 13, 21^
21. If there are two or more government agencies involved in external monitoring, clarifies the roles and responsibilities between different government agencies in external monitoring	(2) Clarifies roles and responsibilities for more than one agency (1) Clarifies roles and responsibilities for one agency (0) Clarifies roles and responsibilities for no agencies (N/A) Only one government agency involved	^12, 21, 23^
22. Allows for monitoring to be conducted often enough that problems can be identified and addressed on a timely basis; specifies a timeline for inspections (e.g., once every 6 months, increasing to once every 2 months if a discrepancy is found)	(2) Describes frequency and how it is responsive to the needs of industry or the stage of fortification implementation (1) Makes mention of a timeline (0) Does not state	^1, 16, 18, 23^
23. States requirement for sampling as part of external monitoring (e.g., describing number of samples, amount, frequency, individual vs. composite, where samples are taken in the process, and percent considered passing)	(2) States that samples should be taken as part of external monitoring (1) States that samples should be taken (generally) (0) States that samples should not be taken (N/A) Does not describe the sampling process	^1, 8, 12, 22, 23^
24. States applicability of using qualitative testing (e.g., spot tests, iChecks) to determine the presence or absence of a vitamin or mineral	(2) States applicability of spot test to determine presence/absence of vitamin or mineral specific to external monitoring (1) States applicability of spot test to determine presence/absence of vitamin or mineral generally (0) Does not state	^21^
25. States registration is required in order to use a logo/be licensed to produce fortified foods	(2) Describes some type of registration or licensing (0) Does not state that registration or licensing is required	^17, 18^
**Commercial Monitoring (Conducted by Government)**		
26. Provides justification for commercial monitoring at retail stores	(2) Provides justification for commercial monitoring (0) Does not provide justification for commercial monitoring	^21^
27. Describes protocols and systems for commercial monitoring	(2) Includes checklists or provides detailed description of commercial monitoring procedures (1) Does not explicitly describe, but references protocols and systems for commercial monitoring (0) Does not describe	^1, 13, 21^
28. If there are two or more government agencies involved in commercial monitoring, clarifies the roles and responsibilities between different government agencies in commercial monitoring	(2) Clarifies roles and responsibilities for more than one agency (1) Clarifies roles and responsibilities for one agency (0) Clarifies roles and responsibilities for no agencies (N/A) Only one government agency involved	^12, 21, 23^
29. Allows for monitoring to be conducted often enough that problems at the production site or import companies can be identified and addressed on a timely basis; specifies a timeline for inspections (e.g., once every 6 months) or works with production companies to correct noncompliance	(2) Describes frequency and how it is responsive to the needs of industry or the stage of fortification implementation (1) Makes mention of a timeline (0) Does not state (N/A) No commercial monitoring occurs	^1, 16, 18, 23^
30. States requirement for sampling as part of commercial monitoring (e.g., describing number of samples, amount, frequency, individual vs. composite, where samples are taken in the process, and percent considered passing)	(2) States that samples should be taken as part of commercial monitoring (1) States that samples should be taken (generally) (0) States that samples should not be taken (N/A) Does not describe the sampling process	^1, 8, 12, 22, 23^
**Import Monitoring (Conducted by Government)**		
31. Provides justification for import monitoring at points of entry	(2) Provides justification for import monitoring (0) Does not provide justification for import monitoring	^21^
32. Describes protocols and systems for import monitoring	(2) Includes checklists or detailed description of import monitoring procedures (1) Does not explicitly state, but references protocols and systems for import monitoring (0) Does not state	^1, 13, 21^
33. If there are two or more government agencies involved in import monitoring, clarifies the roles and responsibilities between different government agencies in import monitoring	(2) Clarifies roles and responsibilities for more than one agency (1) Clarifies roles and responsibilities for one agency (0) Clarifies roles and responsibilities for no agencies (N/A) Only one government agency involved	^12, 21, 23^
34. States requirement for sampling as part of import monitoring (e.g., describing number of samples, amount, frequency, individual vs. composite, where samples are taken in the process, and percent considered passing)	(2) States that samples should be taken as part of import monitoring (1) States that samples should be taken (generally) (0) States that samples should not be taken (N/A) Does not describe the sampling process	^1, 8, 12, 22, 23^
**Enforcement/Penalties**		
35. Indicates roles and responsibilities in enforcing the legislation	(2) States the role and responsibilities of government in enforcement (0) Does not state	^14, 17, 22^
36. States incentives to start fortification	(2) States any incentives to encourage fortification initiation (e.g., tax incentives for new equipment or premix) (0) Does not state	^13, 17, 22^
37. States incentives to continue fortification, including ensuring compliance	(2) States any incentives to encourage the continuation of fortification (e.g., transport priority, favorable tax or tariff treatment, or patent rights) (0) Does not state	^13, 17, 22^
38. States penalties to compel compliance	(2) States any penalties (0) Does not state	^12, 13, 14, 17, 22^
39. Penalties are objectively defined (e.g., first penalty=$100, second penalty=$300)	(2) Penalties are objectively laid out in the document (e.g., first penalty=$100, second penalty=$300) (0) Penalties are not objectively laid out (N/A) No penalties are stated (answered 0 to previous question)	^10^
40. States that enforcement is required to include feedback and support to improve performance and correct noncompliance	(2) Requires any feedback/support to improve performance (0) Does not require	^1, 10, 17, 18, 21, 23^
**Laboratory**		
41. References required analytical assays for nutrients (e.g., liquid chromatography-mass spectrometry for folic acid, atomic absorption for iron and zinc)	(2) References required assays (0) Does not state requirements	^8^
42. States recognition that laboratory results are subject to several sources of variation and do not provide conclusive evidence of compliance or noncompliance	(2) States recognition that lab results are subject to variation (0) Does not state recognition	^21^
43. Focuses on the quantitative analysis of "marker" micronutrients such as iron	(2) Focuses on quantitative analysis of marker micronutrient such as iron (0) Does not state	^12, 21, 23^
**Reporting**		
44. States how government monitoring results are shared with stakeholders	(2) States how results are shared with stakeholders (0) Does not state how results are shared	^17^

a As identified in the literature and by content experts.

We developed a checklist of 44 items that should be ideally included in a set of fortification documents.

### Document Review

Two reviewers independently reviewed a set of documents for a given country-grain combination and came to a consensus on the scoring of the 44 items. All coauthors conducted the reviews (8 reviewers in total); the 3 reviewers involved in the development of the checklist and the pilot (KJM, CLL, HP) were paired with the other 5 reviewers to ensure consistency across reviews. Three reviewers (XL, LMD-R, HP) reviewed the Spanish-language sets in Spanish. Reviewers completed the checklist by scoring each item using “does not contain item” or “contains item in its totality.” About one-third of items on the checklist (n=15) also had “contains item to some degree” as an option. One-quarter of items on the checklist (n=10) had “not applicable” as an option. There was a comment field adjacent to each item for qualitative observations. If there was a discrepancy in the scoring between reviewers that the pair could not come to consensus on, a third-person arbiter resolved it.

### Country Outreach

After we completed reviews in June 2016, we reached out to in-country contacts, particularly National Fortification Alliance members, via email for all countries included in the review to confirm that all appropriate documentation was included in the review and that preliminary reviews seemed accurate for the given documentation. If documentation was missing and then sent by contacts, we completed a second review including the additional documentation.

### Data Analysis

For each item in the checklist, we calculated the percentage of countries with documentation that fully contained that item. Country-grain combinations receiving a score of “not applicable” for an item were removed from the denominator for that item. We also examined differences in scores by grain, by region,^27^ and by income level.^28^ We conducted a sensitivity analysis, using chi-square tests, to gauge whether the completeness of the documentation reviewed differed between those countries that responded to the country outreach efforts and verified completeness of documentation versus those that did not. Qualitatively, we extracted clear and flexible passages from documents that illustrated language that fully contained each item of interest.

## RESULTS

Of the 72 country-grain combinations reviewed, 55 (76%) were of wheat flour, 11 (15%) of maize flour, and 6 (8%) of rice. The majority of documentation came from countries in the Americas (46%, n=33) and Africa (32%, n=23). Among those with wheat flour documentation, the countries were mainly from the Americas (42%, n=23) and Africa (33%, n=18), with Europe (13%, n=7), the Pacific (5%, n=3), Asia (4%, n=2), and the Middle East (4%, n=2) contributing a smaller proportion. Among those with maize flour documentation, 55% was from the Americas (n=6) and 45% from Africa (n=5). The majority of rice documentation was also from the Americas (67%, n=4), though the Asia and Pacific regions also contributed documentation (17%, n=1 each). The majority of documentation came from upper middle-income countries (33%, n=24) and lower middle-income countries (40%, n=29), with low-income countries contributing 13% (n=9) and high-income countries contributing 14% (n=10) of documentation (based on World Bank classification of countries by income^28^). Most documentation was originally in English (42%, n=30) or Spanish (35%, n=25).

Most of the documentation we reviewed came from countries in the Americas and Africa.

**FIGURE 2:**
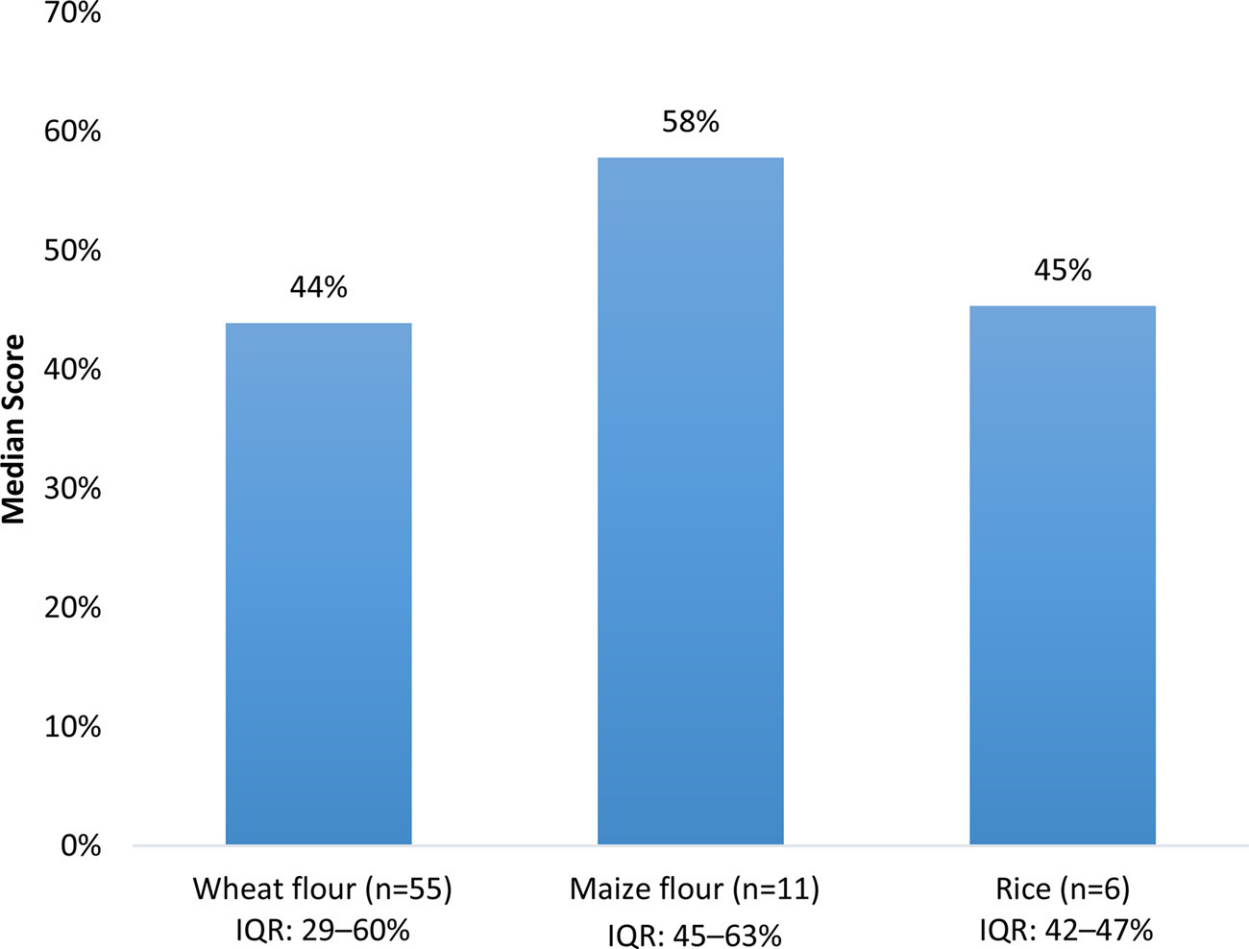
Median Country-Grain Combination Scores^a^ by Cereal Grain in Countries With Mandatory Cereal-Grain Fortification Abbreviation: IQR, interquartile range. ^a^ Country-grain combination refers to the unit of analysis; countries that mandate the fortification of multiple cereal grains will contribute more than one country-grain combination (e.g. Philippines-wheat and Philippines-rice). Scores based on number of checklist items fully documented out of total applicable checklist items.

**FIGURE 3:**
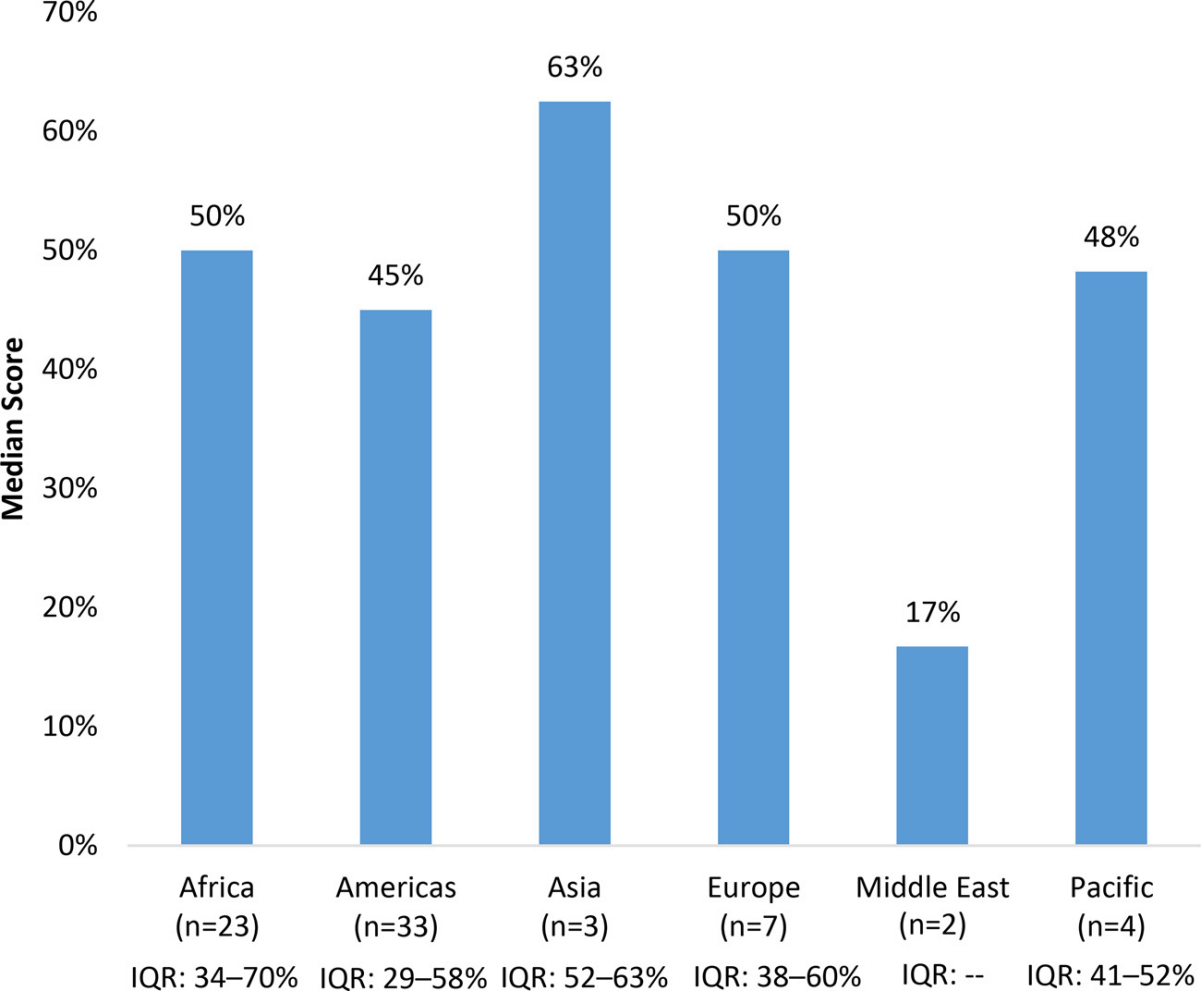
Median Country-Grain Combination Scores^a^ by Geographic Region in Countries With Mandatory Cereal-Grain Fortification Abbreviation: IQR, interquartile range. ^a^ Country-grain combination refers to the unit of analysis; countries that mandate the fortification of multiple cereal grains will contribute more than one country-grain combination (e.g. Philippines-wheat and Philippines-rice). Scores based on number of checklist items fully documented out of total applicable checklist items.

**FIGURE 4:**
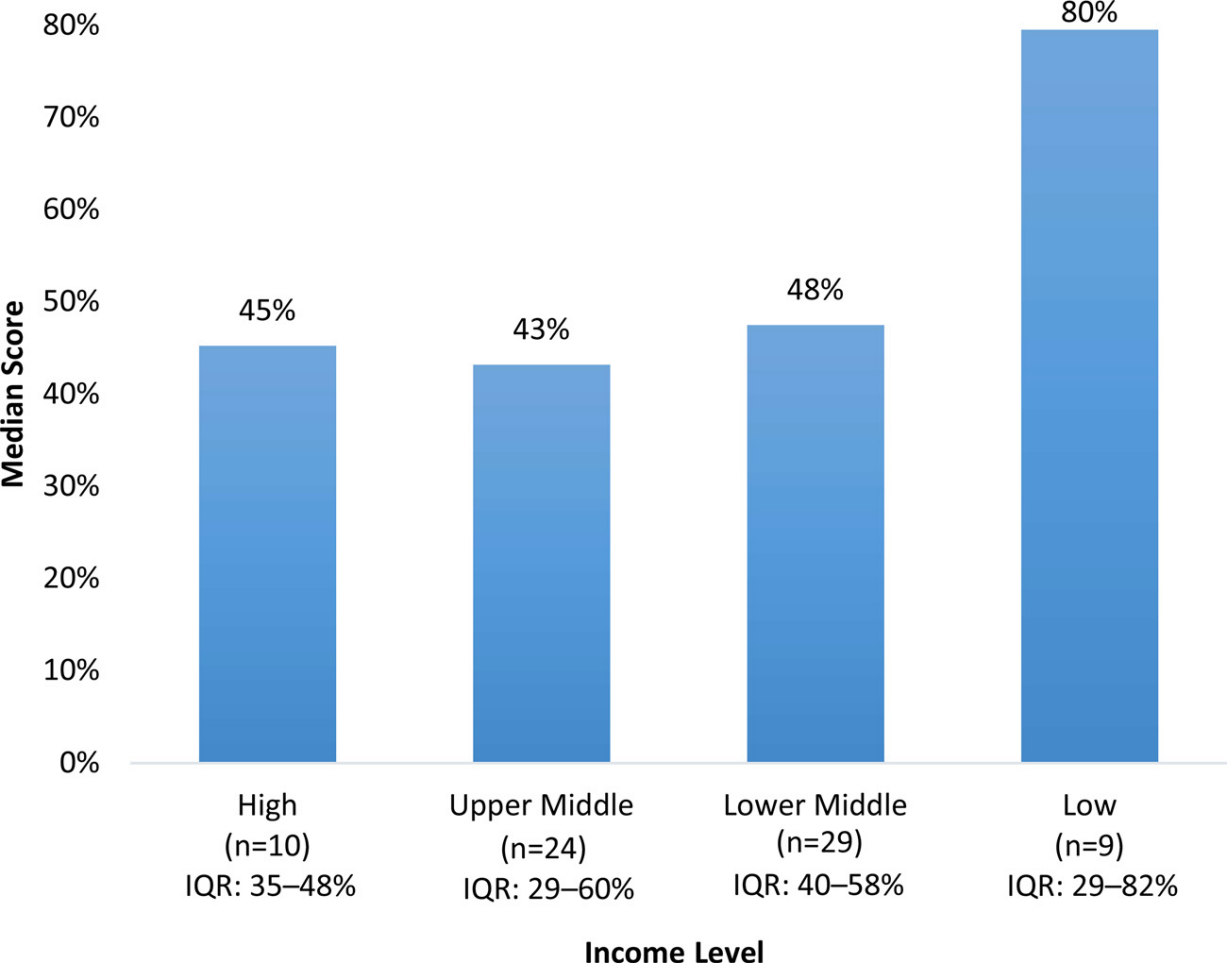
Median Country-Grain Combination Scores^a^ by Income Level in Countries With Mandatory Cereal-Grain Fortification Abbreviation: IQR, interquartile range. ^a^ Country-grain combination refers to the unit of analysis; countries that mandate the fortification of multiple cereal grains will contribute more than one country-grain combination (e.g. Philippines-wheat and Philippines-rice). Scores based on number of checklist items fully documented out of total applicable checklist items.

On average, 46% of checklist items were fully present in reviews. When examining median scores by grain, maize flour scores were higher (58%) than rice (45%) and wheat flour (44%) scores (Figure 2). The median scores by region showed some variability, with Asia scoring the highest (63%), followed by Africa (50%), Europe (50%), the Pacific (48%), the Americas (45%), and the Middle East (17%) (Figure 3). Comparing scores by income classification, the median scores for high income (45%), upper middle-income (43%), and lower middle-income (48%) were very similar, while low-income countries had a notably higher median score (80%) (Figure 4).

On average, 46% of checklist items were fully present in our document review.

Documentation for every country-grain combination (100%, N=72) stated the nutrients required to be added through fortification (Table 2). The fortification levels of those nutrients were stated by 96% of country-grain combinations; 54% stated one number, whereas 42% stated an allowable range. The majority (88%) of country-grain combinations stated at least one fortificant to be used for fortification (e.g., type of iron compound). Documentation for country-grain combinations was also relatively abundant for other general items commonly found in legislation and standards documents: stating the food vehicle to be fortified (97%), labeling as fortified food (78%), providing definitions to terms specific to fortification (76%), and providing the effective date or giving a grace period for when fortification is to begin (72%).

**TABLE 2.** Percentage of Country-Grain Combinations^a^ With Documented Items in Fortification Legislation, Standards, and Monitoring Documents (N=72)

Item	Eligible (N)	% (n) Fully Meeting	% (n) Partly Meeting	% (n) Not Meeting
**General**				
1. Food vehicle stated in legislation	72	97% (70)	–	3% (2)
2. Public health objective/purpose	72	69% (50)	–	31% (22)
3. Accepted international norms	72	54% (39)	–	46% (33)
4. Definitions specific to fortification	72	76% (55)	–	24% (17)
5. Repeals of prior documentation^b^	59	71% (42)	–	29% (17)
6. Effective date/grace period	72	72% (52)	–	28% (20)
**Micronutrients/Premix**				
7. Nutrients required	72	100% (72)	–	0% (0)
8. Fortificants (chemical compounds)	72	88% (63)	–	13% (9)
9. Fortification levels	72	42% (30)	54% (39)	4% (3)
10. Bioavailability of fortificants	72	31% (22)	–	69% (50)
11. Nutrient stability	72	54% (39)	–	46% (33)
**Costing**				
12. Cost sharing of fortification	72	19% (14)	–	81% (58)
13. Financial responsibility of monitoring and enforcement	72	35% (25)	–	65% (47)
**Labeling**				
14. Labeling required	72	78% (56)	–	22% (16)
15. Guidance on health claims	72	50% (36)	–	50% (36)
**Internal Monitoring (conducted by industry during production)**				
16. Sampling process outlined^b^	31	71% (22)	29% (9)	0% (0)
17. Industry QA/QC justified/required	72	64% (46)	–	36% (26)
18. Applicability of qualitative tests	72	29% (21)	1% (1)	69% (50)
**External Monitoring (conducted by government at production sites)**				
19. External monitoring justified	72	64% (46)	–	36% (26)
20. Protocols and systems described	72	33% (24)	28% (20)	39% (28)
21. Roles and responsibilities clarified^b^	56	45% (25)	7% (4)	48% (27)
22. Timeline for inspections outlined	72	26% (19)	13% (9)	61% (44)
23. Sampling process outlined^b^	45	67% (30)	33% (15)	0% (0)
24. Applicability of qualitative tests	72	19% (14)	1% (1)	79% (57)
25. Registration requirements	72	38% (27)	–	63% (45)
**Commercial Monitoring (conducted by government at market or distribution sites)**				
26. Commercial monitoring justified	72	47% (34)	–	53% (38)
27. Protocols and systems described	72	19% (14)	21% (15)	60% (43)
28. Roles and responsibilities clarified^b^	63	32% (20)	0% (0)	68% (43)
29. Timeline for inspections outlined^b^	44	14% (6)	25% (11)	61% (27)
30. Sampling process outlined^b^	28	71% (20)	29% (8)	0% (0)
**Import Monitoring (conducted by government at ports/points of entry)**				
31. Import monitoring justified	72	64% (46)	–	36% (26)
32. Protocols and systems described	72	35% (25)	26% (19)	39% (28)
33. Roles and responsibilities clarified^b^	59	42% (25)	2% (1)	56% (33)
34. Sampling process outlined^b^	29	62% (18)	38% (11)	0% (0)
**Enforcement/Penalties**				
35. Enforcement roles and responsibilities clarified	72	69% (50)	–	31% (22)
36. Incentives to start fortification	72	14% (10)	–	86% (62)
37. Incentives to continue fortification	72	10% (7)	–	90% (65)
38. Penalties to compel compliance	72	68% (49)	–	32% (23)
39. Penalties objectively defined^b^	49	31% (15)	–	69% (34)
40. Enforcement includes feedback	72	18% (13)	–	82% (59)
**Laboratory**				
41. Analytical methods identified	72	60% (43)	–	40% (29)
42. Recognition of laboratory variation	72	11% (8)	–	89% (64)
43. Quantitative analysis of "marker" micronutrients such as iron	72	36% (26)	–	64% (46)
**Reporting**				
44. Dissemination of monitoring results described	72	31% (22)	–	69% (50)

Abbreviations: QA/QC, quality assurance/quality control.

a Country-grain combination refers to the unit of analysis; countries that mandate the fortification of multiple cereal grains will contribute more than one country-grain combination (e.g., Philippines-wheat and Philippines-rice).

b The number eligible differs for these items due to a “not applicable” option on the scoring checklist.

Documentation of monitoring procedures among country-grain combinations was less common (Table 2). About two-thirds (64%) of country-grain combinations stated that industry is required to conduct quality assurance/quality control as part of internal monitoring and 29% described the applicability of qualitative (spot) tests. Of countries that included a sampling protocol, 71% of country-grain combinations clearly outlined a sampling process for internal monitoring. The requirement for external monitoring at the production site was documented for 64% of country-grain combinations; 33% of country-grain combinations provided a detailed description of external monitoring protocols and systems. The same pattern held for commercial and import monitoring: while 47% and 64% of country-grain combinations stated a requirement for commercial and import monitoring, respectively, 19% and 35% described the protocols for commercial and import monitoring in detail.

While 64% of country-grain combinations documented the requirement for external monitoring at the production site, only 33% provided a detailed description of the monitoring protocols and systems.

Similarly, 68% of country-grain combinations stated there were penalties to compel compliance, yet of these, only 31% laid out objectively defined penalties (e.g., first penalty=$100, second penalty=$300) (Table 2). Furthermore, only 18% of country-grain combinations stated that enforcement should include feedback and support to improve performance and correct noncompliance. Very few (14%) country-grain combinations stated that they provide incentives to start fortification (e.g., reduction of taxes for fortification equipment) and fewer (10%) provided incentives to continue fortification (e.g., reduction of taxes for fortification premix).

Other gaps in documentation existed in regards to the cost of monitoring fortification and laboratory procedures (Table 2). For example, only 35% stated consideration of the financial responsibility of monitoring and enforcing fortification (e.g., cost of laboratory testing; explicit budget for monitoring activities). Furthermore, only 19% of country-grain combinations stated that the cost of fortification is regulated through cost-sharing schemes between government and industry or reduced taxes for fortification inputs. Regarding laboratory procedures, while 60% of country-grain combinations referenced the required analytical assays for measuring nutrients in food, only 36% of country-grain combinations focused on the quantitative analysis of a marker micronutrient(s) such as iron, which limits the amount of laboratory analysis required but accurately assesses fortification since fortificants are typically added together in premix. Even fewer (11%) explicitly recognized that laboratory results are subject to several sources of variation and do not alone provide conclusive evidence of compliance. Lastly, results showed that less than one-third (31%) stated how government monitoring results are shared with stakeholders, including consumers.

Less than one-third of country-grain combinations stated how government monitoring results are shared with stakeholders.

A sensitivity analysis investigated differences between reviews that were considered complete (i.e., country representatives confirmed that all relevant documents were included in the reviews) versus those that were unconfirmed in regards to completeness (Supplement Table 2). We observed few differences between the subset of 23 reviews that were confirmed by country representatives and those that were not. Differences were noted for 5 of 44 items: stating the food vehicle to be fortified, describing the sampling process for internal monitoring, stating the applicability of qualitative testing in internal monitoring, clarifying roles and responsibilities in external monitoring, and requiring enforcement to include feedback to those monitored.

We identified what we considered excellent examples of each of the 44 checklist items from English-language and English-translated documents (Supplement Table 3). We compiled completed checklists for each country-grain combination, including extracted sample language from the documents reviewed (Supplement Table 4).

## DISCUSSION

To our knowledge, this is the first comprehensive review of legislation, standards, and monitoring documents for any fortified food, and specifically for fortified grains. Our study provides a standardized checklist of 44 key criteria that the literature suggests are important components of fortification legislation, standards, and monitoring documents, which was in turn employed on a global level to systematically measure the documentation of each country-grain combination and to evaluate country policies against these criteria. We found that countries document the technical specifications for fortification, such as allowable food vehicles and nutrients required, and most document systems for internal, external, and import monitoring. However, documentation is lacking in some areas, such as describing the roles and responsibilities for monitoring between governmental agencies, providing detailed descriptions of protocols and systems for monitoring, addressing the costs of fortification and fortification monitoring, outlining enforcement strategies, and describing how government monitoring results are reported to stakeholders. Lack of documentation persisted largely around the areas that would influence product compliance to national standard, while sufficient documentation existed around areas that establish a mandatory program. This is important to highlight, as it is a more complex piece that needs to be carefully outlined (e.g., assigning clear roles and responsibilities for regulatory monitoring, establishing detailed protocols for conducting regulatory monitoring) if a program is going to be monitored for long-term adherence and impact.^7^^,^^21^ Given these gaps, our checklist could be used as a guide to strengthen existing documentation or assist in developing new documentation, as this would ensure important areas, such as product compliance, are outlined.

Most countries lack documentation around the areas that would influence product compliance to national standard.

Our study found similar results as a review of legislative frameworks for corn flour and maize meal fortification.^8^ Similar to our study, Makhumula and colleagues found that legislative and standards documents commonly describe fortificants used, fortification labeling, reference analytical assays, and sampling procedures.^8^ In our review, 100% (n=11) of maize flour countries documented the fortificants to be used and 91% (n=10) of maize flour countries clearly labeled their maize flour as fortified. Of the 4 maize flour countries that stated the use of sampling in external and import monitoring, 3 provided details on sampling procedures for external and import monitoring. The majority of maize flour countries referenced the required analytical assays for nutrients (73%, n=8) in our review. Differences observed might be because the checklist for our study was specific to fortification, whereas Makhumula and colleagues' review was not limited to fortification. In particular, this might explain any differences observed in sampling procedures and analytical assays; sampling and laboratory testing could include food safety parameters in Makhumula and colleagues' review.

One notable conclusion from Makhumula and colleagues was that countries fortifying maize flour take a variety of approaches to setting fortification levels, such as specifying the minimum amount required or an allowable range.^8^ In our study, a slight majority (55%, n=6) of maize flour countries provide a range or a single number within an allowable range, while 45% (n=5) of countries provide a single number only. Opposing results were found for wheat flour and rice in this study: 40% (n=22) of wheat flour and 33% (n=2) of rice countries provide a range or single number within an allowable range, while 53% (n=29) of wheat flour and 67% (n=4) of rice countries provide a single number only (the remaining 7% (n=4) of wheat flour countries did not state fortification levels). These results confirm Makhumula's maize flour findings and suggest there are inconsistencies between food vehicles and countries in stating fortification levels, and clarity is needed on this issue.

A 2015 survey by Luthringer and colleagues highlights the gap between legislation, standards, and monitoring documents and barriers in the monitoring of fortified foods identified by regulatory agencies and the food industry.^7^ Gaps identified in the documentation reviewed in our study support the survey results reported by Luthringer et al. For example, when asked in the survey to prioritize regulatory monitoring elements needing improvement to ensure compliance against national fortification standards, industry respondents prioritized incentives and penalties for enforcement, both areas of weakness in the documentation reviewed in our study (only 10% of documentation stated incentives are offered to continue fortification and 21% objectively defined penalties). While incentives are rarely documented, Luthringer and colleagues' study suggests that both regulatory agency and industry respondents believed that incentives could encourage compliance with fortification regulations. Survey results also indicated that only slightly more than half of regulatory agencies report regularly sharing their data with stakeholders. In the documentation reviewed in our study, only 31% of country-grain combinations require government monitoring results to be reported. Together, these results suggest that few countries require results to be reported, while slightly more claim to report results. The benefits of a requirement to report results could be written into official documentation, compelling the reporting of results to stakeholders and creating an accountability structure. Lastly, Luthringer et al.'s study identified the lack of clarity in the roles of government authorities as a barrier to effective monitoring; our study found a similar lack of clarity in the documentation.^7^ When two or more agencies were involved, roles and responsibilities were only clarified in 32% to 45% of documentation, dependent on monitoring type, indicating that this is an area of documentation that could be strengthened. On the whole, the opinions expressed by regulatory agency and industry respondents in the survey conducted by Luthringer et al. are confirmed by the present study. Not only do the survey respondents think that documentation is unclear but also our study confirms that documentation is generally lacking in the areas identified by the survey respondents.

While our study aligns with previous studies of fortification legislation, standards, and monitoring documents, it also highlights concerns that previous studies have not raised.^7^^,^^8^^,^^10^^,^^21^^,^^23^^,^^24^ For instance, the majority of previous studies have not reviewed the content of monitoring documents.^7^^,^^8^^,^^10^^,^^24^ Our study found that the details included in monitoring documents vary widely—some countries do not have any monitoring procedures in their dossier of documentation, while other countries have a unique manual for each type of monitoring. In particular, our study identifies the lack of documented protocols and systems in external, commercial, and import monitoring (39%, 60%, and 39%, respectively). Our study corroborates a previous study by van den Wijngaart et al. (2013)^21^ that identified the issue of poorly established or weakly designed protocols and systems for regulatory monitoring of salt and wheat flour fortification in countries of the Association of Southeast Asian Nations (ASEAN); our study found that for the 3 types of regulatory monitoring, 21% to 28% of country-grain combinations did not explicitly describe their monitoring protocols (scored as “partly meeting”). While the van den Wijngaart et al. study and our study are in agreement about the variable quality of monitoring procedures, our study notes the complete lack of documentation. This finding emphasizes the opportunity to improve upon existing monitoring procedures and the opportunity to implement well-designed monitoring procedures for the first time. Additionally, our study shows infrequent documentation requiring qualitative (spot) tests in internal (29%) and external monitoring (19%). While it is possible that spot tests are used but not documented in monitoring procedures, this result shows an opportunity to expand the use of this simple, fast, and inexpensive method.^11^

There is a lack of documented protocols and systems in external, commercial, and import monitoring.

### Strengths and Limitations

First, an important strength of our study is that such a comprehensive review for any cereal grain and for any fortified food has not previously been done. Previous efforts have focused on reviewing legislation and/or standards, while few have included monitoring documents; our study included all 3 types of documents for a more complete sense of content. Furthermore, each country in our study was objectively scored using a standardized checklist of key items in official fortification legislation, standards, and monitoring documents by two separate reviewers. Other strengths of our study are the inclusion of documents from 84% of countries (68 of 81 countries) that mandated cereal-grain fortification as of January 31, 2015, regardless of the language of the document, and the examination of multiple food vehicles within the cereal-grain family. The checklist we created for this study can be used by countries as a framework for starting a new fortification program or assessing a current fortification program. An additional strength is that this methodology presents a model with which to expand this research to other food vehicles, such as salt and vegetable oils.

The main limitation of our study is the possibility of missing documentation. However, attempts were made to collect missing documentation from country representatives, and a sensitivity analysis showed few differences between those countries where country representatives confirmed documentation and those who did not. It was assumed that the knowledge of and access to documents was complete and up to date for the country representatives that were reached. Furthermore, selection bias may be an issue, as those countries that were excluded due to a lack of documentation likely have less comprehensive legislation, standards, and/or monitoring guidelines than those included. This bias may be particularly problematic when comparing scores by region, as there were 7 excluded countries from the Middle East region (78% of countries in the region), 5 from Africa (22%), and 1 from Asia (25%). It is possible that some details were lost in the translation of the documents into English from some languages, but few documents were not already in English or Spanish. Furthermore, it is possible that the checklist was incomplete and did not include some important items that countries prioritize in their documents. However, this risk is low and was mitigated through an inclusive process of listing, refining, and prioritizing the items by considering the existing literature, expert opinion, and through pilot testing for other items that were in reviewed documents. Lastly, our study only addresses documentation, not implementation; while it seems plausible that countries with good documentation also have good implementation of that documentation (and vice-versa), our study did not address implementation of the documentation reviewed.

## CONCLUSION

In conclusion, our comprehensive review of 72 country-grain combinations found that the majority adequately document the required food vehicles, nutrients, and amounts of fortificants for fortification. Most countries have documented justification of the need for monitoring, but detailed protocols, roles and responsibilities for monitoring between agencies, and systems are not well defined. Furthermore, few countries document strategies for paying for the cost of fortification or alleviating the burden on industry through tax exemption or other economic incentives, which can be important in ensuring the sustainability and success of a fortification program. By identifying areas that are often weak or absent in legislation, standards, and monitoring documents, countries with existing mandatory fortification can improve upon these items in revisions to their documents, while countries that are new to fortification will have a better sense of what to include in their policies and programs from the beginning.

Going forward, this study's checklist can be used by many stakeholders. The in-country representatives of private, civic, and public sectors who oversee fortification activities can use the checklist to assess and revise the documents that guide their country's programs. Organizations that provide technical assistance to countries can use the checklist to find common themes across countries and offer technical assistance through regional workshops, for example, or targeted technical assistance based on countries' specific needs.

## Supplementary Material

17-00427-Marks-SupplementTable1.pdf

17-00427-Marks-SupplementTable2.pdf

17-00427-Marks-SupplementTable4.pdf

17-00427-Marks-SupplementTable3.pdf

## Notes

**Peer Reviewed**

**Funding:** Kristin Marks's time was supported by the Food Fortification Initiative. Corey Luthringer's time was supported by an agreement between GAIN and the Bill & Melinda Gates Foundation. Laird Ruth's time was supported by an agreement between the United States Centers for Disease Control and Prevention (CDC) and McKing Consulting Corporation. Laura Rowe's time was supported by Project Healthy Children. Noor Khan's and Luz Maria De-Regil's time was supported by agreements between Nutrition International and Global Affairs Canada and the Department for International Development UK. Ximena López's time was supported by Granotec Foundation. Helena Pachón's time was supported by an agreement between the United States Centers for Disease Control and Prevention (CDC), McKing Consulting Corporation, and Emory University.

## References

[1] Allen L de Benoist B Dary O Hurrell RF, eds. Guidelines on Food Fortification With Micronutrients. Geneva: World Health Organization and Food and Agriculture Organization; 2006. http://www.who.int/nutrition/publications/guide_food_fortification_micronutrients.pdf. Accessed April 18, 2018.

[2] Blencowe H Cousens S Modell B Lawn J. Folic acid to reduce neonatal mortality from neural tube disorders. Int J Epidemiol. 2010;39(suppl 1):i110–i121. 10.1093/ije/dyq028. 20348114 PMC2845867

[3] Atta CAM Fiest KM Frolkis AD. Global birth prevalence of spina bifida by folic acid fortification status: a systematic review and meta-analysis. Am J Public Health. 2016;106(1):e24–e34. 10.2105/AJPH.2015.302902. 26562127 PMC4695937

[4] Food Fortification Initiative (FFI). Defeating Anemia: 2015 Year in Review. Atlanta, GA: FFI; 2016. http://www.ffinetwork.org/about/stay_informed/publications/documents/FFI2015Review.pdf. Accessed April 18, 2018.

[5] Food Fortification Initiative (FFI). Say Hello to a Fortified Future: 2016 Year in Review. Atlanta, GA: FFI; 2017. http://ffinetwork.org/about/stay_informed/publications/documents/FFI2016Review.pdf. Accessed April 18, 2018.

[6] Zimmerman S Baldwin R Codling K. Mandatory policy: most successful way to maximize fortification's effect on vitamin and mineral deficiency. Indian J Comm Health. 2014;26(suppl S2):369–374. http://www.iapsmupuk.org/journal/index.php/IJCH/article/view/958. Accessed April 18, 2018.

[7] Luthringer CL Rowe LA Vossenaar M Garrett GS. Regulatory monitoring of fortified foods: identifying barriers and good practices. Glob Health Sci Pract. 2015;3(3):446–461. 10.9745/GHSP-D-15-00171. 26374804 PMC4570017

[8] Makhumula P Dary O Guamuch M Tom C Afidra R Rambeloson Z. Legislative frameworks for corn flour and maize meal fortification. Ann N Y Acad Sci. 2014;1312(1):91–104. 10.1111/nyas.12349. 24521440

[9] Allen LH. New approaches for designing and evaluating food fortification programs. J Nutr. 2006;136(4):1055–1058. 10.1093/jn/136.4.1055. 16549476

[10] Dijkhuizen MA Wieringa FT Soekarjo D Van KT Laillou A. Legal framework for food fortification: examples from Vietnam and Indonesia. Food Nutr Bull. 2013;34(2_suppl):S112–S123. 10.1177/15648265130342S113. 24050002

[11] Flour Millers Toolkit. Food Fortification Initiative website. http://www.ffinetwork.org/implement/toolkit.html. Accessed April 18, 2018.

[12] Forsman C Milani P Schondebare JA Matthias D Guyondet C. Rice fortification: a comparative analysis in mandated settings. Ann N Y Acad Sci. 2014;1324(1):67–81. 10.1111/nyas.12453. 24913356

[13] Gayer J Smith G. Micronutrient fortification of food in Southeast Asia: recommendations from an expert workshop. Nutrients. 2015;7(12):646–658. 10.3390/nu7010646. 25608937 PMC4303859

[14] Hemenway D. Monitoring and Compliance: The Political Economy of Inspection. Greenwich, CT: JAI Press; 1985. 10.1002/pam.4050050217

[15] Isabelle M Chan P Wijaya SY. Report on Regulatory Status of Micronutrient Fortification in Southeast Asia. Singapore: International Life Sciences Institute Southeast Asian Region; 2011. http://ilsi.org/publication/report-on-regulatory-status-of-micronutrient-fortification-in-southeast-asia/. Accessed April 18, 2018.

[16] Lawrence M. Challenges in translating scientific evidence into mandatory food fortification policy: an antipodean case study of the folate–neural tube defect relationship. Public Health Nutr. 2005;8(8):1235–1241. 10.1079/PHN2005749. 16372918

[17] Nathan R. Regulation of fortified foods to address micronutrient malnutrition: legislation, regulations and enforcement. 3rd ed. Ottawa, Canada: Micronutrient Initiative; 1999. http://ffinetwork.org/documents/Legislation_Manual.pdf. Accessed April 18, 2018.

[18] Peña-Rosas JP Parvanta I Van Der Haar F Chapel TJ. Monitoring and evaluation in flour fortification programs: design and implementation considerations. Nutr Rev. 2008;66(3):148–162. 10.1111/j.1753-4887.2008.00019.x. 18289179

[19] Sullivan KM. The challenges of implementing and monitoring of salt iodisation programmes. Best Pract Res Clin Endocrinol Metab. 2010;24(1):101–106. 10.1016/j.beem.2009.09.001. 20172474

[20] Tan KYM Beek EM Chan MY Zhao X Stevenson L. Health claims on food products in Southeast Asia: regulatory frameworks, barriers, and opportunities. Nutr Rev. 2015;73(9):634–641. 10.1093/nutrit/nuv029. 26269489

[21] van den Wijngaart A Bégin F Codling K Randall P Johnson QW. Regulatory monitoring systems of fortified salt and wheat flour in selected ASEAN countries. Food Nutr Bull. 2013;34(2_suppl):S102–S111. 10.1177/15648265130342S112. 24050001

[22] Vapnek J Spreij M. Perspectives and Guidelines on Food Legislation, With a New Model Food Law. Rome: Food and Agriculture Organization of the United Nations; 2005. http://www.fao.org/3/a-a0274e.pdf. Accessed April 18, 2018.

[23] Wirth J Nichols E Masd H Barham R Johnson Q Serdula M. External mill monitoring of wheat flour fortification programs: an approach for program managers using experiences from Jordan. Nutrients. 2013;5(12):4741–4759. 10.3390/nu5114741. 24284616 PMC3847758

[24] UNICEF East Asia and Regional Office (EAPRO). Review of National Legislation for Universal Salt Iodisation: South and East Asia and the Pacific. Bangkok: UNICEF EAPRO; 2015. https://www.unicef.org/eapro/Universal_Salt_Iodisation_in_South_and_East_Asia_and_Pacific.pdf.

[25] Caribbean Community Secretariat. Specification for wheat flour. Caribbean Community Standard. CCS 0024: 1992. Barbados, WI: CARICOM Export Development Project; Georgetown, Guyana: Caribbean Community Secretariat. 1995.

[26] Food Fortification Initiative (FFI). New Grain, New Name: 2014 Year in Review. Atlanta, GA: FFI; 2015. http://www.ffinetwork.org/about/stay_informed/publications/documents/FFI2014Review.pdf. Accessed April 18, 2018.

[27] Regional Activity. Food Fortification Initiative website. http://ffinetwork.org/regional_activity/. Accessed April 18, 2018.

[28] World Bank Country and Lending Groups. World Bank website. https://datahelpdesk.worldbank.org/knowledgebase/articles/906519-world-bank-country-and-lending-groups. Accessed April 18, 2018.

