# Supplementary material for: Review of Grain Fortification Legislation, Standards, and Monitoring Documents
Source: Glob Health Sci Pract. 2018 Jun 27;6(2):356–71. doi: 10.9745/GHSP-D-17-00427 (PMC6024620; doi:10.9745/GHSP-D-17-00427)
Supplement: 17-00427-Marks-SupplementTable1.pdf [file 17-00427-Marks-SupplementTable1.pdf]

Marks KJ, Luthringer CL, Ruth LJ, et al. Review of grain fortification legislation, standards, and monitoring documents. *Glob Health Sci Pract*. 2018;6(2). https://doi.org/10.9745/GHSP-D-17-00427

SUPPLEMENT TABLE 1. Characteristics of the Study Sample (N=72 Country-Grain Combinations*)

Mandatorily Fortified Grain**	Country	Region	Documents Reviewed
**Wheat Flour (n=55)**			
	Argentina	Americas	1. Law 25.630 and Regulatory Decree No. 597/2003. On Prevention of Anemia and Neural Tube Malformations. n.p. 2002.<br>2. ANMAT. Control of enrichment of wheat flour with iron and vitamins. Regulation 2280/2005. n.p. 2005.<br>3. Chapter V. Standards for food labeling and advertising. Joint Resolution SPRyRS 149/2005 and SAGPyA 683/2005. n.p. n.d.<br>4. Argentine Food Code. Law 18.284. The hygienic sanitary, bromatological and commercial identification provisions of the Food Regulations approved by Decree No. 141/53, with its amending and complementary norms, are hereby declared applicable throughout the territory of the Republic, under the name of the Argentine Food Code. Argentina: Senasa. 1969.

Mandatorily Fortified Grain**	Country	Region	Documents Reviewed
	Australia	Pacific	1. Food Standards Australia New Zealand (FSANZ). Information for Australia & New Zealand laboratories regarding folic acid proficiency testing program. Canberra, Australia: FSANZ. n.d.<br>2. Federal Register of Legislative Instruments F2015C00179. Standard 1.2.4 labelling of ingredients. Australia: Author. 2015.<br>3. Federal Register of Legislative Instruments F2015C00052. Standard 1.4.1 contaminants and natural toxicants. Australia: Author. 2015.<br>4. Federal Register of Legislative Instruments F2009C00836. Standard 1.2.2 food identification requirements (Issue 100). Australia: Author. n.d.<br>5. Federal Register of Legislative Instruments F2014C01180. Standard 1.2.5 date marking of food. Australia: Author. 2014.<br>6. Federal Register of Legislative Instruments F2014C01190. Standard 2.1.1 cereals and cereal products. Australia: Author. 2014.<br>7. Federal Register of Legislative Instruments F2011C00541. Standard 1.3.2 vitamins and minerals (Issue 124). Australia: Author. n.d.<br>8. Food Standards Australia New Zealand (FSANZ). Australian user guide. Mandatory folic acid fortification. Implementing the requirements of the mandatory fortification with folic acid under Standard 2.1.1 – cereals and cereal products. (n.p.): FSANZ. 2009.<br>9. Implementation Sub Committee Co-ordinated Food Survey Plan. National mandatory folic acid fortification of wheat flour for making bread compliance survey of flour mills producing wheat flour for making bread in October 2010 - March 2011. (n.p.): Author. 2012.
	Belize	Americas	1. Belize Bureau of Standards (BBS). Belize national standard. BZS 2. Specification for wheat flour. Belize City, Belize: BBS. 1998.
	Bolivia	Americas	1. Constitutional President of the Republic. Supreme Decree No. 24.420. Integrated program for the forecasting and control of nutritional anemia. (n.p.): Author. n.d.<br>2. Ministry of Health and Sports. Legislation and regulation of wheat flour, mixtures of flour and fortified products. Series: Normative technical documents. No. 238. La Paz, Bolivia: Graff Press. 2011.

Mandatorily Fortified Grain**	Country	Region	Documents Reviewed
	Brazil	Americas	1. National Health Surveillance Agency (ANVISA). RDC Resolution No. 344. Approve the technical regulations for the wheat flour and corn flour fortification with iron and folic acid, found in the annex to this resolution. Brazil: Official Federal Gazette. 2002.<br>2. Decree No. 1711. n.p. 2012.<br>3. Germani, R., Ascheri, J. L. R., Silva, F. T., Torrezan, R., Silva, K. L., Netto, A. G., & Nutti, M. R. Manual for fortification of wheat flour with iron. Documents 46. Rio de Janeiro, Brazil: Embrapa Agroindústria de Alimentos. 2001.<br>4. The President of the Republic. Law No. 6,437. Sets forth the violations to federal sanitary legislation, establishes their respective penalties, and makes other provisions. n.p. 1977.
	Burkina Faso	Africa	1. Minister of Industry, Commerce and Artisans, Minister of Health, Minister of Economy and Finance, & Minister of Agriculture and Hydraulics. Inter-ministerial order No 2012-0232 / MICA / MS / MEF / MAH obligatory enrichment of refined vegetable oils of vitamin A and of common wheat flour of iron and folic acid. Burkina Faso: Author. 2012.
	Cameroon	Africa	1. Republic of Cameroon. Standards and Quality Agency (ANOR). Cameroon Standard NC 01:2000-03, REV.1 (2011). Wheat flour enriched with iron, folic acid, zinc and vitamin B12. Cameroon: ANOR. 2011.<br>2. Republic of Cameroon. The Minister of Public Health, The Minister of Industry, Mines and Technological Development & The Ministry of Commerce. Joint order no. 2:3 6 9. Making mandatory the standard on wheat flour fortified with iron, folic acid, zinc and vitamin B12. Cameroon: Author. 2011.
	Canada	Americas	1. Division 13 grain and bakery products.<br>B.13.001. [S]. Flour, white flour, enriched flour or enriched white flour. n.p. n.d.<br>2. Food and Drug Regulations. C.R.C., c. 870. Canada: Minister of Justice. 2014.
	Cape Verde	Africa	1. The Republic of Cape Verde. Official Bulletin. Series I Number 46. Cape Verde: Cape Verdean National Press. SA. 2014.

Mandatorily Fortified Grain**	Country	Region	Documents Reviewed
	Caribbean (CARICOM)^ (Antigua and Barbuda, Bahamas, Barbados, Dominica, Grenada, Guyana, Haiti, Jamaica, Saint Kitts and Nevis, Saint Lucia, Saint Vincent and the Grenadines, Suriname, and Trinidad and Tobago)	Americas	1. Caribbean Community Secretariat. Specification for wheat flour. Caribbean Community Standard. CCS 0024: 1992. Barbados, W.I.: CARICOM Export Development Project; Georgetown, Guyana: Caribbean Community Secretariat. 1995.
	Chile	Americas	1. Chile's government, Ministry of Health, Instiitute of Public Health, & SEREMI Health Metropolitan Region. Technical standard for monitoring and supervision of the fortification of wheat flour with vitamins and minerals. Chile: Author. 2011.<br>2. Ministry of Health. Legal Division. Sanitary regulation of food. DTO. No. 977/96. Chile: Author. 2014.<br>3. Ministry of Health, Institute of Public Health, Metropolitan Health Service of the Environment, Center of Human Nutrition U of Chile, Institute of Food Engineering (ICYTAL), & U. Austral. Technical standard for the fortification of wheat flour with vitamins and minerals. n.p. 1999.

Mandatorily Fortified Grain**	Country	Region	Documents Reviewed
	Colombia	Americas	1. Decree Number 1944 of 1996. Which regulates the fortification of wheat flour and establishes the conditions for marketing, labeling, monitoring and control. Colombia: n.p. 1996.<br>2. Llinás, H. A. INVIMA is allowed to inform the community in general. 400-1491-13. Subject: Procedure for import of products whose main ingredients is wheat flour. Bogotá, Colombia: National Institute of Medicine and Food Surveillance. 2013.<br>3. The Colombian Institute of Technical Standards and Certification (ICONTEC), ed. Colombian technical standard. NTC 267. Wheat flour (8th update). Bogotá, D. C., Colombia: ICONTEC. 2013.
	Congo	Africa	1. Ministry of Industrial Development and Private Sector Promotion, Ministry of Health and the Population & Ministry of Commerce and Supplies. Order No. 11236/MDIPSP/MSP/MCS - approving the regulations on iron-fortified wheat flour. Brazzaville, Republic of the Congo: Author. 2013.

Mandatorily Fortified Grain**	Country	Region	Documents Reviewed
	Costa Rica	Americas	1. The President of the Republic and the Minister of Health. Regulation for the enrichment of wheat flour. No. 26371-S. (n.p.): La Gaceta (No. 205). 1997.<br>2. The President of the Republic and the Minister of Health. N° 30030-S. n.p. n.d.<br>3. Institute of Nutrition of Central America and Panama (INCAP), United Nations Children's Fund (UNICEF) & Food and Nutrition Foundation of Central America and Panama (FANCAP). Manual for the external monitoring of wheat flour fortification (Technical Audit and Inspection) (2nd ed.). n.p. 2011.<br>4. Institute of Nutrition of Central America and Panama (INCAP), United Nations Children's Fund (UNICEF) & Food and Nutrition Foundation of Central America and Panama (FANCAP). Manual for the inspection of fortified foods at sales sites. (Commercial monitoring) (2nd ed.). n.p. 2011.<br>5. Ministry of Economy, Salvadoran Agency for Technical Regulation, Ministry of Development, Industry and Commerce, Ministry of Industry and Commerce, Ministry of Economy, Industry and Commerce, eds. Nutrition labeling of prepackaged food products for human consumption for the population from 3 years of age. Central American Technical Regulation. RTCA 67.01.60:10. n.p. n.d.<br>6. Ministry of Economy, National Council of Science and Technology, Ministry of Development, Industry and Commerce, Ministry of Industry and Commerce, Ministry of Economy Industry and Commerce, eds. Flours. Fortified wheat flour. Specifications. Central American technical regulation. RTCA 67.01.15:06. n.p. n.d.
	Côte d'Ivoire	Africa	1. Ministry of Public Health and Hygiene, Ministry of Industry and Promotion of the Private Sector, Ministry of Trade & Minister Delegate to the Prime Minister responsible for the Economy and Finance. Making the iron and folic acid fortification of wheat flour for bread in the Ivory Coast mandatory. Abidjan, Republic of the Ivory Coast: Author. 2007.<br>2. N'dri-Yoman, T. A. To the Director of the Modern Mills of Cote d'Ivoire. Subject: Authorization for the use of ferrous fumarate. N°12980 /MSES/CAB. Abidjan, Republic of the Cote d'Ivoire: Ministry Of Health And From the Fight Against AIDS. 2011.

Mandatorily Fortified Grain**	Country	Region	Documents Reviewed
	Cuba	Americas	1. Cuban National Bureau of Standards. Wheat flour — Specifications (1st ed.). Cuban Standards. NC 877: 2012. Havana, Cuba: National Office of Normalization. 2012.<br>2. Cereals and cereal products — Sampling (1st ed.). Reference number - ISO 24333: 2009(E). Geneva, Switzerland: ISO. 2009.<br>3. Codex Standard for Wheat Flour. Codex Stan 152-1985. n.p. n.d.
	Djibouti	Africa	1. Prime Minister. Minister of Economy and Finance responsible for Industry and Planning, & Ministry Delegate responsibble for Trade, SME's, Handicrafts, Tourism, and Formalisation. On fortification of wheat flour intended for human consumption in the Republic of Djibouti. Djibouti: Author. n.d.
	Dominican Republic	Americas	1. Secretariat of state of public health and social assistance (SESPAS). General law of health. Law No. 42-01. Santo Domingo, Dominican Republic: SESPAS. 2001.<br>2. Dominican Institute for Quality (INDOCAL), ed. Dominican Technical Regulation. Flour. Fortified wheat flour. Specifications (1st ed.).<br>RTD 616-2009. (n.p.): INDOCAL. 2009.<br>3. Ministry of Economy, National Council of Science and Technology, Ministry of Development, Industry and Commerce, Ministry of Industry and Commerce, Ministry of Economy Industry and Commerce, eds. Flours. Fortified wheat flour. Specifications. Central American technical regulation. RTCA 67.01.15:06. n.p. n.d.<br>4. Subsecretariat of State of Health in Nutrition (SESPAS), GAIN & Banco Mundial. Plan for monitoring and evaluation of food fortification in the Dominican Republic. No. 10. Nutrition Collection Series of Plans, Programs and Projects. Santo Domingo, Dominican Republic: SESPAS. 2009.<br>5. Ministry of Economy, National Council of Science and Technology, Ministry of Economy, Industry and Commerce, Ministry of Development, Industry and Commerce & Secretariat of Industry and Commerce, eds. General labeling of prepackaged food (prepackaged). Central American technical regulation. RTCA 67.01.02:10. n.p. n.d.<br>6. Dominican Institute for Quality<br>(INDOCAL), ed. Dominican Technical Regulation. Labeling of prepackaged foods. Nutrition labeling. Regulation for nutrition labeling (1st ed.).<br>RTD 675. (n.p.): INDOCAL. 2011.

Mandatorily Fortified Grain**	Country	Region	Documents Reviewed
	Ecuador	Americas	1. Constitutional President of the Republic, Minister of Public Health, & Secretary General of Public Administration. No. 4139. Considering that according to studies carried out worldwide and recommended by the International Organizations PAHO / WHO UNICEF, the most important deficiencies in micronutrients are: iodine, iron and vitamin A (2nd suppl.). Official Record No. 1.008. Quito, Ecuador: Author. 1996.<br>2. Ministry of Public Health of Ecuador & National Coordination of Nutrition. Guidelines for the implementation of the internal monitoring system of the wheat flour fortification program. (System of guarantee and control of quality AC / DC in plants of production). Quito, Ecuador: Author. 2012.<br>3. Ministry of Public Health of Ecuador & National Coordination of Nutrition. Guidelines for the implementation of the external monitoring system and post-registration control of the wheat flour fortification program (inspection and technical evaluation). (n.p.): Author. 2012.<br>4. Ministry of Public Health. No. 00000564. Considering: That: Executive decree No. 4139, published in Official Gazette No. 1008 dated August 1, 1996, issued the regulations for the fortification and enrichment of Wheat flour in Ecuador for the prevention of nutritional anemias. Quito, Ecuador: n.p. 2011.<br>5. Ecuadorian Institute for Standardization (INEN). Ecuadorian Technical Standard. Wheat flour. Specifications (1st ed.). NTE INEN 616:2006 (3rd Revision). Quito, Ecuador: INEN. 2006.<br>6. Ecuadorian Institute for Standardization (INEN). Ecuadorian Technical Standard. Food products labelling for human consumption. Part 2. Nutritional labelling. Specifications (1st ed.). NTE INEN 1334-2:2011 (2nd Revision). Quito, Ecuador: INEN. 2011.
	Egypt	Africa	1. Global Alliance for Improved Nutrition, United Nations World Food Programme. Quality Assurance (QA) & Quality Control (QC). Rome: Global Alliance for Improved Nutrition, United Nations World Food Programme. 2010.

Mandatorily Fortified Grain**	Country	Region	Documents Reviewed
	El Salvador	Americas	1. Ministry of Economy, National Council of Science and Technology, Ministry of Development, Industry and Commerce, Ministry of Industry and Commerce, & Ministry of Economy Industry and Commerce, eds. Flours. Fortified Wheat Flour. Specifications. Central American technical regulation. RTCA 67.01.15:07. n.p. n.d.<br>2. Ministry of Health. Occupational Safety and Health Committee. Manual of technical procedures for the monitoring and evaluation of the food fortification program (1st update). San Salvador, El Salvador: Author. 2011.<br>3. Ministry of Economy,<br>Salvadoran Agency for Technical Regulation, Ministry of Development, Industry and Commerce, Ministry of Industry and Commerce, & Ministry of Economy, Industry and Commerce, eds. Nutritional labeling of foodstuffs prepared for human consumption for population from 3 years of age. Central American Technical Regulation. RTCA 67.01.60:10. n.p. n.d.
	Fiji	Pacific	1. Food Safety Regulations 2009. Legal Notice No. 20. Food and Safety Act 2003 (No. 10 of 2003). Fiji Islands: n.p. n.d.
	Ghana	Africa	1. Parliament of the Republic of Ghana. Public Health Act, 2012. Act 851. Accra, Ghana: Assembly Press. 2012.<br>2. Minister of Health. Directive for the fortification of all wheat flour and vegetable oil(s) locally produced and/or imported. Accra, Ghana: n.p. n.d.<br>3. Food and Drugs Board. Regulatory monitoring of fortified wheat flour. Guidelines for millers & food control agencies. n.p. 2007.<br>4. Standard specification for fortificant premix for wheat flour. Ghana standard. FDGS 809: 2006. n.p. n.d.<br>5. Cereals and pulses - specification for fortified soft wheat flour. Ghana standard. FDGS 812: 2006. n.p. n.d.<br>6. Cereals and pulses - specification for fortified strong wheat flour. Ghana standard. FDGS 811: 2006. n.p. n.d.

Mandatorily Fortified Grain**	Country	Region	Documents Reviewed
	Guatemala	Americas	1. Ministry of Economy, National Council of Science and Technology, Ministry of Development, Industry and Commerce, Secretariat for Industry and Commerce, & Ministry of Economy Industry and Commerce, eds. Flours. Fortified wheat flour. Specifications. Central American technical regulation. RTCA 67.01.15:07. Annex Resolution No. 201-2007 (Comieco-XLV). n.p. n.d.<br>2. Institute of Nutrition of Central America and Panama, United Nations Children's Fund, & Foundation for Food and Nutrition of Central America and Panama. Manual for the external monitoring of wheat flour fortification (Technical Audit and Inspection) (2nd ed.). n.p. 2011.<br>3. Institute of Nutrition of Central America and Panama, United Nations Children's Fund, & Foundation for Food and Nutrition of Central America and Panama. Manual for the inspection of fortified foods in sales sites (Commercial monitoring) (2nd ed.). n.p. 2011.<br>4. Institute of Nutrition of Central America and Panama, United Nations Children's Fund, & Foundation for Food and Nutrition of Central America and Panama. Manual for the inspection of fortified foods at points of entry of imported foods (2nd ed.). n.p. 2011.<br>5. Institute of Nutrition of Central America and Panama, United Nations Children's Fund, & Foundation for Food and Nutrition of Central America and Panama. Manual for the internal monitoring of wheat flour fortification (Quality assurance and quality control, AC / DC) (2nd ed.). n.p. 2011.<br>6. Ministry of Economy, National Council of Science and Technology, Ministry of Development, Industry and Commerce, Ministry of Industry and Commerce, & Ministry of Economy Industry and Commerce, eds. Flours. Fortified wheat flour. Specifications. Central American Technical Regulation. RTCA 67.01.15:06. n.p. n.d.<br>7. Ministry of Economy, Salvadoran Agency for Technical Regulation, Ministry of Development, Industry and Commerce, Secretary of Industry and Commerce, & Ministry of Economy, Industry and Commerce, eds. Nutrition labeling of prepackaged food products for human consumption for the population from 3 years of age. Central American technical regulation. RTCA 67.01.60:10. n.p. n.d.

Mandatorily Fortified Grain**	Country	Region	Documents Reviewed
	Honduras	Americas	1. Ministry of Economy, National Council of Science and Technology, Ministry of Development, Industry and Commerce, Secretariat for Industry and Commerce, & Ministry of Economy Industry and Commerce, eds. Flours. Fortified wheat flour. Specifications. Central American technical regulation. RTCA 67.01.15:07. Annex Resolution No. 201-2007 (Comieco-XLV). n.p. n.d.<br>2. Institute of Nutrition of Central America and Panama, United Nations Children's Fund, & Foundation for Food and Nutrition of Central America and Panama. Manual for the external monitoring of wheat flour fortification (Technical Audit and Inspection) (2nd ed.). n.p. 2011.<br>3. Institute of Nutrition of Central America and Panama, United Nations Children's Fund, & Foundation for Food and Nutrition of Central America and Panama. Manual for the inspection of fortified foods in sales sites (Commercial monitoring) (2nd ed.). n.p. 2011.<br>4. Institute of Nutrition of Central America and Panama, United Nations Children's Fund, & Foundation for Food and Nutrition of Central America and Panama. Manual for the inspection of fortified foods at points of entry of imported foods (2nd ed.). n.p. 2011.<br>5. Institute of Nutrition of Central America and Panama, United Nations Children's Fund, & Foundation for Food and Nutrition of Central America and Panama. Manual for the internal monitoring of wheat flour fortification (Quality assurance and quality control, AC / DC) (2nd ed.). n.p. 2011.<br>6. Ministry of Economy, National Council of Science and Technology, Ministry of Development, Industry and Commerce, Ministry of Industry and Commerce, & Ministry of Economy Industry and Commerce, eds. Flours. Fortified wheat flour. Specifications. Central American technical regulation. RTCA 67.01.15:06. n.p. n.d.<br>7. Ministry of Economy, Salvadoran Agency for Technical Regulation, Ministry of Development, Industry and Commerce, Secretary of Industry and Commerce, & Ministry of Economy, Industry and Commerce, eds. Nutrition labeling of prepackaged food products for human consumption for the population from 3 years of age. Central American technical regulation. RTCA 67.01.60:10. n.p. n.d.

Mandatorily Fortified Grain**	Country	Region	Documents Reviewed
	Indonesia	Asia	1. Food Sampling Guideline for 2013. n.p. n.d.<br>2. Minister of Industry of the Republic of Indonesia. Regulation of the Minister of Industry of the Republic of Indonesia No. 35/M-IND/PER/3/2011 on obligatory application of the Indonesian national standard (SNI) for wheat flour as a foodstuff. Jakarta, Indonesia: Author. 2011.<br>3. Minister of Industry of the Republic of Indonesia. The Decree of the Ministry of Industry No. 29/M/SK/2/1995 on passage and application of the Indonesian national standards and compulsory use of SNI logo on 10 (Ten) industrial products. Jakarta, Indonesia: Author. 1995.<br>4. National Standardization Body (BSN). Wheat flour as food. Indonesian National Standard. SNI 3751:2009. Jakarta, Indonesia: BSN. n.d.

	Kazakhstan	Europe	1. Ministry of Industry and Trade of the Republic of Kazakhstan. Committee on Technical Regulation and Metrology. Fortified (enriched) baking wheat flour. General specifications (formal ed.). State standard of the Republic of Kazakhstan. Astana, Kazakhstan: Author. 2007.<br>2. Kazakh Academy of Nutrition. Bread and bakery products enriched with vitamin-mineral premix “KAP Complex No1”. ST LLP 40261271-07-2006 (1st ed.). Almaty, Kazakhstan: Author. 2006.<br>3. Kazakh Academy of Nutrition. Vitamin-mineral premix “KAP Complex No1” ST LLP 40261271-01-2006 (1st ed.). Almaty, Kazakhstan: Author. 2006.<br>4. Ministry of Health of the Republic of Kazakhstan. Scientific and practical centre of expertise and monitoring. Information on the progress made in execution of the Order of the Chairman of RSP CSSES # 70 on enhanced control of premium and first grade wheat flour fortification for the first half of 2011. Almaty, Kazakhstan: Author. 2011.<br>5. Union of grain processors and bakers of Kazakhstan. Legislative and regulatory framework on flour enrichment in Kazakhstan. n.p. 2011. 6. The President of the Republic of Kazakhstan. On quality and safety of food products. No. 543-II ZRK. Astana, Kazakhstan: Author. 2004.<br>7. Union of grain processors of bread bakers of Kazakhstan. Mixture for preliminary flour fortification. ST TOO - 2007. Astana, Kazakhstan: Author. 2006.<br>8. Minister of National Economy. The rules for the enrichment (fortification) of food products subject to sanitary and epidemiological surveillance. n.p. 2015.<br>9. Ministry of Industry and Trade of the Republic of Kazakhstan. Technical Regulation and Metrology Committee. Wheat. Technical conditions (official ed.). State standard of the Republic of Kazakhstan. ST RK 1046. Astana city, Kazakhstan: Author. 2008.<br>10. Ministry of Industry and Trade of the Republic of Kazakhstan. Committee on Technical Regulating and Metrology. Wheat bread flour. Fortified. General technical specifications (official ed.). The GOST of the Republic of Kazakhstan. ST RK 1741. Astana, Kazakhstan: Author. 2008.<br>11. The President of the Republic of Kazakhstan. On public health and health care system. No. 193-IV. n.p. 2009.<br>12. The President of the Republic of Kazakhstan. Administrative offences code. No. 155. n.p. 2001.

Mandatorily Fortified Grain**	Country	Region	Documents Reviewed
	Kenya	Africa	1. East African Community (EAC). Fortified wheat flour — specification. Final Draft East African Standard. FDEAS 767:2012. Arusha, Tanzania: EAC. 2012. 2. Minister for Public Health and Sanitation. The food, drugs and chemical substances act. Kenya: Kenya Gazette Supplement No.62. Legislative Supplement No.19. Legal Notice No.62. 2012.<br>3. Ministry of Public Health & Sanitation. Division of Nutrition. Guideline for internal monitoring of fortified maize and wheat Flour. Kenya: Author. n.d.
	Kosovo	Europe	1. President of the Republic of Kosovo. Law No.04/L-114 on flour fortification. Pristina, Kosovo: Official Gazette of the Republic of Kosovo. No. 26. 2012.<br>2. Ministry of Agriculture, Forestry, and Rural Development. Administrative instruction MAFRD No. 07/2013. For flour enrichment standard, flour enrichment control and quality control. Pristina, Kosovo: Author. 2013.
	Kuwait	Middle East	1. Standardization Organization For G.C.C (GSO). Wheat flour. UAE.S GSO 194 :2006. United Arab Emirates: Emirates Authority for Standardization (EMSA). 2006.
	Kyrgyzstan	Europe	1. President of the Kyrgyz Republic. Law of the Kyrgyz Republic on the enriching of baking flour. Kyrgyzstan: Author. n.d.<br>2. President of the Kyrgyz Republic. Law of the Kyrgyz Republic on fortification of baking flour. Kyrgyzstan: Author. 2009.
	Liberia	Africa	1. Food fortification (wheat flour). Required content of vitamins and minerals in fortified wheat flour. n.p. n.d.<br>2. The National Fortification Alliance of Liberia (NFA). Guidelines of the National Fortification Alliance of Liberia. Monrovia, Liberia: NFA. n.d.

Mandatorily Fortified Grain**	Country	Region	Documents Reviewed
	Mexico	Americas	1. Secretary of Health. Official Mexican Standard NOM-247-SSA1-2008, products and services. Cereals and their products. cereal flour, meal or semolina. Food based on: cereals, edible seeds, flour, semoles or semolines or their mixtures. Bakery products. Sanitary and nutrimental provisions and specifications. Test methods. Mexico. n.d.
	Morocco	Africa	1. Food fortified with vitamins and minerals in Morocco. n.p. n.d.<br>2. The Minister of Agriculture and Agricultural Development, & the Minister of Health. Decree No. 2-04-52 of 29 chaoual 1426 on the enrichment of flour. (Box No. 5384 of 05 January 2006, page 10). Rabat, Morocco: Author. 2006.<br>3. Training Institute of the Milling Industry (IFIM). Practical guide to the fortification of flour. rue El Brihmi El Idrissi: National Federation of Milling. 2004.
	Nicaragua	Americas	1. Ministry of Economy, National Council of Science and Technology, Ministry of Development, Industry and Commerce, Secretariat for Industry and Commerce, & Ministry of Economy Industry and Commerce, eds. Flours. Fortified wheat flour. Specifications. Central American technical regulation. RTCA 67.01.15:07. n.p. n.d.<br>2. Ministry of Economy, Salvadoran Agency for Technical Regulation, Ministry of Development, Industry and Commerce, Secretary of Industry and Commerce, & Ministry of Economy, Industry and Commerce, eds. Nutrition labeling of prepackaged food products for human consumption for the population from 3 years of age. Central American technical regulation. RTCA 67.01.60:10. n.p. n.d.

Mandatorily Fortified Grain**	Country	Region	Documents Reviewed
	Nigeria	Africa	1. National Agency for Food and Drug Administration and Control (NAFDAC). National monitoring & evaluation framework for food fortification programmes in Nigeria. Abuja, Nigeria: NAFDAC. 2014.<br>2. Nigerian Industrial Standard. Standard for fortificants premix. NIS 475: 2015. Abuja, Nigeria: Standards Organisation of Nigeria. 2015.<br>3. Nigerian Industrial Standard. Standard for wheat flour. NIS 121: 2015. Abuja, Nigeria: Standards Organisation of Nigeria. 2015.<br>4. Nigerian Industrial Standard. Standard for composite flour. NIS 294: 2015. Abuja, Nigeria: Standards Organisation of Nigeria. 2015.<br>5. Nigerian Industrial Standard. Standard for wheat semolina. NIS 396: 2015. Abuja, Nigeria: Standards Organisation of Nigeria. 2015.
	Palestine Occupied Territory	Middle East	1. Ministry of Health. Nutrition Department. Palestinian flour fortification formula. Ramallah, Palestine Territory: Author. 2010.
	Panama	Americas	1. The President of the Republic. Ministry of Health. Fortification of wheat flour. Executive decree No. 80. Panama: Official Gazette (No. 24,772). 2003.<br>2. Institute of Nutrition of Central America and Panama, Pan American Health Organization, & Ministry of Health Panama. Manual for the monitoring of fortified wheat flour. Republic of Panama: Author. 2011.<br>3. Panamanian Food Safety Authority (AUPSA). Procedural Manual. Bulk Food Sampling (Version 13.05.2009). MPDNAC-004-09. (n.p.): AUPSA. n.d.

Mandatorily Fortified Grain**	Country	Region	Documents Reviewed
	Paraguay	Americas	1. Ministry of Public Health and Social Welfare. General Secretary. Resolution S. G. No. 27. By which the technical regulation of the enrichment of the wheat flour is approved, and the resolution S.G. No. 272, dated June 8, 2001. (n.p.): Author. 2002.<br>2. Presidency of the Republic. Ministry of Public Health and Social welfare. By which it is declared mandatory the enrichment of the wheat flour, with iron and vitamins. Decree No. 20830. (n.p.): Author. 1998.<br>3. National government, National Institute of Food and Nutrition (INAN), & Ministry of Public Health and Social welfare. Guide for the assurance and quality control of the process of enrichment of wheat flour in processing establishments. Internal monitoring. (n.p.): Author. 2014.
	Peru	Americas	1. The President of the Republic. Regulation of law No.28314, which provided fortification of wheat flour with micronutrients. Supreme decret. No. 012-2006-SA. Laws. Health. Peru: El Peruano. 2006.<br>2. Ministry of Health, National Institute of Health, National Food and Nutrition Center, & Executive Director of Science and Food Technology. Situation of fortification of wheat flour in Peru during the years 2009 and 2010. Lima, Peru: Author. 2013.<br>3. Institute of industrial technological research and technical standards (ITINTEC). Wheat flour for domestic consumption and industrial use. Peru National Technical Standard. ITINTEC 205.027. Lima, Peru: ITINTEC. 1986.
	Philippines	Asia	1. Department of Health. Office of the Secretary. The implementing rules and regulations of republic act no. 8976. Entitled: "An Act Establishing the Philippine Food Fortification Program and for Other Purposes". Manila, Philippines: Department of Health. n.d.

Mandatorily Fortified Grain**	Country	Region	Documents Reviewed
	Republic of Moldova	Europe	1. Decree on the approval of measures to reduce by 2017 disorders determined by iron and folic acid deficiency. No. 171. Chisinau, Republic of Moldova: n.p. 2012.<br>2. Modifications to the classified list of goods of the Republic of Moldova. Annex No. 3 to government decree no.171 of 19 March 2012. n.p. n.d.<br>3. Republic of Moldova. On the approval of the technical regulations "Flour semolina and bran cereals". Government decree no. 68 of 29.01.2009. Republic of Moldova: Official Gazette Nr. 23-26. Article no.: 107. 2009.<br>4. Republic of Moldova. On establishing common principles and requirements of legislation on food safety. Parliament law no. 113 of 18.05.2012. Republic of Moldova: Official Gazette Nr. 143-148. Article no.: 467. 2012.<br>5. Republic of Moldova. On state supervision of public health. Parliament law no. 10 of 02/03/2009. Republic of Moldova: Official Gazette No. 67. Article no.: 183. 2009.<br>6. Republic of Moldova. On food products. Parliament law no. 78 of 18.03.2004. Republic of Moldova: Official Gazette Nr. 83-87. Article no.: 107. 2004.
	Senegal	Africa	1. Soft wheat flour fortified with iron and vitamin B9. Specifications (Rev 3). Senegal standard. NS03-052. n.p. 2013.<br>2. Ministry of Commerce, Industry and Informal Sector, ITA, The Senegalese Committee for the Fortification of Food in Micronutrients (COSFAM), & Micronutrient Initiative (MI). Manual of Procedures. Quality control of fortified oils and flours. (n.p.): Author. 2012.<br>3. Ministry of Commerce. Decree rendering mandatory the application of regulations for refined edible oils enriched with vitamin A and wheat bread flour enriched with iron and folic acid. No. 2009-872. Dakar, Senegal: Author. 2009.
	Sierra Leone	Africa	1. Cereals and pulses - Specification for fortified soft wheat flour. Sierra Leone Standard. SLS 39: 2010. n.p. n.d.
	Solomon Islands	Pacific	1. Pacific standard on wheat flour. n.p. n.d.<br>2. The Pure Food Act 1996 (No. 4 of 1996). (n.p.): Pacific Islands Legal Information Institute. 1996. Available at: http://www.paclii.org/sb/legis/num_act/pfa1996113/<br>3. Pure food (food control) regulations 2010. Legal Notice No.154. Solomon Islands: Supplement to the Solomon Islands Gazette. S.I. No.70. 2010.

Mandatorily Fortified Grain**	Country	Region	Documents Reviewed
	South Africa	Africa	1. Department of Health. No. R 1206. Foodstuffs, cosmetics, and disinfectants act, 1972 (Act No. 54 of 1972). Amendment of regulations relating to the fortification of certain foodstuffs. (n.p.): Government Gazette No. 31584. 2008.<br>2. Department of Health. No. R 7634. Foodstuffs, cosmetics, and disinfectants Act, 1972 (Act No. 54 of 1972). Regulations relating to the fortification of certain foodstuffs. (n.p.): Government notice. 2003.<br>3. Foodstuffs, cosmetics, and disinfectants Act 54 of 1972. n.p. n.d.
	Tanzania, United Republic of	Africa	1. The United Republic of Tanzania. Minister for Health and Social Welfare. The Tanzania food, drugs and cosmetics act. The Tanzania foods, drugs and cosmetics regulations and order, 2011. Government notice no. 205-209. Dar es Salaam, Tanzania: Author. 2011.<br>2. Tanzania Food and Drugs Authority (TFDA). Guidelines for conducting external monitoring food fortification (1st ed.). Dar es Salaam, Tanzania: TFDA. 2012.<br>3. Tanzania Bureau of Standards (TBS). Wheat flour – Specification (2nd ed.). Tanzania Standard. TZS 439:2010. Dar es Salaam, Tanzania: TBS. 2010.<br>4. Tanzania Food and Drugs Authority (TFDA). Manual for internal monitoring of fortified wheat flour (1st ed.). (n.p.): TFDA. 2013.
	Togo	Africa	1. Ministry of Health, Ministry of Commerce and of Promotion of the Private Sector, & Ministry of Industry and of the Free Zone and Technological Innovations. Decree no. 2012-010 / PR relating to enriching refined oils and flour with micronutrients. Lomé, Togo: Author. 2012.<br>2. Ministry of Health, Ministry of Commerce and of Promotion of the Private Sector, & Ministry of Industry and of the Free Zone and Technological Innovations. Inter-Ministerial Order No. 133/2013/MS/ /MCPSP /MIZFIT defining the conditions for the application of Decree No. 2012-010 / PR on the enrichment of refined oils and wheat flour in micronutrients. Lomé, Togo: Author. 2013.<br>3. Ministry of Health, & Ministry Delegate to the President of the Republic in charge of Trade and Promotion of the Private Sector. Interministerial Order No. 0127 /MS/ MDCPSP on the establishment, organization, attributions and functioning of the National Committee for Food Fortification (CNFA). Lomé, Togo: Author. 2009.

Mandatorily Fortified Grain**	Country	Region	Documents Reviewed
	Turkmenistan	Europe	1. Law of Turkmenistan. On ensuring the safety and quality of food products. Ashgabat, Turkmenistan: n.p. 2014.<br>2. Law of Turkmenistan. On Food Security. XVIII-2-B. Ashgabat, Turkmenistan: Bulletin of the Mejlis of Turkmenistan. No. 2. p. 14. 2000.<br>3. President of Turkmenistan. Decree of the President of Turkmenistan No.7855 on the fortification of wheat flour with iron and folic acid. n.p. 2006.
	Uganda	Africa	1. Ministry of Health, & Uganda National Bureau of Standards. Manual for regulatory monitoring of fortified foods. Kampala, Uganda: n.p. n.d.<br>2. The food and drugs (food fortification) regulations, 2005. Statutory Instruments Supplement No. 2. Entebbe, Uganda: UPPC. 2005.<br>3. East African Community (EAC). Fortified wheat flour — specification. Final Draft East African Standard. FDEAS 767:2012. Arusha, Tanzania: EAC. 2012.<br>4. The food and drugs (food fortification) (amendment) regulations, 2011. Statutory Instruments Supplement No. 30. Entebbe, Uganda: UPPC. 2011.
	United Kingdom	Europe	1. Food Standards Agency. The bread and flour regulations 1998 (as amended). Guidance Notes (Version 1). (n.p.): Foods Standard Agency. 2008.<br>2. McQuillan, M. Bread and flour regulations 1998. London, United Kingdom: Department for Environment, Food and Rural Affairs. 2013.<br>3. Department for Environment, Food and Rural Affairs (DEFRA). Bread and flour regulations 1998. A summary of responses to the consultation and government reply. London, United Kingdom: Crown. 2013.<br>4. Food. The bread and flour regulations 1998. Statutory Instruments. No.141. United Kingdom: n.p. 1998.<br>5. Scientific Advisory Committee on Nutrition (SACN). Nutritional implication of repealing the UK bread and flour regulations. (n.p.): SACN. 2012.

Mandatorily Fortified Grain**	Country	Region	Documents Reviewed
	United States of America	Americas	1. Food and Drug Administration (FDA). § 137.165 Enriched flour. 21 CFR Ch. I (4–1–03 Edition). USA: FDA. n.d.<br>2. U.S. Food and Drug Administration (FDA). Guide to inspections of grain product manufacturers. USA: FDA. 2003.<br>3. U.S. Food and Drug Administration (FDA). 9-1 - Import procedures. USA: FDA. n.d.<br>4. Department of Health and Human Services. Food and Drug Administration. Food standards: Amendment of standards of identity for enriched grain products to require addition of folic acid. (n.p.): Federal Register. Rules and regulations. Vol. 61. No. 44. 1996.<br>5. U.S. Food and Drug Administration (FDA). Center for Food Safety and Applied Nutrition. Guidance for industry: Questions and Answers on FDA's fortification policy. Maryland, USA: FDA. 2015.<br>6. U.S. Food and Drug Administration (FDA). Part 104 Nutritional quality guidelines for foods. Code of federal regulations. Title 21 - Food and Drugs. MD, USA: FDA. n.d.
	Uruguay	Americas	1. Ministry of Public Health, Ministry of Economy and Finance, & Ministry of Industry, Energy and Mining. Seen: studies carried out by the Nutrition Committee of the Uruguayan Society of Pediatrics, in a joint project with UNICEF, of which there are obvious nutritional deficiencies. Montevideo, Uruguay: Author. 2006.<br>2. Ministry of Public Health. Ministry of Foreign Affairs, Ministry of Industry, Mining and Energy, & Ministry of Livestock, Agriculture and Fisheries. Law No.18.071. Prevention of various diseases. For the purpose, the fortification of certain foods. Montevideo, Uruguay: Author. No. 27144. 2006.<br>3. Ministry of Public Health, Ministry of Economy and Finance, & Ministry of Industry, Energy and Mining. Views: Law No. 18,071 of December 11, 2007 and Executive Decree No. 130/006 of May 4, 2006. Case no. 37. Montevideo, Uruguay: Author. 2010.
	Uzbekistan	Europe	1. Prime Minister. Measures for the implementation of the law of the Republic of Uzbekistan "on prevention of micronutrient deficiency among the population". Resolution of the Cabinet of the Ministry of the Republic of Uzbekistan. No. 260. n.p. 2010.<br>2. Ministry of Health of the Republic of Uzbekistan, JSC "Uzdonmahsulot", Global Alliance for improved Nutrition (GAIN), United Nations Children's Fund (UNICEF), & World Bank. Microelements - macro consequences. Tashkent, Uzbekistan: UNICEF. n.d.

Mandatorily Fortified Grain**	Country	Region	Documents Reviewed
	Venezuela	Americas	1. Covenin. Wheat flour (4th Review). Venezuela Standard. 217:2001. Caracas, Venezuela: Fondonorma. n.d.<br>2. Covenin. Sampling procedures for inspection by attributes. Part 1: Sampling Schemes Indexed by Quality of Acceptance (NCA) for lot-by-lot inspection. Venezuela Standard. 3133-1:2001 (2859-1:1999). Caracas, Venezuela: Fondonorma. n.d.<br>3. Covenin. Grain-leguminous-oilseeds and by-products sampling. Venezuela Standard. 612-82. Caracas, Venezuela: Fondonorma. n.d.
**Maize Flour (n=11)**			
	Brazil	Americas	1. National Health Surveillance Agency (ANVISA). RDC Resolution No. 344. Approve the technical regulations for the wheat flour and corn flour fortification with iron and folic acid, found in the annex to this resolution. Brazil: Official Federal Gazette. 2002.<br>2. Decree No. 1711. n.p. 2012.<br>3. Germani, R., Ascheri, J. L. R., Silva, F. T., Torrezan, R., Silva, K. L., Netto, A. G., & Nutti, M. R. Manual for fortification of wheat flour with iron. Documents 46. Rio de Janeiro, Brazil: Embrapa Agroindústria de Alimentos. 2001.<br>4. The President of the Republic. Law No. 6,437. Sets forth the violations to federal sanitary legislation, establishes their respective penalties, and makes other provisions. Brasília, Brazil: n.p. 1977.

Mandatorily Fortified Grain**	Country	Region	Documents Reviewed
	Costa Rica	Americas	1. The President of the Republic, & the Minister of Health. Regulation for the enrichment of maize flour. No. 28086-S. (n.p): Gazette. No. 184. 1999.<br>2. The President of the Republic, & the Minister of Health. Costa Rica: Executive power. Decrees. No. 28086-S. San José, Costa Rica: n.p. 1999.<br>3. Institute of Nutrition of Central America and Panama (INCAP), United Nations Children's Fund (UNICEF) & Food and Nutrition Foundation of Central America and Panama (FANCAP). Manual for the inspection of fortified foods at sales sites. (Commercial monitoring) (2nd ed.). n.p. 2011.<br>4. Ministry of Economy, National Council of Science and Technology, Ministry of Economy, Industry and Commerce, Ministry of Development, Industry and Commerce, Ministry of Industry and Commerce, eds. General labeling of previously packaged foods (pre-packaged). Central American technical regulation. RTCA 67.01.02:10. n.p. n.d.<br>5. Ministry of Economy, Salvadoran Agency for Technical Regulation, Ministry of Development, Industry and Commerce, Ministry of Industry and Commerce, Ministry of Economy, Industry and Commerce, eds. Nutrition labeling of prepackaged food products for human consumption for the population from 3 years of age. Central American technical regulation. RTCA 67.01.60:10. n.p. n.d.<br>6. The President of the Republic, & the Ministers of Foreign Trade and Economy, Industry and Commerce. Executive Decree No. 37280 -COMEX-MEIC. Tegucigalpa, Honduras: n.p. 2012.<br>7. The President of the Republic, & the Minister of Health. Decree 31595-S de 2-12-2003 Gaceta 16 del 23-1-2004. San José, Costa Rica: n.p. 2003.

Mandatorily Fortified Grain**	Country	Region	Documents Reviewed
	El Salvador	Americas	1. National Council of Science and Technology (CONACYT). Flour. Nixtamalized corn flour (1st update). Norma Salvadorena. NSO 67.03.02:08. San Salvador, El Salvador: CONACYT. n.d.<br>2. National Council of Science and Technology (CONACYT). Flour. Nixtamalized corn flour. Norma Salvadorena. NSO 67.03.02:03. San Salvador, El Salvador: CONACYT. n.d.<br>3. Agreement No. 402.<br>The Executive Body in the Economy Branch. Having regard to the request made by Carlos Roberto Ochoa Cordova, Executive Director of the National Council of Science and Technology, CONACYT, on the adoption of the mandatory Salvadorean standard: general rule for the labeling of prepackaged foods NSO 67.10.01:03. San Salvador, El Salvador: Official Journal. Volume no. 359. 2003.<br>4. Ministry of Economy, Salvadoran Agency for Technical Regulation, Ministry of Development, Industry and Commerce, Ministry of Industry and Commerce, & Ministry of Economy, Industry and Commerce, eds. Nutritional labeling of foodstuffs prepared for human consumption for population from 3 years of age. Central American technical regulation. RTCA 67.01.60:10. n.p. n.d.<br>5. Ministry of Health. Occupational Safety and Health Committee. Manual of technical procedures for the monitoring and evaluation of the food fortification program (1st update). San Salvador, El Salvador: Author. 2011.
	Kenya	Africa	1. East African Community (EAC). Fortified milled maize (corn) products — specification. Final Draft East African Standard. FDEAS 768:2012. Arusha, Tanzania: EAC. 2012. 2. Minister for Public Health and Sanitation. The food, drugs and chemical substances act. Kenya: Kenya Gazette Supplement. No.62. Legislative Supplement No.19. Legal Notice No.62. 2012.<br>3. Ministry of Public Health & Sanitation. Division of Nutrition. Guideline for internal monitoring of fortified maize and wheat Flour. Kenya: Author. n.d.
	Mexico	Americas	1. Secretary of Health. Official Mexican Standard NOM-247-SSA1-2008, products and services. Cereals and their products. cereal flour, meal or semolina. Food based on: cereals, edible seeds, flour, semoles or semolines or their mixtures. Bakery products. Sanitary and nutrimental provisions and specifications. Test methods. Mexico: n.p. n.d.

Marks KJ, Luthringer CL, Ruth LJ, et al. Review of grain fortification legislation, standards, and monitoring documents. *Glob Health Sci Pract*. 2018;6(2). https://doi.org/10.9745/GHSP-D-17-00427

Mandatorily Fortified Grain**	Country	Region	Documents Reviewed
	Nigeria	Africa	1. National Agency for Food and Drug Administration and Control (NAFDAC). National monitoring & evaluation framework for food fortification programmes in Nigeria. Abuja, Nigeria: NAFDAC. 2014.<br>2. Nigerian Industrial Standard. Standard for fortificants premix. NIS 475: 2015. Abuja, Nigeria: Standards Organisation of Nigeria. 2015.<br>3. Nigerian Industrial Standard. Standard for whole maize meal. NIS 822: 2015. Abuja, Nigeria: Standards Organisation of Nigeria. 2015.<br>4. Nigerian Industrial Standard. Standard for composite flour. NIS 294: 2015. Abuja, Nigeria: Standards Organisation of Nigeria. 2015.<br>5. Nigerian Industrial Standard. Standard for maize grit. NIS 718: 2010. Abuja, Nigeria: Standards Organisation of Nigeria. 2010.<br>6. Nigerian Industrial Standard. Standard for maize flour. NIS 723: 2010. Abuja, Nigeria: Standards Organisation of Nigeria. 2010.
	South Africa	Africa	1. Department of Health. No. R 1206. Foodstuffs, cosmetics, and disinfectants act, 1972 (Act No. 54 of 1972). Amendment of regulations relating to the fortification of certain foodstuffs. (n.p.): Government Gazette. No. 31584. 2008.<br>2. Department of Health. No. R 7634. Foodstuffs, cosmetics, and disinfectants act, 1972 (Act No. 54 of 1972). Regulations relating to the fortification of certain foodstuffs. (n.p.): Government notice. 2003.<br>3. Foodstuffs, cosmetics, and disinfectants act. No. 54 of 1972. n.p. n.d.<br>4. Minister of Agriculture, Forestry and Fisheries. Proclamation No. 3 of 2016. Agricultural products standards act, 1990 (Act No. 119 of 1990). Regulations relating to the grading, packing, and marking of maize products intended for sale in the Republic of South Africa. (n.p): Government Gazette. No. 39613. 2016.

Mandatorily Fortified Grain**	Country	Region	Documents Reviewed
	Tanzania, United Republic of	Africa	1. The United Republic of Tanzania. Minister for Health and Social Welfare. The Tanzania food, drugs and cosmetics act. The Tanzania foods, drugs and cosmetics regulations and order, 2011. Dar es Salaam, Tanzania: Author. Government notice no. 205-209. 2011.<br>2. Tanzania Food and Drugs Authority (TFDA). Guidelines for conducting external monitoring food fortification (1st ed.). Dar es Salaam, Tanzania: TFDA. 2012.<br>3. Tanzania Bureau of Standards (TBS). Maize flour – specification (2nd ed.). Tanzania Standard. TZS 328:2010. Dar es Salaam, Tanzania: TBS. 2010.<br>4. Tanzania Food and Drugs Authority (TFDA). Manual for internal monitoring of fortified maize flour. Dar es Salaam, Tanzania: TFDA. 2010.
	Uganda	Africa	1. Ministry of Health, & Uganda National Bureau of Standards. Manual for regulatory monitoring of fortified foods. Kampala, Uganda: Author. n.d.<br>2. Minister of Health. The food and drugs (food fortification) regulations, 2005. Entebbe, Uganda: UPPC. Statutory Instruments Supplement No. 2. 2005.<br>3. Uganda National Bureau of Standards (UNBS). Fortified milled maize products – specification (2nd ed.). Uganda Standard. US 509. Kampala, Uganda: UNBS. 2006.<br>4. Minister of Health. The food and drugs (food fortification) (amendment) regulations, 2011. Entebbe, Uganda: UPPC. Statutory Instruments Supplement No. 30. 2011.<br>5. East African Community (EAC). Fortified milled maize (corn) products — specification. Final Draft East African Standard. FDEAS 768:2012. Arusha, Tanzania: EAC. 2012.

Mandatorily Fortified Grain**	Country	Region	Documents Reviewed
	United States of America	Americas	1. U.S. Food and Drug Administration (FDA). Sec. 137.260 Enriched corn meals. Part 137 Cereal flours and related products. Title 21 - Food and Drugs. Maryland, USA: FDA. Code of federal regulations. Title 21. Volume 2. 2012.<br>2. U.S. Food and Drug Administration (FDA). Center for Food Safety and Applied Nutrition. Guidance for industry: questions and answers on FDA's fortification policy. Maryland, USA: FDA. 2015.<br>3. U.S. Food and Drug Administration (FDA). Part 104 Nutritional quality guidelines for foods. Title 21 - Food and Drugs. Maryland, USA: FDA. Code of federal regulations. Title 21. Volume 2. 2015.
	Venezuela	Americas	1. Covenin. Precooked cornmeal (3rd review). Venezuela Standard. 2135:1996. Caracas, Venezuela: Fondonorma. n.d.
**Rice (n=6)**			
	Costa Rica	Americas	1. The President The Republic, & Ministry of Health. Regulation for rice enrichment. No. 30031. (n.p.): Gazette. No. 1. 2002.<br>2. The President of the Republic, & The Minister of Health. Executive power. Decrees. No. 33121-S. San José, Costa Rica: La Gaceta. No. 106. 2006.<br>3. Ministry of Economy, Salvadoran Agency for Technical Regulation, Ministry of Development, Industry and Commerce, Ministry of Industry and Commerce, Ministry of Economy, Industry and Commerce, eds. Nutrition labeling of prepackaged food products for human consumption for the population from 3 years of age. Central American technical regulation. RTCA 67.01.60:10. n.p. n.d.

Mandatorily Fortified Grain**	Country	Region	Documents Reviewed
	Nicaragua	Americas	1. Nicaraguan mandatory technical standard. Rice fortification. NTON 03 091 - 11. Managua, Nicaragua: La Gaceta. No. 112. 2014.<br><br>2. Ministry of Health. Ministerial Resolution. No. 235. Managua, Nicaragua: Author. 2009.<br><br>3. Ministry of Economy, Salvadoran Agency for Technical Regulation, Ministry of Development, Industry and Commerce, Secretary of Industry and Commerce, & Ministry of Economy, Industry and Commerce, eds. Nutrition labeling of prepackaged food products for human consumption for the population from 3 years of age. Central American technical regulation. RTCA 67.01.60:10. n.p. n.d.
	Panama	Americas	1. Law 33. Creating the rice fortification program. Panama: Offical Digital Gazette. No. 26314. 2009.
	Papua New Guinea	Pacific	1. Food Sanitation Regulation 2007. Statutory instrument. No. 01 of 2007. Independent State of Papua New Guinea: n.p. n.d.
	Philippines	Asia	1. Republic of the Philippines. An act establishing the Philippine food fortification program and for other purposes. Republic act no. 8976. Manila, Philippines: n.p. 2000.<br>2. Republic Act No. 832 - An act to regulate the sale, exchange, or delivery of home pounded, undermilled, milled or polished rice, and providing penalty for violation thereof. n.p. 1952.

Marks KJ, Luthringer CL, Ruth LJ, et al. Review of grain fortification legislation, standards, and monitoring documents. *Glob Health Sci Pract*. 2018;6(2). https://doi.org/10.9745/GHSP-D-17-00427

Mandatorily Fortified Grain**	Country	Region	Documents Reviewed
	United States of America	Americas	1. U.S. Food and Drug Administration (FDA). Sec. 137.350 Enriched rice. Part 137 Cereal flours and related products. Title 21 - Food and Drugs. Maryland, USA: FDA. Code of federal regulations. Title 21. Volume 2. 2015.<br>2. U.S. Food and Drug Administration (FDA). Center for Food Safety and Applied Nutrition. Guidance for industry: questions and answers on FDA's fortification policy. Maryland, USA: FDA. 2015.<br>3. U.S. Food and Drug Administration (FDA). Part 104 Nutritional quality guidelines for foods. Title 21 - Food and Drugs. Maryland, USA: FDA. Code of federal regulations. Title 21. Volume 2. 2015.

Abbreviations: n.p. no information of publisher/place of publication found; n.d. no date of publication found.
*Country-grain combination refers to the unit of analysis; countries that mandate the fortification of multiple cereal grains will contribute more than one country-grain combination (e.g., Philippines-wheat and Philippines-rice).
**Mandatory fortification as of 31 January 2015.
^Caribbean Community and Common Market (CARICOM) standard (Caribbean Community Secretariat, 1995).

Marks KJ, Luthringer CL, Ruth LJ, et al. Review of grain fortification legislation, standards, and monitoring documents. *Glob Health Sci Pract*. 2018;6(2). https://doi.org/10.9745/GHSP-D-17-00427
