# Supplementary material for: Review of Grain Fortification Legislation, Standards, and Monitoring Documents
Source: Glob Health Sci Pract. 2018 Jun 27;6(2):356–71. doi: 10.9745/GHSP-D-17-00427 (PMC6024620; doi:10.9745/GHSP-D-17-00427)
Supplement: 17-00427-Marks-SupplementTable2.pdf [file 17-00427-Marks-SupplementTable2.pdf]

Marks KJ, Luthringer CL, Ruth LJ, et al. Review of grain fortification legislation, standards, and monitoring documents. *Glob Health Sci Pract*. 2018;6(2). https://doi.org/10.9745/GHSP-D-17-00427

SUPPLEMENT TABLE 2. Sensitivity analysis comparing percentage of country-grain combinations* with documented items of fortification legislation, standards, and monitoring documents by verification status (N=72)

Item	Verified Reviews** (n=23)	Unverified Reviews** (n=49)	p-value^
General			
Food vehicle stated in legislation	91%	100%	0.04
Public health objective/purpose	74%	67%	0.58
Accepted international norms	57%	53%	0.79
Definitions specific to fortification	78%	76%	0.80
Repeals of prior documentation	61%	57%	0.77
Effective date/grace period	74%	71%	0.83
Micronutrients/premix			
Nutrients required	100%	100%	1.00
Fortificants (chemical compounds)	91%	86%	0.51
Fortification levels	43%	41%	0.83
Bioavailability of fortificants	22%	35%	0.27
Nutrient stability	48%	55%	0.57
Costing			
Cost sharing of fortification	26%	16%	0.34
Financial responsibility of monitoring and enforcement	43%	31%	0.29
Labeling			
Labeling required	83%	76%	0.51
Guidance on health claims	48%	51%	0.80
Internal monitoring (conducted by industry during production)			
Sampling process outlined	48%	22%	0.03
Industry QA/QC justified/required	70%	61%	0.50
Applicability of qualitative tests	48%	20%	0.02
External monitoring (conducted by government at production sites)			
External monitoring justified	78%	57%	0.08
Protocols and systems described	39%	31%	0.48
Roles and responsibilities clarified	52%	27%	0.03
Timeline for inspections outlined	35%	22%	0.27
Sampling process outlined	52%	37%	0.22
Applicability of qualitative tests	30%	14%	0.11
Registration requirements	43%	35%	0.48
Commercial monitoring (conducted by government at market or distribution sites)			
Commercial monitoring justified	48%	47%	0.94
Protocols and systems described	30%	14%	0.11
Roles and responsibilities clarified	35%	24%	0.37

Item	Verified Reviews** (n=23)	Unverified Reviews** (n=49)	p-value^
Timeline for inspections outlined	4%	10%	0.41
Sampling process outlined	30%	27%	0.73
Import monitoring (conducted by government at ports/points of entry)			
Import monitoring justified	70%	61%	0.50
Protocols and systems described	35%	35%	0.99
Roles and responsibilities clarified	30%	37%	0.61
Sampling process outlined	35%	20%	0.19
Enforcement/penalties			
Enforcement roles and responsibilities clarified	83%	63%	0.10
Incentives to start fortification	22%	10%	0.19
Incentives to continue fortification	9%	10%	0.84
Penalties to compel compliance	70%	67%	0.85
Penalties objectively defined	17%	22%	0.63
Enforcement includes feedback	39%	8%	0.001
Laboratory			
Analytical methods identified	65%	57%	0.52
Recognition of laboratory variation	13%	10%	0.73
Quantitative analysis of "marker" micronutrients such as iron	52%	29%	0.05
Reporting			
Dissemination of monitoring results described	35%	29%	0.60

*Country-grain combination refers to the unit of analysis; countries that mandate the fortification of multiple cereal grains will contribute more than one country-grain combination (e.g. Philippines-wheat and Philippines-rice).

**Verified reviews=country-grain combinations where country representatives confirmed that all appropriate documentation was included in review.

^Compared using chi-square tests.
