# Supplementary material for: Review of Grain Fortification Legislation, Standards, and Monitoring Documents
Source: Glob Health Sci Pract. 2018 Jun 27;6(2):356–71. doi: 10.9745/GHSP-D-17-00427 (PMC6024620; doi:10.9745/GHSP-D-17-00427)
Supplement: 17-00427-Marks-SupplementTable4.pdf [file 17-00427-Marks-SupplementTable4.pdf]

Marks KJ, Luthringer CL, Ruth LJ, et al. Review of grain fortification legislation, standards, and monitoring documents. *Glob Health Sci Pract*. 2018;6(2). https://doi.org/10.9745/GHSP-D-17-00427

SUPPLEMENT TABLE 4. Original Scoring Sheet for Each Country-Grain Combination Reviewed, Ordered by Grain and Country Name (N=72)

Argentina Wheat

Element	Scoring (use drop-down menus)	Comments (open text)
**GENERAL**		
1. States that legislation applies to at least one food vehicle fit for human consumption (types/grades to be fortified)	(2) States at least one type fit for human consumption	ANEXO 1 "ARTICULO 1º — Las disposiciones de la Ley Nº 25.630 y de este Decreto Reglamentario se aplican a todas las harinas de trigo que se utilizan para el consumo humano en el país, sean éstas de producción nacional o importadas, para su consumo directo o procesadas."
2. States the public health objective; purpose and scope of legislation	(2) States the public health objective or general purpose of legislation	"ARTICULO 1º — La presente ley tiene como objeto la prevención de las anemias y las malformaciones del tubo neural, tales como la anencefalia y la espina bífida."
3. References latest available science or accepted international norms and recommendations, particularly for items that may not be covered in the country's documents	(2) States the documents referenced	These are listed as references, but it is not stated how/when they were used, if at all. "2.0. REFERENCIAS<br>2.1. Food Fortification: Technology and Quality Control. (FAO Food And Nutrition Paper - 60) Report of an FAO. Rome, 1996.<br>2.2. Norma Técnica para la Fortificación de la Harina de Trigo con Vitaminas y Minerales. Ministerio de Salud de Chile, 1999.<br>2.3. Reglamento Técnico de Fortificación de Harina de Trigo. Ministerio de Salud y Previsión Social y Ministerio de Desarrollo Económico de Bolivia, 1997.<br>2.4. WHO-EMRO Flour Fortification homepage."
4. Provides definitions that includes terms that are specific to fortification (e.g. fortified food, premix, fortificant, food vehicle)	(2) States at least one term related to fortification	ANEXO "3.0. DEFINICIONES<br>Para los propósitos de este plan se aplican las siguientes definiciones:<br>3.1. Harina: Producto obtenido de la molienda del endosperma del grano de<br>trigo (Artículo 661 del Código Alimentario Argentino).<br>3.2. Núcleo Vitamínico: mezcla a base de sulfato ferroso, mononitrato de<br>tiamina, riboflavina, nicotinamida, ácido fólico y una matriz adecuada utilizada<br>para enriquecer la harina.<br>3.3. Harina Enriquecida: aquella a la que se han adicionado hierro y vitaminas<br>con el objeto de resolver deficiencias de la alimentación que se traducen en<br>fenómenos de carencia colectiva (Artículo 1369 del Código Alimentario<br>Argentino).<br>3.4. Alimento Farináceo o Alimento Elaborado a Base de Harina: Producto<br>obtenido a partir de harina y agua potable, con o sin el agregado de otros<br>productos alimenticios de uso permitido para esta clase de alimentos (Capítulo<br>IX del Código Alimentario Argentino)."
5. Provides repeals (if there is at least one prior document about fortification)	(N/A) No prior documents about fortification	As best I can tell, ref 1 from 2002 is first document on fortification.
6. Provides effective date or gives grace period for when fortification is to begin (e.g. effective 6 months from signing)	(2) States effective date or grace period for when fortification is to begin (e.g. effective 6 months from signing)	"ARTICULO 10. — El Poder Ejecutivo reglamentará la presente dentro de los sesenta (60) días de su promulgación, introduciendo, en ese mismo plazo, las modificaciones al Código Alimentario Argentino necesarias para el cumplimiento de la ley."
**MICRONUTRIENTS/PREMIX**		
7. States nutrients required	(2) States nutrients	"ARTICULO 3º — La harina de trigo destinada al consumo que se comercializa en el mercado nacional, será adicionada con hierro, ácido fólico, tiamina, riboflavina y niacina en las proporciones que a continuación se indican:"
8. States fortificants (chemical compounds) to be used (including fortificants that are allowable as options)	(2) States fortificants for at least one nutrient	Does so in a table in "ARTICULO 3º".
9. States fortification levels	(1) States one number only	Does so in a table in "ARTICULO 3º".
10. States consideration of bioavailability/biological activity of fortificants	(0) Does not state any consideration	
11. States consideration of nutrient stability	(0) Does not state any consideration	
**COSTING**		
12. States that the cost of fortification is regulated through cost sharing schemes (between government, industry, consumers) or tax measures [to assist industry]	(0) Does not state any consideration	
13. States consideration of the financial responsibility of monitoring and enforcing fortification (schedule of fees, budget) [on the government side]	(2) Shows consideration that monitoring costs money	Ref 2 describes what governmental agencies will be responsible for assuming the different costs of monitoring: "8.0. COSTOS"
**LABELING**		

14. Includes some sort of statement/label/logo that makes it clear that the product is fortified	(2) Includes a statement, label, or logo	ANEXO 1 "ARTICULO 4º — Todos los envases que contengan harina enriquecida deberán ser rotulados de acuerdo a lo prescrito en el artículo 661 del Código Alimentario Argentino a la que se le agregará la leyenda, "harina enriquecida Ley Nº 25.630", además de lo establecido en las exigencias de rotulación vigentes para los alimentos en general y las particulares que correspondan a los alimentos comprendidos en la excepción de lo determinado en la última parte del segundo párrafo del artículo anterior en lo referido a la leyenda que deben contener.<br>A efectos de la correcta identificación de las harinas de exportación y los productos elaborados para tal fin, deberá agregarse en los correspondientes documentos y/o envases la leyenda "producto exclusivo para exportación"."
15. Provides guidance on health claims that can be made for this product (specific to micronutrients added through fortification)	(0) Does not provide	I reviewed the Argentinean Food Code, and the food labeling chapter in particular and found no reference to this for fortified flour.
**INTERNAL MONITORING (conducted by industry)**		
16. If samples are to be taken, describes the sampling process: for example, number of samples, amount, frequency, individual vs. composite, where samples are taken in the process, and percent considered passing	(N/A) Does not describe the sampling process	Ref 2 describes in great detail what the government should do as part of its monitoring; there is no comparable detailed description of what industry should do.
17. States that industry is required to follow quality assurance/quality control in regards to fortification	(2) States requirement of QA/QC for fortification	(Ref 1) ANEXO 1 "ARTICULO 5º — La garantía de calidad y control interno de la harina de trigo enriquecida en los niveles requeridos en el artículo 3º de la Ley Nº 25.630 son de responsabilidad de los industriales, los que deberán designar un Director Técnico responsable, en los términos establecidos en el artículo 1346 del Código Alimentario Argentino.<br>El Director Técnico y la empresa asumirán ante las autoridades sanitarias, la responsabilidad por la calidad del enriquecimiento del producto." (Ref 2) "4.0. INTRODUCCION Los industriales molineros son responsables ante las autoridades de control de los alimentos de liberar al consumo la harina de trigo enriquecida con hierro y vitaminas para satisfacer el aumento previsto de la IDR (Ingesta Diaria Recomendada) para cada uno de estos nutrientes."
18. States applicability of using qualitative testing to determine the presence or absence of a vitamin or mineral (spot tests, iChecks)	(0) Does not state	Does not state specifically for internal monitoring; does so for government monitoring.
**EXTERNAL MONITORING (conducted by government)**		
19.States requirement for external monitoring at the production site to assure compliance with standards and regulations	(2) States requirement for external monitoring or the need for audits/inspections	[illegible] cumplimiento de la Ley Nº 25.630 y de la presente reglamentación estará a cargo del INSTITUTO NACIONAL DE ALIMENTOS (INAL), de las Autoridades Sanitarias jurisdiccionales y del SERVICIO NACIONAL DE SANIDAD Y CALIDAD AGROALIMENTARIA (SENASA), en el ámbito de sus incumbencias, por los medios que consideren pertinentes. A tales fines el INSTITUTO NACIONAL DE ALIMENTOS (INAL) podrá celebrar acuerdos con instituciones provinciales, nacionales o internacionales. El control se efectuará sobre la base del protocolo de inspección, toma de muestras y análisis que el INSTITUTO NACIONAL DE ALIMENTOS (INAL) elaborará a tal efecto." (Ref 2) ANEXO: "4.0. INTRODUCCION Los industriales molineros son responsables ante las autoridades de control de los alimentos de liberar al consumo la harina de trigo enriquecida con hierro y vitaminas para satisfacer el aumento previsto de la IDR (Ingesta Diaria Recomendada) para cada uno de estos nutrientes. Para asegurar ese objetivo los organismos de control de los alimentos supervisarán la documentación, el proceso de enriquecimiento y el contenido de los nutrientes de la harina tanto en el sitio de la producción y de importación como en el de venta."... "Los organismos de control focalizarán su tarea en los siguientes puntos:<br>· Producción de la harina de trigo enriquecida.<br>· Harina de trigo importada enriquecida.<br>· Harina de trigo enriquecida acondicionada para su venta al consumidor.<br>· Productos elaborados a base de harina de trigo enriquecida.

20. Describes protocols and systems for regulatory monitoring	(2) Includes checklists or provides detailed description of regulatory monitoring procedures	"Que por medio de la Disposición ANMAT Nº 1930/95 se estableció el procedimiento uniforme para la realización de inspecciones a efectuarse por parte del mencionado Instituto." "Que es conveniente que las inspecciones destinadas a fiscalizar el enriquecimiento de la harina de trigo con hierro y vitaminas se desarrollen siguiendo criterios uniformes tendientes a verificar que en los establecimientos se hallen controlados los puntos necesarios para una efectiva acción, a cuyos efectos el INAL elaboró un Plan Nacional de Fiscalización de la Ley Nº 25.630 y su Decreto Reglamentario Nº 597/2003 que comprende los procedimientos de inspección y de muestreo, los modelos de tarjetas, actas y protocolos y el instructivo para el llenado de actas." (Ref 2) Details for the inspection are noted in "4.0. DESCRIPCION DE LAS ACTIVIDADES".
21. If there are two or more government agencies involved in external monitoring, clarifies the roles and responsibilities between different government agencies in external monitoring	(2) Clarifies roles and responsibilities for more than one agency	ANEXO 1 "ARTICULO 6º — ... La vigilancia del cumplimiento de la Ley Nº 25.630 y de la presente reglamentación estará a cargo del INSTITUTO NACIONAL DE ALIMENTOS (INAL), de las Autoridades Sanitarias jurisdiccionales y del SERVICIO NACIONAL DE SANIDAD Y CALIDAD AGROALIMENTARIA (SENASA), en el ámbito de sus incumbencias, por los medios que consideren pertinentes. A tales fines el INSTITUTO NACIONAL DE ALIMENTOS (INAL) podrá celebrar acuerdos con instituciones provinciales, nacionales o internacionales. El control se efectuará sobre la base del protocolo de inspección, toma de muestras y análisis que el INSTITUTO NACIONAL DE ALIMENTOS (INAL) elaborará a tal efecto."
22. Allows for monitoring to be conducted often enough that problems can be identified and addressed on a timely basis; specifies a timeline for inspections (i.e. once every 6 months, increasing to once every 2 months if a discrepancy is found)	(2) Describes frequency and how it is responsive to the needs of industry or the stage of fortification implementation	"4.2.1.1. En el Molino ...<br>Los inspectores inspeccionarán los molinos regularmente. Las inspecciones deberán ser suficientemente extensas como para permitir una observación detallada de todas las actividades de la producción, en caso de no cumplirse los objetivos en el primer día de la inspección deberá continuar al día siguiente. Estas inspecciones se llevarán a cabo (con una frecuencia mínima semestral) según el Cronograma de Inspecciones que se establezca oportunamente, el cual podrá ser modificado cuando se juzgue necesario. Las muestras de harina que los inspectores tomen en estas inspecciones serán enviadas al laboratorio para su control y evaluar cualquier obstáculo o dificultad en el proceso de producción, indicando medidas correctivas.<br>El propósito del control de calidad es facilitar la acción correctiva inmediata durante el proceso de fabricación."
23. If samples are to be taken, describes the sampling process: for example, number of samples, amount, frequency, individual vs. composite, where samples are taken in the process, and percent considered passing	(2) States that samples should be taken as part of external monitoring	"4.3. METODOLOGIA PARA LA TOMA DE MUESTRA PRIMARIA." … "4.3.3.2.a. En el Molino Se tomarán, al azar, 5 (cinco) muestras de harina de trigo que constituirán la muestra ORIGINAL, 5 (cinco) muestras que constituirán la muestra DUPLICADO y 5 (cinco) muestras que constituirán la muestra TRIPLICADO, en caso de corresponder 5 (cinco) muestras que constituirán la muestra CONTROL, del lote que se está elaborando en el momento de la inspección. La cantidad de cada una de las muestras estará comprendida entre 250 y 1000 gramos para el producto a granel y entre 500 y 1000 gramos para la harina acondicionada para la venta al por menor.<br>Queda a criterio del inspector actuante tomar muestras de lotes de días anteriores o existentes en depósito."
24. States applicability of using qualitative testing to determine the presence or absence of a vitamin or mineral (spot tests, iChecks)	(2) States applicability of spot test to determine presence/absence of vitamin or mineral specific to external monitoring	(Ref 2) The iron spot test is used in a semi-quantitative manner. "4.3.4. Método Analítico a Utilizar para el Testeo de las Muestras en los Sitios de Inspección<br>4.3.4.1. Determinación semicuantitativa de hierro.<br>Se llevará a cabo mediante la técnica de Colorimetría visual." ... "APENDICE I-V<br>PROCEDIMIENTO DE ANALISIS"

25. States registration is required in order to use a logo/be licensed to produce fortified foods	(0) Does not state that registration or licensing is required	Ref 1 states that ANMAT/INAL will maintain a databaes of flour producers in the country, including those that are exempt from the fortification law. ANEXO 1 "ARTICULO 6º — La ADMINISTRACION NACIONAL DE MEDICAMENTOS, ALIMENTOS Y TECNOLOGIA MEDICA (A.N.M.A.T.), a través del INSTITUTO NACIONAL DE ALIMENTOS (INAL) llevará una base de datos, la cual se cargará con aquellos que remitan las jurisdicciones sanitarias provinciales y el GOBIERNO de la CIUDAD AUTONOMA DE BUENOS AIRES, a los fines de registrar todos los establecimientos elaboradores de harina del país, la que contendrá como mínimo UN (1) subregistro de los establecimientos exceptuados en la última parte del párrafo 1º del artículo 3º de la presente reglamentación." (Ref 2) ANEXO 4.0 INTRODUCCION "Todos los establecimientos elaboradores de harina autorizados del país deben estar registrados."
**COMMERCIAL MONITORING (conducted by government)**		
26. Provides justification for commercial monitoring at retail stores	(2) Provides justification for commercial monitoring	Los industriales molineros son responsables ante las autoridades de control de los alimentos de liberar al consumo la harina de trigo enriquecida con hierro y vitaminas para satisfacer el aumento previsto de la IDR (Ingesta Diaria Recomendada) para cada uno de estos nutrientes. Para asegurar ese objetivo los organismos de control de los alimentos supervisarán la documentación, el proceso de enriquecimiento y el contenido de los nutrientes de la harina tanto en el sitio de la producción y de importación como en el de venta." ... "Los organismos de control focalizarán su tarea en los siguientes puntos:<br>· Producción de la harina de trigo enriquecida.<br>· Harina de trigo importada enriquecida.<br>· Harina de trigo enriquecida acondicionada para su venta al consumidor.<br>· Productos elaborados a base de harina de trigo enriquecida.<br>· Productos importados elaborados a base de harina de trigo enriquecida<br>Las autoridades de control fiscalizarán los procesos de producción y los niveles de nutrientes que necesariamente deben contener las harinas enriquecidas y los productos con ellas elaborados.<br>Están exceptuados los productos para regímenes especiales que requieran una proporción mayor o menor de los nutrientes que deben adicionarse por ley, las harinas destinadas a exportación y las harinas destinadas a elaborar productos para exportación y las contempladas en la Ley 25.127 de Producción Ecológica, Biológica u Orgánica."
27. Describes protocols and systems for commercial monitoring	(2) Includes checklists or provides detailed description of regulatory monitoring procedures	la inspección de los sitios de producción, importación, venta y de los establecimientos productores y fraccionadores de alimentos farináceos." "4.2.3.2.c. En los establecimientos fraccionadores/elaboradores de productos<br>farináceos<br>El inspector deberá corroborar la existencia de harina enriquecida la que deberá estar debidamente rotulada de acuerdo con la legislación vigente. El inspector deberá controlar los protocolos del/los proveedores de harina enriquecida que se está utilizando en el momento de la inspección y de la que está almacenada y podrá determinar por análisis químico semicuantitativo la presencia y el nivel aproximado de hierro en la harina de trigo empleada como materia prima para la elaboración de los productos, para ello tomará 5 (cinco) muestras al azar y dejará constancia de los resultados obtenidos en el Acta de Inspección (Apéndice I-IC (para los Establecimientos Elaboradores) del presente procedimiento)<br>En caso de duda tomará muestras según lo establecido en el punto 4.3. del presente documento y las enviará inmediatamente, junto con el Acta de inspección, al laboratorio de referencia o a los autorizados por éste, para su análisis.<br>Quedan exceptuados de este control los productos para regímenes especiales que requieran una proporción mayor o menor de los nutrientes que deben adicionarse por ley y los productos farináceos destinados a exportación.<br>El inspector deberá seguir la guía para el "control del

28. If there are two or more government agencies involved in commercial monitoring, clarifies the roles and responsibilities between different government agencies in commercial monitoring	(2) Clarifies roles and responsibilities for more than one agency	asegurar ese objetivo<br>los organismos de control de los alimentos supervisarán la documentación, el<br>proceso de enriquecimiento y el contenido de los nutrientes de la harina tanto<br>en el sitio de la producción y de importación como en el de venta.<br>El MINISTERIO de SALUD a través de la ADMINISTRACION NACIONAL DE<br>MEDICAMENTOS, ALIMENTOS Y TECNOLOGIA MEDICA y del INSTITUTO<br>NACIONAL de ALIMENTOS, en coordinación con las Autoridades Sanitarias<br>Jurisdiccionales y el SERVICIO NACIONAL de CALIDAD Y SANIDAD<br>AGROALIMENTARIA en el ámbito de sus incumbencias, tendrá a su cargo la<br>vigilancia del cumplimiento de la Ley Nº 25.630 de prevención de las anemias y<br>malformaciones del tubo neural en los términos del Decreto Reglamentario<br>597/2003, por los medios que considere pertinentes.<br>También coordinará las acciones con otros organismos oficiales (nacionales,<br>provinciales y municipales) tendientes a fiscalizar el cumplimiento de la citada<br>ley.<br>De acuerdo con la citada ley la harina de trigo destinada al
29. Allows for monitoring to be conducted often enough that problems at the production site or import companies can be identified and addressed on a timely basis; specifies a timeline for inspections (i.e. once every 6 months) or works with production companies to correct noncompliance	(0) Does not state	
30. If samples are to be taken, describes the sampling process: for example, number of samples, amount, frequency, individual vs. composite, where samples are taken in the process, and percent considered passing	(2) States that samples should be taken as part of commercial monitoring	"4.3. METODOLOGIA PARA LA TOMA DE MUESTRA PRIMARIA." ... "4.3.3.2.c. En las bocas de expendio<br>Se tomarán, al azar, 5 (cinco) muestras que constituirán la muestra ORIGINAL, 5 (cinco) muestras que constituirán la muestra DUPLICADO y 5 (cinco) muestras que constituirán la muestra TRIPLICADO, en caso de corresponder 5 (cinco) muestras que constituirán la muestra CONTROL, de un mismo lote.<br>Las muestras serán remitidas al laboratorio en su envase original, pudiendo ser de 500 ó 1000 gramos en caso de harina de trigo o de alimentos elaborados a base de harina de trigo acondicionados para la venta al por menor o de 250 a 1000 gramos para harina de trigo de uso industrial.<br>4.3.3.2.d. En los establecimientos fraccionadores/elaboradores de alimentos farináceos<br>Se tomarán, al azar, 5 (cinco) muestras que constituirán la muestra ORIGINAL, 5 (cinco) muestras que constituirán la muestra DUPLICADO y 5 (cinco) muestras que constituirán la muestra TRIPLICADO, en caso de corresponder 5 (cinco) muestras que constituirán la muestra CONTROL, del lote de la harina que se está fraccionando o del producto que se está elaborando en el momento de la inspección."
**IMPORT MONITORING (conducted by government)**		

31. Provides justification for import monitoring at points of entry	(2) Provides justification for import monitoring	Los industriales molineros son responsables ante las autoridades de control de los alimentos de liberar al consumo la harina de trigo enriquecida con hierro y vitaminas para satisfacer el aumento previsto de la IDR (Ingesta Diaria Recomendada) para cada uno de estos nutrientes. Para asegurar ese objetivo los organismos de control de los alimentos supervisarán la documentación, el proceso de enriquecimiento y el contenido de los nutrientes de la harina tanto en el sitio de la producción y de importación como en el de venta." ... "Los organismos de control focalizarán su tarea en los siguientes puntos:<br>· Producción de la harina de trigo enriquecida.<br>· Harina de trigo importada enriquecida.<br>· Harina de trigo enriquecida acondicionada para su venta al consumidor.<br>· Productos elaborados a base de harina de trigo enriquecida.<br>· Productos importados elaborados a base de harina de trigo enriquecida<br>Las autoridades de control fiscalizarán los procesos de producción y los niveles de nutrientes que necesariamente deben contener las harinas enriquecidas y los productos con ellas elaborados.<br>Están exceptuados los productos para regímenes especiales que requieran una proporción mayor o menor de los nutrientes que deben adicionarse por ley, las harinas destinadas a exportación y las harinas destinadas a elaborar productos para exportación y las contempladas en la Ley 25.127 de Producción Ecológica, Biológica u Orgánica."
32. Describes protocols and systems for import monitoring	(2) Includes checklists or detailed description of import monitoring procedures	(Ref 2) Details for the inspection are noted in "4.0. DESCRIPCION DE LAS ACTIVIDADES".
33. If there are two or more government agencies involved in import monitoring, clarifies the roles and responsibilities between different government agencies in import monitoring	(2) Clarifies roles and responsibilities for more than one agency	"4.2.3.2.b. En las aduanas y depósitos Atento a la vigencia de la ley 25.630 y su Decreto Reglamentario 597/03 las autoridades aduaneras no podrán liberar los productos importados que incluye la ley sin la intervención de la autoridad sanitaria competente (Res. ANA Nº 445/96), a los efectos de prevenir que cualquiera de estos productos ingrese sin que se verifique su conformidad con las normas vigentes ya que el contenido de nutrientes debe cumplir los mismos requisitos que los productos localmente producidos. Las autoridades competentes deberán verificar, junto con la documentación pertinente, la composición cualitativa y cuantitativa de los nutrientes. Esta deberá estar de acuerdo con los niveles de hierro y vitaminas establecidos en la ley Nº 25.630 y su Decreto Reglamentario 597/2003."
34. If samples are to be taken, describes the sampling process: for example, number of samples, amount, frequency, individual vs. composite, where samples are taken in the process, and percent considered passing	(2) States that samples should be taken as part of import monitoring	"4.2.3.2.b. El inspector tomará muestras de harina al azar, de cada uno de los lotes que componen la importación y procederá a testearlas siguiendo el procedimiento que consta en Apéndice I-V del presente documento y deberá dejar constancia de los resultados obtenidos en el Acta de Inspección correspondiente (Apéndice I-IB (para las Aduanas y Depósitos) del presente procedimiento) En caso de encontrar anormalidades o resultados dudosos deberá proceder a tomar muestras según lo establecido en el punto 4.3. del presente documento.<br>Las remitirá inmediatamente al laboratorio para su control, junto con el Acta de la inspección." ...<br>"4.3. METODOLOGIA PARA LA TOMA DE MUESTRA PRIMARIA." ... "4.3.3.2.b. En las aduanas y depósitos<br>Se tomarán, al azar, 5 (cinco) muestras de harina de trigo o de alimentos elaborados a base de harina de trigo que constituirán la muestra ORIGINAL, 5 (cinco) muestras que constituirán la muestra DUPLICADO y 5 (cinco) muestras que constituirán la muestra TRIPLICADO, de un mismo lote, de 250 ó 1000 gramos."
**ENFORCEMENT/PENALTIES**		

35. Indicates roles and responsibilities in enforcing the legislation	(2) States the role and responsibilities of government in enforcement	"ARTICULO 2º — El Ministerio de Salud, a través del Instituto Nacional de Alimentos, será el organismo de control del cumplimiento de la presente ley." "ARTICULO 7º — Para la aplicación de la presente ley, el Ministerio de Salud ejercerá sus funciones por sí o en colaboración con otros organismos nacionales, provinciales y municipales, organizaciones no gubernamentales e instituciones internacionales." "ARTICULO 8º — El Ministerio de Salud, en el ámbito del Consejo Federal de Salud (COFESA), coordinará acciones con las autoridades sanitarias de las provincias y de la ciudad de Buenos Aires, para asegurar la implementación de la presente ley."
36. States incentives to start fortification	(0) Does not state	
37. States incentives to continue fortification, including ensuring compliance	(0) Does not state	
38. States penalties to compel compliance	(2) States any penalties	Penalties are not stated explicitly, but the reader is directed to another document where they are presumably stated: (Ref 1) "ARTICULO 6º —Las infracciones a la presente ley y a su reglamentación serán pasibles de las penalidades contempladas en el artículo 9º de la Ley 18.284 y sus modificatorias." (Ref 4) "Artículo 9.- (según texto modificado por el Decreto N° 341 del 24/02/92):<br>a)<br>1) Unifícanse las sanciones pecuniarias a aplicar a las infracciones cometidas contra las normas sanitarias identificadas en el Anexo I, en las sumas de pesos Mil ($ 1.000) a pesos Un Millón ($ 1.000.000), sin perjuicio de la aplicación de las restantes sanciones administrativas que cupieren y de las denuncias penales que se formularen cuando así correspondiere. La autoridad sanitaria de aplicación graduará los montos a aplicar en cada caso teniendo para ello presente los antecedentes del imputado, la gravedad de la falta y su proyección desde el punto de vista sanitario. En caso de reincidencia, atendiéndose a los mismos parámetros de graduación la sanción podrá establecerse en hasta el décuplo del valor impuesto a la infracción anterior."
39. Penalties are objectively defined (e.g. first penalty=$100, second penalty=$300)	(0) Penalties are not objectively laid out	
40. States that enforcement is required to include feedback and support to improve performance and correct noncompliance	(2) Requires any feedback/support to improve performance	"7.0. MEDIDAS CORRECTIVAS ...<br>El objetivo es detectar el problema de modo que no vuelva a manifestarse en producciones posteriores."
**LABORATORY**		
41. References required analytical assays for nutrients (e.g. Liquid chromatography-mass spectrometry for folic acid, atomic absorption for iron and zinc.)	(0) Does not state requirements	Only the iron spot test, used in a semi-quantitative manner, is mentioned for laboratory analysis: "APENDICE I-V PROCEDIMIENTO DE ANALISIS"
42. States recognition that laboratory results are subject to several sources of variation and do not provide conclusive evidence of compliance or noncompliance	(0) Does not state recognition	
43. Focuses on the quantitative analysis of "marker" micronutrients such as iron	(2) Focuses on quantitative analysis of marker micronutrient such as iron	Only the iron spot test, used in a semi-quantitative manner, is mentioned for laboratory analysis: "APENDICE I-V PROCEDIMIENTO DE ANALISIS"
**REPORTING**		

44. States how government monitoring results are shared with stakeholders	(2) States how results are shared with stakeholders	(Ref 2) Laboratory results are to be shared with government stakeholders every three months, and annually all results are compiled and broken down by site of production, site of monitoring and food type. 6.0 ANALISIS DE LABORATORIO "El laboratorio oficial de referencia será el DEPARTAMENTO de CONTROL Y DESARROLLO DEL INSTITUTO NACIONAL de ALIMENTOS el cual es responsable de las siguientes tareas: ... · Elaborar un informe trimestral con los resultados de las inspecciones y los análisis de laboratorio de la totalidad de muestras analizadas en todo el país recopilados por el Departamento de Vigilancia Alimentaria del INSTITUTO NACIONAL DE ALIMENTOS. Dicho informe será enviado a los Departamentos Legislación y Normatización, Vigilancia Alimentaria e Inspectoría del INSTITUTO NACIONAL de ALIMENTOS, a los organismos oficiales intervinientes y a la Comisión de Micronutrientes del Ministerio de Salud." ... "· Realizar anualmente el análisis estadístico cuantitativo de los niveles de hierro<br>y vitaminas de todos los alimentos controlados, por procedencia (producción nacional e importada); por puntos de control (molinos, aduanas, depósitos, sitios de venta y establecimientos elaboradores de alimentos farináceos) y por tipo de producto (harina, fideos, pan, galletas y galletitas, etc.)."

Australia Wheat

Element	Scoring (use drop-down menus)	Comments (open text)
**GENERAL**		
1. States that legislation applies to at least one food vehicle fit for human consumption (types/grades to be fortified)	(2) States at least one type fit for human consumption	This Standard defines a number of products composed of cereals and qualifies the use of the term 'bread'. It also requires the mandatory fortification of wheat flour for making bread with thiamin and folic acid (Australia only) and the mandatory replacement of non-iodised salt with iodised salt in bread in Australia and New Zealand.
2. States the public health objective; purpose and scope of legislation	(2) States the public health objective or general purpose of legislation	In May 2004, Australia...asked FSANZ to investigate mandatory fortification with folic acid as a possible means of reducing the incidence of Neural Tube Defects.
3. References latest available science or accepted international norms and recommendations, particularly for items that may not be covered in the country's documents	(0) Does not state	
4. Provides definitions that includes terms that are specific to fortification (e.g. fortified food, premix, fortificant, food vehicle)	(0) Does not state	
5. Provides repeals (if there is at least one prior document about fortification)	(2) States repeals	Amendment history "These amendments are made under section 92 of the Food Standards Australia New Zealand Act 1991 unless otherwise indicated. Amendments do not have a specific date for cessation unless indicated as such." (ref 3). "This is a compilation of Standard 2.1.1 as in force on 30 October 2014 (up to Amendment No. 150). It includes any commenced amendment affecting the compilation to that date." (ref 3). "Standard 2.1.1 – Cereals and Cereal Products requires that all wheat flour for making bread, with the exception of flour represented as organic, must be fortified with folic acid from 13 September 2009." (ref 4). "The current voluntary folic acid fortification permissions will not be affected by mandatory folic acid fortification, with the exception that the voluntary permission for adding folic acid to bread is being repealed and replaced with the mandatory requirement. Bread made from other cereal flour can be fortified under the voluntary fortification permissions (refer to Standard 1.3.2 – Vitamins and Minerals)." (ref 4).
6. Provides effective date or gives grace period for when fortification is to begin (e.g. effective 6 months from signing)	(2) States effective date or grace period for when fortification is to begin (e.g. effective 6 months from signing)	The mandatory Standard provides industry with two years to implement requirements and is enforceable from 13 September 2009. The current voluntary folic acid fortification permissions will not be affected by mandatory folic acid fortification, with the exception that the voluntary permission for adding folic acid to bread is being repealed and replaced with the mandatory requirement.
**MICRONUTRIENTS/PREMIX**		
7. States nutrients required	(2) States nutrients	(2) Wheat flour for making bread must contain – (a) no less than 2 mg/kg and no more than 3 mg/kg of folic acid; and (b) no less than 6.4 mg/kg of thiamin.
8. States fortificants (chemical compounds) to be used (including fortificants that are allowable as options)	(2) States fortificants for at least one nutrient	(2) Wheat flour for making bread must contain – (a) no less than 2 mg/kg and no more than 3 mg/kg of folic acid; and (b) no less than 6.4 mg/kg of thiamin.
9. States fortification levels	(2) States a range or number with +/-	(2) Wheat flour for making bread must contain – (a) no less than 2 mg/kg and no more than 3 mg/kg of folic acid; and (b) no less than 6.4 mg/kg of thiamin. The maximum limit for folic acid given in paragraph 4(2)(a) ensures the addition of folic acid to wheat flour for making bread in Australia is in controlled amounts to provide for a safe population intake of dietary folic acid.
10. States consideration of bioavailability/biological activity of fortificants	(0) Does not state any consideration	
11. States consideration of nutrient stability	(2) States consideration of nutrient stability	The level of folic acid will also need to remain within the range of 2-3 mg/kg over the declared shelf-life of the product. Millers should obtain their own information to verify the shelf-life of a product; however information available from overseas indicates a maximum degradation of folic acid in flour and bread pre-mixes of 10% over 12 months ' .
**COSTING**		
12. States that the cost of fortification is regulated through cost sharing schemes (between government, industry, consumers) or tax measures [to assist industry]	(0) Does not state any consideration	
13. States consideration of the financial responsibility of monitoring and enforcing fortification (schedule of fees, budget) [on the government side]	(0) Does not state any consideration	

**LABELING**		
14. Includes some sort of statement/label/logo that makes it clear that the product is fortified	(2) Includes a statement, label, or logo	Only applies to "food for retail sale": "Suppliers will be required to list folic acid either as folic acid or folate in the statement of ingredients on foods that contain wheat flour fortified with folic acid." (ref 4).
15. Provides guidance on health claims that can be made for this product (specific to micronutrients added through fortification)	(2) Provides guidance on health claims specific to micronutrients added through fortification	Clauses 4-9 describe in detail what can and cannot be claimed regarding fortified food, and how to make certain calculations that are relevant to determining if can make a claim or not.
**INTERNAL MONITORING (conducted by industry)**		
16. If samples are to be taken, describes the sampling process: for example, number of samples, amount, frequency, individual vs. composite, where samples are taken in the process, and percent considered passing	(2) States that samples should be taken as part of internal monitoring	Quality assurance sampling: mills<br>The flour miller is responsible for developing and implementing QAA procedures for<br>the collection of samples to verify that folic acid is being added to wheat flour for<br>making bread at the required level. Mills will need a disciplined approach to sampling<br>if they wish to use the results to demonstrate compliance. The components to be<br>considered include frequency, location and type and the following information acts as<br>a guide.
17. States that industry is required to follow quality assurance/quality control in regards to fortification	(2) States requirement of QA/QC for fortification	The Model is based on mills incorporating components designed to control compliance with mandatory folic acid fortification into a quality assurance arrangement (QAA), including monitoring, sampling and analysis protocols. Many mills already have a third party audited QAA in place, and in that case folic acid fortification would be an additional element.
18. States applicability of using qualitative testing to determine the presence or absence of a vitamin or mineral (spot tests, iChecks)	(0) Does not state	
**EXTERNAL MONITORING (conducted by government)**		
19.States requirement for external monitoring at the production site to assure compliance with standards and regulations	(2) States requirement for external monitoring or the need for audits/inspections	Assessing compliance with food standards is the responsibility of state and territory enforcement agencies. These agencies have developed a Compliance and Enforcement Model for mandatory folic acid fortification. Contact your local enforcement officers or state/territory health department for further information on this issue. The Model is based on mills incorporating components designed to control compliance with mandatory folic acid fortification into a quality assurance arrangement (QAA), including monitoring, sampling and analysis protocols. Many mills already have a third party audited QAA in place, and in that case folic acid fortification would be an additional element. Third party audit arrangements can be applied to demonstrate the QAA is in place and achieving the stated outcomes. However, the Model does not preclude enforcement agencies from taking samples for analysis should the need arise.
20. Describes protocols and systems for regulatory monitoring	(1) Does not explicitly describe, but references protocols and systems for regulatory monitoring	
21. If there are two or more government agencies involved in external monitoring, clarifies the roles and responsibilities between different government agencies in external monitoring	(2) Clarifies roles and responsibilities for more than one agency	Enforcement of mandatory folic acid fortification will be the responsibility of state and territory enforcement agencies. The onus is upon suppliers including food companies to ensure compliance with relevant food legislation.
22. Allows for monitoring to be conducted often enough that problems can be identified and addressed on a timely basis; specifies a timeline for inspections (i.e. once every 6 months, increasing to once every 2 months if a discrepancy is found)	(2) Describes frequency and how it is responsive to the needs of industry or the stage of fortification implementation	Frequency Sampling may be on a scheduled basis or triggered by events, such as a mill's failure to take agreed samples, inadequate record keeping or failure to demonstrate corrective actions when problems are identified.
23. If samples are to be taken, describes the sampling process: for example, number of samples, amount, frequency, individual vs. composite, where samples are taken in the process, and percent considered passing	(2) States that samples should be taken as part of external monitoring	Sampling and laboratory analysis to check that the flour contains the required<br>amount of folic acid. Suggested sampling and analysis protocols are outlined<br>below. Quality assurance sampling: mills<br>The flour miller is responsible for developing and implementing QAA procedures for the collection of samples to verify that folic acid is being added to wheat flour for making bread at the required level. Mills will need a disciplined approach to sampling if they wish to use the results to demonstrate compliance. The components to be considered include frequency, location and type and the following information acts as a guide.

24. States applicability of using qualitative testing to determine the presence or absence of a vitamin or mineral (spot tests, iChecks)	(0) Does not state	
25. States registration is required in order to use a logo/be licensed to produce fortified foods	(0) Does not state that registration or licensing is required	
**COMMERCIAL MONITORING (conducted by government)**		
26. Provides justification for commercial monitoring at retail stores	(0) Does not provide justification for commercial monitoring	
27. Describes protocols and systems for commercial monitoring	(0) Does not describe	
28. If there are two or more government agencies involved in commercial monitoring, clarifies the roles and responsibilities between different government agencies in commercial monitoring	(0) Clarifies roles and responsibilities for no agencies	
29. Allows for monitoring to be conducted often enough that problems at the production site or import companies can be identified and addressed on a timely basis; specifies a timeline for inspections (i.e. once every 6 months) or works with production companies to correct noncompliance	(N/A) No commercial monitoring occurs	
30. If samples are to be taken, describes the sampling process: for example, number of samples, amount, frequency, individual vs. composite, where samples are taken in the process, and percent considered passing	(N/A) Does not describe the sampling process	
**IMPORT MONITORING (conducted by government)**		
31. Provides justification for import monitoring at points of entry	(2) Provides justification for import monitoring	Imported wheat flour for making bread must comply with the mandatory folic acid fortification Standard, with the exception of flour represented as organic.
32. Describes protocols and systems for import monitoring	(0) Does not state	
33. If there are two or more government agencies involved in import monitoring, clarifies the roles and responsibilities between different government agencies in import monitoring	(0) Clarifies roles and responsibilities for no agencies	
34. If samples are to be taken, describes the sampling process: for example, number of samples, amount, frequency, individual vs. composite, where samples are taken in the process, and percent considered passing	(N/A) Does not describe the sampling process	
**ENFORCEMENT/PENALTIES**		
35. Indicates roles and responsibilities in enforcing the legislation	(2) States the role and responsibilities of government in enforcement	Assessing compliance with food standards is the responsibility of state and territory enforcement agencies. These agencies have developed a Compliance and Enforcement Model for mandatory folic acid fortification. The Model is based on mills incorporating components designed to control compliance with mandatory folic acid fortification into a quality assurance arrangement (QAA), including monitoring, sampling and analysis protocols. Many mills already have a third party audited QAA in place, and in that case folic acid fortification would be an additional element. Third party audit arrangements can be applied to demonstrate the QAA is in place and achieving the stated outcomes. However, the Model does not preclude enforcement agencies from taking samples for analysis should the need arise.
36. States incentives to start fortification	(0) Does not state	
37. States incentives to continue fortification, including ensuring compliance	(0) Does not state	
38. States penalties to compel compliance	(0) Does not state	
39. Penalties are objectively defined (e.g. first penalty=$100, second penalty=$300)	(N/A) No penalties are stated (Answered (0) to previous question)	
40. States that enforcement is required to include feedback and support to improve performance and correct noncompliance	(0) Does not require	
**LABORATORY**		
41. References required analytical assays for nutrients (e.g. Liquid chromatography-mass spectrometry for folic acid, atomic absorption for iron and zinc.)	(2) References required assays	Does not require, but suggests using kits (Biocore, protein binding assay, and ELISA) as a method of QA

42. States recognition that laboratory results are subject to several sources of variation and do not provide conclusive evidence of compliance or noncompliance	(2) States recognition that lab results are subject to variation	Enforcement agencies should ensure any samples taken at a mill are reflective of product being produced and should include a composite sample that is representative of a 'lot' and should account for laboratory and analytical variability. Results of samples taken by enforcement agencies should be considered in conjunction with information available at the mill that demonstrates the correct amount of folic acid is being added e.g. by examining sampling records and processes and evidence the required range was being achieved to the appropriate level.
43. Focuses on the quantitative analysis of "marker" micronutrients such as iron	(2) Focuses on quantitative analysis of marker micronutrient such as iron	Folic acid, thiamine (and iodine, through the use of iodized salt) should be added to flour used to make bread. Only analysis of folic acid is described for mills and regulatory authorities.
**REPORTING**		
44. States how government monitoring results are shared with stakeholders	(0) Does not state how results are shared	The Australian Institute of Health and Welfare (AIHW) has been given overall responsibility for the monitoring program and reporting on progress.

Belize Wheat

Element	Scoring (use drop-down menus)	Comments (open text)
**GENERAL**		
1. States that legislation applies to at least one food vehicle fit for human consumption (types/grades to be fortified)	(2) States at least one type fit for human consumption	This standard applies to wheat flour (also called flour, white flour, enriched flour) and whole wheat flour that is intended for human consumption, made from clean, sound and marketable grades of millable wheat grains of the species Triticum aestivum L. (common wheat) or from Triticum compactum Host (club wheat)
2. States the public health objective; purpose and scope of legislation	(2) States the public health objective or general purpose of legislation	This standard sets out the requirements for wheat flour, and describes the methods of sampling and testing that should be used. Wheat flour is now manufactured in Belize and local supplies have now replaced imports from other countries. As wheat flour is an important nutritional input in domestic cookery, and in institutional or commercial baking as agrees and acceptable level of identity, purity, quality, acceptability, and nutrient value is desirable so that flour can move freely within the region.
3. References latest available science or accepted international norms and recommendations, particularly for items that may not be covered in the country's documents	(0) Does not state	References Codex, but not in relation to fortification. Says Belize standard has been adapted from Caribbean Community standard. References FAO/WHO Joint Expert Committee on Food Additives
4. Provides definitions that includes terms that are specific to fortification (e.g. fortified food, premix, fortificant, food vehicle)	(2) States at least one term related to fortification	Enriched wheat flour is wheat flour containing vitamins and irons, in the proportions specified in Table 2. Where there is a signficant nutritional deficiency of calcium, calcium may be added within the limits specified in Table 2.
5. Provides repeals (if there is at least one prior document about fortification)	(N/A) No prior documents about fortification	Adapted from Caribbean Community Standards
6. Provides effective date or gives grace period for when fortification is to begin (e.g. effective 6 months from signing)	(0) Does not state	
**MICRONUTRIENTS/PREMIX**		
7. States nutrients required	(2) States nutrients	Thiamine, riboflavin, niacin, iron, calcium (optional), folic acid
8. States fortificants (chemical compounds) to be used (including fortificants that are allowable as options)	(0) Does not state	
9. States fortification levels	(2) States a range or number with +/-	States a range for calcium
10. States consideration of bioavailability/biological activity of fortificants	(0) Does not state any consideration	
11. States consideration of nutrient stability	(2) States consideration of nutrient stability	Wheat flour shall be packed in containers, which will safeguard the hygienic, nutritional, and technological characteristics of the flour, and protect it against contamination.
**COSTING**		
12. States that the cost of fortification is regulated through cost sharing schemes (between government, industry, consumers) or tax measures [to assist industry]	(0) Does not state any consideration	
13. States consideration of the financial responsibility of monitoring and enforcing fortification (schedule of fees, budget) [on the government side]	(0) Does not state any consideration	
**LABELING**		
14. Includes some sort of statement/label/logo that makes it clear that the product is fortified	(2) Includes a statement, label, or logo	Where the vitamins and minerals mentioned in Table 2 are present in the flour, the amount of each present in 100 g of flour (at 14% moisture content) shall be declared, expressed in milligrams (mg), and the flour may be described as "enriched" or "vitamin enriched," and as "calcium enriched" if the level of calcium is in accordance with Table 2 and represents 16.6 percent of the Recommended Daily Allowance set by the Caribbean Food and Nutrition Institute.
15. Provides guidance on health claims that can be made for this product (specific to micronutrients added through fortification)	(0) Does not provide	
**INTERNAL MONITORING (conducted by industry)**		
16. If samples are to be taken, describes the sampling process: for example, number of samples, amount, frequency, individual vs. composite, where samples are taken in the process, and percent considered passing	(1) States that samples should be taken (generally)	The methods of sampling which should be used will depend on whether the wheat flour is: in a consignment of retail packages (up to 25 kg) in a shipment or in a warehouse; in bulk packages (over 25 kg, in a freight container or road or rail tanker); and discharged in a stream from a bulk container, or in production in the mill in a stream moving towards the final packaging operation. See section 11.2.4: Samples shall be taken as follows...

17. States that industry is required to follow quality assurance/quality control in regards to fortification	(2) States requirement of QA/QC for fortification	To be eligible for a licence to use the Caricom Standard Mark, the manufacturer of wheat flour shall operate a quality assurance system conforming to the general requirements of ISO 9002-1994, using adequate staff, sampling procedures and testing equipment, as approved by the Belize Bureau of Standards. It is recommended that wheat flour be produced under an approved quality assurance system.
18. States applicability of using qualitative testing to determine the presence or absence of a vitamin or mineral (spot tests, iChecks)	(0) Does not state	
**EXTERNAL MONITORING (conducted by government)**		
19.States requirement for external monitoring at the production site to assure compliance with standards and regulations	(0) Does not state requirement	
20. Describes protocols and systems for regulatory monitoring	(0) Does not describe	
21. If there are two or more government agencies involved in external monitoring, clarifies the roles and responsibilities between different government agencies in external monitoring	(0) Clarifies roles and responsibilities for no agencies	
22. Allows for monitoring to be conducted often enough that problems can be identified and addressed on a timely basis; specifies a timeline for inspections (i.e. once every 6 months, increasing to once every 2 months if a discrepancy is found)	(0) Does not state	
23. If samples are to be taken, describes the sampling process: for example, number of samples, amount, frequency, individual vs. composite, where samples are taken in the process, and percent considered passing	(N/A) Does not describe the sampling process	The methods of sampling which should be used will depend on whether the wheat flour is: in a consignment of retail packages (up to 25 kg) in a shipment or in a warehouse; in bulk packages (over 25 kg, in a freight container or road or rail tanker); and discharged in a stream from a bulk container, or in production in the mill in a stream moving towards the final packaging operation. See section 11.2.4: Samples shall be taken as follows...
24. States applicability of using qualitative testing to determine the presence or absence of a vitamin or mineral (spot tests, iChecks)	(0) Does not state	
25. States registration is required in order to use a logo/be licensed to produce fortified foods	(0) Does not state that registration or licensing is required	10.1 To be eligible for a license for the CARICOM mark…
**COMMERCIAL MONITORING (conducted by government)**		
26. Provides justification for commercial monitoring at retail stores	(0) Does not provide justification for commercial monitoring	
27. Describes protocols and systems for commercial monitoring	(1) Does not explicitly describe, but references protocols and systems for regulatory monitoring	
28. If there are two or more government agencies involved in commercial monitoring, clarifies the roles and responsibilities between different government agencies in commercial monitoring	(0) Clarifies roles and responsibilities for no agencies	
29. Allows for monitoring to be conducted often enough that problems at the production site or import companies can be identified and addressed on a timely basis; specifies a timeline for inspections (i.e. once every 6 months) or works with production companies to correct noncompliance	(N/A) No commercial monitoring occurs	
30. If samples are to be taken, describes the sampling process: for example, number of samples, amount, frequency, individual vs. composite, where samples are taken in the process, and percent considered passing	(2) States that samples should be taken as part of commercial monitoring	The methods of sampling which should be used will depend on whether the wheat flour is: in a consignment of retail packages (up to 25 kg) in a shipment or in a warehouse; in bulk packages (over 25 kg, in a freight container or road or rail tanker); and discharged in a stream from a bulk container, or in production in the mill in a stream moving towards the final packaging operation. See section 11.2.4: Samples shall be taken as follows...
**IMPORT MONITORING (conducted by government)**		
31. Provides justification for import monitoring at points of entry	(0) Does not provide justification for import monitoring	
32. Describes protocols and systems for import monitoring	(0) Does not state	
33. If there are two or more government agencies involved in import monitoring, clarifies the roles and responsibilities between different government agencies in import monitoring	(0) Clarifies roles and responsibilities for no agencies	
34. If samples are to be taken, describes the sampling process: for example, number of samples, amount, frequency, individual vs. composite, where samples are taken in the process, and percent considered passing	(N/A) Does not describe the sampling process	

**ENFORCEMENT/PENALTIES**		
35. Indicates roles and responsibilities in enforcing the legislation	(0) Does not state	
36. States incentives to start fortification	(0) Does not state	
37. States incentives to continue fortification, including ensuring compliance	(0) Does not state	
38. States penalties to compel compliance	(0) Does not state	
39. Penalties are objectively defined (e.g. first penalty=$100, second penalty=$300)	(N/A) No penalties are stated (Answered (0) to previous question)	
40. States that enforcement is required to include feedback and support to improve performance and correct noncompliance	(0) Does not require	
**LABORATORY**		
41. References required analytical assays for nutrients (e.g. Liquid chromatography-mass spectrometry for folic acid, atomic absorption for iron and zinc.)	(2) References required assays	Referenced in section 12.3
42. States recognition that laboratory results are subject to several sources of variation and do not provide conclusive evidence of compliance or noncompliance	(0) Does not state recognition	
43. Focuses on the quantitative analysis of "marker" micronutrients such as iron	(0) Does not state	
**REPORTING**		
44. States how government monitoring results are shared with stakeholders	(0) Does not state how results are shared	

Bolivia Wheat

Element	Scoring (use drop-down menus)	Comments (open text)
**GENERAL**		
1. States that legislation applies to at least one food vehicle fit for human consumption (types/grades to be fortified)	(2) States at least one type fit for human consumption	"Artículo 2. Obligatoriedad de fortificación. Toda harina de trigo, premezclas de micronutrientes y mezclas a base de harina de trigo producidas en el país, importadas o donadas, que se destine a la venta, donación directa y elaboración de productos derivados, deberán estar fortificados con hierro (sulfato ferroso anhidro), ácido fólico y vitaminas del complejo B..." "Artículo 5. Niveles de la fortificación y procedimiento. ... Toda harina de trigo y mezclas a base de harina de trigos destinados al consumo humano que se encuentre a disposición en el territorio nacional deberán estar fortificados con los siguientes niveles de micronutrientes..."
2. States the public health objective; purpose and scope of legislation	(2) States the public health objective or general purpose of legislation	In introduction to law "Que se ha demostrado que la principal causa de la anemia, en especial en mujeres embarazadas y niños menores de tres años, es el aporte insuficiente de hierro y folato en la alimentación diaria de la mayor parte de nuestra población;<br>Que la forma más eficiente y sostenible para combatir y prevenir la carencia de hierro es fortificando los alimentos de consumo masivo en la población; "
3. References latest available science or accepted international norms and recommendations, particularly for items that may not be covered in the country's documents	(0) Does not state	
4. Provides definitions that includes terms that are specific to fortification (e.g. fortified food, premix, fortificant, food vehicle)	(2) States at least one term related to fortification	"Artículo 19. Glosario de definiciones."
5. Provides repeals (if there is at least one prior document about fortification)	(2) States repeals	"ARTICULO 6°.- DEROGACIONES<br>Se deroga toda disposición contraria al presente Decreto Supremo."
6. Provides effective date or gives grace period for when fortification is to begin (e.g. effective 6 months from signing)	(2) States effective date or grace period for when fortification is to begin (e.g. effective 6 months from signing)	"ARTICULO 4°.- PLAZO DE ADECUACION<br>En el plazo de un año a partir de la fecha de promulgación del presente Decreto Supremo, la harina de trigo de producción nacional, importada y de donación, deberá adecuarse al proceso de fortificación establecido en los artículos precedentes."
**MICRONUTRIENTS/PREMIX**		
7. States nutrients required	(2) States nutrients	Article 4: vitamin B1, B2, niacin, folic acid, iron.
8. States fortificants (chemical compounds) to be used (including fortificants that are allowable as options)	(2) States fortificants for at least one nutrient	Articulo Segundo "el micronutriente de la premezcla para fortificar la harina de trigo para consumo humano es el SULFATO FERROSO ANHIDRO".
9. States fortification levels	(1) States one number only	Article 4: States levels of vitamin B1, B2, niacin, folic acid, and iron that must be in premix (g/kg). Article 5 states minimum levels of nutrients that must be present in wheat flour and in "mixtures made from wheat flour" (mg/kg). Article 5 states that premix should be added at a rate of 200 g per ton of flour (or flour mix).
10. States consideration of bioavailability/biological activity of fortificants	(2) States some consideration of bioavailability (mentions these or related terms)	Vistos y considerando, párafo 5
11. States consideration of nutrient stability	(2) States consideration of nutrient stability	Vistos y considerando, párafo 5
**COSTING**		
12. States that the cost of fortification is regulated through cost sharing schemes (between government, industry, consumers) or tax measures [to assist industry]	(0) Does not state any consideration	
13. States consideration of the financial responsibility of monitoring and enforcing fortification (schedule of fees, budget) [on the government side]	(0) Does not state any consideration	
**LABELING**		
14. Includes some sort of statement/label/logo that makes it clear that the product is fortified	(2) Includes a statement, label, or logo	Artículo 13. Del etiquetado.
15. Provides guidance on health claims that can be made for this product (specific to micronutrients added through fortification)	(0) Does not provide	
**INTERNAL MONITORING (conducted by industry)**		
16. If samples are to be taken, describes the sampling process: for example, number of samples, amount, frequency, individual vs. composite, where samples are taken in the process, and percent considered passing	(N/A) Does not describe the sampling process	

17. States that industry is required to follow quality assurance/quality control in regards to fortification	(2) States requirement of QA/QC for fortification	"Artículo 5. Niveles de la fortificación y procedimiento. Los niveles de adición de micronutrientes…siendo responsabilidad de los industriales molineros, de los importadores y de las entidades donadoras de harina de trigo y mezclas a base de harina de trigo, cumplir obligatoriamente con su aplicación."
18. States applicability of using qualitative testing to determine the presence or absence of a vitamin or mineral (spot tests, iChecks)	(0) Does not state	
**EXTERNAL MONITORING (conducted by government)**		
19.States requirement for external monitoring at the production site to assure compliance with standards and regulations	(2) States requirement for external monitoring or the need for audits/inspections	"Artículo 9. De las responsabilidades. a) Control de la fortificación en las industrias nacionales. El Ministerio de Salud y Deportes, a través del INLASA y los SEDES del país, efectuaran el control de la fortificación de las industrias que elaboran Harina de trigo y mezclas a base de harina de trigos fortificados, en el marco del Sistema de Control de Calidad."
20. Describes protocols and systems for regulatory monitoring	(1) Does not explicitly describe, but references protocols and systems for regulatory monitoring	
21. If there are two or more government agencies involved in external monitoring, clarifies the roles and responsibilities between different government agencies in external monitoring	(N/A) Only one government agency involved	"Artículo 9. De las responsabilidades. a) Control de la fortificación en las industrias nacionales. El Ministerio de Salud y Deportes, a través del INLASA y los SEDES del país, efectuaran el control de la fortificación de las industrias que elaboran Harina de trigo y mezclas a base de harina de trigos fortificados, en el marco del Sistema de Control de Calidad."
22. Allows for monitoring to be conducted often enough that problems can be identified and addressed on a timely basis; specifies a timeline for inspections (i.e. once every 6 months, increasing to once every 2 months if a discrepancy is found)	(0) Does not state	
23. If samples are to be taken, describes the sampling process: for example, number of samples, amount, frequency, individual vs. composite, where samples are taken in the process, and percent considered passing	(1) States that samples should be taken (generally)	Does not provide a great level of detail on sampling. "Artículo 7 … Producción: Determinación cuantitativa en muestras combinadas de la producció de un día y almacén." Prior to this text there is a table stating the "quality criteria for wheat flour and mixes based on wheat flour". For external monitoring, the indicator is "sulfato ferroso anhidro" and the addition level is ">=30 ppm". "Artículo 8. Del Muestreo. Cuando se requiera la toma de muestra esta debe ser solo en la cantidad estrictamente necesaria para el análisis que corresponda."
24. States applicability of using qualitative testing to determine the presence or absence of a vitamin or mineral (spot tests, iChecks)	(0) Does not state	Article 7 states that quantitative tests should be conducted of iron.
25. States registration is required in order to use a logo/be licensed to produce fortified foods	(0) Does not state that registration or licensing is required	
**COMMERCIAL MONITORING (conducted by government)**		
26. Provides justification for commercial monitoring at retail stores	(2) Provides justification for commercial monitoring	Artículo 9. De las responsabilidades. … El Ministerio de Salud y Deportes verificara el cumplimiento de los niveles de fortificación en el marco de un Sistema de Control de Calidad. c) Control en los puntos de comercialización. Los Gobiernos Municipales, a través de las Intendencias serán las responsables de verificar la higiene, peso y venta de harina de trigo y mezclas a base de harina de trigo fortificado."
27. Describes protocols and systems for commercial monitoring	(1) Does not explicitly describe, but references protocols and systems for regulatory monitoring	
28. If there are two or more government agencies involved in commercial monitoring, clarifies the roles and responsibilities between different government agencies in commercial monitoring	(N/A) Only one government agency involved	Artículo 9. De las responsabilidades. … El Ministerio de Salud y Deportes verificara el cumplimiento de los niveles de fortificación en el marco de un Sistema de Control de Calidad. c) Control en los puntos de comercialización. Los Gobiernos Municipales, a través de las Intendencias serán las responsables de verificar la higiene, peso y venta de harina de trigo y mezclas a base de harina de trigo fortificado."
29. Allows for monitoring to be conducted often enough that problems at the production site or import companies can be identified and addressed on a timely basis; specifies a timeline for inspections (i.e. once every 6 months) or works with production companies to correct noncompliance	(0) Does not state	

30. If samples are to be taken, describes the sampling process: for example, number of samples, amount, frequency, individual vs. composite, where samples are taken in the process, and percent considered passing	(1) States that samples should be taken (generally)	Does not provide a great level of detail on sampling. "Artículo 7 … Monitoreo: Determinación cuantitativa en muestras individuales de cada marca existente en lugares de expendio." Prior to this text there is a table stating the "quality criteria for wheat flour and mixes based on wheat flour". For commercial monitoring, the indicator is "sulfato ferroso anhidro" and the addition level is ">=30 ppm". "Artículo 8. Del Muestreo. Cuando se requiera la toma de muestra esta debe ser solo en la cantidad estrictamente necesaria para el análisis que corresponda."
**IMPORT MONITORING (conducted by government)**		
31. Provides justification for import monitoring at points of entry	(2) Provides justification for import monitoring	"Artículo 2. Obligatoriedad de fortificación. Toda harina de trigo, premezclas de micronutrientes y mezclas a base de harina de trigo producidas en el país, importadas o donadas, que se destine a la venta, donación directa y elaboración de productos derivados, deberán estar fortificados con hierro (sulfato ferroso anhidro), ácido fólico y vitaminas del complejo B..."
32. Describes protocols and systems for import monitoring	(2) Includes checklists or detailed description of import monitoring procedures	"Artículo 15. De la importación y donaciones. Para la obtención del certificado de inocuidad alimentaria de importación emitido por el SENASAG, debe presentar entre otros requisitos documentales establecidos en normativa vigente, el certificado sanitario de origen y un certificado que avale la fortificación, emitido por un laboratorio de referencia o laboratorios autorizados y/o reconocidos por la autoridad sanitaria de origen, que certifique los niveles establecidos en este reglamento."
33. If there are two or more government agencies involved in import monitoring, clarifies the roles and responsibilities between different government agencies in import monitoring	(N/A) Only one government agency involved	"Artículo 9. De las responsabilidades. b) Control de la importación de productos fortificados. El SENASAG otorgará el certificado de inocuidad alimentaria de importación, para lo cual el importador deberá presentar entre otros requisitos documentales establecidos en normativa vigente, el certificado sanitario de origen y un certificado que avale la fortificación, emitido por un laboratorio de referencia o laboratorios autorizados y/o reconocidos por la autoridad sanitaria de origen, que certifique los niveles establecidos en este reglamento."
34. If samples are to be taken, describes the sampling process: for example, number of samples, amount, frequency, individual vs. composite, where samples are taken in the process, and percent considered passing	(1) States that samples should be taken (generally)	Does not provide a great level of detail on sampling. "Artículo 7 … Importaciones (c) Auditorías de calidad: Determinación cuantitativa en muestras individuales aleatorias. En el caso de las Auditorías de Calidad, el número total de muestras se obtendrá siguiendo criterios estadísticos de muestreo." Prior to this text there is a table stating the "quality criteria for wheat flour and mixes based on wheat flour". For import monitoring, the indicator is "sulfato ferroso anhidro" and the addition level is ">=30 ppm". "Artículo 8. Del Muestreo. Cuando se requiera la toma de muestra esta debe ser solo en la cantidad estrictamente necesaria para el análisis que corresponda."
**ENFORCEMENT/PENALTIES**		
35. Indicates roles and responsibilities in enforcing the legislation	(2) States the role and responsibilities of government in enforcement	"Artículo 9. De las responsabilidades."
36. States incentives to start fortification	(0) Does not state	
37. States incentives to continue fortification, including ensuring compliance	(0) Does not state	
38. States penalties to compel compliance	(2) States any penalties	"Artículo 16. De las contravenciones."
39. Penalties are objectively defined (e.g. first penalty=$100, second penalty=$300)	(0) Penalties are not objectively laid out	"Artículo 16. De las contravenciones. … los Servicios Departamentales de Salud son los responsables de aplicar las sanciones de acuerdo a legislación vigente."
40. States that enforcement is required to include feedback and support to improve performance and correct noncompliance	(0) Does not require	
**LABORATORY**		
41. References required analytical assays for nutrients (e.g. Liquid chromatography-mass spectrometry for folic acid, atomic absorption for iron and zinc.)	(0) Does not state requirements	Article 10 says that INLASA is the coordinator of the micronutrient laboratory netwwork and will establish techncial procedures and lab methods for the qualitative and quantitative analysis of flour samples and samples made from mixes of flour.
42. States recognition that laboratory results are subject to several sources of variation and do not provide conclusive evidence of compliance or noncompliance	(0) Does not state recognition	

43. Focuses on the quantitative analysis of "marker" micronutrients such as iron	(2) Focuses on quantitative analysis of marker micronutrient such as iron	"Artículo 7. Del Sistema de Control de Calidad para la verificación de la fortificación de la harina de trigo y mezclas a bases de harina de trigo." The table only lists the quantification of iron to determine if samples have been fortified for external, import and commercial monitoring.
**REPORTING**		
44. States how government monitoring results are shared with stakeholders	(0) Does not state how results are shared	

Brazil Wheat

Element	Scoring (use drop-down menus)	Comments (open text)
**GENERAL**		
1. States that legislation applies to at least one food vehicle fit for human consumption (types/grades to be fortified)	(2) States at least one type fit for human consumption	Mandating wheat flour and corn flour fortification with the iron and folic acid. This Technical Regulation applies to the mandatory fortification of wheat flour and corn flour with the iron and folic acid. Excluded from this regulation, due to technological processing, are the following products: tapioca flour or corn flower obtained by maceration; flakes; whole wheat flour and durum wheat flour.
2. States the public health objective; purpose and scope of legislation	(2) States the public health objective or general purpose of legislation	Considering that the nutritional anemia caused by iron deficiency is a serious problem on Public Health, it is the nutritional deficiency of greater magnitude in the world, affecting all cycles of life and in Brazil affects around 21% of children under 5 years of age and around 30% of women in their fertile age (PNDS, 2006);<br><br>Considering that, on children, the anemia is associated to the slow growth, to the cognitive deficit and to the low resistance to infections, and on pregnant women is related to the low weight of the fetus when born and also due to the increase on baby mortality;<br><br>Considering that the strategies for intervention recommended by WHO, ratified by Ministry of Health, to control the anemia caused by iron deficiency are education and nutritional guidance, reinforced foods and universal iron supplements for the more vulnerable groups.<br>considering the recommendations from the World Health Organization-WHO and the Pan American Health Organization-PAHO for fortification of food products with iron and folic acid;
3. References latest available science or accepted international norms and recommendations, particularly for items that may not be covered in the country's documents	(2) States the documents referenced	References: 3.18. The Prevention of Neural Tube Defects with Folic Acid. Pan American Health Organization / Word Health Organization, Division of Health Promotion and Protection, Food and Nutrition Program. Centers for Disease Control and Prevention, Birth Defects and Pediatric Genetics-CDC. P. 5-15. 3.19. Iron Fortification: Where Are We in Terms of Iron Compounds PAHO / FNP / USAID Technical Consultation. Nutrition Reviews, Vol. 60, No. 7 (part II), July. 2002 p.61.
4. Provides definitions that includes terms that are specific to fortification (e.g. fortified food, premix, fortificant, food vehicle)	(2) States at least one term related to fortification	Mix is a mixture where the desired ingredient (in this case, iron) is diluted into another product (called the vehicle), so that its application or use is facilitated. Bio-availability is the degree to which specific nutrients found¬ in the foods are used, with reference to the total content (100%) of the ¬main nutrient considered. (MS Ordinance 710/GM of June 10, 1999).
5. Provides repeals (if there is at least one prior document about fortification)	(2) States repeals	Art.9 – this Ordinance enters into force on the date of its publication.<br>Art.10 – The Ordinance Nº 14/GM, January 3, 2000 is revoked, published on Diário Oficial da União nº5, January 8, 2002, section 1, page 59.
6. Provides effective date or gives grace period for when fortification is to begin (e.g. effective 6 months from signing)	(2) States effective date or grace period for when fortification is to begin (e.g. effective 6 months from signing)	Art.9 – this Ordinance enters into force on the date of its publication.<br>Art.10 – The Ordinance Nº 14/GM, January 3, 2000 is revoked, published on Diário Oficial da União nº5, January 8, 2002, section 1, page 59. In another document: Art. 2 Businesses have a term of 18 (eighteen) months from the date of publication of this Regulation to adapt their products
**MICRONUTRIENTS/PREMIX**		
7. States nutrients required	(2) States nutrients	4.1. The addition of iron and folic acid in wheat flour and the corn meal that is pre-packaged in the customer's absence and ready to offer to the consumer, those for industrial use, including baking and flour added to pre-mixtures is mandatory, and each 100g of wheat flour and corn flour should provide at least 4.2 mg (four point two milligrams) of iron and 150 micrograms (one hundred fifty micrograms) of folic acid.

8. States fortificants (chemical compounds) to be used (including fortificants that are allowable as options)	(2) States fortificants for at least one nutrient	4.5. Companies may use the following food-grade iron compounds: dehydrated iron sulfate (dry); ferrous fumarate; reduced iron - 325 mesh Tyler; electrolytic iron - 325 mesh Tyler; Sodium and iron EDTA (NaFeEDTA); and iron glycinate chelate.<br>Other compounds may be used provided that the bioavailability is not inferior to the compounds listed.
9. States fortification levels	(1) States one number only	4.1. The addition of iron and folic acid in wheat flour and the corn meal that is pre-packaged in the customer's absence and ready to offer to the consumer, those for industrial use, including baking and flour added to pre-mixtures is mandatory, and each 100g of wheat flour and corn flour should provide at least 4.2 mg (four point two milligrams) of iron and 150 micrograms (one hundred fifty micrograms) of folic acid.
10. States consideration of bioavailability/biological activity of fortificants	(2) States some consideration of bioavailability (mentions these or related terms)	4.4. Companies should ensure that the food grade iron compounds are bioavailable. AND Bio-availability- this is the proportion of the ingredient ingested that is available for the metabolic process and varies for each type of iron. Ferrous sulfate and fumarate have good bio-availability, however, elemental ¬iron is considered lower. Bio-availability also varies from person to person, since many factors, such as the food itself, the individual's diet and the way in which the product is processed, affect the organism's ability to absorb different types of iron.
11. States consideration of nutrient stability	(2) States consideration of nutrient stability	4.6. Companies should use food-grade folic acid, ensuring the stability of the wheat flour and the corn flour within the period of validity of the same. b) Stability - in the ferrous sulfate and fumarate, there are pro-oxidants that can accelerate the development of rancidity in fats. Since flours have small quantities of fats, the addition of ferrous sulfate or fumarate can reduce its shelf-life.
**COSTING**		
12. States that the cost of fortification is regulated through cost sharing schemes (between government, industry, consumers) or tax measures [to assist industry]	(2) States consideration of either cost regulation method	To plan an iron-added wheat flour fortification program, it is important to determine the costs involved in the proper allocation of resources. In Brazil, since this program is mandatory, the funding regarding the disclosure and clarifications to the consumers about the iron-added flours and the difference they make compared to previously commercialized flours should be borne by the government itself. The costs to establish a national iron fortification program will depend on factors such as the number and size of the mills, the adoption of the quality guarantee system, regulations on the subject and rigor in inspecting the foods as well as the level of iron to be added.<br>For the fortification of flours, the necessary capital is basically concentrated in three items: 1) the iron source, 2) the iron dispenser/feeder and 3) quality control analyses. A list of some of the national suppliers of iron sources (Annex VI), equipment (Annex VII) and laboratories that can perform the necessary analyses (Annex VIII) are presented in the annexes.
13. States consideration of the financial responsibility of monitoring and enforcing fortification (schedule of fees, budget) [on the government side]	(2) Shows consideration that monitoring costs money	...should be borne by the government itself. The costs to establish a national iron fortification program will depend on factors such as the number and size of the mills, the adoption of the quality guarantee system, regulations on the subject and rigor in inspecting the foods as well as the level of iron to be added.<br>For the fortification of flours, the necessary capital is basically concentrated in three items: 1) the iron source, 2) the iron dispenser/feeder and 3) quality control analyses. ALSO see Table 4, part b for breakdown of expected monitoring costs
**LABELING**		
14. Includes some sort of statement/label/logo that makes it clear that the product is fortified	(2) Includes a statement, label, or logo	5. LABELLING<br>5.1. Wheat flours and corn flours should be designated using the conventional name of the product according to the specific legislation, followed by one of the following expressions: Fortified with iron and folic acid, enriched with iron and folic acid or rich in iron and folic acid.
15. Provides guidance on health claims that can be made for this product (specific to micronutrients added through fortification)	(2) Provides guidance on health claims specific to micronutrients added through fortification	According to this ordinance, the use of any expression of a therapeutic nature on the label of essential nutrient added foods is prohibited since these foods follow the General Labeling Standards, according to the specific legislation.

**INTERNAL MONITORING (conducted by industry)**		
16. If samples are to be taken, describes the sampling process: for example, number of samples, amount, frequency, individual vs. composite, where samples are taken in the process, and percent considered passing	(2) States that samples should be taken as part of internal monitoring	Quality control<br>6.2.1. Sampling<br>All flour samples should be collected after they have been packaged. The random collection of at least 3 (three) packages (of 1 or 5kg) from each -sample is recommended. For 50kg sacks, a sample of about 1kg can be taken from 3 sacks, or directly from the mouth of the bagger, for non-automatic systems.
17. States that industry is required to follow quality assurance/quality control in regards to fortification	(2) States requirement of QA/QC for fortification	Entire manual is about QA/QC
18. States applicability of using qualitative testing to determine the presence or absence of a vitamin or mineral (spot tests, iChecks)	(2) States applicability of spot test to determine presence/absence of vitamin or mineral specific to internal monitoring	3) Spot Test: all qualitative or semi-quantitative evaluation (Annex III) of the flour should be performed just after the sample collection so that any problem, such as excess or insufficient adding, can be corrected. Not only should the iron be observed, but also its homogenous distribution in the flour as well.
**EXTERNAL MONITORING (conducted by government)**		
19.States requirement for external monitoring at the production site to assure compliance with standards and regulations	(0) Does not state requirement	
20. Describes protocols and systems for regulatory monitoring	(0) Does not describe	
21. If there are two or more government agencies involved in external monitoring, clarifies the roles and responsibilities between different government agencies in external monitoring	(2) Clarifies roles and responsibilities for more than one agency	Articles 3-5 in English Portaria No 1793 Document. Details responsibilities of the MoH, Commission, and ANVISA.
22. Allows for monitoring to be conducted often enough that problems can be identified and addressed on a timely basis; specifies a timeline for inspections (i.e. once every 6 months, increasing to once every 2 months if a discrepancy is found)	(0) Does not state	
23. If samples are to be taken, describes the sampling process: for example, number of samples, amount, frequency, individual vs. composite, where samples are taken in the process, and percent considered passing	(1) States that samples should be taken (generally)	the Health Surveillance Agency carries out a collection of only one sample per batch for inspection, never the average of various samples from the same batch. The values obtained should always be within the range of ± 20% of the value indicated on the label, according to that indicated in the labeling standards (RDC Resolution No. 40 of 03/21/2001).
24. States applicability of using qualitative testing to determine the presence or absence of a vitamin or mineral (spot tests, iChecks)	(0) Does not state	
25. States registration is required in order to use a logo/be licensed to produce fortified foods	(2) Describes some type of registration or licensing	It is important to highlight that the iron-added wheat flours are exempt from the requirement for registration with the responsible agency from the Ministry of Health (Resolution No. 23 of March 15, 2000). Companies should report only the initiation of manufacturing or product importation to the State, Federal District or Municipal health au¬thorities, according to the template annexed to Resolution 23/2000 and Resolution 22/2000 or through the program for Products Exempted from the Requirement to Register - PRODIR, being able to initiate the commercialization of the product.
**COMMERCIAL MONITORING (conducted by government)**		
26. Provides justification for commercial monitoring at retail stores	(0) Does not provide justification for commercial monitoring	
27. Describes protocols and systems for commercial monitoring	(0) Does not describe	
28. If there are two or more government agencies involved in commercial monitoring, clarifies the roles and responsibilities between different government agencies in commercial monitoring	(0) Clarifies roles and responsibilities for no agencies	
29. Allows for monitoring to be conducted often enough that problems at the production site or import companies can be identified and addressed on a timely basis; specifies a timeline for inspections (i.e. once every 6 months) or works with production companies to correct noncompliance	(N/A) No commercial monitoring occurs	
30. If samples are to be taken, describes the sampling process: for example, number of samples, amount, frequency, individual vs. composite, where samples are taken in the process, and percent considered passing	(N/A) Does not describe the sampling process	
**IMPORT MONITORING (conducted by government)**		
31. Provides justification for import monitoring at points of entry	(0) Does not provide justification for import monitoring	
32. Describes protocols and systems for import monitoring	(0) Does not state	

33. If there are two or more government agencies involved in import monitoring, clarifies the roles and responsibilities between different government agencies in import monitoring	(N/A) Only one government agency involved	Art.5 – For the National Agency of Sanitary Surveillance (ANVISA)<br><br>I – to implement the national monitoring for the wheat and corn flours produced in every mill and industries, as well as products exposed in the market.<br>II – coordinate and regulate the hygiene control of the establishments that produce the flours and the exposed products in the market;<br>III – to analyze and publicize, in a systematic way, the control results and the follow up on the wheat and corn flours;<br>IV – to promote the inclusion of official labs in the control programs for analytical quality;<br>V – to promote awareness in the productive sector in a way to attend to the present health legislation;<br>VI – to monitor the imported wheat and corn flours to check if the comply with the present legislation, in particular for the amounts of iron and folic acid.
34. If samples are to be taken, describes the sampling process: for example, number of samples, amount, frequency, individual vs. composite, where samples are taken in the process, and percent considered passing	(N/A) Does not describe the sampling process	
**ENFORCEMENT/PENALTIES**		
35. Indicates roles and responsibilities in enforcing the legislation	(2) States the role and responsibilities of government in enforcement	Article 14. The penalties herein addressed shall be applied by the competent health surveillance authorities of the Ministry of Health, States, Federal District and Territories, pursuant to their respective duties established in the legislation or delegated thereto by agreements.
36. States incentives to start fortification	(2) States any incentives to encourage fortification initiation (e.g. tax incentives for new equipment or premix)	In Brazil, since this program is mandatory, the funding regarding the disclosure and c¬larifications to the consumers about the iron-added flours and the difference they make compared to previously commercialized flours should be borne by the government itself. The costs to establish a national iron fortification program will depend on factors such as the number and size of the mills, the adoption of the quality guarantee system, regulations on the subject and rigor in inspecting the foods as well as the level of iron to be added.<br>For the fortification of flours, the necessary capital is basically concentrated in three items: 1) the iron source, 2) the iron dispenser/feeder and 3) quality control analyses. A list of some of the national suppliers of iron sources (Annex VI), equipment (Annex VII) and laboratories that can perform the necessary analyses (Annex VIII) are presented in the annexes.
37. States incentives to continue fortification, including ensuring compliance	(0) Does not state	
38. States penalties to compel compliance	(2) States any penalties	Art. 3. Failure to fulfill the terms of this Resolution constitutes a health violation subject to the penalties set forth in Law No. 6437 of August 20, 1977 and other applicable provisions.
39. Penalties are objectively defined (e.g. first penalty=$100, second penalty=$300)	(2) Penalties are objectively laid out in the document (e.g. first penalty=$100, second penalty=$300)	Paragraph 1. The penalty of fine shall consist in payment of the following values: (Included by Provisional Measure no. 2,190-34, 2001)<br><br>I - for minor violations, from R$ 2.000,00 to R$ 75.000,00; (Included by provisional Measure no. 2,190-34, 2001)<br>II - for serious violations, from R$ 75.000,00 to R$ 200.000,00; (Included by provisional Measure no. 2,190-34, 2001)<br>III - for major violations, from R$ 200.000,00 to R$ 1.500.000,00. (Included by provisional Measure no. 2,190-34, 2001)<br>Paragraph 2. The fines herein addressed shall be applied in double amount in the case of recidivism. (Included by provisional Measure no. 2,190-34, 2001)

40. States that enforcement is required to include feedback and support to improve performance and correct noncompliance	(2) Requires any feedback/support to improve performance	Article 6. When imposing penalties and assessing their severity, health surveillance authorities shall take the following into account:<br>I - mitigating and aggravating circumstances;<br><br>II - the severity of violations in view of their consequences to public health;<br>III - criminal record of the offender with regard to sanitary regulations. Article 7. The following are mitigating circumstances:<br>I - the offender's act is not deemed essential to the course of events;<br>II - the sanitary legislation has been misunderstood, which is deemed excusable due to the offender's incapacity to admit the unlawfulness of the act;<br>III - the offender has spontaneously and immediately intended to repair or mitigate the consequences of his/her harmful act to public health;<br>IV - the offender has suffered possibly resistible coercion in order to commit the offense;<br>V - if primary offender and minor violation.
**LABORATORY**		
41. References required analytical assays for nutrients (e.g. Liquid chromatography-mass spectrometry for folic acid, atomic absorption for iron and zinc.)	(2) References required assays	The quantitative analysis can be performed using spectrophotometry or atomic absorption. The references for these two methods are shown below:<br><br>a) Method 944.02 by the AOAC. 16th edition, 1997.<br>b) Method 965.09 by the AOAC. 16th edition, 1995.
42. States recognition that laboratory results are subject to several sources of variation and do not provide conclusive evidence of compliance or noncompliance	(0) Does not state recognition	
43. Focuses on the quantitative analysis of "marker" micronutrients such as iron	(2) Focuses on quantitative analysis of marker micronutrient such as iron	The whole manual is about iron.
**REPORTING**		
44. States how government monitoring results are shared with stakeholders	(2) States how results are shared with stakeholders	III – to analyze and publicize, in a systematic way, the control results and the follow up on the wheat and corn flours (responsibility of ANVISA)

Burkina Faso Wheat

Element	Scoring (use drop-down menus)	Comments (open text)
**GENERAL**		
1. States that legislation applies to at least one food vehicle fit for human consumption (types/grades to be fortified)	(2) States at least one type fit for human consumption	Article 1(2) - wheat bread flour enriched with iron and folic acid.
2. States the public health objective; purpose and scope of legislation	(2) States the public health objective or general purpose of legislation	Given Law no. 23/ADP of 19 May 1994 codifying public health; Given that lack of iron constitute a public health problem in BF; Given the need to combat the difficulties caused by lack of iron and to promote health of the population
3. References latest available science or accepted international norms and recommendations, particularly for items that may not be covered in the country's documents	(2) States the documents referenced	
4. Provides definitions that includes terms that are specific to fortification (e.g. fortified food, premix, fortificant, food vehicle)	(0) Does not state	
5. Provides repeals (if there is at least one prior document about fortification)	(2) States repeals	Article 6: [this] abrogates all pre-existing contrary provisions
6. Provides effective date or gives grace period for when fortification is to begin (e.g. effective 6 months from signing)	(2) States effective date or grace period for when fortification is to begin (e.g. effective 6 months from signing)	Article 5: 6 months from date of publication to conform
**MICRONUTRIENTS/PREMIX**		
7. States nutrients required	(2) States nutrients	iron and folic acid
8. States fortificants (chemical compounds) to be used (including fortificants that are allowable as options)	(0) Does not state	
9. States fortification levels	(0) Does not state	
10. States consideration of bioavailability/biological activity of fortificants	(0) Does not state any consideration	
11. States consideration of nutrient stability	(0) Does not state any consideration	
**COSTING**		
12. States that the cost of fortification is regulated through cost sharing schemes (between government, industry, consumers) or tax measures [to assist industry]	(0) Does not state any consideration	
13. States consideration of the financial responsibility of monitoring and enforcing fortification (schedule of fees, budget) [on the government side]	(0) Does not state any consideration	
**LABELING**		
14. Includes some sort of statement/label/logo that makes it clear that the product is fortified	(0) Does not include statement, label, or logo	
15. Provides guidance on health claims that can be made for this product (specific to micronutrients added through fortification)	(0) Does not provide	
**INTERNAL MONITORING (conducted by industry)**		
16. If samples are to be taken, describes the sampling process: for example, number of samples, amount, frequency, individual vs. composite, where samples are taken in the process, and percent considered passing	(N/A) Does not describe the sampling process	
17. States that industry is required to follow quality assurance/quality control in regards to fortification	(0) Does not state requirement	
18. States applicability of using qualitative testing to determine the presence or absence of a vitamin or mineral (spot tests, iChecks)	(0) Does not state	
**EXTERNAL MONITORING (conducted by government)**		
19.States requirement for external monitoring at the production site to assure compliance with standards and regulations	(2) States requirement for external monitoring or the need for audits/inspections	Article 2: Quality control is ensured throughout distribution chain by trained public service agents and/or additional individuals appointed for that purpose.
20. Describes protocols and systems for regulatory monitoring	(1) Does not explicitly describe, but references protocols and systems for regulatory monitoring	
21. If there are two or more government agencies involved in external monitoring, clarifies the roles and responsibilities between different government agencies in external monitoring	(0) Clarifies roles and responsibilities for no agencies	Article 7: Ministries of Health, Agribulture and Hydraulics, Economy and Finance, Industry/Commerce/Artisans are all charged to apply the order as relevant. All 4 Ministers signed on.
22. Allows for monitoring to be conducted often enough that problems can be identified and addressed on a timely basis; specifies a timeline for inspections (i.e. once every 6 months, increasing to once every 2 months if a discrepancy is found)	(0) Does not state	
23. If samples are to be taken, describes the sampling process: for example, number of samples, amount, frequency, individual vs. composite, where samples are taken in the process, and percent considered passing	(N/A) Does not describe the sampling process	
24. States applicability of using qualitative testing to determine the presence or absence of a vitamin or mineral (spot tests, iChecks)	(0) Does not state	
25. States registration is required in order to use a logo/be licensed to produce fortified foods	(0) Does not state that registration or licensing is required	

**COMMERCIAL MONITORING (conducted by government)**		
26. Provides justification for commercial monitoring at retail stores	(0) Does not provide justification for commercial monitoring	
27. Describes protocols and systems for commercial monitoring	(0) Does not describe	
28. If there are two or more government agencies involved in commercial monitoring, clarifies the roles and responsibilities between different government agencies in commercial monitoring	(0) Clarifies roles and responsibilities for no agencies	
29. Allows for monitoring to be conducted often enough that problems at the production site or import companies can be identified and addressed on a timely basis; specifies a timeline for inspections (i.e. once every 6 months) or works with production companies to correct noncompliance	(0) Does not state	
30. If samples are to be taken, describes the sampling process: for example, number of samples, amount, frequency, individual vs. composite, where samples are taken in the process, and percent considered passing	(N/A) Does not describe the sampling process	
**IMPORT MONITORING (conducted by government)**		
31. Provides justification for import monitoring at points of entry	(0) Does not provide justification for import monitoring	
32. Describes protocols and systems for import monitoring	(0) Does not state	
33. If there are two or more government agencies involved in import monitoring, clarifies the roles and responsibilities between different government agencies in import monitoring	(0) Clarifies roles and responsibilities for no agencies	
34. If samples are to be taken, describes the sampling process: for example, number of samples, amount, frequency, individual vs. composite, where samples are taken in the process, and percent considered passing	(N/A) Does not describe the sampling process	
**ENFORCEMENT/PENALTIES**		
35. Indicates roles and responsibilities in enforcing the legislation	(2) States the role and responsibilities of government in enforcement	Article 4: Minister of Commerce can…
36. States incentives to start fortification	(0) Does not state	
37. States incentives to continue fortification, including ensuring compliance	(0) Does not state	
38. States penalties to compel compliance	(2) States any penalties	Article 3: Failure to observe Article 1 constitutes an infraction punishable by administrative and criminal sanctions; Article 4: Minister of Commerce can take the following administrative measures…
39. Penalties are objectively defined (e.g. first penalty=$100, second penalty=$300)	(0) Penalties are not objectively laid out	
40. States that enforcement is required to include feedback and support to improve performance and correct noncompliance	(0) Does not require	
**LABORATORY**		
41. References required analytical assays for nutrients (e.g. Liquid chromatography-mass spectrometry for folic acid, atomic absorption for iron and zinc.)	(0) Does not state requirements	
42. States recognition that laboratory results are subject to several sources of variation and do not provide conclusive evidence of compliance or noncompliance	(0) Does not state recognition	
43. Focuses on the quantitative analysis of "marker" micronutrients such as iron	(0) Does not state	
**REPORTING**		
44. States how government monitoring results are shared with stakeholders	(0) Does not state how results are shared	

Cameroon Wheat

Element	Scoring (use drop-down menus)	Comments (open text)
**GENERAL**		
1. States that legislation applies to at least one food vehicle fit for human consumption (types/grades to be fortified)	(2) States at least one type fit for human consumption	MAKING APPLICATION OF THE STANDARD ON WHEAT FLOUR FORTIFIED WITH IRON, FOLIC ACID, VITAMIN AND ZINC B12 MANDATORY. (1) The product referred to in Article 1 above and intended for human consumption is subject to a certification procedure prior to its consumption.
2. States the public health objective; purpose and scope of legislation	(0) Does not state	
3. References latest available science or accepted international norms and recommendations, particularly for items that may not be covered in the country's documents	(2) States the documents referenced	The following reference documents are indispensable for the application of this<br>standard. For dated references, only the cited edition applies. For undated references,<br>the latest edition of the reference document applies (including possible amendments).<br>- CAC / RCP 1-1969, Rev.4.2003: International Code of Practice recommends -General Principles<br>in matters of food hygiene; and its national equivalence, NC 30: 2001-20;<br>- CAC / GL21-1997: Principles governing the establishment and implementation of the microbiology criteria<br>for food;<br>- CAC / GL9 -1987, Amend.2. (1989 and 1991): General principles governing the addition<br>of nutrients to foods;<br>- CODEX STAN 1 -1985, Amend.7.2010: General standard for labeling of<br>prepacked food; and its national equivalence, NC 04: 2000-20 - Labeling of<br>Prepackaged Foods in Cameroon;<br>- CODEX STAN 192-1995, Rev.11.2010- General Standard Codex for food additives<br>; and its national equivalence NC 31: 2001-20 - Standard for food additives;<br>– CODEX STAN 193-1995, Rev.4.2009, Amend.2.2010: General Standard for<br>contaminants and toxins present in products intended for
4. Provides definitions that includes terms that are specific to fortification (e.g. fortified food, premix, fortificant, food vehicle)	(2) States at least one term related to fortification	3.1 Product Definition<br>Wheat flour enriched with iron, folic acid, zinc and vitamin B12 is the product made<br>from regular wheat grains, Triticum aestivum L. or branched wheat, Triticum compactum Host., or<br>all mixtures of the latter, by milling or grinding processes in which the bran and the<br>germ are partially eliminated and the rest reduced to sufficiently fine powder, to which a<br>specified amount of iron, folic acid, vitamin B12 and zinc is added.<br>3.2.5 The **enrichment** is adding a foodstuff or several nutrients which are essential or not normally contained in the food, has the effect of preventing or correcting a deficiency in one or more nutrients in the population or of specific population groups. 3.2.7 An essential nutrient is any substance normally consumed as<br>a constituent of food, is necessary for the growth, development and maintenance of good<br>health and can not be synthesized in sufficient quantities by the body
5. Provides repeals (if there is at least one prior document about fortification)	(N/A) No prior documents about fortification	
6. Provides effective date or gives grace period for when fortification is to begin (e.g. effective 6 months from signing)	(2) States effective date or grace period for when fortification is to begin (e.g. effective 6 months from signing)	From the date of signing this Order, standard NC 01:2000-03, Ver. 1 (2011) on wheat flour fortified with iron, folic acid, zinc and vitamin B12 will be mandatory. The importers and local producers concerned shall have a period of four (4) months from the date of signing of this Order, to sell their stocks of flour.
**MICRONUTRIENTS/PREMIX**		
7. States nutrients required	(2) States nutrients	Tolerance<br>Iron (fumarate) 60} 10%<br>Folic acid 5.0} 10%<br>Zinc (Zinc oxide) 95} 10%<br>Vitamin B12 (Cyanocobalamin) 0.04} 10%

8. States fortificants (chemical compounds) to be used (including fortificants that are allowable as options)	(2) States fortificants for at least one nutrient	Tolerance<br>Iron (fumarate) 60} 10%<br>Folic acid 5.0} 10%<br>Zinc (Zinc oxide) 95} 10%<br>Vitamin B12 (Cyanocobalamin) 0.04} 10%
9. States fortification levels	(2) States a range or number with +/-	Tolerance<br>Iron (fumarate) 60} 10%<br>Folic acid 5.0} 10%<br>Zinc (Zinc oxide) 95} 10%<br>Vitamin B12 (Cyanocobalamin) 0.04} 10%
10. States consideration of bioavailability/biological activity of fortificants	(0) Does not state any consideration	
11. States consideration of nutrient stability	(2) States some consideration of bioavailability (mentions these or related terms)	9.3 Product storage conditions should appear on the label in order to better<br>keep sensitive ingredients.
**COSTING**		
12. States that the cost of fortification is regulated through cost sharing schemes (between government, industry, consumers) or tax measures [to assist industry]	(0) Does not state any consideration	
13. States consideration of the financial responsibility of monitoring and enforcing fortification (schedule of fees, budget) [on the government side]	(0) Does not state any consideration	
**LABELING**		
14. Includes some sort of statement/label/logo that makes it clear that the product is fortified	(2) Includes a statement, label, or logo	Without prejudice to the specifications in the standard on labelling of pre-packaged foods and laws and regulations on the matter, the affixing of the fortification logo on the product label subject to the standard referred to in Article 1 of this Order is mandatory.<br>9.4 The identification logo for the enriched wheat flour with iron, folic acid, zinc and vitamin B12 must be clearly marked and in a visible manner on the packaging.<br>9.1 The name of the product declared on the label must be "Enriched wheat with iron, folic acid,<br>zinc and vitamin B12".
15. Provides guidance on health claims that can be made for this product (specific to micronutrients added through fortification)	(0) Does not provide	
**INTERNAL MONITORING (conducted by industry)**		
16. If samples are to be taken, describes the sampling process: for example, number of samples, amount, frequency, individual vs. composite, where samples are taken in the process, and percent considered passing	(N/A) Does not describe the sampling process	
17. States that industry is required to follow quality assurance/quality control in regards to fortification	(0) Does not state requirement	
18. States applicability of using qualitative testing to determine the presence or absence of a vitamin or mineral (spot tests, iChecks)	(0) Does not state	
**EXTERNAL MONITORING (conducted by government)**		
19.States requirement for external monitoring at the production site to assure compliance with standards and regulations	(0) Does not state requirement	(1) The product referred to in Article 1 above and intended for human consumption is subject to a certification procedure prior to its consumption.<br>(2) The certification referred to in paragraph (1) above results in the issuance of a certificate of conformity.<br>(3) The certificate of conformity is issued by the Agency for Standards and Quality (ANOR). (1) Importers and local producers are required to have a certificate of conformity before any distribution and release for consumption of their product in the national territory.<br>(2) The certificate of conformity is presented at the request of the competent public services, particularly during customs import operations.
20. Describes protocols and systems for regulatory monitoring	(0) Does not describe	
21. If there are two or more government agencies involved in external monitoring, clarifies the roles and responsibilities between different government agencies in external monitoring	(0) Clarifies roles and responsibilities for no agencies	
22. Allows for monitoring to be conducted often enough that problems can be identified and addressed on a timely basis; specifies a timeline for inspections (i.e. once every 6 months, increasing to once every 2 months if a discrepancy is found)	(0) Does not state	
23. If samples are to be taken, describes the sampling process: for example, number of samples, amount, frequency, individual vs. composite, where samples are taken in the process, and percent considered passing	(N/A) Does not describe the sampling process	

24. States applicability of using qualitative testing to determine the presence or absence of a vitamin or mineral (spot tests, iChecks)	(0) Does not state	
25. States registration is required in order to use a logo/be licensed to produce fortified foods	(2) Describes some type of registration or licensing	(1) The product referred to in Article 1 above and intended for human consumption is subject to a certification procedure prior to its consumption.<br>(2) The certification referred to in paragraph (1) above results in the issuance of a certificate of conformity.<br>(3) The certificate of conformity is issued by the Agency for Standards and Quality (ANOR). (1) Importers and local producers are required to have a certificate of conformity before any distribution and release for consumption of their product in the national territory.<br>(2) The certificate of conformity is presented at the request of the competent public services, particularly during customs import operations.
**COMMERCIAL MONITORING (conducted by government)**		
26. Provides justification for commercial monitoring at retail stores	(0) Does not provide justification for commercial monitoring	
27. Describes protocols and systems for commercial monitoring	(0) Does not describe	
28. If there are two or more government agencies involved in commercial monitoring, clarifies the roles and responsibilities between different government agencies in commercial monitoring	(0) Clarifies roles and responsibilities for no agencies	
29. Allows for monitoring to be conducted often enough that problems at the production site or import companies can be identified and addressed on a timely basis; specifies a timeline for inspections (i.e. once every 6 months) or works with production companies to correct noncompliance	(N/A) No commercial monitoring occurs	
30. If samples are to be taken, describes the sampling process: for example, number of samples, amount, frequency, individual vs. composite, where samples are taken in the process, and percent considered passing	(N/A) Does not describe the sampling process	
**IMPORT MONITORING (conducted by government)**		
31. Provides justification for import monitoring at points of entry	(0) Does not provide justification for import monitoring	(1) Importers and local producers are required to have a certificate of conformity before any distribution and release for consumption of their product in the national territory.<br>(2) The certificate of conformity is presented at the request of the competent public services, particularly during customs import operations.
32. Describes protocols and systems for import monitoring	(0) Does not state	
33. If there are two or more government agencies involved in import monitoring, clarifies the roles and responsibilities between different government agencies in import monitoring	(0) Clarifies roles and responsibilities for no agencies	
34. If samples are to be taken, describes the sampling process: for example, number of samples, amount, frequency, individual vs. composite, where samples are taken in the process, and percent considered passing	(N/A) Does not describe the sampling process	
**ENFORCEMENT/PENALTIES**		
35. Indicates roles and responsibilities in enforcing the legislation	(0) Does not state	The relevant departments of the Ministry responsible for public health, the Ministry responsible for standardisation and the Ministry for Trade are each individually responsible with regard to the implementation of this Order, which will be announced and published in every place so required
36. States incentives to start fortification	(0) Does not state	
37. States incentives to continue fortification, including ensuring compliance	(0) Does not state	
38. States penalties to compel compliance	(0) Does not state	
39. Penalties are objectively defined (e.g. first penalty=$100, second penalty=$300)	(N/A) No penalties are stated (Answered (0) to previous question)	
40. States that enforcement is required to include feedback and support to improve performance and correct noncompliance	(0) Does not require	
**LABORATORY**		

41. References required analytical assays for nutrients (e.g. Liquid chromatography-mass spectrometry for folic acid, atomic absorption for iron and zinc.)	(2) References required assays	10.3 Evaluation of the content of essential nutrients<br>Table 8: Method for determination of the essential nutrients of wheat flour<br>enriched with iron, folic acid, zinc and vitamin B12.<br>Essential Nutrients Analysis Methods<br>High performance liquid chromatography (HPLC) iron (Fumarate).<br>High performance liquid chromatography folic acid (HPLC).<br>High performance liquid chromatography zinc (zinc oxide) (HPLC).<br>High performance liquid chromatography Vitamin B12 (Cyanocobalamin) (HPLC).
42. States recognition that laboratory results are subject to several sources of variation and do not provide conclusive evidence of compliance or noncompliance	(0) Does not state recognition	
43. Focuses on the quantitative analysis of "marker" micronutrients such as iron	(0) Does not state	
**REPORTING**		
44. States how government monitoring results are shared with stakeholders	(0) Does not state how results are shared	

Canada Wheat

Element	Scoring (use drop-down menus)	Comments (open text)
**GENERAL**		
1. States that legislation applies to at least one food vehicle fit for human consumption (types/grades to be fortified)	(2) States at least one type fit for human consumption	Flour, White Flour, Enriched Flour or Enriched White Flour
2. States the public health objective; purpose and scope of legislation	(0) Does not state	
3. References latest available science or accepted international norms and recommendations, particularly for items that may not be covered in the country's documents	(2) States the documents referenced	b) where no specifications are set out in this Part for that additive but specifications are set out for it in the Food Chemicals Codex, Fourth Edition, 1996, published by the National Academy of Sciences, Washington, D.C., United States, as amended from time to time, meet those specifications;
4. Provides definitions that includes terms that are specific to fortification (e.g. fortified food, premix, fortificant, food vehicle)	(2) States at least one term related to fortification	A.01.010 lists definitions such as acceptable method, lot number, manufacturer or distributor. B.01.001 defines agricultural chemical (which includes vitamin, mineral nutrient or amino acid), common name, component, daily value, durable life, durable life date, energy value, food additive (which excludes vitamins, mineral nutrients and aminoc acids, other than those listed in the tables in Division 16), ingredient, nutrition facts table, overage, parts per million or ppm, principal display panel, reasonable daily intake, recommended daily intake, reference amount, reference standard, weighted recommended nutrient intake. Section B.01.010.1 defines food allergen, gluten. D.01.001 defines brand name, common name, prepackaged product, and others defined in previous sections.
5. Provides repeals (if there is at least one prior document about fortification)	(2) States repeals	[Repealed, SOR/94-227, s. 1] This consolidation is current to September 29, 2014. The last amendments came into force on August 21, 2014. Any amendments that were not in force as of September 29, 2014 are set out at the end of this document under the heading “Amendments Not in Force”.
6. Provides effective date or gives grace period for when fortification is to begin (e.g. effective 6 months from signing)	(2) States effective date or grace period for when fortification is to begin (e.g. effective 6 months from signing)	Page 2: "This consolidation is current to September 29, 2014. The last amendments came into force on August 21, 2014. Any amendments that were not in force as of September 29, 2014 are set out at the end of this document under the heading “Amendments Not in Force”."
**MICRONUTRIENTS/PREMIX**		
7. States nutrients required	(2) States nutrients	shall contain in 100 grams of flour: 0.64 milligram of thiamine, 0.40 milligram of riboflavin, 5.30 milligrams of niacin or niacinamide, 0.15 milligram of folic acid, and 4.4 milligrams of iron;
8. States fortificants (chemical compounds) to be used (including fortificants that are allowable as options)	(2) States fortificants for at least one nutrient	shall contain in 100 grams of flour: 0.64 milligram of thiamine, 0.40 milligram of riboflavin, 5.30 milligrams of niacin or niacinamide, 0.15 milligram of folic acid, and 4.4 milligrams of iron;
9. States fortification levels	(1) States one number only	No acceptable range, just values. Gordon says "While not reflected in B.13.001 Health Canada has established as a result of a research initiative conducted by the Canadian National Millers Association, a tolerance of 80 to 175% of stipulated minimum enrichment levels, reflecting what is practically achievable under usual mill operating conditions"
10. States consideration of bioavailability/biological activity of fortificants	(2) States some consideration of bioavailability (mentions these or related terms)	
11. States consideration of nutrient stability	(0) Does not state any consideration	
**COSTING**		
12. States that the cost of fortification is regulated through cost sharing schemes (between government, industry, consumers) or tax measures [to assist industry]	(0) Does not state any consideration	
13. States consideration of the financial responsibility of monitoring and enforcing fortification (schedule of fees, budget) [on the government side]	(0) Does not state any consideration	
**LABELING**		
14. Includes some sort of statement/label/logo that makes it clear that the product is fortified	(0) Does not include statement, label, or logo	

15. Provides guidance on health claims that can be made for this product (specific to micronutrients added through fortification)	(0) Does not provide	(1) No person shall, on the label of or in any advertisement for a food, other than in the nutrition facts table, if any, include a declaration of the food's energy value or the amount of a nutrient contained in the food unless it is declared in the following manner, per serving of stated size:(b) in the case of a vitamin set out in column I of Table I to Division 1 of Part D or a mineral nutrient set out in column I of Table I to Division 2 of Part D, in the units specified in that column;
**INTERNAL MONITORING (conducted by industry)**		
16. If samples are to be taken, describes the sampling process: for example, number of samples, amount, frequency, individual vs. composite, where samples are taken in the process, and percent considered passing	(N/A) Does not describe the sampling process	
17. States that industry is required to follow quality assurance/quality control in regards to fortification	(0) Does not state requirement	
18. States applicability of using qualitative testing to determine the presence or absence of a vitamin or mineral (spot tests, iChecks)	(0) Does not state	
**EXTERNAL MONITORING (conducted by government)**		
19.States requirement for external monitoring at the production site to assure compliance with standards and regulations	(0) Does not state requirement	
20. Describes protocols and systems for regulatory monitoring	(0) Does not describe	
21. If there are two or more government agencies involved in external monitoring, clarifies the roles and responsibilities between different government agencies in external monitoring	(1) Clarifies roles and responsibilities for one agency	"A.01.022. An inspector shall perform the functions and duties and carry out the responsibilities in respect of foods and drugs prescribed by the Act, and these Regulations. A.01.023. The authority of an inspector extends to and includes the whole of Canada." "A.01.026. An inspector may, for the proper administration of the Act or these Regulations, take photographs of (a) any article that is referred to in subsection 23(2) of the Act; (b) any place where, on reasonable grounds, he believes any article referred to in paragraph (a) is manufactured, prepared, preserved, packaged or stored; and (c) anything that, on reasonable grounds, he believes is used or capable of being used for the manufacture, preparation, preservation, packaging or storing of any article referred to in paragraph (a)." SAMPLING "A.01.050. When taking a sample of an article pursuant to paragraph 23(1)(a) of the Act, an inspector shall
22. Allows for monitoring to be conducted often enough that problems can be identified and addressed on a timely basis; specifies a timeline for inspections (i.e. once every 6 months, increasing to once every 2 months if a discrepancy is found)	(0) Does not state	

23. If samples are to be taken, describes the sampling process: for example, number of samples, amount, frequency, individual vs. composite, where samples are taken in the process, and percent considered passing	(1) States that samples should be taken (generally)	SAMPLING<br>A.01.050. When taking a sample of an article pursuant to paragraph 23(1)(a) of the Act, an inspector shall inform the owner thereof or the person from whom the sample is being obtained of the inspector’s intention to submit the sample or a part thereof to an analyst for analysis or examination, and<br>(a) where, in the opinion of the inspector, division of the procured quantity would not interfere with analysis or examination<br>(i) divide the quantity into three parts,<br>(ii) identify the three parts as the owner’s portion, the sample, and the duplicate sample and where only one part bears the label, that part shall be identified as the sample,<br>(iii) seal each part in such a manner that it cannot be opened without breaking the seal, and<br>(iv) deliver the part identified as the owner’s portion to the owner or the person from whom the sample was obtained and forward the sample and the duplicate sample to an analyst for analysis or examination;<br>or<br>(b) where, in the opinion of the inspector, division of the procured quantity would interfere with analysis or examination<br>(i) identify the entire quantity as the sample,<br>(ii) seal the sample in such a manner that it cannot be opened without breaking the seal, and<br>(iii) forward the sample to an analyst for analysis or examination.<br>A.01.051. Where the owner or the person from whom the
24. States applicability of using qualitative testing to determine the presence or absence of a vitamin or mineral (spot tests, iChecks)	(0) Does not state	
25. States registration is required in order to use a logo/be licensed to produce fortified foods	(0) Does not state that registration or licensing is required	
**COMMERCIAL MONITORING (conducted by government)**		
26. Provides justification for commercial monitoring at retail stores	(0) Does not provide justification for commercial monitoring	
27. Describes protocols and systems for commercial monitoring	(0) Does not describe	
28. If there are two or more government agencies involved in commercial monitoring, clarifies the roles and responsibilities between different government agencies in commercial monitoring	(0) Clarifies roles and responsibilities for no agencies	
29. Allows for monitoring to be conducted often enough that problems at the production site or import companies can be identified and addressed on a timely basis; specifies a timeline for inspections (i.e. once every 6 months) or works with production companies to correct noncompliance	(N/A) No commercial monitoring occurs	
30. If samples are to be taken, describes the sampling process: for example, number of samples, amount, frequency, individual vs. composite, where samples are taken in the process, and percent considered passing	(N/A) Does not describe the sampling process	
**IMPORT MONITORING (conducted by government)**		
31. Provides justification for import monitoring at points of entry	(0) Does not provide justification for import monitoring	
32. Describes protocols and systems for import monitoring	(0) Does not state	
33. If there are two or more government agencies involved in import monitoring, clarifies the roles and responsibilities between different government agencies in import monitoring	(2) Clarifies roles and responsibilities for more than one agency	"A.01.043. Where an inspector, upon examination of a food or drug or sample thereof or on receipt of a report of an analyst of the result of an analysis or examination of the food or drug or sample, is of the opinion that the sale of the food or drug in Canada would constitute a violation of the Act or these Regulations, the inspector shall so notify in writing the collector of customs concerned and the importer."

34. If samples are to be taken, describes the sampling process: for example, number of samples, amount, frequency, individual vs. composite, where samples are taken in the process, and percent considered passing	(1) States that samples should be taken (generally)	SAMPLING<br>A.01.050. When taking a sample of an article pursuant to paragraph 23(1)(a) of the Act, an inspector shall inform the owner thereof or the person from whom the sample is being obtained of the inspector's intention to submit the sample or a part thereof to an analyst for analysis or examination, and<br>(a) where, in the opinion of the inspector, division of the procured quantity would not interfere with analysis or examination<br>(i) divide the quantity into three parts,<br>(ii) identify the three parts as the owner's portion, the sample, and the duplicate sample and where only one part bears the label, that part shall be identified as the sample,<br>(iii) seal each part in such a manner that it cannot be opened without breaking the seal, and<br>(iv) deliver the part identified as the owner's portion to the owner or the person from whom the sample was obtained and forward the sample and the duplicate sample to an analyst for analysis or examination;<br>or<br>(b) where, in the opinion of the inspector, division of the procured quantity would interfere with analysis or examination<br>(i) identify the entire quantity as the sample,<br>(ii) seal the sample in such a manner that it cannot be opened without breaking the seal, and<br>(iii) forward the sample to an analyst for analysis or examination.<br>A.01.051. Where the owner or the person from whom the
**ENFORCEMENT/PENALTIES**		
35. Indicates roles and responsibilities in enforcing the legislation	(2) States the role and responsibilities of government in enforcement	"A.01.022. An inspector shall perform the functions and duties and carry out the responsibilities in respect of foods and drugs prescribed by the Act, and these Regulations.<br>A.01.023. The authority of an inspector extends to and includes the whole of Canada."<br>"A.01.026. An inspector may, for the proper administration of the Act or these Regulations, take photographs of<br>(a) any article that is referred to in subsection 23(2) of the Act;<br>(b) any place where, on reasonable grounds, he believes any article referred to in paragraph (a) is manufactured, prepared, preserved, packaged or stored; and
36. States incentives to start fortification	(0) Does not state	
37. States incentives to continue fortification, including ensuring compliance	(0) Does not state	
38. States penalties to compel compliance	(0) Does not state	
39. Penalties are objectively defined (e.g. first penalty=$100, second penalty=$300)	(N/A) No penalties are stated (Answered (0) to previous question)	
40. States that enforcement is required to include feedback and support to improve performance and correct noncompliance	(0) Does not require	
**LABORATORY**		
41. References required analytical assays for nutrients (e.g. Liquid chromatography-mass spectrometry for folic acid, atomic absorption for iron and zinc.)	(0) Does not state requirements	
42. States recognition that laboratory results are subject to several sources of variation and do not provide conclusive evidence of compliance or noncompliance	(0) Does not state recognition	
43. Focuses on the quantitative analysis of "marker" micronutrients such as iron	(0) Does not state	
**REPORTING**		
44. States how government monitoring results are shared with stakeholders	(0) Does not state how results are shared	

Cape Verde Wheat

Element	Scoring (use drop-down menus)	Comments (open text)
**GENERAL**		
1. States that legislation applies to at least one food vehicle fit for human consumption (types/grades to be fortified)	(2) States at least one type fit for human consumption	Article 2(1): Wheat flours type 45, 55, 65<br>Article 2(4): Excluding type 80, 110, and 150 for technical processing limitations and low consumption
2. States the public health objective; purpose and scope of legislation	(2) States the public health objective or general purpose of legislation	Provides WHO data, effects of lack of iron, states that iron deficiency anemia is a major nutritional problem with an impact on public health. Provides data on prevalence in Cape Verde. Gives consumption data for wheat flour. Given this, it is necessary to impose mandatory fortification.
3. References latest available science or accepted international norms and recommendations, particularly for items that may not be covered in the country's documents	(2) States the documents referenced	Article 1: Establishes procedure for fortification using technical standards and procedures of the CODEX for the addition of these nutrients.
4. Provides definitions that includes terms that are specific to fortification (e.g. fortified food, premix, fortificant, food vehicle)	(2) States at least one term related to fortification	Article 3: wheat flour, fortified food, folic acid, iron, bioavailability, nutrient, essential nutrient all defined.
5. Provides repeals (if there is at least one prior document about fortification)	(2) States repeals	Article 19 - repeals
6. Provides effective date or gives grace period for when fortification is to begin (e.g. effective 6 months from signing)	(2) States effective date or grace period for when fortification is to begin (e.g. effective 6 months from signing)	Article 17 - 6 months grace period
**MICRONUTRIENTS/PREMIX**		
7. States nutrients required	(2) States nutrients	iron and folic acid
8. States fortificants (chemical compounds) to be used (including fortificants that are allowable as options)	(2) States fortificants for at least one nutrient	ferrous sulphate or sodium EDTA and iron NaFeEDTA
9. States fortification levels	(1) States one number only	max and mins as per ISO and CODEX - table in Annex
10. States consideration of bioavailability/biological activity of fortificants	(2) States some consideration of bioavailability (mentions these or related terms)	Compounds must be food-grade and bioavailable, must be imported from certified distributors
11. States consideration of nutrient stability	(2) States consideration of nutrient stability	Choice of compounds should ensure the stability of nutrients
**COSTING**		
12. States that the cost of fortification is regulated through cost sharing schemes (between government, industry, consumers) or tax measures [to assist industry]	(0) Does not state any consideration	
13. States consideration of the financial responsibility of monitoring and enforcing fortification (schedule of fees, budget) [on the government side]	(0) Does not state any consideration	
**LABELING**		
14. Includes some sort of statement/label/logo that makes it clear that the product is fortified	(2) Includes a statement, label, or logo	Article 6 - labelling and logo rules
15. Provides guidance on health claims that can be made for this product (specific to micronutrients added through fortification)	(2) Provides guidance on health claims specific to micronutrients added through fortification	need to establish rules on marketing of fortified wheat flour
**INTERNAL MONITORING (conducted by industry)**		
16. If samples are to be taken, describes the sampling process: for example, number of samples, amount, frequency, individual vs. composite, where samples are taken in the process, and percent considered passing	(N/A) Does not describe the sampling process	
17. States that industry is required to follow quality assurance/quality control in regards to fortification	(2) States requirement of QA/QC for fortification	Article 7(4) - economic operators should have the specialized technique to monitor and carry out the procecss. Article 9 - QC process and Article 10 - QC Certification
18. States applicability of using qualitative testing to determine the presence or absence of a vitamin or mineral (spot tests, iChecks)	(0) Does not state	
**EXTERNAL MONITORING (conducted by government)**		
19.States requirement for external monitoring at the production site to assure compliance with standards and regulations	(2) States requirement for external monitoring or the need for audits/inspections	need to establish rules on the supervision of production.
20. Describes protocols and systems for regulatory monitoring	(1) Does not explicitly describe, but references protocols and systems for regulatory monitoring	mentions inspections
21. If there are two or more government agencies involved in external monitoring, clarifies the roles and responsibilities between different government agencies in external monitoring	(2) Clarifies roles and responsibilities for more than one agency	Article 8 - MoH responsible for fortification policy, MoI responsible for managing production and marketing policy. Government responsibility for authorization and supervision of imports. Independent body responsible for regulating food is responsible for licensing, management of customs, and control of economic fraud via inspections.
22. Allows for monitoring to be conducted often enough that problems can be identified and addressed on a timely basis; specifies a timeline for inspections (i.e. once every 6 months, increasing to once every 2 months if a discrepancy is found)	(0) Does not state	

23. If samples are to be taken, describes the sampling process: for example, number of samples, amount, frequency, individual vs. composite, where samples are taken in the process, and percent considered passing	(N/A) Does not describe the sampling process	
24. States applicability of using qualitative testing to determine the presence or absence of a vitamin or mineral (spot tests, iChecks)	(0) Does not state	
25. States registration is required in order to use a logo/be licensed to produce fortified foods	(2) Describes some type of registration or licensing	Article 8: licensing of business production units.
**COMMERCIAL MONITORING (conducted by government)**		
26. Provides justification for commercial monitoring at retail stores	(0) Does not provide justification for commercial monitoring	Article 10 (1/2) - responsibility of the trader to guarantee quality during period of validity (expiry date) and presenting it to relevent authorities if requested.
27. Describes protocols and systems for commercial monitoring	(0) Does not describe	
28. If there are two or more government agencies involved in commercial monitoring, clarifies the roles and responsibilities between different government agencies in commercial monitoring	(0) Clarifies roles and responsibilities for no agencies	
29. Allows for monitoring to be conducted often enough that problems at the production site or import companies can be identified and addressed on a timely basis; specifies a timeline for inspections (i.e. once every 6 months) or works with production companies to correct noncompliance	(0) Does not state	
30. If samples are to be taken, describes the sampling process: for example, number of samples, amount, frequency, individual vs. composite, where samples are taken in the process, and percent considered passing	(N/A) Does not describe the sampling process	
**IMPORT MONITORING (conducted by government)**		
31. Provides justification for import monitoring at points of entry	(2) Provides justification for import monitoring	need to establish rules on importing and exporting of fortified wheat flour
32. Describes protocols and systems for import monitoring	(0) Does not state	
33. If there are two or more government agencies involved in import monitoring, clarifies the roles and responsibilities between different government agencies in import monitoring	(2) Clarifies roles and responsibilities for more than one agency	See # 21
34. If samples are to be taken, describes the sampling process: for example, number of samples, amount, frequency, individual vs. composite, where samples are taken in the process, and percent considered passing	(N/A) Does not describe the sampling process	
**ENFORCEMENT/PENALTIES**		
35. Indicates roles and responsibilities in enforcing the legislation	(2) States the role and responsibilities of government in enforcement	Article 11(1) - independent entiry responsible for regulation of food products and control of economic fraud. Any other govt agency can be asked to collaborate as necessary
36. States incentives to start fortification	(0) Does not state	
37. States incentives to continue fortification, including ensuring compliance	(0) Does not state	
38. States penalties to compel compliance	(2) States any penalties	Article 11(4) - seizure of product, closure of establishment, destruction of product in the event of public health risk
39. Penalties are objectively defined (e.g. first penalty=$100, second penalty=$300)	(2) Penalties are objectively laid out in the document (e.g. first penalty=$100, second penalty=$300)	Article 12 - fees, escalating based on number of offenses
40. States that enforcement is required to include feedback and support to improve performance and correct noncompliance	(0) Does not require	
**LABORATORY**		
41. References required analytical assays for nutrients (e.g. Liquid chromatography-mass spectrometry for folic acid, atomic absorption for iron and zinc.)	(0) Does not state requirements	
42. States recognition that laboratory results are subject to several sources of variation and do not provide conclusive evidence of compliance or noncompliance	(0) Does not state recognition	
43. Focuses on the quantitative analysis of "marker" micronutrients such as iron	(0) Does not state	
**REPORTING**		
44. States how government monitoring results are shared with stakeholders	(0) Does not state how results are shared	

CARICOM Wheat

Element	Scoring (use drop-down menus)	Comments (open text)
**GENERAL**		
1. States that legislation applies to at least one food vehicle fit for human consumption (types/grades to be fortified)	(2) States at least one type fit for human consumption	1.1 - wheat flour for human consumption from common or club wheat; 1.2 - exceptions listed
2. States the public health objective; purpose and scope of legislation	(2) States the public health objective or general purpose of legislation	
3. References latest available science or accepted international norms and recommendations, particularly for items that may not be covered in the country's documents	(2) States the documents referenced	0.4 - Guidance from the Codex, regulations from Canada/Trinidad and Tobago
4. Provides definitions that includes terms that are specific to fortification (e.g. fortified food, premix, fortificant, food vehicle)	(2) States at least one term related to fortification	2.2 - Enriched wheat flour; 2.3 - food additive
5. Provides repeals (if there is at least one prior document about fortification)	(0) Does not state	
6. Provides effective date or gives grace period for when fortification is to begin (e.g. effective 6 months from signing)	(0) Does not state	
**MICRONUTRIENTS/PREMIX**		
7. States nutrients required	(2) States nutrients	4.2 - thiamine, riboflavin, niacin, iron, calcium (optional)
8. States fortificants (chemical compounds) to be used (including fortificants that are allowable as options)	(0) Does not state	
9. States fortification levels	(2) States a range or number with +/-	Table 2 provides a min/max
10. States consideration of bioavailability/biological activity of fortificants	(0) Does not state any consideration	
11. States consideration of nutrient stability	(2) States consideration of nutrient stability	8.1 - packaged to safeguard the hygienic, nutritional, and technological characteristics of the flour
**COSTING**		
12. States that the cost of fortification is regulated through cost sharing schemes (between government, industry, consumers) or tax measures [to assist industry]	(0) Does not state any consideration	
13. States consideration of the financial responsibility of monitoring and enforcing fortification (schedule of fees, budget) [on the government side]	(0) Does not state any consideration	
**LABELING**		
14. Includes some sort of statement/label/logo that makes it clear that the product is fortified	(2) Includes a statement, label, or logo	0.2.2 - products can be identified by the Caricom Standard Mark; 9.1.f - described as "enriched" or "vitamin enriched" if levels are in accordance with Table 2
15. Provides guidance on health claims that can be made for this product (specific to micronutrients added through fortification)	(2) Provides guidance on health claims specific to micronutrients added through fortification	
**INTERNAL MONITORING (conducted by industry)**		
16. If samples are to be taken, describes the sampling process: for example, number of samples, amount, frequency, individual vs. composite, where samples are taken in the process, and percent considered passing	(2) States that samples should be taken as part of internal monitoring	11.4 - describes sampling during production
17. States that industry is required to follow quality assurance/quality control in regards to fortification	(2) States requirement of QA/QC for fortification	3.4 - mill processing shall be done so that there are minimal adverse effects on the nutritive value and useful technological characteristics of the flour; 10 - QA/QC system must conform to ISO 9002-1987, may include sampling/test procedures
18. States applicability of using qualitative testing to determine the presence or absence of a vitamin or mineral (spot tests, iChecks)	(0) Does not state	
**EXTERNAL MONITORING (conducted by government)**		
19.States requirement for external monitoring at the production site to assure compliance with standards and regulations	(0) Does not state requirement	
20. Describes protocols and systems for regulatory monitoring	(0) Does not describe	
21. If there are two or more government agencies involved in external monitoring, clarifies the roles and responsibilities between different government agencies in external monitoring	(2) Clarifies roles and responsibilities for more than one agency	
22. Allows for monitoring to be conducted often enough that problems can be identified and addressed on a timely basis; specifies a timeline for inspections (i.e. once every 6 months, increasing to once every 2 months if a discrepancy is found)	(0) Does not state	
23. If samples are to be taken, describes the sampling process: for example, number of samples, amount, frequency, individual vs. composite, where samples are taken in the process, and percent considered passing	(N/A) Does not describe the sampling process	
24. States applicability of using qualitative testing to determine the presence or absence of a vitamin or mineral (spot tests, iChecks)	(0) Does not state	

25. States registration is required in order to use a logo/be licensed to produce fortified foods	(2) Describes some type of registration or licensing	10.1 - eligibility requirements to use the Caricom Standard Mark and/or StV&G Standard Mark
**COMMERCIAL MONITORING (conducted by government)**		
26. Provides justification for commercial monitoring at retail stores	(0) Does not provide justification for commercial monitoring	
27. Describes protocols and systems for commercial monitoring	(1) Does not explicitly describe, but references protocols and systems for regulatory monitoring	11.2 Sampling described, but nothing else
28. If there are two or more government agencies involved in commercial monitoring, clarifies the roles and responsibilities between different government agencies in commercial monitoring	(2) Clarifies roles and responsibilities for more than one agency	
29. Allows for monitoring to be conducted often enough that problems at the production site or import companies can be identified and addressed on a timely basis; specifies a timeline for inspections (i.e. once every 6 months) or works with production companies to correct noncompliance	(0) Does not state	Table 4 - number of samples depending on lot size
30. If samples are to be taken, describes the sampling process: for example, number of samples, amount, frequency, individual vs. composite, where samples are taken in the process, and percent considered passing	(2) States that samples should be taken as part of commercial monitoring	11.2 - describes sampling process in detail
**IMPORT MONITORING (conducted by government)**		
31. Provides justification for import monitoring at points of entry	(0) Does not provide justification for import monitoring	
32. Describes protocols and systems for import monitoring	(1) Does not explicitly state, but references protocols and systems for import monitoring	11.3 Sampling
33. If there are two or more government agencies involved in import monitoring, clarifies the roles and responsibilities between different government agencies in import monitoring	(2) Clarifies roles and responsibilities for more than one agency	
34. If samples are to be taken, describes the sampling process: for example, number of samples, amount, frequency, individual vs. composite, where samples are taken in the process, and percent considered passing	(2) States that samples should be taken as part of import monitoring	11.3 Sampling from bulk containers, freight containers, and tank trucks (presumably for import/export)
**ENFORCEMENT/PENALTIES**		
35. Indicates roles and responsibilities in enforcing the legislation	(2) States the role and responsibilities of government in enforcement	
36. States incentives to start fortification	(0) Does not state	
37. States incentives to continue fortification, including ensuring compliance	(0) Does not state	
38. States penalties to compel compliance	(0) Does not state	
39. Penalties are objectively defined (e.g. first penalty=$100, second penalty=$300)	(N/A) No penalties are stated (Answered (0) to previous question)	
40. States that enforcement is required to include feedback and support to improve performance and correct noncompliance	(0) Does not require	
**LABORATORY**		
41. References required analytical assays for nutrients (e.g. Liquid chromatography-mass spectrometry for folic acid, atomic absorption for iron and zinc.)	(2) References required assays	12.3 - nutrient assays should follow analytical methods from AOAC, ICC, IUPAC, or AACC
42. States recognition that laboratory results are subject to several sources of variation and do not provide conclusive evidence of compliance or noncompliance	(0) Does not state recognition	
43. Focuses on the quantitative analysis of "marker" micronutrients such as iron	(0) Does not state	
**REPORTING**		
44. States how government monitoring results are shared with stakeholders	(0) Does not state how results are shared	

Chile Wheat

Element	Scoring (use drop-down menus)	Comments (open text)
**GENERAL**		
1. States that legislation applies to at least one food vehicle fit for human consumption (types/grades to be fortified)	(2) States at least one type fit for human consumption	"Desde la década del 50 del siglo pasado, en Chile, ha sido obligatoria la fortificación de la harina de trigo con tiamina, riboflavina, niacina y hierro, además, el Ministerio de Salud de Chile decidió incorporar el ácido fólico en la fortificación de la harina de trigo, en forma obligatoria a partir de enero del año 2000, tal como lo establece el artículo 350 Decreto 977/1996."
2. States the public health objective; purpose and scope of legislation	(2) States the public health objective or general purpose of legislation	"I. ANTECEDENTES<br>Desde la década del 50 del siglo pasado, en Chile, ha sido obligatoria la fortificación de la harina de trigo con tiamina, riboflavina, niacina y hierro, además, el Ministerio de Salud de Chile decidió incorporar el ácido fólico en la fortificación de la harina de trigo, en forma obligatoria a partir de enero del año 2000, tal como lo establece el artículo 350 Decreto 977/1996. Estas medidas de Salud Pública han demostrado ser exitosas ya que han permitido reducir el índice de riesgo nutricional en hombres y mujeres de todas las edades derivados de déficit de dichas vitaminas, han reducido la prevalencia de defectos de cierre del tubo neural en un 43,3 % y, aunque no hay evaluación formal del impacto de la fortificación de la harina con hierro, evidencias indirectas sugieren que las bajas frecuencias de anemia observadas desde 1974 hasta la actualidad en escolares, adolescentes, y mujeres en edad fértil se explicarían en una parte importante por la fortificación, especialmente desde 1967i."
3. References latest available science or accepted international norms and recommendations, particularly for items that may not be covered in the country's documents	(0) Does not state	
4. Provides definitions that includes terms that are specific to fortification (e.g. fortified food, premix, fortificant, food vehicle)	(2) States at least one term related to fortification	VI GLOSARIO
5. Provides repeals (if there is at least one prior document about fortification)	(0) Does not state	
6. Provides effective date or gives grace period for when fortification is to begin (e.g. effective 6 months from signing)	(2) States effective date or grace period for when fortification is to begin (e.g. effective 6 months from signing)	Ref 2 focused on the addition of folic acid to wheat flour; other nutrients have been added to wheat flour for decades. "IV. EJECUCIÓN DEL PROGRAMA DE FORTIFICACIÓN CON ÁCIDO FOLICO<br>A. IMPLEMENTACION<br>La aplicación de la fortificación de la harina de trigo con ácido fólico en los niveles señalados en el artículo 350 del Reglamento, se deberá iniciar a partir del 1° de Enero del año 2000. se contempla la inicio un periodo de 6 meses, como un periodo de ajuste, y luego de esos 6 meses la autoridad sanitaria empezará a sancionar si este no se cumple dentro de lo establecido."
**MICRONUTRIENTS/PREMIX**		
7. States nutrients required	(2) States nutrients	Ref 2 focused on the addition of folic acid to wheat flour; other nutrients have been added to wheat flour for decades. "Forma química del nutriente y cantidad que se usará en la fortificación: Para la fortificación de la harina de trigo se utilizará el ácido fólico y tal como establece el artículo 350 del Reglamento Sanitario de los Alimentos, la harina de trigo deberá contener mínimo 2 y un máximo 2,4 mg de ácido fólico/kg de harina." "Cuadro 1. Contenido (mg/kg) de vitaminas y hierro en harinas 80% extracción" (Ref 3) "Artículo 350.- La harina deberá contener como mínimo las siguientes cantidades de vitaminas y sales minerales:"
8. States fortificants (chemical compounds) to be used (including fortificants that are allowable as options)	(2) States fortificants for at least one nutrient	Ref 2 focused on the addition of folic acid to wheat flour; other nutrients have been added to wheat flour for decades. "Forma química del nutriente y cantidad que se usará en la fortificación: Para la fortificación de la harina de trigo se utilizará el ácido fólico y tal como establece el artículo 350 del Reglamento Sanitario de los Alimentos, la harina de trigo deberá contener mínimo 2 y un máximo 2,4 mg de ácido fólico/kg de harina." (Ref 3) "El hierro debe agregarse en forma de sulfato ferroso, en el evento de no ser esto posible podrá usarse fumarato ferroso siempre que se mantenga la equivalencia con el sulfato ferroso."

9. States fortification levels	(2) States a range or number with +/-	Ref 2 focused on the addition of folic acid to wheat flour; other nutrients have been added to wheat flour for decades. "Forma química del nutriente y cantidad que se usará en la fortificación: Para la fortificación de la harina de trigo se utilizará el ácido fólico y tal como establece el artículo 350 del Reglamento Sanitario de los Alimentos, la harina de trigo deberá contener mínimo 2 y un máximo 2,4 mg de ácido fólico/kg de harina." "Cuadro 1. Contenido (mg/kg) de vitaminas y hierro en harinas 80% extracción"
10. States consideration of bioavailability/biological activity of fortificants	(2) States some consideration of bioavailability (mentions these or related terms)	"El hierro debe agregarse en forma de sulfato ferroso, en el evento de no ser esto posible podrá usarse fumarato ferroso siempre que se mantenga la equivalencia con el sulfato ferroso."
11. States consideration of nutrient stability	(2) States consideration of nutrient stability	Ref 2 focused on the addition of folic acid to wheat flour; other nutrients have been added to wheat flour for decades. "Para la definición e la cantidad de nutriente a incorporar se consideraron entre otros factores tales como: forma química del nutriente que se va adicionar, ingesta recomendada de folato, aporte de folato en las dietas habituales la cantidad de harina de trigo que consume la población, la estabilidad de ácido fólico adicionado a la harina de trigo y los riesgos que puede presentar la ingesta excesiva de ácido fólico."
**COSTING**		
12. States that the cost of fortification is regulated through cost sharing schemes (between government, industry, consumers) or tax measures [to assist industry]	(0) Does not state any consideration	
13. States consideration of the financial responsibility of monitoring and enforcing fortification (schedule of fees, budget) [on the government side]	(0) Does not state any consideration	
**LABELING**		
14. Includes some sort of statement/label/logo that makes it clear that the product is fortified	(2) Includes a statement, label, or logo	(Ref 3) "i) “Con adición de vitaminas y/o minerales”. Si se adicionan vitaminas y/o minerales, estos productos alimenticios deberán clasificarse según corresponda como “Alimento Fortificado” o “Suplemento Alimentario”, respetando los límites establecidos para cada nutriente en cada categoría."
15. Provides guidance on health claims that can be made for this product (specific to micronutrients added through fortification)	(2) Provides guidance on health claims specific to micronutrients added through fortification	(Ref 3) Gives guidance on when can use terms enriched or fortified. ARTÍCULO 120 … “ENRIQUECIDO” “FORTIFICADO” "Este descriptor sólo podrá usarse si el alimento ha sido modificado agregando un 10% ó más de la DDR, por porción de consumo habitual."…
**INTERNAL MONITORING (conducted by industry)**		
16. If samples are to be taken, describes the sampling process: for example, number of samples, amount, frequency, individual vs. composite, where samples are taken in the process, and percent considered passing	(N/A) Does not describe the sampling process	
17. States that industry is required to follow quality assurance/quality control in regards to fortification	(0) Does not state requirement	
18. States applicability of using qualitative testing to determine the presence or absence of a vitamin or mineral (spot tests, iChecks)	(0) Does not state	
**EXTERNAL MONITORING (conducted by government)**		
19.States requirement for external monitoring at the production site to assure compliance with standards and regulations	(2) States requirement for external monitoring or the need for audits/inspections	
20. Describes protocols and systems for regulatory monitoring	(2) Includes checklists or provides detailed description of regulatory monitoring procedures	"IV ACTIVIDADES DE VIGILANCIA" Describes protocols in detail. Form to register samples taken is in ANEXO 2. "V ACTIVIDADES DE FISCALIZACIÓN ... El proceso de fiscalización se realizará de acuerdo a lo establecido en el artículo 69 del Reglamento Sanitario de los Alimentos, y se contempla dos tipos de acciones, las cuales pueden realizarse en conjunto o separadamente, según lo determine la Autoridad Sanitaria competente. 1. Auditoría de la sistematización de las buenas prácticas de fabricación de las harinas de trigo. 2. Control de la fortificación por muestreo del producto y análisis de laboratorio de las vitaminas y minerales contemplados en el artículo 350 del Reglamento Sanitario de los Alimentos."

21. If there are two or more government agencies involved in external monitoring, clarifies the roles and responsibilities between different government agencies in external monitoring	(N/A) Only one government agency involved	"Le corresponde a las Secretarías Regionales Ministeriales de Salud la verificación del cumplimiento de la normativa, para lo cual se establecen planes de vigilancia y fiscalización de la harina de trigo de producción nacional e importada. Dentro de sus actividades se planifican las visitas a los molinos e importadores de harina de trigo, proveedores de premezclas de vitaminas y minerales y muestreos del producto. El Instituto de Salud Pública (ISP) es el Laboratorio Nacional de Referencia para la red de laboratorios del sector salud, hasta ahora es el único laboratorio que ha llevado la centralización de la cuantificación de estos micronutrientes en la matriz harina proveniente de las SEREMI de Salud del país."
22. Allows for monitoring to be conducted often enough that problems can be identified and addressed on a timely basis; specifies a timeline for inspections (i.e. once every 6 months, increasing to once every 2 months if a discrepancy is found)	(2) Describes frequency and how it is responsive to the needs of industry or the stage of fortification implementation	"IV ACTIVIDADES DE VIGILANCIA …<br>La frecuencia del muestreo será de cuatro veces al año. Los meses en que se haga el muestreo serán definidos por cada SEREMI de Salud en coordinación con el Instituto de Salud Pública. … Como productos de las actividades de vigilancia se llevarán a cabo las siguientes acciones:<br>El ISP entregará los resultados de los análisis de laboratorio de los molinos por Región a las SEREMI de Salud correspondiente quienes a su vez notificarán de los resultados a los molinos respectivos."
23. If samples are to be taken, describes the sampling process: for example, number of samples, amount, frequency, individual vs. composite, where samples are taken in the process, and percent considered passing	(2) States that samples should be taken as part of external monitoring	(Ref 1) Very detailed in section IV. "IV ACTIVIDADES DE VIGILANCIA<br>Se ha realizado un estudio en la Región Metropolitana que muestra que el coeficiente de variación producido por el método de muestreo, se minimiza al utilizar un protocolo de muestreo de 8 unidades muestreales tomadas cada media hora durante media jornada de trabajo del molino, para conformar una muestra compuesta de 400 g. ... "<br>(Ref 2) I think this refers to the passing rate of samples. "Cuadro 7. Probabilidad de aceptación del plan de inspección propuesto"
24. States applicability of using qualitative testing to determine the presence or absence of a vitamin or mineral (spot tests, iChecks)	(0) Does not state	
25. States registration is required in order to use a logo/be licensed to produce fortified foods	(0) Does not state that registration or licensing is required	
**COMMERCIAL MONITORING (conducted by government)**		
26. Provides justification for commercial monitoring at retail stores	(0) Does not provide justification for commercial monitoring	Not applicable; no commercial monitoring described
27. Describes protocols and systems for commercial monitoring	(0) Does not describe	Not applicable; no commercial monitoring described
28. If there are two or more government agencies involved in commercial monitoring, clarifies the roles and responsibilities between different government agencies in commercial monitoring	(0) Clarifies roles and responsibilities for no agencies	Not applicable; no commercial monitoring described
29. Allows for monitoring to be conducted often enough that problems at the production site or import companies can be identified and addressed on a timely basis; specifies a timeline for inspections (i.e. once every 6 months) or works with production companies to correct noncompliance	(N/A) No commercial monitoring occurs	Not applicable; no commercial monitoring described
30. If samples are to be taken, describes the sampling process: for example, number of samples, amount, frequency, individual vs. composite, where samples are taken in the process, and percent considered passing	(N/A) Does not describe the sampling process	Not applicable; no commercial monitoring described
**IMPORT MONITORING (conducted by government)**		
31. Provides justification for import monitoring at points of entry	(2) Provides justification for import monitoring	Le corresponde a las Secretarías Regionales Ministeriales de Salud la verificación del cumplimiento de la normativa, para lo cual se establecen planes de vigilancia y fiscalización de la harina de trigo de producción nacional e importada. Dentro de sus actividades se planifican las visitas a los molinos e importadores de harina de trigo, proveedores de premezclas de vitaminas y minerales y muestreos del producto."
32. Describes protocols and systems for import monitoring	(0) Does not state	

33. If there are two or more government agencies involved in import monitoring, clarifies the roles and responsibilities between different government agencies in import monitoring	(N/A) Only one government agency involved	"Le corresponde a las Secretarías Regionales Ministeriales de Salud la verificación del cumplimiento de la normativa, para lo cual se establecen planes de vigilancia y fiscalización de la harina de trigo de producción nacional e importada. Dentro de sus actividades se planifican las visitas a los molinos e importadores de harina de trigo, proveedores de premezclas de vitaminas y minerales y muestreos del producto. El Instituto de Salud Pública (ISP) es el Laboratorio Nacional de Referencia para la red de laboratorios del sector salud, hasta ahora es el único laboratorio que ha llevado la centralización de la cuantificación de estos micronutrientes en la matriz harina proveniente de las SEREMI de Salud del país."
34. If samples are to be taken, describes the sampling process: for example, number of samples, amount, frequency, individual vs. composite, where samples are taken in the process, and percent considered passing	(N/A) Does not describe the sampling process	My read of ref 1 is that the sampling is for the mills, not the importers or importation sites.
**ENFORCEMENT/PENALTIES**		
35. Indicates roles and responsibilities in enforcing the legislation	(0) Does not state	
36. States incentives to start fortification	(0) Does not state	
37. States incentives to continue fortification, including ensuring compliance	(0) Does not state	
38. States penalties to compel compliance	(2) States any penalties	(Ref 1)"V ACTIVIDADES DE FISCALIZACIÓN ...<br>Si producto de las actividades de fiscalización, se verifica incumplimientos del Reglamento Sanitario de los Alimentos, la autoridad sanitaria respectiva realizará las acciones correspondientes de acuerdo a sus atribuciones.<br>(Ref 3) "DE LAS SANCIONES<br>ARTÍCULO 542.- Las infracciones a las disposiciones del presente reglamento serán sancionadas por los Servicios de Salud en cuyo territorio se hayan cometido, previa instrucción del respectivo sumario, en conformidad con lo establecido en el Libro X del Código Sanitario."
39. Penalties are objectively defined (e.g. first penalty=$100, second penalty=$300)	(0) Penalties are not objectively laid out	
40. States that enforcement is required to include feedback and support to improve performance and correct noncompliance	(0) Does not require	
**LABORATORY**		
41. References required analytical assays for nutrients (e.g. Liquid chromatography-mass spectrometry for folic acid, atomic absorption for iron and zinc.)	(2) References required assays	(Ref 1) "III METODOLOGÍA DE ANÁLISIS<br>Las técnicas de laboratorio utilizadas para la evaluación del contenido de vitaminas y minerales establecidos por el Reglamento Sanitario de los Alimentos para la fortificación de harinas con estos nutrientes, serán aquellas que el Instituto de Salud Pública determine y que se encuentran disponibles en su página web o por otros medios."<br>(Ref 2) Has method to quantify folic acid, riboflavin, and thiamin. "V METODOS.<br>A.- ACIDO FOLICO METODO HPLC"
42. States recognition that laboratory results are subject to several sources of variation and do not provide conclusive evidence of compliance or noncompliance	(2) States recognition that lab results are subject to variation	"Evaluaciones de los resultados de los análisis llevados a cabo por el ISP demuestran que los coeficientes de variación de los contenidos de ácido fólico en la harina varían entre molinos, desde el 80% aproximadamente hasta sobre el 200%. Como fuentes de variación de los resultados se pueden encontrar tres: el proceso de fortificación de la harina; el procedimiento seguido para la toma de muestra y el análisis de laboratorio."
43. Focuses on the quantitative analysis of "marker" micronutrients such as iron	(0) Does not state	Quantitatively analyzes four nutrients in samples.
**REPORTING**		
44. States how government monitoring results are shared with stakeholders	(2) States how results are shared with stakeholders	"EL ISP elaborará un informe anual de los resultados de los análisis de laboratorio, el cual debe ser distribuido por el MINSAL a las SEREMI de Salud."

Colombia Wheat

Element	Scoring (use drop-down menus)	Comments (open text)
**GENERAL**		
1. States that legislation applies to at least one food vehicle fit for human consumption (types/grades to be fortified)	(2) States at least one type fit for human consumption	"Artículo 1º. Campo de aplicación. Las disposiciones del presente reglamento se aplican a la harina de trigo que se comercializa en el territorio nacional para la venta directa al consumidor, como para la fabricación de productos de panadería, pastelería, galletería, pastas alimenticias, y otros."
2. States the public health objective; purpose and scope of legislation	(2) States the public health objective or general purpose of legislation	"Que es necesario reglamentar la fortificación de la harina de trigo con micronutrientes deficientes en la dieta colombiana"
3. References latest available science or accepted international norms and recommendations, particularly for items that may not be covered in the country's documents	(0) Does not state	
4. Provides definitions that includes terms that are specific to fortification (e.g. fortified food, premix, fortificant, food vehicle)	(2) States at least one term related to fortification	"Artículo 3º. Para efectos de este decreto se establecen en las siguientes definiciones."
5. Provides repeals (if there is at least one prior document about fortification)	(N/A) No prior documents about fortification	
6. Provides effective date or gives grace period for when fortification is to begin (e.g. effective 6 months from signing)	(2) States effective date or grace period for when fortification is to begin (e.g. effective 6 months from signing)	"Artículo 12. Los industriales fabricantes de la harina de trigo tendrán un plazo de 8 meses para dar cumplimiento a lo previsto en este Decreto."
**MICRONUTRIENTS/PREMIX**		
7. States nutrients required	(2) States nutrients	"Artículo 2º. Obligatoriedad de fortificación. La harina de trigo que se comercializa en el territorio nacional deberá estar fortificada con vitamina B1, vitamina B2, niacina, acido fólico y hierro. Parágrafo. La adición de calcio podrá hacerse de manera opcional."
8. States fortificants (chemical compounds) to be used (including fortificants that are allowable as options)	(2) States fortificants for at least one nutrient	"Artículo 6º. Formas químicas de los micronutrientes. Los micronutrientes que hacen parte de la premezcla deberán ser adicionados en las formas químicas siguientes:"
9. States fortification levels	(1) States one number only	States minimum amount. "Artículo 4º. Requisitos. La harina de trigo que se comercializa en el territorio nacional deberá estar adicionada o añadida con las siguientes cantidades mínimas de micronutrientes por cada kilogramo de harina:"
10. States consideration of bioavailability/biological activity of fortificants	(0) Does not state any consideration	"Artículo 7º. De las competencias técnicas. El Ministerio de Salud podrá modificar los micronutrientes, las formas químicas de los mismos y las cantidades de fortificación, de acuerdo con los avances de los conocimientos científicos del tema."
11. States consideration of nutrient stability	(0) Does not state any consideration	
**COSTING**		
12. States that the cost of fortification is regulated through cost sharing schemes (between government, industry, consumers) or tax measures [to assist industry]	(0) Does not state any consideration	
13. States consideration of the financial responsibility of monitoring and enforcing fortification (schedule of fees, budget) [on the government side]	(0) Does not state any consideration	
**LABELING**		
14. Includes some sort of statement/label/logo that makes it clear that the product is fortified	(2) Includes a statement, label, or logo	"Artículo 9º. Rotulado. El rótulo del envase o empaque de la harina de trigo, además de las condiciones de rotulado señaladas en la Resolución 8688 de 1979 o las que modifiquen, sustituyan o adicionen, deberá contener en forma destacada la leyenda Harina de Trigo Fortificada con la declaración de las cantidades de los micronutrientes adicionados en miligramos por kilogramo (mg/kg) de harina."
15. Provides guidance on health claims that can be made for this product (specific to micronutrients added through fortification)	(0) Does not provide	
**INTERNAL MONITORING (conducted by industry)**		
16. If samples are to be taken, describes the sampling process: for example, number of samples, amount, frequency, individual vs. composite, where samples are taken in the process, and percent considered passing	(N/A) Does not describe the sampling process	
17. States that industry is required to follow quality assurance/quality control in regards to fortification	(0) Does not state requirement	"Artículo 8º. De la responsabilidad. La fortificación de la harina de trigo con los micronutrientes es responsabilidad de los industriales fabricantes de la harina de trigo. Así mismo, para la fabricación de productos alimenticios en los cuales se utilice esta materia prima, deberán elaborarse con harina de trigo fortificada según los requisitos establecidos en este Decreto."

18. States applicability of using qualitative testing to determine the presence or absence of a vitamin or mineral (spot tests, iChecks)	(0) Does not state	
**EXTERNAL MONITORING (conducted by government)**		
19.States requirement for external monitoring at the production site to assure compliance with standards and regulations	(2) States requirement for external monitoring or the need for audits/inspections	Does not state from where INVIMA will take samples of flour and premix; it is implicit. "Artículo 10. Vigilancia y control. El control y la vigilancia en el cumplimiento de las disposiciones contenidos en el presente Decreto para la Harina de Trigo Fortificada y de las premezclas, estará a cargo del Instituto Nacional de Vigilancia de Medicamentos y Alimentos, Invima, y las entidades territoriales competentes. Se tomarán periódicamente muestras de harina de trigo y de las premezclas para su análisis por parte del Invima."
20. Describes protocols and systems for regulatory monitoring	(0) Does not describe	
21. If there are two or more government agencies involved in external monitoring, clarifies the roles and responsibilities between different government agencies in external monitoring	(N/A) Only one government agency involved	"Artículo 10. Vigilancia y control. El control y la vigilancia en el cumplimiento de las disposiciones contenidos en el presente Decreto para la Harina de Trigo Fortificada y de las premezclas, estará a cargo del Instituto Nacional de Vigilancia de Medicamentos y Alimentos, Invima, y las entidades territoriales competentes. Se tomarán periódicamente muestras de harina de trigo y de las premezclas para su análisis por parte del Invima."
22. Allows for monitoring to be conducted often enough that problems can be identified and addressed on a timely basis; specifies a timeline for inspections (i.e. once every 6 months, increasing to once every 2 months if a discrepancy is found)	(0) Does not state	
23. If samples are to be taken, describes the sampling process: for example, number of samples, amount, frequency, individual vs. composite, where samples are taken in the process, and percent considered passing	(1) States that samples should be taken (generally)	"Artículo 10. Vigilancia y control. El control y la vigilancia en el cumplimiento de las disposiciones contenidos en el presente Decreto para la Harina de Trigo Fortificada y de las premezclas, estará a cargo del Instituto Nacional de Vigilancia de Medicamentos y Alimentos, Invima, y las entidades territoriales competentes. Se tomarán periódicamente muestras de harina de trigo y de las premezclas para su análisis por parte del Invima."
24. States applicability of using qualitative testing to determine the presence or absence of a vitamin or mineral (spot tests, iChecks)	(0) Does not state	
25. States registration is required in order to use a logo/be licensed to produce fortified foods	(0) Does not state that registration or licensing is required	
**COMMERCIAL MONITORING (conducted by government)**		
26. Provides justification for commercial monitoring at retail stores	(0) Does not provide justification for commercial monitoring	
27. Describes protocols and systems for commercial monitoring	(0) Does not describe	
28. If there are two or more government agencies involved in commercial monitoring, clarifies the roles and responsibilities between different government agencies in commercial monitoring	(0) Clarifies roles and responsibilities for no agencies	
29. Allows for monitoring to be conducted often enough that problems at the production site or import companies can be identified and addressed on a timely basis; specifies a timeline for inspections (i.e. once every 6 months) or works with production companies to correct noncompliance	(N/A) No commercial monitoring occurs	
30. If samples are to be taken, describes the sampling process: for example, number of samples, amount, frequency, individual vs. composite, where samples are taken in the process, and percent considered passing	(N/A) Does not describe the sampling process	
**IMPORT MONITORING (conducted by government)**		
31. Provides justification for import monitoring at points of entry	(2) Provides justification for import monitoring	(Ref 1) "Artículo 4º. Requisitos. ... Parágrafo. La harina de trigo que se importe y se comercialice en el país deberá cumplir con estos requisitos." (Ref 2) Domestically produced or imported food products with wheat flour as main ingredient must use fortified wheat flour. "Por lo anterior, el titular del Registro Sanitario de estos productos debe garantizar el cumplimiento del a fortificación de la harina de trigo que emplea para la elaboración de los mismos, se trate de alimentos elaborados en el país o importados".
32. Describes protocols and systems for import monitoring	(0) Does not state	
33. If there are two or more government agencies involved in import monitoring, clarifies the roles and responsibilities between different government agencies in import monitoring	(0) Clarifies roles and responsibilities for no agencies	

34. If samples are to be taken, describes the sampling process: for example, number of samples, amount, frequency, individual vs. composite, where samples are taken in the process, and percent considered passing	(N/A) Does not describe the sampling process	
**ENFORCEMENT/PENALTIES**		
35. Indicates roles and responsibilities in enforcing the legislation	(2) States the role and responsibilities of government in enforcement	"Artículo 10. Vigilancia y control. El control y la vigilancia en el cumplimiento de las disposiciones contenidos en el presente Decreto para la Harina de Trigo Fortificada y de las premezclas, estará a cargo del Instituto Nacional de Vigilancia de Medicamentos y Alimentos, Invima, y las entidades territoriales competentes. Se tomarán periódicamente muestras de harina de trigo y de las premezclas para su análisis por parte del Invima."
36. States incentives to start fortification	(0) Does not state	
37. States incentives to continue fortification, including ensuring compliance	(0) Does not state	
38. States penalties to compel compliance	(2) States any penalties	"Artículo 11. De la aplicación de las medidas sanitarias. Corresponde al Instituto Nacional de Vigilancia de Medicamentos y Alimentos, Invima, y a las entidades territoriales o las autoridades sanitarias delegadas tomar las medidas sanitarias preventivas y de seguridad, adelantar los procedimientos y establecer las sanciones que se deriven del incumplimiento de las especificaciones que se señalan en el presente Decreto, conforme a lo establecido en el Decreto 2780 de 1991 o los que lo modifiquen, adicionen o sustituyan."
39. Penalties are objectively defined (e.g. first penalty=$100, second penalty=$300)	(0) Penalties are not objectively laid out	
40. States that enforcement is required to include feedback and support to improve performance and correct noncompliance	(0) Does not require	
**LABORATORY**		
41. References required analytical assays for nutrients (e.g. Liquid chromatography-mass spectrometry for folic acid, atomic absorption for iron and zinc.)	(0) Does not state requirements	
42. States recognition that laboratory results are subject to several sources of variation and do not provide conclusive evidence of compliance or noncompliance	(0) Does not state recognition	
43. Focuses on the quantitative analysis of "marker" micronutrients such as iron	(0) Does not state	
**REPORTING**		
44. States how government monitoring results are shared with stakeholders	(0) Does not state how results are shared	

Congo Wheat

Element	Scoring (use drop-down menus)	Comments (open text)
**GENERAL**		
1. States that legislation applies to at least one food vehicle fit for human consumption (types/grades to be fortified)	(2) States at least one type fit for human consumption	Article 1 - iron-fortified wheat flour for human consumption
2. States the public health objective; purpose and scope of legislation	(0) Does not state	
3. References latest available science or accepted international norms and recommendations, particularly for items that may not be covered in the country's documents	(0) Does not state	
4. Provides definitions that includes terms that are specific to fortification (e.g. fortified food, premix, fortificant, food vehicle)	(0) Does not state	
5. Provides repeals (if there is at least one prior document about fortification)	(0) Does not state	
6. Provides effective date or gives grace period for when fortification is to begin (e.g. effective 6 months from signing)	(2) States effective date or grace period for when fortification is to begin (e.g. effective 6 months from signing)	Article 5 - Within 6 months of signature date
**MICRONUTRIENTS/PREMIX**		
7. States nutrients required	(2) States nutrients	Article 2 - Iron
8. States fortificants (chemical compounds) to be used (including fortificants that are allowable as options)	(2) States fortificants for at least one nutrient	Article 2 - in the form of ferrous fumarate or ferrous sulphate
9. States fortification levels	(1) States one number only	Article 2 - 60ppm
10. States consideration of bioavailability/biological activity of fortificants	(0) Does not state any consideration	
11. States consideration of nutrient stability	(0) Does not state any consideration	
**COSTING**		
12. States that the cost of fortification is regulated through cost sharing schemes (between government, industry, consumers) or tax measures [to assist industry]	(0) Does not state any consideration	
13. States consideration of the financial responsibility of monitoring and enforcing fortification (schedule of fees, budget) [on the government side]	(0) Does not state any consideration	
**LABELING**		
14. Includes some sort of statement/label/logo that makes it clear that the product is fortified	(0) Does not include statement, label, or logo	
15. Provides guidance on health claims that can be made for this product (specific to micronutrients added through fortification)	(0) Does not provide	
**INTERNAL MONITORING (conducted by industry)**		
16. If samples are to be taken, describes the sampling process: for example, number of samples, amount, frequency, individual vs. composite, where samples are taken in the process, and percent considered passing	(N/A) Does not describe the sampling process	
17. States that industry is required to follow quality assurance/quality control in regards to fortification	(0) Does not state requirement	
18. States applicability of using qualitative testing to determine the presence or absence of a vitamin or mineral (spot tests, iChecks)	(0) Does not state	
**EXTERNAL MONITORING (conducted by government)**		
19.States requirement for external monitoring at the production site to assure compliance with standards and regulations	(0) Does not state requirement	
20. Describes protocols and systems for regulatory monitoring	(1) Does not explicitly describe, but references protocols and systems for regulatory monitoring	Article 3 - regulations on iron-fortified wheat flour as well as the specific characteristics thereof are obtained from the general managers of the industry. The modalities of obtaining that information are determined via order by the Minister responsible for the industry.
21. If there are two or more government agencies involved in external monitoring, clarifies the roles and responsibilities between different government agencies in external monitoring	(0) Clarifies roles and responsibilities for no agencies	
22. Allows for monitoring to be conducted often enough that problems can be identified and addressed on a timely basis; specifies a timeline for inspections (i.e. once every 6 months, increasing to once every 2 months if a discrepancy is found)	(0) Does not state	
23. If samples are to be taken, describes the sampling process: for example, number of samples, amount, frequency, individual vs. composite, where samples are taken in the process, and percent considered passing	(N/A) Does not describe the sampling process	
24. States applicability of using qualitative testing to determine the presence or absence of a vitamin or mineral (spot tests, iChecks)	(0) Does not state	
25. States registration is required in order to use a logo/be licensed to produce fortified foods	(0) Does not state that registration or licensing is required	

**COMMERCIAL MONITORING (conducted by government)**		
26. Provides justification for commercial monitoring at retail stores	(0) Does not provide justification for commercial monitoring	
27. Describes protocols and systems for commercial monitoring	(0) Does not describe	
28. If there are two or more government agencies involved in commercial monitoring, clarifies the roles and responsibilities between different government agencies in commercial monitoring	(0) Clarifies roles and responsibilities for no agencies	
29. Allows for monitoring to be conducted often enough that problems at the production site or import companies can be identified and addressed on a timely basis; specifies a timeline for inspections (i.e. once every 6 months) or works with production companies to correct noncompliance	(0) Does not state	
30. If samples are to be taken, describes the sampling process: for example, number of samples, amount, frequency, individual vs. composite, where samples are taken in the process, and percent considered passing	(N/A) Does not describe the sampling process	
**IMPORT MONITORING (conducted by government)**		
31. Provides justification for import monitoring at points of entry	(0) Does not provide justification for import monitoring	
32. Describes protocols and systems for import monitoring	(0) Does not state	
33. If there are two or more government agencies involved in import monitoring, clarifies the roles and responsibilities between different government agencies in import monitoring	(0) Clarifies roles and responsibilities for no agencies	
34. If samples are to be taken, describes the sampling process: for example, number of samples, amount, frequency, individual vs. composite, where samples are taken in the process, and percent considered passing	(N/A) Does not describe the sampling process	
**ENFORCEMENT/PENALTIES**		
35. Indicates roles and responsibilities in enforcing the legislation	(0) Does not state	
36. States incentives to start fortification	(0) Does not state	
37. States incentives to continue fortification, including ensuring compliance	(0) Does not state	
38. States penalties to compel compliance	(2) States any penalties	Article 6 - violations are punished in accordance with the laws and regulations in force
39. Penalties are objectively defined (e.g. first penalty=$100, second penalty=$300)	(0) Penalties are not objectively laid out	
40. States that enforcement is required to include feedback and support to improve performance and correct noncompliance	(0) Does not require	
**LABORATORY**		
41. References required analytical assays for nutrients (e.g. Liquid chromatography-mass spectrometry for folic acid, atomic absorption for iron and zinc.)	(0) Does not state requirements	
42. States recognition that laboratory results are subject to several sources of variation and do not provide conclusive evidence of compliance or noncompliance	(0) Does not state recognition	
43. Focuses on the quantitative analysis of "marker" micronutrients such as iron	(0) Does not state	
**REPORTING**		
44. States how government monitoring results are shared with stakeholders	(0) Does not state how results are shared	

Costa Rica Wheat

Element	Scoring (use drop-down menus)	Comments (open text)
**GENERAL**		
1. States that legislation applies to at least one food vehicle fit for human consumption (types/grades to be fortified)	(2) States at least one type fit for human consumption	"Artículo 1°—Ámbito de aplicación. Las disposiciones del presente reglamento se aplican a la harina de trigo y a la sémola de trigo que se utiliza para consumo humano en el país, sean éstas de producción nacional, importadas o donadas."<br>"Artículo 3°—Obligatoriedad del enriquecimiento. La harina de trigo que se utilice en el país para consumo humano, ya sea de uso casero o en la industria alimentaria, deberá estar enriquecida con hierro y ácido fólico y reconstituida con tiamina, riboflavina y niacina."
2. States the public health objective; purpose and scope of legislation	(2) States the public health objective or general purpose of legislation	"Considerando:<br>1°—Que es función esencial del Estado velar por la protección de la salud de la población.<br>2°—Que la Administración Pública tiene la responsabilidad de garantizar el bienestar de los ciudadanos, sin obstaculizar innecesariamente las condiciones de competitividad para el desenvolvimiento de la actividad económica del país.<br>3°—Que la nutrición adecuada y la ingestión de alimentos de buena calidad y en condiciones sanitarias, son esenciales para la salud.<br>4°—Que los resultados aportados por las Encuestas Nacionales de Nutrición de 1982 y 1996, mostraron que las anemias nutricionales constituyen un problema de salud pública."
3. References latest available science or accepted international norms and recommendations, particularly for items that may not be covered in the country's documents	(0) Does not state	
4. Provides definitions that includes terms that are specific to fortification (e.g. fortified food, premix, fortificant, food vehicle)	(2) States at least one term related to fortification	"Artículo 2°—Definiciones. Para efectos del presente reglamento se entenderá por:"
5. Provides repeals (if there is at least one prior document about fortification)	(2) States repeals	"Artículo 9°—Derogatoria. Deróguese el decreto ejecutivo N° 18 del 28 de julio de 1958 y todos aquellos que se opongan al presente Reglamento."
6. Provides effective date or gives grace period for when fortification is to begin (e.g. effective 6 months from signing)	(2) States effective date or grace period for when fortification is to begin (e.g. effective 6 months from signing)	"Artículo 10.—El presente reglamento regirá a partir del día 1° de noviembre de 1997.<br>Transitorio I.—Se otorga un plazo de 3 meses a partir de la fecha de publicación del presente Reglamento, a efecto de que los productores y distribuidores adapten el material de empaque existentes a las exigencias de lo aquí establecido."
**MICRONUTRIENTS/PREMIX**		
7. States nutrients required	(2) States nutrients	"Artículo 3°—Obligatoriedad del enriquecimiento. La harina de trigo que se utilice en el país para consumo humano, ya sea de uso casero o en la industria alimentaria, deberá estar enriquecida con hierro y ácido fólico y reconstituida con tiamina, riboflavina y niacina."
8. States fortificants (chemical compounds) to be used (including fortificants that are allowable as options)	(2) States fortificants for at least one nutrient	"Artículo 4° ...Hierro(como fumarato ferroso)"
9. States fortification levels	(1) States one number only	"Artículo 4°—Niveles de enriquecimiento. La harina y sémola de trigo deberán ser fortificadas con hierro y vitaminas del complejo B, los cuales pueden provenir de una o varias mezclas con excipientes, de manera tal que una dilución específica de ellos produzca los niveles mínimos que se especifican a continuación: [lists nutrients and levels for wheat flour and for semolina, including intrinsic amount] Estos valores incluyen el contenido intrínseco micronutrientes en la harina y sémola de trigo.<br>Para cumplir con estos requisitos, se recomienda que los niveles de micronutrientes a adicionar durante el proceso de fabricación de la harina y sémola de trigo sean: [lists nutrients to be added through fortification]"
10. States consideration of bioavailability/biological activity of fortificants	(0) Does not state any consideration	
11. States consideration of nutrient stability	(0) Does not state any consideration	
**COSTING**		
12. States that the cost of fortification is regulated through cost sharing schemes (between government, industry, consumers) or tax measures [to assist industry]	(0) Does not state any consideration	
13. States consideration of the financial responsibility of monitoring and enforcing fortification (schedule of fees, budget) [on the government side]	(2) Shows consideration that monitoring costs money	"II. Procedimiento<br>a. Plan, presupuesto y calendario"
**LABELING**		

14. Includes some sort of statement/label/logo that makes it clear that the product is fortified	(2) Includes a statement, label, or logo	"Artículo 6°...<br>El etiquetado de la harina y sémola de trigo se ajustará a lo estipulado en la Norma General de Etiquetado de los Alimentos Preenvasados y deberá especificar que se trata de un producto enriquecido con la frase "HARINA DE TRIGO ENRIQUECIDA" o "HARINA DE TRIGO FORTIFICADA", además deberá contener un panel que incluya el contenido final total de micronutrientes (el adicionado y el contenido naturalmente)."
15. Provides guidance on health claims that can be made for this product (specific to micronutrients added through fortification)	(2) Provides guidance on health claims specific to micronutrients added through fortification	Artículo 6°... Los productos elaborados con harina de trigo enriquecida podrán declarar que son fortificados con hierro y vitaminas, siempre y cuando la proporción de harina en su composición sea superior al 30% (ref `)." Ref 5: "7.1 Declaraciones Nutricionales<br>Las únicas declaraciones de propiedades nutricionales permitidas deben ser las que se refieran a energía, proteínas, carbohidratos, grasas y los componentes de las mismas, fibra, vitaminas y minerales para los cuales se hayan establecido recomendaciones nutricionales."<br>"7.4 Declaraciones de Propiedades Saludables"<br>Anexo G "3.9 Ácido fólico y defectos del conducto (tubo) neural:"
**INTERNAL MONITORING (conducted by industry)**		
16. If samples are to be taken, describes the sampling process: for example, number of samples, amount, frequency, individual vs. composite, where samples are taken in the process, and percent considered passing	(N/A) Does not describe the sampling process	
17. States that industry is required to follow quality assurance/quality control in regards to fortification	(2) States requirement of QA/QC for fortification	"Artículo 5°—Garantía y control de calidad. La garantía de calidad o control interno de la harina enriquecida con los niveles indicados en el artículo 4° son de responsabilidad de los industriales y de los importadores de harina de trigo."
18. States applicability of using qualitative testing to determine the presence or absence of a vitamin or mineral (spot tests, iChecks)	(0) Does not state	
**EXTERNAL MONITORING (conducted by government)**		
19.States requirement for external monitoring at the production site to assure compliance with standards and regulations	(2) States requirement for external monitoring or the need for audits/inspections	"Artículo 5° ...<br>La fiscalización y el monitoreo de la calidad de la fortificación de la harina de trigo en fábricas, sitios de expendio y otros, es responsabilidad del Ministerio de Salud, institución que además diseñará y establecerá un sistema de control de calidad para la harina de trigo enriquecida, asimismo, dicho Ministerio podrá verificar los niveles de las vitaminas en la premezcla y en la harina de trigo, mediante análisis cuantitativos eventuales y establecerá los criterios técnicos de los procedimientos y técnicas de laboratorio, para el análisis de las muestras de harinas enriquecidas."
20. Describes protocols and systems for regulatory monitoring	(2) Includes checklists or provides detailed description of regulatory monitoring procedures	"II. Procedimiento (Inspectores de la Autoridad Sanitaria)"
21. If there are two or more government agencies involved in external monitoring, clarifies the roles and responsibilities between different government agencies in external monitoring	(N/A) Only one government agency involved	"Artículo 5° ...<br>La fiscalización y el monitoreo de la calidad de la fortificación de la harina de trigo en fábricas, sitios de expendio y otros, es responsabilidad del Ministerio de Salud, institución que además diseñará y establecerá un sistema de control de calidad para la harina de trigo enriquecida, asimismo, dicho Ministerio podrá verificar los niveles de las vitaminas en la premezcla y en la harina de trigo, mediante análisis cuantitativos eventuales y establecerá los criterios técnicos de los procedimientos y técnicas de laboratorio, para el análisis de las muestras de harinas enriquecidas."
22. Allows for monitoring to be conducted often enough that problems can be identified and addressed on a timely basis; specifies a timeline for inspections (i.e. once every 6 months, increasing to once every 2 months if a discrepancy is found)	(2) Describes frequency and how it is responsive to the needs of industry or the stage of fortification implementation	"El propósito de la planificación de las visitas de inspección es asegurar que:<br>• se designan los recursos para visitar los molinos de trigo por lo menos dos veces al año[1]. [1] Cuando se inicien las actividades de inspección las visitas deben ser más frecuentes, por lo menos 1 vez cada tres meses. Cuando se demuestre que el proceso está controlado y los resultados son satisfactorios las visitas se pueden espaciar. Debido a que esta actividad está sujeta a presupuesto anual, estos gastos se asignan en el rubro de imprevistos."

23. If samples are to be taken, describes the sampling process: for example, number of samples, amount, frequency, individual vs. composite, where samples are taken in the process, and percent considered passing	(2) States that samples should be taken as part of external monitoring	"c. Inspección<br>4. Al final de la auditoría, tome cinco4 (5) muestras de harina para la inspección por pruebas de verificación o corroboración y una muestra de la premezcla que se está utilizando en ese momento (refiérase a la sección C)."
24. States applicability of using qualitative testing to determine the presence or absence of a vitamin or mineral (spot tests, iChecks)	(2) States applicability of spot test to determine presence/absence of vitamin or mineral specific to external monitoring	"Todas las muestras de harina (incluyendo las muestras simples) contienen hierroadicionado, el cual se utiliza como micronutriente "indicador"[6]:<br>Mancha de hierro (prueba cualitativa) [7]/Presente"<br>"[7] La prueba cualitativa de hierro se usa para asegurar que todas las muestras individuales tomadas en el molino están fortificadas y no introducir sesgo al preparar muestras compuestas con muestras sin fortificación. Si una muestra no presenta hierro es señal de un problema en el proceso que se debe investigar. Los resultados de esta prueba no se utilizan para concluir y tomar acciones sobre incumplimientos en la fortificación, solamente los resultados de los análisis cuantitativos realizados en el laboratorio de la Autoridad Sanitaria o ente regulador responsable."
25. States registration is required in order to use a logo/be licensed to produce fortified foods	(2) Describes some type of registration or licensing	"Artículo 5° ...<br>El registro sanitario de las harinas enriquecidas y de los derivados de las harinas, será otorgado por el Ministerio de Salud y tendrá una vigencia de un año. Su renovación deberá ser solicitada cumpliéndose con los requisitos del caso y antes de su vencimiento."
**COMMERCIAL MONITORING (conducted by government)**		
26. Provides justification for commercial monitoring at retail stores	(2) Provides justification for commercial monitoring	"El Ministerio de Salud se reserva el derecho de confirmar, mediante muestreo y análisis de muestras en el mercado o en las bodegas del importador, el cumplimiento de la harina importada con los niveles de enriquecimiento establecidos en el presente reglamento. Los costos que esto ocasione serán asumidos por el importador."
27. Describes protocols and systems for commercial monitoring	(2) Includes checklists or provides detailed description of regulatory monitoring procedures	"B. VISITAS DE INSPECCIÓN A LOS SITIOS DE VENTA AL DETALLE, AL POR MAYOR Y PANADERÍAS"
28. If there are two or more government agencies involved in commercial monitoring, clarifies the roles and responsibilities between different government agencies in commercial monitoring	(N/A) Only one government agency involved	"El Ministerio de Salud se reserva el derecho de confirmar, mediante muestreo y análisis de muestras en el mercado o en las bodegas del importador, el cumplimiento de la harina importada con los niveles de enriquecimiento establecidos en el presente reglamento. Los costos que esto ocasione serán asumidos por el importador."
29. Allows for monitoring to be conducted often enough that problems at the production site or import companies can be identified and addressed on a timely basis; specifies a timeline for inspections (i.e. once every 6 months) or works with production companies to correct noncompliance	(1) Makes mention of a timeline	This is focused on commercial monitoring only:<br>"• Basado en el número total de distritos del país, planifique por lo menos una visita al año a cada uno de los distritos del país. La frecuencia e intensidad de muestreo depende de la densidad poblacional, la cantidad de alimento vendido en la región y factores de riesgos tales como la ubicación cerca de fronteras con otros países donde el alimento no se fortifica. Se podrían incluir las panaderías como un lugar para tomar muestras."

30. If samples are to be taken, describes the sampling process: for example, number of samples, amount, frequency, individual vs. composite, where samples are taken in the process, and percent considered passing	(2) States that samples should be taken as part of commercial monitoring	"Este manual describe los procedimientos para realizar las visitas de inspección en cualquier establecimiento que venda alimentos fortificados."<br>"V. Procedimientos<br>a. Visitas de los inspectores<br>5. Escoja un empaque sellado de aproximadamente 500 g (0.5 kg) de cada marca de alimento fortificado en el establecimiento. Si el alimento no se encuentra en esta presentación, tome la muestra de alimento en la siguiente presentación para venta al detalle. Si los envases son más pequeños, recolecte suficientes paquetes para llegar al peso especificado (Ej.: 2 empaques de 250 g).<br>6. Si el alimento se vende por peso o volumen de sacos más grandes, tome una muestra de aproximadamente 500 g (0.5 kg) de este producto. Asegúrese que el saco es nuevo, de otra forma no existe garantía que el producto adentro del mismo corresponde al nombre de la fábrica en la etiqueta.<br>7. Empaque las muestras dentro de una caja y transpórtelas a la oficina local de la Autoridad Sanitaria, donde el encargado de la misma enviará las muestras y copias de los informes a la sede de la Autoridad Sanitaria con la frecuencia que se ha determinado en el plan de trabajo anual."
**IMPORT MONITORING (conducted by government)**		
31. Provides justification for import monitoring at points of entry	(2) Provides justification for import monitoring	"Artículo 5°—Garantía y control de calidad. La garantía de calidad o control interno de la harina enriquecida con los niveles indicados en el artículo 4° son de responsabilidad de los industriales y de los importadores de harina de trigo. Para autorizar el desalmacenaje de la harina de trigo importada, el importador deberá demostrar mediante el certificado de calidad emitido por una autoridad del país de origen o certificado de análisis realizado en un laboratorio acreditado, la conformidad del producto con el enriquecimiento establecido en el presente reglamento, para cada partida de importación.<br>El Ministerio de Salud se reserva el derecho de confirmar, mediante muestreo y análisis de muestras en el mercado o en las bodegas del importador, el cumplimiento de la harina importada con los niveles de enriquecimiento establecidos en el presente reglamento. Los costos que esto ocasione serán asumidos por el importador."
32. Describes protocols and systems for import monitoring	(2) Includes checklists or detailed description of import monitoring procedures	"Artículo 5°—Garantía y control de calidad. La garantía de calidad o control interno de la harina enriquecida con los niveles indicados en el artículo 4° son de responsabilidad de los industriales y de los importadores de harina de trigo. Para autorizar el desalmacenaje de la harina de trigo importada, el importador deberá demostrar mediante el certificado de calidad emitido por una autoridad del país de origen o certificado de análisis realizado en un laboratorio acreditado, la conformidad del producto con el enriquecimiento establecido en el presente reglamento, para cada partida de importación.<br>El Ministerio de Salud se reserva el derecho de confirmar, mediante muestreo y análisis de muestras en el mercado o en las bodegas del importador, el cumplimiento de la harina importada con los niveles de enriquecimiento establecidos en el presente reglamento. Los costos que esto ocasione serán asumidos por el importador."
33. If there are two or more government agencies involved in import monitoring, clarifies the roles and responsibilities between different government agencies in import monitoring	(N/A) Only one government agency involved	"Artículo 5°...<br>El Ministerio de Salud se reserva el derecho de confirmar, mediante muestreo y análisis de muestras en el mercado o en las bodegas del importador, el cumplimiento de la harina importada con los niveles de enriquecimiento establecidos en el presente reglamento. Los costos que esto ocasione serán asumidos por el importador."
34. If samples are to be taken, describes the sampling process: for example, number of samples, amount, frequency, individual vs. composite, where samples are taken in the process, and percent considered passing	(1) States that samples should be taken (generally)	"Artículo 5°...<br>El Ministerio de Salud se reserva el derecho de confirmar, mediante muestreo y análisis de muestras en el mercado o en las bodegas del importador, el cumplimiento de la harina importada con los niveles de enriquecimiento establecidos en el presente reglamento. Los costos que esto ocasione serán asumidos por el importador."
**ENFORCEMENT/PENALTIES**		

35. Indicates roles and responsibilities in enforcing the legislation	(2) States the role and responsibilities of government in enforcement	"Artículo 8°...<br>Asimismo, el Ministerio de Salud coordinará con los demás entes públicos involucrados en la materia, todas las acciones necesarias para asegurar la cabal aplicación y cumplimiento del presente Reglamento."
36. States incentives to start fortification	(0) Does not state	
37. States incentives to continue fortification, including ensuring compliance	(0) Does not state	
38. States penalties to compel compliance	(2) States any penalties	"Artículo 5° ...<br>En caso de establecerse que un lote de harina de trigo producida en el país o importada no esté debidamente enriquecida, se procederá al decomiso de la cantidad total, siguiendo los procedimientos establecidos por el Ministerio de Salud."
39. Penalties are objectively defined (e.g. first penalty=$100, second penalty=$300)	(0) Penalties are not objectively laid out	
40. States that enforcement is required to include feedback and support to improve performance and correct noncompliance	(0) Does not require	
**LABORATORY**		
41. References required analytical assays for nutrients (e.g. Liquid chromatography-mass spectrometry for folic acid, atomic absorption for iron and zinc.)	(2) References required assays	"8.1 Métodos físico-químicos ...<br>AOAC 14.011 Determinación del hierro<br>AOAC 43.046 Determinación de niacina<br>AOAC 43.144 Determinación de ácido fólico<br>AOAC 957.17 Determinación de Vitamina B1<br>AOAC 970.65 Determinación de Vitamina B2"
42. States recognition that laboratory results are subject to several sources of variation and do not provide conclusive evidence of compliance or noncompliance	(0) Does not state recognition	
43. Focuses on the quantitative analysis of "marker" micronutrients such as iron	(2) Focuses on quantitative analysis of marker micronutrient such as iron	"c. Análisis de las muestras<br>9. Al recibir las muestras, el laboratorio deberá detectar la presencia de los micronutrientes indicador4 en todas las muestras simples usando pruebas cualitativas."
**REPORTING**		
44. States how government monitoring results are shared with stakeholders	(2) States how results are shared with stakeholders	Refers to external monitoring only (ref 3): "Los resultados de las actividades de auditoría e inspección deberían ser consolidadas dos veces al año y determinar el grado de cumplimiento de las metas de fortificación, los obstáculos a superar y las acciones a tomar. Se recomienda preparar y publicar un informe anual donde los resultados del monitoreo externo se presenten gráficamente para divulgar la situación del programa de fortificación en el país, junto con resultados de otras actividades de control de alimentos o de vigilancia tales como monitoreo comercial o vigilancia en hogares." This document only refers to the commercial monitoring results (ref 4): "Los resultados de las actividades de monitoreo comercial se deberían consolidar dos veces al año en informes. Estos informes contribuyen a definir el grado de éxito en el cumplimiento de las metas de fortificación, exponer los obstáculos que se necesitan superar y definir las acciones a seguir. Se recomienda que se prepare y publique un informe anual, en el cual se presente la información gráficamente para describir la situación de los programas de alimentos fortificados en el país, junto con información de otras actividades de monitoreo de las autoridades sanitarias o de vigilancia."

Cote d'Ivoire Wheat

Element	Scoring (use drop-down menus)	Comments (open text)
**GENERAL**		
fit for human consumption (types/grades to be fortified)	(2) States at least one type fit for human consumption	Article 1/2 - wheat flour for bread for human consumption
2. States the public health objective; purpose and scope of legislation	(2) States the public health objective or general purpose of legislation	Article 6(9) - requirement to label use-by date if the flour is used as a nutrient and sold as such for public health reasons
3. References latest available science or accepted international norms and recommendations, particularly for items that may not be covered in the country's documents	(0) Does not state	
4. Provides definitions that includes terms that are specific to fortification (e.g. fortified food, premix, fortificant, food vehicle)	(0) Does not state	
5. Provides repeals (if there is at least one prior document about fortification)	(0) Does not state	
fortification is to begin (e.g. effective 6 months from signing)	fortification is to begin (e.g. effective 6 months from signing)	any unfortified stocks
**MICRONUTRIENTS/PREMIX**		
7. States nutrients required	(2) States nutrients	Article 1/2 - iron and folic acid
8. States fortificants (chemical compounds) to be used (including fortificants that are allowable as options)	(2) States fortificants for at least one nutrient	Article 2 - electrolytic form
9. States fortification levels	(1) States one number only	Article 2 - 60 ppm iron and 1.5 ppm folic acid
10. States consideration of bioavailability/biological activity of fortificants	(2) States some consideration of bioavailability (mentions these or related terms)	Attached letter states consideration to switch for bioavailability
11. States consideration of nutrient stability	(2) States consideration of nutrient stability	Article 6(9) - requirement to label use-by date if the flour is
**COSTING**		
12. States that the cost of fortification is regulated through cost sharing schemes (between government, industry, consumers) or tax measures [to assist industry]	(0) Does not state any consideration	
13. States consideration of the financial responsibility of monitoring and enforcing fortification (schedule of fees, budget) [on the government side]	(0) Does not state any consideration	
**LABELING**		
14. Includes some sort of statement/label/logo that makes it clear that the product is fortified	(2) Includes a statement, label, or logo	Article 6 - wording fortified with iron and folic acid
15. Provides guidance on health claims that can be made for this product (specific to micronutrients added through fortification)	(0) Does not provide	
**INTERNAL MONITORING (conducted by industry)**		
16. If samples are to be taken, describes the sampling process: for example, number of samples, amount, frequency, individual vs. composite, where samples are taken in the process, and percent considered passing	(N/A) Does not describe the sampling process	
17. States that industry is required to follow quality assurance/quality control in regards to fortification	(2) States requirement of QA/QC for fortification	Article 7 - flour manufacturers are subject to compliance control for all batches by a state-approved lab. Compliance control must verify content of iron and folic acid
18. States applicability of using qualitative testing to determine the presence or absence of a vitamin or mineral (spot tests, iChecks)	(0) Does not state	
**EXTERNAL MONITORING (conducted by government)**		
19.States requirement for external monitoring at the production site to assure compliance with standards and regulations	(0) Does not state requirement	
20. Describes protocols and systems for regulatory monitoring	(1) Does not explicitly describe, but references protocols and systems for regulatory monitoring	Having regard to decree 95-372 on national standardisation and national system for certifying compliance with standards; having regard to decree 2002-196 laying down modes of proof of compliance with standards
21. If there are two or more government agencies involved in external monitoring, clarifies the roles and responsibilities between different government agencies in external monitoring	(0) Clarifies roles and responsibilities for no agencies	
22. Allows for monitoring to be conducted often enough that problems can be identified and addressed on a timely basis; specifies a timeline for inspections (i.e. once every 6 months, increasing to once every 2 months if a discrepancy is found)	(0) Does not state	
23. If samples are to be taken, describes the sampling process: for example, number of samples, amount, frequency, individual vs. composite, where samples are taken in the process, and percent considered passing	(N/A) Does not describe the sampling process	
24. States applicability of using qualitative testing to determine the presence or absence of a vitamin or mineral (spot tests, iChecks)	(0) Does not state	
25. States registration is required in order to use a logo/be licensed to produce fortified foods	(0) Does not state that registration or licensing is required	

**COMMERCIAL MONITORING (conducted by government)**		
26. Provides justification for commercial monitoring at retail stores	(0) Does not provide justification for commercial monitoring	
27. Describes protocols and systems for commercial monitoring	(0) Does not describe	
28. If there are two or more government agencies involved in commercial monitoring, clarifies the roles and responsibilities between different government agencies in commercial monitoring	(0) Clarifies roles and responsibilities for no agencies	
29. Allows for monitoring to be conducted often enough that problems at the production site or import companies can be identified and addressed on a timely basis; specifies a timeline for inspections (i.e. once every 6 months) or works with production companies to correct noncompliance	(0) Does not state	
30. If samples are to be taken, describes the sampling process: for example, number of samples, amount, frequency, individual vs. composite, where samples are taken in the process, and percent considered passing	(N/A) Does not describe the sampling process	
**IMPORT MONITORING (conducted by government)**		
31. Provides justification for import monitoring at points of entry	(0) Does not provide justification for import monitoring	
32. Describes protocols and systems for import monitoring	(1) Does not explicitly state, but references protocols and systems for import monitoring	Article 7 - flour importers are subject to compliance control for all batches by a state-approved lab. Compliance control must verify content of iron and folic acid
33. If there are two or more government agencies involved in import monitoring, clarifies the roles and responsibilities between different government agencies in import monitoring	(0) Clarifies roles and responsibilities for no agencies	
34. If samples are to be taken, describes the sampling process: for example, number of samples, amount, frequency, individual vs. composite, where samples are taken in the process, and percent considered passing	(N/A) Does not describe the sampling process	
**ENFORCEMENT/PENALTIES**		
35. Indicates roles and responsibilities in enforcing the legislation	(0) Does not state	
36. States incentives to start fortification	(0) Does not state	
37. States incentives to continue fortification, including ensuring compliance	(0) Does not state	
38. States penalties to compel compliance	(2) States any penalties	fraud in the sale of goods and adulteration of foodstuffs
39. Penalties are objectively defined (e.g. first penalty=$100, second penalty=$300)	(0) Penalties are not objectively laid out	
40. States that enforcement is required to include feedback and support to improve performance and correct noncompliance	(0) Does not require	
**LABORATORY**		
41. References required analytical assays for nutrients (e.g. Liquid chromatography-mass spectrometry for folic acid, atomic absorption for iron and zinc.)	(0) Does not state requirements	
42. States recognition that laboratory results are subject to several sources of variation and do not provide conclusive evidence of compliance or noncompliance	(0) Does not state recognition	
43. Focuses on the quantitative analysis of "marker" micronutrients such as iron	(0) Does not state	
**REPORTING**		
44. States how government monitoring results are shared with stakeholders	(0) Does not state how results are shared	

Cuba Wheat

Element	Scoring (use drop-down menus)	Comments (open text)
**GENERAL**		
1. States that legislation applies to at least one food vehicle fit for human consumption (types/grades to be fortified)	(2) States at least one type fit for human consumption	"1.1 La presente Norma se aplica a la harina de trigo para el consumo humano, elaborada con trigo común, Triticum aestivum L. o con trigo ramificado, Triticum compactum Host., o una mezcla de los mismos, que ha sido preenvasada y está lista para la venta al consumidor o está destinada para utilizarla en la elaboración de otros productos alimenticios. La harina de trigo puede ser fortificada o no, según el apartado 3.1.2." Section 1.2 clearly defines the flours and uses that are excluded from the fortification requirement.
2. States the public health objective; purpose and scope of legislation	(0) Does not state	
3. References latest available science or accepted international norms and recommendations, particularly for items that may not be covered in the country's documents	(2) States the documents referenced	"2 Referencias normativas<br>Las siguientes normas de referencia son indispensables para la aplicación de esta norma. Para las referencias fechadas, solo es aplicable la edición citada. Para las referencias no fechadas, se aplica la última edición del documento de referencia (incluyendo todas las enmiendas)."
4. Provides definitions that includes terms that are specific to fortification (e.g. fortified food, premix, fortificant, food vehicle)	(2) States at least one term related to fortification	"3.1 Definición del producto"
5. Provides repeals (if there is at least one prior document about fortification)	(0) Does not state	
6. Provides effective date or gives grace period for when fortification is to begin (e.g. effective 6 months from signing)	(0) Does not state	
**MICRONUTRIENTS/PREMIX**		
7. States nutrients required	(2) States nutrients	Table in section 3.1.2
8. States fortificants (chemical compounds) to be used (including fortificants that are allowable as options)	(2) States fortificants for at least one nutrient	Table in section 3.1.2, e.g. "Hierro (como sulfato ferroso anhidro), mg/kg"
9. States fortification levels	(2) States a range or number with +/-	Table in section 3.1.2
10. States consideration of bioavailability/biological activity of fortificants	(0) Does not state any consideration	
11. States consideration of nutrient stability	(0) Does not state any consideration	
**COSTING**		
12. States that the cost of fortification is regulated through cost sharing schemes (between government, industry, consumers) or tax measures [to assist industry]	(0) Does not state any consideration	
13. States consideration of the financial responsibility of monitoring and enforcing fortification (schedule of fees, budget) [on the government side]	(0) Does not state any consideration	
**LABELING**		
14. Includes some sort of statement/label/logo that makes it clear that the product is fortified	(0) Does not include statement, label, or logo	Is not noted in section 9, *may* be noted in NC 108:2012 which is referenced in section 9. "9 Etiquetado<br>Deberán aplicarse las disposiciones de la NC 108:2012 cumpliendo con toda la información que debe llevar la etiqueta y donde no debe faltar:"
15. Provides guidance on health claims that can be made for this product (specific to micronutrients added through fortification)	(0) Does not provide	
**INTERNAL MONITORING (conducted by industry)**		
16. If samples are to be taken, describes the sampling process: for example, number of samples, amount, frequency, individual vs. composite, where samples are taken in the process, and percent considered passing	(1) States that samples should be taken (generally)	Section 9 (labeling) mentions sampling, the norms that should be followed for sampling and the lab methods that should be applied. However, it's not clear for what type(s) of monitoring the labeling applies. "Las harinas se muestrearan según la NC 86-02:1988 o según la ISO 24333: 2009, la humedad se<br>determinará por la NC ISO 712: 2002, las cenizas según la NC ISO 2171:2002, las proteínas por la<br>NC ISO 20483: 2009, el gluten húmedo y seco por las NC ISO 21415-1 al 4 ó NC 375: 2009, la<br>granulación por la NC 86-10 (1984) o la AOAC 965.22, la acidez de la grasa ISO 7305 (1986) o<br>AOAC 939.05, índice de caída NC 449:2006, propiedades de la masa empleando un alveógrafo<br>(NC 672: 2008) y el conteo de hongos filamentosos por la NC-ISO 7954: 2002(En revisión)."
17. States that industry is required to follow quality assurance/quality control in regards to fortification	(0) Does not state requirement	

18. States applicability of using qualitative testing to determine the presence or absence of a vitamin or mineral (spot tests, iChecks)	(1) States applicability of spot test to determine presence/absence of vitamin or mineral generally	Section 9 lists the references from AOAC of the methods that should be applied for different nutrients. The first method is the iron spot test; the rest I presume are quantitative assays for all nutrients to be added to fortified flour. "En caso de harinas fortificadas, se recomiendan los siguientes métodos de ensayo;"
**EXTERNAL MONITORING (conducted by government)**		
19.States requirement for external monitoring at the production site to assure compliance with standards and regulations	(0) Does not state requirement	
20. Describes protocols and systems for regulatory monitoring	(0) Does not describe	
21. If there are two or more government agencies involved in external monitoring, clarifies the roles and responsibilities between different government agencies in external monitoring	(0) Clarifies roles and responsibilities for no agencies	
22. Allows for monitoring to be conducted often enough that problems can be identified and addressed on a timely basis; specifies a timeline for inspections (i.e. once every 6 months, increasing to once every 2 months if a discrepancy is found)	(0) Does not state	
23. If samples are to be taken, describes the sampling process: for example, number of samples, amount, frequency, individual vs. composite, where samples are taken in the process, and percent considered passing	(1) States that samples should be taken (generally)	ISO 24333 describes sampling in extraordinary detail. For example "Table 3 — Sampling procedure to obtain the minimum mass of laboratory sample for flowing milled and other cereal products".
24. States applicability of using qualitative testing to determine the presence or absence of a vitamin or mineral (spot tests, iChecks)	(0) Does not state	
25. States registration is required in order to use a logo/be licensed to produce fortified foods	(0) Does not state that registration or licensing is required	
**COMMERCIAL MONITORING (conducted by government)**		
26. Provides justification for commercial monitoring at retail stores	(0) Does not provide justification for commercial monitoring	
27. Describes protocols and systems for commercial monitoring	(0) Does not describe	
28. If there are two or more government agencies involved in commercial monitoring, clarifies the roles and responsibilities between different government agencies in commercial monitoring	(0) Clarifies roles and responsibilities for no agencies	
29. Allows for monitoring to be conducted often enough that problems at the production site or import companies can be identified and addressed on a timely basis; specifies a timeline for inspections (i.e. once every 6 months) or works with production companies to correct noncompliance	(N/A) No commercial monitoring occurs	
30. If samples are to be taken, describes the sampling process: for example, number of samples, amount, frequency, individual vs. composite, where samples are taken in the process, and percent considered passing	(1) States that samples should be taken (generally)	Section 9 (labeling) mentions sampling, the norms that should be followed for sampling and the lab methods that should be applied. However, it's not clear for what type(s) of monitoring the labeling applies. "Las harinas se muestrearan según la NC 86-02:1988 o según la ISO 24333: 2009, la humedad se determinará por la NC ISO 712: 2002, las cenizas según la NC ISO 2171:2002, las proteínas por la NC ISO 20483: 2009, el gluten húmedo y seco por las NC ISO 21415-1 al 4 ó NC 375: 2009, la granulación por la NC 86-10 (1984) o la AOAC 965.22, la acidez de la grasa ISO 7305 (1986) o AOAC 939.05, índice de caída NC 449:2006, propiedades de la masa empleando un alveógrafo (NC 672: 2008) y el conteo de hongos filamentosos por la NC-ISO 7954: 2002(En revisión)."
**IMPORT MONITORING (conducted by government)**		
31. Provides justification for import monitoring at points of entry	(0) Does not provide justification for import monitoring	
32. Describes protocols and systems for import monitoring	(0) Does not state	
33. If there are two or more government agencies involved in import monitoring, clarifies the roles and responsibilities between different government agencies in import monitoring	(0) Clarifies roles and responsibilities for no agencies	
34. If samples are to be taken, describes the sampling process: for example, number of samples, amount, frequency, individual vs. composite, where samples are taken in the process, and percent considered passing	(N/A) Does not describe the sampling process	
**ENFORCEMENT/PENALTIES**		
35. Indicates roles and responsibilities in enforcing the legislation	(0) Does not state	
36. States incentives to start fortification	(0) Does not state	

37. States incentives to continue fortification, including ensuring compliance	(0) Does not state	
38. States penalties to compel compliance	(0) Does not state	
39. Penalties are objectively defined (e.g. first penalty=$100, second penalty=$300)	(N/A) No penalties are stated (Answered (0) to previous question)	
40. States that enforcement is required to include feedback and support to improve performance and correct noncompliance	(0) Does not require	
**LABORATORY**		
41. References required analytical assays for nutrients (e.g. Liquid chromatography-mass spectrometry for folic acid, atomic absorption for iron and zinc.)	(2) References required assays	"Las harinas se muestrearan según la NC 86-02:1988 o según la ISO 24333: 2009, la humedad se determinará por la NC ISO 712: 2002, las cenizas según la NC ISO 2171:2002, las proteínas por la NC ISO 20483: 2009, el gluten húmedo y seco por las NC ISO 21415-1 al 4 ó NC 375: 2009, la granulación por la NC 86-10 (1984) o la AOAC 965.22, la acidez de la grasa ISO 7305 (1986) o AOAC 939.05, índice de caída NC 449:2006, propiedades de la masa empleando un alveógrafo (NC 672: 2008) y el conteo de hongos filamentosos por la NC-ISO 7954: 2002(En revisión)." ...
42. States recognition that laboratory results are subject to several sources of variation and do not provide conclusive evidence of compliance or noncompliance	(0) Does not state recognition	
43. Focuses on the quantitative analysis of "marker" micronutrients such as iron	(0) Does not state	
**REPORTING**		
44. States how government monitoring results are shared with stakeholders	(0) Does not state how results are shared	

Djibouti Wheat

Element	Scoring (use drop-down menus)	Comments (open text)
**GENERAL**		
1. States that legislation applies to at least one food vehicle fit for human consumption (types/grades to be fortified)	(2) States at least one type fit for human consumption	Article 1 - all broad consumer foods that can be fortified must be fortified; Article 2 - flour produced by crushing wheat, manufactured, packaged, or imported in Djibouti must be fortified with iron-vitamins;
2. States the public health objective; purpose and scope of legislation	(2) States the public health objective or general purpose of legislation	Article 1 - with the aim of reducing the serious problems caused by micronutrient deficiencies including iron, vitamin A, and iodine among children and women.
3. References latest available science or accepted international norms and recommendations, particularly for items that may not be covered in the country's documents	(2) States the documents referenced	Having regard to decree 2011-0204 establishing the Natl Committee of Codex Alimentarius
4. Provides definitions that includes terms that are specific to fortification (e.g. fortified food, premix, fortificant, food vehicle)	(2) States at least one term related to fortification	Article 3 - fortified wheat flour
5. Provides repeals (if there is at least one prior document about fortification)	(0) Does not state	
6. Provides effective date or gives grace period for when fortification is to begin (e.g. effective 6 months from signing)	(2) States effective date or grace period for when fortification is to begin (e.g. effective 6 months from signing)	Article 15 - 6 months grace period
**MICRONUTRIENTS/PREMIX**		
7. States nutrients required	(2) States nutrients	Article 4
8. States fortificants (chemical compounds) to be used (including fortificants that are allowable as options)	(2) States fortificants for at least one nutrient	Article 4 - electrolytic iron and zinc oxide
9. States fortification levels	(1) States one number only	Article 4
10. States consideration of bioavailability/biological activity of fortificants	(0) Does not state any consideration	
11. States consideration of nutrient stability	(0) Does not state any consideration	
**COSTING**		
12. States that the cost of fortification is regulated through cost sharing schemes (between government, industry, consumers) or tax measures [to assist industry]	(0) Does not state any consideration	
13. States consideration of the financial responsibility of monitoring and enforcing fortification (schedule of fees, budget) [on the government side]	(0) Does not state any consideration	
**LABELING**		
14. Includes some sort of statement/label/logo that makes it clear that the product is fortified	(2) Includes a statement, label, or logo	Article 8 - labeling must make it clear that flour is fortified and contain the fortified flour logo
15. Provides guidance on health claims that can be made for this product (specific to micronutrients added through fortification)	(0) Does not provide	
**INTERNAL MONITORING (conducted by industry)**		
16. If samples are to be taken, describes the sampling process: for example, number of samples, amount, frequency, individual vs. composite, where samples are taken in the process, and percent considered passing	(N/A) Does not describe the sampling process	
17. States that industry is required to follow quality assurance/quality control in regards to fortification	(2) States requirement of QA/QC for fortification	Article 10 - QC must be carried out at all stages, including production
18. States applicability of using qualitative testing to determine the presence or absence of a vitamin or mineral (spot tests, iChecks)	(0) Does not state	
**EXTERNAL MONITORING (conducted by government)**		
19.States requirement for external monitoring at the production site to assure compliance with standards and regulations	(2) States requirement for external monitoring or the need for audits/inspections	Article 11 - QC dept of Ministry of Economy and Finance with the Epi Directorate and National Lab of Food Analysis shall monitor the quality and hygiene of fortified flour.
20. Describes protocols and systems for regulatory monitoring	(0) Does not describe	Article 11
21. If there are two or more government agencies involved in external monitoring, clarifies the roles and responsibilities between different government agencies in external monitoring	(0) Clarifies roles and responsibilities for no agencies	
22. Allows for monitoring to be conducted often enough that problems can be identified and addressed on a timely basis; specifies a timeline for inspections (i.e. once every 6 months, increasing to once every 2 months if a discrepancy is found)	(0) Does not state	
23. If samples are to be taken, describes the sampling process: for example, number of samples, amount, frequency, individual vs. composite, where samples are taken in the process, and percent considered passing	(N/A) Does not describe the sampling process	
24. States applicability of using qualitative testing to determine the presence or absence of a vitamin or mineral (spot tests, iChecks)	(0) Does not state	
25. States registration is required in order to use a logo/be licensed to produce fortified foods	(0) Does not state that registration or licensing is required	
**COMMERCIAL MONITORING (conducted by government)**		

26. Provides justification for commercial monitoring at retail stores	(2) Provides justification for commercial monitoring	
27. Describes protocols and systems for commercial monitoring	(0) Does not describe	
28. If there are two or more government agencies involved in commercial monitoring, clarifies the roles and responsibilities between different government agencies in commercial monitoring	(0) Clarifies roles and responsibilities for no agencies	
29. Allows for monitoring to be conducted often enough that problems at the production site or import companies can be identified and addressed on a timely basis; specifies a timeline for inspections (i.e. once every 6 months) or works with production companies to correct noncompliance	(0) Does not state	
30. If samples are to be taken, describes the sampling process: for example, number of samples, amount, frequency, individual vs. composite, where samples are taken in the process, and percent considered passing	(N/A) Does not describe the sampling process	
**IMPORT MONITORING (conducted by government)**		
31. Provides justification for import monitoring at points of entry	(2) Provides justification for import monitoring	Article 10 - QC must be carried out at all stages, including importation
32. Describes protocols and systems for import monitoring	(0) Does not state	Article 10
33. If there are two or more government agencies involved in import monitoring, clarifies the roles and responsibilities between different government agencies in import monitoring	(0) Clarifies roles and responsibilities for no agencies	
34. If samples are to be taken, describes the sampling process: for example, number of samples, amount, frequency, individual vs. composite, where samples are taken in the process, and percent considered passing	(N/A) Does not describe the sampling process	
**ENFORCEMENT/PENALTIES**		
35. Indicates roles and responsibilities in enforcing the legislation	(2) States the role and responsibilities of government in enforcement	Article 13 - government can impose penalties and seize non-fortified goods
36. States incentives to start fortification	(0) Does not state	
37. States incentives to continue fortification, including ensuring compliance	(0) Does not state	
38. States penalties to compel compliance	(2) States any penalties	Article 13 - criminal penalties, seizure and prohibition of sale, fortification at owner's expense.
39. Penalties are objectively defined (e.g. first penalty=$100, second penalty=$300)	(2) Penalties are objectively laid out in the document (e.g. first penalty=$100, second penalty=$300)	
40. States that enforcement is required to include feedback and support to improve performance and correct noncompliance	(0) Does not require	
**LABORATORY**		
41. References required analytical assays for nutrients (e.g. Liquid chromatography-mass spectrometry for folic acid, atomic absorption for iron and zinc.)	(2) References required assays	Article 12 - analytical methods used for QC are those authorised by the MoPH and MoT
42. States recognition that laboratory results are subject to several sources of variation and do not provide conclusive evidence of compliance or noncompliance	(0) Does not state recognition	
43. Focuses on the quantitative analysis of "marker" micronutrients such as iron	(0) Does not state	
**REPORTING**		
44. States how government monitoring results are shared with stakeholders	(0) Does not state how results are shared	

Dominican Republic Wheat

Element	Scoring (use drop-down menus)	Comments (open text)
**GENERAL**		
1. States that legislation applies to at least one food vehicle fit for human consumption (types/grades to be fortified)	(2) States at least one type fit for human consumption	"1.2 Campo de aplicación. El presente reglamento se aplica a la harina de trigo fortificada para el consumo humano, elaborada con trigo común, Triticum aestivum L. o con trigo ramificado, Triticum compactum Host, o una mezcla de los mismos, a granel o preenvasada y que está lista para la venta al consumidor o está destinada para utilizarla en la elaboración de otros productos alimenticios."
2. States the public health objective; purpose and scope of legislation	(0) Does not state	
3. References latest available science or accepted international norms and recommendations, particularly for items that may not be covered in the country's documents	(0) Does not state	
4. Provides definitions that includes terms that are specific to fortification (e.g. fortified food, premix, fortificant, food vehicle)	(2) States at least one term related to fortification	"3. DEFINICIONES"
5. Provides repeals (if there is at least one prior document about fortification)	(2) States repeals	"y sustituye a la NORDOM 616 Harina de trigo fortificada."
6. Provides effective date or gives grace period for when fortification is to begin (e.g. effective 6 months from signing)	(0) Does not state	
**MICRONUTRIENTS/PREMIX**		
7. States nutrients required	(2) States nutrients	"4.7.1 Los niveles mínimos de micronutrientes para la fortificación de la harina de trigo son los señalados en la tabla siguiente:"
8. States fortificants (chemical compounds) to be used (including fortificants that are allowable as options)	(2) States fortificants for at least one nutrient	"4.7.2 La fuente de hierro a utilizar en la fortificación debe ser fumarato ferroso."
9. States fortification levels	(1) States one number only	Minimum levels required. "4.7.1 Los niveles mínimos de micronutrientes para la fortificación de la harina de trigo son los señalados en la tabla siguiente:"
10. States consideration of bioavailability/biological activity of fortificants	(0) Does not state any consideration	
11. States consideration of nutrient stability	(0) Does not state any consideration	
**COSTING**		
12. States that the cost of fortification is regulated through cost sharing schemes (between government, industry, consumers) or tax measures [to assist industry]	(0) Does not state any consideration	
13. States consideration of the financial responsibility of monitoring and enforcing fortification (schedule of fees, budget) [on the government side]	(0) Does not state any consideration	
**LABELING**		
14. Includes some sort of statement/label/logo that makes it clear that the product is fortified	(2) Includes a statement, label, or logo	"5.2 Etiquetado Además de los requisitos establecidos en el Reglamento Técnico Centroamericano de Etiquetado General para Alimentos Preenvasados, se aplican las siguientes disposiciones específicas:<br>• Nombre del producto<br>• Contenido específico de Micronutrientes.<br>Nota 1: Mientras no entre en vigencia el Reglamento Técnico Centroamericano de Etiquetado General para Alimentos Preenvasados, cada país aplicará la normativa vigente en su país."
15. Provides guidance on health claims that can be made for this product (specific to micronutrients added through fortification)	(0) Does not provide	
**INTERNAL MONITORING (conducted by industry)**		
16. If samples are to be taken, describes the sampling process: for example, number of samples, amount, frequency, individual vs. composite, where samples are taken in the process, and percent considered passing	(N/A) Does not describe the sampling process	
17. States that industry is required to follow quality assurance/quality control in regards to fortification	(0) Does not state requirement	
18. States applicability of using qualitative testing to determine the presence or absence of a vitamin or mineral (spot tests, iChecks)	(0) Does not state	
**EXTERNAL MONITORING (conducted by government)**		
19.States requirement for external monitoring at the production site to assure compliance with standards and regulations	(2) States requirement for external monitoring or the need for audits/inspections	"b.1 Información General<br>Las actividades de auditoría e inspección del Sistema de Garantía de Calidad (SGC) de los programas de alimentos fortificados serán realizadas por la instancia que corresponda (DIGENOR/DIGESA), siguiendo los lineamientos establecidos en el Manual del Sistema de Garantía de Calidad de Harinas de Trigo y de Maíz fortificadas con hierro, ácido fólico y otras vitaminas del complejo B."

20. Describes protocols and systems for regulatory monitoring	(1) Does not explicitly describe, but references protocols and systems for regulatory monitoring	"b.1 Información General ...<br>Durante las visitas los inspectores deben realizar dos actividades: primero una auditoría y después, un muestreo de harina de trigo usando los formularios "Lista de Verificación para Ejercicio de Monitoreo Externo" de esa publicación anexos."
21. If there are two or more government agencies involved in external monitoring, clarifies the roles and responsibilities between different government agencies in external monitoring	(2) Clarifies roles and responsibilities for more than one agency	"La Dirección General de Normas y Sistema (DIGENOR), adscrita a la Secretaría de Estado de Industria y Comercio, está encargada de fomentar, programar y controlar el incumplimiento de la política de desarrollo industrial. En el programa de fortificaron de alimentos, DIGENOR es el organismo encargado de la creación y la debida aplicación de las Normas de Calidad y demás disposiciones emanadas de la Comisión Nacional de Normas y Sistemas de Calidad en la República Dominicana, en coordinación con la Dirección General de Salud Ambiental (DIGESA) y de la aplicación de sanciones al no cumplimiento de las normas. (Contacto: Julio Santana y Héctor Martínez, Correo Electrónico: digenor@codetel.net.do y hemojes@codetel.net.do) "
22. Allows for monitoring to be conducted often enough that problems can be identified and addressed on a timely basis; specifies a timeline for inspections (i.e. once every 6 months, increasing to once every 2 months if a discrepancy is found)	(2) Describes frequency and how it is responsive to the needs of industry or the stage of fortification implementation	"b.1 Información General ...<br>Para confirmar la ejecución de las actividades de control y aseguramiento de la calidad, los inspectores visitarán los molinos o envasadoras, en el inicio del programa, una (1) vez al mes, y cuando se considere establecido el programa, una visita cada cuatro meses."
23. If samples are to be taken, describes the sampling process: for example, number of samples, amount, frequency, individual vs. composite, where samples are taken in the process, and percent considered passing	(2) States that samples should be taken as part of external monitoring	"b.2 Muestreo y Metodología<br><br>b.2.1 Harina de Trigo<br>Para la toma de muestras se tomarán en cada molino un total de cinco muestras de 1,5 Kg. y de cada una de ellas se prepararán tres réplicas de quinientos (500) gramos en frascos o fundas de plástico. De estas, una se dejará en el molino, otra se enviará al Laboratorio Nacional de Salud Pública Dr. Defilló (LNSPDD) para análisis, y la otra para el organismo de control (DIGESA/DIGENOR) como contra muestra oficial. (La fecha de almacenamiento estará de acuerdo con la fecha de vencimiento que estipule el molino en el empaque). "
24. States applicability of using qualitative testing to determine the presence or absence of a vitamin or mineral (spot tests, iChecks)	(0) Does not state	
25. States registration is required in order to use a logo/be licensed to produce fortified foods	(0) Does not state that registration or licensing is required	
**COMMERCIAL MONITORING (conducted by government)**		
26. Provides justification for commercial monitoring at retail stores	(0) Does not provide justification for commercial monitoring	
27. Describes protocols and systems for commercial monitoring	(0) Does not describe	
28. If there are two or more government agencies involved in commercial monitoring, clarifies the roles and responsibilities between different government agencies in commercial monitoring	(0) Clarifies roles and responsibilities for no agencies	
29. Allows for monitoring to be conducted often enough that problems at the production site or import companies can be identified and addressed on a timely basis; specifies a timeline for inspections (i.e. once every 6 months) or works with production companies to correct noncompliance	(N/A) No commercial monitoring occurs	
30. If samples are to be taken, describes the sampling process: for example, number of samples, amount, frequency, individual vs. composite, where samples are taken in the process, and percent considered passing	(N/A) Does not describe the sampling process	
**IMPORT MONITORING (conducted by government)**		
31. Provides justification for import monitoring at points of entry	(0) Does not provide justification for import monitoring	
32. Describes protocols and systems for import monitoring	(0) Does not state	
33. If there are two or more government agencies involved in import monitoring, clarifies the roles and responsibilities between different government agencies in import monitoring	(0) Clarifies roles and responsibilities for no agencies	
34. If samples are to be taken, describes the sampling process: for example, number of samples, amount, frequency, individual vs. composite, where samples are taken in the process, and percent considered passing	(N/A) Does not describe the sampling process	
**ENFORCEMENT/PENALTIES**		

35. Indicates roles and responsibilities in enforcing the legislation	(2) States the role and responsibilities of government in enforcement	(Ref 1) "De La Alimentación y La Nutrición<br>Art. 39. - La Secretaría de Estado de Salud Pública y Asistencia Social (SESPAS), en coordinación con los actores relacionados con el campo de la alimentación y nutrición, participará en el diseño, implementación y evaluación de las políticas, los planes y programas correspondientes; así como en la vigilancia alimentaria y nutricional.<br>PÁRRAFO.- Para los fines del presente artículo, la SESPAS fortalecerá el Instituto Dominicano de Alimentación y Nutrición (IDAN), y en coordinación con las instituciones correspondientes reglamentará sus atribuciones, composición y funcionamiento." (Ref 2) "La vigilancia y verificación de este Reglamento Técnico le corresponde a la Secretaría de Estado de Salud Pública y Asistencia Social y a la Dirección General de Normas y Sistemas de Calidad, DIGENOR."
36. States incentives to start fortification	(0) Does not state	
37. States incentives to continue fortification, including ensuring compliance	(0) Does not state	
38. States penalties to compel compliance	(0) Does not state	
39. Penalties are objectively defined (e.g. first penalty=$100, second penalty=$300)	(N/A) No penalties are stated (Answered (0) to previous question)	
40. States that enforcement is required to include feedback and support to improve performance and correct noncompliance	(0) Does not require	
**LABORATORY**		
41. References required analytical assays for nutrients (e.g. Liquid chromatography-mass spectrometry for folic acid, atomic absorption for iron and zinc.)	(2) References required assays	(Ref 2) Lists AOAC methods for iron, niacin, folic acid, B1 and B2 analyses. "7.1 Métodos físico-químicos." (Ref 5) b.2 Muestreo y Metodología<br>b.2.1 Harina de Trigo ...<br>El análisis químico de las muestras por métodos cuantitativos será responsabilidad de LNSPDD y los resultados serán notificados a la SESPAS. El LNSPDD realizará determinaciones cuantitativas de hierro y por lo menos a una muestra de cada marca, deberá hacer la determinación de una vitamina del Complejo B (de preferencia ácido fólico).<br>Los resultados de los análisis correspondientes se enviarán al organismo de control (DIGESA/DIGENOR) y a Nutrición de la SESPAS. Los métodos de análisis serán los utilizados por LNSPDD: Determinación de hierro: (método espectrofotométrico de AOAC 944.02, 199) y Determinación de ácido fólico (Anguila G. 1996.U.S. Food and Drug Administration Methods for the Microbiological Analysis of Selected Nutrients. Association of Official Chemists, Inc; Nueva York. 86 p.)."
42. States recognition that laboratory results are subject to several sources of variation and do not provide conclusive evidence of compliance or noncompliance	(0) Does not state recognition	
43. Focuses on the quantitative analysis of "marker" micronutrients such as iron	(0) Does not state	
**REPORTING**		
44. States how government monitoring results are shared with stakeholders	(2) States how results are shared with stakeholders	Ref 5 reads like a proposal, therefore this part does, too, of reporting back to the donor. "a. Reporte de Progreso de Unidad Ejecutora del Proyecto<br>Información General<br>La Unidad Ejecutora del Proyecto recopila información acerca de la ejecución financiera y administrativa por cada componente, y una descripción de los objetivos alcanzados con la partida presupuestaria. Este reporte se hace a las más altas instancias de la SESPAS y a la oficina del Banco Mundial, en representación de GAIN, y en él se describen en detalle los logros y las actividades realizadas en el proyecto, de acuerdo a lo planificado."

Ecuador Wheat

Element	Scoring (use drop-down menus)	Comments (open text)
**GENERAL**		
1. States that legislation applies to at least one food vehicle fit for human consumption (types/grades to be fortified)	(2) States at least one type fit for human consumption	(ref 1) "Art. 1-Establecer la obligatoriedad de la fortificación y el enriquecimiento con hierro, ácido fólico y vitaminas de complejo B, de todas las harinas de trigo de producción local, importadas y donadas que se consuman o comercialicen en el país, en forma primaria o como productos elaborados." (ref 5) "2.1 Esta norma se aplica a la harina de trigo fortificada o enriquecida que se destina al consumo directo y al uso industrial, principalmente para la elaboración de pan, pastas, fideos y galletas."
2. States the public health objective; purpose and scope of legislation	(2) States the public health objective or general purpose of legislation	"Art. 2-/estas medidas contribuirán a disminuir la morbi-mortalidad de los grupos poblacionales más susceptibles como son los niños y mujeres embarazadas."
3. References latest available science or accepted international norms and recommendations, particularly for items that may not be covered in the country's documents	(0) Does not state	
4. Provides definitions that includes terms that are specific to fortification (e.g. fortified food, premix, fortificant, food vehicle)	(2) States at least one term related to fortification	"3. DEFINICIONES"
5. Provides repeals (if there is at least one prior document about fortification)	(2) States repeals	DISPOSICION FINAL.
6. Provides effective date or gives grace period for when fortification is to begin (e.g. effective 6 months from signing)	(2) States effective date or grace period for when fortification is to begin (e.g. effective 6 months from signing)	Art. 2
**MICRONUTRIENTS/PREMIX**		
7. States nutrients required	(2) States nutrients	Art. 1
8. States fortificants (chemical compounds) to be used (including fortificants that are allowable as options)	(2) States fortificants for at least one nutrient	Art. 1 states fortificants for all nutrients
9. States fortification levels	(2) States a range or number with +/-	Art. 1
10. States consideration of bioavailability/biological activity of fortificants	(0) Does not state any consideration	
11. States consideration of nutrient stability	(0) Does not state any consideration	
**COSTING**		
12. States that the cost of fortification is regulated through cost sharing schemes (between government, industry, consumers) or tax measures [to assist industry]	(2) States consideration of either cost regulation method	"DISPOSICIONES TRANSITORIAS, PRIMERA.-El Ministerio de Salud Pública entregara al inicio del programa de fortificación y por una sola vez a cada una de las industrias harineras existentes el equipo necesario, (un dosificador, un horno estatico, una balanza mecánica y una lámpara de luz ultravioleta) para realizar la premezcla. La calidad del equipo entregado será certificada por el Ministerio de Salud Pública. Para las adquisiciones futuras este Portafolio recomendará y certificará los equipos."
13. States consideration of the financial responsibility of monitoring and enforcing fortification (schedule of fees, budget) [on the government side]	(2) Shows consideration that monitoring costs money	(page 19) "• El costo de las muestras trimestrales tomadas por el área de Nutrición será asumido por la Coordinación Nacional de Nutrición (como parte del monitoreo externo). • El costo de las muestras semestrales recolectadas por los técnicos de las Unidades Sanitarias Provinciales será asumido por la empresa y los resultado forman parte de la Evaluación Posregistro Sanitario (parte del monitoreo externo)."
**LABELING**		
14. Includes some sort of statement/label/logo that makes it clear that the product is fortified	(2) Includes a statement, label, or logo	"7.3 Rotulado. Los envases deben llevar etiquetas de material que pueda ser cocido o de fácil adherencia a los mismos. Cada etiqueta llevará impresa, con características legibles e indelebles, la siguiente información: ... c) designación del producto, ejemplo: “Harina de trigo panificable extra fortificada”,
15. Provides guidance on health claims that can be made for this product (specific to micronutrients added through fortification)	(0) Does not provide	Not in ref 6.
**INTERNAL MONITORING (conducted by industry)**		
16. If samples are to be taken, describes the sampling process: for example, number of samples, amount, frequency, individual vs. composite, where samples are taken in the process, and percent considered passing	(2) States that samples should be taken as part of internal monitoring	(page 17) "El molino debe establecer mecanismos para tomar, analizar y preservar muestras de harina, registrar resultados e implementar acciones correctivas cuando se establezcan inconformidades. Pare el efecto, debe: • Realizar determinaciones cualitativas y semicuantitativas de hierro: (Anexo 4). Método de manchas para la determinación de hierro en harina de trigo), siguiendo los siguientes pasos:"

17. States that industry is required to follow quality assurance/quality control in regards to fortification	(2) States requirement of QA/QC for fortification	(page 10) "En el Ecuador, los subsistemas de monitoreo interno y externo se implementan con el propósito de asegurar y garantizar que la harina de trigo producida y comercializada en el país cumpla con las Normas de Harina de Trigo (en modificación) y con el Reglamento de Fortificación y Enriquecimiento de la Harina de Trigo en el Ecuador para Prevención de Anemias Nutricionales, emitido bajo Decreto Ejecutivo No. 4139, publicado en el Registro Oficial No. 1008, con fecha 10 de agosto de 1996, y sus modificaciones (Anexo 2). Se reconoce que los molinos o plantas procesadoras de trigo deben establecer un sistema interno de garantía y control de calidad, que la Coordinación Nacional de Nutrición debe establecer los mecanismos para implementar el sistema de monitoreo externo y el Proceso de Control y Mejoramiento en Vigilancia Sanitaria. En coordinación con las direcciones provinciales de Salud y con el Instituto de Higiene y Medicina Tropical Leopoldo Izquieta Pérez."
18. States applicability of using qualitative testing to determine the presence or absence of a vitamin or mineral (spot tests, iChecks)	(2) States applicability of spot test to determine presence/absence of vitamin or mineral specific to internal monitoring	(page 17) "El molino debe establecer mecanismos para tomar, analizar y preservar muestras de harina, registrar resultados e implementar acciones correctivas cuando se establezcan inconformidades. Pare el efecto, debe:<br>• Realizar determinaciones cualitativas y semicuantitativas de hierro: (Anexo 4). Método de manchas para la determinación de hierro en harina de trigo), siguiendo los siguientes pasos:"
**EXTERNAL MONITORING (conducted by government)**		
19.States requirement for external monitoring at the production site to assure compliance with standards and regulations	(2) States requirement for external monitoring or the need for audits/inspections	"Art. 7-El control de la calidad de la harina de trigo enriquecida estará a cargo de la Dirección General de Salud del Ministerio de Salud Pública a través del IIDES. Programa Integrado de Nutrientes, sobre la base de las normas NTE INEN correspondientes, mediante muestreo realizado periódicamente en los molinos, aduanas, distribuirdoras, bodegas de importadores, panaderías y cualquier otro lugar en el que se elabore productos con harina de trigo."
20. Describes protocols and systems for regulatory monitoring	(2) Includes checklists or provides detailed description of regulatory monitoring procedures	(page 14) "Lineamientos para la implementación del sistema de monitoreo externo del programa de fortificación de harina de trigo. (Evaluación Técnica e Inspección en fábrica)"
21. If there are two or more government agencies involved in external monitoring, clarifies the roles and responsibilities between different government agencies in external monitoring	(2) Clarifies roles and responsibilities for more than one agency	(page 15) "La evaluación técnica de la fábrica, estará a cargo de la Dirección Nacional de Vigilancia y Control Sanitario de acuerdo a lo establecido en el Reglamento de Buenas Prácticas de Manufactura de Alimentos (Decreto Ejecutivo 3253) y Acuerdo Ministerial 091<br>La inspección y análisis del proceso de fortificación están a cargo de la Coordinación Nacional de Nutrición en coordinación con las Direcciones Provinciales de Salud, estas consistirán en:<br>1. Toma de muestras harina para la determinación del contenido de los micronutrientes mediante un análisis de laboratorio.<br>2. Toma de muestras de premezcla de micronutrientes para verificar la certeza del Certificado de Análisis (CA) proporcionado por el proveedor."
22. Allows for monitoring to be conducted often enough that problems can be identified and addressed on a timely basis; specifies a timeline for inspections (i.e. once every 6 months, increasing to once every 2 months if a discrepancy is found)	(0) Does not state	Not specified in ref 3.
23. If samples are to be taken, describes the sampling process: for example, number of samples, amount, frequency, individual vs. composite, where samples are taken in the process, and percent considered passing	(2) States that samples should be taken as part of external monitoring	(page 18) "i. Toma de muestras de harina de trigo (banco de muestras)"
24. States applicability of using qualitative testing to determine the presence or absence of a vitamin or mineral (spot tests, iChecks)	(0) Does not state	
25. States registration is required in order to use a logo/be licensed to produce fortified foods	(0) Does not state that registration or licensing is required	
**COMMERCIAL MONITORING (conducted by government)**		

26. Provides justification for commercial monitoring at retail stores	(2) Provides justification for commercial monitoring	"Art. 7-El control de la calidad de la harina de trigo enriquecida estará a cargo de la Dirección General de Salud del Ministerio de Salud Pública a través del IIDES. Programa Integrado de Nutrientes, sobre la base de las normas NTE INEN correspondientes, mediante muestreo realizado periódicamente en los molinos, aduanas, distribuirdoras, bodegas de importadores, panaderías y cualquier otro lugar en el que se elabore productos con harina de trigo."
27. Describes protocols and systems for commercial monitoring	(2) Includes checklists or provides detailed description of regulatory monitoring procedures	(page 24) "Lineamientos para la implementación inspecciones técnicas para sitios de expendio (Monitoreo)"
28. If there are two or more government agencies involved in commercial monitoring, clarifies the roles and responsibilities between different government agencies in commercial monitoring	(N/A) Only one government agency involved	(page 24) States that work will be done by inspectors, but does not state the entity they work for. (page 26) "La Coordinación Nacional de Nutrición con apoyo de la Dirección Nacional de Vigilancia y Control Sanitario y las Direcciones Provinciales de Salud desarrollará un plan nacional anual de visitas de inspección técnica de fábricas, aduanas y lugares de expendio, que incluya:"
29. Allows for monitoring to be conducted often enough that problems at the production site or import companies can be identified and addressed on a timely basis; specifies a timeline for inspections (i.e. once every 6 months) or works with production companies to correct noncompliance	(1) Makes mention of a timeline	(page 24) "Basado en el número de parroquias del país o de las provincias objetivo, se planificará por lo menos una visita al año, la frecuencia e intensidad de muestreo dependerá de la densidad poblacional, la cantidad de producto consumido en la región y factores de riesgo tales como la ubicación cerca de fronteras que sean puerto de entrada de harina no fortificada, se podría tomar a las panadería como lugar de toma de muestras."
30. If samples are to be taken, describes the sampling process: for example, number of samples, amount, frequency, individual vs. composite, where samples are taken in the process, and percent considered passing	(1) States that samples should be taken (generally)	(page 25) "La determinación de micronutrientes se realizará con la toma muestras al azar basado en la NTE INEN 476, y se enviarán a analizar en laboratorios acreditados."
**IMPORT MONITORING (conducted by government)**		
31. Provides justification for import monitoring at points of entry	(2) Provides justification for import monitoring	"Art. 7-El control de la calidad de la harina de trigo enriquecida estará a cargo de la Dirección General de Salud del Ministerio de Salud Pública a través del IIDES. Programa Integrado de Nutrientes, sobre la base de las normas NTE INEN correspondientes, mediante muestreo realizado periódicamente en los molinos, aduanas, distribuirdoras, bodegas de importadores, panaderías y cualquier otro lugar en el que se elabore productos con harina de trigo."
32. Describes protocols and systems for import monitoring	(2) Includes checklists or detailed description of import monitoring procedures	(page 21) "Lineamientos para la implementación de inspección técnica en aduanas"
33. If there are two or more government agencies involved in import monitoring, clarifies the roles and responsibilities between different government agencies in import monitoring	(2) Clarifies roles and responsibilities for more than one agency	(page 21) "Lineamientos para la implementación de inspección técnica en aduanas"
34. If samples are to be taken, describes the sampling process: for example, number of samples, amount, frequency, individual vs. composite, where samples are taken in the process, and percent considered passing	(2) States that samples should be taken as part of import monitoring	(page 23) "Toma de muestras para análisis cuantitativo"
**ENFORCEMENT/PENALTIES**		
35. Indicates roles and responsibilities in enforcing the legislation	(2) States the role and responsibilities of government in enforcement	(page 12) "El Ministerio de Salud reconoce la importancia de implementar sistemas de monitoreo y evaluación de los programas de fortificación de alimentos, por lo que exhorta a la industria molinera o plantas procesadoras de trigo ha establecer un sistema interno de aseguramiento y control de la calidad del proceso de fortificación, y delega a la Coordinación Nacional de Nutrición del Ministerio de Salud Pública la responsabilidad de coordinar conjuntamente con la Dirección Nacional de Vigilancia y Control Sanitario, las Direcciones Provinciales de Salud y el Instituto Nacional de Higiene y Medicina Tropical "Leopoldo Izquieta Pérez, las acciones pertinentes para establecer un sistema ágil y sencillo de monitoreo externo de los programas que no solo permita evaluar su desempeño sino también facilitar el tramite del Registro Sanitario de las harinas."

36. States incentives to start fortification	(2) States any incentives to encourage fortification initiation (e.g. tax incentives for new equipment or premix)	"DISPOSICIONES TRANSITORIAS, PRIMERA.-El Ministerio de Salud Pública entregara al inicio del programa de fortificación y por una sola vez a cada una de las industrias harineras existentes el equipo necesario, (un dosificador, un horno estatico, una balanza mecánica y una lámpara de luz ultravioleta) para realizar la premezcla. La calidad del equipo entregado será certificada por el Ministerio de Salud Pública. Para las adquisiciones futuras este Portafolio recomendará y certificará los equipos."
37. States incentives to continue fortification, including ensuring compliance	(0) Does not state	
38. States penalties to compel compliance	(2) States any penalties	"Art. 12.-En el caso de incumplimiento de las especificaciones que se señalan en los numerales de este reglamento se procederá a sancionar de acuerdo con el Código de la Salud y demás cuerpos legales."
39. Penalties are objectively defined (e.g. first penalty=$100, second penalty=$300)	(2) Penalties are objectively laid out in the document (e.g. first penalty=$100, second penalty=$300)	"Art. 13.-En el caso de comprobarse reincidencia en el incumplimiento de los artículos de este reglamento se procederá a la clausura temporal hasta que se soluciones el problemas por un lapso no mayor de 15 días. De persistir tal reincidencia comprobada se clausurará por un nuevo período de 30 días y si se comprueba una tercera infracción se procederá a una clausura definitiva y se retirará el permiso de producción y comercialización de producto así como el Registro Sanitario correspondiente."
40. States that enforcement is required to include feedback and support to improve performance and correct noncompliance	(2) Requires any feedback/support to improve performance	(page 20) "Recepción de resultado, consolidación y retroalimentación a plantas productoras.<br>• La Coordinación Nacional de Nutrición entregará los resultados de análisis de la muestras recolectadas periódicamente a todas las empresas con el fin de realizar una La retroalimentación que permitirá reajustar sus procesos o dosificación en caso de incumplimiento o mantenerse trabajando en las mismas condiciones en caso de que todo esté dentro de los parámetros de control establecidos.<br>• La copia de los resultados entregados a Vigilancia y Control Sanitario y/o a las Direcciones Provinciales permitirá programar las auditorías o inspecciones para poder verificar los correctivos realizados por la empresa y en caso de no hacerlo establecer sanciones."
**LABORATORY**		
41. References required analytical assays for nutrients (e.g. Liquid chromatography-mass spectrometry for folic acid, atomic absorption for iron and zinc.)	(2) References required assays	Anexo 5-9: método hierro, niacina, tiamina, riboflavina, ácido fólico
42. States recognition that laboratory results are subject to several sources of variation and do not provide conclusive evidence of compliance or noncompliance	(0) Does not state recognition	
43. Focuses on the quantitative analysis of "marker" micronutrients such as iron	(2) Focuses on quantitative analysis of marker micronutrient such as iron	(page 22) "Confirmación de la presencia de micronutrientes indicadores"
**REPORTING**		
44. States how government monitoring results are shared with stakeholders	(2) States how results are shared with stakeholders	(page 26) "Difusión de resultados del sistema de monitoreo externo del programa de fortificación de harina de trigo. Los resultados de las actividades de auditoría e inspección deberían ser consolidadas tres veces al año y determinar el grado de cumplimiento de las metas de fortificación, los obstáculos a superar y las acciones a tomar. Se recomienda preparar y publicar un informe anual donde los resultados del monitoreo externo se presenten gráficamente para divulgar la situación del programa de fortificación en el país, junto con resultados de otras actividades de control de alimentos o de vigilancia tales como monitoreo comercial o vigilancia en hogares."

Egypt Wheat

Element	Scoring (use drop-down menus)	Comments (open text)
**GENERAL**		
1. States that legislation applies to at least one food vehicle fit for human consumption (types/grades to be fortified)	(0) Does not state	Therefore it has been decided to implement national flour fortification program with iron & folic acid as a long-term strategy, given that it had already proved to be the most effective means of improving iron intake in many of the developed and developing countries.
2. States the public health objective; purpose and scope of legislation	(2) States the public health objective or general purpose of legislation	Iron deficiency anemia is considered as public health problem in the Egyptian community. Different age groups are affected with the disease particularly woman of childbearing age, young children, scholar age and adolescents.
3. References latest available science or accepted international norms and recommendations, particularly for items that may not be covered in the country's documents	(0) Does not state	
4. Provides definitions that includes terms that are specific to fortification (e.g. fortified food, premix, fortificant, food vehicle)	(0) Does not state	
5. Provides repeals (if there is at least one prior document about fortification)	(N/A) No prior documents about fortification	
6. Provides effective date or gives grace period for when fortification is to begin (e.g. effective 6 months from signing)	(0) Does not state	
**MICRONUTRIENTS/PREMIX**		
7. States nutrients required	(2) States nutrients	The level of addition is 200 gm of premix added to one metric ton of flour to produce fortified flow contains 30 ppm of iron and 1.5 ppm of folic acid.
8. States fortificants (chemical compounds) to be used (including fortificants that are allowable as options)	(2) States fortificants for at least one nutrient	The premix used in EFFP is composed of:<br>1- Ferrous sulphate 42%.<br>2- Folic Acid 0.75%.<br>3- Calcium Carbonate (filler) 57.25%
9. States fortification levels	(1) States one number only	The level of addition is 200 gm of premix added to one metric ton of flour to produce fortified flow contains 30 ppm of iron and 1.5 ppm of folic acid.
10. States consideration of bioavailability/biological activity of fortificants	(0) Does not state any consideration	
11. States consideration of nutrient stability	(2) States consideration of nutrient stability	Upon receipt of the shipment, the production lot number(s) should be recorded and retained. It is recommended that a first-in, first out (FIFO) system of stock rotation be employed since the vitamins in the fortification premix have a limited shelf life in terms of their biological effectiveness and stability. Once a premix box has been opened it should be used within a few days.
**COSTING**		
12. States that the cost of fortification is regulated through cost sharing schemes (between government, industry, consumers) or tax measures [to assist industry]	(0) Does not state any consideration	
13. States consideration of the financial responsibility of monitoring and enforcing fortification (schedule of fees, budget) [on the government side]	(0) Does not state any consideration	
**LABELING**		
14. Includes some sort of statement/label/logo that makes it clear that the product is fortified	(0) Does not include statement, label, or logo	
15. Provides guidance on health claims that can be made for this product (specific to micronutrients added through fortification)	(0) Does not provide	
**INTERNAL MONITORING (conducted by industry)**		

16. If samples are to be taken, describes the sampling process: for example, number of samples, amount, frequency, individual vs. composite, where samples are taken in the process, and percent considered passing	(2) States that samples should be taken as part of internal monitoring	Quantitative tests for mills should be done on documented composite samples taken on the production line or warehouse and sent by the mill or by MOHP/MOSS Inspector. 3.1 Who is responsible for sample collection?<br>Sample collection for internal monitoring (QC):<br>1- Millers.<br>2- Laboratory technicians Composite samples are created by blending small samples of fortified flour taken at spaced time intervals across a production shift.<br>The composite sample made up of 5 to 10 spot samples should be representative of a production lot, such as an 8 hour run.<br>The samples making up the composite sample should be of the same size (200) taken evenly over the production period in order to represent total production throughout the period.<br>The purpose of the composite sample is to estimate the average value of the nutrients in a production run, an amount of 2 gm will be taken for analysis.<br><br>Also....described the qualitative testing process as part of internal monitoring at length.
17. States that industry is required to follow quality assurance/quality control in regards to fortification	(2) States requirement of QA/QC for fortification	The manual is developed to describe the steps, methods and procedures undertaken to establish a unique and efficient QC&QA system for internal and external monitoring and evaluation (M&E) of the Egyptian flour fortification program (EFFP).
18. States applicability of using qualitative testing to determine the presence or absence of a vitamin or mineral (spot tests, iChecks)	(2) States applicability of spot test to determine presence/absence of vitamin or mineral specific to internal monitoring	Qualitative tests are simple and rapid tests that can be done at the mill to determine if a flour sample has been fortified or not.<br>The primary test used for this purpose is the Iron Spot Test.<br>The iron spot test when properly done with known fortified flour samples afford a rough estimate on the level of fortification in an unknown sample. This is the most common test used by mills for quality control to assure that a correct amount of premix is being added to flour and a uniform product is being achieved.
**EXTERNAL MONITORING (conducted by government)**		
19.States requirement for external monitoring at the production site to assure compliance with standards and regulations	(2) States requirement for external monitoring or the need for audits/inspections	The manual is developed to describe the steps, methods and procedures undertaken to establish a unique and efficient QC&QA system for internal and external monitoring and evaluation (M&E) of the Egyptian flour fortification program (EFFP).
20. Describes protocols and systems for regulatory monitoring	(2) Includes checklists or provides detailed description of regulatory monitoring procedures	
21. If there are two or more government agencies involved in external monitoring, clarifies the roles and responsibilities between different government agencies in external monitoring	(2) Clarifies roles and responsibilities for more than one agency	Manual seems to be more focused on internal monitoring inspectors of Ministry of Health & Population(MOHP), and Ministry of Social Solidarities (MOSS). 3.1 Who is responsible for sample collection?<br>Sample collection for internal monitoring (QC):<br>1- Millers.<br>2- Laboratory technicians.<br>Sample collection for external monitoring (QA):<br>1- Millers.<br>2- MOSS inspectors.<br>3- MOHP inspectors.<br>4- NNI technicians.
22. Allows for monitoring to be conducted often enough that problems can be identified and addressed on a timely basis; specifies a timeline for inspections (i.e. once every 6 months, increasing to once every 2 months if a discrepancy is found)	(2) Describes frequency and how it is responsive to the needs of industry or the stage of fortification implementation	In the first operational month daily composite sample shall be collected and send to central lab of the designated governorate for quantitative analysis.<br>The composite sample shall be collected afterwards on regular interval (weekly/ monthly/ 3month) depending on need

		composite samples taken on the production line or warehouse and sent by the mill or by MOHP/MOSS Inspector. The quality inspector at the mill will be responsible for preparation of samples for quantitative analysis. Put 2 grams of flour in crucible. Place in a muffle furnace at 550 C for 6 Hours until complete ashing. Dissolve ash in dilute HCL (0.1 N) and made up to fixed volume. Determine the iron concentration using the atomic absorption spectrometer. 3.1 Who is responsible for sample collection?<br>Sample collection for internal monitoring (QC):<br>1- Millers.<br>2- Laboratory technicians.<br>Sample collection for external monitoring (QA):<br>1- Millers.<br>2- MOSS inspectors.<br>3- MOHP inspectors.<br>4- NNI technicians.<br>Composite samples are created by blending small samples of fortified flour taken at spaced time intervals across a production shift.<br>The composite sample made up of 5 to 10 spot samples should be representative of a production lot, such as an 8 hour run.<br>The samples making up the composite sample should be of the same size (200) taken evenly over the production period in order to represent total production throughout the period.<br>The purpose of the composite sample is to estimate the 3.1 Who is responsible for sample collection?<br>Sample collection for internal monitoring (QC):<br>1- Millers.<br>2- Laboratory technicians.<br>Sample collection for external monitoring (QA):<br>1- Millers.<br>2- MOSS inspectors.<br>3- MOHP inspectors.<br>4- NNI technicians.
23. If samples are to be taken, describes the sampling process: for example, number of samples, amount, frequency, individual vs. composite, where samples are taken in the process, and percent considered passing	(2) States that samples should be taken as part of external monitoring	
24. States applicability of using qualitative testing to determine the presence or absence of a vitamin or mineral (spot tests, iChecks)	(2) States applicability of spot test to determine presence/absence of vitamin or mineral specific to external monitoring	3.2 What is the type of the collected samples?<br>Simple sample of fortified flour for iron spot test (Qualitative analysis), an amount of 5 grams of flour will be taken for analysis.
25. States registration is required in order to use a logo/be licensed to produce fortified foods	(0) Does not state that registration or licensing is required	
**COMMERCIAL MONITORING (conducted by government)**		
26. Provides justification for commercial monitoring at retail stores	(0) Does not provide justification for commercial monitoring	
27. Describes protocols and systems for commercial monitoring	(1) Does not explicitly describe, but references protocols and systems for regulatory monitoring	
28. If there are two or more government agencies involved in commercial monitoring, clarifies the roles and responsibilities between different government agencies in commercial monitoring	(0) Clarifies roles and responsibilities for no agencies	
29. Allows for monitoring to be conducted often enough that problems at the production site or import companies can be identified and addressed on a timely basis; specifies a timeline for inspections (i.e. once every 6 months) or works with production companies to correct noncompliance	(0) Does not state	
30. If samples are to be taken, describes the sampling process: for example, number of samples, amount, frequency, individual vs. composite, where samples are taken in the process, and percent considered passing	(2) States that samples should be taken as part of commercial monitoring	Samples of fortified flour in the stores shall be collected from packages based on the following principles:<br>1. One random sample for each 25 packages in the store.<br>2. The collected samples shall be mixed together to form one composite random sample.<br>Samples of bread made of fortified flour shall be collected from bakeries for qualitative & quantitative analysis (NNI). Five loaves of bread shall be collected randomly for two different bakers.
**IMPORT MONITORING (conducted by government)**		
31. Provides justification for import monitoring at points of entry	(0) Does not provide justification for import monitoring	
32. Describes protocols and systems for import monitoring	(0) Does not state	

33. If there are two or more government agencies involved in import monitoring, clarifies the roles and responsibilities between different government agencies in import monitoring	(0) Clarifies roles and responsibilities for no agencies	
34. If samples are to be taken, describes the sampling process: for example, number of samples, amount, frequency, individual vs. composite, where samples are taken in the process, and percent considered passing	(N/A) Does not describe the sampling process	
**ENFORCEMENT/PENALTIES**		
35. Indicates roles and responsibilities in enforcing the legislation	(2) States the role and responsibilities of government in enforcement	inspectors of Ministry of Health & Population(MOHP), and Ministry of Social Solidarities (MOSS) The Government central lab is associated with the enforcement and regulatory function of the central government. It may do quantitative testing of flour samples collected at mills, stores, homes and markets during mill audits.
36. States incentives to start fortification	(0) Does not state	
37. States incentives to continue fortification, including ensuring compliance	(0) Does not state	
38. States penalties to compel compliance	(0) Does not state	
39. Penalties are objectively defined (e.g. first penalty=$100, second penalty=$300)	(N/A) No penalties are stated (Answered (0) to previous question)	
40. States that enforcement is required to include feedback and support to improve performance and correct noncompliance	(0) Does not require	
**LABORATORY**		
41. References required analytical assays for nutrients (e.g. Liquid chromatography-mass spectrometry for folic acid, atomic absorption for iron and zinc.)	(2) References required assays	Questions on the accuracy of the results should be referred to a certified, reference laboratory using officially approved testing procedures. It is highly recommended that any lab running quantitative tests: Use a fortified food standard with certified levels of micronutrients to correct for any bias in the results. Regularly run a fortified flour standard to assess the lab's analytical error for each assay. The quality inspector at the mill will be responsible for preparation of samples for quantitative analysis. Put 2 grams of flour in crucible. Place in a muffle furnace at 550 C for 6 Hours until complete ashing. Dissolve ash in dilute HCL (0.1 N) and made up to fixed volume. Determine the iron concentration using the atomic absorption spectrometer.
42. States recognition that laboratory results are subject to several sources of variation and do not provide conclusive evidence of compliance or noncompliance	(2) States recognition that lab results are subject to variation	There will always been some variability in the amount of iron found through the spot tests as well as through quantitative tests. Adjustment is called for if a systematic trend (i.e. consistently low or consistently high or trending low or high) over time is noticed.
43. Focuses on the quantitative analysis of "marker" micronutrients such as iron	(2) Focuses on quantitative analysis of marker micronutrient such as iron	Typically testing will be done of one of the premix ingredients as an "indicator" of the others. This method is valid method for premix that has been properly designed, manufactured and mixed to achieve a constant ratio of the different nutrients. Because the ratio is constant, measuring one of the micronutrients in flour can verify the delivery doses of the others.
**REPORTING**		
44. States how government monitoring results are shared with stakeholders	(0) Does not state how results are shared	

El Salvador Wheat

Element	Scoring (use drop-down menus)	Comments (open text)
**GENERAL**		
1. States that legislation applies to at least one food vehicle fit for human consumption (types/grades to be fortified)	(2) States at least one type fit for human consumption	"2. AMBITO DE APLICACIÓN<br>El presente reglamento se aplica a la harina de trigo fortificada para el consumo humano, elaborada con trigo común, Triticum aestivum L. o con trigo ramificado, Triticum compactum Host, o una mezcla de los mismos, a granel o preenvasada y que está lista para la venta al consumidor o esta destinada para utilizarla en la elaboración de otros productos alimenticios."
2. States the public health objective; purpose and scope of legislation	(2) States the public health objective or general purpose of legislation	"El Programa Nacional de Fortificación de Alimentos constituye una intervención prioritaria para el país, ya que se facilitan los micro nutrientes necesarios para la población salvadoreña, dicho aporte se ve reflejado en la disminución de las deficiencias por micro nutrientes especialmente en yodo y vitamina "A". Para dar respuesta a las necesidades nutricionales de la población, el Gobierno de la República, organismos internacionales y la empresa privada impulsan permanentemente la fortificación de: Sal con Yodo, Azúcar con vitamina "A", Harinas de Trigo y de Maíz Nixtamalizado con Hierro, Acido Fólico y vitaminas del complejo "B". "
3. References latest available science or accepted international norms and recommendations, particularly for items that may not be covered in the country's documents	(0) Does not state	
4. Provides definitions that includes terms that are specific to fortification (e.g. fortified food, premix, fortificant, food vehicle)	(2) States at least one term related to fortification	"3. DEFINICIONES"
5. Provides repeals (if there is at least one prior document about fortification)	(0) Does not state	Wheat flour was first fortified starting in 1995 (page 9)
6. Provides effective date or gives grace period for when fortification is to begin (e.g. effective 6 months from signing)	(0) Does not state	
**MICRONUTRIENTS/PREMIX**		
7. States nutrients required	(2) States nutrients	"4.7 Fortificación de la harina de trigo<br>4.7.1 Los niveles mínimos de micronutrientes para la fortificación de la harina de trigo son los señalados en la tabla siguiente:"
8. States fortificants (chemical compounds) to be used (including fortificants that are allowable as options)	(2) States fortificants for at least one nutrient	"4.7.2 La fuente de hierro a utilizar en la fortificación debe ser fumarato ferroso."
9. States fortification levels	(1) States one number only	"4.7 Fortificación de la harina de trigo<br>4.7.1 Los niveles mínimos de micronutrientes para la fortificación de la harina de trigo son los señalados en la tabla siguiente:"
10. States consideration of bioavailability/biological activity of fortificants	(0) Does not state any consideration	
11. States consideration of nutrient stability	(0) Does not state any consideration	
**COSTING**		
12. States that the cost of fortification is regulated through cost sharing schemes (between government, industry, consumers) or tax measures [to assist industry]	(0) Does not state any consideration	
13. States consideration of the financial responsibility of monitoring and enforcing fortification (schedule of fees, budget) [on the government side]	(0) Does not state any consideration	
**LABELING**		
14. Includes some sort of statement/label/logo that makes it clear that the product is fortified	(0) Does not include statement, label, or logo	
15. Provides guidance on health claims that can be made for this product (specific to micronutrients added through fortification)	(2) Provides guidance on health claims specific to micronutrients added through fortification	"7.1 Declaraciones Nutricionales<br>Las únicas declaraciones de propiedades nutricionales permitidas deben ser las que se refieran a energía, proteínas, carbohidratos, grasas y los componentes de las mismas, fibra, vitaminas y minerales para los cuales se hayan establecido recomendaciones nutricionales."<br>"7.4 Declaraciones de Propiedades Saludables"<br>Anexo G "3.9 Ácido fólico y defectos del conducto (tubo) neural:"
**INTERNAL MONITORING (conducted by industry)**		
16. If samples are to be taken, describes the sampling process: for example, number of samples, amount, frequency, individual vs. composite, where samples are taken in the process, and percent considered passing	(N/A) Does not describe the sampling process	
17. States that industry is required to follow quality assurance/quality control in regards to fortification	(0) Does not state requirement	

18. States applicability of using qualitative testing to determine the presence or absence of a vitamin or mineral (spot tests, iChecks)	(0) Does not state	
**EXTERNAL MONITORING (conducted by government)**		
19.States requirement for external monitoring at the production site to assure compliance with standards and regulations	(2) States requirement for external monitoring or the need for audits/inspections	(page 25) "Actividad 1: Muestreo de harina en fábricas de harina de trigo"
20. Describes protocols and systems for regulatory monitoring	(2) Includes checklists or provides detailed description of regulatory monitoring procedures	(page 25) "Actividad 1: Muestreo de harina en fábricas de harina de trigo"
21. If there are two or more government agencies involved in external monitoring, clarifies the roles and responsibilities between different government agencies in external monitoring	(2) Clarifies roles and responsibilities for more than one agency	(page 26) Example: "6. Envío de resultados de análisis fuera de norma: Los informes de resultados que se encuentren fuera de norma, son enviados por el Laboratorio de Control de Calidad de Alimentos y Aguas a la Unidad de Atención al Ambiente, en el plazo de 8 días, posteriores a la recepción de las muestras. "
22. Allows for monitoring to be conducted often enough that problems can be identified and addressed on a timely basis; specifies a timeline for inspections (i.e. once every 6 months, increasing to once every 2 months if a discrepancy is found)	(2) Describes frequency and how it is responsive to the needs of industry or the stage of fortification implementation	(page 34) "9.1 Proceso: Evaluación del programa de fortificación de alimentos<br>9.1.1 En el establecimiento local de salud<br>1. Programación de muestreo y visitas de vigilancia: Se recibe la calendarización anual por parte del SIBASI. Con ésta programación, respetando el número de muestras y fechas asignadas por la UAA, el Inspector Técnico en saneamiento ambiental o supervisor local, las incluye en la programación anual operativa." (page 37) "6. Acción a realizar: Con base a resultados obtenidos de la evaluación se debe:<br>a) Priorizar visitas de vigilancia en aquellos establecimientos productores en los cuales en base a evaluación se haya identificado una deficiente fortificación."
23. If samples are to be taken, describes the sampling process: for example, number of samples, amount, frequency, individual vs. composite, where samples are taken in the process, and percent considered passing	(2) States that samples should be taken as part of external monitoring	(page 25) "Actividad 1: Muestreo de harina en fábricas de harina de trigo"
24. States applicability of using qualitative testing to determine the presence or absence of a vitamin or mineral (spot tests, iChecks)	(0) Does not state	
25. States registration is required in order to use a logo/be licensed to produce fortified foods	(0) Does not state that registration or licensing is required	
**COMMERCIAL MONITORING (conducted by government)**		
26. Provides justification for commercial monitoring at retail stores	(2) Provides justification for commercial monitoring	(page 27) "Actividad 2: Muestreo de harina en lugares de distribución de producto nacional e importado"
27. Describes protocols and systems for commercial monitoring	(2) Includes checklists or provides detailed description of regulatory monitoring procedures	(page 27) "Actividad 2: Muestreo de harina en lugares de distribución de producto nacional e importado"
28. If there are two or more government agencies involved in commercial monitoring, clarifies the roles and responsibilities between different government agencies in commercial monitoring	(2) Clarifies roles and responsibilities for more than one agency	(page 28) Example: "6. Envío de resultados de análisis fuera de norma: Los informes de resultados que se encuentren fuera de norma, el Laboratorio de Control de Calidad de Alimentos y Aguas los envía a la Unidad de Atención al Ambiente, en el plazo de 8 días, posteriores a la recepción de la muestra. "
29. Allows for monitoring to be conducted often enough that problems at the production site or import companies can be identified and addressed on a timely basis; specifies a timeline for inspections (i.e. once every 6 months) or works with production companies to correct noncompliance	(2) Describes frequency and how it is responsive to the needs of industry or the stage of fortification implementation	(page 27) "1. Toma de Muestras: Cada SIBASI debe tomar 4 muestras mensuales o una cantidad mayor si fuese necesario a solicitud de la UAA (2 muestra importadas y 2 nacionales). Las muestras se toman en duplicado, alternando marcas y tipos<br>(suave, fuerte, semi-fuerte, otras), deben enviarse debidamente identificadas incluyendo lote de producción y país de procedencia para importadas." (page 34) "9.1 Proceso: Evaluación del programa de fortificación de alimentos<br>9.1.1 En el establecimiento local de salud<br>1. Programación de muestreo y visitas de vigilancia: Se recibe la calendarización anual por parte del SIBASI. Con ésta programación, respetando el número de muestras y fechas asignadas por la UAA, el Inspector Técnico en saneamiento ambiental o supervisor local, las incluye en la programación anual operativa." (page 37) "6. Acción a realizar: Con base a resultados obtenidos de la evaluación se debe:<br>a) Priorizar visitas de vigilancia en aquellos establecimientos productores en los cuales en base a evaluación se haya identificado una deficiente fortificación."
30. If samples are to be taken, describes the sampling process: for example, number of samples, amount, frequency, individual vs. composite, where samples are taken in the process, and percent considered passing	(2) States that samples should be taken as part of commercial monitoring	(page 27) "Actividad 2: Muestreo de harina en lugares de distribución de producto nacional e importado"
**IMPORT MONITORING (conducted by government)**		

31. Provides justification for import monitoring at points of entry	(2) Provides justification for import monitoring	(page 27) "Actividad 2: Muestreo de harina en lugares de distribución de producto nacional e importado"
32. Describes protocols and systems for import monitoring	(2) Includes checklists or detailed description of import monitoring procedures	(page 27) "Actividad 2: Muestreo de harina en lugares de distribución de producto nacional e importado"
33. If there are two or more government agencies involved in import monitoring, clarifies the roles and responsibilities between different government agencies in import monitoring	(2) Clarifies roles and responsibilities for more than one agency	(page 28) Example: "6. Envío de resultados de análisis fuera de norma: Los informes de resultados que se encuentren fuera de norma, el Laboratorio de Control de Calidad de Alimentos y Aguas los envía a la Unidad de Atención al Ambiente, en el plazo de 8 días, posteriores a la recepción de la muestra. "
34. If samples are to be taken, describes the sampling process: for example, number of samples, amount, frequency, individual vs. composite, where samples are taken in the process, and percent considered passing	(2) States that samples should be taken as part of import monitoring	(page 27) "Actividad 2: Muestreo de harina en lugares de distribución de producto nacional e importado"
**ENFORCEMENT/PENALTIES**		
35. Indicates roles and responsibilities in enforcing the legislation	(2) States the role and responsibilities of government in enforcement	"8. VIGILANCIA Y VERIFICACIÓN<br>La vigilancia y verificación de este Reglamento Técnico Centroamericano les corresponde a las Autoridades Competentes de cada país de la Región Centroamericana."
36. States incentives to start fortification	(0) Does not state	
37. States incentives to continue fortification, including ensuring compliance	(0) Does not state	
38. States penalties to compel compliance	(2) States any penalties	(page 37) "6. Acción a realizar: Con base a resultados obtenidos de la evaluación se debe:<br>...b) En aquellos casos que los establecimientos productores de alimentos fortificados sean reincidentes o no corrijan las deficiencias identificadas, se debe solicitar apoyo técnico y jurídico previo a iniciar un expediente legal administrativo, lo cual puede conllevar a la aplicación de multas, sanciones y cierres temporales o definitivos si fuere necesario."
39. Penalties are objectively defined (e.g. first penalty=$100, second penalty=$300)	(0) Penalties are not objectively laid out	
40. States that enforcement is required to include feedback and support to improve performance and correct noncompliance	(0) Does not require	
**LABORATORY**		
41. References required analytical assays for nutrients (e.g. Liquid chromatography-mass spectrometry for folic acid, atomic absorption for iron and zinc.)	(2) References required assays	"Determinación del hierro AOAC 32.1.09.17<br>Determinación de niacina AOAC 45.1.10.17<br>Determinación de ácido fólico AOAC 45.2.01.17<br>Determinación de Vitamina B1 AOAC 957.17<br>Determinación de Vitamina B2 AOAC 970.65"
42. States recognition that laboratory results are subject to several sources of variation and do not provide conclusive evidence of compliance or noncompliance	(0) Does not state recognition	
43. Focuses on the quantitative analysis of "marker" micronutrients such as iron	(0) Does not state	
**REPORTING**		
44. States how government monitoring results are shared with stakeholders	(2) States how results are shared with stakeholders	(page 39) A nivel nacional "4. Redacción de informe: El delegado de salud responsable del programa de vigilancia de la fortificación de alimentos, elabora informe y es remitido a la Dirección General de Salud."

Fiji Wheat

Element	Scoring (use drop-down menus)	Comments (open text)
**GENERAL**		
1. States that legislation applies to at least one food vehicle fit for human consumption (types/grades to be fortified)	(2) States at least one type fit for human consumption	Schedule 19.3(4) - wheat flour for human consumption by common wheat
2. States the public health objective; purpose and scope of legislation	(0) Does not state	
3. References latest available science or accepted international norms and recommendations, particularly for items that may not be covered in the country's documents	(2) States the documents referenced	Part 2, 4(1) - Codex prevails if no other standard exists
4. Provides definitions that includes terms that are specific to fortification (e.g. fortified food, premix, fortificant, food vehicle)	(0) Does not state	Part 1, 3 - food additive, nutrient content claim, nutrient comparative claim, nutrient function claim - nothing specific to fortification
5. Provides repeals (if there is at least one prior document about fortification)	(2) States repeals	Part 12, 53 - previous food law revoked
6. Provides effective date or gives grace period for when fortification is to begin (e.g. effective 6 months from signing)	(2) States effective date or grace period for when fortification is to begin (e.g. effective 6 months from signing)	Part 1, 1(2) - regs come into force the day they are published in the gazette
**MICRONUTRIENTS/PREMIX**		
7. States nutrients required	(2) States nutrients	Schedule 19.3(4) - thiamin, riboflavin, niacin, folic acid, iron, zinc
8. States fortificants (chemical compounds) to be used (including fortificants that are allowable as options)	(2) States fortificants for at least one nutrient	See above
9. States fortification levels	(1) States one number only	See above
10. States consideration of bioavailability/biological activity of fortificants	(0) Does not state any consideration	
11. States consideration of nutrient stability	(2) States consideration of nutrient stability	Schedule 19.3(3)(e) - packaged in containers to safeguard nutritional qualities
**COSTING**		
12. States that the cost of fortification is regulated through cost sharing schemes (between government, industry, consumers) or tax measures [to assist industry]	(0) Does not state any consideration	
13. States consideration of the financial responsibility of monitoring and enforcing fortification (schedule of fees, budget) [on the government side]	(2) Shows consideration that monitoring costs money	Part 10, 50 - denotes fees for re-inspection
**LABELING**		
14. Includes some sort of statement/label/logo that makes it clear that the product is fortified	(2) Includes a statement, label, or logo	Schedule 19.3(3)(ii) - labelled with "fortified" or "enriched"
15. Provides guidance on health claims that can be made for this product (specific to micronutrients added through fortification)	(2) Provides guidance on health claims specific to micronutrients added through fortification	Part 5, 22 - describes nutrient content labeling; 24 - describes prohibited claims, especially for nutrient and health claims; 25 - permitted claims (see also schedule 10); 25(7) fortified food claims
**INTERNAL MONITORING (conducted by industry)**		
16. If samples are to be taken, describes the sampling process: for example, number of samples, amount, frequency, individual vs. composite, where samples are taken in the process, and percent considered passing	(2) States that samples should be taken as part of internal monitoring	Part 2, 6(2/3) - food business operators should sample as often as needed to verify food safety and compliance with regulations. Up to 10 samples should be used to determine compliance.
17. States that industry is required to follow quality assurance/quality control in regards to fortification	(2) States requirement of QA/QC for fortification	Part 2, 6(2/1) - see above
18. States applicability of using qualitative testing to determine the presence or absence of a vitamin or mineral (spot tests, iChecks)	(0) Does not state	
**EXTERNAL MONITORING (conducted by government)**		
19.States requirement for external monitoring at the production site to assure compliance with standards and regulations	(2) States requirement for external monitoring or the need for audits/inspections	
20. Describes protocols and systems for regulatory monitoring	(1) Does not explicitly describe, but references protocols and systems for regulatory monitoring	Details on sampling, labeling samples, and analysis for various types of samples, but nothing very clear on inspections or monitoring.
21. If there are two or more government agencies involved in external monitoring, clarifies the roles and responsibilities between different government agencies in external monitoring	(1) Clarifies roles and responsibilities for one agency	Licensing agency only.
22. Allows for monitoring to be conducted often enough that problems can be identified and addressed on a timely basis; specifies a timeline for inspections (i.e. once every 6 months, increasing to once every 2 months if a discrepancy is found)	(2) Describes frequency and how it is responsive to the needs of industry or the stage of fortification implementation	Part 10, 49 - describes frequency of inspections. Schedule 28 gives planned number of annual inspections plus tightened and loosened timelines for offenders and good businesses
23. If samples are to be taken, describes the sampling process: for example, number of samples, amount, frequency, individual vs. composite, where samples are taken in the process, and percent considered passing	(2) States that samples should be taken as part of external monitoring	Part 3, 8/9 describes sampling procedure; 10 describes number of samples, 11 describes labelling samples, schedule 29 has details on number of samples based on risk and lot size - changing as per tightened and loosened schedules as above
24. States applicability of using qualitative testing to determine the presence or absence of a vitamin or mineral (spot tests, iChecks)	(0) Does not state	

25. States registration is required in order to use a logo/be licensed to produce fortified foods	(2) Describes some type of registration or licensing	Part 9, 47 - all food businesses must be licensed
**COMMERCIAL MONITORING (conducted by government)**		
26. Provides justification for commercial monitoring at retail stores	(2) Provides justification for commercial monitoring	
27. Describes protocols and systems for commercial monitoring	(1) Does not explicitly describe, but references protocols and systems for regulatory monitoring	Details on sampling/analysis. Not much else.
28. If there are two or more government agencies involved in commercial monitoring, clarifies the roles and responsibilities between different government agencies in commercial monitoring	(0) Clarifies roles and responsibilities for no agencies	
29. Allows for monitoring to be conducted often enough that problems at the production site or import companies can be identified and addressed on a timely basis; specifies a timeline for inspections (i.e. once every 6 months) or works with production companies to correct noncompliance	(0) Does not state	
30. If samples are to be taken, describes the sampling process: for example, number of samples, amount, frequency, individual vs. composite, where samples are taken in the process, and percent considered passing	(1) States that samples should be taken (generally)	Part 3, 8/9 describes sampling process
**IMPORT MONITORING (conducted by government)**		
31. Provides justification for import monitoring at points of entry	(2) Provides justification for import monitoring	Part 3, 13 - not clear whether this is something imposed on Fijian exporters, or if this is a statement that Fijian importers need to ensure exporters in other countries do...
32. Describes protocols and systems for import monitoring	(1) Does not explicitly state, but references protocols and systems for import monitoring	
33. If there are two or more government agencies involved in import monitoring, clarifies the roles and responsibilities between different government agencies in import monitoring	(0) Clarifies roles and responsibilities for no agencies	
34. If samples are to be taken, describes the sampling process: for example, number of samples, amount, frequency, individual vs. composite, where samples are taken in the process, and percent considered passing	(2) States that samples should be taken as part of import monitoring	
**ENFORCEMENT/PENALTIES**		
35. Indicates roles and responsibilities in enforcing the legislation	(0) Does not state	
36. States incentives to start fortification	(0) Does not state	
37. States incentives to continue fortification, including ensuring compliance	(0) Does not state	
38. States penalties to compel compliance	(2) States any penalties	Section 11, 51 - fines and imprisonment; 52 - publishing names of offenders
39. Penalties are objectively defined (e.g. first penalty=$100, second penalty=$300)	(2) Penalties are objectively laid out in the document (e.g. first penalty=$100, second penalty=$300)	Section 11, 51 - max fines and imprisonment sentences laid out
40. States that enforcement is required to include feedback and support to improve performance and correct noncompliance	(0) Does not require	
**LABORATORY**		
41. References required analytical assays for nutrients (e.g. Liquid chromatography-mass spectrometry for folic acid, atomic absorption for iron and zinc.)	(2) References required assays	Part 3, 12 - assays, analysis methods, and lab requirements; Fiji Gazette - iron market analytical method referenced using atomic absorp. Spec.
42. States recognition that laboratory results are subject to several sources of variation and do not provide conclusive evidence of compliance or noncompliance	(0) Does not state recognition	
43. Focuses on the quantitative analysis of "marker" micronutrients such as iron	(2) Focuses on quantitative analysis of marker micronutrient such as iron	Fiji Gazette Supplement - Iron as a market
**REPORTING**		
44. States how government monitoring results are shared with stakeholders	(0) Does not state how results are shared	

Ghana Wheat

Element	Scoring (use drop-down menus)	Comments (open text)
**GENERAL**		
1. States that legislation applies to at least one food vehicle fit for human consumption (types/grades to be fortified)	(2) States at least one type fit for human consumption	standards for soft and strong wheat
2. States the public health objective; purpose and scope of legislation	(2) States the public health objective or general purpose of legislation	Given Law no. 23/ADP of 19 May 1994 codifying public health; Given that lack of iron constitute a public health problem in BF; Given the need to combat the difficulties caused by lack of iron and to promote health of the population
3. References latest available science or accepted international norms and recommendations, particularly for items that may not be covered in the country's documents	(2) States the documents referenced	WHO, Codex guidelines
4. Provides definitions that includes terms that are specific to fortification (e.g. fortified food, premix, fortificant, food vehicle)	(2) States at least one term related to fortification	2012 Act (107.2) - fortified food definition
5. Provides repeals (if there is at least one prior document about fortification)	(2) States repeals	2012 Act (175) - repeals
6. Provides effective date or gives grace period for when fortification is to begin (e.g. effective 6 months from signing)	(2) States effective date or grace period for when fortification is to begin (e.g. effective 6 months from signing)	Ministerial Directive
**MICRONUTRIENTS/PREMIX**		
7. States nutrients required	(2) States nutrients	iron and folic acid
8. States fortificants (chemical compounds) to be used (including fortificants that are allowable as options)	(2) States fortificants for at least one nutrient	standards docs
9. States fortification levels	(2) States a range or number with +/-	standards docs - premix only has a single number
10. States consideration of bioavailability/biological activity of fortificants	(0) Does not state any consideration	
11. States consideration of nutrient stability	(2) States consideration of nutrient stability	Reg Mon - 3.4.1 Packaging for stability
**COSTING**		
12. States that the cost of fortification is regulated through cost sharing schemes (between government, industry, consumers) or tax measures [to assist industry]	(0) Does not state any consideration	
13. States consideration of the financial responsibility of monitoring and enforcing fortification (schedule of fees, budget) [on the government side]	(0) Does not state any consideration	
**LABELING**		
14. Includes some sort of statement/label/logo that makes it clear that the product is fortified	(2) Includes a statement, label, or logo	Ministerial Directive 5 - labeling must conform to the approved format by FDB
15. Provides guidance on health claims that can be made for this product (specific to micronutrients added through fortification)	(2) Provides guidance on health claims specific to micronutrients added through fortification	2012 Act (103) - deception of consumers
**INTERNAL MONITORING (conducted by industry)**		
16. If samples are to be taken, describes the sampling process: for example, number of samples, amount, frequency, individual vs. composite, where samples are taken in the process, and percent considered passing	(2) States that samples should be taken as part of internal monitoring	Reg Mon 3.3.4a and b
17. States that industry is required to follow quality assurance/quality control in regards to fortification	(2) States requirement of QA/QC for fortification	2012 Act (97.4) - requirement of GMP for registration and production
18. States applicability of using qualitative testing to determine the presence or absence of a vitamin or mineral (spot tests, iChecks)	(2) States applicability of spot test to determine presence/absence of vitamin or mineral specific to internal monitoring	Reg Mon 3.3.4a
**EXTERNAL MONITORING (conducted by government)**		
19.States requirement for external monitoring at the production site to assure compliance with standards and regulations	(2) States requirement for external monitoring or the need for audits/inspections	Article 2: Quality control is ensured throughout distribution chain by trained public service agents and/or additional individuals appointed for that purpose.
20. Describes protocols and systems for regulatory monitoring	(2) Includes checklists or provides detailed description of regulatory monitoring procedures	
21. If there are two or more government agencies involved in external monitoring, clarifies the roles and responsibilities between different government agencies in external monitoring	(N/A) Only one government agency involved	2012 Act (81) - FDA roles
22. Allows for monitoring to be conducted often enough that problems can be identified and addressed on a timely basis; specifies a timeline for inspections (i.e. once every 6 months, increasing to once every 2 months if a discrepancy is found)	(2) Describes frequency and how it is responsive to the needs of industry or the stage of fortification implementation	Reg Mon - 4.2.1
23. If samples are to be taken, describes the sampling process: for example, number of samples, amount, frequency, individual vs. composite, where samples are taken in the process, and percent considered passing	(2) States that samples should be taken as part of external monitoring	2012 Act (135.1a) - may take samples
24. States applicability of using qualitative testing to determine the presence or absence of a vitamin or mineral (spot tests, iChecks)	(1) States applicability of spot test to determine presence/absence of vitamin or mineral generally	
25. States registration is required in order to use a logo/be licensed to produce fortified foods	(2) Describes some type of registration or licensing	Ministerial Directive 3 - company must be registered with the FDB as producer/importer

**COMMERCIAL MONITORING (conducted by government)**		
26. Provides justification for commercial monitoring at retail stores	(2) Provides justification for commercial monitoring	2012 Act (107.1) - cannot distribute, trade, or sell non-fortified foods
27. Describes protocols and systems for commercial monitoring	(2) Includes checklists or provides detailed description of regulatory monitoring procedures	
28. If there are two or more government agencies involved in commercial monitoring, clarifies the roles and responsibilities between different government agencies in commercial monitoring	(2) Clarifies roles and responsibilities for more than one agency	
29. Allows for monitoring to be conducted often enough that problems at the production site or import companies can be identified and addressed on a timely basis; specifies a timeline for inspections (i.e. once every 6 months) or works with production companies to correct noncompliance	(1) Makes mention of a timeline	Reg Mon - pg 38 - sampling should be annual
30. If samples are to be taken, describes the sampling process: for example, number of samples, amount, frequency, individual vs. composite, where samples are taken in the process, and percent considered passing	(2) States that samples should be taken as part of commercial monitoring	
**IMPORT MONITORING (conducted by government)**		
31. Provides justification for import monitoring at points of entry	(2) Provides justification for import monitoring	2012 Act (99) - registration of immporters
32. Describes protocols and systems for import monitoring	(2) Includes checklists or detailed description of import monitoring procedures	2012 Act (99) - registration of importers, checklist of register items
33. If there are two or more government agencies involved in import monitoring, clarifies the roles and responsibilities between different government agencies in import monitoring	(2) Clarifies roles and responsibilities for more than one agency	FDB or CEPS
34. If samples are to be taken, describes the sampling process: for example, number of samples, amount, frequency, individual vs. composite, where samples are taken in the process, and percent considered passing	(1) States that samples should be taken (generally)	
**ENFORCEMENT/PENALTIES**		
35. Indicates roles and responsibilities in enforcing the legislation	(2) States the role and responsibilities of government in enforcement	Article 4: Minister of Commerce can…
36. States incentives to start fortification	(0) Does not state	
37. States incentives to continue fortification, including ensuring compliance	(0) Does not state	
38. States penalties to compel compliance	(2) States any penalties	Article 3: Failure to observe Article 1 constitutes an infraction punishable by administrative and criminal sanctions; Article 4: Minister of Commerce can take the following administrative measures…; 2012 Act (110) - penalties
39. Penalties are objectively defined (e.g. first penalty=$100, second penalty=$300)	(2) Penalties are objectively laid out in the document (e.g. first penalty=$100, second penalty=$300)	2012 Act (110) - fines and prison sentences
40. States that enforcement is required to include feedback and support to improve performance and correct noncompliance	(2) Requires any feedback/support to improve performance	Analytical results should go back to the mill for feedback - Reg Mon doc
**LABORATORY**		
41. References required analytical assays for nutrients (e.g. Liquid chromatography-mass spectrometry for folic acid, atomic absorption for iron and zinc.)	(2) References required assays	Analytical methods for iron and a B vitamin - Reg Mon 4.3
42. States recognition that laboratory results are subject to several sources of variation and do not provide conclusive evidence of compliance or noncompliance	(2) States recognition that lab results are subject to variation	Reg Mon - Section 1.2
43. Focuses on the quantitative analysis of "marker" micronutrients such as iron	(2) Focuses on quantitative analysis of marker micronutrient such as iron	Reg Mon - 3.3.4a
**REPORTING**		
44. States how government monitoring results are shared with stakeholders	(2) States how results are shared with stakeholders	2012 Act (138) - quarterly reports of analysts

Guatemala Wheat

Element	Scoring (use drop-down menus)	Comments (open text)
**GENERAL**		
1. States that legislation applies to at least one food vehicle fit for human consumption (types/grades to be fortified)	(2) States at least one type fit for human consumption	"2. AMBITO DE APLICACIÓN<br>El presente reglamento se aplica a la harina de trigo fortificada para el consumo humano, elaborada con trigo común, Triticum aestivum L. o con trigo ramificado, Triticum compactum Host, o una mezcla de los mismos, a granel o preenvasada y que está lista para la venta al consumidor o esta destinada para utilizarla en la elaboración de otros productos alimenticios."
2. States the public health objective; purpose and scope of legislation	(0) Does not state	
3. References latest available science or accepted international norms and recommendations, particularly for items that may not be covered in the country's documents	(0) Does not state	
4. Provides definitions that includes terms that are specific to fortification (e.g. fortified food, premix, fortificant, food vehicle)	(2) States at least one term related to fortification	"3. DEFINICIONES"
5. Provides repeals (if there is at least one prior document about fortification)	(0) Does not state	
6. Provides effective date or gives grace period for when fortification is to begin (e.g. effective 6 months from signing)	(0) Does not state	
**MICRONUTRIENTS/PREMIX**		
7. States nutrients required	(2) States nutrients	"4.7 Fortificación de la harina de trigo"
8. States fortificants (chemical compounds) to be used (including fortificants that are allowable as options)	(2) States fortificants for at least one nutrient	"4.7.2 La fuente de hierro a utilizar en la fortificación debe ser fumarato ferroso."
9. States fortification levels	(1) States one number only	4.7.1 "Nivel mínimo a alcanzar (mg/kg de harina)"
10. States consideration of bioavailability/biological activity of fortificants	(0) Does not state any consideration	
11. States consideration of nutrient stability	(0) Does not state any consideration	
**COSTING**		
12. States that the cost of fortification is regulated through cost sharing schemes (between government, industry, consumers) or tax measures [to assist industry]	(0) Does not state any consideration	
13. States consideration of the financial responsibility of monitoring and enforcing fortification (schedule of fees, budget) [on the government side]	(2) Shows consideration that monitoring costs money	"II. Procedimiento<br>a. Plan, presupuesto y calendario"
**LABELING**		
14. Includes some sort of statement/label/logo that makes it clear that the product is fortified	(0) Does not include statement, label, or logo	
15. Provides guidance on health claims that can be made for this product (specific to micronutrients added through fortification)	(2) Provides guidance on health claims specific to micronutrients added through fortification	"7.1 Declaraciones Nutricionales<br>Las únicas declaraciones de propiedades nutricionales permitidas deben ser las que se refieran a energía, proteínas, carbohidratos, grasas y los componentes de las mismas, fibra, vitaminas y minerales para los cuales se hayan establecido recomendaciones nutricionales."<br>"7.4 Declaraciones de Propiedades Saludables"<br>Anexo G "3.9 Ácido fólico y defectos del conducto (tubo) neural:"
**INTERNAL MONITORING (conducted by industry)**		
16. If samples are to be taken, describes the sampling process: for example, number of samples, amount, frequency, individual vs. composite, where samples are taken in the process, and percent considered passing	(2) States that samples should be taken as part of internal monitoring	"II. Procedimientos<br>a. Supervisión y muestreo (Personal del Departamento de Control de Calidad)"
17. States that industry is required to follow quality assurance/quality control in regards to fortification	(2) States requirement of QA/QC for fortification	(P 1) "La fortificación de harina de trigo con vitaminas y minerales requiere la implementación de actividades de aseguramiento y control de calidad para asegurar que la harina fortificada satisface los requisitos establecidos en los reglamentos y normas."
18. States applicability of using qualitative testing to determine the presence or absence of a vitamin or mineral (spot tests, iChecks)	(2) States applicability of spot test to determine presence/absence of vitamin or mineral specific to internal monitoring	"Método de mancha para la determinación de hierro adicionado a la harina de trigo fortificada."
**EXTERNAL MONITORING (conducted by government)**		
19.States requirement for external monitoring at the production site to assure compliance with standards and regulations	(2) States requirement for external monitoring or the need for audits/inspections	"Las actividades de auditoría técnica e inspección realizadas en los molinos de trigo son parte de las actividades de verificación del cumplimiento de la ley realizadas por el Estado, para asegurar que la harina de trigo satisface la calidad de nutrientes, como también las especificaciones de inocuidad establecidas en las normas y regulaciones."
20. Describes protocols and systems for regulatory monitoring	(2) Includes checklists or provides detailed description of regulatory monitoring procedures	"II. Procedimiento (Inspectores de la Autoridad Sanitaria)"

21. If there are two or more government agencies involved in external monitoring, clarifies the roles and responsibilities between different government agencies in external monitoring	(N/A) Only one government agency involved	"I. Objetivos y responsabilidad<br>El supervisor de los inspectores de la Autoridad Sanitaria es responsable de alcanzar los objetivos e informar el plan al Jefe de la Autoridad Sanitaria correspondiente."
22. Allows for monitoring to be conducted often enough that problems can be identified and addressed on a timely basis; specifies a timeline for inspections (i.e. once every 6 months, increasing to once every 2 months if a discrepancy is found)	(2) Describes frequency and how it is responsive to the needs of industry or the stage of fortification implementation	"El propósito de la planificación de las visitas de inspección es asegurar que:<br>• se designan los recursos para visitar los molinos de trigo por lo menos dos veces al año[1]. [1] Cuando se inicien las actividades de inspección las visitas deben ser más frecuentes, por lo menos 1 vez cada tres meses. Cuando se demuestre que el proceso está controlado y los resultados son satisfactorios las visitas se pueden espaciar. Debido a que esta actividad está sujeta a presupuesto anual, estos gastos se asignan en el rubro de imprevistos."
23. If samples are to be taken, describes the sampling process: for example, number of samples, amount, frequency, individual vs. composite, where samples are taken in the process, and percent considered passing	(2) States that samples should be taken as part of external monitoring	"c. Inspección<br>4. Al final de la auditoría, tome cinco4 (5) muestras de harina para la inspección por pruebas de verificación o corroboración y una muestra de la premezcla que se está utilizando en ese momento (refiérase a la sección C)."
24. States applicability of using qualitative testing to determine the presence or absence of a vitamin or mineral (spot tests, iChecks)	(2) States applicability of spot test to determine presence/absence of vitamin or mineral specific to external monitoring	"Todas las muestras de harina (incluyendo las muestras simples) contienen hierroadicionado, el cual se utiliza como micronutriente “indicador”[6]:<br>Mancha de hierro (prueba cualitativa)[7]/Presente"<br>"[7] La prueba cualitativa de hierro se usa para asegurar que todas las muestras individuales tomadas en el molino están fortificadas y no introducir sesgo al preparar muestras compuestas con muestras sin fortificación. Si una muestra no presenta hierro es señal de un problema en el proceso que se debe investigar. Los resultados de esta prueba no se utilizan para concluir y tomar acciones sobre incumplimientos en la fortificación, solamente los resultados de los análisis cuantitativos realizados en el laboratorio de la Autoridad Sanitaria o ente regulador responsable."
25. States registration is required in order to use a logo/be licensed to produce fortified foods	(0) Does not state that registration or licensing is required	
**COMMERCIAL MONITORING (conducted by government)**		
26. Provides justification for commercial monitoring at retail stores	(2) Provides justification for commercial monitoring	"La inspección de alimentos fortificados en sitios de venta o inspección comercial es la verificación del cumplimiento legal de los alimentos fortificados vendidos en los supermercados, mercados, tiendas, pulperías o colmados, sitios de venta al por mayor. También incluye la inspección en panaderías como un lugar conveniente de muestreo de los alimentos fortificados tales como sal, azúcar, harinas y aceite1. Este monitoreo permite que se detecten en el mercado marcas que no están aprobadas por el Ministerio de Salud o que no cumplen con los reglamentos locales de fortificación. También ayuda a confirmar si las marcas que se han inspeccionado previamente en efecto cumplen los requerimientos, según lo confirman los inspectores durante el proceso de monitoreo externo."
27. Describes protocols and systems for commercial monitoring	(2) Includes checklists or provides detailed description of regulatory monitoring procedures	"B. VISITAS DE INSPECCIÓN A LOS SITIOS DE VENTA AL DETALLE, AL POR MAYOR Y PANADERÍAS"
28. If there are two or more government agencies involved in commercial monitoring, clarifies the roles and responsibilities between different government agencies in commercial monitoring	(2) Clarifies roles and responsibilities for more than one agency	Not specific to Guatemala, Central America-wide recommendation:<br>"El monitoreo comercial es responsabilidad de la Autoridad Sanitaria correspondiente, en cooperación con otros entes gubernamentales a cargo de velar por el cumplimiento de estos reglamentos tales como el Departamento de Protección al Consumidor y funcionarios de gobierno de los ministerios de Industria, Comercio y Finanzas. El monitoreo a este nivel también debería involucrar a los inspectores municipales y otras autoridades locales, para que realicen visitas de inspección y tomen muestras en sus respectivas áreas. La Autoridad Sanitaria es responsable de preparar un Plan de Muestreo y proporcionar capacitación técnica para realizar las inspecciones."

29. Allows for monitoring to be conducted often enough that problems at the production site or import companies can be identified and addressed on a timely basis; specifies a timeline for inspections (i.e. once every 6 months) or works with production companies to correct noncompliance	(1) Makes mention of a timeline	This is focused on commercial monitoring only: "• Basado en el número total de distritos del país, planifique por lo menos una visita al año a cada uno de los distritos del país. La frecuencia e intensidad de muestreo depende de la densidad poblacional, la cantidad de alimento vendido en la región y factores de riesgos tales como la ubicación cerca de fronteras con otros países donde el alimento no se fortifica. Se podrían incluir las panaderías como un lugar para tomar muestras."
30. If samples are to be taken, describes the sampling process: for example, number of samples, amount, frequency, individual vs. composite, where samples are taken in the process, and percent considered passing	(2) States that samples should be taken as part of commercial monitoring	"Este manual describe los procedimientos para realizar las visitas de inspección en cualquier establecimiento que venda alimentos fortificados." "V. Procedimientos a. Visitas de los inspectores 5. Escoja un empaque sellado de aproximadamente 500 g (0.5 kg) de cada marca de alimento fortificado en el establecimiento. Si el alimento no se encuentra en esta presentación, tome la muestra de alimento en la siguiente presentación para venta al detalle. Si los envases son más pequeños, recolecte suficientes paquetes para llegar al peso especificado (Ej.: 2 empaques de 250 g). 6. Si el alimento se vende por peso o volumen de sacos más grandes, tome una muestra de aproximadamente 500 g (0.5 kg) de este producto. Asegúrese que el saco es nuevo, de otra forma no existe garantía que el producto adentro del mismo corresponde al nombre de la fábrica en la etiqueta. 7. Empaque las muestras dentro de una caja y transpórtelas a la oficina local de la Autoridad Sanitaria, donde el encargado de la misma enviará las muestras y copias de los informes a la sede de la Autoridad Sanitaria con la frecuencia que se ha determinado en el plan de trabajo anual."
**IMPORT MONITORING (conducted by government)**		
31. Provides justification for import monitoring at points of entry	(2) Provides justification for import monitoring	"I. Objetivos y Responsabilidad El propósito de evaluar los requisitos para autorizar la entrada es: • Asegurar que los productos importados cuentan con la documentación que compruebe que los reglamentos y normas nacionales que apliquen se cumplen. • Confirmar que el alimento cumple con las condiciones de fortificación basado en la presencia de uno o más micronutrientes clave."
32. Describes protocols and systems for import monitoring	(2) Includes checklists or detailed description of import monitoring procedures	"II. Procedimiento"
33. If there are two or more government agencies involved in import monitoring, clarifies the roles and responsibilities between different government agencies in import monitoring	(2) Clarifies roles and responsibilities for more than one agency	Not specific to Guatemala as this is a Central America-wide document. "Los oficiales de aduanas[1], en conjunto con la Autoridad Sanitaria[2] correspondiente responsable de autorizar el ingreso de los alimentos importados y de comprobar su calidad, deben realizar las tareas de recolectar las muestras, transportarlas adecuadamente y hacerlas llegar al laboratorio y revisar la documentación, previo a que se permita el ingreso del alimentos a la cadena de distribución dentro del país." "[1] Aunque los oficiales de aduanas no participen directamente en todas las etapas del procedimiento de inspección, tal como el muestreo, ellos deben estar informados sobre los requisitos para importación de los alimentos fortificados para poder brindar el apoyo que la Autoridad Sanitaria necesite. [2] Dependiendo del país, uno o más entes del Estado podrían estar involucrados en este proceso tales como Ministerio de Agricultura, Ministerio de Industria y Comercio, Ministerio de Salud, Organismos de Normalización y otros."
34. If samples are to be taken, describes the sampling process: for example, number of samples, amount, frequency, individual vs. composite, where samples are taken in the process, and percent considered passing	(2) States that samples should be taken as part of import monitoring	"II. Procedimiento c. Confirmación de la presencia de micronutrientes indicadores" "B. DOCUMENTACIÓN DEL CUMPLIMIENTO CON EL CONTENIDO DE MICRONUTRIENTE A TRAVÉS DEL ANÁLISIS DE LABORATORIO"
**ENFORCEMENT/PENALTIES**		

35. Indicates roles and responsibilities in enforcing the legislation	(2) States the role and responsibilities of government in enforcement	(Ref 1) Generic: "8. VIGILANCIA Y VERIFICACIÓN<br>La vigilancia y verificación de este Reglamento Técnico Centroamericano les corresponde a las Autoridades Competentes de cada país de la Región Centroamericana."
36. States incentives to start fortification	(0) Does not state	
37. States incentives to continue fortification, including ensuring compliance	(0) Does not state	
38. States penalties to compel compliance	(0) Does not state	
39. Penalties are objectively defined (e.g. first penalty=$100, second penalty=$300)	(N/A) No penalties are stated (Answered (0) to previous question)	
40. States that enforcement is required to include feedback and support to improve performance and correct noncompliance	(2) Requires any feedback/support to improve performance	Import monitoring: "I. Objetivos y responsabilidad<br>El objetivo de documentar el cumplimiento en términos del contenido de micronutriente es:<br>• Proporcionar una base para dar a los importadores recomendaciones específicas para mejorar la calidad."
**LABORATORY**		
41. References required analytical assays for nutrients (e.g. Liquid chromatography-mass spectrometry for folic acid, atomic absorption for iron and zinc.)	(2) References required assays	"7. MÉTODOS DE ANÁLISIS"<br>"Determinación del hierro AOAC 32.1.09.17<br>Determinación de niacina AOAC 45.1.10.17<br>Determinación de ácido fólico AOAC 45.2.01.17<br>Determinación de Vitamina B1 AOAC 957.17<br>Determinación de Vitamina B2 AOAC 970.65"
42. States recognition that laboratory results are subject to several sources of variation and do not provide conclusive evidence of compliance or noncompliance	(0) Does not state recognition	Just the opposite "III. Registros e informes<br>2. Cada vez que una muestra no pase los análisis cuantitativos debido a que el contenido de micronutriente es menor al mínimo legal, la Oficina de Importación debería enviar inmediatamente al importador una carta de advertencia para que tome acciones inmediatas."
43. Focuses on the quantitative analysis of "marker" micronutrients such as iron	(0) Does not state	"c. Análisis de las muestras<br>9. Al recibir las muestras, el laboratorio deberá detectar la presencia de los micronutrientes indicador4 en todas las muestras simples usando pruebas cualitativas."
**REPORTING**		
44. States how government monitoring results are shared with stakeholders	(2) States how results are shared with stakeholders	(Import monitoring) "El Jefe de la Autoridad Sanitaria presentaría este informe a la Comisión Nacional de Fortificación de Alimentos cada seis meses, indicando las fechas, marcas, cantidades y acciones tomadas con relación a la inspección de alimentos fortificados importados."

Honduras Wheat

Element	Scoring (use drop-down menus)	Comments (open text)
**GENERAL**		
1. States that legislation applies to at least one food vehicle fit for human consumption (types/grades to be fortified)	(2) States at least one type fit for human consumption	"2. AMBITO DE APLICACIÓN<br>El presente reglamento se aplica a la harina de trigo fortificada para el consumo humano, elaborada con trigo común, Triticum aestivum L. o con trigo ramificado, Triticum compactum Host, o una mezcla de los mismos, a granel o preenvasada y que está lista para la venta al consumidor o esta destinada para utilizarla en la elaboración de otros productos alimenticios."
2. States the public health objective; purpose and scope of legislation	(2) States the public health objective or general purpose of legislation	(page 1) "CONSIDERANDO: ...han demostrado que la población hondureña en general sufre de deficiencias de micronutrientes que en gran medida pueden prevenirse o evitarse con la fortificación de algunos alimentos usados como vehículos de transporte de nutrientes... Que es deber del Estado proteger la salud y el bienestar de los habitantes..."
3. References latest available science or accepted international norms and recommendations, particularly for items that may not be covered in the country's documents	(0) Does not state	
4. Provides definitions that includes terms that are specific to fortification (e.g. fortified food, premix, fortificant, food vehicle)	(2) States at least one term related to fortification	"3. DEFINICIONES"
5. Provides repeals (if there is at least one prior document about fortification)	(2) States repeals	"ARTICULO 15. DEROGATORIA"
6. Provides effective date or gives grace period for when fortification is to begin (e.g. effective 6 months from signing)	(2) States effective date or grace period for when fortification is to begin (e.g. effective 6 months from signing)	"ARTICULO 14. REGLAMENTACION" "ARTICULO 16. VIGENCIA"
**MICRONUTRIENTS/PREMIX**		
7. States nutrients required	(2) States nutrients	"4.7 Fortificación de la harina de trigo"
8. States fortificants (chemical compounds) to be used (including fortificants that are allowable as options)	(2) States fortificants for at least one nutrient	"4.7.2 La fuente de hierro a utilizar en la fortificación debe ser fumarato ferroso."
9. States fortification levels	(1) States one number only	4.7.1 "Nivel mínimo a alcanzar (mg/kg de harina)"
10. States consideration of bioavailability/biological activity of fortificants	(0) Does not state any consideration	
11. States consideration of nutrient stability	(0) Does not state any consideration	
**COSTING**		
12. States that the cost of fortification is regulated through cost sharing schemes (between government, industry, consumers) or tax measures [to assist industry]	(0) Does not state any consideration	
13. States consideration of the financial responsibility of monitoring and enforcing fortification (schedule of fees, budget) [on the government side]	(2) Shows consideration that monitoring costs money	(P 3) "ARTICULO 6. DEL ENTE REGULADOR. Corresponde al órgano regulador: ... 10) Elaborar su Prespuesto y Plan Operativo Annual y someterlo a la aprobación de la autoridad superior para que a su vez lo incluya en el Proyecto de Presupuesto de la Secretaría de Estado en el Despacho de Salud;" (P 3) "ARTICULO 12. PRESUPUESTO."
**LABELING**		
14. Includes some sort of statement/label/logo that makes it clear that the product is fortified	(0) Does not include statement, label, or logo	
15. Provides guidance on health claims that can be made for this product (specific to micronutrients added through fortification)	(2) Provides guidance on health claims specific to micronutrients added through fortification	"7.1 Declaraciones Nutricionales<br>Las únicas declaraciones de propiedades nutricionales permitidas deben ser las que se refieran a energía, proteínas, carbohidratos, grasas y los componentes de las mismas, fibra, vitaminas y minerales para los cuales se hayan establecido recomendaciones nutricionales."<br>"7.4 Declaraciones de Propiedades Saludables"<br>Anexo G "3.9 Ácido fólico y defectos del conducto (tubo) neural:"
**INTERNAL MONITORING (conducted by industry)**		
16. If samples are to be taken, describes the sampling process: for example, number of samples, amount, frequency, individual vs. composite, where samples are taken in the process, and percent considered passing	(N/A) Does not describe the sampling process	
17. States that industry is required to follow quality assurance/quality control in regards to fortification	(0) Does not state requirement	
18. States applicability of using qualitative testing to determine the presence or absence of a vitamin or mineral (spot tests, iChecks)	(0) Does not state	
**EXTERNAL MONITORING (conducted by government)**		
19.States requirement for external monitoring at the production site to assure compliance with standards and regulations	(2) States requirement for external monitoring or the need for audits/inspections	"Las actividades de auditoría técnica e inspección realizadas en los molinos de trigo son parte de las actividades de verificación del cumplimiento de la ley realizadas por el Estado, para asegurar que la harina de trigo satisface la calidad de nutrientes, como también las especificaciones de inocuidad establecidas en las normas y regulaciones."

20. Describes protocols and systems for regulatory monitoring	(2) Includes checklists or provides detailed description of regulatory monitoring procedures	"II. Procedimiento (Inspectores de la Autoridad Sanitaria)"
21. If there are two or more government agencies involved in external monitoring, clarifies the roles and responsibilities between different government agencies in external monitoring	(N/A) Only one government agency involved	"I. Objetivos y responsabilidad<br>El supervisor de los inspectores de la Autoridad Sanitaria es responsable de alcanzar los objetivos e informar el plan al Jefe de la Autoridad Sanitaria correspondiente."
22. Allows for monitoring to be conducted often enough that problems can be identified and addressed on a timely basis; specifies a timeline for inspections (i.e. once every 6 months, increasing to once every 2 months if a discrepancy is found)	(2) Describes frequency and how it is responsive to the needs of industry or the stage of fortification implementation	"El propósito de la planificación de las visitas de inspección es asegurar que:<br>• se designan los recursos para visitar los molinos de trigo por lo menos dos veces al año[1]. [1] Cuando se inicien las actividades de inspección las visitas deben ser más frecuentes, por lo menos 1 vez cada tres meses. Cuando se demuestre que el proceso está controlado y los resultados son satisfactorios las visitas se pueden espaciar. Debido a que esta actividad está sujeta a presupuesto anual, estos gastos se asignan en el rubro de imprevistos."
23. If samples are to be taken, describes the sampling process: for example, number of samples, amount, frequency, individual vs. composite, where samples are taken in the process, and percent considered passing	(2) States that samples should be taken as part of external monitoring	"c. Inspección<br>4. Al final de la auditoría, tome cinco4 (5) muestras de harina para la inspección por pruebas de verificación o corroboración y una muestra de la premezcla que se está utilizando en ese momento (refiérase a la sección C)."
24. States applicability of using qualitative testing to determine the presence or absence of a vitamin or mineral (spot tests, iChecks)	(2) States applicability of spot test to determine presence/absence of vitamin or mineral specific to external monitoring	"Todas las muestras de harina (incluyendo las muestras simples) contienen hierroadicionado, el cual se utiliza como micronutriente “indicador”[6]:<br>Mancha de hierro (prueba cualitativa) [7]/Presente"<br>"[7] La prueba cualitativa de hierro se usa para asegurar que todas las muestras individuales tomadas en el molino están fortificadas y no introducir sesgo al preparar muestras compuestas con muestras sin fortificación. Si una muestra no presenta hierro es señal de un problema en el proceso que se debe investigar. Los resultados de esta prueba no se utilizan para concluir y tomar acciones sobre incumplimientos en la fortificación, solamente los resultados de los análisis cuantitativos realizados en el laboratorio de la Autoridad Sanitaria o ente regulador responsable."
25. States registration is required in order to use a logo/be licensed to produce fortified foods	(0) Does not state that registration or licensing is required	
**COMMERCIAL MONITORING (conducted by government)**		
26. Provides justification for commercial monitoring at retail stores	(2) Provides justification for commercial monitoring	"La inspección de alimentos fortificados en sitios de venta o inspección comercial es la verificación del cumplimiento legal de los alimentos fortificados vendidos en los supermercados, mercados, tiendas, pulperías o colmados, sitios de venta al por mayor. También incluye la inspección en panaderías como un lugar conveniente de muestreo de los alimentos fortificados tales como sal, azúcar, harinas y aceite1. Este monitoreo permite que se detecten en el mercado marcas que no están aprobadas por el Ministerio de Salud o que no cumplen con los reglamentos locales de fortificación. También ayuda a confirmar si las marcas que se han inspeccionado previamente en efecto cumplen los requerimientos, según lo confirman los inspectores durante el proceso de monitoreo externo."
27. Describes protocols and systems for commercial monitoring	(2) Includes checklists or provides detailed description of regulatory monitoring procedures	"B. VISITAS DE INSPECCIÓN A LOS SITIOS DE VENTA AL DETALLE, AL POR MAYOR Y PANADERÍAS"
28. If there are two or more government agencies involved in commercial monitoring, clarifies the roles and responsibilities between different government agencies in commercial monitoring	(2) Clarifies roles and responsibilities for more than one agency	Not specific to Guatemala, Central America-wide recommendation:<br>"El monitoreo comercial es responsabilidad de la Autoridad Sanitaria correspondiente, en cooperación con otros entes gubernamentales a cargo de velar por el cumplimiento de estos reglamentos tales como el Departamento de Protección al Consumidor y funcionarios de gobierno de los ministerios de Industria, Comercio y Finanzas. El monitoreo a este nivel también debería involucrar a los inspectores municipales y otras autoridades locales, para que realicen visitas de inspección y tomen muestras en sus respectivas áreas. La Autoridad Sanitaria es responsable de preparar un Plan de Muestreo y proporcionar capacitación técnica para realizar las inspecciones."

29. Allows for monitoring to be conducted often enough that problems at the production site or import companies can be identified and addressed on a timely basis; specifies a timeline for inspections (i.e. once every 6 months) or works with production companies to correct noncompliance	(1) Makes mention of a timeline	This is focused on commercial monitoring only:<br>"• Basado en el número total de distritos del país, planifique por lo menos una visita al año a cada uno de los distritos del país. La frecuencia e intensidad de muestreo depende de la densidad poblacional, la cantidad de alimento vendido en la región y factores de riesgos tales como la ubicación cerca de fronteras con otros países donde el alimento no se fortifica. Se podrían incluir las panaderías como un lugar para tomar muestras."
30. If samples are to be taken, describes the sampling process: for example, number of samples, amount, frequency, individual vs. composite, where samples are taken in the process, and percent considered passing	(2) States that samples should be taken as part of commercial monitoring	"Este manual describe los procedimientos para realizar las visitas de inspección en cualquier establecimiento que venda alimentos fortificados."<br>"V. Procedimientos<br>a. Visitas de los inspectores<br>5. Escoja un empaque sellado de aproximadamente 500 g (0.5 kg) de cada marca de alimento fortificado en el establecimiento. Si el alimento no se encuentra en esta presentación, tome la muestra de alimento en la siguiente presentación para venta al detalle. Si los envases son más pequeños, recolecte suficientes paquetes para llegar al peso especificado (Ej.: 2 empaques de 250 g).<br>6. Si el alimento se vende por peso o volumen de sacos más grandes, tome una muestra de aproximadamente 500 g (0.5 kg) de este producto. Asegúrese que el saco es nuevo, de otra forma no existe garantía que el producto adentro del mismo corresponde al nombre de la fábrica en la etiqueta.<br>7. Empaque las muestras dentro de una caja y transpórtelas a la oficina local de la Autoridad Sanitaria, donde el encargado de la misma enviará las muestras y copias de los informes a la sede de la Autoridad Sanitaria con la frecuencia que se ha determinado en el plan de trabajo anual."
**IMPORT MONITORING (conducted by government)**		
31. Provides justification for import monitoring at points of entry	(2) Provides justification for import monitoring	"I. Objetivos y Responsabilidad<br>El propósito de evaluar los requisitos para autorizar la entrada es:<br>• Asegurar que los productos importados cuentan con la documentación que compruebe que los reglamentos y normas nacionales que apliquen se cumplen.<br>• Confirmar que el alimento cumple con las condiciones de fortificación basado en la presencia de uno o más micronutrientes clave."
32. Describes protocols and systems for import monitoring	(2) Includes checklists or detailed description of import monitoring procedures	"II. Procedimiento"
33. If there are two or more government agencies involved in import monitoring, clarifies the roles and responsibilities between different government agencies in import monitoring	(2) Clarifies roles and responsibilities for more than one agency	Not specific to Guatemala as this is a Central America-wide document. "Los oficiales de aduanas[1], en conjunto con la Autoridad Sanitaria[2] correspondiente responsable de autorizar el ingreso de los alimentos importados y de comprobar su calidad, deben realizar las tareas de recolectar las muestras, transportarlas adecuadamente y hacerlas llegar al laboratorio y revisar la documentación, previo a que se permita el ingreso del alimentos a la cadena de distribución dentro del país."<br>"[1] Aunque los oficiales de aduanas no participen directamente en todas las etapas del procedimiento de inspección, tal como el muestreo, ellos deben estar informados sobre los requisitos para importación de los alimentos fortificados para poder brindar el apoyo que la Autoridad Sanitaria necesite.<br>[2] Dependiendo del país, uno o más entes del Estado podrían estar involucrados en este proceso tales como Ministerio de Agricultura, Ministerio de Industria y Comercio, Ministerio de Salud, Organismos de Normalización y otros."
34. If samples are to be taken, describes the sampling process: for example, number of samples, amount, frequency, individual vs. composite, where samples are taken in the process, and percent considered passing	(2) States that samples should be taken as part of import monitoring	"II. Procedimiento<br>c. Confirmación de la presencia de micronutrientes indicadores"<br>"B. DOCUMENTACIÓN DEL CUMPLIMIENTO CON EL CONTENIDO DE MICRONUTRIENTE A TRAVÉS DEL ANÁLISIS DE LABORATORIO"
**ENFORCEMENT/PENALTIES**		

35. Indicates roles and responsibilities in enforcing the legislation	(2) States the role and responsibilities of government in enforcement	(P 2) "ARTICULO 3. ORGANIZACION ADMINISTRATIVA" (P 3) "ARTICULO 6. DEL ENTE REGULADOR. Corresponde al órgano regulador: …"
36. States incentives to start fortification	(2) States any incentives to encourage fortification initiation (e.g. tax incentives for new equipment or premix)	(P 3) "ARTICULO 10. INCENTIVOS FISCALES. Quedan libres del pago de impuestos de importación, tasa y sobretasas, los equipos, accesorios, respuestos e insumos que sean necesarios para la fortificación de alimentos en el marco de esta Ley."
37. States incentives to continue fortification, including ensuring compliance	(2) States any incentives to encourage the continuation of fortification (e.g. transport priority, favorable tax or tariff treatment, or patent rights)	(P 3) "ARTICULO 10. INCENTIVOS FISCALES. Quedan libres del pago de impuestos de importación, tasa y sobretasas, los equipos, accesorios, respuestos e insumos que sean necesarios para la fortificación de alimentos en el marco de esta Ley."
38. States penalties to compel compliance	(2) States any penalties	(P 3) "ARTICULO 11. SANCIONES"
39. Penalties are objectively defined (e.g. first penalty=$100, second penalty=$300)	(0) Penalties are not objectively laid out	
40. States that enforcement is required to include feedback and support to improve performance and correct noncompliance	(2) Requires any feedback/support to improve performance	Import monitoring: "I. Objetivos y responsabilidad<br>El objetivo de documentar el cumplimiento en términos del contenido de micronutriente es:<br>• Proporcionar una base para dar a los importadores recomendaciones específicas para mejorar la calidad."
**LABORATORY**		
41. References required analytical assays for nutrients (e.g. Liquid chromatography-mass spectrometry for folic acid, atomic absorption for iron and zinc.)	(2) References required assays	"7. MÉTODOS DE ANÁLISIS"<br>"Determinación del hierro AOAC 32.1.09.17<br>Determinación de niacina AOAC 45.1.10.17<br>Determinación de ácido fólico AOAC 45.2.01.17<br>Determinación de Vitamina B1 AOAC 957.17<br>Determinación de Vitamina B2 AOAC 970.65"
42. States recognition that laboratory results are subject to several sources of variation and do not provide conclusive evidence of compliance or noncompliance	(0) Does not state recognition	Just the opposite "III. Registros e informes<br>2. Cada vez que una muestra no pase los análisis cuantitativos debido a que el contenido de micronutriente es menor al mínimo legal, la Oficina de Importación debería enviar inmediatamente al importador una carta de advertencia para que tome acciones inmediatas."
43. Focuses on the quantitative analysis of "marker" micronutrients such as iron	(0) Does not state	"c. Análisis de las muestras<br>9. Al recibir las muestras, el laboratorio deberá detectar la presencia de los micronutrientes indicador4 en todas las muestras simples usando pruebas cualitativas."
**REPORTING**		
44. States how government monitoring results are shared with stakeholders	(2) States how results are shared with stakeholders	(P 2) "ARTICULO 5. DE LA AUTORIDAD SUPERIOR. Corresonde a la autoridad superior: …6) Recibir informes trimestrales del órgano ejecutor…" (P 3) "ARTICULO 6. DEL ENTE REGULADOR. Corresponde al órgano regulador: … 9) Presentar informes trimestrales, anuales y puntuales a la Autoridad Superior..."

Indonesia Wheat

Element	Scoring (use drop-down menus)	Comments (open text)
**GENERAL**		
1. States that legislation applies to at least one food vehicle fit for human consumption (types/grades to be fortified)	(2) States at least one type fit for human consumption	THE REGULATION OF THE MINISTER OF INDUSTRY ON THE OBLIGATORY APPLICATION OF THE INDONESIAN NATIONAL STANDARD (SNI) ON WHEAT FLOUR AS FOODSTUFF. Wheat Flour as foodstuff which shall further be referred to as wheat flour is the flour made from the endosperm of Triticum aestivum L (club wheat) and/or Triticum compactum Host or a mixture of both with the addition of Fe, Zn, vitamin B1, vitamin B2 and folic acid as fortification.
2. States the public health objective; purpose and scope of legislation	(2) States the public health objective or general purpose of legislation	that Wheat Flour as Food Material is widely consumed by community and is a good fortificant for satisfying community nutrition;
3. References latest available science or accepted international norms and recommendations, particularly for items that may not be covered in the country's documents	(0) Does not state	
4. Provides definitions that includes terms that are specific to fortification (e.g. fortified food, premix, fortificant, food vehicle)	(0) Does not state	Food, both processed and unprocessed, is anything that originates from biological and water sources, is designated as a food or a drink for human consumption, and includes food additives, raw food materials, and other materials used to prepare, process, and or produce foods and drinks-->Not specific to fortification
5. Provides repeals (if there is at least one prior document about fortification)	(2) States repeals	whereas in connection with the revision to the Indonesian National Standard (SNI) for Wheat Flour as Foodstuff which is an obligatory application by Regulation of the Minister of Industry Number 49/M-IND/PER/7/2008, it is necessary to rearrange the matters related to the application of SNI on Wheat Flour as Foodstuff as regulated in the Regulation of the Minister meant above;
6. Provides effective date or gives grace period for when fortification is to begin (e.g. effective 6 months from signing)	(2) States effective date or grace period for when fortification is to begin (e.g. effective 6 months from signing)	This Regulation of the Minister shall become effective 2 (two) months from the date of its enactment.
**MICRONUTRIENTS/PREMIX**		
7. States nutrients required	(2) States nutrients	Wheat Flour as foodstuff which shall further be referred to as wheat flour is the flour made from the endosperm of Triticum aestivum L (club wheat) and/or Triticum compactum Host or a mixture of both with the addition of Fe, Zn, vitamin B1, vitamin B2 and folic acid as fortification.
8. States fortificants (chemical compounds) to be used (including fortificants that are allowable as options)	(0) Does not state	Standards document does not state fortificants
9. States fortification levels	(1) States one number only	Table 1 of standards document
10. States consideration of bioavailability/biological activity of fortificants	(0) Does not state any consideration	
11. States consideration of nutrient stability	(0) Does not state any consideration	
**COSTING**		
12. States that the cost of fortification is regulated through cost sharing schemes (between government, industry, consumers) or tax measures [to assist industry]	(0) Does not state any consideration	
13. States consideration of the financial responsibility of monitoring and enforcing fortification (schedule of fees, budget) [on the government side]	(0) Does not state any consideration	
**LABELING**		
14. Includes some sort of statement/label/logo that makes it clear that the product is fortified	(2) Includes a statement, label, or logo	Certificate of Product Using the SNI Mark, hereinafter referred to as SNI Certificate, is a Certificate of Product Using the SNI Mark issued by the Product Certification Agency to a producer that is able to produce Wheat Flour as foodstuff according to the requirements of SNI.
15. Provides guidance on health claims that can be made for this product (specific to micronutrients added through fortification)	(2) Provides guidance on health claims specific to micronutrients added through fortification	Specifically for food products that make mention of nutrition claims, in order to verify the truthfulness of these, parameters of macronutrient and micronutrient contents are examined (as per the claims made).
**INTERNAL MONITORING (conducted by industry)**		
16. If samples are to be taken, describes the sampling process: for example, number of samples, amount, frequency, individual vs. composite, where samples are taken in the process, and percent considered passing	(1) States that samples should be taken (generally)	For domestic products, testing the quality of the product according to SNI in each production lot per 3 (three) months;
17. States that industry is required to follow quality assurance/quality control in regards to fortification	(0) Does not state requirement	
18. States applicability of using qualitative testing to determine the presence or absence of a vitamin or mineral (spot tests, iChecks)	(0) Does not state	
**EXTERNAL MONITORING (conducted by government)**		

19.States requirement for external monitoring at the production site to assure compliance with standards and regulations	(0) Does not state requirement	
20. Describes protocols and systems for regulatory monitoring	(0) Does not describe	
21. If there are two or more government agencies involved in external monitoring, clarifies the roles and responsibilities between different government agencies in external monitoring	(2) Clarifies roles and responsibilities for more than one agency	(2) The LSPro issuing the SNI Certificate for Wheat Flour as a Foodstuff shall be responsible for conducting a surveillance on the use of the SNI Mark from the SNI Certificate that was issued. (1) The Directorate General for Development of Industry shall guide and supervise the performance of:<br>a. the application of SNI for Wheat Flour as meant in Article 2 at the factory; and<br>b. the use of Wheat Flour as Non Food as meant in Article 4 paragraph (1) at the factory in relation to the provisions as meant in Article 4 paragraph (2)<br>(2) The supervision as meant in paragraph (1) shall be exercised at least 1 (one) time in a year by assigning a Controlling Officer.<br>(3) BPKIMI shall provide guidance to the Conformity Assessment Agency in the framework of obligatory application of SNI on Wheat Flour as Foodstuff.
22. Allows for monitoring to be conducted often enough that problems can be identified and addressed on a timely basis; specifies a timeline for inspections (i.e. once every 6 months, increasing to once every 2 months if a discrepancy is found)	(1) Makes mention of a timeline	Samplings are conducted once (one stage) in a year, i.e. during July 2013.
23. If samples are to be taken, describes the sampling process: for example, number of samples, amount, frequency, individual vs. composite, where samples are taken in the process, and percent considered passing	(1) States that samples should be taken (generally)	SNI-19-0428-1998 Instructions on Sampling
24. States applicability of using qualitative testing to determine the presence or absence of a vitamin or mineral (spot tests, iChecks)	(0) Does not state	
25. States registration is required in order to use a logo/be licensed to produce fortified foods	(2) Describes some type of registration or licensing	The Company producing or importing wheat flour as a foodstuff as meant in Article 2 is required to comply with the provisions of SNI by :<br>a. obtaining an SNI Certificate for Wheat Flour in conformity with the SNI provisions as meant in Article 2;
**COMMERCIAL MONITORING (conducted by government)**		
26. Provides justification for commercial monitoring at retail stores	(2) Provides justification for commercial monitoring	This guideline addresses sampling activities for food products as part of an oversight effort to ensure compliance in both food security and food surveillance that comprises priority food samplings, school snacks samplings, and food packaging samplings. The target of the activity is any wheat flour that is in circulation (vendors/ retailers/ agents/ distributors), that either has or has not a trade name, at the district/ city circulation level.
27. Describes protocols and systems for commercial monitoring	(1) Does not explicitly describe, but references protocols and systems for commercial monitoring	3.1.5.2.2.1. Time Frame<br>Samplings are conducted once (one stage) in a year, i.e. during July 2013.<br>3.1.5.2.2.2. Sampling<br>In taking samples of wheat flour, the following things should be taken into account:<br>- Samplings are conducted for any wheat flour trade name in circulation; samples are randomly picked from retailers/ vendors/ agents/ distributors.<br>- Samples are taken at districts/ cities periodically or as programmed by stakeholder instrumentalities on either an individual or collective basis.<br>- During each stage, samplings are carried out for 2 (two) identified districts/ cities, and for each of the districts/ cities 2 (two) sub-districts are picked in a random fashion.<br>- In each selected sub-district, 1 (one) retailer/ vendor/ agent/ distributor is identified. The sub-district's largest marketplace should be picked as the sampling location.<br>- Samplings at retailers/ vendors/ agents/ distributors are conducted as follows:<br>From each identified market retailer/ vendor/ agent/ distributor, pick at least 2 (two) packages of which combined weight equals 4kg, one pack for laboratory examination [...]<br>For a clearer description, sampling of edible wheat flour at the circulation level per stage for each trade name is as given in the following illustrative table.

28. If there are two or more government agencies involved in commercial monitoring, clarifies the roles and responsibilities between different government agencies in commercial monitoring	(2) Clarifies roles and responsibilities for more than one agency	(2) The LSPro issuing the SNI Certificate for Wheat Flour as a Foodstuff shall be responsible for conducting a surveillance on the use of the SNI Mark from the SNI Certificate that was issued. (1) The Directorate General for Development of Industry shall guide and supervise the performance of:<br>a. the application of SNI for Wheat Flour as meant in Article 2 at the factory; and<br>b. the use of Wheat Flour as Non Food as meant in Article 4 paragraph (1) at the factory in relation to the provisions as meant in Article 4 paragraph (2)<br>(2) The supervision as meant in paragraph (1) shall be exercised at least 1 (one) time in a year by assigning a Controlling Officer.<br>(3) BPKIMI shall provide guidance to the Conformity Assessment Agency in the framework of obligatory application of SNI on Wheat Flour as Foodstuff.
29. Allows for monitoring to be conducted often enough that problems at the production site or import companies can be identified and addressed on a timely basis; specifies a timeline for inspections (i.e. once every 6 months) or works with production companies to correct noncompliance	(1) Makes mention of a timeline	Samplings are conducted once (one stage) in a year, i.e. during July 2013.
30. If samples are to be taken, describes the sampling process: for example, number of samples, amount, frequency, individual vs. composite, where samples are taken in the process, and percent considered passing	(2) States that samples should be taken as part of commercial monitoring	3.1.5.2.2.1. Time Frame<br>Samplings are conducted once (one stage) in a year, i.e. during July 2013.<br>3.1.5.2.2.2. Sampling<br>In taking samples of wheat flour, the following things should be taken into account:<br>- Samplings are conducted for any wheat flour trade name in circulation; samples are randomly picked from retailers/ vendors/ agents/ distributors.<br>- Samples are taken at districts/ cities periodically or as programmed by stakeholder instrumentalities on either an individual or collective basis.<br>- During each stage, samplings are carried out for 2 (two) identified districts/ cities, and for each of the districts/ cities 2 (two) sub-districts are picked in a random fashion.<br>- In each selected sub-district, 1 (one) retailer/ vendor/ agent/ distributor is identified. The sub-district's largest marketplace should be picked as the sampling location.<br>- Samplings at retailers/ vendors/ agents/ distributors are conducted as follows:<br>From each identified market retailer/ vendor/ agent/ distributor, pick at least 2 (two) packages of which combined weight equals 4kg, one pack for laboratory examination [...]<br>For a clearer description, sampling of edible wheat flour at the circulation level per stage for each trade name is as given in the following illustrative table.
**IMPORT MONITORING (conducted by government)**		
31. Provides justification for import monitoring at points of entry	(2) Provides justification for import monitoring	(2) The import of wheat flour used for non food purposes as meant in paragraph (1) shall be subject to the following terms :<br>a. It must be imported only by a Producer Importer (PI); and<br>b. It is accompanied by a letter concerning the use of wheat flour for non food purpose issued by the Directorate General for Development of Industry. Wheat Flour as Foodstuff as meant in Article 2 and sold within the country which is domestically produced or imported shall be required to meet the provisions of SNI as meant in Article 3.

32. Describes protocols and systems for import monitoring	(1) Does not explicitly state, but references protocols and systems for import monitoring	2. For imported wheat flour, it should be accompanied by a Certificate of Analysis (CoA) document which at least contains :<br>a) the name and address of the company;<br>b) the name of the testing laboratory;<br>c) the date of testing and results of the testing that meet the parameters of SNI conducted by a laboratory that has signed an MoU with LSPro in Indonesia; and<br>d) has attached an Official Report of Taking a Sample; or<br>3. For imported Wheat Flour which does not have the Certificate of Analysis (CoA) document attached as meant in point 2, a sample will have to be taken and tested in accordance with the parameters of SNI as meant in Article 2 paragraph (1) by a testing laboratory appointed by the LSPro.
33. If there are two or more government agencies involved in import monitoring, clarifies the roles and responsibilities between different government agencies in import monitoring	(2) Clarifies roles and responsibilities for more than one agency	(2) The LSPro issuing the SNI Certificate for Wheat Flour as a Foodstuff shall be responsible for conducting a surveillance on the use of the SNI Mark from the SNI Certificate that was issued. (1) The Directorate General for Development of Industry shall guide and supervise the performance of:<br>a. the application of SNI for Wheat Flour as meant in Article 2 at the factory; and<br>b. the use of Wheat Flour as Non Food as meant in Article 4 paragraph (1) at the factory in relation to the provisions as meant in Article 4 paragraph (2)<br>(2) The supervision as meant in paragraph (1) shall be exercised at least 1 (one) time in a year by assigning a Controlling Officer.<br>(3) BPKIMI shall provide guidance to the Conformity Assessment Agency in the framework of obligatory application of SNI on Wheat Flour as Foodstuff.
34. If samples are to be taken, describes the sampling process: for example, number of samples, amount, frequency, individual vs. composite, where samples are taken in the process, and percent considered passing	(2) States that samples should be taken as part of import monitoring	For imported Wheat Flour which does not have the Certificate of Analysis (CoA) document attached as meant in point 2, a sample will have to be taken and tested in accordance with the parameters of SNI as meant in Article 2 paragraph (1) by a testing laboratory appointed by the LSPro.
**ENFORCEMENT/PENALTIES**		
35. Indicates roles and responsibilities in enforcing the legislation	(0) Does not state	
36. States incentives to start fortification	(0) Does not state	
37. States incentives to continue fortification, including ensuring compliance	(0) Does not state	
38. States penalties to compel compliance	(2) States any penalties	(1) The Wheat Flour as a Foodstuff which is imported as meant in paragraph (1) must be destroyed or exported back to the country of origin by the importer of said wheat flour.
39. Penalties are objectively defined (e.g. first penalty=$100, second penalty=$300)	(0) Penalties are not objectively laid out	
40. States that enforcement is required to include feedback and support to improve performance and correct noncompliance	(0) Does not require	
**LABORATORY**		
41. References required analytical assays for nutrients (e.g. Liquid chromatography-mass spectrometry for folic acid, atomic absorption for iron and zinc.)	(2) References required assays	The Testing Laboratory is a laboratory that examines and tests the sample of goods in accordance with the specifications/testing methods for SNI.<br>The sample is then examined for Fe, Zn, vitamin B1, vitamin B2 fortification by using methods identified in SNI No. 01-3517-2006 and its revision (Wheat Flour as a Foodstuff).
42. States recognition that laboratory results are subject to several sources of variation and do not provide conclusive evidence of compliance or noncompliance	(0) Does not state recognition	
43. Focuses on the quantitative analysis of "marker" micronutrients such as iron	(0) Does not state	
**REPORTING**		

44. States how government monitoring results are shared with stakeholders	(2) States how results are shared with stakeholders	Examination results for wheat flour that came back TMS should immediately be reported with:<br>- Chief of the National Drug and Food Examination Centre, by attaching the CP-LCP.<br>- Examination results for examination parameters included in the list of examination parameters made self-contained for the Drug and Food Supervisory Body as attached in Annex 14 need not report the CP-LCP with the chief of the National Drug and Food Examination Centre or be made subject to a validity examination at the National Drug and Food Examination Centre. Examination results can be followed through by the Drug and Food Supervisory Body and be reported with the director for Food Inspection and Certification with a courtesy copy made to the chief of the National Drug and Food Examination Centre.<br>- The director for Food Inspection and Certification. Reports of examination results for food products that came back TMS are sent as hard copies by mail and as soft copies to inspeksipangan@yahoo.com and panganppomn@gmail.com in MS Excel format as attached in Annex 9.

Kazakhstan Wheat

Element	Scoring (use drop-down menus)	Comments (open text)
**GENERAL**		
1. States that legislation applies to at least one food vehicle fit for human consumption (types/grades to be fortified)	(2) States at least one type fit for human consumption	QFS - Article 11.3 - high and first class wheat
2. States the public health objective; purpose and scope of legislation	(2) States the public health objective or general purpose of legislation	Mixture document: for the prevention of iron deficiency anemia
3. References latest available science or accepted international norms and recommendations, particularly for items that may not be covered in the country's documents	(2) States the documents referenced	State Standard - Part 4 - harmonized with CODEX
4. Provides definitions that includes terms that are specific to fortification (e.g. fortified food, premix, fortificant, food vehicle)	(2) States at least one term related to fortification	QFS - Article 1
5. Provides repeals (if there is at least one prior document about fortification)	(2) States repeals	QFS - Article 2 - international treaty rules apply in a dispute
6. Provides effective date or gives grace period for when fortification is to begin (e.g. effective 6 months from signing)	(2) States effective date or grace period for when fortification is to begin (e.g. effective 6 months from signing)	QFS - Article 22
**MICRONUTRIENTS/PREMIX**		
7. States nutrients required	(2) States nutrients	Org Standard - Part 1
8. States fortificants (chemical compounds) to be used (including fortificants that are allowable as options)	(2) States fortificants for at least one nutrient	Org Standard - Part 1
9. States fortification levels	(2) States a range or number with +/-	State Standard - Annex A
10. States consideration of bioavailability/biological activity of fortificants	(0) Does not state any consideration	
11. States consideration of nutrient stability	(2) States consideration of nutrient stability	QFS - Article 14.4
**COSTING**		
12. States that the cost of fortification is regulated through cost sharing schemes (between government, industry, consumers) or tax measures [to assist industry]	(0) Does not state any consideration	
13. States consideration of the financial responsibility of monitoring and enforcing fortification (schedule of fees, budget) [on the government side]	(0) Does not state any consideration	
**LABELING**		
14. Includes some sort of statement/label/logo that makes it clear that the product is fortified	(2) Includes a statement, label, or logo	QFS - Article 14.3.1 - food product value, macro/micro elements
15. Provides guidance on health claims that can be made for this product (specific to micronutrients added through fortification)	(2) Provides guidance on health claims specific to micronutrients added through fortification	State Standard FWF07 - 5.11.3 /(pg 11) - "Recommended by the MOH of Kaz as Healthy Food"
**INTERNAL MONITORING (conducted by industry)**		
16. If samples are to be taken, describes the sampling process: for example, number of samples, amount, frequency, individual vs. composite, where samples are taken in the process, and percent considered passing	(1) States that samples should be taken (generally)	
17. States that industry is required to follow quality assurance/quality control in regards to fortification	(2) States requirement of QA/QC for fortification	QFS - Article 18
18. States applicability of using qualitative testing to determine the presence or absence of a vitamin or mineral (spot tests, iChecks)	(0) Does not state	
**EXTERNAL MONITORING (conducted by government)**		
19.States requirement for external monitoring at the production site to assure compliance with standards and regulations	(2) States requirement for external monitoring or the need for audits/inspections	QFS - Article 9.1 - state supervision and control over quality and safety of foods
20. Describes protocols and systems for regulatory monitoring	(1) Does not explicitly describe, but references protocols and systems for regulatory monitoring	QFS - Article 12.1 - technical documentation should include…;
21. If there are two or more government agencies involved in external monitoring, clarifies the roles and responsibilities between different government agencies in external monitoring	(2) Clarifies roles and responsibilities for more than one agency	Legislative Framework - 3.2 Committee of State Sanitary… regulate monitoring of import, production, sale; 4 - MOH Committee hosting workshops, MOH,MOEDT approve checklists,
22. Allows for monitoring to be conducted often enough that problems can be identified and addressed on a timely basis; specifies a timeline for inspections (i.e. once every 6 months, increasing to once every 2 months if a discrepancy is found)	(1) Makes mention of a timeline	Framework - 3.2 - twice a year for import, production and sale
23. If samples are to be taken, describes the sampling process: for example, number of samples, amount, frequency, individual vs. composite, where samples are taken in the process, and percent considered passing	(2) States that samples should be taken as part of external monitoring	State Standard - 6.4 - medium sample with weight of less than 500 grams formed by combination of mixing single samples every 2 hours of a shift.
24. States applicability of using qualitative testing to determine the presence or absence of a vitamin or mineral (spot tests, iChecks)	(0) Does not state	
25. States registration is required in order to use a logo/be licensed to produce fortified foods	(2) Describes some type of registration or licensing	QFS - Article 7 - not specific to fortified foods, but makes mention of all foods/dietary supplements
**COMMERCIAL MONITORING (conducted by government)**		
26. Provides justification for commercial monitoring at retail stores	(2) Provides justification for commercial monitoring	QFS - Article 16.2 - no sale unless meets regulatory requirements

27. Describes protocols and systems for commercial monitoring	(0) Does not describe	
28. If there are two or more government agencies involved in commercial monitoring, clarifies the roles and responsibilities between different government agencies in commercial monitoring	(2) Clarifies roles and responsibilities for more than one agency	Legislative Framework - 3.2 Committee of State Sanitary… regulate monitoring of import, production, sale; 4 - MOH Committee hosting workshops, MOH,MOEDT approve checklists,
29. Allows for monitoring to be conducted often enough that problems at the production site or import companies can be identified and addressed on a timely basis; specifies a timeline for inspections (i.e. once every 6 months) or works with production companies to correct noncompliance	(1) Makes mention of a timeline	Framework - 3.2 - twice a year for import, production and sale
30. If samples are to be taken, describes the sampling process: for example, number of samples, amount, frequency, individual vs. composite, where samples are taken in the process, and percent considered passing	(N/A) Does not describe the sampling process	
**IMPORT MONITORING (conducted by government)**		
31. Provides justification for import monitoring at points of entry	(2) Provides justification for import monitoring	QFS - Article 13.4 - importation prohibited without complying.
32. Describes protocols and systems for import monitoring	(1) Does not explicitly state, but references protocols and systems for import monitoring	QFS - Article 17.3 - checkpoints, inspections, etc.
33. If there are two or more government agencies involved in import monitoring, clarifies the roles and responsibilities between different government agencies in import monitoring	(2) Clarifies roles and responsibilities for more than one agency	Legislative Framework - 3.2 Committee of State Sanitary… regulate monitoring of import, production, sale; 4 - MOH Committee hosting workshops, MOH,MOEDT approve checklists,
34. If samples are to be taken, describes the sampling process: for example, number of samples, amount, frequency, individual vs. composite, where samples are taken in the process, and percent considered passing	(N/A) Does not describe the sampling process	
**ENFORCEMENT/PENALTIES**		
35. Indicates roles and responsibilities in enforcing the legislation	(0) Does not state	
36. States incentives to start fortification	(0) Does not state	
37. States incentives to continue fortification, including ensuring compliance	(0) Does not state	
38. States penalties to compel compliance	(2) States any penalties	QFS - Article 8 - suspend activities
39. Penalties are objectively defined (e.g. first penalty=$100, second penalty=$300)	(0) Penalties are not objectively laid out	
40. States that enforcement is required to include feedback and support to improve performance and correct noncompliance	(0) Does not require	
**LABORATORY**		
41. References required analytical assays for nutrients (e.g. Liquid chromatography-mass spectrometry for folic acid, atomic absorption for iron and zinc.)	(2) References required assays	Org Standards - Part 5.6 - mass content of vitamins; State Standards - Part 7.17 methodologies to use
42. States recognition that laboratory results are subject to several sources of variation and do not provide conclusive evidence of compliance or noncompliance	(0) Does not state recognition	
43. Focuses on the quantitative analysis of "marker" micronutrients such as iron	(2) Focuses on quantitative analysis of marker micronutrient such as iron	State Standard - Part 6.2 - given compliance of iron, content of remaining fortificants should be deemed consistent with standards
**REPORTING**		
44. States how government monitoring results are shared with stakeholders	(0) Does not state how results are shared	

Kenya Wheat

Element	Scoring (use drop-down menus)	Comments (open text)
**GENERAL**		
1. States that legislation applies to at least one food vehicle fit for human consumption (types/grades to be fortified)	(2) States at least one type fit for human consumption	DEAS 767 - 1 Scope - wheat flour from common wheat, club wheat, or a mix for human consumption
2. States the public health objective; purpose and scope of legislation	(2) States the public health objective or general purpose of legislation	DEAS 767 - Introduction - recognition that high levels of malnutrition in the region
3. References latest available science or accepted international norms and recommendations, particularly for items that may not be covered in the country's documents	(2) States the documents referenced	During the preparation of this standard, reference was made to the following documents… 319. Where no specifications are set out in any part of these regulations for the fortification of any food articles, but specifications have been established by the joint FAO/WHO Codex Alimentarius Commission, the specifications of the Codex Alimentarius Commission shall apply.
4. Provides definitions that includes terms that are specific to fortification (e.g. fortified food, premix, fortificant, food vehicle)	(2) States at least one term related to fortification	Section 3 Definitions
5. Provides repeals (if there is at least one prior document about fortification)	(2) States repeals	DEAS 767 - Within 6 months of a declaration of an EA Standard, Partner States shall adopt the approved text without deviation and withdraw any existing national standard
6. Provides effective date or gives grace period for when fortification is to begin (e.g. effective 6 months from signing)	(2) States effective date or grace period for when fortification is to begin (e.g. effective 6 months from signing)	DEAS 767 - Within 6 months of a declaration of an EA Standard, Partner States shall adopt the approved text without deviation and withdraw any existing national standard
**MICRONUTRIENTS/PREMIX**		
7. States nutrients required	(2) States nutrients	Table 3
8. States fortificants (chemical compounds) to be used (including fortificants that are allowable as options)	(2) States fortificants for at least one nutrient	Table 3
9. States fortification levels	(2) States a range or number with +/-	Gives range around specified value
10. States consideration of bioavailability/biological activity of fortificants	(0) Does not state any consideration	
11. States consideration of nutrient stability	(2) States consideration of nutrient stability	6.3 .4 The supplier of the premix should provide an accompaning stability data for the fortificants and premixes.
**COSTING**		
12. States that the cost of fortification is regulated through cost sharing schemes (between government, industry, consumers) or tax measures [to assist industry]	(0) Does not state any consideration	
13. States consideration of the financial responsibility of monitoring and enforcing fortification (schedule of fees, budget) [on the government side]	(0) Does not state any consideration	
**LABELING**		
14. Includes some sort of statement/label/logo that makes it clear that the product is fortified	(2) Includes a statement, label, or logo	10.1.xi
15. Provides guidance on health claims that can be made for this product (specific to micronutrients added through fortification)	(2) Provides guidance on health claims specific to micronutrients added through fortification	Section 10.2
**INTERNAL MONITORING (conducted by industry)**		
16. If samples are to be taken, describes the sampling process: for example, number of samples, amount, frequency, individual vs. composite, where samples are taken in the process, and percent considered passing	(2) States that samples should be taken as part of internal monitoring	Take 500 g samples of flour every hour, mix well, and check using the iron-spot test (see Section D) that the micronutrient premix is being delivered.
17. States that industry is required to follow quality assurance/quality control in regards to fortification	(2) States requirement of QA/QC for fortification	This guideline describes the steps to be carried out to assure quality of wheat flour fortified with vitamins and minerals. In general,<br>they cover the receipt and inspection of the premix, the wheat flour fortification process, and quality control of the fortified wheat<br>flour.
18. States applicability of using qualitative testing to determine the presence or absence of a vitamin or mineral (spot tests, iChecks)	(2) States applicability of spot test to determine presence/absence of vitamin or mineral specific to internal monitoring	The guideline also includes a spot-test method to determine iron in wheat flour, since it has been used as the micronutrient indicator for timely verification that the micronutrient premix is being incorporated to the flour in the mill.
**EXTERNAL MONITORING (conducted by government)**		
19.States requirement for external monitoring at the production site to assure compliance with standards and regulations	(2) States requirement for external monitoring or the need for audits/inspections	National health authorities visit the wheat mills throughout the year to carry out technical audits and inspection of the fortification process and product.
20. Describes protocols and systems for regulatory monitoring	(1) Does not explicitly describe, but references protocols and systems for regulatory monitoring	National health authorities visit the wheat mills throughout the year to carry out technical audits and inspection of the fortification process and product. The government activities are mainly based on checking the producer's records. Therefore, it is important to keep in mind that "what has not been recorded has not been done". See Table C-1.

21. If there are two or more government agencies involved in external monitoring, clarifies the roles and responsibilities between different government agencies in external monitoring	(N/A) Only one government agency involved	Public Health Officers.
22. Allows for monitoring to be conducted often enough that problems can be identified and addressed on a timely basis; specifies a timeline for inspections (i.e. once every 6 months, increasing to once every 2 months if a discrepancy is found)	(1) Makes mention of a timeline	Doesn't say how often: National health authorities visit the wheat mills throughout the year to carry out technical audits and inspection of the fortification process and product.
23. If samples are to be taken, describes the sampling process: for example, number of samples, amount, frequency, individual vs. composite, where samples are taken in the process, and percent considered passing	(N/A) Does not describe the sampling process	
24. States applicability of using qualitative testing to determine the presence or absence of a vitamin or mineral (spot tests, iChecks)	(0) Does not state	
25. States registration is required in order to use a logo/be licensed to produce fortified foods	(0) Does not state that registration or licensing is required	
**COMMERCIAL MONITORING (conducted by government)**		
26. Provides justification for commercial monitoring at retail stores	(0) Does not provide justification for commercial monitoring	
27. Describes protocols and systems for commercial monitoring	(0) Does not describe	
28. If there are two or more government agencies involved in commercial monitoring, clarifies the roles and responsibilities between different government agencies in commercial monitoring	(0) Clarifies roles and responsibilities for no agencies	
29. Allows for monitoring to be conducted often enough that problems at the production site or import companies can be identified and addressed on a timely basis; specifies a timeline for inspections (i.e. once every 6 months) or works with production companies to correct noncompliance	(0) Does not state	
30. If samples are to be taken, describes the sampling process: for example, number of samples, amount, frequency, individual vs. composite, where samples are taken in the process, and percent considered passing	(N/A) Does not describe the sampling process	
**IMPORT MONITORING (conducted by government)**		
31. Provides justification for import monitoring at points of entry	(2) Provides justification for import monitoring	Wheat flour producers and importers are responsible of complying with the standards and regulations regarding wheat flour<br>fortification in the country.
32. Describes protocols and systems for import monitoring	(1) Does not explicitly state, but references protocols and systems for import monitoring	
33. If there are two or more government agencies involved in import monitoring, clarifies the roles and responsibilities between different government agencies in import monitoring	(0) Clarifies roles and responsibilities for no agencies	
34. If samples are to be taken, describes the sampling process: for example, number of samples, amount, frequency, individual vs. composite, where samples are taken in the process, and percent considered passing	(N/A) Does not describe the sampling process	
**ENFORCEMENT/PENALTIES**		
35. Indicates roles and responsibilities in enforcing the legislation	(0) Does not state	
36. States incentives to start fortification	(0) Does not state	
37. States incentives to continue fortification, including ensuring compliance	(0) Does not state	
38. States penalties to compel compliance	(0) Does not state	
39. Penalties are objectively defined (e.g. first penalty=$100, second penalty=$300)	(N/A) No penalties are stated (Answered (0) to previous question)	
40. States that enforcement is required to include feedback and support to improve performance and correct noncompliance	(0) Does not require	
**LABORATORY**		
41. References required analytical assays for nutrients (e.g. Liquid chromatography-mass spectrometry for folic acid, atomic absorption for iron and zinc.)	(2) References required assays	and minerals can be found in the literature and the mill should seek a<br>reliable external laboratory to analyze the samples.
42. States recognition that laboratory results are subject to several sources of variation and do not provide conclusive evidence of compliance or noncompliance	(0) Does not state recognition	
43. Focuses on the quantitative analysis of "marker" micronutrients such as iron	(2) Focuses on quantitative analysis of marker micronutrient such as iron	Send the samples to an external laboratory to determine their iron content quantitatively.
**REPORTING**		
44. States how government monitoring results are shared with stakeholders	(0) Does not state how results are shared	Reports seem to be all internal

Kosovo Wheat

Element	Scoring (use drop-down menus)	Comments (open text)
**GENERAL**		
1. States that legislation applies to at least one food vehicle fit for human consumption (types/grades to be fortified)	(2) States at least one type fit for human consumption	L-114: Article 2 - Scope - mandatory flour fortification with iron and folic acid, voluntary with vitamin A, B12, and zinc; L-114: Article 9(1) - types 400 and 500 are required.
2. States the public health objective; purpose and scope of legislation	(2) States the public health objective or general purpose of legislation	L-114: Article 1 - "as one of the ways that aims to prevent and reduce the diseases caused by the lack of iron and folic acid"
3. References latest available science or accepted international norms and recommendations, particularly for items that may not be covered in the country's documents	(2) States the documents referenced	L-114: Article 5(1) - "fortification with iron and folic acid is obligatory and shall be implemented according to the international standards"
4. Provides definitions that includes terms that are specific to fortification (e.g. fortified food, premix, fortificant, food vehicle)	(2) States at least one term related to fortification	L-114: Article 3 - fortified flour, folic acid, iron, vitamin A, vitamin B12, zinc, etc.
5. Provides repeals (if there is at least one prior document about fortification)	(0) Does not state	
6. Provides effective date or gives grace period for when fortification is to begin (e.g. effective 6 months from signing)	(2) States effective date or grace period for when fortification is to begin (e.g. effective 6 months from signing)	L-114: Article 15 - 6 months after publication
**MICRONUTRIENTS/PREMIX**		
7. States nutrients required	(2) States nutrients	1884: Article 3(2) - iron and folic acid
8. States fortificants (chemical compounds) to be used (including fortificants that are allowable as options)	(2) States fortificants for at least one nutrient	1884: Article 3(2) - iron of the form Ferro Sulfate
9. States fortification levels	(1) States one number only	1884: Article 3(2) - 25.2 mg/kg iron, 1.5mg/kg folic acid
10. States consideration of bioavailability/biological activity of fortificants	(0) Does not state any consideration	
11. States consideration of nutrient stability	(2) States consideration of nutrient stability	L-114: Article 10(1) - Packaging should be done to in order to conserve vitamins and minerals.
**COSTING**		
12. States that the cost of fortification is regulated through cost sharing schemes (between government, industry, consumers) or tax measures [to assist industry]	(0) Does not state any consideration	
13. States consideration of the financial responsibility of monitoring and enforcing fortification (schedule of fees, budget) [on the government side]	(2) Shows consideration that monitoring costs money	L-114: Article 14 - All incomes coming due to the implementation of this law goes to the budget of the Republic of Kosovo; 1884: Article 10(5/6) - expenses split between producer and Food and Vet Agency
**LABELING**		
14. Includes some sort of statement/label/logo that makes it clear that the product is fortified	(2) Includes a statement, label, or logo	L-114: Article 10(2.6) - The word "Fortified" shall be placed on the packaging label; 1884: Article 4 - also to contain a logo
15. Provides guidance on health claims that can be made for this product (specific to micronutrients added through fortification)	(0) Does not provide	
**INTERNAL MONITORING (conducted by industry)**		
16. If samples are to be taken, describes the sampling process: for example, number of samples, amount, frequency, individual vs. composite, where samples are taken in the process, and percent considered passing	(2) States that samples should be taken as part of internal monitoring	1884: Article 10(2.2) - quality and quantity analysis in an accredited lab
assurance/quality control in regards to fortification	(2) States requirement of QA/QC for fortification	import, package, and selling are obliged to guarantee the
18. States applicability of using qualitative testing to determine the presence or absence of a vitamin or mineral (spot tests, iChecks)	(2) States applicability of spot test to determine presence/absence of vitamin or mineral specific to internal monitoring	1884: Article 9(3) - no spot test, but describes that producers should keep record of quantity produced and used premix on a regular basis; 1884: Article 10(2.1) - iron spot test
**EXTERNAL MONITORING (conducted by government)**		
19.States requirement for external monitoring at the production site to assure compliance with standards and regulations	(2) States requirement for external monitoring or the need for audits/inspections	1884: Article 11(1) - official control carried out by inspectors to control and verify completion of the criteria for enriched flour in compliance with procedures
20. Describes protocols and systems for regulatory monitoring	(2) Includes checklists or provides detailed description of regulatory monitoring procedures	1884: Article 11(2) - according to instructions and forms attached
21. If there are two or more government agencies involved in external monitoring, clarifies the roles and responsibilities between different government agencies in external monitoring	(2) Clarifies roles and responsibilities for more than one agency	L-114: Article 4 and 5(3) - "Respective Ministry with this Law"; L-114: Article 7: authorized and accredited institutions shall carry out the assessment of control of flour in accordance with provisions…; L-114: Article 10(4) - Conditions for packaging and labeling shall be regulated by the respective Ministry and Ministry of Trade and Industry; 1884: Article 10(6) - official controls by inspectors - Food and Vet Agency
22. Allows for monitoring to be conducted often enough that problems can be identified and addressed on a timely basis; specifies a timeline for inspections (i.e. once every 6 months, increasing to once every 2 months if a discrepancy is found)	(0) Does not state	
23. If samples are to be taken, describes the sampling process: for example, number of samples, amount, frequency, individual vs. composite, where samples are taken in the process, and percent considered passing	(1) States that samples should be taken (generally)	

24. States applicability of using qualitative testing to determine the presence or absence of a vitamin or mineral (spot tests, iChecks)	(2) States applicability of spot test to determine presence/absence of vitamin or mineral specific to external monitoring	1884: Article 10(3) - Spot tests done by producers, inspectors…
25. States registration is required in order to use a logo/be licensed to produce fortified foods	(0) Does not state that registration or licensing is required	
**COMMERCIAL MONITORING (conducted by government)**		
26. Provides justification for commercial monitoring at retail stores	(2) Provides justification for commercial monitoring	L-114: Article 6 - Business entities in production, transport, import, package, and selling are obliged to guarantee the safety and quality to the consumer
27. Describes protocols and systems for commercial monitoring	(1) Does not explicitly describe, but references protocols and systems for regulatory monitoring	1884: Article 10(3) - testing by Food and Vet Agency conducted by inspectors at production site, warehouse, and trading
28. If there are two or more government agencies involved in commercial monitoring, clarifies the roles and responsibilities between different government agencies in commercial monitoring	(2) Clarifies roles and responsibilities for more than one agency	1884: Article 10(3) - testing by Food and Vet Agency conducted by inspectors at production site, warehouse, and trading
29. Allows for monitoring to be conducted often enough that problems at the production site or import companies can be identified and addressed on a timely basis; specifies a timeline for inspections (i.e. once every 6 months) or works with production companies to correct noncompliance	(0) Does not state	
30. If samples are to be taken, describes the sampling process: for example, number of samples, amount, frequency, individual vs. composite, where samples are taken in the process, and percent considered passing	(N/A) Does not describe the sampling process	
**IMPORT MONITORING (conducted by government)**		
31. Provides justification for import monitoring at points of entry	(2) Provides justification for import monitoring	L-114: Article 6 - Business entities in production, transport, import, package, and selling are obliged to guarantee the safety and quality to the consumer; L-114: Article 9(5) - flour imported should be fortified according to the law
32. Describes protocols and systems for import monitoring	(2) Includes checklists or detailed description of import monitoring procedures	L-114: Article 11(5) - Checklist of items required for import; 1884: Article 7 - premix producer should be registered with MoAg
33. If there are two or more government agencies involved in import monitoring, clarifies the roles and responsibilities between different government agencies in import monitoring	(1) Clarifies roles and responsibilities for one agency	1884: Article 7 - Ministry of Agriculture, Forestry, and Rural Development registers premix producers for import
34. If samples are to be taken, describes the sampling process: for example, number of samples, amount, frequency, individual vs. composite, where samples are taken in the process, and percent considered passing	(N/A) Does not describe the sampling process	
**ENFORCEMENT/PENALTIES**		
35. Indicates roles and responsibilities in enforcing the legislation	(2) States the role and responsibilities of government in enforcement	1884: Article 15 - non-application of the administration is sanctioned by Article 11 of Law-114.
36. States incentives to start fortification	(0) Does not state	
37. States incentives to continue fortification, including ensuring compliance	(0) Does not state	
38. States penalties to compel compliance	(2) States any penalties	L-114: Article 11(3) - businesses that cause th eloss of quality and quantity of components, verified with lab analyses, shall be baned from sale and revoked executing of the activity; L-114: Article 12 - Offenses
39. Penalties are objectively defined (e.g. first penalty=$100, second penalty=$300)	(2) Penalties are objectively laid out in the document (e.g. first penalty=$100, second penalty=$300)	L-114: Article 12 - offenses
40. States that enforcement is required to include feedback and support to improve performance and correct noncompliance	(0) Does not require	
**LABORATORY**		
41. References required analytical assays for nutrients (e.g. Liquid chromatography-mass spectrometry for folic acid, atomic absorption for iron and zinc.)	(2) References required assays	1884: Annex 2 - spec method for iron; Annex 3 - HPLC method for folic acid
42. States recognition that laboratory results are subject to several sources of variation and do not provide conclusive evidence of compliance or noncompliance	(0) Does not state recognition	
43. Focuses on the quantitative analysis of "marker" micronutrients such as iron	(2) Focuses on quantitative analysis of marker micronutrient such as iron	** Does only provide testing for iron and folic acid - voluntary vitamins (A, B12, zinc) are not tested for
**REPORTING**		
44. States how government monitoring results are shared with stakeholders	(0) Does not state how results are shared	

Kuwait Wheat

Element	Scoring (use drop-down menus)	Comments (open text)
**GENERAL**		
1. States that legislation applies to at least one food vehicle fit for human consumption (types/grades to be fortified)	(2) States at least one type fit for human consumption	This standard is concerned with wheat flour (Triticum aestivum L, Triticum compactum Host or mixtures thereof), enriched wheat flour and enriched treated wheat flour for human consumption.
2. States the public health objective; purpose and scope of legislation	(0) Does not state	
3. References latest available science or accepted international norms and recommendations, particularly for items that may not be covered in the country's documents	(0) Does not state	
4. Provides definitions that includes terms that are specific to fortification (e.g. fortified food, premix, fortificant, food vehicle)	(2) States at least one term related to fortification	Enriched wheat flour: Wheat flour to which some minerals, vitamins and other<br>suitable nutrients are added.
5. Provides repeals (if there is at least one prior document about fortification)	(2) States repeals	This standard has been approved as Gulf (Technical Regulation by GSO Board of Directors in<br>its meeting No 5 .held on 16 / 4 / 1427 / H , 14/ 5 /2006 The approved standard will<br>replace and supersede the standard No. (194 /1994)
6. Provides effective date or gives grace period for when fortification is to begin (e.g. effective 6 months from signing)	(0) Does not state	Date of Publish in Offical Gazete 30/04/2007
**MICRONUTRIENTS/PREMIX**		
7. States nutrients required	(2) States nutrients	Annex (3)<br>Enrichment Substances<br>Substance Fraction<br>Thiamine 6.38 mg/kg min.<br>Riboflavin 3.96 mg/kg min.<br>Niacin 52.91 mg/kg min.<br>Calcium content 2115 mg/kg max.<br>Iron 30 mg/kg min.<br>Folic acid 1.5-2 mg/kg min.<br>Vitamin D 551.15 IU min<br>Wheat germ 5% max
8. States fortificants (chemical compounds) to be used (including fortificants that are allowable as options)	(0) Does not state	Annex (3)<br>Enrichment Substances<br>Substance Fraction<br>Thiamine 6.38 mg/kg min.<br>Riboflavin 3.96 mg/kg min.<br>Niacin 52.91 mg/kg min.<br>Calcium content 2115 mg/kg max.<br>Iron 30 mg/kg min.<br>Folic acid 1.5-2 mg/kg min.<br>Vitamin D 551.15 IU min<br>Wheat germ 5% max
9. States fortification levels	(1) States one number only	Annex (3)<br>Enrichment Substances<br>Substance Fraction<br>Thiamine 6.38 mg/kg min.<br>Riboflavin 3.96 mg/kg min.<br>Niacin 52.91 mg/kg min.<br>Calcium content 2115 mg/kg max.<br>Iron 30 mg/kg min.<br>Folic acid 1.5-2 mg/kg min.<br>Vitamin D 551.15 IU min<br>Wheat germ 5% max
10. States consideration of bioavailability/biological activity of fortificants	(2) States some consideration of bioavailability (mentions these or related terms)	Iron and calcium shall only be added in forms that are unharmful and easy to<br>absorb.
11. States consideration of nutrient stability	(0) Does not state any consideration	The packages must keep the product clean, and does not affect its natural characteristics. (natural characteristics could mean stability).
**COSTING**		
12. States that the cost of fortification is regulated through cost sharing schemes (between government, industry, consumers) or tax measures [to assist industry]	(0) Does not state any consideration	
13. States consideration of the financial responsibility of monitoring and enforcing fortification (schedule of fees, budget) [on the government side]	(0) Does not state any consideration	
**LABELING**		

14. Includes some sort of statement/label/logo that makes it clear that the product is fortified	(2) Includes a statement, label, or logo	9- LABELLING<br>Without prejudice to the requirements of GSO Standards mentioned in items 2.1<br>and 2.4 the following information shall be declared on the label of each package:<br>- Extraction ratio and type (White - Wheatmeal - Wholemeal).<br>- Additives if used.<br>- Names of enrichment substances and its ratio in 100 g if added.
15. Provides guidance on health claims that can be made for this product (specific to micronutrients added through fortification)	(0) Does not provide	Could be in GSO 9/1995 "Labelling of Prepackaged Foods", but we don't know
**INTERNAL MONITORING (conducted by industry)**		
16. If samples are to be taken, describes the sampling process: for example, number of samples, amount, frequency, individual vs. composite, where samples are taken in the process, and percent considered passing	(1) States that samples should be taken (generally)	Sampling shall be carried out according to GSO standard mentioned in item 2.5. (couldn't find standard for free, had to be purchased)
17. States that industry is required to follow quality assurance/quality control in regards to fortification	(0) Does not state requirement	5.16 may have QA/QC discussion, but not clear
18. States applicability of using qualitative testing to determine the presence or absence of a vitamin or mineral (spot tests, iChecks)	(0) Does not state	Tests and examination shall be carried out according to GSO standard mentioned<br>in item 2.11 (could not find this standard online)
**EXTERNAL MONITORING (conducted by government)**		
19.States requirement for external monitoring at the production site to assure compliance with standards and regulations	(0) Does not state requirement	
20. Describes protocols and systems for regulatory monitoring	(0) Does not describe	
21. If there are two or more government agencies involved in external monitoring, clarifies the roles and responsibilities between different government agencies in external monitoring	(0) Clarifies roles and responsibilities for no agencies	
22. Allows for monitoring to be conducted often enough that problems can be identified and addressed on a timely basis; specifies a timeline for inspections (i.e. once every 6 months, increasing to once every 2 months if a discrepancy is found)	(0) Does not state	
23. If samples are to be taken, describes the sampling process: for example, number of samples, amount, frequency, individual vs. composite, where samples are taken in the process, and percent considered passing	(1) States that samples should be taken (generally)	Sampling shall be carried out according to GSO standard mentioned in item 2.5. (couldn't find standard for free, had to be purchased)
24. States applicability of using qualitative testing to determine the presence or absence of a vitamin or mineral (spot tests, iChecks)	(0) Does not state	Tests and examination shall be carried out according to GSO standard mentioned<br>in item 2.11 (could not find this standard online)
25. States registration is required in order to use a logo/be licensed to produce fortified foods	(0) Does not state that registration or licensing is required	
**COMMERCIAL MONITORING (conducted by government)**		
26. Provides justification for commercial monitoring at retail stores	(0) Does not provide justification for commercial monitoring	
27. Describes protocols and systems for commercial monitoring	(0) Does not describe	
28. If there are two or more government agencies involved in commercial monitoring, clarifies the roles and responsibilities between different government agencies in commercial monitoring	(0) Clarifies roles and responsibilities for no agencies	
29. Allows for monitoring to be conducted often enough that problems at the production site or import companies can be identified and addressed on a timely basis; specifies a timeline for inspections (i.e. once every 6 months) or works with production companies to correct noncompliance	(N/A) No commercial monitoring occurs	
30. If samples are to be taken, describes the sampling process: for example, number of samples, amount, frequency, individual vs. composite, where samples are taken in the process, and percent considered passing	(N/A) Does not describe the sampling process	
**IMPORT MONITORING (conducted by government)**		
31. Provides justification for import monitoring at points of entry	(0) Does not provide justification for import monitoring	
32. Describes protocols and systems for import monitoring	(0) Does not state	
33. If there are two or more government agencies involved in import monitoring, clarifies the roles and responsibilities between different government agencies in import monitoring	(0) Clarifies roles and responsibilities for no agencies	

34. If samples are to be taken, describes the sampling process: for example, number of samples, amount, frequency, individual vs. composite, where samples are taken in the process, and percent considered passing	(N/A) Does not describe the sampling process
**ENFORCEMENT/PENALTIES**	
35. Indicates roles and responsibilities in enforcing the legislation	(0) Does not state
36. States incentives to start fortification	(0) Does not state
37. States incentives to continue fortification, including ensuring compliance	(0) Does not state
38. States penalties to compel compliance	(0) Does not state
39. Penalties are objectively defined (e.g. first penalty=$100, second penalty=$300)	(N/A) No penalties are stated (Answered (0) to previous question)
40. States that enforcement is required to include feedback and support to improve performance and correct noncompliance	(0) Does not require
**LABORATORY**	
41. References required analytical assays for nutrients (e.g. Liquid chromatography-mass spectrometry for folic acid, atomic absorption for iron and zinc.)	(0) Does not state requirements
42. States recognition that laboratory results are subject to several sources of variation and do not provide conclusive evidence of compliance or noncompliance	(0) Does not state recognition
43. Focuses on the quantitative analysis of "marker" micronutrients such as iron	(0) Does not state
**REPORTING**	
44. States how government monitoring results are shared with stakeholders	(0) Does not state how results are shared

Kyrgyzstan Wheat

Element	Scoring (use drop-down menus)	Comments (open text)
**GENERAL**		
1. States that legislation applies to at least one food vehicle fit for human consumption (types/grades to be fortified)	(2) States at least one type fit for human consumption	Article 4: enrichment of premium and first grade white baking flour
2. States the public health objective; purpose and scope of legislation	(2) States the public health objective or general purpose of legislation	Article 3: prevention of diseases and conditions caused by insufficiency of iron and other microlements and vitamins...
3. References latest available science or accepted international norms and recommendations, particularly for items that may not be covered in the country's documents	(2) States the documents referenced	Article 2 - international agreements
4. Provides definitions that includes terms that are specific to fortification (e.g. fortified food, premix, fortificant, food vehicle)	(2) States at least one term related to fortification	
5. Provides repeals (if there is at least one prior document about fortification)	(2) States repeals	Article 2: if international agreements establish different rules, then those take precedence
6. Provides effective date or gives grace period for when fortification is to begin (e.g. effective 6 months from signing)	(2) States effective date or grace period for when fortification is to begin (e.g. effective 6 months from signing)	Article 15
**MICRONUTRIENTS/PREMIX**		
7. States nutrients required	(2) States nutrients	enrichment of flour with iron
8. States fortificants (chemical compounds) to be used (including fortificants that are allowable as options)	(0) Does not state	Maybe these are in a technical regulation?
9. States fortification levels	(0) Does not state	
10. States consideration of bioavailability/biological activity of fortificants	(0) Does not state any consideration	
11. States consideration of nutrient stability	(2) States consideration of nutrient stability	Article 11 - packaging should allow for preservation of vitamins in the quantity specified
**COSTING**		
12. States that the cost of fortification is regulated through cost sharing schemes (between government, industry, consumers) or tax measures [to assist industry]	(2) States consideration of either cost regulation method	Article 4 - cost sharing by preferential use of fortified flour in educational, healthcare, social development, justice, and other budgeted establishments; also taking measures for economic stimulation of legal and physical persons involved in production by disbursing funds for premis and other means
13. States consideration of the financial responsibility of monitoring and enforcing fortification (schedule of fees, budget) [on the government side]	(2) Shows consideration that monitoring costs money	Article 4 - government adops and finances specific national programmes, including fortified flour
**LABELING**		
14. Includes some sort of statement/label/logo that makes it clear that the product is fortified	(2) Includes a statement, label, or logo	Article 11 - includes the words "obogashchennoy" and "baytylgan" to signify "enriched"
15. Provides guidance on health claims that can be made for this product (specific to micronutrients added through fortification)	(2) Provides guidance on health claims specific to micronutrients added through fortification	Article 7 - requreiemtns are subject to adherence in terms of... prevention of actions which mislead consumers
**INTERNAL MONITORING (conducted by industry)**		
16. If samples are to be taken, describes the sampling process: for example, number of samples, amount, frequency, individual vs. composite, where samples are taken in the process, and percent considered passing	(N/A) Does not describe the sampling process	
17. States that industry is required to follow quality assurance/quality control in regards to fortification	(2) States requirement of QA/QC for fortification	Article 7: people involved in production, transportation, storage, and sale must guarantee to consumers its safety and quality
18. States applicability of using qualitative testing to determine the presence or absence of a vitamin or mineral (spot tests, iChecks)	(0) Does not state	
**EXTERNAL MONITORING (conducted by government)**		
19.States requirement for external monitoring at the production site to assure compliance with standards and regulations	(2) States requirement for external monitoring or the need for audits/inspections	Article 4 - government will monitor fulfillment of compulsory requirements
20. Describes protocols and systems for regulatory monitoring	(1) Does not explicitly describe, but references protocols and systems for regulatory monitoring	Article 9 - use of identifal forms and methods for monitoring enrichment and consumption
21. If there are two or more government agencies involved in external monitoring, clarifies the roles and responsibilities between different government agencies in external monitoring	(0) Clarifies roles and responsibilities for no agencies	
22. Allows for monitoring to be conducted often enough that problems can be identified and addressed on a timely basis; specifies a timeline for inspections (i.e. once every 6 months, increasing to once every 2 months if a discrepancy is found)	(0) Does not state	
23. If samples are to be taken, describes the sampling process: for example, number of samples, amount, frequency, individual vs. composite, where samples are taken in the process, and percent considered passing	(N/A) Does not describe the sampling process	

24. States applicability of using qualitative testing to determine the presence or absence of a vitamin or mineral (spot tests, iChecks)	(0) Does not state	
25. States registration is required in order to use a logo/be licensed to produce fortified foods	(2) Describes some type of registration or licensing	
**COMMERCIAL MONITORING (conducted by government)**		
26. Provides justification for commercial monitoring at retail stores	(2) Provides justification for commercial monitoring	Article 5 - local authorities provide for monitoring of conditions of sale of enriched flour
27. Describes protocols and systems for commercial monitoring	(1) Does not explicitly describe, but references protocols and systems for regulatory monitoring	Article 5.5 - Monitor… sale… in accordance with requirements established by normative legal acts
28. If there are two or more government agencies involved in commercial monitoring, clarifies the roles and responsibilities between different government agencies in commercial monitoring	(0) Clarifies roles and responsibilities for no agencies	
29. Allows for monitoring to be conducted often enough that problems at the production site or import companies can be identified and addressed on a timely basis; specifies a timeline for inspections (i.e. once every 6 months) or works with production companies to correct noncompliance	(0) Does not state	
30. If samples are to be taken, describes the sampling process: for example, number of samples, amount, frequency, individual vs. composite, where samples are taken in the process, and percent considered passing	(N/A) Does not describe the sampling process	
**IMPORT MONITORING (conducted by government)**		
31. Provides justification for import monitoring at points of entry	(2) Provides justification for import monitoring	Article 10 - procedure for import of enriched flour is realised in accordance with legislation
32. Describes protocols and systems for import monitoring	(1) Does not explicitly state, but references protocols and systems for import monitoring	Article 12 - list of items needed for import
33. If there are two or more government agencies involved in import monitoring, clarifies the roles and responsibilities between different government agencies in import monitoring	(0) Clarifies roles and responsibilities for no agencies	
34. If samples are to be taken, describes the sampling process: for example, number of samples, amount, frequency, individual vs. composite, where samples are taken in the process, and percent considered passing	(N/A) Does not describe the sampling process	
**ENFORCEMENT/PENALTIES**		
35. Indicates roles and responsibilities in enforcing the legislation	(2) States the role and responsibilities of government in enforcement	Article 4 - monitors fulfillment of compulsory requirements for enrichment of flour
36. States incentives to start fortification	(2) States any incentives to encourage fortification initiation (e.g. tax incentives for new equipment or premix)	Article 4 - takes measures for the economic stimulation of producers
37. States incentives to continue fortification, including ensuring compliance	(0) Does not state	
38. States penalties to compel compliance	(2) States any penalties	Article 12 - removal from sale
39. Penalties are objectively defined (e.g. first penalty=$100, second penalty=$300)	(0) Penalties are not objectively laid out	
40. States that enforcement is required to include feedback and support to improve performance and correct noncompliance	(0) Does not require	
**LABORATORY**		
41. References required analytical assays for nutrients (e.g. Liquid chromatography-mass spectrometry for folic acid, atomic absorption for iron and zinc.)	(0) Does not state requirements	
42. States recognition that laboratory results are subject to several sources of variation and do not provide conclusive evidence of compliance or noncompliance	(0) Does not state recognition	
43. Focuses on the quantitative analysis of "marker" micronutrients such as iron	(0) Does not state	
**REPORTING**		
44. States how government monitoring results are shared with stakeholders	(0) Does not state how results are shared	

Liberia Wheat

Element	Scoring (use drop-down menus)	Comments (open text)
**GENERAL**		
1. States that legislation applies to at least one food vehicle fit for human consumption (types/grades to be fortified)	(2) States at least one type fit for human consumption	The National Fortification Alliance (NFA) of Liberia is proud to announce that revised food fortification standards have been adopted for Liberia through the Ministry of Commerce and Industry and the Ministry of Health and Social Welfare for the following staple foods: sugar, wheat flour, cooking oil and salt.
2. States the public health objective; purpose and scope of legislation	(2) States the public health objective or general purpose of legislation	The purpose of this Regulation is to strengthen the adopted Food Fortification Standards of the Division of Standards of the MoCI and Food Safety Guidelines of the Division of Environmental and Occupational Health (DEOH) of the MoHSW for the provision, specifically but not limited to, the mandatory fortification of salt, wheat flour, sugar, and cooking oil, and to authorize the MoCI and MoHSW to require or permit the fortification of other foods to address and alleviate other nutritional deficiencies of the people of Liberia and to otherwise promote their nutritional status and health.
3. References latest available science or accepted international norms and recommendations, particularly for items that may not be covered in the country's documents	(2) States the documents referenced	In establishing standards for food, the Ministries shall take into account fully the recommended international standards of the Codex Alimentarius Commission, including those related to fortification of foods.
4. Provides definitions that includes terms that are specific to fortification (e.g. fortified food, premix, fortificant, food vehicle)	(2) States at least one term related to fortification	As used in this Regulation, the following terms shall be given the meanings described below: additive, adulterate, advertisement, authorized officer, certificate of analysis, distribute, drug, essential nutrient, export, food, fortified food or enriched food, good manufacturing practice, hazard analysis and critical control point, import, ingredient, iodized salt, label, license, logo, manufacture, ministers, monitoring, non-conformance, package, person, premises, preventive maintenance, quality control, regulatory requirements, sanitation operations, sell, verification and validation
5. Provides repeals (if there is at least one prior document about fortification)	(2) States repeals	This Regulation shall become effective 01.07.2014 and shall repeal all prior inconsistent provisions of other enactments in force on the effective date.
6. Provides effective date or gives grace period for when fortification is to begin (e.g. effective 6 months from signing)	(2) States effective date or grace period for when fortification is to begin (e.g. effective 6 months from signing)	This Regulation shall become effective 01.07.2014 and shall repeal all prior inconsistent provisions of other enactments in force on the effective date.
**MICRONUTRIENTS/PREMIX**		
7. States nutrients required	(2) States nutrients	Liberia gives a table with a column for the nutrient and a column for the fortification compound.
8. States fortificants (chemical compounds) to be used (including fortificants that are allowable as options)	(2) States fortificants for at least one nutrient	Liberia gives a table with a column for the nutrient and a column for the fortification compound.
9. States fortification levels	(2) States a range or number with +/-	A specific value with an allowable range is given
10. States consideration of bioavailability/biological activity of fortificants	(0) Does not state any consideration	
11. States consideration of nutrient stability	(2) States consideration of nutrient stability	1. Examining the fortificant(s) to ensure that specification are met.<br>- Certificate of analysis exists for every delivery of the fortificant(s).<br>- Check if the fortificant(s) used is still within the market shelf-life.
**COSTING**		
12. States that the cost of fortification is regulated through cost sharing schemes (between government, industry, consumers) or tax measures [to assist industry]	(2) States consideration of either cost regulation method	Programmatic costs for the fortification program may be supported by the appropriate agency, by development partners, or by seeking external funding, as appropriate
13. States consideration of the financial responsibility of monitoring and enforcing fortification (schedule of fees, budget) [on the government side]	(0) Does not state any consideration	
**LABELING**		
14. Includes some sort of statement/label/logo that makes it clear that the product is fortified	(2) Includes a statement, label, or logo	All products designated as fortified should bare the ENRICHED logo in accordance with the logo guidelines.
15. Provides guidance on health claims that can be made for this product (specific to micronutrients added through fortification)	(0) Does not provide	
**INTERNAL MONITORING (conducted by industry)**		
16. If samples are to be taken, describes the sampling process: for example, number of samples, amount, frequency, individual vs. composite, where samples are taken in the process, and percent considered passing	(2) States that samples should be taken as part of internal monitoring	The production facility must do the following and document it accordingly. Samples during production will be done every shift or every batch to confirm that fortification is taking place. In continuous processes, a sample is collected every hour or two hours depending on production volumes and tested qualitatively. A composite sample is made for the shift (or day) and tested quantitatively in-house where possible.

17. States that industry is required to follow quality assurance/quality control in regards to fortification	(2) States requirement of QA/QC for fortification	All persons who import, manufacture, package or repackage, label, sell, or export food shall establish procedures and carry out activities for quality assurance in accordance with requirements prescribed in regulations to ensure that their activities and the food in their possession or under their control meets applicable regulatory requirements.
18. States applicability of using qualitative testing to determine the presence or absence of a vitamin or mineral (spot tests, iChecks)	(2) States applicability of spot test to determine presence/absence of vitamin or mineral specific to internal monitoring	a sample is collected every hour or two hours depending on production volumes and tested qualitatively.
**EXTERNAL MONITORING (conducted by government)**		
19.States requirement for external monitoring at the production site to assure compliance with standards and regulations	(2) States requirement for external monitoring or the need for audits/inspections	Production facilities are inspected through the standards inspectors of the Ministry of Commerce and Industry to ensure the standards are adhered to. Appropriate product packaging to ensure that the fortificants levels claimed are in accordance with the standard levels.
20. Describes protocols and systems for regulatory monitoring	(2) Includes checklists or provides detailed description of regulatory monitoring procedures	• General QA practices and record keeping including sample collection, production procedures, and equipment maintenance. This includes ensuring appropriate hygiene levels.<br>• Appropriate product packaging to ensure that the fortificants levels claimed are in accordance with the standard levels. In the same manner, the storage areas and practices must be inspected.
21. If there are two or more government agencies involved in external monitoring, clarifies the roles and responsibilities between different government agencies in external monitoring	(2) Clarifies roles and responsibilities for more than one agency	The MoCI, through their Standards Officers, is responsible for inspecting local industry and manufacturers in cooperation with the MoHSW;
22. Allows for monitoring to be conducted often enough that problems can be identified and addressed on a timely basis; specifies a timeline for inspections (i.e. once every 6 months, increasing to once every 2 months if a discrepancy is found)	(2) Describes frequency and how it is responsive to the needs of industry or the stage of fortification implementation	All producers in the country must be inspected at least three times per year by MoCI Standards inspectors. For new facilities that become established, testing should be done once a month for the first three or four months. Inspections following the initial inspection cycle may be limited to industries and importers that have a history of non-compliance. However, all industries must be inspected once a year regardless of compliance.
23. If samples are to be taken, describes the sampling process: for example, number of samples, amount, frequency, individual vs. composite, where samples are taken in the process, and percent considered passing	(2) States that samples should be taken as part of external monitoring	External inspectors to facilities should have a total of 5 flour samples and one premix sample from each visit.
24. States applicability of using qualitative testing to determine the presence or absence of a vitamin or mineral (spot tests, iChecks)	(2) States applicability of spot test to determine presence/absence of vitamin or mineral specific to external monitoring	The composite samples are sent to the National Standards Laboratory or they are tested in the county if iChecks are available. Due to the nature of reagents used for some qualitative tests, the following should still be done in a laboratory. The County Water Laboratories would serve as ideal sites for such test to be conducted: Qualitative test for iron - iron spot test
25. States registration is required in order to use a logo/be licensed to produce fortified foods	(2) Describes some type of registration or licensing	A certified copy of registration certificate of the company; Industry, importers and manufacturers are not permitted to use the logo unless they are authorized by the NSL. Authorization means the following: NSL will test the products annually to renew importer's, manufacturer's and industry's right to use the FFL. NSL will notify manufacturers each year of their fortification logo use status.
**COMMERCIAL MONITORING (conducted by government)**		
26. Provides justification for commercial monitoring at retail stores	(2) Provides justification for commercial monitoring	Commercial inspection is the verification of legal compliance of fortified foods sold in retail supermarkets, markets, grocery stores, and wholesale stores. It also includes inspection at bakeries as a convenient sampling site for fortified foods namely salt, sugar, flour and cooking oil. This monitoring allows for the detection in the market of brands that are not approved by the Ministry of Health or do not comply with local fortification regulations. It also helps to confirm whether brands that have previously been inspected in factories and importation sites are indeed fulfilling the requirements as claimed by inspectors during the external monitoring process. Furthermore, commercial monitoring serves as an education tool since inspectors are able to inform the retailers about the existence of the fortification program, the benefits of fortification, their role as retailers, and their rights as consumers.
27. Describes protocols and systems for commercial monitoring	(2) Includes checklists or provides detailed description of commercial monitoring procedures	Table 1: Inspection form for commercial inspections:

28. If there are two or more government agencies involved in commercial monitoring, clarifies the roles and responsibilities between different government agencies in commercial monitoring	(2) Clarifies roles and responsibilities for more than one agency	Inspectors of the Ministry of Health and Social Welfare and the Ministry of Commerce and Industry will conduct commercial monitoring at the market level.
29. Allows for monitoring to be conducted often enough that problems at the production site or import companies can be identified and addressed on a timely basis; specifies a timeline for inspections (i.e. once every 6 months) or works with production companies to correct noncompliance	(2) Describes frequency and how it is responsive to the needs of industry or the stage of fortification implementation	Wholesalers and retailers should be inspected at least once per quarter.
30. If samples are to be taken, describes the sampling process: for example, number of samples, amount, frequency, individual vs. composite, where samples are taken in the process, and percent considered passing	(2) States that samples should be taken as part of commercial monitoring	Inspectors should fill out the attached forms for each brand in the market. The forms must include brand name information as it is our means of tracking the importers that will be held responsible in cases of non-compliance. Tests at market level shall include qualitative tests as the first method of choice. Samples that are positive to the qualitative testing shall be grouped (by brand name) to make one composite sample of the brand for the area.
**IMPORT MONITORING (conducted by government)**		
31. Provides justification for import monitoring at points of entry	(2) Provides justification for import monitoring	The inspection of food at border or Ports is part of the responsibilities of the Government of Liberia to ensure that foods entering the border are compliant with the technical regulations of the national food fortification program. This is to make sure that specified foods are not imported and distributed to consumers if they are not fortified appropriately. The process involves collecting food samples and reviewing documentation and declarations on food labels. This is achieved by reviewing the Certificate of Conformity or Analysis (COA) accompanying imported food batches as well as collecting samples at ports of entry and testing them qualitatively on site.
32. Describes protocols and systems for import monitoring	(2) Includes checklists or detailed description of import monitoring procedures	The process involves collecting food samples and reviewing documentation and declarations on food labels. This is achieved by reviewing the Certificate of Conformity or Analysis (COA) accompanying imported food batches as well as collecting samples at ports of entry and testing them qualitatively on site.
33. If there are two or more government agencies involved in import monitoring, clarifies the roles and responsibilities between different government agencies in import monitoring	(2) Clarifies roles and responsibilities for more than one agency	Officials from the customs officers (Ministry of Finance) in conjunction with the responsible government institution in charge of certifying the quality of foods (e.g. Ministry of Commerce and Industry and Ministry of Health and Social Welfare) should perform the task of collecting samples, testing them qualitatively and reviewing documentation before the food can be allowed to enter the country.
34. If samples are to be taken, describes the sampling process: for example, number of samples, amount, frequency, individual vs. composite, where samples are taken in the process, and percent considered passing	(2) States that samples should be taken as part of import monitoring	The process involves collecting food samples and reviewing documentation and declarations on food labels. This is achieved by reviewing the Certificate of Conformity or Analysis (COA) accompanying imported food batches as well as collecting samples at ports of entry and testing them qualitatively on site.
**ENFORCEMENT/PENALTIES**		
35. Indicates roles and responsibilities in enforcing the legislation	(2) States the role and responsibilities of government in enforcement	The roles and responsibilities section of these Guidelines clarify the respective responsibilities of the different Ministries and Ministry Divisions, as well as those of the private sector and civil society.
36. States incentives to start fortification	(0) Does not state	
37. States incentives to continue fortification, including ensuring compliance	(2) States any incentives to encourage the continuation of fortification (e.g. transport priority, favorable tax or tariff treatment, or patent rights)	Foods that are fortified in compliance with regulatory requirements shall enjoy priority over non-fortified foods of the same class and category with respect to transport, storage, and display, including retail shelf space; shall be entitled to carry a logo authorized by the Ministries; and shall be entitled to any other favored treatment established by the government.

38. States penalties to compel compliance	(2) States any penalties	Penalties authorized by this section may be imposed for each substantial violation of regulatory requirements and may be imposed singly or in combination, as follows: a. Imposition of a civil fine of no less than 1000 United States Dollars, in accordance with criteria established in regulations, taking into account the seriousness, including scale of production and the potential harm for the consumers of the violation(s), whether the same or similar violations have occurred previously, and such other factors as the Ministries deems appropriate. In accordance to the severity, the amount charged is at the discretion of the MoCI; b. Issuance of an order to cease and desist from any activity that does not comply with regulatory requirements; c. Confiscation and destruction or other disposition of food that does not meet regulatory requirements; d. Adverse publicity of unfavorable inspection, investigation of analysis results; and e. License restriction, suspension or revocation.
39. Penalties are objectively defined (e.g. first penalty=$100, second penalty=$300)	(0) Penalties are not objectively laid out	Needs to be more explicit
40. States that enforcement is required to include feedback and support to improve performance and correct noncompliance	(2) Requires any feedback/support to improve performance	(This is a key action point...ie not completed yet) Ensure a comprehensive monitoring database is created to house collected national fortification data by staple, importer, brand, and test result and that allows for reporting back on a quarterly basis and so that issues of non-compliance can be dealt with in a timely and effective manner.
**LABORATORY**		
41. References required analytical assays for nutrients (e.g. Liquid chromatography-mass spectrometry for folic acid, atomic absorption for iron and zinc.)	(0) Does not state requirements	
42. States recognition that laboratory results are subject to several sources of variation and do not provide conclusive evidence of compliance or noncompliance	(0) Does not state recognition	
43. Focuses on the quantitative analysis of "marker" micronutrients such as iron	(2) Focuses on quantitative analysis of marker micronutrient such as iron	If the iCheck is available, quantitative testing can be conducted at import sites otherwise the samples should be sent to the National Standards Laboratory for determining the level of an indicator nutrient (e.g. vitamin A for oil and sugar, iron for flour and iodine for salt).
**REPORTING**		
44. States how government monitoring results are shared with stakeholders	(2) States how results are shared with stakeholders	NSL presents results to the NFA; Letter of Award is signed by NFA chair. Duplicate copies of the Award and the results are filed and housed in the NSL and the NFA Secretariat for record keeping purposes.

Mexico Wheat

Element	Scoring (use drop-down menus)	Comments (open text)
**GENERAL**		
1. States that legislation applies to at least one food vehicle fit for human consumption (types/grades to be fortified)	(2) States at least one type fit for human consumption	"1.3 Esta Norma Oficial Mexicana establece los nutrimentos que se deben adicionar y restituir en las harinas de trigo y de maíz nixtamalizado y su nivel de adición, exceptuándose las utilizadas para: frituras, como texturizantes o espesantes y base para harinas preparadas.<br>1.4 Esta Norma Oficial Mexicana es de observancia obligatoria en el Territorio Nacional para las personas físicas o morales que se dedican al proceso o importación de los productos objeto de esta Norma destinados a los consumidores en el Territorio Nacional."
2. States the public health objective; purpose and scope of legislation	(0) Does not state	
3. References latest available science or accepted international norms and recommendations, particularly for items that may not be covered in the country's documents	(2) States the documents referenced	"10 Concordancia con normas internacionales<br>Esta norma no es equivalente con normas internacionales o normas mexicanas, excepto el apartado 5.2.2 referente a harinas de cereales, sémolas o semolinas en donde es parcialmente equivalente a:<br>Norma Codex para la harina de trigo. Codex Stan 152-1985 (Rev. 1-1995).<br>11 Bibliografía"
4. Provides definitions that includes terms that are specific to fortification (e.g. fortified food, premix, fortificant, food vehicle)	(2) States at least one term related to fortification	"3. Definiciones"
5. Provides repeals (if there is at least one prior document about fortification)	(2) States repeals	"13 Vigencia<br>...<br>13.2 A su entrada en vigor, la presente norma oficial mexicana cancela las Normas Oficiales Mexicanas:"
6. Provides effective date or gives grace period for when fortification is to begin (e.g. effective 6 months from signing)	(2) States effective date or grace period for when fortification is to begin (e.g. effective 6 months from signing)	"13 Vigencia<br>13.1 La presente Norma Oficial Mexicana entrará en vigor a los ciento ochenta días naturales contados a partir de la fecha de su publicación en el Diario Oficial de la Federación."
**MICRONUTRIENTS/PREMIX**		
7. States nutrients required	(2) States nutrients	"5.2.2.7 Especificaciones nutrimentales<br>i) Las harinas de trigo y de maíz nixtamalizado deben ser restituidas con los siguientes nutrimentos y en los niveles que se indican a continuación. [table with thiamin, riboflavin, niacin]<br>ii) Las harinas de trigo y de maíz nixtamalizado deben ser adicionadas con los siguientes nutrimentos y en los niveles que se indican a continuación. [table with folic acid, iron, zinc]"
8. States fortificants (chemical compounds) to be used (including fortificants that are allowable as options)	(2) States fortificants for at least one nutrient	States for all nutrients.
9. States fortification levels	(1) States one number only	"5.2.2.7 Especificaciones nutrimentales<br>i) Las harinas de trigo y de maíz nixtamalizado deben ser restituidas con los siguientes nutrimentos y en los niveles que se indican a continuación. [table with thiamin, riboflavin, niacin]<br>ii) Las harinas de trigo y de maíz nixtamalizado deben ser adicionadas con los siguientes nutrimentos y en los niveles que se indican a continuación. [table with folic acid, iron, zinc]"
10. States consideration of bioavailability/biological activity of fortificants	(2) States some consideration of bioavailability (mentions these or related terms)	"ii.1) Cuando se utilice sulfato ferroso como fuente de hierro, el aporte debe ser de 31,61% como ión ferroso; si se utiliza fumarato ferroso el aporte será de 31,4%<br>ii.2) Cuando se utilice óxido de zinc como fuente de zinc, el aporte del mismo corresponderá al 79,54%.<br>ii.3) Se podrán utilizar otras fuentes de hierro y zinc, siempre que la cantidad biodisponible sea, al menos, equivalente a la de las fuentes recomendadas."
11. States consideration of nutrient stability	(0) Does not state any consideration	
**COSTING**		
12. States that the cost of fortification is regulated through cost sharing schemes (between government, industry, consumers) or tax measures [to assist industry]	(0) Does not state any consideration	
13. States consideration of the financial responsibility of monitoring and enforcing fortification (schedule of fees, budget) [on the government side]	(0) Does not state any consideration	
**LABELING**		

14. Includes some sort of statement/label/logo that makes it clear that the product is fortified	(2) Includes a statement, label, or logo	"8.14.1 Las harinas de trigo y de maíz nixtamalizado preenvasadas adicionadas con ácido fólico, hierro y zinc y restituidas con vitamina B1, vitamina B2, vitamina B3, deben cumplir con lo siguiente:<br>8.14.2 Sólo podrán utilizar la siguiente denominación:<br>i) Harina de trigo adicionada con ácido fólico o folacina o folato (vitamina Bc o vitamina B9)*, zinc y hierro, restituida con Vitamina B1 (mononitrato de tiamina)*, Vitamina B2 (riboflavina)* y Vitamina B3 (niacina)*.<br>ii) Harina de maíz nixtamalizado adicionada con ácido fólico o folacina o folato (vitamina Bc o vitamina B9)*, hierro y zinc y restituida con Vitamina B1 (mononitrato de tiamina), Vitamina B2 (riboflavina)*, Vitamina B3 (niacina)*.<br>* Los términos entre paréntesis serán opcionales."
15. Provides guidance on health claims that can be made for this product (specific to micronutrients added through fortification)	(0) Does not provide	
**INTERNAL MONITORING (conducted by industry)**		
16. If samples are to be taken, describes the sampling process: for example, number of samples, amount, frequency, individual vs. composite, where samples are taken in the process, and percent considered passing	(N/A) Does not describe the sampling process	
17. States that industry is required to follow quality assurance/quality control in regards to fortification	(2) States requirement of QA/QC for fortification	"iv) Para efectos de control, los establecimientos que procesan harinas de trigo y de maíz nixtamalizado deberán contar con la siguiente información relativa a la restitución y adición de nutrimentos:<br>iv.1) Procedimientos escritos del proceso de restitución y adición y de los controles aplicados para garantizar su eficiencia, incluidas las medidas correctivas que se aplicarán en caso de desviaciones.<br>iv.2) Registro de las variables críticas del proceso que demuestren que se cumplen los procedimientos de restitución y adición, incluyendo reportes de las acciones correctivas aplicadas cuando se detecten desviaciones o incumplimiento de las especificaciones nutrimentales y resultados de análisis de producto terminado (autocontroles)."
18. States applicability of using qualitative testing to determine the presence or absence of a vitamin or mineral (spot tests, iChecks)	(0) Does not state	
**EXTERNAL MONITORING (conducted by government)**		
19.States requirement for external monitoring at the production site to assure compliance with standards and regulations	(0) Does not state requirement	
20. Describes protocols and systems for regulatory monitoring	(0) Does not describe	
21. If there are two or more government agencies involved in external monitoring, clarifies the roles and responsibilities between different government agencies in external monitoring	(0) Clarifies roles and responsibilities for no agencies	
22. Allows for monitoring to be conducted often enough that problems can be identified and addressed on a timely basis; specifies a timeline for inspections (i.e. once every 6 months, increasing to once every 2 months if a discrepancy is found)	(0) Does not state	
23. If samples are to be taken, describes the sampling process: for example, number of samples, amount, frequency, individual vs. composite, where samples are taken in the process, and percent considered passing	(N/A) Does not describe the sampling process	"APENDICE NORMATIVO B. MUESTREO DE CEREALES<br>Generalidades<br>1. El muestreo debe ser realizado por un técnico en muestreo con un instrumento de muestreo que permita obtener la muestra. En el caso de producto en costales, el instrumento debe llegar al centro de cada costal muestreado."
24. States applicability of using qualitative testing to determine the presence or absence of a vitamin or mineral (spot tests, iChecks)	(0) Does not state	
25. States registration is required in order to use a logo/be licensed to produce fortified foods	(0) Does not state that registration or licensing is required	
**COMMERCIAL MONITORING (conducted by government)**		
26. Provides justification for commercial monitoring at retail stores	(0) Does not provide justification for commercial monitoring	
27. Describes protocols and systems for commercial monitoring	(0) Does not describe	
28. If there are two or more government agencies involved in commercial monitoring, clarifies the roles and responsibilities between different government agencies in commercial monitoring	(0) Clarifies roles and responsibilities for no agencies	

29. Allows for monitoring to be conducted often enough that problems at the production site or import companies can be identified and addressed on a timely basis; specifies a timeline for inspections (i.e. once every 6 months) or works with production companies to correct noncompliance	(N/A) No commercial monitoring occurs	
30. If samples are to be taken, describes the sampling process: for example, number of samples, amount, frequency, individual vs. composite, where samples are taken in the process, and percent considered passing	(N/A) Does not describe the sampling process	
**IMPORT MONITORING (conducted by government)**		
31. Provides justification for import monitoring at points of entry	(0) Does not provide justification for import monitoring	
32. Describes protocols and systems for import monitoring	(0) Does not state	
33. If there are two or more government agencies involved in import monitoring, clarifies the roles and responsibilities between different government agencies in import monitoring	(0) Clarifies roles and responsibilities for no agencies	
34. If samples are to be taken, describes the sampling process: for example, number of samples, amount, frequency, individual vs. composite, where samples are taken in the process, and percent considered passing	(N/A) Does not describe the sampling process	
**ENFORCEMENT/PENALTIES**		
35. Indicates roles and responsibilities in enforcing the legislation	(0) Does not state	"APENDICE NORMATIVO B. MUESTREO DE CEREALES … 2.4.1 Las Secretarías están facultadas para efectuar el muestreo en unidades de transporte en cualquier momento y lugar."
36. States incentives to start fortification	(0) Does not state	
37. States incentives to continue fortification, including ensuring compliance	(0) Does not state	
38. States penalties to compel compliance	(0) Does not state	
39. Penalties are objectively defined (e.g. first penalty=$100, second penalty=$300)	(N/A) No penalties are stated (Answered (0) to previous question)	
40. States that enforcement is required to include feedback and support to improve performance and correct noncompliance	(0) Does not require	
**LABORATORY**		
41. References required analytical assays for nutrients (e.g. Liquid chromatography-mass spectrometry for folic acid, atomic absorption for iron and zinc.)	(2) References required assays	"5 Método de prueba para la determinacion de cadmio, plomo, fierro y zinc en productos objeto de esta norma alimentos por espectrometría de absorción atómica.<br>6 Determinación de Vitamina B1 y B2 por Cromatografía Líquida de Alta Resolución (HPLC).<br>7 Determinación de Niacina. Método microbiológico<br>8 Determinación de Acido Fólico. Método microbiológico."
42. States recognition that laboratory results are subject to several sources of variation and do not provide conclusive evidence of compliance or noncompliance	(0) Does not state recognition	
43. Focuses on the quantitative analysis of "marker" micronutrients such as iron	(0) Does not state	
**REPORTING**		
44. States how government monitoring results are shared with stakeholders	(0) Does not state how results are shared	

Morocco Wheat

Element	Scoring (use drop-down menus)	Comments (open text)
**GENERAL**		
1. States that legislation applies to at least one food vehicle fit for human consumption (types/grades to be fortified)	(2) States at least one type fit for human consumption	Flour produced by crushing bread wheat – with the exception of wholegrain flour – produced, packaged, and marketed by the industrial flourmill sector or imported into the national territory must be enriched with a blend of ferrous vitamins, consisting of elementary iron, folic acid, vitamin B1, vitamin B2 and vitamin PP.
2. States the public health objective; purpose and scope of legislation	(2) States the public health objective or general purpose of legislation	talks about ill effects of micronutrient deficiencies
3. References latest available science or accepted international norms and recommendations, particularly for items that may not be covered in the country's documents	(2) States the documents referenced	Preamble, mentions children's summit
4. Provides definitions that includes terms that are specific to fortification (e.g. fortified food, premix, fortificant, food vehicle)	(0) Does not state	
5. Provides repeals (if there is at least one prior document about fortification)	(N/A) No prior documents about fortification	
6. Provides effective date or gives grace period for when fortification is to begin (e.g. effective 6 months from signing)	(2) States effective date or grace period for when fortification is to begin (e.g. effective 6 months from signing)	ART. 5. – Industrial flourmills will have six months, from the date of publication of the above-mentioned joint order, to take the measures necessary to implement enrichment operations for wheat bread flour.
**MICRONUTRIENTS/PREMIX**		
7. States nutrients required	(2) States nutrients	Standards document
8. States fortificants (chemical compounds) to be used (including fortificants that are allowable as options)	(2) States fortificants for at least one nutrient	Standards document, specifies iron types (though not clearly)
9. States fortification levels	(2) States a range or number with +/-	Gives a range for iron (45-65ppm)
10. States consideration of bioavailability/biological activity of fortificants	(2) States some consideration of bioavailability (mentions these or related terms)	bioavailability of antivitamins
11. States consideration of nutrient stability	(2) States consideration of nutrient stability	Shelf life of premix (usually a year from packaging), avoid heat, humidity, sun
**COSTING**		
12. States that the cost of fortification is regulated through cost sharing schemes (between government, industry, consumers) or tax measures [to assist industry]	(0) Does not state any consideration	
13. States consideration of the financial responsibility of monitoring and enforcing fortification (schedule of fees, budget) [on the government side]	(0) Does not state any consideration	
**LABELING**		
14. Includes some sort of statement/label/logo that makes it clear that the product is fortified	(2) Includes a statement, label, or logo	ART. 2. – In addition to the indications provided in the regulations in force regarding labelling, packaging must include a label that indicates:<br>– the term “enriched flour” in very apparent and legible lettering;<br>– the logo for enriched food products, determined by decision of the Minister of Health.<br>In addition, the labelling of imported enriched flour must indicate the country of origin.<br>No indication of therapeutic properties may be included in this label.
15. Provides guidance on health claims that can be made for this product (specific to micronutrients added through fortification)	(2) Provides guidance on health claims specific to micronutrients added through fortification	ART. 2. – In addition to the indications provided in the regulations in force regarding labelling, packaging must include a label that indicates:<br>– the term “enriched flour” in very apparent and legible lettering;<br>– the logo for enriched food products, determined by decision of the Minister of Health.<br>In addition, the labelling of imported enriched flour must indicate the country of origin.<br>No indication of therapeutic properties may be included in this label.
**INTERNAL MONITORING (conducted by industry)**		
16. If samples are to be taken, describes the sampling process: for example, number of samples, amount, frequency, individual vs. composite, where samples are taken in the process, and percent considered passing	(2) States that samples should be taken as part of internal monitoring	page 45, every two hours
17. States that industry is required to follow quality assurance/quality control in regards to fortification	(2) States requirement of QA/QC for fortification	QA/QC described
18. States applicability of using qualitative testing to determine the presence or absence of a vitamin or mineral (spot tests, iChecks)	(2) States applicability of spot test to determine presence/absence of vitamin or mineral specific to internal monitoring	Spot tests
**EXTERNAL MONITORING (conducted by government)**		

19.States requirement for external monitoring at the production site to assure compliance with standards and regulations	(0) Does not state requirement	ART. 3. – Monitoring and verification analyses of the concentration of various components of the iron blend – vitamins and elementary iron in enriched flour – can be carried out at any stage from production to consumption, by agents trained for this purpose pursuant to the provisions of the above-mentioned Law no. 13-83 regarding the prevention of fraudulent merchandise.
20. Describes protocols and systems for regulatory monitoring	(0) Does not describe	
21. If there are two or more government agencies involved in external monitoring, clarifies the roles and responsibilities between different government agencies in external monitoring	(0) Clarifies roles and responsibilities for no agencies	
22. Allows for monitoring to be conducted often enough that problems can be identified and addressed on a timely basis; specifies a timeline for inspections (i.e. once every 6 months, increasing to once every 2 months if a discrepancy is found)	(0) Does not state	
23. If samples are to be taken, describes the sampling process: for example, number of samples, amount, frequency, individual vs. composite, where samples are taken in the process, and percent considered passing	(N/A) Does not describe the sampling process	
24. States applicability of using qualitative testing to determine the presence or absence of a vitamin or mineral (spot tests, iChecks)	(0) Does not state	
25. States registration is required in order to use a logo/be licensed to produce fortified foods	(0) Does not state that registration or licensing is required	
**COMMERCIAL MONITORING (conducted by government)**		
26. Provides justification for commercial monitoring at retail stores	(0) Does not provide justification for commercial monitoring	ART. 3. – Monitoring and verification analyses of the concentration of various components of the iron blend – vitamins and elementary iron in enriched flour – can be carried out at any stage from production to consumption, by agents trained for this purpose pursuant to the provisions of the above-mentioned Law no. 13-83 regarding the prevention of fraudulent merchandise.
27. Describes protocols and systems for commercial monitoring	(0) Does not describe	
28. If there are two or more government agencies involved in commercial monitoring, clarifies the roles and responsibilities between different government agencies in commercial monitoring	(0) Clarifies roles and responsibilities for no agencies	
29. Allows for monitoring to be conducted often enough that problems at the production site or import companies can be identified and addressed on a timely basis; specifies a timeline for inspections (i.e. once every 6 months) or works with production companies to correct noncompliance	(N/A) No commercial monitoring occurs	
30. If samples are to be taken, describes the sampling process: for example, number of samples, amount, frequency, individual vs. composite, where samples are taken in the process, and percent considered passing	(N/A) Does not describe the sampling process	
**IMPORT MONITORING (conducted by government)**		
31. Provides justification for import monitoring at points of entry	(0) Does not provide justification for import monitoring	ART. 3. – Monitoring and verification analyses of the concentration of various components of the iron blend – vitamins and elementary iron in enriched flour – can be carried out at any stage from production to consumption, by agents trained for this purpose pursuant to the provisions of the above-mentioned Law no. 13-83 regarding the prevention of fraudulent merchandise.
32. Describes protocols and systems for import monitoring	(0) Does not state	
33. If there are two or more government agencies involved in import monitoring, clarifies the roles and responsibilities between different government agencies in import monitoring	(0) Clarifies roles and responsibilities for no agencies	
34. If samples are to be taken, describes the sampling process: for example, number of samples, amount, frequency, individual vs. composite, where samples are taken in the process, and percent considered passing	(N/A) Does not describe the sampling process	
**ENFORCEMENT/PENALTIES**		
35. Indicates roles and responsibilities in enforcing the legislation	(0) Does not state	
36. States incentives to start fortification	(0) Does not state	
37. States incentives to continue fortification, including ensuring compliance	(0) Does not state	
38. States penalties to compel compliance	(0) Does not state	
39. Penalties are objectively defined (e.g. first penalty=$100, second penalty=$300)	(N/A) No penalties are stated (Answered (0) to previous question)	

40. States that enforcement is required to include feedback and support to improve performance and correct noncompliance	(0) Does not require	
**LABORATORY**		
41. References required analytical assays for nutrients (e.g. Liquid chromatography-mass spectrometry for folic acid, atomic absorption for iron and zinc.)	(2) References required assays	HPLC for iron and vitamin B2
42. States recognition that laboratory results are subject to several sources of variation and do not provide conclusive evidence of compliance or noncompliance	(0) Does not state recognition	
43. Focuses on the quantitative analysis of "marker" micronutrients such as iron	(2) Focuses on quantitative analysis of marker micronutrient such as iron	just iron and B2
**REPORTING**		
44. States how government monitoring results are shared with stakeholders	(0) Does not state how results are shared	

Nicaragua Wheat

Element	Scoring (use drop-down menus)	Comments (open text)
**GENERAL**		
1. States that legislation applies to at least one food vehicle fit for human consumption (types/grades to be fortified)	(2) States at least one type fit for human consumption	"2. AMBITO DE APLICACIÓN<br>El presente reglamento se aplica a la harina de trigo fortificada para el consumo humano, elaborada con trigo común, Triticum aestivum L. o con trigo ramificado, Triticum compactum Host, o una mezcla de los mismos, a granel o preenvasada y que está lista para la venta al consumidor o esta destinada para utilizarla en la elaboración de otros productos alimenticios."
2. States the public health objective; purpose and scope of legislation	(0) Does not state	
3. References latest available science or accepted international norms and recommendations, particularly for items that may not be covered in the country's documents	(0) Does not state	
4. Provides definitions that includes terms that are specific to fortification (e.g. fortified food, premix, fortificant, food vehicle)	(2) States at least one term related to fortification	"3. DEFINICIONES"
5. Provides repeals (if there is at least one prior document about fortification)	(0) Does not state	
6. Provides effective date or gives grace period for when fortification is to begin (e.g. effective 6 months from signing)	(0) Does not state	
**MICRONUTRIENTS/PREMIX**		
7. States nutrients required	(2) States nutrients	"4.7 Fortificación de la harina de trigo"
8. States fortificants (chemical compounds) to be used (including fortificants that are allowable as options)	(2) States fortificants for at least one nutrient	"4.7.2 La fuente de hierro a utilizar en la fortificación debe ser fumarato ferroso."
9. States fortification levels	(1) States one number only	4.7.1 "Nivel mínimo a alcanzar (mg/kg de harina)"
10. States consideration of bioavailability/biological activity of fortificants	(0) Does not state any consideration	
11. States consideration of nutrient stability	(0) Does not state any consideration	
**COSTING**		
12. States that the cost of fortification is regulated through cost sharing schemes (between government, industry, consumers) or tax measures [to assist industry]	(0) Does not state any consideration	
13. States consideration of the financial responsibility of monitoring and enforcing fortification (schedule of fees, budget) [on the government side]	(0) Does not state any consideration	
**LABELING**		
14. Includes some sort of statement/label/logo that makes it clear that the product is fortified	(0) Does not include statement, label, or logo	
15. Provides guidance on health claims that can be made for this product (specific to micronutrients added through fortification)	(2) Provides guidance on health claims specific to micronutrients added through fortification	"7.1 Declaraciones Nutricionales<br>Las únicas declaraciones de propiedades nutricionales permitidas deben ser las que se refieran a energía, proteínas, carbohidratos, grasas y los componentes de las mismas, fibra, vitaminas y minerales para los cuales se hayan establecido recomendaciones nutricionales."<br>"7.4 Declaraciones de Propiedades Saludables"<br>Anexo G "3.9 Ácido fólico y defectos del conducto (tubo) neural:"
**INTERNAL MONITORING (conducted by industry)**		
16. If samples are to be taken, describes the sampling process: for example, number of samples, amount, frequency, individual vs. composite, where samples are taken in the process, and percent considered passing	(N/A) Does not describe the sampling process	
17. States that industry is required to follow quality assurance/quality control in regards to fortification	(0) Does not state requirement	
18. States applicability of using qualitative testing to determine the presence or absence of a vitamin or mineral (spot tests, iChecks)	(0) Does not state	
**EXTERNAL MONITORING (conducted by government)**		
19.States requirement for external monitoring at the production site to assure compliance with standards and regulations	(0) Does not state requirement	
20. Describes protocols and systems for regulatory monitoring	(0) Does not describe	
21. If there are two or more government agencies involved in external monitoring, clarifies the roles and responsibilities between different government agencies in external monitoring	(0) Clarifies roles and responsibilities for no agencies	
22. Allows for monitoring to be conducted often enough that problems can be identified and addressed on a timely basis; specifies a timeline for inspections (i.e. once every 6 months, increasing to once every 2 months if a discrepancy is found)	(0) Does not state	

23. If samples are to be taken, describes the sampling process: for example, number of samples, amount, frequency, individual vs. composite, where samples are taken in the process, and percent considered passing	(N/A) Does not describe the sampling process	
24. States applicability of using qualitative testing to determine the presence or absence of a vitamin or mineral (spot tests, iChecks)	(0) Does not state	
25. States registration is required in order to use a logo/be licensed to produce fortified foods	(0) Does not state that registration or licensing is required	
**COMMERCIAL MONITORING (conducted by government)**		
26. Provides justification for commercial monitoring at retail stores	(0) Does not provide justification for commercial monitoring	
27. Describes protocols and systems for commercial monitoring	(0) Does not describe	
28. If there are two or more government agencies involved in commercial monitoring, clarifies the roles and responsibilities between different government agencies in commercial monitoring	(0) Clarifies roles and responsibilities for no agencies	
29. Allows for monitoring to be conducted often enough that problems at the production site or import companies can be identified and addressed on a timely basis; specifies a timeline for inspections (i.e. once every 6 months) or works with production companies to correct noncompliance	(N/A) No commercial monitoring occurs	
30. If samples are to be taken, describes the sampling process: for example, number of samples, amount, frequency, individual vs. composite, where samples are taken in the process, and percent considered passing	(N/A) Does not describe the sampling process	
**IMPORT MONITORING (conducted by government)**		
31. Provides justification for import monitoring at points of entry	(0) Does not provide justification for import monitoring	
32. Describes protocols and systems for import monitoring	(0) Does not state	
33. If there are two or more government agencies involved in import monitoring, clarifies the roles and responsibilities between different government agencies in import monitoring	(0) Clarifies roles and responsibilities for no agencies	
34. If samples are to be taken, describes the sampling process: for example, number of samples, amount, frequency, individual vs. composite, where samples are taken in the process, and percent considered passing	(N/A) Does not describe the sampling process	
**ENFORCEMENT/PENALTIES**		
35. Indicates roles and responsibilities in enforcing the legislation	(2) States the role and responsibilities of government in enforcement	(Ref 1) Generic: "8. VIGILANCIA Y VERIFICACIÓN La vigilancia y verificación de este Reglamento Técnico Centroamericano les corresponde a las Autoridades Competentes de cada país de la Región Centroamericana."
36. States incentives to start fortification	(0) Does not state	
37. States incentives to continue fortification, including ensuring compliance	(0) Does not state	
38. States penalties to compel compliance	(0) Does not state	
39. Penalties are objectively defined (e.g. first penalty=$100, second penalty=$300)	(N/A) No penalties are stated (Answered (0) to previous question)	
40. States that enforcement is required to include feedback and support to improve performance and correct noncompliance	(0) Does not require	
**LABORATORY**		
41. References required analytical assays for nutrients (e.g. Liquid chromatography-mass spectrometry for folic acid, atomic absorption for iron and zinc.)	(2) References required assays	"7. MÉTODOS DE ANÁLISIS" "Determinación del hierro AOAC 32.1.09.17 Determinación de niacina AOAC 45.1.10.17 Determinación de ácido fólico AOAC 45.2.01.17 Determinación de Vitamina B1 AOAC 957.17 Determinación de Vitamina B2 AOAC 970.65"
42. States recognition that laboratory results are subject to several sources of variation and do not provide conclusive evidence of compliance or noncompliance	(0) Does not state recognition	
43. Focuses on the quantitative analysis of "marker" micronutrients such as iron	(0) Does not state	
**REPORTING**		
44. States how government monitoring results are shared with stakeholders	(0) Does not state how results are shared	

Nigeria Wheat

Element	Scoring (use drop-down menus)	Comments (open text)
**GENERAL**		
1. States that legislation applies to at least one food vehicle fit for human consumption (types/grades to be fortified)	(2) States at least one type fit for human consumption	…requirements for wheat flour and composite wheat flour (10% cassava flour inclusion)
2. States the public health objective; purpose and scope of legislation	(2) States the public health objective or general purpose of legislation	The standard is reviewed to further improve on the control of micronutrient deficiency in Nigeria…and promote quality, safe, healthy, and better nutrition for consumers in line with the National Policy on Nutrition
3. References latest available science or accepted international norms and recommendations, particularly for items that may not be covered in the country's documents	(2) States the documents referenced	The Codex Alimentarius Commission…[was] referenced and such information [is] hereby duly acknowledged. -->Section on Normative References
4. Provides definitions that includes terms that are specific to fortification (e.g. fortified food, premix, fortificant, food vehicle)	(2) States at least one term related to fortification	NIS 475 defines fortificant, food vehicle, micronutrient, etc.
5. Provides repeals (if there is at least one prior document about fortification)	(2) States repeals	This version replaces the 2010 version
6. Provides effective date or gives grace period for when fortification is to begin (e.g. effective 6 months from signing)	(0) Does not state	2000-2002?
**MICRONUTRIENTS/PREMIX**		
7. States nutrients required	(2) States nutrients	4.1 of NIS 121, Table 1
8. States fortificants (chemical compounds) to be used (including fortificants that are allowable as options)	(2) States fortificants for at least one nutrient	4.1 of NIS 121, Table 1
9. States fortification levels	(1) States one number only	4.1 of NIS 121, Table 1
10. States consideration of bioavailability/biological activity of fortificants	(2) States some consideration of bioavailability (mentions these or related terms)	The electrolytic iron as fortificant is replaced with NaFeEDTA that is more bioavailable.
11. States consideration of nutrient stability	(2) States consideration of nutrient stability	1.3 Fortificant is stored under suitable conditions and is used on the "first-in, first-out" basis.
**COSTING**		
12. States that the cost of fortification is regulated through cost sharing schemes (between government, industry, consumers) or tax measures [to assist industry]	(0) Does not state any consideration	
13. States consideration of the financial responsibility of monitoring and enforcing fortification (schedule of fees, budget) [on the government side]	(0) Does not state any consideration	
**LABELING**		
14. Includes some sort of statement/label/logo that makes it clear that the product is fortified	(2) Includes a statement, label, or logo	NIS475 says labeling must include premix composition and name of product Labelling of Vitamin A fortified foods, which include an Eye Logo for its identification,
15. Provides guidance on health claims that can be made for this product (specific to micronutrients added through fortification)	(0) Does not provide	
**INTERNAL MONITORING (conducted by industry)**		
16. If samples are to be taken, describes the sampling process: for example, number of samples, amount, frequency, individual vs. composite, where samples are taken in the process, and percent considered passing	(2) States that samples should be taken as part of internal monitoring	3.3.1a Shift composite samples are taken from the packaging line. 200g samples are taken every 30 minutes. 8 consecutive samples constitute one shift composite sample. (By QC/QA Department)
17. States that industry is required to follow quality assurance/quality control in regards to fortification	(2) States requirement of QA/QC for fortification	Figure 2.1 Internal Monitoring (factories or packers) QA/QC by the Company's Dept. of Quality Control. Outline further in later tables.
18. States applicability of using qualitative testing to determine the presence or absence of a vitamin or mineral (spot tests, iChecks)	(2) States applicability of spot test to determine presence/absence of vitamin or mineral specific to internal monitoring	Quality assurance of vitamin A and iron in fortificants 3.1 Spot density in flour samples for iron is comparable to flour standards containing the expected factory minimum, average and maximum iron levels. (completed by company's QA/QC department) 3.1b Use the iron spot test on the shift composite sample.
**EXTERNAL MONITORING (conducted by government)**		
19.States requirement for external monitoring at the production site to assure compliance with standards and regulations	(2) States requirement for external monitoring or the need for audits/inspections	The product shall sample in compliance with any of the following sampling methods:
20. Describes protocols and systems for regulatory monitoring	(2) Includes checklists or provides detailed description of regulatory monitoring procedures	Sampling at the mill shall be on not less than six (6) containers. If the sampling is from a millstream, an interval between each taking shall be 10-15 minutes. The micronutrients shall be analyzed using the test methods specified in table 2 of NIS:475:2015 Standard for Fortificant Premix of any Internationally acceptable Standard methods.
21. If there are two or more government agencies involved in external monitoring, clarifies the roles and responsibilities between different government agencies in external monitoring	(0) Clarifies roles and responsibilities for no agencies	Table 4.1: NAFDAC and SON, but doesn't give roles and responsibilities

22. Allows for monitoring to be conducted often enough that problems can be identified and addressed on a timely basis; specifies a timeline for inspections (i.e. once every 6 months, increasing to once every 2 months if a discrepancy is found)	(1) Makes mention of a timeline	Table
23. If samples are to be taken, describes the sampling process: for example, number of samples, amount, frequency, individual vs. composite, where samples are taken in the process, and percent considered passing	(2) States that samples should be taken as part of external monitoring	Sampling at the mill shall be on not less than six (6) containers. If the sampling is from a millstream, an interval between each taking shall be 10-15 minutes. The micronutrients shall be analyzed using the test methods specified in table 2 of NIS:475:2015 Standard for Fortificant Premix of any Internationally acceptable Standard methods. See Table 8.
24. States applicability of using qualitative testing to determine the presence or absence of a vitamin or mineral (spot tests, iChecks)	(2) States applicability of spot test to determine presence/absence of vitamin or mineral specific to external monitoring	In 2012, UNICEF and GAIN donated i-Check equipments a vitamin A test kits to NAFDAC to be used for semi quantitative spot analysis of food products (flour, vegetable oil and sugar). Both NAFDAC and the industry were trained by GAIN on the use of i-Check equipment.
25. States registration is required in order to use a logo/be licensed to produce fortified foods	(2) Describes some type of registration or licensing	Denominator: Total number of registered wheat/maize industries in the Country
**COMMERCIAL MONITORING (conducted by government)**		
26. Provides justification for commercial monitoring at retail stores	(2) Provides justification for commercial monitoring	Figure 2.1: Commercial monitoring (at distribution and retail stores)-verification of legal compliance (NAFDAC) (orange book document)
27. Describes protocols and systems for commercial monitoring	(0) Does not describe	Barely any mention of commercial monitoring beyond Figure 2.1
28. If there are two or more government agencies involved in commercial monitoring, clarifies the roles and responsibilities between different government agencies in commercial monitoring	(0) Clarifies roles and responsibilities for no agencies	NAFDAC (Figure 2.1)--doesn't state roles and responsibilities
29. Allows for monitoring to be conducted often enough that problems at the production site or import companies can be identified and addressed on a timely basis; specifies a timeline for inspections (i.e. once every 6 months) or works with production companies to correct noncompliance	(N/A) No commercial monitoring occurs	Barely any mention of commercial monitoring beyond Figure 2.1
30. If samples are to be taken, describes the sampling process: for example, number of samples, amount, frequency, individual vs. composite, where samples are taken in the process, and percent considered passing	(N/A) Does not describe the sampling process	Barely any mention of commercial monitoring beyond Figure 2.1
**IMPORT MONITORING (conducted by government)**		
31. Provides justification for import monitoring at points of entry	(2) Provides justification for import monitoring	Certification procedure: Certificate of conformity or inspection, COA (NAFDAC and Customs)
32. Describes protocols and systems for import monitoring	(1) Does not explicitly state, but references protocols and systems for import monitoring	Certificate of conformity from country of origin; fortified with Iron to standard imported into the country
33. If there are two or more government agencies involved in import monitoring, clarifies the roles and responsibilities between different government agencies in import monitoring	(0) Clarifies roles and responsibilities for no agencies	Certificate of conformity or inspection, COA (NAFDAC and Customs)--doesn't state roles and responsibilities
34. If samples are to be taken, describes the sampling process: for example, number of samples, amount, frequency, individual vs. composite, where samples are taken in the process, and percent considered passing	(N/A) Does not describe the sampling process	
**ENFORCEMENT/PENALTIES**		
35. Indicates roles and responsibilities in enforcing the legislation	(0) Does not state	
36. States incentives to start fortification	(0) Does not state	
37. States incentives to continue fortification, including ensuring compliance	(0) Does not state	
38. States penalties to compel compliance	(0) Does not state	Enforcement is mentioned in passing, but never stated explicitly nor in relation to wheat flour fortification (salt only)
39. Penalties are objectively defined (e.g. first penalty=$100, second penalty=$300)	(N/A) No penalties are stated (Answered (0) to previous question)	
40. States that enforcement is required to include feedback and support to improve performance and correct noncompliance	(0) Does not require	
**LABORATORY**		
41. References required analytical assays for nutrients (e.g. Liquid chromatography-mass spectrometry for folic acid, atomic absorption for iron and zinc.)	(2) References required assays	The micronutrients shall be analyzed using the test methods specified in Table 2 of NIS 475:2015 or any internationally acceptable Standard methods.
42. States recognition that laboratory results are subject to several sources of variation and do not provide conclusive evidence of compliance or noncompliance	(0) Does not state recognition	

43. Focuses on the quantitative analysis of "marker" micronutrients such as iron	(2) Focuses on quantitative analysis of marker micronutrient such as iron	3.1 Spot density in flour samples for iron is comparable to flour standards containing the expected factory minimum, average and maximum iron levels. 3.2 Random sample of two daily-composite samples are taken and added to monthly composite samples and analysed for quantitative determination of iron and vitamin A using i-Check.
**REPORTING**		
44. States how government monitoring results are shared with stakeholders	(2) States how results are shared with stakeholders	The food fortification results framework provides an overview of all fortification activities within the country supported through various partners, regulators and stakeholders. The results framework depicts the linkage of activities on food fortification by various key players, key targets, technical areas and expected outcomes over the lifetime of the programme. Efforts have been made to ensure the inclusion of key impact and outcome indicators as well as the documentation of process indicators (input and output). The M&E system will ensure project-wide monitoring and that all indicators listed on the results framework are tracked and reported accordingly. The measurement and analysis and reporting of the indicators would enable all stakeholders to track progress, demonstrate results and take corrective actions where necessary to improve on the delivery of appropriate food fortification to improve the health status of the population.

Palestine Occupied Territory Wheat

Element	Scoring (use drop-down menus)	Comments (open text)
**GENERAL**		
1. States that legislation applies to at least one food vehicle fit for human consumption (types/grades to be fortified)	(2) States at least one type fit for human consumption	Wheat flour
2. States the public health objective; purpose and scope of legislation	(0) Does not state	
3. References latest available science or accepted international norms and recommendations, particularly for items that may not be covered in the country's documents	(0) Does not state	
4. Provides definitions that includes terms that are specific to fortification (e.g. fortified food, premix, fortificant, food vehicle)	(0) Does not state	
5. Provides repeals (if there is at least one prior document about fortification)	(N/A) No prior documents about fortification	
6. Provides effective date or gives grace period for when fortification is to begin (e.g. effective 6 months from signing)	(0) Does not state	
**MICRONUTRIENTS/PREMIX**		
7. States nutrients required	(2) States nutrients	Folate, iron, B12, B1, B2, B6, Niacin, Zinc, Vitamin A, Vitamin D
8. States fortificants (chemical compounds) to be used (including fortificants that are allowable as options)	(2) States fortificants for at least one nutrient	folic acid, ferroud sulfate dried, vitamin B12 0.1% WS, thiamin mononitrate, riboflavin, pyridoxin, niacinamide, zinc oxide, vitamin A palm., vitamin D3
9. States fortification levels	(2) States a range or number with +/-	Min. addition level, average addition level, maximum tolerance level
10. States consideration of bioavailability/biological activity of fortificants	(0) Does not state any consideration	
11. States consideration of nutrient stability	(0) Does not state any consideration	Requires labeling to state expiration date and storage conditions...does this count?
**COSTING**		
12. States that the cost of fortification is regulated through cost sharing schemes (between government, industry, consumers) or tax measures [to assist industry]	(0) Does not state any consideration	
13. States consideration of the financial responsibility of monitoring and enforcing fortification (schedule of fees, budget) [on the government side]	(0) Does not state any consideration	
**LABELING**		
14. Includes some sort of statement/label/logo that makes it clear that the product is fortified	(2) Includes a statement, label, or logo	Colored fortification logo should be printed in labeling
15. Provides guidance on health claims that can be made for this product (specific to micronutrients added through fortification)	(0) Does not provide	
**INTERNAL MONITORING (conducted by industry)**		
16. If samples are to be taken, describes the sampling process: for example, number of samples, amount, frequency, individual vs. composite, where samples are taken in the process, and percent considered passing	(N/A) Does not describe the sampling process	"Sampling: same as drug"
17. States that industry is required to follow quality assurance/quality control in regards to fortification	(0) Does not state requirement	
18. States applicability of using qualitative testing to determine the presence or absence of a vitamin or mineral (spot tests, iChecks)	(0) Does not state	
**EXTERNAL MONITORING (conducted by government)**		
19.States requirement for external monitoring at the production site to assure compliance with standards and regulations	(0) Does not state requirement	
20. Describes protocols and systems for regulatory monitoring	(0) Does not describe	
21. If there are two or more government agencies involved in external monitoring, clarifies the roles and responsibilities between different government agencies in external monitoring	(0) Clarifies roles and responsibilities for no agencies	
22. Allows for monitoring to be conducted often enough that problems can be identified and addressed on a timely basis; specifies a timeline for inspections (i.e. once every 6 months, increasing to once every 2 months if a discrepancy is found)	(0) Does not state	
23. If samples are to be taken, describes the sampling process: for example, number of samples, amount, frequency, individual vs. composite, where samples are taken in the process, and percent considered passing	(N/A) Does not describe the sampling process	
24. States applicability of using qualitative testing to determine the presence or absence of a vitamin or mineral (spot tests, iChecks)	(0) Does not state	

25. States registration is required in order to use a logo/be licensed to produce fortified foods	(2) Describes some type of registration or licensing	The Importer or Premix Agent should registrate the Premix before using or marketing.
**COMMERCIAL MONITORING (conducted by government)**		
26. Provides justification for commercial monitoring at retail stores	(0) Does not provide justification for commercial monitoring	
27. Describes protocols and systems for commercial monitoring	(0) Does not describe	
28. If there are two or more government agencies involved in commercial monitoring, clarifies the roles and responsibilities between different government agencies in commercial monitoring	(0) Clarifies roles and responsibilities for no agencies	
29. Allows for monitoring to be conducted often enough that problems at the production site or import companies can be identified and addressed on a timely basis; specifies a timeline for inspections (i.e. once every 6 months) or works with production companies to correct noncompliance	(N/A) No commercial monitoring occurs	
30. If samples are to be taken, describes the sampling process: for example, number of samples, amount, frequency, individual vs. composite, where samples are taken in the process, and percent considered passing	(N/A) Does not describe the sampling process	
**IMPORT MONITORING (conducted by government)**		
31. Provides justification for import monitoring at points of entry	(2) Provides justification for import monitoring	Only for premix - page 3
32. Describes protocols and systems for import monitoring	(1) Does not explicitly state, but references protocols and systems for import monitoring	Only for premix - page 3
33. If there are two or more government agencies involved in import monitoring, clarifies the roles and responsibilities between different government agencies in import monitoring	(0) Clarifies roles and responsibilities for no agencies	
34. If samples are to be taken, describes the sampling process: for example, number of samples, amount, frequency, individual vs. composite, where samples are taken in the process, and percent considered passing	(1) States that samples should be taken (generally)	Only for premix - page 3
**ENFORCEMENT/PENALTIES**		
35. Indicates roles and responsibilities in enforcing the legislation	(0) Does not state	
36. States incentives to start fortification	(0) Does not state	
37. States incentives to continue fortification, including ensuring compliance	(0) Does not state	
38. States penalties to compel compliance	(0) Does not state	
39. Penalties are objectively defined (e.g. first penalty=$100, second penalty=$300)	(N/A) No penalties are stated (Answered (0) to previous question)	
40. States that enforcement is required to include feedback and support to improve performance and correct noncompliance	(0) Does not require	
**LABORATORY**		
41. References required analytical assays for nutrients (e.g. Liquid chromatography-mass spectrometry for folic acid, atomic absorption for iron and zinc.)	(0) Does not state requirements	
42. States recognition that laboratory results are subject to several sources of variation and do not provide conclusive evidence of compliance or noncompliance	(0) Does not state recognition	
43. Focuses on the quantitative analysis of "marker" micronutrients such as iron	(0) Does not state	
**REPORTING**		
44. States how government monitoring results are shared with stakeholders	(0) Does not state how results are shared	

Panama Wheat

Element	Scoring (use drop-down menus)	Comments (open text)
**GENERAL**		
1. States that legislation applies to at least one food vehicle fit for human consumption (types/grades to be fortified)	(2) States at least one type fit for human consumption	Considerando
2. States the public health objective; purpose and scope of legislation	(2) States the public health objective or general purpose of legislation	"CONSIDERANDO: …Que la anemia nutricional constituye la deficiencia específica de micronutrientes, más común en nuestro país…Que las anomalías congénitas constituyen una de las principales cuasas de muerte, en los menores de un año…"
3. References latest available science or accepted international norms and recommendations, particularly for items that may not be covered in the country's documents	(0) Does not state	
4. Provides definitions that includes terms that are specific to fortification (e.g. fortified food, premix, fortificant, food vehicle)	(0) Does not state	
5. Provides repeals (if there is at least one prior document about fortification)	(2) States repeals	Artículo 6
6. Provides effective date or gives grace period for when fortification is to begin (e.g. effective 6 months from signing)	(2) States effective date or grace period for when fortification is to begin (e.g. effective 6 months from signing)	Artículo 5
**MICRONUTRIENTS/PREMIX**		
7. States nutrients required	(2) States nutrients	Artículo 1
8. States fortificants (chemical compounds) to be used (including fortificants that are allowable as options)	(2) States fortificants for at least one nutrient	"Artículo 2. El tipo de hierro que se deberá agregar es el fumarato ferroso, en un nivel de 45 mg/Kg. Sin embargo, este compuesto y cualquier otro de los micronutrientes, podrán ser modificados por el Ministerio de Salud, si así lo recomiendan los oganismos públicos, nacionales e internacionales."
9. States fortification levels	(1) States one number only	Artículo 1. Minimum levels.
10. States consideration of bioavailability/biological activity of fortificants	(0) Does not state any consideration	
11. States consideration of nutrient stability	(0) Does not state any consideration	
**COSTING**		
12. States that the cost of fortification is regulated through cost sharing schemes (between government, industry, consumers) or tax measures [to assist industry]	(0) Does not state any consideration	
13. States consideration of the financial responsibility of monitoring and enforcing fortification (schedule of fees, budget) [on the government side]	(2) Shows consideration that monitoring costs money	"A. Planificación de la visitas de inspección<br>1. Objetivos y responsabilidad:<br>El propósito de la planificación de las visitas de inspección es asegurar que:<br>Se designen los recursos para visitar los molinos de trigo por lo menos dos veces al año[1].<br>Los inspectores reciben capacitación apropiada en el proceso de fortificación y muestreo para realizar las actividades de auditoría e inspección."<br>"[1.] Cuando se inicien las actividades de inspección las visitas deben ser más frecuentes, por lo menos vez cada tres meses. Cuando se demuestre que el proceso está controlado y los resultados son satisfactorios las visitas se pueden espaciar. Debido a que esta actividad está sujeta a presupuesto anual, estos gastos se asignan en el rubro de imprevistos."
**LABELING**		
14. Includes some sort of statement/label/logo that makes it clear that the product is fortified	(0) Does not include statement, label, or logo	
15. Provides guidance on health claims that can be made for this product (specific to micronutrients added through fortification)	(0) Does not provide	
**INTERNAL MONITORING (conducted by industry)**		
16. If samples are to be taken, describes the sampling process: for example, number of samples, amount, frequency, individual vs. composite, where samples are taken in the process, and percent considered passing	(2) States that samples should be taken as part of internal monitoring	C. Control de calidad de la harina fortificada
17. States that industry is required to follow quality assurance/quality control in regards to fortification	(2) States requirement of QA/QC for fortification	"1. Monitore interno Los productores e importadores de harina de trigo son responsables de cumplir con las regulaciones de fortificación de harina de trigo en el país donde ésta se comercializa. La fortificación de harina de trigo con vitaminas y minerales requiere la implementación de actividades de aseguramiento y control de calidad para asegurar que la harina fortificada satisface los requisitos establecidos en los reglamentos y normas."

18. States applicability of using qualitative testing to determine the presence or absence of a vitamin or mineral (spot tests, iChecks)	(2) States applicability of spot test to determine presence/absence of vitamin or mineral specific to internal monitoring	C. Control de calidad de la harina fortificada
**EXTERNAL MONITORING (conducted by government)**		
19.States requirement for external monitoring at the production site to assure compliance with standards and regulations	(2) States requirement for external monitoring or the need for audits/inspections	"2. Monitoreo externo<br>La auditoría técnica e inspección de la fortificación de las harinas de trigo es realizada por el Departamento de Protección de Alimentos (DEPA) del Ministerio de Salud... Las actividades de auditoría técnica e inspección realizadas en los molinos de trigo son parte de las actividades de verificación del cumplimiento de la ley realizadas por el Departamento de Protección de Alimentos (DEPA), para asegurar que la harina de trigo satisface la calidad de nutrientes, como también las especificaciones de inocuidad establecidas en las normas y regulaciones."
20. Describes protocols and systems for regulatory monitoring	(2) Includes checklists or provides detailed description of regulatory monitoring procedures	"Procedimiento para realizar las tomas de muestras de harinas de trigo, premezclas y pan en las panaderías, molinos u otros establecimientos:"
21. If there are two or more government agencies involved in external monitoring, clarifies the roles and responsibilities between different government agencies in external monitoring	(2) Clarifies roles and responsibilities for more than one agency	"Es el nivel nacional del Departamento de Protección de Alimentos en la cual se realiza la coordinación con el Instituto Conmemorativo Gorgas de estudios a la Salud ICGE o con el IEA Instituto Especializado de Análisis de la Universidad de Panamá a través del Programa de Alimentos Diversos de la sección de Inspección Nacional de Plantas (INPLA). Esta sección elabora la programación anual de muestras de las harinas fortificadas a tomar y analizar. El programa anual es enviado a los niveles regionales."
22. Allows for monitoring to be conducted often enough that problems can be identified and addressed on a timely basis; specifies a timeline for inspections (i.e. once every 6 months, increasing to once every 2 months if a discrepancy is found)	(1) Makes mention of a timeline	"A. Planificación de la visitas de inspección<br>1. Objetivos y responsabilidad:<br>El propósito de la planificación de las visitas de inspección es asegurar que:<br>Se designen los recursos para visitar los molinos de trigo por lo menos dos veces al año[1].<br>Los inspectores reciben capacitación apropiada en el proceso de fortificación y muestreo para realizar las actividades de auditoría e inspección."<br>"[1.] Cuando se inicien las actividades de inspección las visitas deben ser más frecuentes, por lo menos vez cada tres meses. Cuando se demuestre que el proceso está controlado y los resultados son satisfactorios las visitas se pueden espaciar. Debido a que esta actividad está sujeta a presupuesto anual, estos gastos se asignan en el rubro de imprevistos."
23. If samples are to be taken, describes the sampling process: for example, number of samples, amount, frequency, individual vs. composite, where samples are taken in the process, and percent considered passing	(2) States that samples should be taken as part of external monitoring	Artículo 3
24. States applicability of using qualitative testing to determine the presence or absence of a vitamin or mineral (spot tests, iChecks)	(2) States applicability of spot test to determine presence/absence of vitamin or mineral specific to external monitoring	"ANEXO 1<br>Método de manchas para la determinación cualitativa del hierro agregado a la harina de trigo fortificada"
25. States registration is required in order to use a logo/be licensed to produce fortified foods	(0) Does not state that registration or licensing is required	
**COMMERCIAL MONITORING (conducted by government)**		
26. Provides justification for commercial monitoring at retail stores	(2) Provides justification for commercial monitoring	"b. Monitoreo Comercial:<br>Recomendaciones generales para el muestreo y seguimiento de resultados fuera de norma:<br>Se realizará el muestreo de las harinas y premezcla en todas las regiones de salud a nivel de molinos y panaderías."
27. Describes protocols and systems for commercial monitoring	(2) Includes checklists or provides detailed description of regulatory monitoring procedures	"Procedimiento para realizar las tomas de muestras de harinas de trigo, premezclas y pan en las panaderías, molinos u otros establecimientos:"
28. If there are two or more government agencies involved in commercial monitoring, clarifies the roles and responsibilities between different government agencies in commercial monitoring	(2) Clarifies roles and responsibilities for more than one agency	Same as those for external monitoring.
29. Allows for monitoring to be conducted often enough that problems at the production site or import companies can be identified and addressed on a timely basis; specifies a timeline for inspections (i.e. once every 6 months) or works with production companies to correct noncompliance	(1) Makes mention of a timeline	Same as for external monitoring.

30. If samples are to be taken, describes the sampling process: for example, number of samples, amount, frequency, individual vs. composite, where samples are taken in the process, and percent considered passing	(2) States that samples should be taken as part of commercial monitoring	(Ref 1) Artículo 3. (Ref 2) "Las muestras deben ser tomadas aplicando los procedimientos detallados en el presente documento y utilizando el formato: De toma de muestras de alimentos"
**IMPORT MONITORING (conducted by government)**		
31. Provides justification for import monitoring at points of entry	(2) Provides justification for import monitoring	"1. Monitore interno Los productores e importadores de harina de trigo son responsables de cumplir con las regulaciones de fortificación de harina de trigo en el país donde ésta se comercializa."
32. Describes protocols and systems for import monitoring	(2) Includes checklists or detailed description of import monitoring procedures	(Ref 3) This manual focuses on the taking of imported samples. "II. OBJETIVO El propósito del presente manual es describir en base a las regulaciones vigentes, el procedimiento general para la toma de muestra para el control oficial de alimentos importados a granel, con la finalidad de estandarizar dicho procedimiento." (Ref 2) More broadly describes import monitoring procedures for flour and premix: "Los inspectores de esta institución realizan la toma de muestra según el manual de procedimiento MPDNAC-004-09 muestreo para alimentos a granel, versión 13-05-2009 y el manual de procedimiento para alimentos preenvasados importados. Estos productos son evaluados a su importación al país en las áreas y recintos cuarentenarios. Las premezclas introducidas al país como materia prima, se le realizará auditoría de calidad mediante la evaluación de la conformidad, en la cual se determinará el tipo de hierro utilizado y las presencias de las vitaminas y minerales declaradas en la fórmula."
33. If there are two or more government agencies involved in import monitoring, clarifies the roles and responsibilities between different government agencies in import monitoring	(N/A) Only one government agency involved	"3. Harina de trigo importada, premezclas/harinas. Monitoreo en aduanas. La Autoridad Panameña de Seguridad Alimentaria (AUPSA) ha establecido los procedimientos para el muestreo de los alimentos importados, basados en un diseño de muestreo estadístico que toma en consideración la categorización de los alimentos clasificados según su fracción arancelaria, para la cual se valoran las variables de riesgos a considerar para la protección de la salud pública, así como la protección del patrimonio vegetal y animal en la República de Panamá."
34. If samples are to be taken, describes the sampling process: for example, number of samples, amount, frequency, individual vs. composite, where samples are taken in the process, and percent considered passing	(2) States that samples should be taken as part of import monitoring	Artículo 3
**ENFORCEMENT/PENALTIES**		
35. Indicates roles and responsibilities in enforcing the legislation	(2) States the role and responsibilities of government in enforcement	(Ref 1) Artículo 3. (Ref 2). "C. Vigilancia de la fortificación de harina de trigo en Panamá La vigilancia y fortalecimiento de estas intervenciones ha sido tarea de la Comisión Nacional de Micronutrientes, coordinada por la Dirección General de Salud..."
36. States incentives to start fortification	(0) Does not state	
37. States incentives to continue fortification, including ensuring compliance	(0) Does not state	
38. States penalties to compel compliance	(2) States any penalties	(Ref 1) Artículo 4. (Ref 2) "Definición de acciones a tomar en caso de incumplimiento Con base en el marco legal las autoridades sanitarias ha definido según el marco legal las acciones a aplicar cuando se encuentran incumplimientos durante una visita de inspección a las fábricas que fortifican alimentos. Se ha determinado que las acciones que aplican los países incluyen advertencias o acciones legales, como un incumplimiento reiterado podría ser objeto de una boleta de citación ante la región de salud correspondiente."
39. Penalties are objectively defined (e.g. first penalty=$100, second penalty=$300)	(0) Penalties are not objectively laid out	Artículo 4
40. States that enforcement is required to include feedback and support to improve performance and correct noncompliance	(2) Requires any feedback/support to improve performance	" Cuando se encuentra un incumplimiento en la inspección o auditoria realizada reconocida como una falta leve o no conformidad durante una visita, se deberá proporcionar todos los aspectos que requieren ser mejorados técnicamente en las áreas que necesitan mejorar y, darle seguimiento a la implementación de las mismas con visitas más frecuentes. (Cuadro B-2 Auditorías)"
**LABORATORY**		

41. References required analytical assays for nutrients (e.g. Liquid chromatography-mass spectrometry for folic acid, atomic absorption for iron and zinc.)	(0) Does not state requirements	Only uses iron spot test in semi-quantitative way. " Registre los resultados en el Cuadro C-1, expresados en los intervalos: 0-30 mg/kg, 30-40 mg/kg, 40-50 mg/kg, 50-60 mg/kg y > 60 mg/kg."
42. States recognition that laboratory results are subject to several sources of variation and do not provide conclusive evidence of compliance or noncompliance	(0) Does not state recognition	
43. Focuses on the quantitative analysis of "marker" micronutrients such as iron	(2) Focuses on quantitative analysis of marker micronutrient such as iron	" Prueba de mancha de hierro En el laboratorio, mezcle bien la muestra compuesta por turno y toma aproximadamente 250 g para realizar la "prueba semicuantitativa de mancha de hierro", la cual se basa en la densidad de los puntos en comparación con controles de concentración conocida de hierro adicionado (Ver Anexo)."
**REPORTING**		
44. States how government monitoring results are shared with stakeholders	(2) States how results are shared with stakeholders	"III. Monitoreo externo Los resultados de las actividades de auditoría e inspección deberían ser consolidadas dos veces al año y determinar el grado de cumplimiento de las metas de fortificación, los obstáculos a superar y las acciones a tomar. Se recomienda preparar y publicar un informe anual donde los resultados del monitoreo externo se presenten gráficamente para divulgar la situación del programa de fortificación en el país, junto con resultados de otras actividades de control de alimentos o de vigilancia tales como monitoreo comercial o vigilancia en hogares."

Paraguay Wheat

Element	Scoring (use drop-down menus)	Comments (open text)
**GENERAL**		
1. States that legislation applies to at least one food vehicle fit for human consumption (types/grades to be fortified)	(2) States at least one type fit for human consumption	(Ref 1) ARTICULO 2. (Ref 2) ARTICULO 1
2. States the public health objective; purpose and scope of legislation	(2) States the public health objective or general purpose of legislation	(Pages 1-2) "CONSIDERANDO" & ARTICULO 1
3. References latest available science or accepted international norms and recommendations, particularly for items that may not be covered in the country's documents	(2) States the documents referenced	ARTICULO 4. MERCOSUR norms are to be used if a technical specification is not explicitly noted in the Paraguayan norm.
4. Provides definitions that includes terms that are specific to fortification (e.g. fortified food, premix, fortificant, food vehicle)	(2) States at least one term related to fortification	"1. DEFINICIONES"
5. Provides repeals (if there is at least one prior document about fortification)	(2) States repeals	ARTICULO 6
6. Provides effective date or gives grace period for when fortification is to begin (e.g. effective 6 months from signing)	(2) States effective date or grace period for when fortification is to begin (e.g. effective 6 months from signing)	ARTICULO 8
**MICRONUTRIENTS/PREMIX**		
7. States nutrients required	(2) States nutrients	1.3 HARINA DE TRIGO ENRIQUECIDA CON HIERRO Y VITAMINAS
8. States fortificants (chemical compounds) to be used (including fortificants that are allowable as options)	(2) States fortificants for at least one nutrient	CALIDAD DE LOS MICRONUTRIENTES ADICIONADOS A LA HARINA
9. States fortification levels	(1) States one number only	1.3 HARINA DE TRIGO ENRIQUECIDA CON HIERRO Y VITAMINAS
10. States consideration of bioavailability/biological activity of fortificants	(0) Does not state any consideration	
11. States consideration of nutrient stability	(0) Does not state any consideration	CALIDAD DE LOS MICRONUTRIENTES ADICIONADOS A LA HARINA
**COSTING**		
12. States that the cost of fortification is regulated through cost sharing schemes (between government, industry, consumers) or tax measures [to assist industry]	(0) Does not state any consideration	
13. States consideration of the financial responsibility of monitoring and enforcing fortification (schedule of fees, budget) [on the government side]	(0) Does not state any consideration	
**LABELING**		
14. Includes some sort of statement/label/logo that makes it clear that the product is fortified	(0) Does not include statement, label, or logo	(Ref 1) Has section "3. ETIQUETADO" stating that MERCOSUR resolutions for labeling must be followed.
15. Provides guidance on health claims that can be made for this product (specific to micronutrients added through fortification)	(0) Does not provide	
**INTERNAL MONITORING (conducted by industry)**		
16. If samples are to be taken, describes the sampling process: for example, number of samples, amount, frequency, individual vs. composite, where samples are taken in the process, and percent considered passing	(2) States that samples should be taken as part of internal monitoring	(Page 19) "8.3.3.2.Toma de muestras para el control de calidad"
17. States that industry is required to follow quality assurance/quality control in regards to fortification	(2) States requirement of QA/QC for fortification	"5. Responsabilidad<br>Los establecimientos elaboradores de harina enriquecida son responsables de cumplir<br>con los reglamentos para el enriquecimiento y demás parámetros de calidad e<br>inocuidad de la harina, a través de la implementación de la presente Guía."
18. States applicability of using qualitative testing to determine the presence or absence of a vitamin or mineral (spot tests, iChecks)	(2) States applicability of spot test to determine presence/absence of vitamin or mineral specific to internal monitoring	(Page 16) "Para cumplir con el objetivo de control del enriquecimiento se debe tomar muestras de la línea de producción de harina, siguiendo el procedimiento descrito en el punto 8.3.3.2 "Toma de muestra para el control de calidad", para verificar que la premezcla está siendo dosificada correctamente, usando la prueba de la mancha de hierro (Ver ANEXO 6. Método de la mancha de hierro)."
**EXTERNAL MONITORING (conducted by government)**		
19.States requirement for external monitoring at the production site to assure compliance with standards and regulations	(2) States requirement for external monitoring or the need for audits/inspections	(Page 7) "Uno de los métodos utilizados para el control de dicho Programa/Estrategia, es la vigilancia del enriquecimiento de la harina de trigo. Esta vigilancia abarca los siguientes aspectos:<br>• Monitoreo interno en planta (Aseguramiento y Control de Calidad), a cargo del establecimiento elaborador.<br>• Monitoreo externo, a cargo del INAN (Auditoria Técnica e Inspección).<br>•<br>• Monitoreo comercial en sitios de venta, a cargo del INAN."
20. Describes protocols and systems for regulatory monitoring	(0) Does not describe	

21. If there are two or more government agencies involved in external monitoring, clarifies the roles and responsibilities between different government agencies in external monitoring	(N/A) Only one government agency involved	(Page 7) "Uno de los métodos utilizados para el control de dicho Programa/Estrategia, es la vigilancia del enriquecimiento de la harina de trigo. Esta vigilancia abarca los siguientes aspectos:<br>• Monitoreo interno en planta (Aseguramiento y Control de Calidad), a cargo del establecimiento elaborador.<br>• Monitoreo externo, a cargo del INAN (Auditoria Técnica e Inspección).<br>•<br>• Monitoreo comercial en sitios de venta, a cargo del INAN."
22. Allows for monitoring to be conducted often enough that problems can be identified and addressed on a timely basis; specifies a timeline for inspections (i.e. once every 6 months, increasing to once every 2 months if a discrepancy is found)	(0) Does not state	
23. If samples are to be taken, describes the sampling process: for example, number of samples, amount, frequency, individual vs. composite, where samples are taken in the process, and percent considered passing	(N/A) Does not describe the sampling process	
24. States applicability of using qualitative testing to determine the presence or absence of a vitamin or mineral (spot tests, iChecks)	(0) Does not state	
25. States registration is required in order to use a logo/be licensed to produce fortified foods	(2) Describes some type of registration or licensing	6. REGISTRO DE ELABORADORES E IMPORTADORES EN EL INAN"
**COMMERCIAL MONITORING (conducted by government)**		
26. Provides justification for commercial monitoring at retail stores	(2) Provides justification for commercial monitoring	(Page 7) "Uno de los métodos utilizados para el control de dicho Programa/Estrategia, es la vigilancia del enriquecimiento de la harina de trigo. Esta vigilancia abarca los siguientes aspectos:<br>• Monitoreo interno en planta (Aseguramiento y Control de Calidad), a cargo del establecimiento elaborador.<br>• Monitoreo externo, a cargo del INAN (Auditoria Técnica e Inspección).<br>•<br>• Monitoreo comercial en sitios de venta, a cargo del INAN."
27. Describes protocols and systems for commercial monitoring	(0) Does not describe	
28. If there are two or more government agencies involved in commercial monitoring, clarifies the roles and responsibilities between different government agencies in commercial monitoring	(N/A) Only one government agency involved	(Page 7) "Uno de los métodos utilizados para el control de dicho Programa/Estrategia, es la vigilancia del enriquecimiento de la harina de trigo. Esta vigilancia abarca los siguientes aspectos:<br>• Monitoreo interno en planta (Aseguramiento y Control de Calidad), a cargo del establecimiento elaborador.<br>• Monitoreo externo, a cargo del INAN (Auditoria Técnica e Inspección).<br>•<br>• Monitoreo comercial en sitios de venta, a cargo del INAN."
29. Allows for monitoring to be conducted often enough that problems at the production site or import companies can be identified and addressed on a timely basis; specifies a timeline for inspections (i.e. once every 6 months) or works with production companies to correct noncompliance	(0) Does not state	
30. If samples are to be taken, describes the sampling process: for example, number of samples, amount, frequency, individual vs. composite, where samples are taken in the process, and percent considered passing	(N/A) Does not describe the sampling process	
**IMPORT MONITORING (conducted by government)**		
31. Provides justification for import monitoring at points of entry	(2) Provides justification for import monitoring	7. AUTORIZACION DE DESPACHOS DE LA HARINA ENRIQUECIDA IMPORTADA
32. Describes protocols and systems for import monitoring	(1) Does not explicitly state, but references protocols and systems for import monitoring	7. AUTORIZACION DE DESPACHOS DE LA HARINA ENRIQUECIDA IMPORTADA
33. If there are two or more government agencies involved in import monitoring, clarifies the roles and responsibilities between different government agencies in import monitoring	(N/A) Only one government agency involved	7. AUTORIZACION DE DESPACHOS DE LA HARINA ENRIQUECIDA IMPORTADA
34. If samples are to be taken, describes the sampling process: for example, number of samples, amount, frequency, individual vs. composite, where samples are taken in the process, and percent considered passing	(N/A) Does not describe the sampling process	
**ENFORCEMENT/PENALTIES**		
35. Indicates roles and responsibilities in enforcing the legislation	(2) States the role and responsibilities of government in enforcement	ARTICULO 5. INAN is responsible for ensuringfortification decree is followed. ARTICULO 6 forms an interinstitutional commision in charge of making sure article 5 is followed.

36. States incentives to start fortification	(0) Does not state	
37. States incentives to continue fortification, including ensuring compliance	(0) Does not state	
38. States penalties to compel compliance	(2) States any penalties	(Ref 1) ARTICULO 5. (Ref 2) ARTICULO 7 [though it's hard to read]
39. Penalties are objectively defined (e.g. first penalty=$100, second penalty=$300)	(0) Penalties are not objectively laid out	
40. States that enforcement is required to include feedback and support to improve performance and correct noncompliance	(0) Does not require	
**LABORATORY**		
41. References required analytical assays for nutrients (e.g. Liquid chromatography-mass spectrometry for folic acid, atomic absorption for iron and zinc.)	(2) References required assays	"ANEXO 11 Métodos propuestos que pueden utilizar las empresas de acuerdo a su infraestructura"
42. States recognition that laboratory results are subject to several sources of variation and do not provide conclusive evidence of compliance or noncompliance	(0) Does not state recognition	
43. Focuses on the quantitative analysis of "marker" micronutrients such as iron	(0) Does not state	Document with results of external and commercial monitoring samples indicates that all nutrients in standard are measured; not just one.
**REPORTING**		
44. States how government monitoring results are shared with stakeholders	(0) Does not state how results are shared	

Peru Wheat

Element	Scoring (use drop-down menus)	Comments (open text)
**GENERAL**		
1. States that legislation applies to at least one food vehicle fit for human consumption (types/grades to be fortified)	(2) States at least one type fit for human consumption	ARTICULO 1
2. States the public health objective; purpose and scope of legislation	(0) Does not state	
3. References latest available science or accepted international norms and recommendations, particularly for items that may not be covered in the country's documents	(0) Does not state	
4. Provides definitions that includes terms that are specific to fortification (e.g. fortified food, premix, fortificant, food vehicle)	(2) States at least one term related to fortification	ARTICULO 2
5. Provides repeals (if there is at least one prior document about fortification)	(0) Does not state	"Cuarta" on page 3.
6. Provides effective date or gives grace period for when fortification is to begin (e.g. effective 6 months from signing)	(0) Does not state	
**MICRONUTRIENTS/PREMIX**		
7. States nutrients required	(2) States nutrients	ARTICULO 4
8. States fortificants (chemical compounds) to be used (including fortificants that are allowable as options)	(2) States fortificants for at least one nutrient	ARTICULO 4
9. States fortification levels	(1) States one number only	ARTICULO 4
10. States consideration of bioavailability/biological activity of fortificants	(0) Does not state any consideration	
11. States consideration of nutrient stability	(2) States consideration of nutrient stability	ARTICULO 4 (noted in text in 2nd paragraph below the table listing the fortification compounds)
**COSTING**		
12. States that the cost of fortification is regulated through cost sharing schemes (between government, industry, consumers) or tax measures [to assist industry]	(0) Does not state any consideration	
13. States consideration of the financial responsibility of monitoring and enforcing fortification (schedule of fees, budget) [on the government side]	(0) Does not state any consideration	
**LABELING**		
14. Includes some sort of statement/label/logo that makes it clear that the product is fortified	(2) Includes a statement, label, or logo	ARTICULO 5
15. Provides guidance on health claims that can be made for this product (specific to micronutrients added through fortification)	(0) Does not provide	
**INTERNAL MONITORING (conducted by industry)**		
16. If samples are to be taken, describes the sampling process: for example, number of samples, amount, frequency, individual vs. composite, where samples are taken in the process, and percent considered passing	(N/A) Does not describe the sampling process	
17. States that industry is required to follow quality assurance/quality control in regards to fortification	(0) Does not state requirement	
18. States applicability of using qualitative testing to determine the presence or absence of a vitamin or mineral (spot tests, iChecks)	(0) Does not state	
**EXTERNAL MONITORING (conducted by government)**		
19.States requirement for external monitoring at the production site to assure compliance with standards and regulations	(2) States requirement for external monitoring or the need for audits/inspections	ARTICULO 6
20. Describes protocols and systems for regulatory monitoring	(2) Includes checklists or provides detailed description of regulatory monitoring procedures	"6. INSPECCION Y RECEPCION"
21. If there are two or more government agencies involved in external monitoring, clarifies the roles and responsibilities between different government agencies in external monitoring	(N/A) Only one government agency involved	ARTICULO 6. Del aseguramiento de la calidad<br>"El CENAN es el órgano responsable de realizar inspecciones, muestreos y análisis periódicos a la harina de trigo de procedencia nacional, importada y/o donada, a fin de asegurar el cumplimiento del presente Reglamento.<br>La inspección se realizará en molinos, aduanas, distribuidoras, almacenes de importadores, otros lugares en que se encuentre a nivel nacional y dentro de toda la cadena de producción y de consumo.<br>La toma de muestras de harina de trigo se realizará en cantidad suficiente para ser sometida a análisis físicos, químicos y microbiológicos, de acuerdo con los métodos recomendados por las Normas Técnicas Peruanas.<br>Las entidades del sector público y sector privado brindarán su más amplia colaboración a las dependencias del Ministerio de Salud para el cumplimiento de sus funciones en el marco del presente Reglamento."

22. Allows for monitoring to be conducted often enough that problems can be identified and addressed on a timely basis; specifies a timeline for inspections (i.e. once every 6 months, increasing to once every 2 months if a discrepancy is found)	(0) Does not state	
23. If samples are to be taken, describes the sampling process: for example, number of samples, amount, frequency, individual vs. composite, where samples are taken in the process, and percent considered passing	(2) States that samples should be taken as part of external monitoring	(Ref 1) ARTICULO 6. (Ref 3) "6. INSPECCION Y RECEPCION".
24. States applicability of using qualitative testing to determine the presence or absence of a vitamin or mineral (spot tests, iChecks)	(0) Does not state	
25. States registration is required in order to use a logo/be licensed to produce fortified foods	(0) Does not state that registration or licensing is required	
**COMMERCIAL MONITORING (conducted by government)**		
26. Provides justification for commercial monitoring at retail stores	(2) Provides justification for commercial monitoring	ARTICULO 6
27. Describes protocols and systems for commercial monitoring	(0) Does not describe	
28. If there are two or more government agencies involved in commercial monitoring, clarifies the roles and responsibilities between different government agencies in commercial monitoring	(N/A) Only one government agency involved	ARTICULO 6. Del aseguramiento de la calidad "El CENAN es el órgano responsable de realizar inspecciones, muestreos y análisis periódicos a la harina de trigo de procedencia nacional, importada y/o donada, a fin de asegurar el cumplimiento del presente Reglamento. La inspección se realizará en molinos, aduanas, distribuidoras, almacenes de importadores, otros lugares en que se encuentre a nivel nacional y dentro de toda la cadena de producción y de consumo. La toma de muestras de harina de trigo se realizará en cantidad suficiente para ser sometida a análisis físicos, químicos y microbiológicos, de acuerdo con los métodos recomendados por las Normas Técnicas Peruanas. Las entidades del sector público y sector privado brindarán su más amplia colaboración a las dependencias del Ministerio de Salud para el cumplimiento de sus funciones en el marco del presente Reglamento."
29. Allows for monitoring to be conducted often enough that problems at the production site or import companies can be identified and addressed on a timely basis; specifies a timeline for inspections (i.e. once every 6 months) or works with production companies to correct noncompliance	(N/A) No commercial monitoring occurs	
30. If samples are to be taken, describes the sampling process: for example, number of samples, amount, frequency, individual vs. composite, where samples are taken in the process, and percent considered passing	(1) States that samples should be taken (generally)	ARTICULO 6
**IMPORT MONITORING (conducted by government)**		
31. Provides justification for import monitoring at points of entry	(2) Provides justification for import monitoring	ARTICULO 3, ARTICULO 6
32. Describes protocols and systems for import monitoring	(2) Includes checklists or detailed description of import monitoring procedures	ARTICULO 3
33. If there are two or more government agencies involved in import monitoring, clarifies the roles and responsibilities between different government agencies in import monitoring	(N/A) Only one government agency involved	ARTICULO 6. Del aseguramiento de la calidad "El CENAN es el órgano responsable de realizar inspecciones, muestreos y análisis periódicos a la harina de trigo de procedencia nacional, importada y/o donada, a fin de asegurar el cumplimiento del presente Reglamento. La inspección se realizará en molinos, aduanas, distribuidoras, almacenes de importadores, otros lugares en que se encuentre a nivel nacional y dentro de toda la cadena de producción y de consumo. La toma de muestras de harina de trigo se realizará en cantidad suficiente para ser sometida a análisis físicos, químicos y microbiológicos, de acuerdo con los métodos recomendados por las Normas Técnicas Peruanas. Las entidades del sector público y sector privado brindarán su más amplia colaboración a las dependencias del Ministerio de Salud para el cumplimiento de sus funciones en el marco del presente Reglamento."
34. If samples are to be taken, describes the sampling process: for example, number of samples, amount, frequency, individual vs. composite, where samples are taken in the process, and percent considered passing	(1) States that samples should be taken (generally)	ARTICULO 6
**ENFORCEMENT/PENALTIES**		

35. Indicates roles and responsibilities in enforcing the legislation	(2) States the role and responsibilities of government in enforcement	ARTICULO 6
36. States incentives to start fortification	(0) Does not state	
37. States incentives to continue fortification, including ensuring compliance	(0) Does not state	
38. States penalties to compel compliance	(2) States any penalties	ARTICULO 7
39. Penalties are objectively defined (e.g. first penalty=$100, second penalty=$300)	(0) Penalties are not objectively laid out	No, not in ARTICULO 7 or in ARTICULO 8
40. States that enforcement is required to include feedback and support to improve performance and correct noncompliance	(0) Does not require	
**LABORATORY**		
41. References required analytical assays for nutrients (e.g. Liquid chromatography-mass spectrometry for folic acid, atomic absorption for iron and zinc.)	(0) Does not state requirements	
42. States recognition that laboratory results are subject to several sources of variation and do not provide conclusive evidence of compliance or noncompliance	(0) Does not state recognition	
43. Focuses on the quantitative analysis of "marker" micronutrients such as iron	(0) Does not state	
**REPORTING**		
44. States how government monitoring results are shared with stakeholders	(0) Does not state how results are shared	

Philippines Wheat

Element	Scoring (use drop-down menus)	Comments (open text)
**GENERAL**		
1. States that legislation applies to at least one food vehicle fit for human consumption (types/grades to be fortified)	(2) States at least one type fit for human consumption	Sec. 6. Mandatory Food Fortification. - (a) The fortification of staple foods based on standards set by the DOH through the BFAD is hereby made mandatory for the following: (2) Wheat flour - with vitamin A and Iron
2. States the public health objective; purpose and scope of legislation	(2) States the public health objective or general purpose of legislation	SEC. 2. Declaration of Policies. - Section 15 of Article II of the Constitution provides that the State shall protect and promote the right to health of the people and instill health consciousness among them. The state recognizes that nutritional deficiency problems in the Philippines, based on nutrition surveys, include deficiencies in energy, iron, vitamin A, iodine, thiamin and riboflavin. To a minor extent, the Filipino diet is also deficient in ascorbic acid, calcium and folate. The State recognizes that food fortification is vital where there is a demonstrated need to increase the intake of an essential nutrient by one or more population groups, as manifested in dietary, biochemical or clinical evidences of deficiency. Food fortification is considered important in the promotion of optimal health and to compensate for the loss of nutrients due to processing and/or storage of food. Food fortification, therefore, shall be carried out to compensate for the inadequacies in Filipino diet, based on present-day needs as measured using the most recent Recommended Dietary Allowances (RDA).
3. References latest available science or accepted international norms and recommendations, particularly for items that may not be covered in the country's documents	(0) Does not state	The DOH guidelines on micronutrient fortification of processed foods or food products included in Administrative Order No. 4-A series of 1995 and such other necessary guidelines that may be issued by the DOH, shall serve as a basis for the addition of micronutrient(s) to processed foods or food products
4. Provides definitions that includes terms that are specific to fortification (e.g. fortified food, premix, fortificant, food vehicle)	(2) States at least one term related to fortification	Rule III Definition of terms. Section 1. For the purpose of this act, the following terms shall mean: fortification, fortificant, micronutrient, etc.
5. Provides repeals (if there is at least one prior document about fortification)	(2) States repeals	Sec. 14. Repealing Clause. -All laws, decrees, rule and regulations, executive orders inconsistent with the provisions of this Act are hereby repealed or modified accordingly.
6. Provides effective date or gives grace period for when fortification is to begin (e.g. effective 6 months from signing)	(2) States effective date or grace period for when fortification is to begin (e.g. effective 6 months from signing)	SECTION 2. The Implementation of the Mandatory Food Fortification for wheat flour, refined sugar, cooking oil and rice, including those milled and/or distributed by the Natioanl food Authority, shall commence after four years from the effectivity of Rep. Act NO. 8976, which was November 7, 2000, hence the Implementing Rules and Regulation for Mandatory Food Fortification shall take effect on November 7, 2004.
**MICRONUTRIENTS/PREMIX**		
7. States nutrients required	(2) States nutrients	Rule VI, Section 1.2 Table
8. States fortificants (chemical compounds) to be used (including fortificants that are allowable as options)	(2) States fortificants for at least one nutrient	Rule VI, Section 1.2 Table
9. States fortification levels	(2) States a range or number with +/-	Ranges given for both vitamin A and iron
10. States consideration of bioavailability/biological activity of fortificants	(0) Does not state any consideration	
11. States consideration of nutrient stability	(2) States consideration of nutrient stability	SECTION 3. The label of fortified staple food products offered for sale in the market shall include a statement of the fortificant added and the amount present within the shelf life of the product.
**COSTING**		
12. States that the cost of fortification is regulated through cost sharing schemes (between government, industry, consumers) or tax measures [to assist industry]	(2) States consideration of either cost regulation method	Rule IX-Support to Affected Manufacturers
13. States consideration of the financial responsibility of monitoring and enforcing fortification (schedule of fees, budget) [on the government side]	(2) Shows consideration that monitoring costs money	SECTION 2. The agencies mentioned in Rule VIII and Rule IX mandated to implement and support the food fortification program shall allocate part of their budget for the implementation and support of this program.
**LABELING**		
14. Includes some sort of statement/label/logo that makes it clear that the product is fortified	(2) Includes a statement, label, or logo	Sangkap Pinoy Seal Program – a strategy to encourage manufacturers to fortify processed foods or food products with essential nutrients at levels approved by the DOH. The fundamental concept of the program is to authorize manufacturers to use the DOH seal of acceptance for processed foods or food products, after these products passed a set of defined criteria. The seal is a guide used by consumers in selecting nutritious foods.

15. Provides guidance on health claims that can be made for this product (specific to micronutrients added through fortification)	(0) Does not provide	
**INTERNAL MONITORING (conducted by industry)**		
16. If samples are to be taken, describes the sampling process: for example, number of samples, amount, frequency, individual vs. composite, where samples are taken in the process, and percent considered passing	(N/A) Does not describe the sampling process	
17. States that industry is required to follow quality assurance/quality control in regards to fortification	(2) States requirement of QA/QC for fortification	SECTION 1. In accordance with the mandate of Sec. 7 of Rep. Act No. 8976, the agencies responsible for the implementation of this law shall establish a quality assurance system with respect to food fortification. However, manufacturers and importers of processed food or food products or Repackers shall also establish their own quality assurance system, which shall conform to the quality assurance system of the implementing agency. Annex I is the General Quality Assurance system for Food Fortification to be used as guide.
18. States applicability of using qualitative testing to determine the presence or absence of a vitamin or mineral (spot tests, iChecks)	(0) Does not state	
**EXTERNAL MONITORING (conducted by government)**		
19.States requirement for external monitoring at the production site to assure compliance with standards and regulations	(0) Does not state requirement	
20. Describes protocols and systems for regulatory monitoring	(1) Does not explicitly describe, but references protocols and systems for regulatory monitoring	Rule VIII "SECTION 5. The BFAD shall formulate the standard operating procedures (SOP) for monitoring the implementation of this Act."
21. If there are two or more government agencies involved in external monitoring, clarifies the roles and responsibilities between different government agencies in external monitoring	(2) Clarifies roles and responsibilities for more than one agency	Rule VIII "SECTION 4. The DOH through BFAD, as the lead implementing agency shall be assisted in the monitoring and review of the program by the following:<br>a. Sugar Regulatory Administration (SRA) for sugar; b. National Food Authority (NFA) for rice; c. Philippine Coconut Authority (PCA); d. Bureau of Customs for imported products e. The other agencies enumerated in Rule No. IX.<br>The BFAD shall continue to monitor and review the fortification of wheat flour.<br>SECTION 5. The BFAD shall formulate the standard operating procedures (SOP) for monitoring the implementation of this Act."
22. Allows for monitoring to be conducted often enough that problems can be identified and addressed on a timely basis; specifies a timeline for inspections (i.e. once every 6 months, increasing to once every 2 months if a discrepancy is found)	(0) Does not state	
23. If samples are to be taken, describes the sampling process: for example, number of samples, amount, frequency, individual vs. composite, where samples are taken in the process, and percent considered passing	(N/A) Does not describe the sampling process	
24. States applicability of using qualitative testing to determine the presence or absence of a vitamin or mineral (spot tests, iChecks)	(0) Does not state	
25. States registration is required in order to use a logo/be licensed to produce fortified foods	(2) Describes some type of registration or licensing	SECTION 4. The above named business establishments enumerated in the preceding section except food service establishments shall be duly licensed with the concerned government agencies such as National Food Authority for rice … They shall likewise inform BFAD prior to the process of fortification and they shall register their products with BFAD prior to distribution.
**COMMERCIAL MONITORING (conducted by government)**		
26. Provides justification for commercial monitoring at retail stores	(2) Provides justification for commercial monitoring	SECTION 6. The Local Government Units (LGUs) shall assist in the monitoring of foods mandated to be fortified, in public markets retail stores, and food service establishments. It shall likewise check if the labels of fortified products contain nutrition facts stating the nutrient added and its quantity. The LGUs shall designate only one set from the following officials enumerated in Rep. Act No. 8976, Sec. 8 to conduct the monitoring or checking functions: Head officers or Agricultural officers or Nutritionist-dieticians or Sanitary Inspectors The LGUs shall inform BFAD which of the officers enumerated above has been assigned to monitor the fortified products. The LGUs shall submit reports on monitoring to the Bureau of Food and Drugs (BFAD) in the manner and form prescribed by the latter.
27. Describes protocols and systems for commercial monitoring	(1) Does not explicitly describe, but references protocols and systems for regulatory monitoring	

28. If there are two or more government agencies involved in commercial monitoring, clarifies the roles and responsibilities between different government agencies in commercial monitoring	(2) Clarifies roles and responsibilities for more than one agency	SECTION 6. The Local Government Units (LGUs) shall assist in the monitoring<br>of foods mandated to be fortified, in public markets, retail stores, and food service<br>establishments. It shall likewise check if the labels of fortified products contain nutrition<br>facts stating the nutrient added and its quantity. The LGUs shall designate only one set<br>from the following officials enumerated in Rep. Act No. 8976, Sec. 8 to conduct the<br>monitoring or checking functions:<br>Health officers or<br>Agricultural officers or<br>Nutritionist-dieticians or<br>Sanitary Inspectors<br>The LGUs shall inform BFAD which of the officers enumerated above as been<br>assigned to monitor the fortified products.<br>The LGUs shall submit reports on monitoring to the Bureau of Food and Drugs<br>(BFAD) in the manner and form prescribed by the latter.
29. Allows for monitoring to be conducted often enough that problems at the production site or import companies can be identified and addressed on a timely basis; specifies a timeline for inspections (i.e. once every 6 months) or works with production companies to correct noncompliance	(0) Does not state	
30. If samples are to be taken, describes the sampling process: for example, number of samples, amount, frequency, individual vs. composite, where samples are taken in the process, and percent considered passing	(N/A) Does not describe the sampling process	
**IMPORT MONITORING (conducted by government)**		
31. Provides justification for import monitoring at points of entry	(2) Provides justification for import monitoring	Imported rice, wheat flour, refined sugar, cooking oil and other processed foods or food products that may be identified later by the NNC, shall comply with the requirements of this Act on entry in the country, at the end of manufacturing process and/or at all points of sale or distribution
32. Describes protocols and systems for import monitoring	(2) Includes checklists or detailed description of import monitoring procedures	RULE VII. "SECTION 1. In accordance with the mandate of Sec. 7 of Rep. Act No. 8976, the agencies responsible for the implementation of this law shall establish a quality assurance system with respect to food fortification. However, manufacturers and importers of processed food or food products or repackers shall also establish their own quality assurance system, which shall conform to the quality assurance system of the implementing agency. Annex 1 is the General Quality Assurance system for Food Fortification to be used as a guide."
33. If there are two or more government agencies involved in import monitoring, clarifies the roles and responsibilities between different government agencies in import monitoring	(2) Clarifies roles and responsibilities for more than one agency	SECITON 8. The Bureau of Customs shall inform the DOH through BFAD of all imported rice, sugar, wheat flour and cooking oil, prior to their release, to enable BFAD to ascertain if such imported products are already fortified. BFAD shall undertake a final inspection that the products are really fortified and registered with BFAD as fortified products prior to sale and distribution
34. If samples are to be taken, describes the sampling process: for example, number of samples, amount, frequency, individual vs. composite, where samples are taken in the process, and percent considered passing	(N/A) Does not describe the sampling process	
**ENFORCEMENT/PENALTIES**		
35. Indicates roles and responsibilities in enforcing the legislation	(2) States the role and responsibilities of government in enforcement	The DOH through the BFAD, after notice and hearing, shall impose any or all of the following administrative sanctions in cases of noncompliance with the food fortification guidelines it has set (denial of registration, recall of food, fine)
36. States incentives to start fortification	(2) States any incentives to encourage fortification initiation (e.g. tax incentives for new equipment or premix)	Section 1. The affected manufacturers shall be supported by the following government agencies in the achievement of the purposes of this act
37. States incentives to continue fortification, including ensuring compliance	(2) States any incentives to encourage the continuation of fortification (e.g. transport priority, favorable tax or tariff treatment, or patent rights)	Section 1. The affected manufacturers shall be supported by the following government agencies in the achievement of the purposes of this act
38. States penalties to compel compliance	(2) States any penalties	Rule XI , Section 1. and Sec. 7. Any person found guilty of violating any provision of this Act shall be punished for each and every offense by an imprisonment of not less than thirty days and not more than six months or a fine of not less than two hundred pesos and not more than two thousand pesos or by both such fine and imprisonment in the discretion of the court.

39. Penalties are objectively defined (e.g. first penalty=$100, second penalty=$300)	(2) Penalties are objectively laid out in the document (e.g. first penalty=$100, second penalty=$300)	Rule XI , Section 1. and Sec. 7. Any person found guilty of violating any provision of this Act shall be punished for each and every offense by an imprisonment of not less than thirty days and not more than six months or a fine of not less than two hundred pesos and not more than two thousand pesos or by both such fine and imprisonment in the discretion of the court.
40. States that enforcement is required to include feedback and support to improve performance and correct noncompliance	(0) Does not require	
**LABORATORY**		
41. References required analytical assays for nutrients (e.g. Liquid chromatography-mass spectrometry for folic acid, atomic absorption for iron and zinc.)	(0) Does not state requirements	
42. States recognition that laboratory results are subject to several sources of variation and do not provide conclusive evidence of compliance or noncompliance	(0) Does not state recognition	
43. Focuses on the quantitative analysis of "marker" micronutrients such as iron	(0) Does not state	
**REPORTING**		
44. States how government monitoring results are shared with stakeholders	(0) Does not state how results are shared	The local food industries shall report on the production, marketing and distribution of fortified foods. They shall submit annual reports to the DOH, also indicating their industrial concerns and recommendations.

Republic of Moldova Wheat

Element	Scoring (use drop-down menus)	Comments (open text)
**GENERAL**		
1. States that legislation applies to at least one food vehicle fit for human consumption (types/grades to be fortified)	(2) States at least one type fit for human consumption	Annex 3 - Wheat or meslin flour - page 1 table gives types
2. States the public health objective; purpose and scope of legislation	(2) States the public health objective or general purpose of legislation	Annex 3 - Page 1 - Nominal composition of the council for coordination of the national program to reduce sicknesses determined by iron and folic acid deficiency
3. References latest available science or accepted international norms and recommendations, particularly for items that may not be covered in the country's documents	(2) States the documents referenced	Tech Regs - Page 2(4) - Provides the basis necessary for the application of regs of the Codex
4. Provides definitions that includes terms that are specific to fortification (e.g. fortified food, premix, fortificant, food vehicle)	(2) States at least one term related to fortification	Tech Regs - Page 4(k) - Enriched
5. Provides repeals (if there is at least one prior document about fortification)	(2) States repeals	Law on Food - Page 6(2) - If international agreements includes provisions other than those contained in the legislation, the provisions of international agreements shall prevail.
fortification is to begin (e.g. effective 6 months from signing)	fortification is to begin (e.g. effective 6 months from signing)	the Gazette
**MICRONUTRIENTS/PREMIX**		
7. States nutrients required	(2) States nutrients	Tech Regs - Page 4(7.2) - Iron and folic acid
8. States fortificants (chemical compounds) to be used (including fortificants that are allowable as options)	(2) States fortificants for at least one nutrient	Tech Regs - Page 4(7.2) - iron fumarate or elemental iron, and pteroylmonoglutamic acid
9. States fortification levels	(2) States a range or number with +/-	Tech Regs - Page 5(7.3 and 7.4) - 30 mg iron/kg (+/- 15%) for fumarate...
10. States consideration of bioavailability/biological activity of fortificants	(0) Does not state any consideration	
11. States consideration of nutrient stability	(2) States consideration of nutrient stability	Tech Regs - Page 4(7) - conditions of... to avoid the loss of
**COSTING**		
12. States that the cost of fortification is regulated through cost sharing schemes (between government, industry, consumers) or tax measures [to assist industry]	(2) States consideration of either cost regulation method	Decree 201 - Page 3(12) - enriched wheat flour shall be provided to all pregnant women free of charge covered by healthcare insurance.; Decree 201 - Page 5(16.2) - start fortification in three pilot mills with provision of dosing machines and premixes; Law on Food - Page 17(5) - Food products enriched have an advantage over non-enriched foods in the same category in transportation, storage, and distribution
13. States consideration of the financial responsibility of monitoring and enforcing fortification (schedule of fees, budget) [on the government side]	(2) Shows consideration that monitoring costs money	Decree 201 - Page 1(5) - Actions of the program shall be covered financially from allocations approved yearly in the national public budget and other sources.; Law on Food - Page 21 (5) - expenses for examination, storage, transporrtation, use, or destruction of food that is non-compliant is paid by the food industry
**LABELING**		
14. Includes some sort of statement/label/logo that makes it clear that the product is fortified	(2) Includes a statement, label, or logo	Tech Regs - Page 6(16.1) - enriched flour sold under the name "flour enriched with iron and folic acid""
15. Provides guidance on health claims that can be made for this product (specific to micronutrients added through fortification)	(2) Provides guidance on health claims specific to micronutrients added through fortification	Law on Food - Page 14(10) - Inscriptions of the prophylactic or therapeutic properties of food is prohibited except when they are proved or confirmed by the Ministry of Health. This applies to the advertising of food
**INTERNAL MONITORING (conducted by industry)**		
16. If samples are to be taken, describes the sampling process: for example, number of samples, amount, frequency, individual vs. composite, where samples are taken in the process, and percent considered passing	(1) States that samples should be taken (generally)	Tech Regs - Page 7(21) - sampling… produced in accordance with the rules and analysis methods established in the national standards
17. States that industry is required to follow quality assurance/quality control in regards to fortification	(2) States requirement of QA/QC for fortification	Tech Regs - Page 4(7) - methods and processes of production, packaging, storage, transportation, and marketing of flour wheat… carried out under conditions of eliminating risk of infection, lowering quality, avoid loss of iron and folic acid, and compliance with hygiene standards; Decree 201 - Page 3(11) - "Milling enterprises shall ensure adequate and homogenous fortification of wheat flour with iron and folic acid.; Law on Food - Page 18 (Article 20) - economic entities are obliged to carry out monitoring... (Article 21) - obligation to use HACCP
18. States applicability of using qualitative testing to determine the presence or absence of a vitamin or mineral (spot tests, iChecks)	(0) Does not state	
**EXTERNAL MONITORING (conducted by government)**		
19.States requirement for external monitoring at the production site to assure compliance with standards and regulations	(2) States requirement for external monitoring or the need for audits/inspections	Decree 201 - Page 6(16.11) - monitor quality of flour locally produced and imported

20. Describes protocols and systems for regulatory monitoring	(2) Includes checklists or provides detailed description of regulatory monitoring procedures	Food Safety Law - Page 8(14.2) - authorized authority carries out official controls and other activities, including… monitoring of the entire food chain; Law on PHS - Page 13(Article 18) - lists all the protocols for government inspection of facilities to verify compliance with the sanitation legislation
21. If there are two or more government agencies involved in external monitoring, clarifies the roles and responsibilities between different government agencies in external monitoring	(2) Clarifies roles and responsibilities for more than one agency	Decree 201 - Page 8(30) - coordinated by a multi-stakeholder council, implementation and M&E by MoH and MoAg/Food Industry, Cooperation by Academy of Sciences; Food Safety Law - Page 6(Article 10) - National Agency for Food Safety develops standards, promotes coordination of activities...; Food Safety Law - Page 11(18.2) - Main task of the National Agency for Food Safeety... monitoring food processing, storage, transportation, sale, imports/exports; Food Safety Law - Page 13(19.e) - MoH ensures compliance with hygiene requirements of enterprises engaged in production, processing, sale of food
22. Allows for monitoring to be conducted often enough that problems can be identified and addressed on a timely basis; specifies a timeline for inspections (i.e. once every 6 months, increasing to once every 2 months if a discrepancy is found)	(0) Does not state	Law on PHS - Page 21 (6) - timely detection of issues and their relationship with food products, traceabity of suspicious food provided by the implementation of integrated systems for supervision.
23. If samples are to be taken, describes the sampling process: for example, number of samples, amount, frequency, individual vs. composite, where samples are taken in the process, and percent considered passing	(1) States that samples should be taken (generally)	Law on PHS - Page 13 (6) - staff are allowed to take samples of materials which may present a risk to public health as part of external monitoring of production sites
24. States applicability of using qualitative testing to determine the presence or absence of a vitamin or mineral (spot tests, iChecks)	(0) Does not state	
25. States registration is required in order to use a logo/be licensed to produce fortified foods	(0) Does not state that registration or licensing is required	Law on Food - Page 11(2b) - Refers to a state register of food producers, though not specific to fortification; Law on PHS - Page 16(Article 24(3)) - additives containing only vitamins/minerals are excluded from state registration
**COMMERCIAL MONITORING (conducted by government)**		
26. Provides justification for commercial monitoring at retail stores	(2) Provides justification for commercial monitoring	Tech Regs - Page 7(22) - may be placed on the market only if they meet requirements and comply with the law.
27. Describes protocols and systems for commercial monitoring	(0) Does not describe	Tech Regs - Page 7(22) - accordance of cereals is ensured by the manufacturer/importer
28. If there are two or more government agencies involved in commercial monitoring, clarifies the roles and responsibilities between different government agencies in commercial monitoring	(2) Clarifies roles and responsibilities for more than one agency	Decree 201 - Page 8(30) - coordinated by a multi-stakeholder council, implementation and M&E by MoH and MoAg/Food Industry, Cooperation by Academy of Sciences; Food Safety Law - Page 6(Article 10) - National Agency for Food Safety develops standards, promotes coordination of activities...; Food Safety Law - Page 11(18.2) - Main task of the National Agency for Food Safeety... monitoring food processing, storage, transportation, sale, imports/exports
29. Allows for monitoring to be conducted often enough that problems at the production site or import companies can be identified and addressed on a timely basis; specifies a timeline for inspections (i.e. once every 6 months) or works with production companies to correct noncompliance	(0) Does not state	
30. If samples are to be taken, describes the sampling process: for example, number of samples, amount, frequency, individual vs. composite, where samples are taken in the process, and percent considered passing	(N/A) Does not describe the sampling process	
**IMPORT MONITORING (conducted by government)**		
31. Provides justification for import monitoring at points of entry	(2) Provides justification for import monitoring	Tech Regs - Page 4(7.1) - imported… is enriched with iron and folic acid in accordance with the terms specified
32. Describes protocols and systems for import monitoring	(1) Does not explicitly state, but references protocols and systems for import monitoring	Law on Food - Page 11(2) - authorization is carried out by the authority of the state, involving…
33. If there are two or more government agencies involved in import monitoring, clarifies the roles and responsibilities between different government agencies in import monitoring	(2) Clarifies roles and responsibilities for more than one agency	Decree 201 - Page 8(30) - coordinated by a multi-stakeholder council, implementation and M&E by MoH and MoAg/Food Industry, Cooperation by Academy of Sciences; Food Safety Law - Page 6(Article 10) - National Agency for Food Safety develops standards, promotes coordination of activities...; Food Safety Law - Page 11(18.2) - Main task of the National Agency for Food Safeety... monitoring food processing, storage, transportation, sale, imports/exports; Food Safety Law - Page 13(19.e) - MoH ensures compliance with hygiene requirements of enterprises engaged in production, processing, sale of food

34. If samples are to be taken, describes the sampling process: for example, number of samples, amount, frequency, individual vs. composite, where samples are taken in the process, and percent considered passing	(N/A) Does not describe the sampling process	
**ENFORCEMENT/PENALTIES**		
35. Indicates roles and responsibilities in enforcing the legislation	(2) States the role and responsibilities of government in enforcement	Tech Regulations - Page 1(3) - The monitoring of the enforcement of this decision lies with the Natl Agency for Food Safety and the MoH
36. States incentives to start fortification	(2) States any incentives to encourage fortification initiation (e.g. tax incentives for new equipment or premix)	Decree 201 - Page 5(16.4, 16.7, 16.8) - purchases of premix and equipment, training of millers - We are assuming all actions in section 16 are done by government.
37. States incentives to continue fortification, including ensuring compliance	(2) States any incentives to encourage the continuation of fortification (e.g. transport priority, favorable tax or tariff treatment, or patent rights)	Decree 201 - Page 5(16.4, 16.7, 16.8) - favorable import conditions, purchases of premix and equipment, training of millers - Assuming actions are done by government.
38. States penalties to compel compliance	(2) States any penalties	sanctions must be effective, proportionate, and preventative
39. Penalties are objectively defined (e.g. first penalty=$100, second penalty=$300)	(0) Penalties are not objectively laid out	
40. States that enforcement is required to include feedback and support to improve performance and correct noncompliance	(0) Does not require	
**LABORATORY**		
41. References required analytical assays for nutrients (e.g. Liquid chromatography-mass spectrometry for folic acid, atomic absorption for iron and zinc.)	(0) Does not state requirements	
42. States recognition that laboratory results are subject to several sources of variation and do not provide conclusive evidence of compliance or noncompliance	(0) Does not state recognition	
43. Focuses on the quantitative analysis of "marker" micronutrients such as iron	(0) Does not state	
**REPORTING**		
44. States how government monitoring results are shared with stakeholders	(2) States how results are shared with stakeholders	Decree 201 - Page 1(6) - submitting yearly reports to MoH to generalize data

Senegal Wheat

Element	Scoring (use drop-down menus)	Comments (open text)
**GENERAL**		
1. States that legislation applies to at least one food vehicle fit for human consumption (types/grades to be fortified)	(2) States at least one type fit for human consumption	First article. — The enrichment of edible oils with vitamin A and of wheat bread flour with iron and folic acid is rendered mandatory within the national territory. It applies to wheat flour obtained from species Triticum aestivum (wheat) or L Triticum compactum HOST. (club wheat), or a mixture of both.
2. States the public health objective; purpose and scope of legislation	(2) States the public health objective or general purpose of legislation	The lack of vitamins and minerals, or "invisible hunger" constitutes a real public health problem in the world and particularly in Sub-Saharan Africa, where a third of the population continues to suffer from it. In Senegal, the proportion of children under the age of six affected by a lack of sub-clinical vitamin A is 61% while anaemia, which is the most often the consequent of a nutritional deficiency of iron, afflicts 82.6% of children from 6 to 59 months, and 59.1% of women from 15 to 49 (2005 Demographic and Health Investigation in Senegal).
3. References latest available science or accepted international norms and recommendations, particularly for items that may not be covered in the country's documents	(2) States the documents referenced	They must be prepared according to International Code of Hygienic Practice and in accordance with the general principles of the Food Hygiene Codex Alimentarius. References Codex as normative text in many other places as well.
4. Provides definitions that includes terms that are specific to fortification (e.g. fortified food, premix, fortificant, food vehicle)	(0) Does not state	Provides definitions, but nothing related to fortification
5. Provides repeals (if there is at least one prior document about fortification)	(N/A) No prior documents about fortification	Thus, the will of the Senegalese Government is further confirmed by the adoption of Decree No. 2009-872 of 10 September 2009 mandating the implementation of standards on oils enriched with vitamin A and enriched wheat flour with iron and folic acid (vitamin B9). Henceforth, it is the obligation of all operators (manufacturers or importers) to only make available adequately enriched oils and flour.
6. Provides effective date or gives grace period for when fortification is to begin (e.g. effective 6 months from signing)	(0) Does not state	Signed 10 September 2009, but no effective date given.
**MICRONUTRIENTS/PREMIX**		
7. States nutrients required	(2) States nutrients	The content of ferrous sulfate, ferrous fumarate and iron EDTA must be between 55 and 65 mg / kg to the production, importation and distribution. Vitamin B9 content must be between 2.25 and 2,75mg / kg to the production, importation and distribution.
8. States fortificants (chemical compounds) to be used (including fortificants that are allowable as options)	(2) States fortificants for at least one nutrient	The content of ferrous sulfate, ferrous fumarate and iron EDTA must be between 55 and 65 mg / kg to the production, importation and distribution. Vitamin B9 content must be between 2.25 and 2,75mg / kg to the production, importation and distribution.
9. States fortification levels	(2) States a range or number with +/-	The content of ferrous sulfate, ferrous fumarate and iron EDTA must be between 55 and 65 mg / kg to the production, importation and distribution. Vitamin B9 content must be between 2.25 and 2,75mg / kg to the production, importation and distribution.
10. States consideration of bioavailability/biological activity of fortificants	(0) Does not state any consideration	
11. States consideration of nutrient stability	(2) States consideration of nutrient stability	6.1.1. Packaging materials, packing and accessories as well as places used for the transactions must be clean, dry and free of extraneous odors. Packages must be sealed to ensure optimum stability of iron and vitamin B9. Any exposure to light and moisture should be avoided. 6.1.2. The soft wheat flour fortified with iron and vitamin B9 must be packed in containers which preserve the hygienic, nutritional and technological qualities.
**COSTING**		
12. States that the cost of fortification is regulated through cost sharing schemes (between government, industry, consumers) or tax measures [to assist industry]	(0) Does not state any consideration	
13. States consideration of the financial responsibility of monitoring and enforcing fortification (schedule of fees, budget) [on the government side]	(0) Does not state any consideration	
**LABELING**		
14. Includes some sort of statement/label/logo that makes it clear that the product is fortified	(2) Includes a statement, label, or logo	product name with "soft wheat flour fortified with iron and vitamin B9";
15. Provides guidance on health claims that can be made for this product (specific to micronutrients added through fortification)	(0) Does not provide	

**INTERNAL MONITORING (conducted by industry)**		
16. If samples are to be taken, describes the sampling process: for example, number of samples, amount, frequency, individual vs. composite, where samples are taken in the process, and percent considered passing	(N/A) Does not describe the sampling process	
17. States that industry is required to follow quality assurance/quality control in regards to fortification	(0) Does not state requirement	
18. States applicability of using qualitative testing to determine the presence or absence of a vitamin or mineral (spot tests, iChecks)	(0) Does not state	
**EXTERNAL MONITORING (conducted by government)**		
19.States requirement for external monitoring at the production site to assure compliance with standards and regulations	(2) States requirement for external monitoring or the need for audits/inspections	This procedure is intended to describe the methodology used by the agents of the DCI during the quality control of oils and fortified flour in manufacturing.
20. Describes protocols and systems for regulatory monitoring	(2) Includes checklists or provides detailed description of regulatory monitoring procedures	Goes into a lot of detail on what must be done, but this is the outline... Like any food product, the manufacturing of oil or fortified flour requires a manufacturing authorization from the DCSC after a control that is exercised at several levels:<br>- Documentary checks;<br>- A control of labeling;<br>- A visual physical check;<br>- A physicochemical and/ or microbiological control;<br>- A check of local manufacturing;<br>- Documents issued
21. If there are two or more government agencies involved in external monitoring, clarifies the roles and responsibilities between different government agencies in external monitoring	(2) Clarifies roles and responsibilities for more than one agency	This manual was developed by the officers of the Directorate of Internal Trade in charge of the quality control of food products. It is an important work tool (logbook) for all employees in the quality control of fortified products. Thus, the central services of the DCI and the decentralized departments will appropriate this reference document to harmonize interventions on the one hand and improve the control effectiveness on the other. The quality control of oils and fortified flour is naturally in the food quality control provided by the Ministry for Trade through the Division of Consumer Affairs and Consumer Safety (DCSC) and decentralized Internal Trade services (regional and departmental services). In total, there are 14 regional services and 20 departmental services for Commerce across the country. It should be noted that this control is carried out in collaboration with other jurisdictions such as the Directorate of Plant Protection (DPV), the Directorate of Veterinary Services (DSV), the Customs Directorate (DGD), etc.
22. Allows for monitoring to be conducted often enough that problems can be identified and addressed on a timely basis; specifies a timeline for inspections (i.e. once every 6 months, increasing to once every 2 months if a discrepancy is found)	(0) Does not state	However, he is subject to regular, unannounced control officers from the DCSC or the decentralized trade services
23. If samples are to be taken, describes the sampling process: for example, number of samples, amount, frequency, individual vs. composite, where samples are taken in the process, and percent considered passing	(2) States that samples should be taken as part of external monitoring	The taking of samples of wheat flour enriched with iron and folic acid for control must be performed according to the method described in the standard (NFV 03-740). The officer must check whether the samples submitted by the applicant are in good standing. He must check:<br>- if the number of samples submitted is sufficient (4-7 samples)<br>- if the samples are not already altered upon filing,<br>- if the conditions for preservation are good.
24. States applicability of using qualitative testing to determine the presence or absence of a vitamin or mineral (spot tests, iChecks)	(0) Does not state	

25. States registration is required in order to use a logo/be licensed to produce fortified foods	(2) Describes some type of registration or licensing	□ Documentation<br>The documents referred to in the context of an application for manufacturing authorization are:<br>- manufacturing authorization request to the Head of the Consumer Division and duly signed by the applicant. It must be specified in the request the product subject of the manufacturing request, its packaging, its weight or volume and its brand. See license application model in the Appendix.<br>- the certificate of registration in the Personal Credit and Commercial Register and (RCCM)<br>- the National Identification number of companies and associations (NINEA) attesting to the applicant's registration at the tax office. If the Distributor of the enriched oils or flour is not adequately established to carry out business (not registered with the RCCM), seize the product and initiate litigation; cf. Copy of Failure to register in the commercial register (DIRC) report;
**COMMERCIAL MONITORING (conducted by government)**		
26. Provides justification for commercial monitoring at retail stores	(0) Does not provide justification for commercial monitoring	
27. Describes protocols and systems for commercial monitoring	(0) Does not describe	
28. If there are two or more government agencies involved in commercial monitoring, clarifies the roles and responsibilities between different government agencies in commercial monitoring	(0) Clarifies roles and responsibilities for no agencies	
29. Allows for monitoring to be conducted often enough that problems at the production site or import companies can be identified and addressed on a timely basis; specifies a timeline for inspections (i.e. once every 6 months) or works with production companies to correct noncompliance	(N/A) No commercial monitoring occurs	
30. If samples are to be taken, describes the sampling process: for example, number of samples, amount, frequency, individual vs. composite, where samples are taken in the process, and percent considered passing	(N/A) Does not describe the sampling process	
**IMPORT MONITORING (conducted by government)**		
31. Provides justification for import monitoring at points of entry	(2) Provides justification for import monitoring	VI.2- On import<br>VI.2.1- Purpose of the procedure<br>This procedure is intended to describe the methodology used by the officers of the DCSC during the quality control of oils and fortified flour in manufacturing.
32. Describes protocols and systems for import monitoring	(2) Includes checklists or detailed description of import monitoring procedures	VI.2- On import<br>VI.2.1- Purpose of the procedure<br>This procedure is intended to describe the methodology used by the officers of the DCSC during the quality control of oils and fortified flour in manufacturing.
33. If there are two or more government agencies involved in import monitoring, clarifies the roles and responsibilities between different government agencies in import monitoring	(2) Clarifies roles and responsibilities for more than one agency	The import control is performed by sworn officers of the Division of Consumer Affairs and Consumer Safety (DCSC) following a definite procedure under the supervision of the Division Head. This manual was developed by the officers of the Directorate of Internal Trade in charge of the quality control of food products. It is an important work tool (logbook) for all employees in the quality control of fortified products.

34. If samples are to be taken, describes the sampling process: for example, number of samples, amount, frequency, individual vs. composite, where samples are taken in the process, and percent considered passing	(2) States that samples should be taken as part of import monitoring	This control is however performed by an equipped laboratory depending on the analytical criteria requested.<br>Reference<br>- 68-508 decree of May 7, 1968<br>- Standards for oils and enriched flours<br>- Methods of analysis (analysis and references)<br>Methodology<br>The officer should proceed to send samples to the laboratory for analysis. To do this, he must:<br>- Ensure the adequacy and compliance of the physical samples to be sent;<br>- Provide for control samples (for counter analysis if necessary);<br>- Ensuring proper storage conditions during the transport of samples to the laboratory;<br>- Ensure the laboratory to make the necessary analyzes;<br>- Specify in the packing slip to analyze the criteria and the references (standards) on which the laboratory must be based to assess compliance<br>Results<br>If the test results are consistent, continue the control;<br>If the results are not consistent, reject the DIPA;<br>If challenged by the applicant analyses, control samples are sent to another laboratory for counter analysis.
**ENFORCEMENT/PENALTIES**		
35. Indicates roles and responsibilities in enforcing the legislation	(2) States the role and responsibilities of government in enforcement	Art. 4. — The Minister of State, Minister of Economy and Finance, the Minister of State, Minister of Mines, Industry, Food Processing of Agricultural Products and P.M.E., the Minister of Health, Prevention and Public Hygiene and the Minister of Commerce are charged, each to the extent relevant, with applying the present decree, which will be published in Official Journal.
36. States incentives to start fortification	(0) Does not state	
37. States incentives to continue fortification, including ensuring compliance	(0) Does not state	If no non-compliance is found after the check, congratulate and encourage the operator.
38. States penalties to compel compliance	(2) States any penalties	Art. 3. — Any violation of the provisions of the present decree is punishable with the sanctions provided by law no. 87-47 of 28 December 1987 of the Customs Code and law no. 66-48 of 27 May 1966 regarding the monitoring of food products and the prevention of fraud.
39. Penalties are objectively defined (e.g. first penalty=$100, second penalty=$300)	(0) Penalties are not objectively laid out	If there is a finding of non-compliant raw materials, seize and initiate litigation; cf. Copy of Quality Deception report (QD). In Annex 10. Nothing is clearly laid out, but penalties seem implied.
40. States that enforcement is required to include feedback and support to improve performance and correct noncompliance	(0) Does not require	This might be there, but it's not clearly stated.
**LABORATORY**		
41. References required analytical assays for nutrients (e.g. Liquid chromatography-mass spectrometry for folic acid, atomic absorption for iron and zinc.)	(2) References required assays	V.2.3- Outsourcing<br>Besides the LCI, the DCSC seeks the services of all the competent laboratories operating in the field of agribusiness. This is, inter alia:<br>- The Laboratory of Analysis and Testing of the Polytechnic College (LAE);<br>- Laboratory of the Pasteur Institute of Dakar;<br>- The laboratory of the Institute of Food Technology (ITA);<br>- Laboratory of food hygiene of animal origin EISMV (HIDAOA)<br>However, in the area of fortification, only the Nutrition Laboratory and the ITA have the necessary equipment (HPLC, atomic absorption spectrometer).
42. States recognition that laboratory results are subject to several sources of variation and do not provide conclusive evidence of compliance or noncompliance	(0) Does not state recognition	
43. Focuses on the quantitative analysis of "marker" micronutrients such as iron	(0) Does not state	
**REPORTING**		
44. States how government monitoring results are shared with stakeholders	(0) Does not state how results are shared	

Sierra Leone Wheat

Element	Scoring (use drop-down menus)	Comments (open text)
**GENERAL**		
1. States that legislation applies to at least one food vehicle fit for human consumption (types/grades to be fortified)	(2) States at least one type fit for human consumption	This Sierra Leone standard specifies the requirements for fortified soft wheat flour (*Triticum aestivum* L.)
2. States the public health objective; purpose and scope of legislation	(0) Does not state	
3. References latest available science or accepted international norms and recommendations, particularly for items that may not be covered in the country's documents	(0) Does not state	Sierra Leone Standards only...The following references contain provisions applicable to this Sierra Leone Standard. At the time of publication, the editions indicated were valid.<br><br>All Standards are subject to revision and parties to agreements based on this Standard are encouraged to investigate the possibility of applying the most recent editions of the standards indicated below:<br><br>2.1 SLS 4 General Standard for the labelling of pre-packaged foods.<br><br>2.2 SLS 2 Food Technology – General principles of food hygiene<br><br>2.3 SLS XXX Methods of sampling for milled cereal, pulse and oil-seed products
4. Provides definitions that includes terms that are specific to fortification (e.g. fortified food, premix, fortificant, food vehicle)	(2) States at least one term related to fortification	3.2 fortificant<br>the prescribed compound which provides the specified micronutrient intended to be added to a food vehicle<br>3.3 food vehicle<br>food product intended to be fortified.<br>3.4 micronutrient<br>a natural or synthesized vitamin or mineral or a trace element that is essential for normal growth, development and maintenance of life and of which a deficiency will be detrimental to health.<br>3.5 fortification<br>the addition of one or more micronutrients by means of a fortificant premix to a food stuff, whether or not it is normally contained in the foodstuff, for the purpose of preventing or correcting a demonstrated deficiency of one or more nutrients in the general population or specific population group.<br>3.6 fortificant premix<br>a blend of fortificants and diluents formulated to provide specified and determinable amounts of micronutrients
5. Provides repeals (if there is at least one prior document about fortification)	(N/A) No prior documents about fortification	
6. Provides effective date or gives grace period for when fortification is to begin (e.g. effective 6 months from signing)	(0) Does not state	
**MICRONUTRIENTS/PREMIX**		
7. States nutrients required	(2) States nutrients	Soft wheat flour shall be fortified with vitamins and minerals as indicated in Table 2<br>Table 2: Fortificants for soft wheat flour<br>Fortificants Quantity mg/kg Tolerance<br>Vitamin A 2.0 ±10%<br>Thiamin 8.4 ±10%<br>Riboflavin 4.5 ±10%<br>Niacin 59.0 ±10%<br>Folic Acid 2.08 ±10%<br>Vitamin B 12 0.01 ±10%<br>Iron 58.5 ±10%<br>Zinc 28.3 ±10%<br>*Note:<br>Vitamin A for Wheat flour fortification is a dry, encapsulated form of Vitamin A palmitate containing (75,000 g RE/g). The most common commercial Premix used contains a blend of Vitamin A palmitate, other vitamins and minerals.

8. States fortificants (chemical compounds) to be used (including fortificants that are allowable as options)	(2) States fortificants for at least one nutrient	Soft wheat flour shall be fortified with vitamins and minerals as indicated in Table 2<br>Table 2: Fortificants for soft wheat flour<br>Fortificants Quantity mg/kg Tolerance<br>Vitamin A 2.0 ±10%<br>Thiamin 8.4 ±10%<br>Riboflavin 4.5 ±10%<br>Niacin 59.0 ±10%<br>Folic Acid 2.08 ±10%<br>Vitamin B 12 0.01 ±10%<br>Iron 58.5 ±10%<br>Zinc 28.3 ±10%<br>*Note:<br>Vitamin A for Wheat flour fortification is a dry, encapsulated form of Vitamin A palmitate containing (75,000 g RE/g). The most common commercial Premix used contains a blend of Vitamin A palmitate, other vitamins and minerals.
9. States fortification levels	(2) States a range or number with +/-	Soft wheat flour shall be fortified with vitamins and minerals as indicated in Table 2<br>Table 2: Fortificants for soft wheat flour<br>Fortificants Quantity mg/kg Tolerance<br>Vitamin A 2.0 ±10%<br>Thiamin 8.4 ±10%<br>Riboflavin 4.5 ±10%<br>Niacin 59.0 ±10%<br>Folic Acid 2.08 ±10%<br>Vitamin B 12 0.01 ±10%<br>Iron 58.5 ±10%<br>Zinc 28.3 ±10%<br>*Note:<br>Vitamin A for Wheat flour fortification is a dry, encapsulated form of Vitamin A palmitate containing (75,000 g RE/g). The most common commercial Premix used contains a blend of Vitamin A palmitate, other vitamins and minerals.
10. States consideration of bioavailability/biological activity of fortificants	(0) Does not state any consideration	
11. States consideration of nutrient stability	(0) Does not state any consideration	
**COSTING**		
12. States that the cost of fortification is regulated through cost sharing schemes (between government, industry, consumers) or tax measures [to assist industry]	(0) Does not state any consideration	
13. States consideration of the financial responsibility of monitoring and enforcing fortification (schedule of fees, budget) [on the government side]	(0) Does not state any consideration	
**LABELING**		
14. Includes some sort of statement/label/logo that makes it clear that the product is fortified	(0) Does not include statement, label, or logo	
15. Provides guidance on health claims that can be made for this product (specific to micronutrients added through fortification)	(0) Does not provide	
**INTERNAL MONITORING (conducted by industry)**		
16. If samples are to be taken, describes the sampling process: for example, number of samples, amount, frequency, individual vs. composite, where samples are taken in the process, and percent considered passing	(1) States that samples should be taken (generally)	7.1 Sampling<br>Sampling shall be done in accordance with the provisions of SLS XX Methods of sampling for milled cereal, pulse and oil-seed products<br>7.2 Testing<br>The samples selected in accordance with 7.1 shall be tested for compliance with the requirements of this specification.
17. States that industry is required to follow quality assurance/quality control in regards to fortification	(0) Does not state requirement	
18. States applicability of using qualitative testing to determine the presence or absence of a vitamin or mineral (spot tests, iChecks)	(0) Does not state	7.1 Sampling<br>Sampling shall be done in accordance with the provisions of SLS XX Methods of sampling for milled cereal, pulse and oil-seed products<br>7.2 Testing<br>The samples selected in accordance with 7.1 shall be tested for compliance with the requirements of this specification.
**EXTERNAL MONITORING (conducted by government)**		
19.States requirement for external monitoring at the production site to assure compliance with standards and regulations	(0) Does not state requirement	
20. Describes protocols and systems for regulatory monitoring	(0) Does not describe	

21. If there are two or more government agencies involved in external monitoring, clarifies the roles and responsibilities between different government agencies in external monitoring	(0) Clarifies roles and responsibilities for no agencies	
22. Allows for monitoring to be conducted often enough that problems can be identified and addressed on a timely basis; specifies a timeline for inspections (i.e. once every 6 months, increasing to once every 2 months if a discrepancy is found)	(0) Does not state	
23. If samples are to be taken, describes the sampling process: for example, number of samples, amount, frequency, individual vs. composite, where samples are taken in the process, and percent considered passing	(1) States that samples should be taken (generally)	7.1 Sampling<br>Sampling shall be done in accordance with the provisions of SLS XX Methods of sampling for milled cereal, pulse and oil-seed products<br>7.2 Testing<br>The samples selected in accordance with 7.1 shall be tested for compliance with the requirements of this specification.
24. States applicability of using qualitative testing to determine the presence or absence of a vitamin or mineral (spot tests, iChecks)	(0) Does not state	
25. States registration is required in order to use a logo/be licensed to produce fortified foods	(0) Does not state that registration or licensing is required	
**COMMERCIAL MONITORING (conducted by government)**		
26. Provides justification for commercial monitoring at retail stores	(0) Does not provide justification for commercial monitoring	
27. Describes protocols and systems for commercial monitoring	(0) Does not describe	
28. If there are two or more government agencies involved in commercial monitoring, clarifies the roles and responsibilities between different government agencies in commercial monitoring	(0) Clarifies roles and responsibilities for no agencies	
29. Allows for monitoring to be conducted often enough that problems at the production site or import companies can be identified and addressed on a timely basis; specifies a timeline for inspections (i.e. once every 6 months) or works with production companies to correct noncompliance	(N/A) No commercial monitoring occurs	
30. If samples are to be taken, describes the sampling process: for example, number of samples, amount, frequency, individual vs. composite, where samples are taken in the process, and percent considered passing	(N/A) Does not describe the sampling process	
**IMPORT MONITORING (conducted by government)**		
31. Provides justification for import monitoring at points of entry	(0) Does not provide justification for import monitoring	
32. Describes protocols and systems for import monitoring	(0) Does not state	
33. If there are two or more government agencies involved in import monitoring, clarifies the roles and responsibilities between different government agencies in import monitoring	(0) Clarifies roles and responsibilities for no agencies	
34. If samples are to be taken, describes the sampling process: for example, number of samples, amount, frequency, individual vs. composite, where samples are taken in the process, and percent considered passing	(N/A) Does not describe the sampling process	
**ENFORCEMENT/PENALTIES**		
35. Indicates roles and responsibilities in enforcing the legislation	(0) Does not state	
36. States incentives to start fortification	(0) Does not state	
37. States incentives to continue fortification, including ensuring compliance	(0) Does not state	
38. States penalties to compel compliance	(0) Does not state	
39. Penalties are objectively defined (e.g. first penalty=$100, second penalty=$300)	(N/A) No penalties are stated (Answered (0) to previous question)	
40. States that enforcement is required to include feedback and support to improve performance and correct noncompliance	(0) Does not require	
**LABORATORY**		
41. References required analytical assays for nutrients (e.g. Liquid chromatography-mass spectrometry for folic acid, atomic absorption for iron and zinc.)	(0) Does not state requirements	
42. States recognition that laboratory results are subject to several sources of variation and do not provide conclusive evidence of compliance or noncompliance	(0) Does not state recognition	
43. Focuses on the quantitative analysis of "marker" micronutrients such as iron	(0) Does not state	
**REPORTING**		
44. States how government monitoring results are shared with stakeholders	(0) Does not state how results are shared	

Solomon Islands Wheat

Element	Scoring (use drop-down menus)	Comments (open text)
**GENERAL**		
1. States that legislation applies to at least one food vehicle fit for human consumption (types/grades to be fortified)	(2) States at least one type fit for human consumption	This standard applies to (a) wheat flour for direct human consumption prepared from common wheat, Triticum aestivum L., or club wheat, Triticum compactum Host., or mixtures thereof, by grinding or milling processes in which the bran and germ are partly removed and the remainder is comminuted to a suitable degree of fineness, and which is prepackaged ready for sale to the consumer or destined for use in other food products; and (b) whole meal, whole-wheat flour or semolina, milled from common wheat, Triticum aestivum L., or club wheat, Triticum compactum Host., or mixtures thereof, and which is prepackaged ready for sale to the consumer or destined for use in other food products.
2. States the public health objective; purpose and scope of legislation	(0) Does not state	
3. References latest available science or accepted international norms and recommendations, particularly for items that may not be covered in the country's documents	(0) Does not state	Where no such standards are prescribed, the Codex General Standard on Food Additives applies
4. Provides definitions that includes terms that are specific to fortification (e.g. fortified food, premix, fortificant, food vehicle)	(0) Does not state	Food Law Part 1, section 2. "In these Regulations unless the context otherwise requires-" and then goes on to define terms. Fortification-specific terms are not addressed in definitions.
5. Provides repeals (if there is at least one prior document about fortification)	(0) Does not state	
6. Provides effective date or gives grace period for when fortification is to begin (e.g. effective 6 months from signing)	(2) States effective date or grace period for when fortification is to begin (e.g. effective 6 months from signing)	These regulations may be cited as the Pure Food (Food Control) regulations 2010 and commence on the date of publication in the Gazette. One year grace period for class 1 businesses and 3 years for class 2 businesses to prepare food safety plans based on HACCP
**MICRONUTRIENTS/PREMIX**		
7. States nutrients required	(2) States nutrients	See section 4a-c;
8. States fortificants (chemical compounds) to be used (including fortificants that are allowable as options)	(2) States fortificants for at least one nutrient	See section 4a-c; 4b discusses allowable options for iron
9. States fortification levels	(1) States one number only	See section 4a-c. No range given.
10. States consideration of bioavailability/biological activity of fortificants	(0) Does not state any consideration	
11. States consideration of nutrient stability	(0) Does not state any consideration	
**COSTING**		
12. States that the cost of fortification is regulated through cost sharing schemes (between government, industry, consumers) or tax measures [to assist industry]	(0) Does not state any consideration	
13. States consideration of the financial responsibility of monitoring and enforcing fortification (schedule of fees, budget) [on the government side]	(0) Does not state any consideration	
**LABELING**		
14. Includes some sort of statement/label/logo that makes it clear that the product is fortified	(2) Includes a statement, label, or logo	Fortified wheat flour shall be labeled as "Fortified" or "Enriched" and shall identify the ingredients added for fortification purposes and shall specify the concentration in which it is present in the final product.
15. Provides guidance on health claims that can be made for this product (specific to micronutrients added through fortification)	(2) Provides guidance on health claims specific to micronutrients added through fortification	Section 21: Prohibited claims. (h). "a claim that a food has obtained an increased or special nutritive value by means of the addition of nutrients, such as vitamins, minerals and amino acids shall be permitted only if such an addition has been made on the basis on nutritional considerations according to the Codex General Principles for the Addition of Essential Nutrients to Foods. Section 22 outlines use of nutrient content claim, comparative claims, nutrition claims, and health claims. First Schedule provides guidance
**INTERNAL MONITORING (conducted by industry)**		
16. If samples are to be taken, describes the sampling process: for example, number of samples, amount, frequency, individual vs. composite, where samples are taken in the process, and percent considered passing	(N/A) Does not describe the sampling process	

		(c) the food products have been produced under conditions of good manufacturing practice (GMP) and the company has employed hazard analysis control point system (HACCP) or their equivalent to assure the safety of the food produced. The production of the described products has been supervised by an authorised inspector of the Department of Environmental Health to assure compliance with GMP and HACCP; or have been produced under conditions of good manufacturing practice (GMP) and the company has employed hazard analysis critical control point system (HACCP) or their equivalent to assure the safety of the food produced. The company is part of an approved quality assurance programme and the described products were produced in accordance with this programme in a processing plant registered as part of the programme. The programme is routinely evaluated by authorised inspectors of the Department of Environmental Health.
17. States that industry is required to follow quality assurance/quality control in regards to fortification	(0) Does not state requirement	
18. States applicability of using qualitative testing to determine the presence or absence of a vitamin or mineral (spot tests, iChecks)	(0) Does not state	
**EXTERNAL MONITORING (conducted by government)**		
19.States requirement for external monitoring at the production site to assure compliance with standards and regulations	(2) States requirement for external monitoring or the need for audits/inspections	Requires inspections - 19th schedule for frequency and fees paid by food businesses
20. Describes protocols and systems for regulatory monitoring	(1) Does not explicitly describe, but references protocols and systems for regulatory monitoring	Hints at this, but nothing concrete
21. If there are two or more government agencies involved in external monitoring, clarifies the roles and responsibilities between different government agencies in external monitoring	(0) Clarifies roles and responsibilities for no agencies	
22. Allows for monitoring to be conducted often enough that problems can be identified and addressed on a timely basis; specifies a timeline for inspections (i.e. once every 6 months, increasing to once every 2 months if a discrepancy is found)	(2) Describes frequency and how it is responsive to the needs of industry or the stage of fortification implementation	"In accordance with the requirements of section 10 of the Act, the frequency with which an appropriate enforcement agency shall undertake inspections of premises and the fees for service to be charged are prescribed in the Nineteenth Schedule" Class I sites receive 4 inspections per annum and Class II sites receive 3 inspections per annum. Sites where defects are found will be inspected more often and forced to pay an additional $50 per inspection.
23. If samples are to be taken, describes the sampling process: for example, number of samples, amount, frequency, individual vs. composite, where samples are taken in the process, and percent considered passing	(N/A) Does not describe the sampling process	
24. States applicability of using qualitative testing to determine the presence or absence of a vitamin or mineral (spot tests, iChecks)	(0) Does not state	
25. States registration is required in order to use a logo/be licensed to produce fortified foods	(2) Describes some type of registration or licensing	Some sites are registered with HACCP and receive regular inspections with that program. Importing companies must notify the Director before attempting to import food.
**COMMERCIAL MONITORING (conducted by government)**		
26. Provides justification for commercial monitoring at retail stores	(0) Does not provide justification for commercial monitoring	
27. Describes protocols and systems for commercial monitoring	(0) Does not describe	
28. If there are two or more government agencies involved in commercial monitoring, clarifies the roles and responsibilities between different government agencies in commercial monitoring	(0) Clarifies roles and responsibilities for no agencies	
29. Allows for monitoring to be conducted often enough that problems at the production site or import companies can be identified and addressed on a timely basis; specifies a timeline for inspections (i.e. once every 6 months) or works with production companies to correct noncompliance	(0) Does not state	
30. If samples are to be taken, describes the sampling process: for example, number of samples, amount, frequency, individual vs. composite, where samples are taken in the process, and percent considered passing	(N/A) Does not describe the sampling process	
**IMPORT MONITORING (conducted by government)**		

31. Provides justification for import monitoring at points of entry	(2) Provides justification for import monitoring	Importing food. 22. (1) An agriculture, quarantine, or health inspector shall, immediately upon receipt of documentation addressing the importation of food for sale, inform the health inspector responsible for inspection of imported food. (2) A person intending to import food for sale shall also notify the Director in writing of his intention to import food. The Director shall, in such cases, direct and cause an appropriate health inspector to carry out any inspection and sampling required. (3) The health inspector identified in subsections (1) and (2) shall review relevant documents including export certifications provided by competent overseas authorities and carry out any inspection and sampling required, as soon as possible and in a manner so as not to unnecessarily delay the release of the food from customs and quarantine
32. Describes protocols and systems for import monitoring	(1) Does not explicitly state, but references protocols and systems for import monitoring	
33. If there are two or more government agencies involved in import monitoring, clarifies the roles and responsibilities between different government agencies in import monitoring	(0) Clarifies roles and responsibilities for no agencies	
34. If samples are to be taken, describes the sampling process: for example, number of samples, amount, frequency, individual vs. composite, where samples are taken in the process, and percent considered passing	(N/A) Does not describe the sampling process	
**ENFORCEMENT/PENALTIES**		
35. Indicates roles and responsibilities in enforcing the legislation	(2) States the role and responsibilities of government in enforcement	Part V and Part VII detail general enforcement procedures and legal proceedings, respectively. Talks about health inspectors.
36. States incentives to start fortification	(0) Does not state	
37. States incentives to continue fortification, including ensuring compliance	(0) Does not state	
38. States penalties to compel compliance	(2) States any penalties	Sites where defects are found will be inspected more often and forced to pay an additional $50 per inspection. (Nineteenth schedule). Also, general penalties outlined in section 68
39. Penalties are objectively defined (e.g. first penalty=$100, second penalty=$300)	(2) Penalties are objectively laid out in the document (e.g. first penalty=$100, second penalty=$300)	A person who is guilty of an offence against this Act for which no other penalty is prescribed is liable to a maximum penalty of one thousand dollars or imprisonment for a term of six months or both, and in the case of a second or subsequent offence a maximum penalty of one thousand five hundred dollars or imprisonment for a term of six months or both.
40. States that enforcement is required to include feedback and support to improve performance and correct noncompliance	(0) Does not require	
**LABORATORY**		
41. References required analytical assays for nutrients (e.g. Liquid chromatography-mass spectrometry for folic acid, atomic absorption for iron and zinc.)	(0) Does not state requirements	
42. States recognition that laboratory results are subject to several sources of variation and do not provide conclusive evidence of compliance or noncompliance	(0) Does not state recognition	
43. Focuses on the quantitative analysis of "marker" micronutrients such as iron	(0) Does not state	
**REPORTING**		
44. States how government monitoring results are shared with stakeholders	(0) Does not state how results are shared	Reports appear to be for internal use only.

South Africa Wheat

Element	Scoring (use drop-down menus)	Comments (open text)
**GENERAL**		
1. States that legislation applies to at least one food vehicle fit for human consumption (types/grades to be fortified)	(2) States at least one type fit for human consumption	The Minister for Health has, in terms of Section 15 (1) of the Foodstuffs, Cosmetics and Disinfectants Act, 1972 (Act No. 54 of 1972), made the regulations in the Schedule. (a) The formulation of the fortification mix for wheat flour based on the micronutrient requirements specified in Annexure V, Table 4a shall be as follows: Table 1.
2. States the public health objective; purpose and scope of legislation	(0) Does not state	
3. References latest available science or accepted international norms and recommendations, particularly for items that may not be covered in the country's documents	(2) States the documents referenced	ensure that the quality standard for diluents and fortificants, independently or mixed with a diluent shall be in accordance with the standards as determined in the latest edition of Food Chemicals Codex (FCC).
4. Provides definitions that includes terms that are specific to fortification (e.g. fortified food, premix, fortificant, food vehicle)	(2) States at least one term related to fortification	In these regulations any word or expression defined in the Act and not defined herein bears the same meaning as in the Act and unless the context otherwise indicates-
5. Provides repeals (if there is at least one prior document about fortification)	(2) States repeals	13. The Regulations on the Enrichment of Maize Meal, promulgated under Government notice No. R 2839 of 21 December 1979 are hereby repealed.
6. Provides effective date or gives grace period for when fortification is to begin (e.g. effective 6 months from signing)	(2) States effective date or grace period for when fortification is to begin (e.g. effective 6 months from signing)	14. These regulations shall come into operation 6 months after the date of final publication.
**MICRONUTRIENTS/PREMIX**		
7. States nutrients required	(2) States nutrients	Special Provisions 9a, Table 1
8. States fortificants (chemical compounds) to be used (including fortificants that are allowable as options)	(2) States fortificants for at least one nutrient	Special Provisions 9a, Table 1
9. States fortification levels	(2) States a range or number with +/-	Special Provisions 9a, Table 1. No range given, but tolerances are detailed in Annexure VI
10. States consideration of bioavailability/biological activity of fortificants	(0) Does not state any consideration	
11. States consideration of nutrient stability	(2) States consideration of nutrient stability	Is vitamin A compound stable as per specification? (Attach findings of<br>internal vitamin A stability trials for each source as indicated in<br>question 17)*<br>* For more information on the vitamin A stability specification, contact the<br>Directorate: Nutrition, Department of Health."
**COSTING**		
12. States that the cost of fortification is regulated through cost sharing schemes (between government, industry, consumers) or tax measures [to assist industry]	(0) Does not state any consideration	
13. States consideration of the financial responsibility of monitoring and enforcing fortification (schedule of fees, budget) [on the government side]	(0) Does not state any consideration	
**LABELING**		
14. Includes some sort of statement/label/logo that makes it clear that the product is fortified	(2) Includes a statement, label, or logo	(b) the claim "Fortified for better health" and the official fortification logo to that effect as indicated in Annexure VII are reserved only for food vehicles, that have been identified in these regulations and may be displayed on the label or in an advertising material;<br>(c) any person who uses the official logo referred to in Annexure VII on labels or in advertising material for foodstuffs other than in accordance with these regulations or any other regulations made in terms of the Act, shall be guilty of an offence.
15. Provides guidance on health claims that can be made for this product (specific to micronutrients added through fortification)	(2) Provides guidance on health claims specific to micronutrients added through fortification	In addition to the Regulations Governing the Advertising and Labelling of Foodstuffs made under the Act, all food vehicles shall be labelled as follows:<br>(a) the claim "enriched with" or "enriched" may only be used in addition to the word "fortified" on one label in cases where a micronutrient other than the specified fortificants is added to a food vehicle or in cases where at least 15% more than the prescribed amounts of fortificants are added to a food vehicle;<br>(b) the claim "Fortified for better health" and the official fortification logo to that effect as indicated in Annexure VII are reserved only for food vehicles, that have been identified in these regulations and may be displayed on the label or in an advertising material;
**INTERNAL MONITORING (conducted by industry)**		

16. If samples are to be taken, describes the sampling process: for example, number of samples, amount, frequency, individual vs. composite, where samples are taken in the process, and percent considered passing	(2) States that samples should be taken as part of internal monitoring	submit one 500 g sample of a fortification mix every six months for each food vehicle to a laboratory that has accreditation for the methods of analysis as indicated by the fortification mix manufacturer, importer or supplier from SANAS or another international accreditation body; keep the analysis report on record and submit a copy of the report to the Director-General; and
17. States that industry is required to follow quality assurance/quality control in regards to fortification	(2) States requirement of QA/QC for fortification	Annexure II implies that manufacturers or suppliers of fortification mixes are responsible for the listed quality control principles 4. ensure that all critical stages of the manufacturing process are monitored to ensure that the correct dosage levels are maintained through the following measures:<br>(a) checking of fortification mix feeders at least once a day to ensure that they are delivering the correct dosage levels;<br>(b) performing visual checks at least twice per shift to ensure that fortification mixes are being used and that no blockages have occurred and keeping record of this;<br>(c) performing two-hourly spot checks to ensure that the product has been dosed correctly by determining one of the components of a fortification mix according to the appropriate analytical method.
18. States applicability of using qualitative testing to determine the presence or absence of a vitamin or mineral (spot tests, iChecks)	(2) States applicability of spot test to determine presence/absence of vitamin or mineral specific to internal monitoring	(internal) performing two-hourly spot checks to ensure that the product has been dosed correctly by determining one of the components of a fortification mix according to the appropriate analytical method.
**EXTERNAL MONITORING (conducted by government)**		
19.States requirement for external monitoring at the production site to assure compliance with standards and regulations	(2) States requirement for external monitoring or the need for audits/inspections	At least two inspection audits per year, including the taking of samples for laboratory analysis, in respect of all registered fortification mix manufacturers, suppliers and importers. to monitor compliance with the Regulations relating to the Fortification of Certain Foodstuffs shall be conducted at their premises and the manufacturers, suppliers or importers themselves shall bear the costs of such audits and analysis.
20. Describes protocols and systems for regulatory monitoring	(2) Includes checklists or provides detailed description of regulatory monitoring procedures	At least two inspection audits per year, including the taking of samples for laboratory analysis, in respect of all registered fortification mix manufacturers, suppliers and importers. to monitor compliance with the Regulations relating to the Fortification of Certain Foodstuffs shall be conducted at their premises and the manufacturers, suppliers or importers themselves shall bear the costs of such audits and analysis.
21. If there are two or more government agencies involved in external monitoring, clarifies the roles and responsibilities between different government agencies in external monitoring	(0) Clarifies roles and responsibilities for no agencies	
22. Allows for monitoring to be conducted often enough that problems can be identified and addressed on a timely basis; specifies a timeline for inspections (i.e. once every 6 months, increasing to once every 2 months if a discrepancy is found)	(1) Makes mention of a timeline	At least two inspection audits per year, including the taking of samples for laboratory analysis, in respect of all registered fortification mix manufacturers, suppliers and importers. to monitor compliance with the Regulations relating to the Fortification of Certain Foodstuffs shall be conducted at their premises and the manufacturers, suppliers or importers themselves shall bear the costs of such audits and analysis.
23. If samples are to be taken, describes the sampling process: for example, number of samples, amount, frequency, individual vs. composite, where samples are taken in the process, and percent considered passing	(2) States that samples should be taken as part of external monitoring	A sample of a fortified food vehicle, taken by an inspector in terms of the Act, shall be analysed for the amounts of nicotinamide / niacinamide, or riboflavin and retinol / vitamin A palmitate, and the results of such a sample shall be considered as representative of the standards prescribed by these Regulations in Table 3 and Table 4.
24. States applicability of using qualitative testing to determine the presence or absence of a vitamin or mineral (spot tests, iChecks)	(0) Does not state	
25. States registration is required in order to use a logo/be licensed to produce fortified foods	(2) Describes some type of registration or licensing	South Africa requires premix producers to be registered and then keep records of their distribution to mills
**COMMERCIAL MONITORING (conducted by government)**		
26. Provides justification for commercial monitoring at retail stores	(0) Does not provide justification for commercial monitoring	
27. Describes protocols and systems for commercial monitoring	(0) Does not describe	
28. If there are two or more government agencies involved in commercial monitoring, clarifies the roles and responsibilities between different government agencies in commercial monitoring	(0) Clarifies roles and responsibilities for no agencies	

29. Allows for monitoring to be conducted often enough that problems at the production site or import companies can be identified and addressed on a timely basis; specifies a timeline for inspections (i.e. once every 6 months) or works with production companies to correct noncompliance	(0) Does not state	
30. If samples are to be taken, describes the sampling process: for example, number of samples, amount, frequency, individual vs. composite, where samples are taken in the process, and percent considered passing	(N/A) Does not describe the sampling process	
**IMPORT MONITORING (conducted by government)**		
31. Provides justification for import monitoring at points of entry	(2) Provides justification for import monitoring	Any person who manufactures, imports, or sells foodstuffs identified as food vehicles which have not been fortified in accordance with these regulations, including the requirements specified in Annexure I, shall be guilty of an offence.
32. Describes protocols and systems for import monitoring	(1) Does not explicitly state, but references protocols and systems for import monitoring	Registered manufacturers, importers or suppliers of fortification mixes shall issue a certificate of compliance as indicated in Annexure IV.
33. If there are two or more government agencies involved in import monitoring, clarifies the roles and responsibilities between different government agencies in import monitoring	(2) Clarifies roles and responsibilities for more than one agency	See Act 54, Reg 14 - if suspected not in compliance, Commissioner may allow the items to pass from his control to another location approved by the DG and detailed for examination or analysis until the result is known. Commissioner should not allow anything to pass from his control unless the importer has furnished the DG with a guarantee that he will pay if he fails to comply. If analysis determines that it can't be imported, it is confiscated, destroyed, returned to place or origin, or otherwise disposed of
34. If samples are to be taken, describes the sampling process: for example, number of samples, amount, frequency, individual vs. composite, where samples are taken in the process, and percent considered passing	(N/A) Does not describe the sampling process	
**ENFORCEMENT/PENALTIES**		
35. Indicates roles and responsibilities in enforcing the legislation	(2) States the role and responsibilities of government in enforcement	Sections 10 and 11 of Act 54, Section 19
36. States incentives to start fortification	(0) Does not state	
37. States incentives to continue fortification, including ensuring compliance	(0) Does not state	
38. States penalties to compel compliance	(2) States any penalties	Section 18 states penalties
39. Penalties are objectively defined (e.g. first penalty=$100, second penalty=$300)	(2) Penalties are objectively laid out in the document (e.g. first penalty=$100, second penalty=$300)	Yes, details first, second, third convictions, and gives limit of fine or maximum imprisonment
40. States that enforcement is required to include feedback and support to improve performance and correct noncompliance	(0) Does not require	
**LABORATORY**		
41. References required analytical assays for nutrients (e.g. Liquid chromatography-mass spectrometry for folic acid, atomic absorption for iron and zinc.)	(2) References required assays	Only accredited analytical methods for which an original certificate or certified copy from SANAS or another internationally accreditation body has been obtained and which are attached to the application (Annexure III) will be accepted.
42. States recognition that laboratory results are subject to several sources of variation and do not provide conclusive evidence of compliance or noncompliance	(0) Does not state recognition	
43. Focuses on the quantitative analysis of "marker" micronutrients such as iron	(2) Focuses on quantitative analysis of marker micronutrient such as iron	A sample of a fortified food vehicle, taken by an inspector in terms of the Act, shall be analysed for the amounts of nicotinamide / niacinamide, or riboflavin and retinol / vitamin A palmitate, and the results of such a sample shall be considered as representative of the standards prescribed by these Regulations in Table 3 and Table 4.
**REPORTING**		
44. States how government monitoring results are shared with stakeholders	(0) Does not state how results are shared	

Tanzania Wheat

Element	Scoring (use drop-down menus)	Comments (open text)
**GENERAL**		
1. States that legislation applies to at least one food vehicle fit for human consumption (types/grades to be fortified)	(2) States at least one type fit for human consumption	Standard is specific to wheat flour
2. States the public health objective; purpose and scope of legislation	(2) States the public health objective or general purpose of legislation	Wheat flour is the most important essential ingredient in the manufacture of breads, biscuits, cakes, pastries and other food products. The safety and quality of the flour have an immense bearing on the safety and quality of these end products. Therefore to ensure safety and good quality wheat flour, this standard has been prepared. Micronutrient malnutrition is a problem of public health significance in Tanzania affecting a large number of children and women of child bearing age, but it is debilitating for all ages and for the national economy as well.
3. References latest available science or accepted international norms and recommendations, particularly for items that may not be covered in the country's documents	(2) States the documents referenced	In the preparation of this Tanzania Standard assistance was derived from:<br>KS 05-169 – 1986 – Specification for wheat flour, published by the Kenya Bureau of Standards.<br>Codex standard 152:1995, Wheat flour, published by the Codex Alimentarius Commission.<br>ISO 7495: 1990 Wheat flour – Determination of wet gluten content by mechanical means, published by the International Organization for Standardization.<br>ISO 5530-1:1997– Wheat flour – Physical characteristics of dough – Part 1 – Determination of water absorption and rheological properties using radiography, published by the International Organization for Standardization.<br>Official Methods of Analysis, 18th Edition 2010, published by the Association of Official Analytical Chemists.
4. Provides definitions that includes terms that are specific to fortification (e.g. fortified food, premix, fortificant, food vehicle)	(2) States at least one term related to fortification	Section 3 on terminology
5. Provides repeals (if there is at least one prior document about fortification)	(2) States repeals	This second edition cancels and replaces the first edition (TZS 439:2006) which has been technically revised.
6. Provides effective date or gives grace period for when fortification is to begin (e.g. effective 6 months from signing)	(2) States effective date or grace period for when fortification is to begin (e.g. effective 6 months from signing)	DEAS 767 - Within 6 months of a declaration of an EA Standard, Partner States shall adopt the approved text without deviation and withdraw any existing national standard
**MICRONUTRIENTS/PREMIX**		
7. States nutrients required	(2) States nutrients	Table 2
8. States fortificants (chemical compounds) to be used (including fortificants that are allowable as options)	(2) States fortificants for at least one nutrient	Table 2
9. States fortification levels	(2) States a range or number with +/-	Table 2, gives range around factory levels
10. States consideration of bioavailability/biological activity of fortificants	(0) Does not state any consideration	
11. States consideration of nutrient stability	(2) States consideration of nutrient stability	Stability is defined as the di fference in time, to the nearest 0.5 min, between the point at<br>which the top of the curve fi rst intercepts the 500 FU line and the point at which the top of the<br>curve leaves the 500 FU line. This value, in general, gives some indication of the tolerance of<br>the flour to mixing.
**COSTING**		
12. States that the cost of fortification is regulated through cost sharing schemes (between government, industry, consumers) or tax measures [to assist industry]	(0) Does not state any consideration	
13. States consideration of the financial responsibility of monitoring and enforcing fortification (schedule of fees, budget) [on the government side]	(2) Shows consideration that monitoring costs money	The monitoring manual states very clearly that costs for monitoring should be planned for, but it doesn't necessarily indicate that adequate funds are dedicated to monitoring
**LABELING**		
14. Includes some sort of statement/label/logo that makes it clear that the product is fortified	(2) Includes a statement, label, or logo	9 Labeling<br>In addition to the requirements given in TZS 538 (see clause 2), each bag shall be labeled to contain the under mentioned information: o) Explicit fortification logo in case of fortified wheat flour.
15. Provides guidance on health claims that can be made for this product (specific to micronutrients added through fortification)	(2) Provides guidance on health claims specific to micronutrients added through fortification	9.2 Nutrition and health claims<br>Fortified wheat flour may have claims on the importance of vitamin A in nutrition and health. Such claims when declared should be consistent with national legislation or standards on nutrition and health claims. Where no national standards or legislation exists such claims should conform to TZS 1340 (see clause 2) and guidelines for use of nutrition and health claims (CAC/GL 23-1997).
**INTERNAL MONITORING (conducted by industry)**		

16. If samples are to be taken, describes the sampling process: for example, number of samples, amount, frequency, individual vs. composite, where samples are taken in the process, and percent considered passing	(2) States that samples should be taken as part of internal monitoring	3.2.2, 3.2.3 (3 daily composite samples; 1 sample from production, 8 samples from storage warehouse (combined)). Combine all five composite samples and divide into three portions of 500g
17. States that industry is required to follow quality assurance/quality control in regards to fortification	(2) States requirement of QA/QC for fortification	Manufacturer of a fortified food shall develop, maintain and routinely follow procedures for safety and quality assurance throughout the manufacturing process to ensure that the final product complies with these regulations and any other regulations made under the Act.
18. States applicability of using qualitative testing to determine the presence or absence of a vitamin or mineral (spot tests, iChecks)	(2) States applicability of spot test to determine presence/absence of vitamin or mineral specific to internal monitoring	Internal monitoring manual - Section 3.3.2 - iron spot tests
**EXTERNAL MONITORING (conducted by government)**		
19.States requirement for external monitoring at the production site to assure compliance with standards and regulations	(2) States requirement for external monitoring or the need for audits/inspections	Technical auditing and inspection activities are carried out at manufacturing facilities as part of the enforcement activities performed by the regulatory Authority in order to ensure that fortified foods meets the nutrient quality and safety specifications established in regulations. During the technical audits, the performance of quality assurance and quality control activities conducted by the producer is examined. The conformity of the fortified food with technical specifications is determined through sampling and chemical analysis of fortified food samples collected from the factory. Samples of premix/fortification may also be taken to verify the validity of the Certificate of Analysis (COA) provided by the supplier.
20. Describes protocols and systems for regulatory monitoring	(2) Includes checklists or provides detailed description of regulatory monitoring procedures	Checklist for inspection of maize/wheat flour manufacturing facilities
21. If there are two or more government agencies involved in external monitoring, clarifies the roles and responsibilities between different government agencies in external monitoring	(N/A) Only one government agency involved	The people directly responsible for achievement of these objectives are the Authority Inspectors, who should pass on the results of the inspection to their supervisor of inspection activities. The Supervisor is responsible for preparing the reports to the factories and reporting every six months to the Head of the regulatory Authority.
22. Allows for monitoring to be conducted often enough that problems can be identified and addressed on a timely basis; specifies a timeline for inspections (i.e. once every 6 months, increasing to once every 2 months if a discrepancy is found)	(2) Describes frequency and how it is responsive to the needs of industry or the stage of fortification implementation	plan at least two yearly inspection to manufacturing facilities. The Authority should then conduct a comprehensive follow-up inspection inspection. , inspectioninspection to assess implementation of corrective actions. The follow up inspectioninspectioninspection, which may take place ahead of schedule if the identified limitations are considered serious
23. If samples are to be taken, describes the sampling process: for example, number of samples, amount, frequency, individual vs. composite, where samples are taken in the process, and percent considered passing	(2) States that samples should be taken as part of external monitoring	Take a 50g sample of the fortificant/premix that is being used for fortification at the factory during the time of inspection. Label it with the name of the mill, name of the manufacturer, micronutrient content indicated, and date of sample collection. 500g of all other samples
24. States applicability of using qualitative testing to determine the presence or absence of a vitamin or mineral (spot tests, iChecks)	(2) States applicability of spot test to determine presence/absence of vitamin or mineral specific to external monitoring	External monitoring manual - Section 2.2.3.d/4.3.2.b
25. States registration is required in order to use a logo/be licensed to produce fortified foods	(2) Describes some type of registration or licensing	List of approved brands per food. This list should be updated every three months or more frequent if needed. Use Second Schedule to apply for license
**COMMERCIAL MONITORING (conducted by government)**		
26. Provides justification for commercial monitoring at retail stores	(2) Provides justification for commercial monitoring	Inspection of food outlets is conducted in order to verify legal compliance of fortified foods sold in retail supermarkets, markets, grocery stores, and wholesale stores. This monitoring allows for the detection in the market of brands that are not approved by the Authority or do not comply with local fortification regulations. It also helps to confirm whether brands that have previously been inspected in factories and importation sites are indeed fulfilling the requirements as claimed by inspectors during the external monitoring process.
27. Describes protocols and systems for commercial monitoring	(2) Includes checklists or provides detailed description of regulatory monitoring procedures	Take samples of approved fortified foods and conduct spot test for iron and where necessary, take samples for quantitative analysis at TFDA head quarters. They should fill in the form F5/TFDA/DFS/FI&E/GL/001 of these guidelines
28. If there are two or more government agencies involved in commercial monitoring, clarifies the roles and responsibilities between different government agencies in commercial monitoring	(2) Clarifies roles and responsibilities for more than one agency	Manual - responsibility of regulatory authority in cooperation with local authorities, involving local inspectors carrying out audit and inspection and taking samples.

29. Allows for monitoring to be conducted often enough that problems at the production site or import companies can be identified and addressed on a timely basis; specifies a timeline for inspections (i.e. once every 6 months) or works with production companies to correct noncompliance	(1) Makes mention of a timeline	"Results of commercial monitoring activities should be consolidated in reports to be issued on quarterly basis. The reports assist defining the degree of success in fulfilling the fortification goals and spells out obstacles that need to be overcome and actions to be taken. It is further recommended that an annual report be prepared and published where data is presented graphically to describe the status of the fortification program in the country, along with information from other general food control or surveillance activities."
30. If samples are to be taken, describes the sampling process: for example, number of samples, amount, frequency, individual vs. composite, where samples are taken in the process, and percent considered passing	(2) States that samples should be taken as part of commercial monitoring	Choose a sealed packaged of about 500gm or 500ml of each brand of each fortified food in the store. If the food is not available in such quantities, take the nearest larger retail-size presentation. If packages are much smaller, collect sufficient packages to make up the specified weight; (e.g. 2 packages of 250 gm).
**IMPORT MONITORING (conducted by government)**		
31. Provides justification for import monitoring at points of entry	(2) Provides justification for import monitoring	The purpose of monitoring fortificants and fortified foods prior to authorization for entry is:-<br>a) To ensure that the imported products are accompanied by adequate documentation to certify that national standards and regulations are being fulfilled.<br>b) To confirm that the food complies with fortification requirements based on the presence of one or more key micronutrients in the imported fortified food.
32. Describes protocols and systems for import monitoring	(2) Includes checklists or detailed description of import monitoring procedures	4.3.3 Taking decisions to authorize<br>a) All samples should test positive for the indicator micronutrient.<br>b) If samples fail the qualitative test or fail to comply in terms of proper documentation and labeling requirements, the affected brand should not be allowed to enter the country.<br>c) If documentation is correct, and samples show the presence of the key micronutrient, authorize importation.
33. If there are two or more government agencies involved in import monitoring, clarifies the roles and responsibilities between different government agencies in import monitoring	(N/A) Only one government agency involved	Supervisor of food inspection activities at the port of entry should ensure that the following tasks are performed; collection of samples, testing and reviewing documentation before the food can be allowed to enter the country
34. If samples are to be taken, describes the sampling process: for example, number of samples, amount, frequency, individual vs. composite, where samples are taken in the process, and percent considered passing	(2) States that samples should be taken as part of import monitoring	4.3.3 Taking decisions to authorize<br>a) All samples should test positive for the indicator micronutrient.<br>b) If samples fail the qualitative test or fail to comply in terms of proper documentation and labeling requirements, the affected brand should not be allowed to enter the country.<br>c) If documentation is correct, and samples show the presence of the key micronutrient, authorize importation.
**ENFORCEMENT/PENALTIES**		
35. Indicates roles and responsibilities in enforcing the legislation	(2) States the role and responsibilities of government in enforcement	The minister may at any time vary, amend or revoke any schedule prescribed in these regulations In the National Food Fortification Program, the Tanzania Food and Drugs Authority has a role to regulate fortified food in the market. These Guidelines have been developed in order to enable the inspectors to conduct effective external monitoring of fortified foods.
36. States incentives to start fortification	(0) Does not state	
37. States incentives to continue fortification, including ensuring compliance	(0) Does not state	
38. States penalties to compel compliance	(2) States any penalties	Any person who contravenes any provision of these regulations commits an offence under the Act. (points 11 and 12 in the Food Act) (fines, lose license)
39. Penalties are objectively defined (e.g. first penalty=$100, second penalty=$300)	(0) Penalties are not objectively laid out	If the factory is found to have not taken any action to solve the problem or if there is proof that the noncompliance is intentional, action should be taken against the factory and this could vary from a written warning to legal action such as a fine and other actions as deemed necessary.
40. States that enforcement is required to include feedback and support to improve performance and correct noncompliance	(2) Requires any feedback/support to improve performance	When a minor non-compliance is found, technical advice should be provided to the production manager on areas that need improvement and follow up with more frequent inspections.
**LABORATORY**		
41. References required analytical assays for nutrients (e.g. Liquid chromatography-mass spectrometry for folic acid, atomic absorption for iron and zinc.)	(2) References required assays	DEAS 767 - Testing conducted using any validated ECSA methods

42. States recognition that laboratory results are subject to several sources of variation and do not provide conclusive evidence of compliance or noncompliance	(2) States recognition that lab results are subject to variation	d) When results are received from the laboratory, compare them with producer's records. Remember that the results from the producer were obtained using semi quantitative methods while the laboratory uses quantitative method. Therefore some variation between the two results is expected. However, if results differ greatly, for example, iron level reported quantitatively was less than the legal minimum and the daily estimated average was greater than 20 mg/kg, the cause of such discrepancy should be investigated. e) Analyze the results and complete the report. The analytical results for ALL five samples should be randomly distributed within acceptable range, irrespective of whether they are samples from production of the day, from storage warehouse or from composite samples of the month. Any significant discrepancy between samples collected during inspection and those stored as daily composite samples should be a cause for concern and should be investigated during next inspection inspection. Prepare letters to advise the inspected factories of the problem.
43. Focuses on the quantitative analysis of "marker" micronutrients such as iron	(2) Focuses on quantitative analysis of marker micronutrient such as iron	4.1.2 Qualitative methods Iron (external lab)<br>4.1.3 Quantitative method Vit. A (external lab)
**REPORTING**		
44. States how government monitoring results are shared with stakeholders	(0) Does not state how results are shared	

Togo Wheat

Element	Scoring (use drop-down menus)	Comments (open text)
**GENERAL**		
1. States that legislation applies to at least one food vehicle fit for human consumption (types/grades to be fortified)	(2) States at least one type fit for human consumption	Article I: Enriching with micro-nutrients is compulsory for the following foodstuffs:<br>- refined edible oils;<br>- wheat flour.
2. States the public health objective; purpose and scope of legislation	(0) Does not state	
3. References latest available science or accepted international norms and recommendations, particularly for items that may not be covered in the country's documents	(2) States the documents referenced	Article 3: The enriched refined edible oils and wheat flour must meet the quality and hygiene conditions defined by international standards for fortification of food, in particular the Codex Alimentarius standards or- the national standards.
4. Provides definitions that includes terms that are specific to fortification (e.g. fortified food, premix, fortificant, food vehicle)	(0) Does not state	
5. Provides repeals (if there is at least one prior document about fortification)	(N/A) No prior documents about fortification	
6. Provides effective date or gives grace period for when fortification is to begin (e.g. effective 6 months from signing)	(2) States effective date or grace period for when fortification is to begin (e.g. effective 6 months from signing)	Article 5: Economic operators have a period of six (6) months from the coming into effect of the decree referred to in Article 3 to comply with the provisions of this decree.
**MICRONUTRIENTS/PREMIX**		
7. States nutrients required	(2) States nutrients	Article 2: The micro-nutrients that must be used to enrich refined edible oils and flour from grinding wheat, manufactured, packaged, or imported in Togo are:<br>vitamin A for the refined edible oils;<br>- and iron-folic acid-zinc compound for the flour.
8. States fortificants (chemical compounds) to be used (including fortificants that are allowable as options)	(2) States fortificants for at least one nutrient	Article 3: The outfits of the compounds to be used in the enrichment of wheat flour produced or imported Togolese territory, before its release for home use are:<br>60g of iron as ferrous fumarate per ton of flour;<br>2.6 g folic acid (vitamin B9) per ton of flour;<br>55 g of zinc as zinc oxide per ton of flour.
9. States fortification levels	(1) States one number only	Article 3: The outfits of the compounds to be used in the enrichment of wheat flour produced or imported Togolese territory, before its release for home use are:<br>60g of iron as ferrous fumarate per ton of flour;<br>2.6 g folic acid (vitamin B9) per ton of flour;<br>55 g of zinc as zinc oxide per ton of flour.
10. States consideration of bioavailability/biological activity of fortificants	(0) Does not state any consideration	
11. States consideration of nutrient stability	(0) Does not state any consideration	
**COSTING**		
12. States that the cost of fortification is regulated through cost sharing schemes (between government, industry, consumers) or tax measures [to assist industry]	(2) States consideration of either cost regulation method	2. Build and maintain the political commitment of decision-makers, manufacturers and development partners, so as to ensure financial support and of equipment, in order to guarantee sustainability of the national strategy for food fortification.<br>3. Develop action plans and mechanisms for mobilising the funds required in such implementation.
13. States consideration of the financial responsibility of monitoring and enforcing fortification (schedule of fees, budget) [on the government side]	(0) Does not state any consideration	
**LABELING**		
14. Includes some sort of statement/label/logo that makes it clear that the product is fortified	(2) Includes a statement, label, or logo	Article 5: The labeling of these products must be in accordance with the Guidelines on Nutrition Labelling of the Codex Alimentarius (CAC / GL2--1985, Rev 2012) and provide at least the following information:<br>-The oil fortified with vitamin A mention for the oil and flour with iron, zinc, and folic acid to flour;<br>-the name or business name and address of the manufacturer;<br>-the regional logotype "ENRICHED"<br>Article 6: The affixing of the logo representing fortified foods is mandatory and subordnonnée compliance with the provisions defined in Articles 2 and 3 above.
15. Provides guidance on health claims that can be made for this product (specific to micronutrients added through fortification)	(0) Does not provide	
**INTERNAL MONITORING (conducted by industry)**		
16. If samples are to be taken, describes the sampling process: for example, number of samples, amount, frequency, individual vs. composite, where samples are taken in the process, and percent considered passing	(N/A) Does not describe the sampling process	
17. States that industry is required to follow quality assurance/quality control in regards to fortification	(0) Does not state requirement	

18. States applicability of using qualitative testing to determine the presence or absence of a vitamin or mineral (spot tests, iChecks)	(0) Does not state	
**EXTERNAL MONITORING (conducted by government)**		
19.States requirement for external monitoring at the production site to assure compliance with standards and regulations	(2) States requirement for external monitoring or the need for audits/inspections	Article 7: unrefined edible oils and wheat flour are subject to control in force in Togo in matters of health safety of food. These controls concern in particular the levels of vitamin A in iron, zinc and folic acid. Article 8: The results of the checks referred to in Article 7 give rise to the issuance of a compliance certificate required for obtaining a marketing authorization.
20. Describes protocols and systems for regulatory monitoring	(1) Does not explicitly describe, but references protocols and systems for regulatory monitoring	Article 10: The quality controls include wheat flour and enriched refined edible oil, packaging and labeling must be performed across the country at all stages, from production or import up has consumption.
21. If there are two or more government agencies involved in external monitoring, clarifies the roles and responsibilities between different government agencies in external monitoring	(0) Clarifies roles and responsibilities for no agencies	Article 11: The officers of ministry of health, quality control and standards services of ministries in charge of trade, industry, agriculture, livestock and fisheries and customs services shall carry out quality control of edible oil and refined flour. -->No roles clarified, but multiple agencies involved.
22. Allows for monitoring to be conducted often enough that problems can be identified and addressed on a timely basis; specifies a timeline for inspections (i.e. once every 6 months, increasing to once every 2 months if a discrepancy is found)	(0) Does not state	
23. If samples are to be taken, describes the sampling process: for example, number of samples, amount, frequency, individual vs. composite, where samples are taken in the process, and percent considered passing	(N/A) Does not describe the sampling process	
24. States applicability of using qualitative testing to determine the presence or absence of a vitamin or mineral (spot tests, iChecks)	(0) Does not state	
25. States registration is required in order to use a logo/be licensed to produce fortified foods	(2) Describes some type of registration or licensing	Article 8: The results of the checks referred to in Article 7 give rise to the issuance of a compliance certificate required for obtaining a marketing authorization.<br>The certificate of conformity issued by the Ministry of Health after verification of the analytical report, is valid for one (1) year.
**COMMERCIAL MONITORING (conducted by government)**		
26. Provides justification for commercial monitoring at retail stores	(0) Does not provide justification for commercial monitoring	Article 10: The quality controls include wheat flour and enriched refined edible oil, packaging and labeling must be performed across the country at all stages, from production or import up has consumption.
27. Describes protocols and systems for commercial monitoring	(0) Does not describe	
28. If there are two or more government agencies involved in commercial monitoring, clarifies the roles and responsibilities between different government agencies in commercial monitoring	(0) Clarifies roles and responsibilities for no agencies	
29. Allows for monitoring to be conducted often enough that problems at the production site or import companies can be identified and addressed on a timely basis; specifies a timeline for inspections (i.e. once every 6 months) or works with production companies to correct noncompliance	(N/A) No commercial monitoring occurs	
30. If samples are to be taken, describes the sampling process: for example, number of samples, amount, frequency, individual vs. composite, where samples are taken in the process, and percent considered passing	(N/A) Does not describe the sampling process	
**IMPORT MONITORING (conducted by government)**		
31. Provides justification for import monitoring at points of entry	(0) Does not provide justification for import monitoring	Article 10: The quality controls include wheat flour and enriched refined edible oil, packaging and labeling must be performed across the country at all stages, from production or import up has consumption.
32. Describes protocols and systems for import monitoring	(0) Does not state	
33. If there are two or more government agencies involved in import monitoring, clarifies the roles and responsibilities between different government agencies in import monitoring	(0) Clarifies roles and responsibilities for no agencies	Article 11: The officers of ministry of health, quality control and standards services of ministries in charge of trade, industry, agriculture, livestock and fisheries and customs services shall carry out quality control of edible oil and refined flour. -->No roles clarified, but multiple agencies involved.
34. If samples are to be taken, describes the sampling process: for example, number of samples, amount, frequency, individual vs. composite, where samples are taken in the process, and percent considered passing	(N/A) Does not describe the sampling process	
**ENFORCEMENT/PENALTIES**		

35. Indicates roles and responsibilities in enforcing the legislation	(2) States the role and responsibilities of government in enforcement	When the product is considered to be sub-standard the administration immediately starts impounding the batches. A period of one month is granted to the producer, importer, or distributor to enrich the products on site under the supervision of the inspection services. On expiry of this period the impounded product shall be quite simply destroyed. Article 13: Any person infringing the provisions of this decree shall be punished pursuant to current legislation.
36. States incentives to start fortification	(0) Does not state	
37. States incentives to continue fortification, including ensuring compliance	(0) Does not state	
38. States penalties to compel compliance	(2) States any penalties	Article 4: The import, manufacture, packaging, sale, marketing, possession for sale, distribution free of charge, and consumption of refined edible oils unenriched with vitamins A and of wheat flour for human consumption unenriched with iron, folic acid and zinc are prohibited throughout the territory of Togo. Article 6: Every infringement of the provisions of this decree is punished pursuant to current legislation. Article 13: Any person infringing the provisions of this decree shall be punished pursuant to current legislation.
39. Penalties are objectively defined (e.g. first penalty=$100, second penalty=$300)	(0) Penalties are not objectively laid out	
40. States that enforcement is required to include feedback and support to improve performance and correct noncompliance	(0) Does not require	A period of one month is granted to the producer, importer, or distributor to enrich the products on site under the supervision of the inspection services. On expiry of this period the impounded product shall be quite simply destroyed.
**LABORATORY**		
41. References required analytical assays for nutrients (e.g. Liquid chromatography-mass spectrometry for folic acid, atomic absorption for iron and zinc.)	(0) Does not state requirements	
42. States recognition that laboratory results are subject to several sources of variation and do not provide conclusive evidence of compliance or noncompliance	(0) Does not state recognition	
43. Focuses on the quantitative analysis of "marker" micronutrients such as iron	(0) Does not state	
**REPORTING**		
44. States how government monitoring results are shared with stakeholders	(0) Does not state how results are shared	

Turkmenistan Wheat

Element	Scoring (use drop-down menus)	Comments (open text)
**GENERAL**		
1. States that legislation applies to at least one food vehicle fit for human consumption (types/grades to be fortified)	(2) States at least one type fit for human consumption	Decree Para 1
2. States the public health objective; purpose and scope of legislation	(2) States the public health objective or general purpose of legislation	Decree intro
international norms and recommendations, particularly for items that may not be covered in the country's documents	(2) States the documents referenced	results of studies confirming their lack in the nutrition of certain groups...
4. Provides definitions that includes terms that are specific to fortification (e.g. fortified food, premix, fortificant, food vehicle)	(2) States at least one term related to fortification	Doc 1 (1) Micronutrients, Nutritional Value; Doc 1 (20.1) Enriched food
5. Provides repeals (if there is at least one prior document about fortification)	(2) States repeals	Doc 1 (2.2) International treaty prevails
6. Provides effective date or gives grace period for when fortification is to begin (e.g. effective 6 months from signing)	(2) States effective date or grace period for when fortification is to begin (e.g. effective 6 months from signing)	Decree para 1
**MICRONUTRIENTS/PREMIX**		
7. States nutrients required	(2) States nutrients	Decree intro
8. States fortificants (chemical compounds) to be used (including fortificants that are allowable as options)	(0) Does not state	
9. States fortification levels	(0) Does not state	
10. States consideration of bioavailability/biological activity of fortificants	(0) Does not state any consideration	
11. States consideration of nutrient stability	(0) Does not state any consideration	
**COSTING**		
12. States that the cost of fortification is regulated through cost sharing schemes (between government, industry, consumers) or tax measures [to assist industry]	(0) Does not state any consideration	
13. States consideration of the financial responsibility of monitoring and enforcing fortification (schedule of fees, budget) [on the government side]	(0) Does not state any consideration	
**LABELING**		
14. Includes some sort of statement/label/logo that makes it clear that the product is fortified	(2) Includes a statement, label, or logo	
15. Provides guidance on health claims that can be made for this product (specific to micronutrients added through fortification)	(0) Does not provide	
**INTERNAL MONITORING (conducted by industry)**		
16. If samples are to be taken, describes the sampling process: for example, number of samples, amount, frequency, individual vs. composite, where samples are taken in the process, and percent considered passing	(N/A) Does not describe the sampling process	
17. States that industry is required to follow quality assurance/quality control in regards to fortification	(2) States requirement of QA/QC for fortification	Doc 1 (12.1)
18. States applicability of using qualitative testing to determine the presence or absence of a vitamin or mineral (spot tests, iChecks)	(0) Does not state	
**EXTERNAL MONITORING (conducted by government)**		
19.States requirement for external monitoring at the production site to assure compliance with standards and regulations	(2) States requirement for external monitoring or the need for audits/inspections	Decree Para 5, 6; Doc 1 (4.1) prevention of production...
20. Describes protocols and systems for regulatory monitoring	(0) Does not describe	
21. If there are two or more government agencies involved in external monitoring, clarifies the roles and responsibilities between different government agencies in external monitoring	(2) Clarifies roles and responsibilities for more than one agency	Decree Para 3-6; Doc 1 (6-8)
22. Allows for monitoring to be conducted often enough that problems can be identified and addressed on a timely basis; specifies a timeline for inspections (i.e. once every 6 months, increasing to once every 2 months if a discrepancy is found)	(0) Does not state	
23. If samples are to be taken, describes the sampling process: for example, number of samples, amount, frequency, individual vs. composite, where samples are taken in the process, and percent considered passing	(N/A) Does not describe the sampling process	
24. States applicability of using qualitative testing to determine the presence or absence of a vitamin or mineral (spot tests, iChecks)	(0) Does not state	
25. States registration is required in order to use a logo/be licensed to produce fortified foods	(2) Describes some type of registration or licensing	Doc 1 (9)
**COMMERCIAL MONITORING (conducted by government)**		
26. Provides justification for commercial monitoring at retail stores	(2) Provides justification for commercial monitoring	T1 - 28/29 - examination of foods and materials, recognition of substandard/dangerous foods; T2 - Article 9 - state control of monitoring food supply, food quality for compliance

27. Describes protocols and systems for commercial monitoring	(1) Does not explicitly describe, but references protocols and systems for regulatory monitoring	T1 - 28/29 - examination
28. If there are two or more government agencies involved in commercial monitoring, clarifies the roles and responsibilities between different government agencies in commercial monitoring	(N/A) Only one government agency involved	T2 State Control of Cabinet of Ministers
29. Allows for monitoring to be conducted often enough that problems at the production site or import companies can be identified and addressed on a timely basis; specifies a timeline for inspections (i.e. once every 6 months) or works with production companies to correct noncompliance	(0) Does not state	
30. If samples are to be taken, describes the sampling process: for example, number of samples, amount, frequency, individual vs. composite, where samples are taken in the process, and percent considered passing	(N/A) Does not describe the sampling process	
**IMPORT MONITORING (conducted by government)**		
31. Provides justification for import monitoring at points of entry	(2) Provides justification for import monitoring	Doc 1 (4.1) - prevention of importation…
32. Describes protocols and systems for import monitoring	(1) Does not explicitly state, but references protocols and systems for import monitoring	T1 - 27.5.2 - requiring certificates of conformity
33. If there are two or more government agencies involved in import monitoring, clarifies the roles and responsibilities between different government agencies in import monitoring	(2) Clarifies roles and responsibilities for more than one agency	Decree Para 3-6; Doc 1 (6-8)
34. If samples are to be taken, describes the sampling process: for example, number of samples, amount, frequency, individual vs. composite, where samples are taken in the process, and percent considered passing	(N/A) Does not describe the sampling process	
**ENFORCEMENT/PENALTIES**		
35. Indicates roles and responsibilities in enforcing the legislation	(2) States the role and responsibilities of government in enforcement	
36. States incentives to start fortification	(0) Does not state	
37. States incentives to continue fortification, including ensuring compliance	(0) Does not state	
38. States penalties to compel compliance	(2) States any penalties	Doc 1 (19.11) - suspend production, organize product recall
39. Penalties are objectively defined (e.g. first penalty=$100, second penalty=$300)	(0) Penalties are not objectively laid out	
40. States that enforcement is required to include feedback and support to improve performance and correct noncompliance	(0) Does not require	
**LABORATORY**		
41. References required analytical assays for nutrients (e.g. Liquid chromatography-mass spectrometry for folic acid, atomic absorption for iron and zinc.)	(0) Does not state requirements	
42. States recognition that laboratory results are subject to several sources of variation and do not provide conclusive evidence of compliance or noncompliance	(0) Does not state recognition	
43. Focuses on the quantitative analysis of "marker" micronutrients such as iron	(0) Does not state	
**REPORTING**		
44. States how government monitoring results are shared with stakeholders	(0) Does not state how results are shared	

Uganda Wheat

Element	Scoring (use drop-down menus)	Comments (open text)
**GENERAL**		
1. States that legislation applies to at least one food vehicle fit for human consumption (types/grades to be fortified)	(2) States at least one type fit for human consumption	ECSA Standard - Part 1
2. States the public health objective; purpose and scope of legislation	(2) States the public health objective or general purpose of legislation	ECSA Standard - Intro, para 2/3
3. References latest available science or accepted international norms and recommendations, particularly for items that may not be covered in the country's documents	(2) States the documents referenced	ECSA Standard - Part 5.2 - shall be stable compounds conforming to specifications in...
4. Provides definitions that includes terms that are specific to fortification (e.g. fortified food, premix, fortificant, food vehicle)	(2) States at least one term related to fortification	ECSA Standard - Part 3
5. Provides repeals (if there is at least one prior document about fortification)	(2) States repeals	ECSA Standard - Foreword - Repeal existing national standards
6. Provides effective date or gives grace period for when fortification is to begin (e.g. effective 6 months from signing)	(0) Does not state	
**MICRONUTRIENTS/PREMIX**		
7. States nutrients required	(2) States nutrients	ECSA Standard - Table 2
8. States fortificants (chemical compounds) to be used (including fortificants that are allowable as options)	(2) States fortificants for at least one nutrient	ECSA Standard - Table 2
9. States fortification levels	(2) States a range or number with +/-	ECSA Standard - Table 2
10. States consideration of bioavailability/biological activity of fortificants	(0) Does not state any consideration	
11. States consideration of nutrient stability	(2) States consideration of nutrient stability	ECSA Standard - Part 5.4
**COSTING**		
12. States that the cost of fortification is regulated through cost sharing schemes (between government, industry, consumers) or tax measures [to assist industry]	(0) Does not state any consideration	
13. States consideration of the financial responsibility of monitoring and enforcing fortification (schedule of fees, budget) [on the government side]	(2) Shows consideration that monitoring costs money	2005 Regs - Second Schedule f - manufacturers/importers are to bear the cost of analysis; Manual - Planning/Budget/Schedule section for each type of monitoring gives consideration for budgeting for inspections
**LABELING**		
14. Includes some sort of statement/label/logo that makes it clear that the product is fortified	(2) Includes a statement, label, or logo	ECSA Standard - Part 10.1
15. Provides guidance on health claims that can be made for this product (specific to micronutrients added through fortification)	(2) Provides guidance on health claims specific to micronutrients added through fortification	ECSA Standard - Part 10.3
**INTERNAL MONITORING (conducted by industry)**		
16. If samples are to be taken, describes the sampling process: for example, number of samples, amount, frequency, individual vs. composite, where samples are taken in the process, and percent considered passing	(1) States that samples should be taken (generally)	ECSA Standard - Part 11 - Refers to ISO 13690
17. States that industry is required to follow quality assurance/quality control in regards to fortification	(2) States requirement of QA/QC for fortification	ECSA Standard - As per EAS 39 - Code of Practice for Hygiene in Food Industry; 2005 Regs - 9(4) - manufacturer/importer shall establish QA system
18. States applicability of using qualitative testing to determine the presence or absence of a vitamin or mineral (spot tests, iChecks)	(2) States applicability of spot test to determine presence/absence of vitamin or mineral specific to internal monitoring	2005 Regs - First Schedule d(iii) - regular spot checks to ensure product has been dosed correctly
**EXTERNAL MONITORING (conducted by government)**		
19.States requirement for external monitoring at the production site to assure compliance with standards and regulations	(2) States requirement for external monitoring or the need for audits/inspections	Manual - Scope
20. Describes protocols and systems for regulatory monitoring	(2) Includes checklists or provides detailed description of regulatory monitoring procedures	Manual - very clear throughout
21. If there are two or more government agencies involved in external monitoring, clarifies the roles and responsibilities between different government agencies in external monitoring	(2) Clarifies roles and responsibilities for more than one agency	2005 Regs - Ministry of Health and NDA, Section 9; Manual - UNBS
22. Allows for monitoring to be conducted often enough that problems can be identified and addressed on a timely basis; specifies a timeline for inspections (i.e. once every 6 months, increasing to once every 2 months if a discrepancy is found)	(2) Describes frequency and how it is responsive to the needs of industry or the stage of fortification implementation	Manual - page 4/5 - 2x/yr, more frequent with identified issues
23. If samples are to be taken, describes the sampling process: for example, number of samples, amount, frequency, individual vs. composite, where samples are taken in the process, and percent considered passing	(2) States that samples should be taken as part of external monitoring	Manual - page 8 for maize, 11 for wheat
24. States applicability of using qualitative testing to determine the presence or absence of a vitamin or mineral (spot tests, iChecks)	(2) States applicability of spot test to determine presence/absence of vitamin or mineral specific to external monitoring	Manual - page 8 for maize, 11 for wheat - estimated iron level from daily composite samples
25. States registration is required in order to use a logo/be licensed to produce fortified foods	(0) Does not state that registration or licensing is required	

**COMMERCIAL MONITORING (conducted by government)**		
26. Provides justification for commercial monitoring at retail stores	(2) Provides justification for commercial monitoring	Manual - Scope
27. Describes protocols and systems for commercial monitoring	(2) Includes checklists or provides detailed description of regulatory monitoring procedures	Manual - Part III
28. If there are two or more government agencies involved in commercial monitoring, clarifies the roles and responsibilities between different government agencies in commercial monitoring	(2) Clarifies roles and responsibilities for more than one agency	2005 Regs - Ministry of Health and NDA, Section 9; Manual - UNBS
29. Allows for monitoring to be conducted often enough that problems at the production site or import companies can be identified and addressed on a timely basis; specifies a timeline for inspections (i.e. once every 6 months) or works with production companies to correct noncompliance	(2) Describes frequency and how it is responsive to the needs of industry or the stage of fortification implementation	Manual - Page 34, 1.3.1 - yearly visit to each region. Frequency and intensity of sampling depending on population, amount of food sold, risk factors - close to borders with non-fortifying countries
30. If samples are to be taken, describes the sampling process: for example, number of samples, amount, frequency, individual vs. composite, where samples are taken in the process, and percent considered passing	(2) States that samples should be taken as part of commercial monitoring	ECSA Standard - Part 11 - Refers to ISO 13690 - Manual, page 36 - 2.3 procedure for inspections, sampling, and analysis
**IMPORT MONITORING (conducted by government)**		
31. Provides justification for import monitoring at points of entry	(2) Provides justification for import monitoring	Manual - Scope
32. Describes protocols and systems for import monitoring	(2) Includes checklists or detailed description of import monitoring procedures	2005 Regs - Second Schedule - responsibility of importer - Manual, section II
33. If there are two or more government agencies involved in import monitoring, clarifies the roles and responsibilities between different government agencies in import monitoring	(2) Clarifies roles and responsibilities for more than one agency	2005 Regs - Ministry of Health and NDA, Section 9; Manual - UNBS
34. If samples are to be taken, describes the sampling process: for example, number of samples, amount, frequency, individual vs. composite, where samples are taken in the process, and percent considered passing	(2) States that samples should be taken as part of import monitoring	2005 Regs - Second Schedule d - samples every 6 months or when required - Manual - page 23 - 1.3.4 preparation of monthly composite samples; Page 28 - 3.4 qualitative testing for iron
**ENFORCEMENT/PENALTIES**		
35. Indicates roles and responsibilities in enforcing the legislation	(2) States the role and responsibilities of government in enforcement	2005 Reg - 4(2) - Minister monitors the implementation and enforcement
36. States incentives to start fortification	(0) Does not state	
37. States incentives to continue fortification, including ensuring compliance	(0) Does not state	
38. States penalties to compel compliance	(2) States any penalties	2005 Regs - 9(3) - impound, stop manufacture, etc.; 11 - imprisonment, destroy products
39. Penalties are objectively defined (e.g. first penalty=$100, second penalty=$300)	(0) Penalties are not objectively laid out	
40. States that enforcement is required to include feedback and support to improve performance and correct noncompliance	(2) Requires any feedback/support to improve performance	Manual - page 5 - providing technical support to factories to correct iddues
**LABORATORY**		
41. References required analytical assays for nutrients (e.g. Liquid chromatography-mass spectrometry for folic acid, atomic absorption for iron and zinc.)	(2) References required assays	ECSA Standard - Part 12 - ECSA or other internationally recognized test methods
42. States recognition that laboratory results are subject to several sources of variation and do not provide conclusive evidence of compliance or noncompliance	(2) States recognition that lab results are subject to variation	Manual - Page 9 (section b) for Maize, page 12 (section b) for wheat
43. Focuses on the quantitative analysis of "marker" micronutrients such as iron	(2) Focuses on quantitative analysis of marker micronutrient such as iron	2005 Regs - First Schedule d(iii) - by determining one of the components of a fortificant...; Manual - Page 22, section 1.3.2 - Confirming the presence of indicator micronutrients - Vitamin A and Iron mentioned in Manual, page 8 and 11 for industry monitoring
**REPORTING**		
44. States how government monitoring results are shared with stakeholders	(2) States how results are shared with stakeholders	Manual page 3 - results should be consolidated minimum 2x/yr, annual report with data presented graphically…

United Kingdom Wheat

Element	Scoring (use drop-down menus)	Comments (open text)
**GENERAL**		
1. States that legislation applies to at least one food vehicle fit for human consumption (types/grades to be fortified)	(2) States at least one type fit for human consumption	1998 Reg - Lists exemptions and types of flour/bread in Sections 3/4
2. States the public health objective; purpose and scope of legislation	(2) States the public health objective or general purpose of legislation	2013 Letter - states that regs still stand after review of health impacts
3. References latest available science or accepted international norms and recommendations, particularly for items that may not be covered in the country's documents	(2) States the documents referenced	Guidance Note - Page 8 - …in accordance with the European Council Regulations
4. Provides definitions that includes terms that are specific to fortification (e.g. fortified food, premix, fortificant, food vehicle)	(0) Does not state	
5. Provides repeals (if there is at least one prior document about fortification)	(2) States repeals	1998 Reg - Schedule 4 - Revocations
6. Provides effective date or gives grace period for when fortification is to begin (e.g. effective 6 months from signing)	(2) States effective date or grace period for when fortification is to begin (e.g. effective 6 months from signing)	1998 Reg - 19 Feb 98
**MICRONUTRIENTS/PREMIX**		
7. States nutrients required	(2) States nutrients	1998 Reg - Schedule 1
8. States fortificants (chemical compounds) to be used (including fortificants that are allowable as options)	(2) States fortificants for at least one nutrient	1998 Reg - Schedule 1
9. States fortification levels	(1) States one number only	1998 Reg - Schedule 1; Guidance Note - Page 10 - Calcium carbonate has a max/min
10. States consideration of bioavailability/biological activity of fortificants	(0) Does not state any consideration	
11. States consideration of nutrient stability	(0) Does not state any consideration	
**COSTING**		
12. States that the cost of fortification is regulated through cost sharing schemes (between government, industry, consumers) or tax measures [to assist industry]	(0) Does not state any consideration	
13. States consideration of the financial responsibility of monitoring and enforcing fortification (schedule of fees, budget) [on the government side]	(0) Does not state any consideration	
**LABELING**		
14. Includes some sort of statement/label/logo that makes it clear that the product is fortified	(0) Does not include statement, label, or logo	Guidance Note - Page 3 - Makes reference to Reg 18(1)(e) of food labelling regulations 1996, page 7 says that added nutrients don't have to be labelled by exemption. Nothing specific to say that the flour is "fortified"
15. Provides guidance on health claims that can be made for this product (specific to micronutrients added through fortification)	(2) Provides guidance on health claims specific to micronutrients added through fortification	Guidance Note - Page 3 - makes reference to Reg EC No 1924/2006 on nutrition and health claims made on food
**INTERNAL MONITORING (conducted by industry)**		
16. If samples are to be taken, describes the sampling process: for example, number of samples, amount, frequency, individual vs. composite, where samples are taken in the process, and percent considered passing	(N/A) Does not describe the sampling process	
17. States that industry is required to follow quality assurance/quality control in regards to fortification	(2) States requirement of QA/QC for fortification	Guidance Note - Page 3 - Makes reference to Food Safety Act
18. States applicability of using qualitative testing to determine the presence or absence of a vitamin or mineral (spot tests, iChecks)	(0) Does not state	
**EXTERNAL MONITORING (conducted by government)**		
19.States requirement for external monitoring at the production site to assure compliance with standards and regulations	(0) Does not state requirement	
20. Describes protocols and systems for regulatory monitoring	(0) Does not describe	
21. If there are two or more government agencies involved in external monitoring, clarifies the roles and responsibilities between different government agencies in external monitoring	(0) Clarifies roles and responsibilities for no agencies	
22. Allows for monitoring to be conducted often enough that problems can be identified and addressed on a timely basis; specifies a timeline for inspections (i.e. once every 6 months, increasing to once every 2 months if a discrepancy is found)	(0) Does not state	
23. If samples are to be taken, describes the sampling process: for example, number of samples, amount, frequency, individual vs. composite, where samples are taken in the process, and percent considered passing	(N/A) Does not describe the sampling process	
24. States applicability of using qualitative testing to determine the presence or absence of a vitamin or mineral (spot tests, iChecks)	(0) Does not state	
25. States registration is required in order to use a logo/be licensed to produce fortified foods	(0) Does not state that registration or licensing is required	

**COMMERCIAL MONITORING (conducted by government)**		
26. Provides justification for commercial monitoring at retail stores	(0) Does not provide justification for commercial monitoring	
27. Describes protocols and systems for commercial monitoring	(0) Does not describe	
28. If there are two or more government agencies involved in commercial monitoring, clarifies the roles and responsibilities between different government agencies in commercial monitoring	(0) Clarifies roles and responsibilities for no agencies	
29. Allows for monitoring to be conducted often enough that problems at the production site or import companies can be identified and addressed on a timely basis; specifies a timeline for inspections (i.e. once every 6 months) or works with production companies to correct noncompliance	(0) Does not state	
30. If samples are to be taken, describes the sampling process: for example, number of samples, amount, frequency, individual vs. composite, where samples are taken in the process, and percent considered passing	(N/A) Does not describe the sampling process	
**IMPORT MONITORING (conducted by government)**		
31. Provides justification for import monitoring at points of entry	(2) Provides justification for import monitoring	Guidance Note - Page 4/5 - import monitoring only when brought in from a third country (outside of EU jurisdiction)
32. Describes protocols and systems for import monitoring	(0) Does not state	
33. If there are two or more government agencies involved in import monitoring, clarifies the roles and responsibilities between different government agencies in import monitoring	(0) Clarifies roles and responsibilities for no agencies	
34. If samples are to be taken, describes the sampling process: for example, number of samples, amount, frequency, individual vs. composite, where samples are taken in the process, and percent considered passing	(N/A) Does not describe the sampling process	
**ENFORCEMENT/PENALTIES**		
35. Indicates roles and responsibilities in enforcing the legislation	(2) States the role and responsibilities of government in enforcement	1998 Regs - Part 8 - each food authority shall enforce and execute regs in its area
36. States incentives to start fortification	(0) Does not state	
37. States incentives to continue fortification, including ensuring compliance	(0) Does not state	
38. States penalties to compel compliance	(2) States any penalties	1998 Regs - Part 7 Offences and penalties
39. Penalties are objectively defined (e.g. first penalty=$100, second penalty=$300)	(0) Penalties are not objectively laid out	1998 Regs - Refers to fines not exceeding level 5 on the standard scale
40. States that enforcement is required to include feedback and support to improve performance and correct noncompliance	(0) Does not require	
**LABORATORY**		
41. References required analytical assays for nutrients (e.g. Liquid chromatography-mass spectrometry for folic acid, atomic absorption for iron and zinc.)	(2) References required assays	
42. States recognition that laboratory results are subject to several sources of variation and do not provide conclusive evidence of compliance or noncompliance	(0) Does not state recognition	
43. Focuses on the quantitative analysis of "marker" micronutrients such as iron	(0) Does not state	
**REPORTING**		
44. States how government monitoring results are shared with stakeholders	(0) Does not state how results are shared	

United States Wheat

Element	Scoring (use drop-down menus)	Comments (open text)
**GENERAL**		
1. States that legislation applies to at least one food vehicle fit for human consumption (types/grades to be fortified)	(2) States at least one type fit for human consumption	Also, in the October 14, 1993, issue of the Federal Register, FDA published a proposal entitled ''Food Standards: Amendment of the Standards of Identity for Enriched Grain Products to Require Addition of Folic Acid,'' (58 FR 53305) (hereinafter referred to as the standards of identity proposal) to amend the following standards of identity to require the addition of folic acid at a fortification level of 140 mg/100 g: enriched bread, rolls, and buns (§ 136.115 (21 CFR 136.115)); enriched flour (§ 137.165 (21 CFR 137.165)); enriched self-rising flour (§ 137.185 (21 CFR 137.185)); enriched corn grits (§ 137.235 (21 CFR 137.235)); enriched corn meals (§ 137.260 (21 CFR 137.260)); enriched farina (§ 137.305 (21 CFR 137.305)); enriched rice (§ 137.350 (21 CFR 137.350)); enriched macaroni products (§ 139.115 (21 CFR 139.115)); enriched nonfat milk macaroni products (§ 139.122 (21 CFR 139.122)); and
2. States the public health objective; purpose and scope of legislation	(2) States the public health objective or general purpose of legislation	This action is being taken to help women of childbearing age to reduce their risk of having a pregnancy affected with spina bifida or other neural tube defects (NTD's) and to comply with the recommendation of the U.S. Public Health Service (PHS) that they consume at least 0.4 mg (400 mg) of folic acid daily.
3. References latest available science or accepted international norms and recommendations, particularly for items that may not be covered in the country's documents	(2) States the documents referenced	The Food and Drug Administration (FDA) is amending the food additive regulations to provide for the safe use of folic acid in foods that are the subject of a standard of identity that requires the addition of folic acid; to provide for its addition to breakfast cereals on a per serving basis; to permit its use in infant formulas, medical foods, and foods for special dietary use; and to incorporate specifications for folic acid consistent with those in the Food Chemicals Codex.
4. Provides definitions that includes terms that are specific to fortification (e.g. fortified food, premix, fortificant, food vehicle)	(2) States at least one term related to fortification	The term "essential nutrient" under the fortification policy refers to the vitamins and minerals assigned Reference Daily Intakes (RDIs) listed in 21 CFR 101.9(c)(8)(iv), as well as protein and potassium that have daily reference values (DRVs) (21 CFR 101.9(c)(9) or 21 CFR 104.20(d)(3)).
5. Provides repeals (if there is at least one prior document about fortification)	(2) States repeals	In the Federal Register of October 14, 1993 (58 FR 53312), FDA proposed to amend the regulation that establishes safe conditions of food use for folic acid, § 172.345 (21 CFR 172.345)).
6. Provides effective date or gives grace period for when fortification is to begin (e.g. effective 6 months from signing)	(2) States effective date or grace period for when fortification is to begin (e.g. effective 6 months from signing)	EFFECTIVE DATE: January 1, 1998. Many comments expressed concern over the statement in the standards of identity proposal that the final rule would become effective 1 year after publication.
**MICRONUTRIENTS/PREMIX**		
7. States nutrients required	(2) States nutrients	(a) It contains in each pound 2.9 milligrams of thiamin, 1.8 milligrams of riboflavin, 24 milligrams of niacin, 0.7 milligrams of folic acid, and 20 milligrams of iron.
8. States fortificants (chemical compounds) to be used (including fortificants that are allowable as options)	(2) States fortificants for at least one nutrient	(a) It contains in each pound 2.9 milligrams of thiamin, 1.8 milligrams of riboflavin, 24 milligrams of niacin, 0.7 milligrams of folic acid, and 20 milligrams of iron. DOES NOT SPECIFY IRON TYPE

9. States fortification levels	(2) States a range or number with +/-	(a) It contains in each pound 2.9 milligrams of thiamin, 1.8 milligrams of riboflavin, 24 milligrams of niacin, 0.7 milligrams of folic acid, and 20 milligrams of iron. The agency is requiring that these products be fortified with folic acid at levels ranging from 0.43 milligrams (mg) to 1.4 mg per pound (mg/lb) or 95 micrograms (mg) to 309 mg/100 grams (g), of product. These values are based on a fortification level of 140 mg/100 g (0.635 mg/lb) of the cereal grain product
10. States consideration of bioavailability/biological activity of fortificants	(2) States some consideration of bioavailability (mentions these or related terms)	With respect to issues of bioavailability, FDA concluded that bioavailability cannot be meaningfully factored into fortification scenarios because issues of bioavailability are very complex, and no systematic data are available on many of the factors that affect bioavailability.
11. States consideration of nutrient stability	(2) States consideration of nutrient stability	(c) The requirement of paragraphs (a) and (b) of this section will be deemed to have been met if reasonable overages of the vitamins and minerals, within the limits of good manufacturing practice, are present to insure that the required levels of the vitamins and minerals are maintained throughout the expected shelf life of the food under customary conditions of distribution and storage. Review food additives and enrichment in storage to determine if allowed in standards (21 CFR 137). Calculate percentages or amounts in the finished product per CWT (hundred weight or 100 lbs.) of flour (remember to be consistent with the units utilized in the standards.). Enrichment held for a year or more, or held at abnormally high temperatures or humidity, may have deteriorated.
**COSTING**		
12. States that the cost of fortification is regulated through cost sharing schemes (between government, industry, consumers) or tax measures [to assist industry]	(2) States consideration of either cost regulation method	c. Reformulation costs. Reformulation costs associated with this option were estimated in the proposal to be $27 million for the first year. The cost of adding the required folic acid is approximately $4 million per year. The cost of testing was estimated to be about $2.5 million per year and the cost of the required label changes $20 million. FDA will use these costs for this final rule as no comments were received on this part of the analysis.
13. States consideration of the financial responsibility of monitoring and enforcing fortification (schedule of fees, budget) [on the government side]	(2) Shows consideration that monitoring costs money	c. Reformulation costs. Reformulation costs associated with this option were estimated in the proposal to be $27 million for the first year. The cost of adding the required folic acid is approximately $4 million per year. The cost of testing was estimated to be about $2.5 million per year and the cost of the required label changes $20 million. FDA will use these costs for this final rule as no comments were received on this part of the analysis.
**LABELING**		

14. Includes some sort of statement/label/logo that makes it clear that the product is fortified	(2) Includes a statement, label, or logo	FDA also notes that the regulations for nutrition labeling in § 101.9(g)(4)(i) require that added nutrients be present in the food at levels that are at least equal to the amount declared on the label. In addition, § 101.9(g)(6) provides for reasonable overages within the limits of CGMP. Thus, the manufacturer bears the responsibility of ensuring that not only will the requirements for added nutrients in the respective standards of identity be met, but also that the content of any added nutrient is accurately declared in nutrition labeling. Therefore, consumers should receive the stated quantity of each added nutrient whether the standard provides for the added nutrient in terms of a single level or a range.
15. Provides guidance on health claims that can be made for this product (specific to micronutrients added through fortification)	(2) Provides guidance on health claims specific to micronutrients added through fortification	but, if insufficient additional calcium is present to meet the 960 milligram level, no claim may be made on the label for calcium as a nutrient; (d) Model health claims. The following are examples of model health claims that may be used in food labeling to describe the relationship between folate and neural tube defects: (1) Examples 1 and 2. Model health claims appropriate for foods containing 100 percent or less of the DV for folate per serving or per unit (general population). The examples contain only the required elements: (i) Healthful diets with adequate folate may reduce a woman's risk of having a child with
**INTERNAL MONITORING (conducted by industry)**		
16. If samples are to be taken, describes the sampling process: for example, number of samples, amount, frequency, individual vs. composite, where samples are taken in the process, and percent considered passing	(N/A) Does not describe the sampling process	
17. States that industry is required to follow quality assurance/quality control in regards to fortification	(0) Does not state requirement	
18. States applicability of using qualitative testing to determine the presence or absence of a vitamin or mineral (spot tests, iChecks)	(0) Does not state	
**EXTERNAL MONITORING (conducted by government)**		
19.States requirement for external monitoring at the production site to assure compliance with standards and regulations	(0) Does not state requirement	
20. Describes protocols and systems for regulatory monitoring	(0) Does not describe	
21. If there are two or more government agencies involved in external monitoring, clarifies the roles and responsibilities between different government agencies in external monitoring	(0) Clarifies roles and responsibilities for no agencies	
22. Allows for monitoring to be conducted often enough that problems can be identified and addressed on a timely basis; specifies a timeline for inspections (i.e. once every 6 months, increasing to once every 2 months if a discrepancy is found)	(0) Does not state	
23. If samples are to be taken, describes the sampling process: for example, number of samples, amount, frequency, individual vs. composite, where samples are taken in the process, and percent considered passing	(1) States that samples should be taken (generally)	Vitamins and minerals - Samples will be collected on assignment basis or if inspectional conditions warrant. 1. Retail Packages 4.5kg (10 lbs or less) -Collect twelve intact packages in duplicate of the same code. 2. Bulk and Retail Packages 4.5kg (Larger than 10 lbs) - Collect 12 - 453 gm (1 lb) subs in duplicate from each of twelve different containers of the same code. Remove flour or other cereal product with a trier inserted the full length of the container. Prepare each sub in individual airtight containers and protect against sunlight by wrapping in opaque paper. Do not fumigate samples.
24. States applicability of using qualitative testing to determine the presence or absence of a vitamin or mineral (spot tests, iChecks)	(0) Does not state	

25. States registration is required in order to use a logo/be licensed to produce fortified foods	(0) Does not state that registration or licensing is required	
**COMMERCIAL MONITORING (conducted by government)**		
26. Provides justification for commercial monitoring at retail stores	(0) Does not provide justification for commercial monitoring	Under Bakery inspections: Determine method of adding enrichment to the product whether by use of enriched flour, enriched salt, or enrichment wafers. If enrichment analyses are made by the firm or contract laboratory, determine sample size, method of collection, and review the most recent assay results. Obtain formulations of standardized products and determine if the firm's manufacturing and enrichment practices results in products conforming with 21 CFR 136.115.
27. Describes protocols and systems for commercial monitoring	(0) Does not describe	
28. If there are two or more government agencies involved in commercial monitoring, clarifies the roles and responsibilities between different government agencies in commercial monitoring	(0) Clarifies roles and responsibilities for no agencies	
29. Allows for monitoring to be conducted often enough that problems at the production site or import companies can be identified and addressed on a timely basis; specifies a timeline for inspections (i.e. once every 6 months) or works with production companies to correct noncompliance	(N/A) No commercial monitoring occurs	
30. If samples are to be taken, describes the sampling process: for example, number of samples, amount, frequency, individual vs. composite, where samples are taken in the process, and percent considered passing	(1) States that samples should be taken (generally)	Enrichment, Minerals and Nutrition Labeling - Bread, rolls, baked or unbaked product: Compliance samples should consist of 12 subsamples of at least 1 lb. each (454 gm) collected in duplicate. Collect consumer size retail packages (2 - 1 lb. subs from each of 12 cases) from separate cases of the same code. Protect enrichment samples from sun by wrapping in opaque paper. Determine and record by sub the gross weight.
**IMPORT MONITORING (conducted by government)**		
31. Provides justification for import monitoring at points of entry	(0) Does not provide justification for import monitoring	Import document super general--not specific to flour
32. Describes protocols and systems for import monitoring	(2) Includes checklists or detailed description of import monitoring procedures	
33. If there are two or more government agencies involved in import monitoring, clarifies the roles and responsibilities between different government agencies in import monitoring	(2) Clarifies roles and responsibilities for more than one agency	The purpose of this chapter is to provide an overview of import procedures for articles subject to the laws and regulations enforced by the Food and Drug Administration (FDA). The chapter also includes an overview of laws and regulations enforced by U.S. Customs and Border Protection (CBP), as they relate to importation of articles regulated by FDA. Division of Authority section
34. If samples are to be taken, describes the sampling process: for example, number of samples, amount, frequency, individual vs. composite, where samples are taken in the process, and percent considered passing	(1) States that samples should be taken (generally)	additional documentation is made, FDA will take appropriate action. If FDA decides to collect a sample, it will provide the filer, importer, owner and/or consignee, where appropriate, with a Notice of Sampling and advise: (1) whether the entry is to be held intact for FDA examination or sampling; or, (2) specify only those items that need be held. Generally, when FDA wishes to sample, it will be acting upon its authority in the Customs regulations (19 CFR 151.4) to collect its own samples for examination. FDA may request an examination or sample of articles under its jurisdiction. If no examination or sample is requested, FDA will so notify CBP and the filer, who is responsible for notifying the importer or other designated parties. This is referred to as a "May Proceed Notice," and indicates that the shipment may proceed without further FDA examination. In the ACS/OASIS process this may occur as a result of the initial FDA/OASIS screening prior to the information being forwarded to the district office, or after the district performs an "On-Screen-Review" of the information provided. (Note: Should the article, at a later time, be found in violation of the law, FDA is not prevented from taking legal action (e.g., seizure, injunction) because it allowed admission of the article without examination at the time of importation.) If FDA requests an examination or sample, FDA will notify CBP and the broker or filer, importer, or other
**ENFORCEMENT/PENALTIES**		

35. Indicates roles and responsibilities in enforcing the legislation	(2) States the role and responsibilities of government in enforcement	The purpose of this chapter is to provide an overview of import procedures for articles subject to the laws and regulations enforced by the Food and Drug Administration (FDA). The chapter also includes an overview of laws and regulations enforced by U.S. Customs and Border Protection (CBP), as they relate to importation of articles regulated by FDA.
36. States incentives to start fortification	(0) Does not state	
37. States incentives to continue fortification, including ensuring compliance	(0) Does not state	
38. States penalties to compel compliance	(2) States any penalties	Consequently, FDA may issue a warning letter and take enforcement action if a marketed food bearing one or more of these nutrient content claims contains a nutrient addition that is inconsistent with the fortification policy as incorporated into the regulations noted above. Nevertheless, FDA strongly encourages you to follow these fortification guidelines regardless of whether any claims appear on the label or in labeling.
39. Penalties are objectively defined (e.g. first penalty=$100, second penalty=$300)	(0) Penalties are not objectively laid out	
40. States that enforcement is required to include feedback and support to improve performance and correct noncompliance	(0) Does not require	
**LABORATORY**		
41. References required analytical assays for nutrients (e.g. Liquid chromatography-mass spectrometry for folic acid, atomic absorption for iron and zinc.)	(2) References required assays	Does this count???<br>The quantitative<br>content of the following vitamins shall<br>be calculated in terms of the following<br>chemically identifiable reference<br>forms:<br>Vitamin<br>Reference form<br>Name Empirical formula<br>Molecular<br>weight<br>Thiamine ... Thiamine chloride<br>hydrochloride.<br>C12H17ClN4OS·HCl 337.28<br>Riboflavin .. Riboflavin .... C17H20N4O6 376.37<br>Niacin .... Niacin .... C6H5NO2 123.11 (c)<br>The method referred to in paragraph<br>(a) of this section is the method<br>prescribed in ‘‘Official Methods of<br>Analysis of the Association of Official<br>Analytical Chemists’’ (AOAC), 13th Ed.<br>(1980), section 8.002, ‘‘Reagent (Displacement<br>soln.),’’
42. States recognition that laboratory results are subject to several sources of variation and do not provide conclusive evidence of compliance or noncompliance	(0) Does not state recognition	
43. Focuses on the quantitative analysis of "marker" micronutrients such as iron	(0) Does not state	
**REPORTING**		
44. States how government monitoring results are shared with stakeholders	(0) Does not state how results are shared	

Uruguay Wheat

Element	Scoring (use drop-down menus)	Comments (open text)
**GENERAL**		
1. States that legislation applies to at least one food vehicle fit for human consumption (types/grades to be fortified)	(2) States at least one type fit for human consumption	(Ref 1) ARTICULO 1, ARTICULO 2, ARTICULO 3. States which flour types must be fortified, which are excluded, and that foods made with flour must use fortified flour. (Ref 3) Exonerates semolina from fortification.
2. States the public health objective; purpose and scope of legislation	(2) States the public health objective or general purpose of legislation	(Ref 1, page 1) "RESULTANDO", (Ref 2) Artículo 1o.
3. References latest available science or accepted international norms and recommendations, particularly for items that may not be covered in the country's documents	(0) Does not state	
4. Provides definitions that includes terms that are specific to fortification (e.g. fortified food, premix, fortificant, food vehicle)	(0) Does not state	
5. Provides repeals (if there is at least one prior document about fortification)	(2) States repeals	ARTICULO 18
6. Provides effective date or gives grace period for when fortification is to begin (e.g. effective 6 months from signing)	(2) States effective date or grace period for when fortification is to begin (e.g. effective 6 months from signing)	(Ref 1) ARTICULO 11, ARTICULO 15. (Ref 2) Artículo 9.
**MICRONUTRIENTS/PREMIX**		
7. States nutrients required	(2) States nutrients	ARTICULO 1
8. States fortificants (chemical compounds) to be used (including fortificants that are allowable as options)	(2) States fortificants for at least one nutrient	ARTICULO 1
9. States fortification levels	(1) States one number only	ARTICULO 1
10. States consideration of bioavailability/biological activity of fortificants	(2) States some consideration of bioavailability (mentions these or related terms)	ARTICULO 5
11. States consideration of nutrient stability	(2) States consideration of nutrient stability	ARTICULO 5
**COSTING**		
12. States that the cost of fortification is regulated through cost sharing schemes (between government, industry, consumers) or tax measures [to assist industry]	(0) Does not state any consideration	
13. States consideration of the financial responsibility of monitoring and enforcing fortification (schedule of fees, budget) [on the government side]	(0) Does not state any consideration	
**LABELING**		
14. Includes some sort of statement/label/logo that makes it clear that the product is fortified	(2) Includes a statement, label, or logo	ARTICULO 12, ARTICULO 13
15. Provides guidance on health claims that can be made for this product (specific to micronutrients added through fortification)	(0) Does not provide	
**INTERNAL MONITORING (conducted by industry)**		
16. If samples are to be taken, describes the sampling process: for example, number of samples, amount, frequency, individual vs. composite, where samples are taken in the process, and percent considered passing	(N/A) Does not describe the sampling process	
17. States that industry is required to follow quality assurance/quality control in regards to fortification	(2) States requirement of QA/QC for fortification	ARTICULO 7, ARTICULO 8
18. States applicability of using qualitative testing to determine the presence or absence of a vitamin or mineral (spot tests, iChecks)	(0) Does not state	
**EXTERNAL MONITORING (conducted by government)**		
19.States requirement for external monitoring at the production site to assure compliance with standards and regulations	(2) States requirement for external monitoring or the need for audits/inspections	ARTICULO 16
20. Describes protocols and systems for regulatory monitoring	(0) Does not describe	
21. If there are two or more government agencies involved in external monitoring, clarifies the roles and responsibilities between different government agencies in external monitoring	(0) Clarifies roles and responsibilities for no agencies	
22. Allows for monitoring to be conducted often enough that problems can be identified and addressed on a timely basis; specifies a timeline for inspections (i.e. once every 6 months, increasing to once every 2 months if a discrepancy is found)	(0) Does not state	
23. If samples are to be taken, describes the sampling process: for example, number of samples, amount, frequency, individual vs. composite, where samples are taken in the process, and percent considered passing	(2) States that samples should be taken as part of external monitoring	ARTICULO 16
24. States applicability of using qualitative testing to determine the presence or absence of a vitamin or mineral (spot tests, iChecks)	(0) Does not state	
25. States registration is required in order to use a logo/be licensed to produce fortified foods	(0) Does not state that registration or licensing is required	
**COMMERCIAL MONITORING (conducted by government)**		

26. Provides justification for commercial monitoring at retail stores	(0) Does not provide justification for commercial monitoring	
27. Describes protocols and systems for commercial monitoring	(0) Does not describe	
28. If there are two or more government agencies involved in commercial monitoring, clarifies the roles and responsibilities between different government agencies in commercial monitoring	(0) Clarifies roles and responsibilities for no agencies	
29. Allows for monitoring to be conducted often enough that problems at the production site or import companies can be identified and addressed on a timely basis; specifies a timeline for inspections (i.e. once every 6 months) or works with production companies to correct noncompliance	(N/A) No commercial monitoring occurs	
30. If samples are to be taken, describes the sampling process: for example, number of samples, amount, frequency, individual vs. composite, where samples are taken in the process, and percent considered passing	(N/A) Does not describe the sampling process	
**IMPORT MONITORING (conducted by government)**		
31. Provides justification for import monitoring at points of entry	(2) Provides justification for import monitoring	ARTICULO 16
32. Describes protocols and systems for import monitoring	(0) Does not state	
33. If there are two or more government agencies involved in import monitoring, clarifies the roles and responsibilities between different government agencies in import monitoring	(0) Clarifies roles and responsibilities for no agencies	
34. If samples are to be taken, describes the sampling process: for example, number of samples, amount, frequency, individual vs. composite, where samples are taken in the process, and percent considered passing	(2) States that samples should be taken as part of import monitoring	ARTICULO 16
**ENFORCEMENT/PENALTIES**		
35. Indicates roles and responsibilities in enforcing the legislation	(2) States the role and responsibilities of government in enforcement	ARTICULO 16
36. States incentives to start fortification	(0) Does not state	
37. States incentives to continue fortification, including ensuring compliance	(0) Does not state	
38. States penalties to compel compliance	(2) States any penalties	ARTICULO 17
39. Penalties are objectively defined (e.g. first penalty=$100, second penalty=$300)	(2) Penalties are objectively laid out in the document (e.g. first penalty=$100, second penalty=$300)	ARTICULO 17
40. States that enforcement is required to include feedback and support to improve performance and correct noncompliance	(0) Does not require	
**LABORATORY**		
41. References required analytical assays for nutrients (e.g. Liquid chromatography-mass spectrometry for folic acid, atomic absorption for iron and zinc.)	(0) Does not state requirements	
42. States recognition that laboratory results are subject to several sources of variation and do not provide conclusive evidence of compliance or noncompliance	(0) Does not state recognition	
43. Focuses on the quantitative analysis of "marker" micronutrients such as iron	(0) Does not state	
**REPORTING**		
44. States how government monitoring results are shared with stakeholders	(2) States how results are shared with stakeholders	Artículo 8

Uzbekistan Wheat

Element	Scoring (use drop-down menus)	Comments (open text)
**GENERAL**		
1. States that legislation applies to at least one food vehicle fit for human consumption (types/grades to be fortified)	(2) States at least one type fit for human consumption	PPM - 1 - 1st grade flour
2. States the public health objective; purpose and scope of legislation	(2) States the public health objective or general purpose of legislation	Resolution - Intro - to reduce iron-deficiency anemia, improve public health
3. References latest available science or accepted international norms and recommendations, particularly for items that may not be covered in the country's documents	(0) Does not state	
4. Provides definitions that includes terms that are specific to fortification (e.g. fortified food, premix, fortificant, food vehicle)	(0) Does not state	
5. Provides repeals (if there is at least one prior document about fortification)	(0) Does not state	
6. Provides effective date or gives grace period for when fortification is to begin (e.g. effective 6 months from signing)	(2) States effective date or grace period for when fortification is to begin (e.g. effective 6 months from signing)	
**MICRONUTRIENTS/PREMIX**		
7. States nutrients required	(2) States nutrients	
8. States fortificants (chemical compounds) to be used (including fortificants that are allowable as options)	(0) Does not state	
9. States fortification levels	(1) States one number only	
10. States consideration of bioavailability/biological activity of fortificants	(0) Does not state any consideration	
11. States consideration of nutrient stability	(0) Does not state any consideration	
**COSTING**		
12. States that the cost of fortification is regulated through cost sharing schemes (between government, industry, consumers) or tax measures [to assist industry]	(2) States consideration of either cost regulation method	Resolution - 1/2/3/7 - financing shared by the Government State Budget, GAIN, UNICEF over 5 years; provision of premix/equipment to industry; govt to revise wholesale prices to build in fortification expenses; premix/equipment exempted from customs duties
13. States consideration of the financial responsibility of monitoring and enforcing fortification (schedule of fees, budget) [on the government side]	(2) Shows consideration that monitoring costs money	
**LABELING**		
14. Includes some sort of statement/label/logo that makes it clear that the product is fortified	(0) Does not include statement, label, or logo	
15. Provides guidance on health claims that can be made for this product (specific to micronutrients added through fortification)	(0) Does not provide	
**INTERNAL MONITORING (conducted by industry)**		
16. If samples are to be taken, describes the sampling process: for example, number of samples, amount, frequency, individual vs. composite, where samples are taken in the process, and percent considered passing	(N/A) Does not describe the sampling process	
17. States that industry is required to follow quality assurance/quality control in regards to fortification	(2) States requirement of QA/QC for fortification	Bylaw - page 11, #4 - To lay personal responsibility for the quality of the produced and sold foodstuff to the management of companies
18. States applicability of using qualitative testing to determine the presence or absence of a vitamin or mineral (spot tests, iChecks)	(0) Does not state	
**EXTERNAL MONITORING (conducted by government)**		
19.States requirement for external monitoring at the production site to assure compliance with standards and regulations	(0) Does not state requirement	Bylaw - page 12, MoH should carry out state sanitary control - we don't think this includes fortification monitoring.
20. Describes protocols and systems for regulatory monitoring	(0) Does not describe	
21. If there are two or more government agencies involved in external monitoring, clarifies the roles and responsibilities between different government agencies in external monitoring	(2) Clarifies roles and responsibilities for more than one agency	Resolution - 4/5 - JSC and Uzstandard to finance, establish standard; MoH carries out M&E, project impact, social marketing with Uzteleradio Company
22. Allows for monitoring to be conducted often enough that problems can be identified and addressed on a timely basis; specifies a timeline for inspections (i.e. once every 6 months, increasing to once every 2 months if a discrepancy is found)	(0) Does not state	
23. If samples are to be taken, describes the sampling process: for example, number of samples, amount, frequency, individual vs. composite, where samples are taken in the process, and percent considered passing	(N/A) Does not describe the sampling process	
24. States applicability of using qualitative testing to determine the presence or absence of a vitamin or mineral (spot tests, iChecks)	(0) Does not state	
25. States registration is required in order to use a logo/be licensed to produce fortified foods	(0) Does not state that registration or licensing is required	

**COMMERCIAL MONITORING (conducted by government)**		
26. Provides justification for commercial monitoring at retail stores	(0) Does not provide justification for commercial monitoring	
27. Describes protocols and systems for commercial monitoring	(0) Does not describe	
28. If there are two or more government agencies involved in commercial monitoring, clarifies the roles and responsibilities between different government agencies in commercial monitoring	(0) Clarifies roles and responsibilities for no agencies	
29. Allows for monitoring to be conducted often enough that problems at the production site or import companies can be identified and addressed on a timely basis; specifies a timeline for inspections (i.e. once every 6 months) or works with production companies to correct noncompliance	(0) Does not state	
30. If samples are to be taken, describes the sampling process: for example, number of samples, amount, frequency, individual vs. composite, where samples are taken in the process, and percent considered passing	(N/A) Does not describe the sampling process	
**IMPORT MONITORING (conducted by government)**		
31. Provides justification for import monitoring at points of entry	(0) Does not provide justification for import monitoring	
32. Describes protocols and systems for import monitoring	(0) Does not state	
33. If there are two or more government agencies involved in import monitoring, clarifies the roles and responsibilities between different government agencies in import monitoring	(0) Clarifies roles and responsibilities for no agencies	
34. If samples are to be taken, describes the sampling process: for example, number of samples, amount, frequency, individual vs. composite, where samples are taken in the process, and percent considered passing	(N/A) Does not describe the sampling process	
**ENFORCEMENT/PENALTIES**		
35. Indicates roles and responsibilities in enforcing the legislation	(0) Does not state	
36. States incentives to start fortification	(2) States any incentives to encourage fortification initiation (e.g. tax incentives for new equipment or premix)	See #12
37. States incentives to continue fortification, including ensuring compliance	(2) States any incentives to encourage the continuation of fortification (e.g. transport priority, favorable tax or tariff treatment, or patent rights)	See #12
38. States penalties to compel compliance	(0) Does not state	
39. Penalties are objectively defined (e.g. first penalty=$100, second penalty=$300)	(N/A) No penalties are stated (Answered (0) to previous question)	
40. States that enforcement is required to include feedback and support to improve performance and correct noncompliance	(0) Does not require	
**LABORATORY**		
41. References required analytical assays for nutrients (e.g. Liquid chromatography-mass spectrometry for folic acid, atomic absorption for iron and zinc.)	(0) Does not state requirements	
42. States recognition that laboratory results are subject to several sources of variation and do not provide conclusive evidence of compliance or noncompliance	(0) Does not state recognition	
43. Focuses on the quantitative analysis of "marker" micronutrients such as iron	(0) Does not state	
**REPORTING**		
44. States how government monitoring results are shared with stakeholders	(0) Does not state how results are shared	

Venezuela Wheat

Element	Scoring (use drop-down menus)	Comments (open text)
**GENERAL**		
1. States that legislation applies to at least one food vehicle fit for human consumption (types/grades to be fortified)	(2) States at least one type fit for human consumption	1 OBJETO
2. States the public health objective; purpose and scope of legislation	(0) Does not state	
3. References latest available science or accepted international norms and recommendations, particularly for items that may not be covered in the country's documents	(0) Does not state	
4. Provides definitions that includes terms that are specific to fortification (e.g. fortified food, premix, fortificant, food vehicle)	(2) States at least one term related to fortification	3 DEFINICIONES
5. Provides repeals (if there is at least one prior document about fortification)	(0) Does not state	
6. Provides effective date or gives grace period for when fortification is to begin (e.g. effective 6 months from signing)	(0) Does not state	
**MICRONUTRIENTS/PREMIX**		
7. States nutrients required	(2) States nutrients	Tabla 1
8. States fortificants (chemical compounds) to be used (including fortificants that are allowable as options)	(2) States fortificants for at least one nutrient	Tabla 1
9. States fortification levels	(2) States a range or number with +/-	Tabla 1
10. States consideration of bioavailability/biological activity of fortificants	(2) States some consideration of bioavailability (mentions these or related terms)	Tabla 1
11. States consideration of nutrient stability	(0) Does not state any consideration	
**COSTING**		
12. States that the cost of fortification is regulated through cost sharing schemes (between government, industry, consumers) or tax measures [to assist industry]	(0) Does not state any consideration	
13. States consideration of the financial responsibility of monitoring and enforcing fortification (schedule of fees, budget) [on the government side]	(0) Does not state any consideration	
**LABELING**		
14. Includes some sort of statement/label/logo that makes it clear that the product is fortified	(2) Includes a statement, label, or logo	9.2.1.1
15. Provides guidance on health claims that can be made for this product (specific to micronutrients added through fortification)	(0) Does not provide	
**INTERNAL MONITORING (conducted by industry)**		
16. If samples are to be taken, describes the sampling process: for example, number of samples, amount, frequency, individual vs. composite, where samples are taken in the process, and percent considered passing	(N/A) Does not describe the sampling process	
17. States that industry is required to follow quality assurance/quality control in regards to fortification	(0) Does not state requirement	
18. States applicability of using qualitative testing to determine the presence or absence of a vitamin or mineral (spot tests, iChecks)	(0) Does not state	
**EXTERNAL MONITORING (conducted by government)**		
19.States requirement for external monitoring at the production site to assure compliance with standards and regulations	(0) Does not state requirement	
20. Describes protocols and systems for regulatory monitoring	(0) Does not describe	
21. If there are two or more government agencies involved in external monitoring, clarifies the roles and responsibilities between different government agencies in external monitoring	(0) Clarifies roles and responsibilities for no agencies	
22. Allows for monitoring to be conducted often enough that problems can be identified and addressed on a timely basis; specifies a timeline for inspections (i.e. once every 6 months, increasing to once every 2 months if a discrepancy is found)	(0) Does not state	
23. If samples are to be taken, describes the sampling process: for example, number of samples, amount, frequency, individual vs. composite, where samples are taken in the process, and percent considered passing	(N/A) Does not describe the sampling process	
24. States applicability of using qualitative testing to determine the presence or absence of a vitamin or mineral (spot tests, iChecks)	(0) Does not state	
25. States registration is required in order to use a logo/be licensed to produce fortified foods	(0) Does not state that registration or licensing is required	
**COMMERCIAL MONITORING (conducted by government)**		
26. Provides justification for commercial monitoring at retail stores	(0) Does not provide justification for commercial monitoring	

27. Describes protocols and systems for commercial monitoring	(0) Does not describe	
28. If there are two or more government agencies involved in commercial monitoring, clarifies the roles and responsibilities between different government agencies in commercial monitoring	(0) Clarifies roles and responsibilities for no agencies	
29. Allows for monitoring to be conducted often enough that problems at the production site or import companies can be identified and addressed on a timely basis; specifies a timeline for inspections (i.e. once every 6 months) or works with production companies to correct noncompliance	(N/A) No commercial monitoring occurs	
30. If samples are to be taken, describes the sampling process: for example, number of samples, amount, frequency, individual vs. composite, where samples are taken in the process, and percent considered passing	(N/A) Does not describe the sampling process	
**IMPORT MONITORING (conducted by government)**		
31. Provides justification for import monitoring at points of entry	(0) Does not provide justification for import monitoring	
32. Describes protocols and systems for import monitoring	(0) Does not state	
33. If there are two or more government agencies involved in import monitoring, clarifies the roles and responsibilities between different government agencies in import monitoring	(0) Clarifies roles and responsibilities for no agencies	
34. If samples are to be taken, describes the sampling process: for example, number of samples, amount, frequency, individual vs. composite, where samples are taken in the process, and percent considered passing	(N/A) Does not describe the sampling process	
**ENFORCEMENT/PENALTIES**		
35. Indicates roles and responsibilities in enforcing the legislation	(0) Does not state	
36. States incentives to start fortification	(0) Does not state	
37. States incentives to continue fortification, including ensuring compliance	(0) Does not state	
38. States penalties to compel compliance	(0) Does not state	
39. Penalties are objectively defined (e.g. first penalty=$100, second penalty=$300)	(N/A) No penalties are stated (Answered (0) to previous question)	
40. States that enforcement is required to include feedback and support to improve performance and correct noncompliance	(0) Does not require	
**LABORATORY**		
41. References required analytical assays for nutrients (e.g. Liquid chromatography-mass spectrometry for folic acid, atomic absorption for iron and zinc.)	(2) References required assays	Tabla 1
42. States recognition that laboratory results are subject to several sources of variation and do not provide conclusive evidence of compliance or noncompliance	(0) Does not state recognition	
43. Focuses on the quantitative analysis of "marker" micronutrients such as iron	(0) Does not state	
**REPORTING**		
44. States how government monitoring results are shared with stakeholders	(0) Does not state how results are shared	

Brazil Maize

Element	Scoring (use drop-down menus)	Comments (open text)
**GENERAL**		
1. States that legislation applies to at least one food vehicle fit for human consumption (types/grades to be fortified)	(2) States at least one type fit for human consumption	Mandating wheat flour and corn flour fortification with the iron and folic acid. This Technical Regulation applies to the mandatory fortification of wheat flour and corn flour with the iron and folic acid. Excluded from this regulation, due to technological processing, are the following products: tapioca flour or corn flower obtained by maceration; flakes; whole wheat flour and durum wheat flour.
2. States the public health objective; purpose and scope of legislation	(2) States the public health objective or general purpose of legislation	Considering that the nutritional anemia caused by iron deficiency is a serious problem on Public Health, it is the nutritional deficiency of greater magnitude in the world, affecting all cycles of life and in Brazil affects around 21% of children under 5 years of age and around 30% of women in their fertile age (PNDS, 2006);<br><br>Considering that, on children, the anemia is associated to the slow growth, to the cognitive deficit and to the low resistance to infections, and on pregnant women is related to the low weight of the fetus when born and also due to the increase on baby mortality;<br><br>Considering that the strategies for intervention recommended by WHO, ratified by Ministry of Health, to control the anemia caused by iron deficiency are education and nutritional guidance, reinforced foods and universal iron supplements for the more vulnerable groups.<br>considering the recommendations from the World Health Organization-WHO and the Pan American Health Organization-PAHO for fortification of food products with iron and folic acid;
3. References latest available science or accepted international norms and recommendations, particularly for items that may not be covered in the country's documents	(2) States the documents referenced	References: 3.18. The Prevention of Neural Tube Defects with Folic Acid. Pan American Health Organization / Word Health Organization, Division of Health Promotion and Protection, Food and Nutrition Program. Centers for Disease Control and Prevention, Birth Defects and Pediatric Genetics-CDC. P. 5-15. 3.19. Iron Fortification: Where Are We in Terms of Iron Compounds PAHO / FNP / USAID Technical Consultation. Nutrition Reviews, Vol. 60, No. 7 (part II), July. 2002 p.61.
4. Provides definitions that includes terms that are specific to fortification (e.g. fortified food, premix, fortificant, food vehicle)	(2) States at least one term related to fortification	Mix is a mixture where the desired ingredient (in this case, iron) is diluted into another product (called the vehicle), so that its application or use is facilitated. Bio-availability is the degree to which specific nutrients found¬ in the foods are used, with reference to the total content (100%) of the ¬main nutrient considered. (MS Ordinance 710/GM of June 10, 1999).
5. Provides repeals (if there is at least one prior document about fortification)	(2) States repeals	Art.9 – this Ordinance enters into force on the date of its publication.<br>Art.10 – The Ordinance Nº 14/GM, January 3, 2000 is revoked, published on Diário Oficial da União nº5, January 8, 2002, section 1, page 59.
6. Provides effective date or gives grace period for when fortification is to begin (e.g. effective 6 months from signing)	(2) States effective date or grace period for when fortification is to begin (e.g. effective 6 months from signing)	Art.9 – this Ordinance enters into force on the date of its publication.<br>Art.10 – The Ordinance Nº 14/GM, January 3, 2000 is revoked, published on Diário Oficial da União nº5, January 8, 2002, section 1, page 59. In another document: Art. 2 Businesses have a term of 18 (eighteen) months from the date of publication of this Regulation to adapt their products
**MICRONUTRIENTS/PREMIX**		
7. States nutrients required	(2) States nutrients	4.1. The addition of iron and folic acid in wheat flour and the corn meal that is pre-packaged in the customer's absence and ready to offer to the consumer, those for industrial use, including baking and flour added to pre-mixtures is mandatory, and each 100g of wheat flour and corn flour should provide at least 4.2 mg (four point two milligrams) of iron and 150 micrograms (one hundred fifty micrograms) of folic acid.

8. States fortificants (chemical compounds) to be used (including fortificants that are allowable as options)	(2) States fortificants for at least one nutrient	4.5. Companies may use the following food-grade iron compounds: dehydrated iron sulfate (dry); ferrous fumarate; reduced iron - 325 mesh Tyler; electrolytic iron - 325 mesh Tyler; Sodium and iron EDTA (NaFeEDTA); and iron glycinate chelate.<br>Other compounds may be used provided that the bioavailability is not inferior to the compounds listed.
9. States fortification levels	(1) States one number only	4.1. The addition of iron and folic acid in wheat flour and the corn meal that is pre-packaged in the customer's absence and ready to offer to the consumer, those for industrial use, including baking and flour added to pre-mixtures is mandatory, and each 100g of wheat flour and corn flour should provide at least 4.2 mg (four point two milligrams) of iron and 150 micrograms (one hundred fifty micrograms) of folic acid.
10. States consideration of bioavailability/biological activity of fortificants	(2) States some consideration of bioavailability (mentions these or related terms)	4.4. Companies should ensure that the food grade iron compounds are bioavailable. AND Bio-availability- this is the proportion of the ingredient ingested that is available for the metabolic process and varies for each type of iron. Ferrous sulfate and fumarate have good bio-availability, however, elemental ¬iron is considered lower. Bio-availability also varies from person to person, since many factors, such as the food itself, the individual's diet and the way in which the product is processed, affect the organism's ability to absorb different types of iron.
11. States consideration of nutrient stability	(2) States consideration of nutrient stability	4.6. Companies should use food-grade folic acid, ensuring the stability of the wheat flour and the corn flour within the period of validity of the same. b) Stability - in the ferrous sulfate and fumarate, there are pro-oxidants that can accelerate the development of rancidity in fats. Since flours have small quantities of fats, the addition of ferrous sulfate or fumarate can reduce its shelf-life.
**COSTING**		
12. States that the cost of fortification is regulated through cost sharing schemes (between government, industry, consumers) or tax measures [to assist industry]	(2) States consideration of either cost regulation method	To plan an iron-added wheat flour fortification program, it is important to determine the costs involved in the proper allocation of resources. In Brazil, since this program is mandatory, the funding regarding the disclosure and clarifications to the consumers about the iron-added flours and the difference they make compared to previously commercialized flours should be borne by the government itself. The costs to establish a national iron fortification program will depend on factors such as the number and size of the mills, the adoption of the quality guarantee system, regulations on the subject and rigor in inspecting the foods as well as the level of iron to be added.<br>For the fortification of flours, the necessary capital is basically concentrated in three items: 1) the iron source, 2) the iron dispenser/feeder and 3) quality control analyses. A list of some of the national suppliers of iron sources (Annex VI), equipment (Annex VII) and laboratories that can perform the necessary analyses (Annex VIII) are presented in the annexes.
13. States consideration of the financial responsibility of monitoring and enforcing fortification (schedule of fees, budget) [on the government side]	(2) Shows consideration that monitoring costs money	...should be borne by the government itself. The costs to establish a national iron fortification program will depend on factors such as the number and size of the mills, the adoption of the quality guarantee system, regulations on the subject and rigor in inspecting the foods as well as the level of iron to be added.<br>For the fortification of flours, the necessary capital is basically concentrated in three items: 1) the iron source, 2) the iron dispenser/feeder and 3) quality control analyses. ALSO see Table 4, part b for breakdown of expected monitoring costs
**LABELING**		
14. Includes some sort of statement/label/logo that makes it clear that the product is fortified	(2) Includes a statement, label, or logo	5. LABELLING<br>5.1. Wheat flours and corn flours should be designated using the conventional name of the product according to the specific legislation, followed by one of the following expressions: Fortified with iron and folic acid, enriched with iron and folic acid or rich in iron and folic acid.

15. Provides guidance on health claims that can be made for this product (specific to micronutrients added through fortification)	(2) Provides guidance on health claims specific to micronutrients added through fortification	According to this ordinance, the use of any expression of a therapeutic nature on the label of essential nutrient added foods is prohibited since these foods follow the General Labeling Standards, according to the specific legislation.
**INTERNAL MONITORING (conducted by industry)**		
16. If samples are to be taken, describes the sampling process: for example, number of samples, amount, frequency, individual vs. composite, where samples are taken in the process, and percent considered passing	(2) States that samples should be taken as part of internal monitoring	Quality control<br>6.2.1. Sampling<br>All flour samples should be collected after they have been packaged. The random collection of at least 3 (three) packages (of 1 or 5kg) from each -sample is recommended. For 50kg sacks, a sample of about 1kg can be taken from 3 sacks, or directly from the mouth of the bagger, for non-automatic systems.
17. States that industry is required to follow quality assurance/quality control in regards to fortification	(2) States requirement of QA/QC for fortification	Entire manual is about QA/QC
18. States applicability of using qualitative testing to determine the presence or absence of a vitamin or mineral (spot tests, iChecks)	(2) States applicability of spot test to determine presence/absence of vitamin or mineral specific to internal monitoring	3) Spot Test: all qualitative or semi-quantitative evaluation (Annex III) of the flour should be performed just after the sample collection so that any problem, such as excess or insufficient adding, can be corrected. Not only should the iron be observed, but also its homogenous distribution in the flour as well.
**EXTERNAL MONITORING (conducted by government)**		
19.States requirement for external monitoring at the production site to assure compliance with standards and regulations	(0) Does not state requirement	
20. Describes protocols and systems for regulatory monitoring	(0) Does not describe	
21. If there are two or more government agencies involved in external monitoring, clarifies the roles and responsibilities between different government agencies in external monitoring	(2) Clarifies roles and responsibilities for more than one agency	Articles 3-5 in English Portaria No 1793 Document. Details responsibilities of the MoH, Commission, and ANVISA.
22. Allows for monitoring to be conducted often enough that problems can be identified and addressed on a timely basis; specifies a timeline for inspections (i.e. once every 6 months, increasing to once every 2 months if a discrepancy is found)	(0) Does not state	
23. If samples are to be taken, describes the sampling process: for example, number of samples, amount, frequency, individual vs. composite, where samples are taken in the process, and percent considered passing	(1) States that samples should be taken (generally)	the Health Surveillance Agency carries out a collection of only one sample per batch for inspection, never the average of various samples from the same batch. The values obtained should always be within the range of ± 20% of the value indicated on the label, according to that indicated in the labeling standards (RDC Resolution No. 40 of 03/21/2001).
24. States applicability of using qualitative testing to determine the presence or absence of a vitamin or mineral (spot tests, iChecks)	(0) Does not state	
25. States registration is required in order to use a logo/be licensed to produce fortified foods	(2) Describes some type of registration or licensing	It is important to highlight that the iron-added wheat flours are exempt from the requirement for registration with the responsible agency from the Ministry of Health (Resolution No. 23 of March 15, 2000). Companies should report only the initiation of manufacturing or product importation to the State, Federal District or Municipal health au¬thorities, according to the template annexed to Resolution 23/2000 and Resolution 22/2000 or through the program for Products Exempted from the Requirement to Register - PRODIR, being able to initiate the commercialization of the product.
**COMMERCIAL MONITORING (conducted by government)**		
26. Provides justification for commercial monitoring at retail stores	(0) Does not provide justification for commercial monitoring	
27. Describes protocols and systems for commercial monitoring	(0) Does not describe	
28. If there are two or more government agencies involved in commercial monitoring, clarifies the roles and responsibilities between different government agencies in commercial monitoring	(0) Clarifies roles and responsibilities for no agencies	
29. Allows for monitoring to be conducted often enough that problems at the production site or import companies can be identified and addressed on a timely basis; specifies a timeline for inspections (i.e. once every 6 months) or works with production companies to correct noncompliance	(N/A) No commercial monitoring occurs	
30. If samples are to be taken, describes the sampling process: for example, number of samples, amount, frequency, individual vs. composite, where samples are taken in the process, and percent considered passing	(N/A) Does not describe the sampling process	
**IMPORT MONITORING (conducted by government)**		

31. Provides justification for import monitoring at points of entry	(0) Does not provide justification for import monitoring	
32. Describes protocols and systems for import monitoring	(0) Does not state	
33. If there are two or more government agencies involved in import monitoring, clarifies the roles and responsibilities between different government agencies in import monitoring	(N/A) Only one government agency involved	Art.5 – For the National Agency of Sanitary Surveillance (ANVISA)<br><br>I – to implement the national monitoring for the wheat and corn flours produced in every mill and industries, as well as products exposed in the market.<br>II – coordinate and regulate the hygiene control of the establishments that produce the flours and the exposed products in the market;<br>III – to analyze and publicize, in a systematic way, the control results and the follow up on the wheat and corn flours;<br>IV – to promote the inclusion of official labs in the control programs for analytical quality;<br>V – to promote awareness in the productive sector in a way to attend to the present health legislation;<br>VI – to monitor the imported wheat and corn flours to check if the comply with the present legislation, in particular for the amounts of iron and folic acid.
34. If samples are to be taken, describes the sampling process: for example, number of samples, amount, frequency, individual vs. composite, where samples are taken in the process, and percent considered passing	(N/A) Does not describe the sampling process	
**ENFORCEMENT/PENALTIES**		
35. Indicates roles and responsibilities in enforcing the legislation	(2) States the role and responsibilities of government in enforcement	Article 14. The penalties herein addressed shall be applied by the competent health surveillance authorities of the Ministry of Health, States, Federal District and Territories, pursuant to their respective duties established in the legislation or delegated thereto by agreements.
36. States incentives to start fortification	(2) States any incentives to encourage fortification initiation (e.g. tax incentives for new equipment or premix)	In Brazil, since this program is mandatory, the funding regarding the disclosure and c¬larifications to the consumers about the iron-added flours and the difference they make compared to previously commercialized flours should be borne by the government itself. The costs to establish a national iron fortification program will depend on factors such as the number and size of the mills, the adoption of the quality guarantee system, regulations on the subject and rigor in inspecting the foods as well as the level of iron to be added.<br>For the fortification of flours, the necessary capital is basically concentrated in three items: 1) the iron source, 2) the iron dispenser/feeder and 3) quality control analyses. A list of some of the national suppliers of iron sources (Annex VI), equipment (Annex VII) and laboratories that can perform the necessary analyses (Annex VIII) are presented in the annexes.
37. States incentives to continue fortification, including ensuring compliance	(0) Does not state	
38. States penalties to compel compliance	(2) States any penalties	Art. 3. Failure to fulfill the terms of this Resolution constitutes a health violation subject to the penalties set forth in Law No. 6437 of August 20, 1977 and other applicable provisions.
39. Penalties are objectively defined (e.g. first penalty=$100, second penalty=$300)	(2) Penalties are objectively laid out in the document (e.g. first penalty=$100, second penalty=$300)	Paragraph 1. The penalty of fine shall consist in payment of the following values: (Included by Provisional Measure no. 2,190-34, 2001)<br><br>I - for minor violations, from R$ 2.000,00 to R$ 75.000,00; (Included by provisional Measure no. 2,190-34, 2001)<br>II - for serious violations, from R$ 75.000,00 to R$ 200.000,00; (Included by provisional Measure no. 2,190-34, 2001)<br>III - for major violations, from R$ 200.000,00 to R$ 1.500.000,00. (Included by provisional Measure no. 2,190-34, 2001)<br>Paragraph 2. The fines herein addressed shall be applied in double amount in the case of recidivism. (Included by provisional Measure no. 2,190-34, 2001)

40. States that enforcement is required to include feedback and support to improve performance and correct noncompliance	(2) Requires any feedback/support to improve performance	Article 6. When imposing penalties and assessing their severity, health surveillance authorities shall take the following into account:<br>I - mitigating and aggravating circumstances;<br><br>II - the severity of violations in view of their consequences to public health;<br>III - criminal record of the offender with regard to sanitary regulations. Article 7. The following are mitigating circumstances:<br>I - the offender's act is not deemed essential to the course of events;<br>II - the sanitary legislation has been misunderstood, which is deemed excusable due to the offender's incapacity to admit the unlawfulness of the act;<br>III - the offender has spontaneously and immediately intended to repair or mitigate the consequences of his/her harmful act to public health;<br>IV - the offender has suffered possibly resistible coercion in order to commit the offense;<br>V - if primary offender and minor violation.
**LABORATORY**		
41. References required analytical assays for nutrients (e.g. Liquid chromatography-mass spectrometry for folic acid, atomic absorption for iron and zinc.)	(2) References required assays	The quantitative analysis can be performed using spectrophotometry or atomic absorption. The references for these two methods are shown below:<br><br>a) Method 944.02 by the AOAC. 16th edition, 1997.<br>b) Method 965.09 by the AOAC. 16th edition, 1995.
42. States recognition that laboratory results are subject to several sources of variation and do not provide conclusive evidence of compliance or noncompliance	(0) Does not state recognition	
43. Focuses on the quantitative analysis of "marker" micronutrients such as iron	(2) Focuses on quantitative analysis of marker micronutrient such as iron	The whole manual is about iron.
**REPORTING**		
44. States how government monitoring results are shared with stakeholders	(2) States how results are shared with stakeholders	III – to analyze and publicize, in a systematic way, the control results and the follow up on the wheat and corn flours (responsibility of ANVISA)

Costa Rica Maize

Element	Scoring (use drop-down menus)	Comments (open text)
**GENERAL**		
1. States that legislation applies to at least one food vehicle fit for human consumption (types/grades to be fortified)	(2) States at least one type fit for human consumption	"Artículo 1°—Las disposiciones del presente reglamento se aplican a la harina de maíz que se utiliza para el consumo humano en el país, sea ésta de producción nacional, donada o importada." "Artículo 3°—La harina de maíz que se utilice en el país para el consumo humano ya sea de uso casero o en la industria alimentaria, deberá estar fortificada con hierro y ácido fólico en forma homogénea y reconstituida con tiamina, niacina y riboflavina. Los costos de dicha disposición serán asumidos por los productores."
2. States the public health objective; purpose and scope of legislation	(2) States the public health objective or general purpose of legislation	"Considerando:<br>1°—Que es función esencial del Estado velar por la salud de la población.<br>2°—Que el hierro es un nutriente indispensable para el desarrollo físico y mental del ser humano."
3. References latest available science or accepted international norms and recommendations, particularly for items that may not be covered in the country's documents	(0) Does not state	
4. Provides definitions that includes terms that are specific to fortification (e.g. fortified food, premix, fortificant, food vehicle)	(2) States at least one term related to fortification	"Artículo 2°—Para efectos del presente reglamento se entenderá por:"
5. Provides repeals (if there is at least one prior document about fortification)	(N/A) No prior documents about fortification	
6. Provides effective date or gives grace period for when fortification is to begin (e.g. effective 6 months from signing)	(2) States effective date or grace period for when fortification is to begin (e.g. effective 6 months from signing)	"Artículo 19.—Rige a partir de su publicación.<br>Transitorio I.—Se otorga un plazo de seis meses a partir de la fecha de publicación del presente Reglamento a efecto de que los productores, importadores y distribuidores cumplan con las disposiciones del presente Reglamento."
**MICRONUTRIENTS/PREMIX**		
7. States nutrients required	(2) States nutrients	"Artículo 3°—La harina de maíz que se utilice en el país para el consumo humano ya sea de uso casero o en la industria alimentaria, deberá estar fortificada con hierro y ácido fólico en forma homogénea y reconstituida con tiamina, niacina y riboflavina. Los costos de dicha disposición serán asumidos por los productores."
8. States fortificants (chemical compounds) to be used (including fortificants that are allowable as options)	(2) States fortificants for at least one nutrient	Artículo 4° "El compuesto de hierro a agregar deberá ser Bisglicinato de hierro, por tanto se requieren 104 mg de dicho compuesto por kilogramo de harina."
9. States fortification levels	(1) States one number only	"Artículo 4°—Los niveles de fortificación mínimos de la harina de maíz serán los siguientes:"
10. States consideration of bioavailability/biological activity of fortificants	(0) Does not state any consideration	
11. States consideration of nutrient stability	(0) Does not state any consideration	
**COSTING**		
12. States that the cost of fortification is regulated through cost sharing schemes (between government, industry, consumers) or tax measures [to assist industry]	(0) Does not state any consideration	
13. States consideration of the financial responsibility of monitoring and enforcing fortification (schedule of fees, budget) [on the government side]	(2) Shows consideration that monitoring costs money	This document is focused on commercial monitoring. (page 11) "a. Plan, presupuesto y calendarización"
**LABELING**		
14. Includes some sort of statement/label/logo that makes it clear that the product is fortified	(2) Includes a statement, label, or logo	"Artículo 13.—El etiquetado de la harina de maíz se ajustará a lo estipulado en la Norma General de Etiquetado de los Alimentos Preenvasados y deberá especificar que se trata de un producto fortificado con la leyenda "HARINA DE MAÍZ FORTIFICADA" o "HARINA DE MAÍZ ENRIQUECIDA", deberá contener un panel que incluya los micronutrientes utilizados en la fortificación y las cantidades indicadas en el artículo 4° del presente reglamento. Al pie del panel deberá indicar que dicha cantidad de hierro equivale a 88 mg de hierro en forma de sulfato ferroso"
15. Provides guidance on health claims that can be made for this product (specific to micronutrients added through fortification)	(2) Provides guidance on health claims specific to micronutrients added through fortification	"7.1 Declaraciones Nutricionales<br>Las únicas declaraciones de propiedades nutricionales permitidas deben ser las que se refieran a energía, proteínas, carbohidratos, grasas y los componentes de las mismas, fibra, vitaminas y minerales para los cuales se hayan establecido recomendaciones nutricionales."<br>"7.4 Declaraciones de Propiedades Saludables"<br>Anexo G "3.9 Ácido fólico y defectos del conducto (tubo) neural:"
**INTERNAL MONITORING (conducted by industry)**		

16. If samples are to be taken, describes the sampling process: for example, number of samples, amount, frequency, individual vs. composite, where samples are taken in the process, and percent considered passing	(N/A) Does not describe the sampling process	
17. States that industry is required to follow quality assurance/quality control in regards to fortification	(2) States requirement of QA/QC for fortification	"Artículo 5°—La garantía de calidad o control interno de la harina de maíz con los niveles indicados en el artículo anterior son de responsabilidad de los industriales y de los importadores de harina de maíz."
18. States applicability of using qualitative testing to determine the presence or absence of a vitamin or mineral (spot tests, iChecks)	(0) Does not state	
**EXTERNAL MONITORING (conducted by government)**		
19.States requirement for external monitoring at the production site to assure compliance with standards and regulations	(2) States requirement for external monitoring or the need for audits/inspections	"Artículo 9°—La fiscalización y el monitoreo de la calidad de la fortificación de la harina de maíz en fábricas, sitios de expendio y otros son responsabilidad de las autoridades del Ministerio de Salud, quienes diseñarán y establecerán un sistema de control de calidad para la harina de maíz fortificada."
20. Describes protocols and systems for regulatory monitoring	(0) Does not describe	
21. If there are two or more government agencies involved in external monitoring, clarifies the roles and responsibilities between different government agencies in external monitoring	(N/A) Only one government agency involved	"Artículo 18.—El Ministerio de Salud coordinará con los demás entes públicos involucrados en la materia todas las acciones necesarias para asegurar la cabal aplicación y cumplimiento del presente reglamento."
22. Allows for monitoring to be conducted often enough that problems can be identified and addressed on a timely basis; specifies a timeline for inspections (i.e. once every 6 months, increasing to once every 2 months if a discrepancy is found)	(0) Does not state	
23. If samples are to be taken, describes the sampling process: for example, number of samples, amount, frequency, individual vs. composite, where samples are taken in the process, and percent considered passing	(N/A) Does not describe the sampling process	
24. States applicability of using qualitative testing to determine the presence or absence of a vitamin or mineral (spot tests, iChecks)	(0) Does not state	
25. States registration is required in order to use a logo/be licensed to produce fortified foods	(0) Does not state that registration or licensing is required	
**COMMERCIAL MONITORING (conducted by government)**		
26. Provides justification for commercial monitoring at retail stores	(2) Provides justification for commercial monitoring	"Artículo 7°—El Ministerio de Salud se reserva el derecho de confirmar, mediante muestreo o análisis de muestras en el mercado o en las bodegas del importador, el cumplimiento de la harina de maíz importada con los niveles de fortificación establecidos en el presente reglamento. Los costos que esto ocasione serán asumidos por el importador."
27. Describes protocols and systems for commercial monitoring	(1) Does not explicitly describe, but references protocols and systems for regulatory monitoring	"Artículo 7°—El Ministerio de Salud se reserva el derecho de confirmar, mediante muestreo o análisis de muestras en el mercado o en las bodegas del importador, el cumplimiento de la harina de maíz importada con los niveles de fortificación establecidos en el presente reglamento. Los costos que esto ocasione serán asumidos por el importador."
28. If there are two or more government agencies involved in commercial monitoring, clarifies the roles and responsibilities between different government agencies in commercial monitoring	(N/A) Only one government agency involved	"Artículo 18.—El Ministerio de Salud coordinará con los demás entes públicos involucrados en la materia todas las acciones necesarias para asegurar la cabal aplicación y cumplimiento del presente reglamento."
29. Allows for monitoring to be conducted often enough that problems at the production site or import companies can be identified and addressed on a timely basis; specifies a timeline for inspections (i.e. once every 6 months) or works with production companies to correct noncompliance	(1) Makes mention of a timeline	This is focused on commercial monitoring only:<br>"• Basado en el número total de distritos del país, planifique por lo menos una visita al año a cada uno de los distritos del país. La frecuencia e intensidad de muestreo depende de la densidad poblacional, la cantidad de alimento vendido en la región y factores de riesgos tales como la ubicación cerca de fronteras con otros países donde el alimento no se fortifica. Se podrían incluir las panaderías como un lugar para tomar muestras."

30. If samples are to be taken, describes the sampling process: for example, number of samples, amount, frequency, individual vs. composite, where samples are taken in the process, and percent considered passing	(2) States that samples should be taken as part of commercial monitoring	"Este manual describe los procedimientos para realizar las visitas de inspección en cualquier establecimiento que venda alimentos fortificados."<br>"V. Procedimientos<br>a. Visitas de los inspectores<br>5. Escoja un empaque sellado de aproximadamente 500 g (0.5 kg) de cada marca de alimento fortificado en el establecimiento. Si el alimento no se encuentra en esta presentación, tome la muestra de alimento en la siguiente presentación para venta al detalle. Si los envases son más pequeños, recolecte suficientes paquetes para llegar al peso especificado (Ej.: 2 empaques de 250 g).<br>6. Si el alimento se vende por peso o volumen de sacos más grandes, tome una muestra de aproximadamente 500 g (0.5 kg) de este producto. Asegúrese que el saco es nuevo, de otra forma no existe garantía que el producto adentro del mismo corresponde al nombre de la fábrica en la etiqueta.<br>7. Empaque las muestras dentro de una caja y transpórtelas a la oficina local de la Autoridad Sanitaria, donde el encargado de la misma enviará las muestras y copias de los informes a la sede de la Autoridad Sanitaria con la frecuencia que se ha determinado en el plan de trabajo anual."
**IMPORT MONITORING (conducted by government)**		
31. Provides justification for import monitoring at points of entry	(2) Provides justification for import monitoring	"Artículo 5°—La garantía de calidad o control interno de la harina de maíz con los niveles indicados en el artículo anterior son de responsabilidad de los industriales y de los importadores de harina de maíz."
32. Describes protocols and systems for import monitoring	(1) Does not explicitly state, but references protocols and systems for import monitoring	"Artículo 6°—Para autorizar el desalmacenaje de la harina de maíz importada, el importador deberá demostrar en el certificado de calidad del país de origen o certificado de análisis realizado en un laboratorio acreditado, la conformidad del producto con la fortificación establecida en el presente reglamento, para cada partida de importaciones." "Artículo 7°—El Ministerio de Salud se reserva el derecho de confirmar, mediante muestreo o análisis de muestras en el mercado o en las bodegas del importador, el cumplimiento de la harina de maíz importada con los niveles de fortificación establecidos en el presente reglamento. Los costos que esto ocasione serán asumidos por el importador."
33. If there are two or more government agencies involved in import monitoring, clarifies the roles and responsibilities between different government agencies in import monitoring	(N/A) Only one government agency involved	"Artículo 18.—El Ministerio de Salud coordinará con los demás entes públicos involucrados en la materia todas las acciones necesarias para asegurar la cabal aplicación y cumplimiento del presente reglamento."
34. If samples are to be taken, describes the sampling process: for example, number of samples, amount, frequency, individual vs. composite, where samples are taken in the process, and percent considered passing	(N/A) Does not describe the sampling process	
**ENFORCEMENT/PENALTIES**		
35. Indicates roles and responsibilities in enforcing the legislation	(2) States the role and responsibilities of government in enforcement	"Artículo 7°—El Ministerio de Salud se reserva el derecho de confirmar, mediante muestreo o análisis de muestras en el mercado o en las bodegas del importador, el cumplimiento de la harina de maíz importada con los niveles de fortificación establecidos en el presente reglamento. Los costos que esto ocasione serán asumidos por el importador."<br>"Artículo 9°—La fiscalización y el monitoreo de la calidad de la fortificación de la harina de maíz en fábricas, sitios de expendio y otros son responsabilidad de las autoridades del Ministerio de Salud, quienes diseñarán y establecerán un sistema de control de calidad para la harina de maíz fortificada."<br>"Artículo 10.—El Ministerio podrá verificar los niveles de las vitaminas y minerales en la premezcla y en la harina de maíz, mediante análisis cuantitativos eventuales y establecerá los criterios técnicos de los procedimientos y técnicas de laboratorio para el análisis de las muestras de harina de maíz fortificada."<br>"Artículo 15.—El etiquetado nutricional y la publicidad serán controlados por el Ministerio de Salud."
36. States incentives to start fortification	(0) Does not state	
37. States incentives to continue fortification, including ensuring compliance	(0) Does not state	

38. States penalties to compel compliance	(2) States any penalties	"Artículo 8°.—En caso de establecerse que un lote de harina de maíz producida en el país o importada no esté debidamente fortificada, se procederá al decomiso de la cantidad total conforme a las disposiciones contenidas en la Ley General de Salud."
39. Penalties are objectively defined (e.g. first penalty=$100, second penalty=$300)	(0) Penalties are not objectively laid out	
40. States that enforcement is required to include feedback and support to improve performance and correct noncompliance	(0) Does not require	
**LABORATORY**		
41. References required analytical assays for nutrients (e.g. Liquid chromatography-mass spectrometry for folic acid, atomic absorption for iron and zinc.)	(0) Does not state requirements	
42. States recognition that laboratory results are subject to several sources of variation and do not provide conclusive evidence of compliance or noncompliance	(0) Does not state recognition	
43. Focuses on the quantitative analysis of "marker" micronutrients such as iron	(2) Focuses on quantitative analysis of marker micronutrient such as iron	"c. Análisis de las muestras<br>9. Al recibir las muestras, el laboratorio deberá detectar la presencia de los micronutrientes indicador[4] en todas las muestras simples usando pruebas cualitativas."
**REPORTING**		
44. States how government monitoring results are shared with stakeholders	(2) States how results are shared with stakeholders	This document only refers to the commercial monitoring results. "Los resultados de las actividades de monitoreo comercial se deberían consolidar dos veces al año en informes. Estos informes contribuyen a definir el grado de éxito en el cumplimiento de las metas de fortificación, exponer los obstáculos que se necesitan superar y definir las acciones a seguir. Se recomienda que se prepare y publique un informe anual, en el cual se presente la información gráficamente para describir la situación de los programas de alimentos fortificados en el país, junto con información de otras actividades de monitoreo de las autoridades sanitarias o de vigilancia."

El Salvador Maize

Element	Scoring (use drop-down menus)	Comments (open text)
**GENERAL**		
1. States that legislation applies to at least one food vehicle fit for human consumption (types/grades to be fortified)	(2) States at least one type fit for human consumption	Refers to nixtamalized maize flour that is nationally produced, imported or donated. "1. OBJETO…2. CAMPO DE APLICACION"
2. States the public health objective; purpose and scope of legislation	(2) States the public health objective or general purpose of legislation	"El Programa Nacional de Fortificación de Alimentos constituye una intervención prioritaria para el país, ya que se facilitan los micro nutrientes necesarios para la población salvadoreña, dicho aporte se ve reflejado en la disminución de las deficiencias por micro nutrientes especialmente en yodo y vitamina "A". Para dar respuesta a las necesidades nutricionales de la población, el Gobierno de la República, organismos internacionales y la empresa privada impulsan permanentemente la fortificación de: Sal con Yodo, Azúcar con vitamina "A", Harinas de Trigo y de Maíz Nixtamalizado con Hierro, Acido Fólico y vitaminas del complejo "B". "
3. References latest available science or accepted international norms and recommendations, particularly for items that may not be covered in the country's documents	(0) Does not state	
4. Provides definitions that includes terms that are specific to fortification (e.g. fortified food, premix, fortificant, food vehicle)	(2) States at least one term related to fortification	"3. DEFINICIONES"
5. Provides repeals (if there is at least one prior document about fortification)	(2) States repeals	"11.2 CORRESPONDENCIA<br>NSO 67.03.02:03 "Harinas. Harina de Maiz Nixtamalizado""
6. Provides effective date or gives grace period for when fortification is to begin (e.g. effective 6 months from signing)	(0) Does not state	
**MICRONUTRIENTS/PREMIX**		
7. States nutrients required	(2) States nutrients	"8. FORTIFICACION"
8. States fortificants (chemical compounds) to be used (including fortificants that are allowable as options)	(2) States fortificants for at least one nutrient	For all nutrients, states fortification compounds. "8. FORTIFICACION"
9. States fortification levels	(1) States one number only	States legal minimum levels. "8. FORTIFICACION"
10. States consideration of bioavailability/biological activity of fortificants	(2) States some consideration of bioavailability (mentions these or related terms)	"8. FORTIFICACION… El hierro se adicionará como fumarato ferroso. Se podrá utilizar otro compuesto de hierro que tenga mayor biodisponibilidad que los anteriores, y no afecte las características tecnológicas de la harina de maíz nixtamalizado, demostrado por investigaciones científicas y avaladas por el Ministerio de Salud Pública y Asistencia Social."
11. States consideration of nutrient stability	(0) Does not state any consideration	
**COSTING**		
12. States that the cost of fortification is regulated through cost sharing schemes (between government, industry, consumers) or tax measures [to assist industry]	(0) Does not state any consideration	
13. States consideration of the financial responsibility of monitoring and enforcing fortification (schedule of fees, budget) [on the government side]	(0) Does not state any consideration	
**LABELING**		
14. Includes some sort of statement/label/logo that makes it clear that the product is fortified	(0) Does not include statement, label, or logo	
15. Provides guidance on health claims that can be made for this product (specific to micronutrients added through fortification)	(2) Provides guidance on health claims specific to micronutrients added through fortification	"7.1 Declaraciones Nutricionales<br>Las únicas declaraciones de propiedades nutricionales permitidas deben ser las que se refieran a energía, proteínas, carbohidratos, grasas y los componentes de las mismas, fibra, vitaminas y minerales para los cuales se hayan establecido recomendaciones nutricionales."<br>"7.4 Declaraciones de Propiedades Saludables"<br>Anexo G "3.9 Ácido fólico y defectos del conducto (tubo) neural:"
**INTERNAL MONITORING (conducted by industry)**		
16. If samples are to be taken, describes the sampling process: for example, number of samples, amount, frequency, individual vs. composite, where samples are taken in the process, and percent considered passing	(N/A) Does not describe the sampling process	
17. States that industry is required to follow quality assurance/quality control in regards to fortification	(0) Does not state requirement	
18. States applicability of using qualitative testing to determine the presence or absence of a vitamin or mineral (spot tests, iChecks)	(0) Does not state	
**EXTERNAL MONITORING (conducted by government)**		
19.States requirement for external monitoring at the production site to assure compliance with standards and regulations	(2) States requirement for external monitoring or the need for audits/inspections	(page 30) "Actividad 1: Muestreo en fábricas de harina de maíz nixtamalizado con hierro"

20. Describes protocols and systems for regulatory monitoring	(2) Includes checklists or provides detailed description of regulatory monitoring procedures	Only describes sample taking. And going back to try to identify the problem if samples don't meet norms. (page 31) "8. Visita de seguimiento por resultados fuera de norma: El delegado de salud realiza visitas para determinar cual es la posible causa de la inadecuada fortificación y recomienda las acciones correctivas necesarias a implementar, a la vez se elabora el informe respectivo y envía copia a la UAA."
21. If there are two or more government agencies involved in external monitoring, clarifies the roles and responsibilities between different government agencies in external monitoring	(2) Clarifies roles and responsibilities for more than one agency	(page 30) "Tareas del Muestreo en fábricas de harina de maíz nixtamalizado…" e.g. "6. Envío de resultados de análisis fuera de norma: Los informes de resultados que se encuentren fuera de norma, son enviados por el Laboratorio de Control de Calidad de Alimentos y Aguas a la Unidad de Atención al Ambiente, en el plazo de 8 días, posteriores a la recepción de la muestra. En la UAA, los resultados son ingresados a la base de datos y luego se remiten vía fax a la fábrica, Región, SIBASI y establecimientos de salud respectivos, así mismo, se debe verificar que la fabrica de harina de maíz posean los resultados enviados por la UAA, de lo contrario el delegado de salud debe informar sobre los mismos."
22. Allows for monitoring to be conducted often enough that problems can be identified and addressed on a timely basis; specifies a timeline for inspections (i.e. once every 6 months, increasing to once every 2 months if a discrepancy is found)	(2) Describes frequency and how it is responsive to the needs of industry or the stage of fortification implementation	(page 29) "1. Toma de muestras: Se toman 2 muestras semanales en duplicado o una cantidad mayor si fuere necesario a solicitud de la UAA, alternando marcas. " (page 34) "9.1 Proceso: Evaluación del programa de fortificación de alimentos<br>9.1.1 En el establecimiento local de salud<br>1. Programación de muestreo y visitas de vigilancia: Se recibe la calendarización anual por parte del SIBASI. Con ésta programación, respetando el número de muestras y fechas asignadas por la UAA, el Inspector Técnico en saneamiento ambiental o supervisor local, las incluye en la programación anual operativa." (page 37) "6. Acción a realizar: Con base a resultados obtenidos de la evaluación se debe:<br>a) Priorizar visitas de vigilancia en aquellos establecimientos productores en los cuales en base a evaluación se haya identificado una deficiente fortificación."
23. If samples are to be taken, describes the sampling process: for example, number of samples, amount, frequency, individual vs. composite, where samples are taken in the process, and percent considered passing	(2) States that samples should be taken as part of external monitoring	(page 30) "Tareas del Muestreo en fábricas de harina de maíz nixtamalizado…"
24. States applicability of using qualitative testing to determine the presence or absence of a vitamin or mineral (spot tests, iChecks)	(0) Does not state	
25. States registration is required in order to use a logo/be licensed to produce fortified foods	(0) Does not state that registration or licensing is required	
**COMMERCIAL MONITORING (conducted by government)**		
26. Provides justification for commercial monitoring at retail stores	(2) Provides justification for commercial monitoring	(page 32) "Actividad 2: Muestreo en lugares de distribución de producto nacional e importado"
27. Describes protocols and systems for commercial monitoring	(2) Includes checklists or provides detailed description of regulatory monitoring procedures	(page 32) "Actividad 2: Muestreo en lugares de distribución de producto nacional e importado"
28. If there are two or more government agencies involved in commercial monitoring, clarifies the roles and responsibilities between different government agencies in commercial monitoring	(2) Clarifies roles and responsibilities for more than one agency	(page 33) For example "6. Envío de resultados de análisis fuera de norma: Los de resultados análisis que se encuentren fuera de norma, son enviados por el Laboratorio de Control de Calidad de Alimentos y Aguas a la Unidad de Atención al Ambiente, en el plazo de 8 días, posteriores a la recepción de la muestra. En la UAA, los resultados son ingresados a la base de datos y luego se remiten vía fax a Región, SIBASI y establecimiento de salud respectivos."

29. Allows for monitoring to be conducted often enough that problems at the production site or import companies can be identified and addressed on a timely basis; specifies a timeline for inspections (i.e. once every 6 months) or works with production companies to correct noncompliance	(2) Describes frequency and how it is responsive to the needs of industry or the stage of fortification implementation	(page 32) "1. Toma de Muestras: Cada SIBASI debe tomar 3 muestras mensuales o una cantidad mayor si fuese necesario a solicitud de la UAA (2 muestra de harina importada y 1 muestras de harina nacional). Las muestras se toman en duplicado, alternando marcas, deben enviarse debidamente identificadas incluyendo lote de producción y país de procedencia para el caso de importadas. Los puntos de muestreo pueden ser tiendas, mercados, supermercados y otros." (page 34) "9.1 Proceso: Evaluación del programa de fortificación de alimentos 9.1.1 En el establecimiento local de salud 1. Programación de muestreo y visitas de vigilancia: Se recibe la calendarización anual por parte del SIBASI. Con ésta programación, respetando el número de muestras y fechas asignadas por la UAA, el Inspector Técnico en saneamiento ambiental o supervisor local, las incluye en la programación anual operativa." (page 37) "6. Acción a realizar: Con base a resultados obtenidos de la evaluación se debe: a) Priorizar visitas de vigilancia en aquellos establecimientos productores en los cuales en base a evaluación se haya identificado una deficiente fortificación."
30. If samples are to be taken, describes the sampling process: for example, number of samples, amount, frequency, individual vs. composite, where samples are taken in the process, and percent considered passing	(2) States that samples should be taken as part of commercial monitoring	(page 32) "Actividad 2: Muestreo en lugares de distribución de producto nacional e importado"
**IMPORT MONITORING (conducted by government)**		
31. Provides justification for import monitoring at points of entry	(2) Provides justification for import monitoring	(page 32) "Actividad 2: Muestreo en lugares de distribución de producto nacional e importado"
32. Describes protocols and systems for import monitoring	(2) Includes checklists or detailed description of import monitoring procedures	(page 32) "Actividad 2: Muestreo en lugares de distribución de producto nacional e importado"
33. If there are two or more government agencies involved in import monitoring, clarifies the roles and responsibilities between different government agencies in import monitoring	(2) Clarifies roles and responsibilities for more than one agency	(page 33) For example "6. Envío de resultados de análisis fuera de norma: Los de resultados análisis que se encuentren fuera de norma, son enviados por el Laboratorio de Control de Calidad de Alimentos y Aguas a la Unidad de Atención al Ambiente, en el plazo de 8 días, posteriores a la recepción de la muestra. En la UAA, los resultados son ingresados a la base de datos y luego se remiten vía fax a Región, SIBASI y establecimiento de salud respectivos."
34. If samples are to be taken, describes the sampling process: for example, number of samples, amount, frequency, individual vs. composite, where samples are taken in the process, and percent considered passing	(2) States that samples should be taken as part of import monitoring	(page 32) "Actividad 2: Muestreo en lugares de distribución de producto nacional e importado"
**ENFORCEMENT/PENALTIES**		
35. Indicates roles and responsibilities in enforcing the legislation	(2) States the role and responsibilities of government in enforcement	(Ref 1)"12. VILIGANCIA Y VERIFICACION". (Ref 5) "3.1 Coordinación de la vigilancia en el nivel superior... 3.2 Coordinación de la vigilancia en el nivel regional... 3.3 Vigilancia a nivel SIBASIs y establecimientos de salud…"
36. States incentives to start fortification	(0) Does not state	
37. States incentives to continue fortification, including ensuring compliance	(0) Does not state	
38. States penalties to compel compliance	(2) States any penalties	(page 37) "6. Acción a realizar: Con base a resultados obtenidos de la evaluación se debe: ...b) En aquellos casos que los establecimientos productores de alimentos fortificados sean reincidentes o no corrijan las deficiencias identificadas, se debe solicitar apoyo técnico y jurídico previo a iniciar un expediente legal administrativo, lo cual puede conllevar a la aplicación de multas, sanciones y cierres temporales o definitivos si fuere necesario."
39. Penalties are objectively defined (e.g. first penalty=$100, second penalty=$300)	(0) Penalties are not objectively laid out	
40. States that enforcement is required to include feedback and support to improve performance and correct noncompliance	(0) Does not require	
**LABORATORY**		
41. References required analytical assays for nutrients (e.g. Liquid chromatography-mass spectrometry for folic acid, atomic absorption for iron and zinc.)	(0) Does not state requirements	(Ref 1) Does *not* list nutrient assays in "10. METODOS DE ANALISIS Y ENSAYO".
42. States recognition that laboratory results are subject to several sources of variation and do not provide conclusive evidence of compliance or noncompliance	(0) Does not state recognition	

43. Focuses on the quantitative analysis of "marker" micronutrients such as iron	(0) Does not state	
**REPORTING**		
44. States how government monitoring results are shared with stakeholders	(2) States how results are shared with stakeholders	(page 39) A nivel nacional "4. Redacción de informe: El delegado de salud responsable del programa de vigilancia de la fortificación de alimentos, elabora informe y es remitido a la Dirección General de Salud."

Kenya Maize

Element	Scoring (use drop-down menus)	Comments (open text)
**GENERAL**		
1. States that legislation applies to at least one food vehicle fit for human consumption (types/grades to be fortified)	(2) States at least one type fit for human consumption	DEAS 768 - 1 Scope - maize meal and maize flour from common maize intended for human consumption
2. States the public health objective; purpose and scope of legislation	(2) States the public health objective or general purpose of legislation	DEAS 768 - Introduction - recognition of high levels of malnutrition
3. References latest available science or accepted international norms and recommendations, particularly for items that may not be covered in the country's documents	(2) States the documents referenced	319. Where no specifications are set out in any part of these regulations for the fortification of any food articles, but specifications have been established by the joint FAO/WHO Codex Alimentarius Commission, the specifications of the Codex Alimentarius Commission shall apply.
4. Provides definitions that includes terms that are specific to fortification (e.g. fortified food, premix, fortificant, food vehicle)	(2) States at least one term related to fortification	Section 3 Definitions
5. Provides repeals (if there is at least one prior document about fortification)	(2) States repeals	DEAS 768 - Within 6 months of a declaration of an EA Standard, Partner States shall adopt the approved text without deviation and withdraw any existing national standard
6. Provides effective date or gives grace period for when fortification is to begin (e.g. effective 6 months from signing)	(2) States effective date or grace period for when fortification is to begin (e.g. effective 6 months from signing)	DEAS 768 - Within 6 months of a declaration of an EA Standard, Partner States shall adopt the approved text without deviation and withdraw any existing national standard
**MICRONUTRIENTS/PREMIX**		
7. States nutrients required	(2) States nutrients	Table 3
8. States fortificants (chemical compounds) to be used (including fortificants that are allowable as options)	(2) States fortificants for at least one nutrient	Table 3
9. States fortification levels	(2) States a range or number with +/-	Gives range around specified value
10. States consideration of bioavailability/biological activity of fortificants	(0) Does not state any consideration	
11. States consideration of nutrient stability	(2) States consideration of nutrient stability	5.4.4 The supplier of the premix should provide an accompanying stability data and certificate of analysis for the fortificants and premixes
**COSTING**		
12. States that the cost of fortification is regulated through cost sharing schemes (between government, industry, consumers) or tax measures [to assist industry]	(0) Does not state any consideration	
13. States consideration of the financial responsibility of monitoring and enforcing fortification (schedule of fees, budget) [on the government side]	(0) Does not state any consideration	
**LABELING**		
14. Includes some sort of statement/label/logo that makes it clear that the product is fortified	(2) Includes a statement, label, or logo	8.1.ix
15. Provides guidance on health claims that can be made for this product (specific to micronutrients added through fortification)	(2) Provides guidance on health claims specific to micronutrients added through fortification	Section 8.2
**INTERNAL MONITORING (conducted by industry)**		
16. If samples are to be taken, describes the sampling process: for example, number of samples, amount, frequency, individual vs. composite, where samples are taken in the process, and percent considered passing	(2) States that samples should be taken as part of internal monitoring	taking 500 g samples of the fortified flour every hour to be used for preparing shift composite samples, and that the spot-test for iron is being used for confirming that the premix is being delivered. The composite samples must be labeled with the day and shift of the sample.
17. States that industry is required to follow quality assurance/quality control in regards to fortification	(2) States requirement of QA/QC for fortification	This manual describes the steps to be carried out to ensure quality of maize flour fortified with vitamins and minerals. In general, this guideline covers the receipt and inspection of the premix, the maize flour fortification process and quality control of the fortified maize flour. The guideline also includes an iron spot test for confirming that the flour is being fortified with a premix containing that nutrient.
18. States applicability of using qualitative testing to determine the presence or absence of a vitamin or mineral (spot tests, iChecks)	(2) States applicability of spot test to determine presence/absence of vitamin or mineral specific to internal monitoring	The guideline also includes an iron spot test for confirming that the flour is being fortified with a premix containing that nutrient.
**EXTERNAL MONITORING (conducted by government)**		
19.States requirement for external monitoring at the production site to assure compliance with standards and regulations	(2) States requirement for external monitoring or the need for audits/inspections	Public Health Officers shall visit the maize mills at different times throughout the year to carry out technical audits and inspection of the fortification process and product.
20. Describes protocols and systems for regulatory monitoring	(1) Does not explicitly describe, but references protocols and systems for regulatory monitoring	Public Health Officers shall visit the maize mills at different times throughout the year to carry out technical audits and inspection of the fortification process and product. This work by government personnel is mainly based on the review of records kept by the producer and so it is important to keep in mind that "what has not been recorded has not been done".

21. If there are two or more government agencies involved in external monitoring, clarifies the roles and responsibilities between different government agencies in external monitoring	(N/A) Only one government agency involved	Public Health Officers.
22. Allows for monitoring to be conducted often enough that problems can be identified and addressed on a timely basis; specifies a timeline for inspections (i.e. once every 6 months, increasing to once every 2 months if a discrepancy is found)	(1) Makes mention of a timeline	Doesn't say how often
23. If samples are to be taken, describes the sampling process: for example, number of samples, amount, frequency, individual vs. composite, where samples are taken in the process, and percent considered passing	(N/A) Does not describe the sampling process	
24. States applicability of using qualitative testing to determine the presence or absence of a vitamin or mineral (spot tests, iChecks)	(0) Does not state	
25. States registration is required in order to use a logo/be licensed to produce fortified foods	(0) Does not state that registration or licensing is required	
**COMMERCIAL MONITORING (conducted by government)**		
26. Provides justification for commercial monitoring at retail stores	(0) Does not provide justification for commercial monitoring	
27. Describes protocols and systems for commercial monitoring	(0) Does not describe	
28. If there are two or more government agencies involved in commercial monitoring, clarifies the roles and responsibilities between different government agencies in commercial monitoring	(0) Clarifies roles and responsibilities for no agencies	
29. Allows for monitoring to be conducted often enough that problems at the production site or import companies can be identified and addressed on a timely basis; specifies a timeline for inspections (i.e. once every 6 months) or works with production companies to correct noncompliance	(0) Does not state	
30. If samples are to be taken, describes the sampling process: for example, number of samples, amount, frequency, individual vs. composite, where samples are taken in the process, and percent considered passing	(N/A) Does not describe the sampling process	
**IMPORT MONITORING (conducted by government)**		
31. Provides justification for import monitoring at points of entry	(2) Provides justification for import monitoring	
32. Describes protocols and systems for import monitoring	(1) Does not explicitly state, but references protocols and systems for import monitoring	
33. If there are two or more government agencies involved in import monitoring, clarifies the roles and responsibilities between different government agencies in import monitoring	(0) Clarifies roles and responsibilities for no agencies	
34. If samples are to be taken, describes the sampling process: for example, number of samples, amount, frequency, individual vs. composite, where samples are taken in the process, and percent considered passing	(N/A) Does not describe the sampling process	
**ENFORCEMENT/PENALTIES**		
35. Indicates roles and responsibilities in enforcing the legislation	(0) Does not state	
36. States incentives to start fortification	(0) Does not state	
37. States incentives to continue fortification, including ensuring compliance	(0) Does not state	
38. States penalties to compel compliance	(0) Does not state	
39. Penalties are objectively defined (e.g. first penalty=$100, second penalty=$300)	(N/A) No penalties are stated (Answered (0) to previous question)	
40. States that enforcement is required to include feedback and support to improve performance and correct noncompliance	(0) Does not require	
**LABORATORY**		
41. References required analytical assays for nutrients (e.g. Liquid chromatography-mass spectrometry for folic acid, atomic absorption for iron and zinc.)	(2) References required assays	
42. States recognition that laboratory results are subject to several sources of variation and do not provide conclusive evidence of compliance or noncompliance	(0) Does not state recognition	
43. Focuses on the quantitative analysis of "marker" micronutrients such as iron	(2) Focuses on quantitative analysis of marker micronutrient such as iron	Send the samples to an external laboratory to determine their iron content quantitatively.
**REPORTING**		
44. States how government monitoring results are shared with stakeholders	(0) Does not state how results are shared	Reports seem to be all internal

Mexico Maize

Element	Scoring (use drop-down menus)	Comments (open text)
**GENERAL**		
1. States that legislation applies to at least one food vehicle fit for human consumption (types/grades to be fortified)	(2) States at least one type fit for human consumption	"1.3 Esta Norma Oficial Mexicana establece los nutrimentos que se deben adicionar y restituir en las harinas de trigo y de maíz nixtamalizado y su nivel de adición, exceptuándose las utilizadas para: frituras, como texturizantes o espesantes y base para harinas preparadas.<br>1.4 Esta Norma Oficial Mexicana es de observancia obligatoria en el Territorio Nacional para las personas físicas o morales que se dedican al proceso o importación de los productos objeto de esta Norma destinados a los consumidores en el Territorio Nacional."
2. States the public health objective; purpose and scope of legislation	(0) Does not state	
3. References latest available science or accepted international norms and recommendations, particularly for items that may not be covered in the country's documents	(2) States the documents referenced	"10 Concordancia con normas internacionales<br>Esta norma no es equivalente con normas internacionales o normas mexicanas, excepto el apartado 5.2.2 referente a harinas de cereales, sémolas o semolinas en donde es parcialmente equivalente a:<br>Norma Codex para la harina de trigo. Codex Stan 152-1985 (Rev. 1-1995).<br>11 Bibliografía"
4. Provides definitions that includes terms that are specific to fortification (e.g. fortified food, premix, fortificant, food vehicle)	(2) States at least one term related to fortification	"3. Definiciones"
5. Provides repeals (if there is at least one prior document about fortification)	(2) States repeals	"13 Vigencia<br>...<br>13.2 A su entrada en vigor, la presente norma oficial mexicana cancela las Normas Oficiales Mexicanas:"
6. Provides effective date or gives grace period for when fortification is to begin (e.g. effective 6 months from signing)	(2) States effective date or grace period for when fortification is to begin (e.g. effective 6 months from signing)	"13 Vigencia<br>13.1 La presente Norma Oficial Mexicana entrará en vigor a los ciento ochenta días naturales contados a partir de la fecha de su publicación en el Diario Oficial de la Federación."
**MICRONUTRIENTS/PREMIX**		
7. States nutrients required	(2) States nutrients	"5.2.2.7 Especificaciones nutrimentales<br>i) Las harinas de trigo y de maíz nixtamalizado deben ser restituidas con los siguientes nutrimentos y en los niveles que se indican a continuación. [table with thiamin, riboflavin, niacin]<br>ii) Las harinas de trigo y de maíz nixtamalizado deben ser adicionadas con los siguientes nutrimentos y en los niveles que se indican a continuación. [table with folic acid, iron, zinc]"
8. States fortificants (chemical compounds) to be used (including fortificants that are allowable as options)	(2) States fortificants for at least one nutrient	States for all nutrients.
9. States fortification levels	(1) States one number only	"5.2.2.7 Especificaciones nutrimentales<br>i) Las harinas de trigo y de maíz nixtamalizado deben ser restituidas con los siguientes nutrimentos y en los niveles que se indican a continuación. [table with thiamin, riboflavin, niacin]<br>ii) Las harinas de trigo y de maíz nixtamalizado deben ser adicionadas con los siguientes nutrimentos y en los niveles que se indican a continuación. [table with folic acid, iron, zinc]"
10. States consideration of bioavailability/biological activity of fortificants	(2) States some consideration of bioavailability (mentions these or related terms)	"ii.1) Cuando se utilice sulfato ferroso como fuente de hierro, el aporte debe ser de 31,61% como ión ferroso; si se utiliza fumarato ferroso el aporte será de 31,4%<br>ii.2) Cuando se utilice óxido de zinc como fuente de zinc, el aporte del mismo corresponderá al 79,54%.<br>ii.3) Se podrán utilizar otras fuentes de hierro y zinc, siempre que la cantidad biodisponible sea, al menos, equivalente a la de las fuentes recomendadas."
11. States consideration of nutrient stability	(0) Does not state any consideration	
**COSTING**		
12. States that the cost of fortification is regulated through cost sharing schemes (between government, industry, consumers) or tax measures [to assist industry]	(0) Does not state any consideration	
13. States consideration of the financial responsibility of monitoring and enforcing fortification (schedule of fees, budget) [on the government side]	(0) Does not state any consideration	
**LABELING**		

14. Includes some sort of statement/label/logo that makes it clear that the product is fortified	(2) Includes a statement, label, or logo	"8.14.1 Las harinas de trigo y de maíz nixtamalizado preenvasadas adicionadas con ácido fólico, hierro y zinc y restituidas con vitamina B1, vitamina B2, vitamina B3, deben cumplir con lo siguiente:<br>8.14.2 Sólo podrán utilizar la siguiente denominación:<br>i) Harina de trigo adicionada con ácido fólico o folacina o folato (vitamina Bc o vitamina B9)*, zinc y hierro, restituida con Vitamina B1 (mononitrato de tiamina)*, Vitamina B2 (riboflavina)* y Vitamina B3 (niacina)*.<br>ii) Harina de maíz nixtamalizado adicionada con ácido fólico o folacina o folato (vitamina Bc o vitamina B9)*, hierro y zinc y restituida con Vitamina B1 (mononitrato de tiamina), Vitamina B2 (riboflavina)*, Vitamina B3 (niacina)*.<br>* Los términos entre paréntesis serán opcionales."
15. Provides guidance on health claims that can be made for this product (specific to micronutrients added through fortification)	(0) Does not provide	
**INTERNAL MONITORING (conducted by industry)**		
16. If samples are to be taken, describes the sampling process: for example, number of samples, amount, frequency, individual vs. composite, where samples are taken in the process, and percent considered passing	(N/A) Does not describe the sampling process	"6. Muestreo<br>El procedimiento de muestreo para los productos objeto de esta norma, debe sujetarse a lo que establece la Ley General de Salud."
17. States that industry is required to follow quality assurance/quality control in regards to fortification	(2) States requirement of QA/QC for fortification	"iv) Para efectos de control, los establecimientos que procesan harinas de trigo y de maíz nixtamalizado deberán contar con la siguiente información relativa a la restitución y adición de nutrimentos:<br>iv.1) Procedimientos escritos del proceso de restitución y adición y de los controles aplicados para garantizar su eficiencia, incluidas las medidas correctivas que se aplicarán en caso de desviaciones.<br>iv.2) Registro de las variables críticas del proceso que demuestren que se cumplen los procedimientos de restitución y adición, incluyendo reportes de las acciones correctivas aplicadas cuando se detecten desviaciones o incumplimiento de las especificaciones nutrimentales y resultados de análisis de producto terminado (autocontroles)."
18. States applicability of using qualitative testing to determine the presence or absence of a vitamin or mineral (spot tests, iChecks)	(0) Does not state	
**EXTERNAL MONITORING (conducted by government)**		
19.States requirement for external monitoring at the production site to assure compliance with standards and regulations	(0) Does not state requirement	
20. Describes protocols and systems for regulatory monitoring	(0) Does not describe	
21. If there are two or more government agencies involved in external monitoring, clarifies the roles and responsibilities between different government agencies in external monitoring	(0) Clarifies roles and responsibilities for no agencies	
22. Allows for monitoring to be conducted often enough that problems can be identified and addressed on a timely basis; specifies a timeline for inspections (i.e. once every 6 months, increasing to once every 2 months if a discrepancy is found)	(0) Does not state	
23. If samples are to be taken, describes the sampling process: for example, number of samples, amount, frequency, individual vs. composite, where samples are taken in the process, and percent considered passing	(N/A) Does not describe the sampling process	"APENDICE NORMATIVO B. MUESTREO DE CEREALES<br>Generalidades<br>1. El muestreo debe ser realizado por un técnico en muestreo con un instrumento de muestreo que permita obtener la muestra. En el caso de producto en costales, el instrumento debe llegar al centro de cada costal muestreado."
24. States applicability of using qualitative testing to determine the presence or absence of a vitamin or mineral (spot tests, iChecks)	(0) Does not state	
25. States registration is required in order to use a logo/be licensed to produce fortified foods	(0) Does not state that registration or licensing is required	
**COMMERCIAL MONITORING (conducted by government)**		
26. Provides justification for commercial monitoring at retail stores	(0) Does not provide justification for commercial monitoring	
27. Describes protocols and systems for commercial monitoring	(0) Does not describe	
28. If there are two or more government agencies involved in commercial monitoring, clarifies the roles and responsibilities between different government agencies in commercial monitoring	(0) Clarifies roles and responsibilities for no agencies	

29. Allows for monitoring to be conducted often enough that problems at the production site or import companies can be identified and addressed on a timely basis; specifies a timeline for inspections (i.e. once every 6 months) or works with production companies to correct noncompliance	(N/A) No commercial monitoring occurs	
30. If samples are to be taken, describes the sampling process: for example, number of samples, amount, frequency, individual vs. composite, where samples are taken in the process, and percent considered passing	(N/A) Does not describe the sampling process	
**IMPORT MONITORING (conducted by government)**		
31. Provides justification for import monitoring at points of entry	(0) Does not provide justification for import monitoring	
32. Describes protocols and systems for import monitoring	(0) Does not state	
33. If there are two or more government agencies involved in import monitoring, clarifies the roles and responsibilities between different government agencies in import monitoring	(0) Clarifies roles and responsibilities for no agencies	
34. If samples are to be taken, describes the sampling process: for example, number of samples, amount, frequency, individual vs. composite, where samples are taken in the process, and percent considered passing	(N/A) Does not describe the sampling process	
**ENFORCEMENT/PENALTIES**		
35. Indicates roles and responsibilities in enforcing the legislation	(0) Does not state	"APENDICE NORMATIVO B. MUESTREO DE CEREALES … 2.4.1 Las Secretarías están facultadas para efectuar el muestreo en unidades de transporte en cualquier momento y lugar."
36. States incentives to start fortification	(0) Does not state	
37. States incentives to continue fortification, including ensuring compliance	(0) Does not state	
38. States penalties to compel compliance	(0) Does not state	
39. Penalties are objectively defined (e.g. first penalty=$100, second penalty=$300)	(N/A) No penalties are stated (Answered (0) to previous question)	
40. States that enforcement is required to include feedback and support to improve performance and correct noncompliance	(0) Does not require	
**LABORATORY**		
41. References required analytical assays for nutrients (e.g. Liquid chromatography-mass spectrometry for folic acid, atomic absorption for iron and zinc.)	(2) References required assays	"5 Método de prueba para la determinacion de cadmio, plomo, fierro y zinc en productos objeto de esta norma alimentos por espectrometría de absorción atómica.<br>6 Determinación de Vitamina B1 y B2 por Cromatografía Líquida de Alta Resolución (HPLC).<br>7 Determinación de Niacina. Método microbiológico<br>8 Determinación de Acido Fólico. Método microbiológico."
42. States recognition that laboratory results are subject to several sources of variation and do not provide conclusive evidence of compliance or noncompliance	(0) Does not state recognition	
43. Focuses on the quantitative analysis of "marker" micronutrients such as iron	(0) Does not state	
**REPORTING**		
44. States how government monitoring results are shared with stakeholders	(0) Does not state how results are shared	

Nigeria Maize

Element	Scoring (use drop-down menus)	Comments (open text)
**GENERAL**		
1. States that legislation applies to at least one food vehicle fit for human consumption (types/grades to be fortified)	(2) States at least one type fit for human consumption	…requirements...for whole maize (corn) meal intended for human consumption. Whole maize meal is the food processed from fully mature, sound, whole kernels of maize, *Zea mays*
2. States the public health objective; purpose and scope of legislation	(2) States the public health objective or general purpose of legislation	The standard is reviewed to further improve on the control of micronutrient deficiency in Nigeria…and promote quality, safe, healthy, and better nutrition for consumers in line with the National Policy on Nutrition
3. References latest available science or accepted international norms and recommendations, particularly for items that may not be covered in the country's documents	(2) States the documents referenced	The Codex Alimentarius Commission…[was] referenced and such information [is] hereby duly acknowledged. -->Section on Normative References
4. Provides definitions that includes terms that are specific to fortification (e.g. fortified food, premix, fortificant, food vehicle)	(2) States at least one term related to fortification	NIS 475 defines fortificant, food vehicle, micronutrient, etc.
5. Provides repeals (if there is at least one prior document about fortification)	(2) States repeals	This edition replaces the 2010 version
6. Provides effective date or gives grace period for when fortification is to begin (e.g. effective 6 months from signing)	(0) Does not state	
**MICRONUTRIENTS/PREMIX**		
7. States nutrients required	(2) States nutrients	5.3 of NIS 822, Table 2
8. States fortificants (chemical compounds) to be used (including fortificants that are allowable as options)	(2) States fortificants for at least one nutrient	5.3 of NIS 822, Table 2
9. States fortification levels	(1) States one number only	5.3 of NIS 822, Table 2
10. States consideration of bioavailability/biological activity of fortificants	(2) States some consideration of bioavailability (mentions these or related terms)	The electrolytic iron as fortificant is replaced with NaFeEDTA that is more bioavailable.
11. States consideration of nutrient stability	(2) States consideration of nutrient stability	Shelf life: 12 months shelf life
**COSTING**		
12. States that the cost of fortification is regulated through cost sharing schemes (between government, industry, consumers) or tax measures [to assist industry]	(0) Does not state any consideration	
13. States consideration of the financial responsibility of monitoring and enforcing fortification (schedule of fees, budget) [on the government side]	(0) Does not state any consideration	
**LABELING**		
14. Includes some sort of statement/label/logo that makes it clear that the product is fortified	(2) Includes a statement, label, or logo	NIS475 says labeling must include premix composition and name of product Labelling of Vitamin A fortified foods, which include an Eye Logo for its identification,
15. Provides guidance on health claims that can be made for this product (specific to micronutrients added through fortification)	(0) Does not provide	
**INTERNAL MONITORING (conducted by industry)**		
16. If samples are to be taken, describes the sampling process: for example, number of samples, amount, frequency, individual vs. composite, where samples are taken in the process, and percent considered passing	(2) States that samples should be taken as part of internal monitoring	3.3.1a Shift composite samples are taken from the packaging line. 200g samples are taken every 30 minutes. 8 consecutive samples constitute one shift composite sample. (By QC/QA Department)
17. States that industry is required to follow quality assurance/quality control in regards to fortification	(2) States requirement of QA/QC for fortification	Figure 2.1 Internal Monitoring (factories or packers) QA/QC by the Company's Dept. of Quality Control. Outline further in later tables.
18. States applicability of using qualitative testing to determine the presence or absence of a vitamin or mineral (spot tests, iChecks)	(2) States applicability of spot test to determine presence/absence of vitamin or mineral specific to internal monitoring	Quality assurance of vitamin A and iron in fortificants 3.1 Spot density in flour samples for iron is comparable to flour standards containing the expected factory minimum, average and maximum iron levels. (completed by company's QA/QC department) 3.1b Use the iron spot test on the shift composite sample.
**EXTERNAL MONITORING (conducted by government)**		
19.States requirement for external monitoring at the production site to assure compliance with standards and regulations	(2) States requirement for external monitoring or the need for audits/inspections	The product shall sample in compliance with any of the following sampling methods:
20. Describes protocols and systems for regulatory monitoring	(2) Includes checklists or provides detailed description of regulatory monitoring procedures	Sampling at the mill shall be on not less than six (6) containers. If the sampling is from a millstream, an interval between each taking shall be 10-15 minutes. The micronutrients shall be analyzed using the test methods specified in table 2 of NIS:475:2015 Standard for Fortificant Premix of any Internationally acceptable Standard methods.
21. If there are two or more government agencies involved in external monitoring, clarifies the roles and responsibilities between different government agencies in external monitoring	(0) Clarifies roles and responsibilities for no agencies	Table 4.1: NAFDAC and SON, but doesn't give roles and responsibilities

22. Allows for monitoring to be conducted often enough that problems can be identified and addressed on a timely basis; specifies a timeline for inspections (i.e. once every 6 months, increasing to once every 2 months if a discrepancy is found)	(1) Makes mention of a timeline	Table 4
23. If samples are to be taken, describes the sampling process: for example, number of samples, amount, frequency, individual vs. composite, where samples are taken in the process, and percent considered passing	(2) States that samples should be taken as part of external monitoring	Sampling at the mill shall be on not less than six (6) containers. If the sampling is from a millstream, an interval between each taking shall be 10-15 minutes. The micronutrients shall be analyzed using the test methods specified in table 2 of NIS:475:2015 Standard for Fortificant Premix of any Internationally acceptable Standard methods. See Table 8.
24. States applicability of using qualitative testing to determine the presence or absence of a vitamin or mineral (spot tests, iChecks)	(2) States applicability of spot test to determine presence/absence of vitamin or mineral specific to external monitoring	In 2012, UNICEF and GAIN donated i-Check equipments a vitamin A test kits to NAFDAC to be used for semi quantitative spot analysis of food products (flour, vegetable oil and sugar). Both NAFDAC and the industry were trained by GAIN on the use of i-Check equipment.
25. States registration is required in order to use a logo/be licensed to produce fortified foods	(2) Describes some type of registration or licensing	Denominator: Total number of registered wheat/maize industries in the Country
**COMMERCIAL MONITORING (conducted by government)**		
26. Provides justification for commercial monitoring at retail stores	(2) Provides justification for commercial monitoring	Figure 2.1: Commercial monitoring (at distribution and retail stores)-verification of legal compliance (NAFDAC)
27. Describes protocols and systems for commercial monitoring	(0) Does not describe	
28. If there are two or more government agencies involved in commercial monitoring, clarifies the roles and responsibilities between different government agencies in commercial monitoring	(0) Clarifies roles and responsibilities for no agencies	NAFDAC (Figure 2.1)--doesn't state roles and responsibilities
29. Allows for monitoring to be conducted often enough that problems at the production site or import companies can be identified and addressed on a timely basis; specifies a timeline for inspections (i.e. once every 6 months) or works with production companies to correct noncompliance	(N/A) No commercial monitoring occurs	
30. If samples are to be taken, describes the sampling process: for example, number of samples, amount, frequency, individual vs. composite, where samples are taken in the process, and percent considered passing	(N/A) Does not describe the sampling process	
**IMPORT MONITORING (conducted by government)**		
31. Provides justification for import monitoring at points of entry	(2) Provides justification for import monitoring	Certification procedure: Certificate of conformity or inspection, COA (NAFDAC and Customs)
32. Describes protocols and systems for import monitoring	(1) Does not explicitly state, but references protocols and systems for import monitoring	Certificate of conformity from country of origin; fortified with Iron to standard imported into the country
33. If there are two or more government agencies involved in import monitoring, clarifies the roles and responsibilities between different government agencies in import monitoring	(0) Clarifies roles and responsibilities for no agencies	Certificate of conformity or inspection, COA (NAFDAC and Customs)--doesn't state roles and responsibilities
34. If samples are to be taken, describes the sampling process: for example, number of samples, amount, frequency, individual vs. composite, where samples are taken in the process, and percent considered passing	(N/A) Does not describe the sampling process	
**ENFORCEMENT/PENALTIES**		
35. Indicates roles and responsibilities in enforcing the legislation	(0) Does not state	Table 4.1 describes method/approach of data collection or calculation and lists who is involved. Also lists analysis, use, and reporting and who is acquiring data
36. States incentives to start fortification	(0) Does not state	
37. States incentives to continue fortification, including ensuring compliance	(0) Does not state	
38. States penalties to compel compliance	(0) Does not state	Enforcement is mentioned in passing, but never stated explicitly nor in relation to maize flour fortification (salt only)
39. Penalties are objectively defined (e.g. first penalty=$100, second penalty=$300)	(N/A) No penalties are stated (Answered (0) to previous question)	
40. States that enforcement is required to include feedback and support to improve performance and correct noncompliance	(0) Does not require	
**LABORATORY**		
41. References required analytical assays for nutrients (e.g. Liquid chromatography-mass spectrometry for folic acid, atomic absorption for iron and zinc.)	(2) References required assays	The micronutrients shall be analyzed using the test methods specified in Table 2 of NIS 475:2015 or any internationally acceptable Standard methods.
42. States recognition that laboratory results are subject to several sources of variation and do not provide conclusive evidence of compliance or noncompliance	(0) Does not state recognition	

43. Focuses on the quantitative analysis of "marker" micronutrients such as iron	(2) Focuses on quantitative analysis of marker micronutrient such as iron	3.1 Spot density in flour samples for iron is comparable to flour standards containing the expected factory minimum, average and maximum iron levels. 3.2 Random sample of two daily-composite samples are taken and added to monthly composite samples and analysed for quantitative determination of iron and vitamin A using i-Check.
**REPORTING**		
44. States how government monitoring results are shared with stakeholders	(2) States how results are shared with stakeholders	The food fortification results framework provides an overview of all fortification activities within the country supported through various partners, regulators and stakeholders. The results framework depicts the linkage of activities on food fortification by various key players, key targets, technical areas and expected outcomes over the lifetime of the programme. Efforts have been made to ensure the inclusion of key impact and outcome indicators as well as the documentation of process indicators (input and output). The M&E system will ensure project-wide monitoring and that all indicators listed on the results framework are tracked and reported accordingly. The measurement and analysis and reporting of the indicators would enable all stakeholders to track progress, demonstrate results and take corrective actions where necessary to improve on the delivery of appropriate food fortification to improve the health status of the population.

South Africa Maize

Element	Scoring (use drop-down menus)	Comments (open text)
**GENERAL**		
1. States that legislation applies to at least one food vehicle fit for human consumption (types/grades to be fortified)	(2) States at least one type fit for human consumption	The Minister for Health has, in terms of Section 15 (1) of the Foodstuffs, Cosmetics and Disinfectants Act, 1972 (Act No. 54 of 1972), made the regulations in the Schedule. (a) The formulation of the fortification mix for maize meal based on the micronutrient requirements specified in Annexure V, Table 4b shall be as follows: Table 2a.
2. States the public health objective; purpose and scope of legislation	(0) Does not state	
3. References latest available science or accepted international norms and recommendations, particularly for items that may not be covered in the country's documents	(2) States the documents referenced	ensure that the quality standard for diluents and fortificants, independently or mixed with a diluent shall be in accordance with the standards as determined in the latest edition of Food Chemicals Codex (FCC).
4. Provides definitions that includes terms that are specific to fortification (e.g. fortified food, premix, fortificant, food vehicle)	(2) States at least one term related to fortification	In these regulations any word or expression defined in the Act and not defined herein bears the same meaning as in the Act and unless the context otherwise indicates-
5. Provides repeals (if there is at least one prior document about fortification)	(2) States repeals	13. The Regulations on the Enrichment of Maize Meal, promulgated under Government notice No. R 2839 of 21 December 1979 are hereby repealed.
6. Provides effective date or gives grace period for when fortification is to begin (e.g. effective 6 months from signing)	(2) States effective date or grace period for when fortification is to begin (e.g. effective 6 months from signing)	14. These regulations shall come into operation 6 months after the date of final publication.
**MICRONUTRIENTS/PREMIX**		
7. States nutrients required	(2) States nutrients	Special Provisions 9a, Table 1
8. States fortificants (chemical compounds) to be used (including fortificants that are allowable as options)	(2) States fortificants for at least one nutrient	Special Provisions 9a, Table 1
9. States fortification levels	(2) States a range or number with +/-	Special Provisions 9a, Table 1. No range given, but tolerances are detailed in Annexure VI
10. States consideration of bioavailability/biological activity of fortificants	(0) Does not state any consideration	
11. States consideration of nutrient stability	(2) States consideration of nutrient stability	Is vitamin A compound stable as per specification? (Attach findings of<br>internal vitamin A stability trials for each source as indicated in<br>question 17)*<br>* For more information on the vitamin A stability specification, contact the<br>Directorate: Nutrition, Department of Health."
**COSTING**		
12. States that the cost of fortification is regulated through cost sharing schemes (between government, industry, consumers) or tax measures [to assist industry]	(0) Does not state any consideration	
13. States consideration of the financial responsibility of monitoring and enforcing fortification (schedule of fees, budget) [on the government side]	(0) Does not state any consideration	
**LABELING**		
14. Includes some sort of statement/label/logo that makes it clear that the product is fortified	(2) Includes a statement, label, or logo	(b) the claim “Fortified for better health” and the official fortification logo to that effect as indicated in Annexure VII are reserved only for food vehicles, that have been identified in these regulations and may be displayed on the label or in an advertising material;<br>(c) any person who uses the official logo referred to in Annexure VII on labels or in advertising material for foodstuffs other than in accordance with these regulations or any other regulations made in terms of the Act, shall be guilty of an offence.
15. Provides guidance on health claims that can be made for this product (specific to micronutrients added through fortification)	(2) Provides guidance on health claims specific to micronutrients added through fortification	In addition to the Regulations Governing the Advertising and Labelling of Foodstuffs made under the Act, all food vehicles shall be labelled as follows:<br>(a) the claim “enriched with” or “enriched” may only be used in addition to the word “fortified” on one label in cases where a micronutrient other than the specified fortificants is added to a food vehicle or in cases where at least 15% more than the prescribed amounts of fortificants are added to a food vehicle;<br>(b) the claim “Fortified for better health” and the official fortification logo to that effect as indicated in Annexure VII are reserved only for food vehicles, that have been identified in these regulations and may be displayed on the label or in an advertising material;
**INTERNAL MONITORING (conducted by industry)**		

16. If samples are to be taken, describes the sampling process: for example, number of samples, amount, frequency, individual vs. composite, where samples are taken in the process, and percent considered passing	(2) States that samples should be taken as part of internal monitoring	submit one 500 g sample of a fortification mix every six months for each food vehicle to a laboratory that has accreditation for the methods of analysis as indicated by the fortification mix manufacturer, importer or supplier from SANAS or another international accreditation body; keep the analysis report on record and submit a copy of the report to the Director-General; and
17. States that industry is required to follow quality assurance/quality control in regards to fortification	(2) States requirement of QA/QC for fortification	Annexure II implies that manufacturers or suppliers of fortification mixes are responsible for the listed quality control principles 4. ensure that all critical stages of the manufacturing process are monitored to ensure that the correct dosage levels are maintained through the following measures:<br>(a) checking of fortification mix feeders at least once a day to ensure that they are delivering the correct dosage levels;<br>(b) performing visual checks at least twice per shift to ensure that fortification mixes are being used and that no blockages have occurred and keeping record of this;<br>(c) performing two-hourly spot checks to ensure that the product has been dosed correctly by determining one of the components of a fortification mix according to the appropriate analytical method.
18. States applicability of using qualitative testing to determine the presence or absence of a vitamin or mineral (spot tests, iChecks)	(2) States applicability of spot test to determine presence/absence of vitamin or mineral specific to internal monitoring	(internal) performing two-hourly spot checks to ensure that the product has been dosed correctly by determining one of the components of a fortification mix according to the appropriate analytical method.
**EXTERNAL MONITORING (conducted by government)**		
19.States requirement for external monitoring at the production site to assure compliance with standards and regulations	(2) States requirement for external monitoring or the need for audits/inspections	At least two inspection audits per year, including the taking of samples for laboratory analysis, in respect of all registered fortification mix manufacturers, suppliers and importers. to monitor compliance with the Regulations relating to the Fortification of Certain Foodstuffs shall be conducted at their premises and the manufacturers, suppliers or importers themselves shall bear the costs of such audits and analysis.
20. Describes protocols and systems for regulatory monitoring	(2) Includes checklists or provides detailed description of regulatory monitoring procedures	At least two inspection audits per year, including the taking of samples for laboratory analysis, in respect of all registered fortification mix manufacturers, suppliers and importers. to monitor compliance with the Regulations relating to the Fortification of Certain Foodstuffs shall be conducted at their premises and the manufacturers, suppliers or importers themselves shall bear the costs of such audits and analysis.
21. If there are two or more government agencies involved in external monitoring, clarifies the roles and responsibilities between different government agencies in external monitoring	(1) Clarifies roles and responsibilities for one agency	Doc 39613 - 8(1) - inspectors should take samples to apply regulations
22. Allows for monitoring to be conducted often enough that problems can be identified and addressed on a timely basis; specifies a timeline for inspections (i.e. once every 6 months, increasing to once every 2 months if a discrepancy is found)	(1) Makes mention of a timeline	At least two inspection audits per year, including the taking of samples for laboratory analysis, in respect of all registered fortification mix manufacturers, suppliers and importers. to monitor compliance with the Regulations relating to the Fortification of Certain Foodstuffs shall be conducted at their premises and the manufacturers, suppliers or importers themselves shall bear the costs of such audits and analysis.
23. If samples are to be taken, describes the sampling process: for example, number of samples, amount, frequency, individual vs. composite, where samples are taken in the process, and percent considered passing	(2) States that samples should be taken as part of external monitoring	A sample of a fortified food vehicle, taken by an inspector in terms of the Act, shall be analysed for the amounts of nicotinamide / niacinamide, or riboflavin and retinol / vitamin A palmitate, and the results of such a sample shall be considered as representative of the standards prescribed by these Regulations in Table 3 and Table 4.
24. States applicability of using qualitative testing to determine the presence or absence of a vitamin or mineral (spot tests, iChecks)	(0) Does not state	
25. States registration is required in order to use a logo/be licensed to produce fortified foods	(2) Describes some type of registration or licensing	South Africa requires premix producers to be registered and then keep records of their distribution to mills
**COMMERCIAL MONITORING (conducted by government)**		
26. Provides justification for commercial monitoring at retail stores	(0) Does not provide justification for commercial monitoring	
27. Describes protocols and systems for commercial monitoring	(0) Does not describe	
28. If there are two or more government agencies involved in commercial monitoring, clarifies the roles and responsibilities between different government agencies in commercial monitoring	(0) Clarifies roles and responsibilities for no agencies	

29. Allows for monitoring to be conducted often enough that problems at the production site or import companies can be identified and addressed on a timely basis; specifies a timeline for inspections (i.e. once every 6 months) or works with production companies to correct noncompliance	(0) Does not state	
30. If samples are to be taken, describes the sampling process: for example, number of samples, amount, frequency, individual vs. composite, where samples are taken in the process, and percent considered passing	(N/A) Does not describe the sampling process	
**IMPORT MONITORING (conducted by government)**		
31. Provides justification for import monitoring at points of entry	(2) Provides justification for import monitoring	Any person who manufactures, imports, or sells foodstuffs identified as food vehicles which have not been fortified in accordance with these regulations, including the requirements specified in Annexure I, shall be guilty of an offence.
32. Describes protocols and systems for import monitoring	(1) Does not explicitly state, but references protocols and systems for import monitoring	Registered manufacturers, importers or suppliers of fortification mixes shall issue a certificate of compliance as indicated in Annexure IV.
33. If there are two or more government agencies involved in import monitoring, clarifies the roles and responsibilities between different government agencies in import monitoring	(2) Clarifies roles and responsibilities for more than one agency	See Act 54, Reg 14 - if suspected not in compliance, Commissioner may allow the items to pass from his control to another location approved by the DG and detailed for examination or analysis until the result is known. Commissioner should not allow anything to pass from his control unless the importer has furnished the DG with a guarantee that he will pay if he fails to comply. If analysis determines that it can't be imported, it is confiscated, destroyed, returned to place or origin, or otherwise disposed of
34. If samples are to be taken, describes the sampling process: for example, number of samples, amount, frequency, individual vs. composite, where samples are taken in the process, and percent considered passing	(N/A) Does not describe the sampling process	
**ENFORCEMENT/PENALTIES**		
35. Indicates roles and responsibilities in enforcing the legislation	(2) States the role and responsibilities of government in enforcement	Sections 10 and 11 of Act 54
36. States incentives to start fortification	(0) Does not state	
37. States incentives to continue fortification, including ensuring compliance	(0) Does not state	
38. States penalties to compel compliance	(2) States any penalties	Section 18 states penalties
39. Penalties are objectively defined (e.g. first penalty=$100, second penalty=$300)	(2) Penalties are objectively laid out in the document (e.g. first penalty=$100, second penalty=$300)	Yes, details first, second, third convictions, and gives limit of fine or maximum imprisonment
40. States that enforcement is required to include feedback and support to improve performance and correct noncompliance	(0) Does not require	
**LABORATORY**		
41. References required analytical assays for nutrients (e.g. Liquid chromatography-mass spectrometry for folic acid, atomic absorption for iron and zinc.)	(2) References required assays	Only accredited analytical methods for which an original certificate or certified copy from SANAS or another internationally accreditation body has been obtained and which are attached to the application (Annexure III) will be accepted.
42. States recognition that laboratory results are subject to several sources of variation and do not provide conclusive evidence of compliance or noncompliance	(0) Does not state recognition	
43. Focuses on the quantitative analysis of "marker" micronutrients such as iron	(2) Focuses on quantitative analysis of marker micronutrient such as iron	A sample of a fortified food vehicle, taken by an inspector in terms of the Act, shall be analysed for the amounts of nicotinamide / niacinamide, or riboflavin and retinol / vitamin A palmitate, and the results of such a sample shall be considered as representative of the standards prescribed by these Regulations in Table 3 and Table 4.
**REPORTING**		
44. States how government monitoring results are shared with stakeholders	(0) Does not state how results are shared	

Tanzania Maize

Element	Scoring (use drop-down menus)	Comments (open text)
**GENERAL**		
1. States that legislation applies to at least one food vehicle fit for human consumption (types/grades to be fortified)	(2) States at least one type fit for human consumption	Standard is specific to maize flour
2. States the public health objective; purpose and scope of legislation	(2) States the public health objective or general purpose of legislation	Maize flour is one of the important food products in the country. The preparation of this standard is meant to ensure that maize flour produced and /or traded in the country is safe and of quality fit for human consumption. Micronutrient malnutrition is a problem of public health significance in Tanzania affecting a large number of children and women of child bearing age, but it is debilitating for all ages and for the national economy as well.
3. References latest available science or accepted international norms and recommendations, particularly for items that may not be covered in the country's documents	(2) States the documents referenced	In the preparation of this Tanzania Standard assistance was derived from: KS – 168:2007 – Specification for milled maize product, published by Kenya Bureau of Standards.
4. Provides definitions that includes terms that are specific to fortification (e.g. fortified food, premix, fortificant, food vehicle)	(2) States at least one term related to fortification	Section 3 on terminology
5. Provides repeals (if there is at least one prior document about fortification)	(2) States repeals	This second edition cancels and replaces the first edition (TZS 439:2006) which has been technically revised.
6. Provides effective date or gives grace period for when fortification is to begin (e.g. effective 6 months from signing)	(2) States effective date or grace period for when fortification is to begin (e.g. effective 6 months from signing)	DEAS 768 - Within 6 months of a declaration of an EA Standard, Partner States shall adopt the approved text without deviation and withdraw any existing national standard
**MICRONUTRIENTS/PREMIX**		
7. States nutrients required	(2) States nutrients	Table 2
8. States fortificants (chemical compounds) to be used (including fortificants that are allowable as options)	(2) States fortificants for at least one nutrient	Table 2
9. States fortification levels	(2) States a range or number with +/-	Table 2, gives range around factory levels
10. States consideration of bioavailability/biological activity of fortificants	(0) Does not state any consideration	
11. States consideration of nutrient stability	(2) States consideration of nutrient stability	Stability is defined as the di fference in time, to the nearest 0.5 min, between the point at which the top of the curve fi rst intercepts the 500 FU line and the point at which the top of the curve leaves the 500 FU line. This value, in general, gives some indication of the tolerance of the flour to mixing.
**COSTING**		
12. States that the cost of fortification is regulated through cost sharing schemes (between government, industry, consumers) or tax measures [to assist industry]	(0) Does not state any consideration	
13. States consideration of the financial responsibility of monitoring and enforcing fortification (schedule of fees, budget) [on the government side]	(2) Shows consideration that monitoring costs money	The monitoring manual states very clearly that costs for monitoring should be planned for, but it doesn't necessarily indicate that adequate funds are dedicated to monitoring
**LABELING**		
14. Includes some sort of statement/label/logo that makes it clear that the product is fortified	(2) Includes a statement, label, or logo	9 Labeling In addition to the requirements given in TZS 538 (see clause 2), each bag shall be labeled to contain the under mentioned information: o) Explicit fortification logo in case of fortified maize flour.
15. Provides guidance on health claims that can be made for this product (specific to micronutrients added through fortification)	(2) Provides guidance on health claims specific to micronutrients added through fortification	7.3 Nutrition and health claims Fortified maize flour may have claims on the importance of vitamin A in nutrition and health. Such claims when declared should be consistent with national legislation or standards on nutrition and health claims. Where no national standards or legislation exists such claims should conform to Codex Alimentarius Guidelines for Claims, TZS 1340 (see clause 2) and guidelines for use of nutrition and health claims (CAC/GL 23-1997).
**INTERNAL MONITORING (conducted by industry)**		
16. If samples are to be taken, describes the sampling process: for example, number of samples, amount, frequency, individual vs. composite, where samples are taken in the process, and percent considered passing	(2) States that samples should be taken as part of internal monitoring	3.2.2, 3.2.3 (3 daily composite samples; 1 sample from production, 8 samples from storage warehouse (combined)). Combine all five composite samples and divide into three portions of 500g
17. States that industry is required to follow quality assurance/quality control in regards to fortification	(2) States requirement of QA/QC for fortification	Manufacturer of a fortified food shall develop, maintain and routinely follow procedures for safety and quality assurance throughout the manufacturing process to ensure that the final product complies with these regulations and any other regulations made under the Act.

18. States applicability of using qualitative testing to determine the presence or absence of a vitamin or mineral (spot tests, iChecks)	(2) States applicability of spot test to determine presence/absence of vitamin or mineral specific to internal monitoring	Internal monitoring manual - Section 3.3.2 on spot tests
**EXTERNAL MONITORING (conducted by government)**		
19.States requirement for external monitoring at the production site to assure compliance with standards and regulations	(2) States requirement for external monitoring or the need for audits/inspections	Technical auditing and inspection activities are carried out at manufacturing facilities as part of the enforcement activities performed by the regulatory Authority in order to ensure that fortified foods meets the nutrient quality and safety specifications established in regulations. During the technical audits, the performance of quality assurance and quality control activities conducted by the producer is examined. The conformity of the fortified food with technical specifications is determined through sampling and chemical analysis of fortified food samples collected from the factory. Samples of premix/fortification may also be taken to verify the validity of the Certificate of Analysis (COA) provided by the supplier.
20. Describes protocols and systems for regulatory monitoring	(2) Includes checklists or provides detailed description of regulatory monitoring procedures	Checklist for inspection of maize/wheat flour manufacturing facilities
21. If there are two or more government agencies involved in external monitoring, clarifies the roles and responsibilities between different government agencies in external monitoring	(N/A) Only one government agency involved	The people directly responsible for achievement of these objectives are the Authority Inspectors, who should pass on the results of the inspection to their supervisor of inspection activities. The Supervisor is responsible for preparing the reports to the factories and reporting every six months to the Head of the regulatory Authority.
22. Allows for monitoring to be conducted often enough that problems can be identified and addressed on a timely basis; specifies a timeline for inspections (i.e. once every 6 months, increasing to once every 2 months if a discrepancy is found)	(2) Describes frequency and how it is responsive to the needs of industry or the stage of fortification implementation	plan at least two yearly inspection to manufacturing facilities. The Authority should then conduct a comprehensive follow-up inspection inspection. , inspectioninspection to assess implementation of corrective actions. The follow up inspectioninspectioninspection, which may take place ahead of schedule if the identified limitations are considered serious
23. If samples are to be taken, describes the sampling process: for example, number of samples, amount, frequency, individual vs. composite, where samples are taken in the process, and percent considered passing	(2) States that samples should be taken as part of external monitoring	Take a 50g sample of the fortificant/premix that is being used for fortification at the factory during the time of inspection. Label it with the name of the mill, name of the manufacturer, micronutrient content indicated, and date of sample collection. 500g of all other samples
24. States applicability of using qualitative testing to determine the presence or absence of a vitamin or mineral (spot tests, iChecks)	(2) States applicability of spot test to determine presence/absence of vitamin or mineral specific to external monitoring	Collect samples based on brand names and perform spot test and record results in form F8/TFDA/DFS/FI&E/GL/001 of these guidelines.
25. States registration is required in order to use a logo/be licensed to produce fortified foods	(2) Describes some type of registration or licensing	List of approved brands per food. This list should be updated every three months or more frequent if needed. Use Second Schedule to apply for license
**COMMERCIAL MONITORING (conducted by government)**		
26. Provides justification for commercial monitoring at retail stores	(2) Provides justification for commercial monitoring	Inspection of food outlets is conducted in order to verify legal compliance of fortified foods sold in retail supermarkets, markets, grocery stores, and wholesale stores. This monitoring allows for the detection in the market of brands that are not approved by the Authority or do not comply with local fortification regulations. It also helps to confirm whether brands that have previously been inspected in factories and importation sites are indeed fulfilling the requirements as claimed by inspectors during the external monitoring process.
27. Describes protocols and systems for commercial monitoring	(2) Includes checklists or provides detailed description of regulatory monitoring procedures	Take samples of approved fortified foods and conduct spot test for iron and where necessary, take samples for quantitative analysis at TFDA head quarters. They should fill in the form F5/TFDA/DFS/FI&E/GL/001 of these guidelines
28. If there are two or more government agencies involved in commercial monitoring, clarifies the roles and responsibilities between different government agencies in commercial monitoring	(2) Clarifies roles and responsibilities for more than one agency	Inspection of food outlets is the responsibility of the regulatory authority, in cooperation with local authorities. Monitoring at this level should also involve local authority inspectors in carrying out audit and inspection inspection and taking of samples in their respective areas. Authority is responsible for preparing the sampling Plan and providing the technical training to carry out the inspections. This part of guidelines describes the procedures for carrying out the inspection inspection at any retail store selling fortified foods. It also describes those responsible for each stage. As any enforcement procedure carried out by a governmental body, warning and legal actions should be given when non-compliances are observed.

29. Allows for monitoring to be conducted often enough that problems at the production site or import companies can be identified and addressed on a timely basis; specifies a timeline for inspections (i.e. once every 6 months) or works with production companies to correct noncompliance	(1) Makes mention of a timeline	Does this mean monitoring happens quarterly? "Results of commercial monitoring activities should be consolidated in reports to be issued on quarterly basis. The reports assist defining the degree of success in fulfilling the fortification goals and spells out obstacles that need to be overcome and actions to be taken. It is further recommended that an annual report be prepared and published where data is presented graphically to describe the status of the fortification program in the country, along with information from other general food control or surveillance activities."
30. If samples are to be taken, describes the sampling process: for example, number of samples, amount, frequency, individual vs. composite, where samples are taken in the process, and percent considered passing	(2) States that samples should be taken as part of commercial monitoring	Choose a sealed packaged of about 500gm or 500ml of each brand of each fortified food in the store. If the food is not available in such quantities, take the nearest larger retail-size presentation. If packages are much smaller, collect sufficient packages to make up the specified weight; (e.g. 2 packages of 250 gm).
**IMPORT MONITORING (conducted by government)**		
31. Provides justification for import monitoring at points of entry	(2) Provides justification for import monitoring	The purpose of monitoring fortificants and fortified foods prior to authorization for entry is:-<br>a) To ensure that the imported products are accompanied by adequate documentation to certify that national standards and regulations are being fulfilled.<br>b) To confirm that the food complies with fortification requirements based on the presence of one or more key micronutrients in the imported fortified food.
32. Describes protocols and systems for import monitoring	(2) Includes checklists or detailed description of import monitoring procedures	4.3.3 Taking decisions to authorize<br>a) All samples should test positive for the indicator micronutrient.<br>b) If samples fail the qualitative test or fail to comply in terms of proper documentation and labeling requirements, the affected brand should not be allowed to enter the country.<br>c) If documentation is correct, and samples show the presence of the key micronutrient, authorize importation.
33. If there are two or more government agencies involved in import monitoring, clarifies the roles and responsibilities between different government agencies in import monitoring	(N/A) Only one government agency involved	Supervisor of food inspection activities at the port of entry should ensure that the following tasks are performed; collection of samples, testing and reviewing documentation before the food can be allowed to enter the country
34. If samples are to be taken, describes the sampling process: for example, number of samples, amount, frequency, individual vs. composite, where samples are taken in the process, and percent considered passing	(2) States that samples should be taken as part of import monitoring	4.3.3 Taking decisions to authorize<br>a) All samples should test positive for the indicator micronutrient.<br>b) If samples fail the qualitative test or fail to comply in terms of proper documentation and labeling requirements, the affected brand should not be allowed to enter the country.<br>c) If documentation is correct, and samples show the presence of the key micronutrient, authorize importation.
**ENFORCEMENT/PENALTIES**		
35. Indicates roles and responsibilities in enforcing the legislation	(2) States the role and responsibilities of government in enforcement	When a brand does not meet the minimum legal requirements (micronutrient content, labeling and packaging) as depicted in the fortification regulations, a warning letter shall be sent to the factory, packaging plant or importer responsible for the brand. As any enforcement procedure carried out by a governmental body, warning and legal actions should be given when non-compliances are observed.
36. States incentives to start fortification	(0) Does not state	
37. States incentives to continue fortification, including ensuring compliance	(0) Does not state	
38. States penalties to compel compliance	(2) States any penalties	Any person who contravenes any provision of these regulations commits an offence under the Act. (points 11 and 12 in the Food Act) (fines, lose license)
39. Penalties are objectively defined (e.g. first penalty=$100, second penalty=$300)	(0) Penalties are not objectively laid out	If the factory is found to have not taken any action to solve the problem or if there is proof that the noncompliance is intentional, action should be taken against the factory and this could vary from a written warning to legal action such as a fine and other actions as deemed necessary.
40. States that enforcement is required to include feedback and support to improve performance and correct noncompliance	(2) Requires any feedback/support to improve performance	When a minor non-compliance is found, technical advice should be provided to the production manager on areas that need improvement and follow up with more frequent inspections.
**LABORATORY**		
41. References required analytical assays for nutrients (e.g. Liquid chromatography-mass spectrometry for folic acid, atomic absorption for iron and zinc.)	(2) References required assays	DEAS 768 - conducted using any ECSA or other internationally recognized test methods - manuals for testing at www.a2zproject.org

		d) When results are received from the laboratory, compare them with producer's records. Remember that the results from the producer were obtained using semi quantitative methods while the laboratory uses quantitative method. Therefore some variation between the two results is expected. However, if results differ greatly, for example, iron level reported quantitatively was less than the legal minimum and the daily estimated average was greater than 20 mg/kg, the cause of such discrepancy should be investigated. e) Analyze the results and complete the report. The analytical results for ALL five samples should be randomly distributed within acceptable range, irrespective of whether they are samples from production of the day, from storage warehouse or from composite samples of the month. Any significant discrepancy between samples collected during inspection and those stored as daily composite samples should be a cause for concern and should be investigated during next inspection inspection. Prepare letters to advise the inspected factories of the problem.
42. States recognition that laboratory results are subject to several sources of variation and do not provide conclusive evidence of compliance or noncompliance	(2) States recognition that lab results are subject to variation	
43. Focuses on the quantitative analysis of "marker" micronutrients such as iron	(2) Focuses on quantitative analysis of marker micronutrient such as iron	4.1.2 Qualitative methods Iron (external lab)<br>4.1.3 Quantitative method Vit. A (external lab)
**REPORTING**		
44. States how government monitoring results are shared with stakeholders	(0) Does not state how results are shared	

Uganda Maize

Element	Scoring (use drop-down menus)	Comments (open text)
**GENERAL**		
1. States that legislation applies to at least one food vehicle fit for human consumption (types/grades to be fortified)	(2) States at least one type fit for human consumption	ECSA Standard - Part 1
2. States the public health objective; purpose and scope of legislation	(2) States the public health objective or general purpose of legislation	ECSA Standard - Intro, para 2/3
3. References latest available science or accepted international norms and recommendations, particularly for items that may not be covered in the country's documents	(2) States the documents referenced	ECSA Standard - Part 5.2 - shall be stable compounds conforming to specifications in…
4. Provides definitions that includes terms that are specific to fortification (e.g. fortified food, premix, fortificant, food vehicle)	(2) States at least one term related to fortification	ECSA Standard - Part 3
5. Provides repeals (if there is at least one prior document about fortification)	(2) States repeals	ECSA Standard - Foreword - Repeal existing national standards
6. Provides effective date or gives grace period for when fortification is to begin (e.g. effective 6 months from signing)	(0) Does not state	
**MICRONUTRIENTS/PREMIX**		
7. States nutrients required	(2) States nutrients	ECSA Standard - Table 2
8. States fortificants (chemical compounds) to be used (including fortificants that are allowable as options)	(2) States fortificants for at least one nutrient	ECSA Standard - Table 2
9. States fortification levels	(2) States a range or number with +/-	ECSA Standard - Table 2
10. States consideration of bioavailability/biological activity of fortificants	(0) Does not state any consideration	
11. States consideration of nutrient stability	(2) States consideration of nutrient stability	ECSA Standard - Part 5.4
**COSTING**		
12. States that the cost of fortification is regulated through cost sharing schemes (between government, industry, consumers) or tax measures [to assist industry]	(0) Does not state any consideration	
13. States consideration of the financial responsibility of monitoring and enforcing fortification (schedule of fees, budget) [on the government side]	(2) Shows consideration that monitoring costs money	2005 Regs - Second Schedule f - manufacturers/importers are to bear the cost of analysis; Manual - Planning/Budget/Schedule section for each type of monitoring gives consideration for budgeting for inspections
**LABELING**		
14. Includes some sort of statement/label/logo that makes it clear that the product is fortified	(2) Includes a statement, label, or logo	ECSA Standard - Part 10.1
15. Provides guidance on health claims that can be made for this product (specific to micronutrients added through fortification)	(2) Provides guidance on health claims specific to micronutrients added through fortification	ECSA Standard - Part 10.3
**INTERNAL MONITORING (conducted by industry)**		
16. If samples are to be taken, describes the sampling process: for example, number of samples, amount, frequency, individual vs. composite, where samples are taken in the process, and percent considered passing	(1) States that samples should be taken (generally)	ECSA Standard - Part 11 - Refers to ISO 13690
17. States that industry is required to follow quality assurance/quality control in regards to fortification	(2) States requirement of QA/QC for fortification	ECSA Standard - As per EAS 39 - Code of Practice for Hygiene in Food Industry; 2005 Regs - 9(4) - manufacturer/importer shall establish QA system
18. States applicability of using qualitative testing to determine the presence or absence of a vitamin or mineral (spot tests, iChecks)	(2) States applicability of spot test to determine presence/absence of vitamin or mineral specific to internal monitoring	2005 Regs - First Schedule d(iii) - regular spot checks to ensure product has been dosed correctly
**EXTERNAL MONITORING (conducted by government)**		
19.States requirement for external monitoring at the production site to assure compliance with standards and regulations	(2) States requirement for external monitoring or the need for audits/inspections	Manual - Scope
20. Describes protocols and systems for regulatory monitoring	(2) Includes checklists or provides detailed description of regulatory monitoring procedures	Manual - very clear throughout
21. If there are two or more government agencies involved in external monitoring, clarifies the roles and responsibilities between different government agencies in external monitoring	(2) Clarifies roles and responsibilities for more than one agency	2005 Regs - Ministry of Health and NDA, Section 9; Manual - UNBS
22. Allows for monitoring to be conducted often enough that problems can be identified and addressed on a timely basis; specifies a timeline for inspections (i.e. once every 6 months, increasing to once every 2 months if a discrepancy is found)	(2) Describes frequency and how it is responsive to the needs of industry or the stage of fortification implementation	Manual - page 4/5 - 2x/yr, more frequent with identified issues
23. If samples are to be taken, describes the sampling process: for example, number of samples, amount, frequency, individual vs. composite, where samples are taken in the process, and percent considered passing	(2) States that samples should be taken as part of external monitoring	Manual - page 8 for maize, 11 for wheat
24. States applicability of using qualitative testing to determine the presence or absence of a vitamin or mineral (spot tests, iChecks)	(2) States applicability of spot test to determine presence/absence of vitamin or mineral specific to external monitoring	Manual - page 8 for maize, 11 for wheat - estimated iron level from daily composite samples
25. States registration is required in order to use a logo/be licensed to produce fortified foods	(0) Does not state that registration or licensing is required	

**COMMERCIAL MONITORING (conducted by government)**		
26. Provides justification for commercial monitoring at retail stores	(2) Provides justification for commercial monitoring	Manual - Scope
27. Describes protocols and systems for commercial monitoring	(2) Includes checklists or provides detailed description of regulatory monitoring procedures	Manual - Part III
28. If there are two or more government agencies involved in commercial monitoring, clarifies the roles and responsibilities between different government agencies in commercial monitoring	(2) Clarifies roles and responsibilities for more than one agency	2005 Regs - Ministry of Health and NDA, Section 9; Manual - UNBS
29. Allows for monitoring to be conducted often enough that problems at the production site or import companies can be identified and addressed on a timely basis; specifies a timeline for inspections (i.e. once every 6 months) or works with production companies to correct noncompliance	(2) Describes frequency and how it is responsive to the needs of industry or the stage of fortification implementation	Manual - Page 34, 1.3.1 - yearly visit to each region. Frequency and intensity of sampling depending on population, amount of food sold, risk factors - close to borders with non-fortifying countries
30. If samples are to be taken, describes the sampling process: for example, number of samples, amount, frequency, individual vs. composite, where samples are taken in the process, and percent considered passing	(2) States that samples should be taken as part of commercial monitoring	ECSA Standard - Part 11 - Refers to ISO 13690 - Manual, page 36 - 2.3 procedure for inspections, sampling, and analysis
**IMPORT MONITORING (conducted by government)**		
31. Provides justification for import monitoring at points of entry	(2) Provides justification for import monitoring	Manual - Scope
32. Describes protocols and systems for import monitoring	(2) Includes checklists or detailed description of import monitoring procedures	2005 Regs - Second Schedule - responsibility of importer - Manual, section II
33. If there are two or more government agencies involved in import monitoring, clarifies the roles and responsibilities between different government agencies in import monitoring	(2) Clarifies roles and responsibilities for more than one agency	2005 Regs - Ministry of Health and NDA, Section 9; Manual - UNBS
34. If samples are to be taken, describes the sampling process: for example, number of samples, amount, frequency, individual vs. composite, where samples are taken in the process, and percent considered passing	(2) States that samples should be taken as part of import monitoring	2005 Regs - Second Schedule d - samples every 6 months or when required - Manual - page 23 - 1.3.4 preparation of monthly composite samples; Page 28 - 3.4 qualitative testing for iron
**ENFORCEMENT/PENALTIES**		
35. Indicates roles and responsibilities in enforcing the legislation	(2) States the role and responsibilities of government in enforcement	2005 Reg - 4(2) - Minister monitors the implementation and enforcement
36. States incentives to start fortification	(0) Does not state	
37. States incentives to continue fortification, including ensuring compliance	(0) Does not state	
38. States penalties to compel compliance	(2) States any penalties	2005 Regs - 9(3) - impound, stop manufacture, etc.; 11 - imprisonment, destroy products
39. Penalties are objectively defined (e.g. first penalty=$100, second penalty=$300)	(0) Penalties are not objectively laid out	
40. States that enforcement is required to include feedback and support to improve performance and correct noncompliance	(2) Requires any feedback/support to improve performance	Manual - page 5 - providing technical support to factories to correct iddues
**LABORATORY**		
41. References required analytical assays for nutrients (e.g. Liquid chromatography-mass spectrometry for folic acid, atomic absorption for iron and zinc.)	(2) References required assays	ECSA Standard - Part 12 - ECSA or other internationally recognized test methods
42. States recognition that laboratory results are subject to several sources of variation and do not provide conclusive evidence of compliance or noncompliance	(2) States recognition that lab results are subject to variation	Manual - Page 9 (section b) for Maize, page 12 (section b) for wheat
43. Focuses on the quantitative analysis of "marker" micronutrients such as iron	(2) Focuses on quantitative analysis of marker micronutrient such as iron	2005 Regs - First Schedule d(iii) - by determining one of the components of a fortificant…; Manual - Page 22, section 1.3.2 - Confirming the presence of indicator micronutrients - Vitamin A and Iron mentioned in Manual, page 8 and 11 for industry monitoring
**REPORTING**		
44. States how government monitoring results are shared with stakeholders	(2) States how results are shared with stakeholders	Manual page 3 - results should be consolidated minimum 2x/yr, annual report with data presented graphically…

United States Maize

Element	Scoring (use drop-down menus)	Comments (open text)
**GENERAL**		
1. States that legislation applies to at least one food vehicle fit for human consumption (types/grades to be fortified)	(2) States at least one type fit for human consumption	(a) Enriched corn meals are the foods, each of which conforms to the definition and standard of identity prescribed for a kind of corn meal by 137.250, 137.255, 137.265, 137.270, 137.275, 137.280, 137.285, and 137.290,
2. States the public health objective; purpose and scope of legislation	(2) States the public health objective or general purpose of legislation	This action is being taken to help women of childbearing age to reduce their risk of having a pregnancy affected with spina bifida or other neural tube defects (NTD's) and to comply with the recommendation of the U.S. Public Health Service (PHS) that they consume at least 0.4 mg (400 mg) of folic acid daily.
3. References latest available science or accepted international norms and recommendations, particularly for items that may not be covered in the country's documents	(2) States the documents referenced	The Food and Drug Administration (FDA) is amending the food additive regulations to provide for the safe use of folic acid in foods that are the subject of a standard of identity that requires the addition of folic acid; to provide for its addition to breakfast cereals on a per serving basis; to permit its use in infant formulas, medical foods, and foods for special dietary use; and to incorporate specifications for folic acid consistent with those in the Food Chemicals Codex.
4. Provides definitions that includes terms that are specific to fortification (e.g. fortified food, premix, fortificant, food vehicle)	(2) States at least one term related to fortification	The term "essential nutrient" under the fortification policy refers to the vitamins and minerals assigned Reference Daily Intakes (RDIs) listed in 21 CFR 101.9(c)(8)(iv), as well as protein and potassium that have daily reference values (DRVs) (21 CFR 101.9(c)(9) or 21 CFR 104.20(d)(3)).
5. Provides repeals (if there is at least one prior document about fortification)	(2) States repeals	(a) Enriched corn meals are the foods, each of which conforms to the definition and standard of identity prescribed for a kind of corn meal by 137.250, 137.255, 137.265, 137.270, 137.275, 137.280, 137.285, and 137.290, except that:
6. Provides effective date or gives grace period for when fortification is to begin (e.g. effective 6 months from signing)	(2) States effective date or grace period for when fortification is to begin (e.g. effective 6 months from signing)	EFFECTIVE DATE: January 1, 1998. Many comments expressed concern over the statement in the standards of identity proposal that the final rule would become effective 1 year after publication.
**MICRONUTRIENTS/PREMIX**		
7. States nutrients required	(2) States nutrients	(1) It contains in each pound not less than 2.0 milligrams (mg) and not more than 3.0 mg of thiamin, not less than 1.2 mg and not more than 1.8 mg of riboflavin, not less than 16 mg and not more than 24 mg of niacin or niacinamide, not less than 0.7 mg and not more than 1.0 mg of folic acid, and not less than 13 mg and not more than 26 mg of iron (Fe); (2) It may contain in each pound not less than 250 U.S.P. units and not more than 1,000 U.S.P. units of vitamin D; and (3) It may contain in each pound not less than 500 milligrams and not more than 750 milligrams of calcium (Ca);Provided, however, That enriched selfrising corn meals shall contain in each pound not more than 1,750 milligrams of calcium (Ca).
8. States fortificants (chemical compounds) to be used (including fortificants that are allowable as options)	(2) States fortificants for at least one nutrient	(1) It contains in each pound not less than 2.0 milligrams (mg) and not more than 3.0 mg of thiamin, not less than 1.2 mg and not more than 1.8 mg of riboflavin, not less than 16 mg and not more than 24 mg of niacin or niacinamide, not less than 0.7 mg and not more than 1.0 mg of folic acid, and not less than 13 mg and not more than 26 mg of iron (Fe); (2) It may contain in each pound not less than 250 U.S.P. units and not more than 1,000 U.S.P. units of vitamin D; and (3) It may contain in each pound not less than 500 milligrams and not more than 750 milligrams of calcium (Ca);Provided, however, That enriched selfrising corn meals shall contain in each pound not more than 1,750 milligrams of calcium (Ca). DOES NOT SPECIFY IRON TYPE

9. States fortification levels	(2) States a range or number with +/-	(1) It contains in each pound not less than 2.0 milligrams (mg) and not more than 3.0 mg of thiamin, not less than 1.2 mg and not more than 1.8 mg of riboflavin, not less than 16 mg and not more than 24 mg of niacin or niacinamide, not less than 0.7 mg and not more than 1.0 mg of folic acid, and not less than 13 mg and not more than 26 mg of iron (Fe); (2) It may contain in each pound not less than 250 U.S.P. units and not more than 1,000 U.S.P. units of vitamin D; and (3) It may contain in each pound not less than 500 milligrams and not more than 750 milligrams of calcium (Ca);Provided, however, That enriched selfrising corn meals shall contain in each pound not more than 1,750 milligrams of calcium (Ca).
10. States consideration of bioavailability/biological activity of fortificants	(2) States some consideration of bioavailability (mentions these or related terms)	With respect to issues of bioavailability, FDA concluded that bioavailability cannot be meaningfully factored into fortification scenarios because issues of bioavailability are very complex, and no systematic data are available on many of the factors that affect bioavailability.
11. States consideration of nutrient stability	(2) States consideration of nutrient stability	(c) The requirement of paragraphs (a) and (b) of this section will be deemed to have been met if reasonable overages of the vitamins and minerals, within the limits of good manufacturing practice, are present to insure that the required levels of the vitamins and minerals are maintained throughout the expected shelf life of the food under customary conditions of distribution and storage.<br>Review food additives and enrichment in storage to determine if allowed in standards (21 CFR 137).<br>Calculate percentages or amounts in the finished product per CWT (hundred weight or 100 lbs.) of flour (remember to be consistent with the units utilized in the standards.). Enrichment held for a year or more, or held at abnormally high temperatures or humidity, may have deteriorated.
**COSTING**		
12. States that the cost of fortification is regulated through cost sharing schemes (between government, industry, consumers) or tax measures [to assist industry]	(2) States consideration of either cost regulation method	c. Reformulation costs. Reformulation costs associated with this option were estimated in the proposal to be $27 million for the first year. The cost of adding the required folic acid is approximately $4 million per year. The cost of testing was estimated to be about $2.5 million per year and the cost of the required label changes $20 million. FDA will use these costs for this final rule as no comments were received on this part of the analysis.
13. States consideration of the financial responsibility of monitoring and enforcing fortification (schedule of fees, budget) [on the government side]	(2) Shows consideration that monitoring costs money	c. Reformulation costs. Reformulation costs associated with this option were estimated in the proposal to be $27 million for the first year. The cost of adding the required folic acid is approximately $4 million per year. The cost of testing was estimated to be about $2.5 million per year and the cost of the required label changes $20 million. FDA will use these costs for this final rule as no comments were received on this part of the analysis.
**LABELING**		
14. Includes some sort of statement/label/logo that makes it clear that the product is fortified	(2) Includes a statement, label, or logo	(b) The name of each kind of enriched corn meal is the word "Enriched" followed by the name of the kind of corn meal used which is prescribed in the definition and standard of identity therefor.<br>(c)Label declaration. Each of the ingredients used in the food shall be declared on the label as required by the applicable sections of parts 101 and 130 of this chapter.

15. Provides guidance on health claims that can be made for this product (specific to micronutrients added through fortification)	(2) Provides guidance on health claims specific to micronutrients added through fortification	but, if insufficient additional calcium is present to meet the 960 milligram level, no claim may be made on the label for calcium as a nutrient; (d) Model health claims. The following are examples of model health claims that may be used in food labeling to describe the relationship between folate and neural tube defects: (1) Examples 1 and 2. Model health claims appropriate for foods containing 100 percent or less of the DV for folate per serving or per unit (general population). The examples contain only the required elements: (i) Healthful diets with adequate folate may reduce a woman's risk of having a child with
**INTERNAL MONITORING (conducted by industry)**		
16. If samples are to be taken, describes the sampling process: for example, number of samples, amount, frequency, individual vs. composite, where samples are taken in the process, and percent considered passing	(N/A) Does not describe the sampling process	
17. States that industry is required to follow quality assurance/quality control in regards to fortification	(0) Does not state requirement	
18. States applicability of using qualitative testing to determine the presence or absence of a vitamin or mineral (spot tests, iChecks)	(0) Does not state	
**EXTERNAL MONITORING (conducted by government)**		
19.States requirement for external monitoring at the production site to assure compliance with standards and regulations	(0) Does not state requirement	
20. Describes protocols and systems for regulatory monitoring	(0) Does not describe	
21. If there are two or more government agencies involved in external monitoring, clarifies the roles and responsibilities between different government agencies in external monitoring	(0) Clarifies roles and responsibilities for no agencies	
22. Allows for monitoring to be conducted often enough that problems can be identified and addressed on a timely basis; specifies a timeline for inspections (i.e. once every 6 months, increasing to once every 2 months if a discrepancy is found)	(0) Does not state	
23. If samples are to be taken, describes the sampling process: for example, number of samples, amount, frequency, individual vs. composite, where samples are taken in the process, and percent considered passing	(1) States that samples should be taken (generally)	Vitamins and minerals - Samples will be collected on assignment basis or if inspectional conditions warrant.<br>1. Retail Packages 4.5kg (10 lbs or less) -Collect twelve intact packages in duplicate of the same code.<br>2. Bulk and Retail Packages 4.5kg (Larger than 10 lbs) - Collect 12 - 453 gm (1 lb) subs in duplicate from each of twelve different containers of the same code. Remove flour or other cereal product with a trier inserted the full length of the container. Prepare each sub in individual airtight containers and protect against sunlight by wrapping in opaque paper. Do not fumigate samples.
24. States applicability of using qualitative testing to determine the presence or absence of a vitamin or mineral (spot tests, iChecks)	(0) Does not state	
25. States registration is required in order to use a logo/be licensed to produce fortified foods	(0) Does not state that registration or licensing is required	
**COMMERCIAL MONITORING (conducted by government)**		
26. Provides justification for commercial monitoring at retail stores	(0) Does not provide justification for commercial monitoring	
27. Describes protocols and systems for commercial monitoring	(0) Does not describe	
28. If there are two or more government agencies involved in commercial monitoring, clarifies the roles and responsibilities between different government agencies in commercial monitoring	(0) Clarifies roles and responsibilities for no agencies	
29. Allows for monitoring to be conducted often enough that problems at the production site or import companies can be identified and addressed on a timely basis; specifies a timeline for inspections (i.e. once every 6 months) or works with production companies to correct noncompliance	(N/A) No commercial monitoring occurs	

30. If samples are to be taken, describes the sampling process: for example, number of samples, amount, frequency, individual vs. composite, where samples are taken in the process, and percent considered passing	(1) States that samples should be taken (generally)	Enrichment, Minerals and Nutrition Labeling - Bread, rolls, baked or unbaked product: Compliance samples should consist of 12 subsamples of at least 1 lb. each (454 gm) collected in duplicate. Collect consumer size retail packages (2 - 1 lb. subs from each of 12 cases) from separate cases of the same code. Protect enrichment samples from sun by wrapping in opaque paper. Determine and record by sub the gross weight.
**IMPORT MONITORING (conducted by government)**		
31. Provides justification for import monitoring at points of entry	(0) Does not provide justification for import monitoring	Import document super general--not specific to flour
32. Describes protocols and systems for import monitoring	(2) Includes checklists or detailed description of import monitoring procedures	
33. If there are two or more government agencies involved in import monitoring, clarifies the roles and responsibilities between different government agencies in import monitoring	(2) Clarifies roles and responsibilities for more than one agency	The purpose of this chapter is to provide an overview of import procedures for articles subject to the laws and regulations enforced by the Food and Drug Administration (FDA). The chapter also includes an overview of laws and regulations enforced by U.S. Customs and Border Protection (CBP), as they relate to importation of articles regulated by FDA. Division of Authority section
34. If samples are to be taken, describes the sampling process: for example, number of samples, amount, frequency, individual vs. composite, where samples are taken in the process, and percent considered passing	(1) States that samples should be taken (generally)	additional documentation is made, FDA will take appropriate action. If FDA decides to collect a sample, it will provide the filer, importer, owner and/or consignee, where appropriate, with a Notice of Sampling and advise: (1) whether the entry is to be held intact for FDA examination or sampling; or, (2) specify only those items that need be held. Generally, when FDA wishes to sample, it will be acting upon its authority in the Customs regulations (19 CFR 151.4) to collect its own samples for examination. FDA may request an examination or sample of articles under its jurisdiction. If no examination or sample is requested, FDA will so notify CBP and the filer, who is responsible for notifying the importer or other designated parties. This is referred to as a "May Proceed Notice," and indicates that the shipment may proceed without further FDA examination. In the ACS/OASIS process this may occur as a result of the initial FDA/OASIS screening prior to the information being forwarded to the district office, or after the district performs an "On-Screen-Review" of the information provided. (Note: Should the article, at a later time, be found in violation of the law, FDA is not prevented from taking legal action (e.g., seizure, injunction) because it allowed admission of the article without examination at the time of importation.) If FDA requests an examination or sample, FDA will notify CBP and the broker or filer, importer, or other
**ENFORCEMENT/PENALTIES**		
35. Indicates roles and responsibilities in enforcing the legislation	(2) States the role and responsibilities of government in enforcement	The purpose of this chapter is to provide an overview of import procedures for articles subject to the laws and regulations enforced by the Food and Drug Administration (FDA). The chapter also includes an overview of laws and regulations enforced by U.S. Customs and Border Protection (CBP), as they relate to importation of articles regulated by FDA.
36. States incentives to start fortification	(0) Does not state	
37. States incentives to continue fortification, including ensuring compliance	(0) Does not state	
38. States penalties to compel compliance	(2) States any penalties	Consequently, FDA may issue a warning letter and take enforcement action if a marketed food bearing one or more of these nutrient content claims contains a nutrient addition that is inconsistent with the fortification policy as incorporated into the regulations noted above. Nevertheless, FDA strongly encourages you to follow these fortification guidelines regardless of whether any claims appear on the label or in labeling.
39. Penalties are objectively defined (e.g. first penalty=$100, second penalty=$300)	(N/A) No penalties are stated (Answered (0) to previous question)	
40. States that enforcement is required to include feedback and support to improve performance and correct noncompliance	(0) Does not require	
**LABORATORY**		

41. References required analytical assays for nutrients (e.g. Liquid chromatography-mass spectrometry for folic acid, atomic absorption for iron and zinc.)	(0) Does not state requirements	The quantitative content of the following vitamins shall be calculated in terms of the following chemically identifiable reference forms:<br>Vitamin<br>Reference form<br>Name Empirical formula<br>Molecular<br>weight<br>Thiamine ... Thiamine chloride hydrochloride.<br>C12H17ClN4OS·HCl 337.28<br>Riboflavin .. Riboflavin .... C17H20N4O6 376.37<br>Niacin .... Niacin .... C6H5NO2 123.11<br>The method referred to in paragraph (a) of this section is the method prescribed in ‘‘Official Methods of Analysis of the Association of Official Analytical Chemists’’ (AOAC), 13th Ed. (1980), section 8.002, ‘‘Reagent (Displacement soln.),’’	(c)
42. States recognition that laboratory results are subject to several sources of variation and do not provide conclusive evidence of compliance or noncompliance	(0) Does not state recognition		
43. Focuses on the quantitative analysis of "marker" micronutrients such as iron	(0) Does not state		
**REPORTING**			
44. States how government monitoring results are shared with stakeholders	(0) Does not state how results are shared		

Venezuela Maize

Element	Scoring (use drop-down menus)	Comments (open text)	
**GENERAL**			
1. States that legislation applies to at least one food vehicle fit for human consumption (types/grades to be fortified)	(2) States at least one type fit for human consumption	1 OBJETO	
2. States the public health objective; purpose and scope of legislation	(0) Does not state		
3. References latest available science or accepted international norms and recommendations, particularly for items that may not be covered in the country's documents	(0) Does not state		
4. Provides definitions that includes terms that are specific to fortification (e.g. fortified food, premix, fortificant, food vehicle)	(2) States at least one term related to fortification	3 DEFINICIONES	
5. Provides repeals (if there is at least one prior document about fortification)	(0) Does not state		
6. Provides effective date or gives grace period for when fortification is to begin (e.g. effective 6 months from signing)	(0) Does not state		
**MICRONUTRIENTS/PREMIX**			
7. States nutrients required	(2) States nutrients	4.3 & Tabla 3	
8. States fortificants (chemical compounds) to be used (including fortificants that are allowable as options)	(2) States fortificants for at least one nutrient		4.3
9. States fortification levels	(2) States a range or number with +/-	Tabla 3	
10. States consideration of bioavailability/biological activity of fortificants	(2) States some consideration of bioavailability (mentions these or related terms)		4.4
11. States consideration of nutrient stability	(0) Does not state any consideration		
**COSTING**			
12. States that the cost of fortification is regulated through cost sharing schemes (between government, industry, consumers) or tax measures [to assist industry]	(0) Does not state any consideration		
13. States consideration of the financial responsibility of monitoring and enforcing fortification (schedule of fees, budget) [on the government side]	(0) Does not state any consideration		
**LABELING**			
14. Includes some sort of statement/label/logo that makes it clear that the product is fortified	(2) Includes a statement, label, or logo	10.2.1.1	
15. Provides guidance on health claims that can be made for this product (specific to micronutrients added through fortification)	(0) Does not provide		
**INTERNAL MONITORING (conducted by industry)**			
16. If samples are to be taken, describes the sampling process: for example, number of samples, amount, frequency, individual vs. composite, where samples are taken in the process, and percent considered passing	(N/A) Does not describe the sampling process		
17. States that industry is required to follow quality assurance/quality control in regards to fortification	(0) Does not state requirement		
18. States applicability of using qualitative testing to determine the presence or absence of a vitamin or mineral (spot tests, iChecks)	(0) Does not state		
**EXTERNAL MONITORING (conducted by government)**			
19.States requirement for external monitoring at the production site to assure compliance with standards and regulations	(0) Does not state requirement		
20. Describes protocols and systems for regulatory monitoring	(0) Does not describe		
21. If there are two or more government agencies involved in external monitoring, clarifies the roles and responsibilities between different government agencies in external monitoring	(0) Clarifies roles and responsibilities for no agencies		
22. Allows for monitoring to be conducted often enough that problems can be identified and addressed on a timely basis; specifies a timeline for inspections (i.e. once every 6 months, increasing to once every 2 months if a discrepancy is found)	(0) Does not state		
23. If samples are to be taken, describes the sampling process: for example, number of samples, amount, frequency, individual vs. composite, where samples are taken in the process, and percent considered passing	(2) States that samples should be taken as part of external monitoring	6 TOMA DE MUESTRAS	
24. States applicability of using qualitative testing to determine the presence or absence of a vitamin or mineral (spot tests, iChecks)	(0) Does not state		
25. States registration is required in order to use a logo/be licensed to produce fortified foods	(0) Does not state that registration or licensing is required		
**COMMERCIAL MONITORING (conducted by government)**			
26. Provides justification for commercial monitoring at retail stores	(0) Does not provide justification for commercial monitoring		

27. Describes protocols and systems for commercial monitoring	(0) Does not describe	
28. If there are two or more government agencies involved in commercial monitoring, clarifies the roles and responsibilities between different government agencies in commercial monitoring	(0) Clarifies roles and responsibilities for no agencies	
29. Allows for monitoring to be conducted often enough that problems at the production site or import companies can be identified and addressed on a timely basis; specifies a timeline for inspections (i.e. once every 6 months) or works with production companies to correct noncompliance	(N/A) No commercial monitoring occurs	
30. If samples are to be taken, describes the sampling process: for example, number of samples, amount, frequency, individual vs. composite, where samples are taken in the process, and percent considered passing	(N/A) Does not describe the sampling process	
**IMPORT MONITORING (conducted by government)**		
31. Provides justification for import monitoring at points of entry	(0) Does not provide justification for import monitoring	
32. Describes protocols and systems for import monitoring	(0) Does not state	
33. If there are two or more government agencies involved in import monitoring, clarifies the roles and responsibilities between different government agencies in import monitoring	(0) Clarifies roles and responsibilities for no agencies	
34. If samples are to be taken, describes the sampling process: for example, number of samples, amount, frequency, individual vs. composite, where samples are taken in the process, and percent considered passing	(N/A) Does not describe the sampling process	
**ENFORCEMENT/PENALTIES**		
35. Indicates roles and responsibilities in enforcing the legislation	(2) States the role and responsibilities of government in enforcement	
36. States incentives to start fortification	(0) Does not state	
37. States incentives to continue fortification, including ensuring compliance	(0) Does not state	
38. States penalties to compel compliance	(0) Does not state	
39. Penalties are objectively defined (e.g. first penalty=$100, second penalty=$300)	(N/A) No penalties are stated (Answered (0) to previous question)	
40. States that enforcement is required to include feedback and support to improve performance and correct noncompliance	(0) Does not require	
**LABORATORY**		
41. References required analytical assays for nutrients (e.g. Liquid chromatography-mass spectrometry for folic acid, atomic absorption for iron and zinc.)	(2) References required assays	Tabla 3
42. States recognition that laboratory results are subject to several sources of variation and do not provide conclusive evidence of compliance or noncompliance	(0) Does not state recognition	
43. Focuses on the quantitative analysis of "marker" micronutrients such as iron	(0) Does not state	Tabla 3/8.1.1--3 of 5 nutrients (or 2 of 4) must meet nutrient standards in a sample
**REPORTING**		
44. States how government monitoring results are shared with stakeholders	(0) Does not state how results are shared	

Costa Rica Rice

Element	Scoring (use drop-down menus)	Comments (open text)
**GENERAL**		
1. States that legislation applies to at least one food vehicle fit for human consumption (types/grades to be fortified)	(2) States at least one type fit for human consumption	"Artículo 1°—Las disposiciones del presente reglamento se aplican al arroz que se utiliza para el consumo directo humano en el país, sea éste de producción nacional, donado o importado."<br>"Articulo 3°—El arroz pilado que se utilice para el consumo humano directo deberá estar fortificado con ácido fólico, vitaminas del complejo B, vitamina E, selenio y zinc, los cuales pueden provenir de una o varias mezclas con excipientes, de manera tal que una dilución específica de ellos produzca los niveles mínimos que se especifican a continuación:"
2. States the public health objective; purpose and scope of legislation	(2) States the public health objective or general purpose of legislation	"Considerando:<br>1°—Que es función del Estado velar por la protección de la salud de la población.<br>2°—Que las malformaciones congénitas del tubo neural están entre las primeras causas de mortalidad infantil y la enfermedad cardiovascular es una de las primeras causas de mortalidad general.<br>3°—Que el ácido fólico es un nutriente indispensable para el desarrollo físico y mental, la prevención de las malformaciones congénitas del tubo neural y la enfermedad cardiovascular en el ser humano.<br>4°—Que los resultados aportados por las encuestas nacionales de nutrición de 1982 y 1996 y los sitios centinelas en alimentación y nutrición de 1999 y 2000, mostraron que las anemias nutricionales por deficiencia de hierro constituyen un problema de salud pública."
3. References latest available science or accepted international norms and recommendations, particularly for items that may not be covered in the country's documents	(0) Does not state	
4. Provides definitions that includes terms that are specific to fortification (e.g. fortified food, premix, fortificant, food vehicle)	(2) States at least one term related to fortification	"Artículo 2°—Para efectos del presente reglamento se entenderá por:"
5. Provides repeals (if there is at least one prior document about fortification)	(N/A) No prior documents about fortification	
6. Provides effective date or gives grace period for when fortification is to begin (e.g. effective 6 months from signing)	(2) States effective date or grace period for when fortification is to begin (e.g. effective 6 months from signing)	"Artículo 14.—Rige a partir de un año después de su publicación.<br>Transitorio I.—Se otorga un plazo adicional de seis meses a partir de la fecha de entrada en vigencia del presente Reglamento a efecto de que los pequeños industriales cumplan con las disposiciones aquí establecidas."
**MICRONUTRIENTS/PREMIX**		
7. States nutrients required	(2) States nutrients	"Articulo 3°—El arroz pilado que se utilice para el consumo humano directo deberá estar fortificado con ácido fólico, vitaminas del complejo B, vitamina E, selenio y zinc, los cuales pueden provenir de una o varias mezclas con excipientes, de manera tal que una dilución específica de ellos produzca los niveles mínimos que se especifican a continuación:"
8. States fortificants (chemical compounds) to be used (including fortificants that are allowable as options)	(0) Does not state	
9. States fortification levels	(1) States one number only	"Articulo 3°—El arroz pilado que se utilice para el consumo humano directo deberá estar fortificado con ácido fólico, vitaminas del complejo B, vitamina E, selenio y zinc, los cuales pueden provenir de una o varias mezclas con excipientes, de manera tal que una dilución específica de ellos produzca los niveles mínimos que se especifican a continuación:"<br>"Estos valores incluyen el contenido intrínseco y natural de micronutrientes en el arroz."<br>"1.2. Para cumplir con estos requisitos, los niveles de micronutrientes a adicionar serán: [lists nutrient levels that should be *added* by fortification]<br>Estos micronutrientes deberán ser agregados al arroz en forma de arroz extruido o arroz recubierto, en ambos casos se deberá asegurar homogeneidad y resistencia al lavado en al menos un 80%."
10. States consideration of bioavailability/biological activity of fortificants	(0) Does not state any consideration	

11. States consideration of nutrient stability	(2) States consideration of nutrient stability	1.2. Para cumplir con estos requisitos, los niveles de micronutrientes a adicionar serán: [lists nutrient levels that should be *added* by fortification]<br>Estos micronutrientes deberán ser agregados al arroz en forma de arroz extruido o arroz recubierto, en ambos casos se deberá asegurar homogeneidad y resistencia al lavado en al menos un 80%.
**COSTING**		
12. States that the cost of fortification is regulated through cost sharing schemes (between government, industry, consumers) or tax measures [to assist industry]	(0) Does not state any consideration	
13. States consideration of the financial responsibility of monitoring and enforcing fortification (schedule of fees, budget) [on the government side]	(0) Does not state any consideration	
**LABELING**		
14. Includes some sort of statement/label/logo that makes it clear that the product is fortified	(2) Includes a statement, label, or logo	"Artículo 9°—El etiquetado del arroz se ajustará a lo estipulado en el Decreto No 26012-ME1C del 15 de abril de 1997 "Norma General de Etiquetado de los Alimentos Preenvasados" y deberá especificar que se trata de un producto fortificado con la frase "ARROZ FORTIFICADO" o "ARROZ ENRIQUECIDO", deberá indicar además el contenido final total de micronutrientes (el adicionado y el contenido naturalmente)."
15. Provides guidance on health claims that can be made for this product (specific to micronutrients added through fortification)	(2) Provides guidance on health claims specific to micronutrients added through fortification	"7.1 Declaraciones Nutricionales<br>Las únicas declaraciones de propiedades nutricionales permitidas deben ser las que se refieran a energía, proteínas, carbohidratos, grasas y los componentes de las mismas, fibra, vitaminas y minerales para los cuales se hayan establecido recomendaciones nutricionales."<br>"7.4 Declaraciones de Propiedades Saludables"<br>Anexo G "3.9 Ácido fólico y defectos del conducto (tubo) neural:"
**INTERNAL MONITORING (conducted by industry)**		
16. If samples are to be taken, describes the sampling process: for example, number of samples, amount, frequency, individual vs. composite, where samples are taken in the process, and percent considered passing	(N/A) Does not describe the sampling process	
17. States that industry is required to follow quality assurance/quality control in regards to fortification	(2) States requirement of QA/QC for fortification	"Artículo 4°—La garantía de calidad o control interno del arroz fortificado con los niveles indicados en el artículo anterior son de responsabilidad de los industriales y de los importadores de arroz."
18. States applicability of using qualitative testing to determine the presence or absence of a vitamin or mineral (spot tests, iChecks)	(0) Does not state	
**EXTERNAL MONITORING (conducted by government)**		
19.States requirement for external monitoring at the production site to assure compliance with standards and regulations	(2) States requirement for external monitoring or the need for audits/inspections	"Artículo 5°—<br>Para el arroz producido en el país se establecerá un sistema de control a nivel de producción."<br>"Articulo 6°—El Ministerio de Salud podrá confirmar, mediante muestreo o análisis de muestras en el mercado o en las bodegas del industrial o importador, el cumplimiento del arroz importado con los niveles de fortificación establecidos en el presente reglamento."
20. Describes protocols and systems for regulatory monitoring	(0) Does not describe	
21. If there are two or more government agencies involved in external monitoring, clarifies the roles and responsibilities between different government agencies in external monitoring	(N/A) Only one government agency involved	"Artículo 18.—El Ministerio de Salud coordinará con los demás entes públicos involucrados en la materia todas las acciones necesarias para asegurar la cabal aplicación y cumplimiento del presente reglamento."
22. Allows for monitoring to be conducted often enough that problems can be identified and addressed on a timely basis; specifies a timeline for inspections (i.e. once every 6 months, increasing to once every 2 months if a discrepancy is found)	(0) Does not state	
23. If samples are to be taken, describes the sampling process: for example, number of samples, amount, frequency, individual vs. composite, where samples are taken in the process, and percent considered passing	(1) States that samples should be taken (generally)	Articulo 6°—El Ministerio de Salud podrá confirmar, mediante muestreo o análisis de muestras en el mercado o en las bodegas del industrial o importador, el cumplimiento del arroz importado con los niveles de fortificación establecidos en el presente reglamento.
24. States applicability of using qualitative testing to determine the presence or absence of a vitamin or mineral (spot tests, iChecks)	(0) Does not state	
25. States registration is required in order to use a logo/be licensed to produce fortified foods	(0) Does not state that registration or licensing is required	
**COMMERCIAL MONITORING (conducted by government)**		

26. Provides justification for commercial monitoring at retail stores	(2) Provides justification for commercial monitoring	"Articulo 6°—El Ministerio de Salud podrá confirmar, mediante muestreo o análisis de muestras en el mercado o en las bodegas del industrial o importador, el cumplimiento del arroz importado con los niveles de fortificación establecidos en el presente reglamento."
27. Describes protocols and systems for commercial monitoring	(1) Does not explicitly describe, but references protocols and systems for regulatory monitoring	"Artículo 8°—La fiscalización y el monitoreo de la calidad de la fortificación del arroz en fábricas, sitios de expendio y otros son responsabilidad de las autoridades del Ministerio de Salud, quienes además diseñaran y establecerán un sistema de control de calidad para el arroz fortificado. Por otra parte el Ministerio podrá verificar los niveles de vitaminas y minerales en la premezcla y en el arroz, mediante análisis cuantitativos eventuales y establecerá los criterios técnicos de los procedimientos y técnicas de laboratorio para el análisis de las muestras de arroz fortificado."
28. If there are two or more government agencies involved in commercial monitoring, clarifies the roles and responsibilities between different government agencies in commercial monitoring	(N/A) Only one government agency involved	"Artículo 8°—La fiscalización y el monitoreo de la calidad de la fortificación del arroz en fábricas, sitios de expendio y otros son responsabilidad de las autoridades del Ministerio de Salud, quienes además diseñaran y establecerán un sistema de control de calidad para el arroz fortificado. Por otra parte el Ministerio podrá verificar los niveles de vitaminas y minerales en la premezcla y en el arroz, mediante análisis cuantitativos eventuales y establecerá los criterios técnicos de los procedimientos y técnicas de laboratorio para el análisis de las muestras de arroz fortificado."
29. Allows for monitoring to be conducted often enough that problems at the production site or import companies can be identified and addressed on a timely basis; specifies a timeline for inspections (i.e. once every 6 months) or works with production companies to correct noncompliance	(0) Does not state	
30. If samples are to be taken, describes the sampling process: for example, number of samples, amount, frequency, individual vs. composite, where samples are taken in the process, and percent considered passing	(N/A) Does not describe the sampling process	
**IMPORT MONITORING (conducted by government)**		
31. Provides justification for import monitoring at points of entry	(2) Provides justification for import monitoring	"Artículo 4°—La garantía de calidad o control interno del arroz fortificado con los niveles indicados en el artículo anterior son de responsabilidad de los industriales y de los importadores de arroz."<br>"Articulo 6°—El Ministerio de Salud podrá confirmar, mediante muestreo o análisis de muestras en el mercado o en las bodegas del industrial o importador, el cumplimiento del arroz importado con los niveles de fortificación establecidos en el presente reglamento."
32. Describes protocols and systems for import monitoring	(2) Includes checklists or detailed description of import monitoring procedures	"Artículo 5°—Para autorizar el desalmacenaje de arroz pulido importado, el importador deberá demostrar en el certificado de conformidad del país de origen o certificado de análisis realizado en un laboratorio acreditado, que el producto cumple con la fortificación establecida en el presente reglamento, para cada partida de importaciones.<br>Para el arroz producido en el país se establecerá un sistema de control a nivel de producción."
33. If there are two or more government agencies involved in import monitoring, clarifies the roles and responsibilities between different government agencies in import monitoring	(N/A) Only one government agency involved	"Artículo 5°—Para autorizar el desalmacenaje de arroz pulido importado, el importador deberá demostrar en el certificado de conformidad del país de origen o certificado de análisis realizado en un laboratorio acreditado, que el producto cumple con la fortificación establecida en el presente reglamento, para cada partida de importaciones.<br>Para el arroz producido en el país se establecerá un sistema de control a nivel de producción."
34. If samples are to be taken, describes the sampling process: for example, number of samples, amount, frequency, individual vs. composite, where samples are taken in the process, and percent considered passing	(1) States that samples should be taken (generally)	"Articulo 6°—El Ministerio de Salud podrá confirmar, mediante muestreo o análisis de muestras en el mercado o en las bodegas del industrial o importador, el cumplimiento del arroz importado con los niveles de fortificación establecidos en el presente reglamento."
**ENFORCEMENT/PENALTIES**		

35. Indicates roles and responsibilities in enforcing the legislation	(2) States the role and responsibilities of government in enforcement	"Artículo 8°—La fiscalización y el monitoreo de la calidad de la fortificación del arroz en fábricas, sitios de expendio y otros son responsabilidad de las autoridades del Ministerio de Salud, quienes además diseñaran y establecerán un sistema de control de calidad para el arroz fortificado. Por otra parte el Ministerio podrá verificar los niveles de vitaminas y minerales en la premezcla y en el arroz, mediante análisis cuantitativos eventuales y establecerá los criterios técnicos de los procedimientos y técnicas de laboratorio para el análisis de las muestras de arroz fortificado." "Artículo 13.—El Ministerio de Salud coordinará con los demás entes públicos involucrados en la materia, todas las acciones necesarias para asegurar la cabal aplicación y cumplimiento del presente Reglamento."
36. States incentives to start fortification	(0) Does not state	
37. States incentives to continue fortification, including ensuring compliance	(0) Does not state	
38. States penalties to compel compliance	(2) States any penalties	"Artículo 7°—En caso de establecerse que un lote de arroz producido en el país o importado no esté debidamente fortificado, se procederá al decomiso de la cantidad total del lote, siguiendo los procedimientos establecidos por el Ministerio de Salud."
39. Penalties are objectively defined (e.g. first penalty=$100, second penalty=$300)	(0) Penalties are not objectively laid out	
40. States that enforcement is required to include feedback and support to improve performance and correct noncompliance	(0) Does not require	
**LABORATORY**		
41. References required analytical assays for nutrients (e.g. Liquid chromatography-mass spectrometry for folic acid, atomic absorption for iron and zinc.)	(0) Does not state requirements	
42. States recognition that laboratory results are subject to several sources of variation and do not provide conclusive evidence of compliance or noncompliance	(0) Does not state recognition	
43. Focuses on the quantitative analysis of "marker" micronutrients such as iron	(0) Does not state	
**REPORTING**		
44. States how government monitoring results are shared with stakeholders	(0) Does not state how results are shared	

Nicaragua Rice

Element	Scoring (use drop-down menus)	Comments (open text)
**GENERAL**		
1. States that legislation applies to at least one food vehicle fit for human consumption (types/grades to be fortified)	(2) States at least one type fit for human consumption	2. CAMPO DE APLICACION
2. States the public health objective; purpose and scope of legislation	(2) States the public health objective or general purpose of legislation	CONSIDERANDO
3. References latest available science or accepted international norms and recommendations, particularly for items that may not be covered in the country's documents	(2) States the documents referenced	6.1.1 Codex
4. Provides definitions that includes terms that are specific to fortification (e.g. fortified food, premix, fortificant, food vehicle)	(2) States at least one term related to fortification	3. DEFINICIONES. There's a typo here "arroz excluido" is written in lieu of "arroz extruido" to describe extruded kernels.
5. Provides repeals (if there is at least one prior document about fortification)	(0) Does not state	
6. Provides effective date or gives grace period for when fortification is to begin (e.g. effective 6 months from signing)	(2) States effective date or grace period for when fortification is to begin (e.g. effective 6 months from signing)	15. ENTRADA EN VIGENCIA
**MICRONUTRIENTS/PREMIX**		
7. States nutrients required	(2) States nutrients	5. FORTIFICACION DE ARROZ
8. States fortificants (chemical compounds) to be used (including fortificants that are allowable as options)	(2) States fortificants for at least one nutrient	5. FORTIFICACION DE ARROZ
9. States fortification levels	(1) States one number only	5. FORTIFICACION DE ARROZ
10. States consideration of bioavailability/biological activity of fortificants	(2) States some consideration of bioavailability (mentions these or related terms)	"SEGUNDO: Es obligatoria la fortificación del arroz y sus derivados con ácido fólico, hierro y otros micronutrientes de la más alta biodisponibilidad…"
11. States consideration of nutrient stability	(2) States consideration of nutrient stability	"5.3 Procedimiento de fortificación. La premezcla de estos micronutrientes debe ser agregada al arroz, en forma de arroz extruido o recubierto, que garantice homogoneidad y resistencia al lavado en al menos un 80%."
**COSTING**		
12. States that the cost of fortification is regulated through cost sharing schemes (between government, industry, consumers) or tax measures [to assist industry]	(0) Does not state any consideration	The opposite: "OCTAVO: Los costos del proceso de fortificación y el valor de los insumos requeridos serán asumidos por los productores y procesadores de arroz."
13. States consideration of the financial responsibility of monitoring and enforcing fortification (schedule of fees, budget) [on the government side]	(0) Does not state any consideration	
**LABELING**		
14. Includes some sort of statement/label/logo that makes it clear that the product is fortified	(2) Includes a statement, label, or logo	10. ETIQUETADO "10.2 Se debe indicar en la etiqueta que el arroz es fortificado, e incluir la información nutricional."
15. Provides guidance on health claims that can be made for this product (specific to micronutrients added through fortification)	(2) Provides guidance on health claims specific to micronutrients added through fortification	"7.1 Declaraciones Nutricionales<br>Las únicas declaraciones de propiedades nutricionales permitidas deben ser las que se refieran a energía, proteínas, carbohidratos, grasas y los componentes de las mismas, fibra, vitaminas y minerales para los cuales se hayan establecido recomendaciones nutricionales."<br>"7.4 Declaraciones de Propiedades Saludables"<br>Anexo G "3.9 Ácido fólico y defectos del conducto (tubo) neural:"
**INTERNAL MONITORING (conducted by industry)**		
16. If samples are to be taken, describes the sampling process: for example, number of samples, amount, frequency, individual vs. composite, where samples are taken in the process, and percent considered passing	(N/A) Does not describe the sampling process	
17. States that industry is required to follow quality assurance/quality control in regards to fortification	(2) States requirement of QA/QC for fortification	11. VIGILANCIA Y CONTROL "11.1 La garantía de calidad o control interno del arroz fortificado con los niveles indicados es responsabilidad de los pequeños, medianos y grandes industrias y de los importadores de arroz."
18. States applicability of using qualitative testing to determine the presence or absence of a vitamin or mineral (spot tests, iChecks)	(0) Does not state	
**EXTERNAL MONITORING (conducted by government)**		
19.States requirement for external monitoring at the production site to assure compliance with standards and regulations	(2) States requirement for external monitoring or the need for audits/inspections	11. VIGILANCIA Y CONTROL "11.3 Para el arroz producido en el país se establecerá un sistema de control a nivel de producción, industrialización y distribución".
20. Describes protocols and systems for regulatory monitoring	(1) Does not explicitly describe, but references protocols and systems for regulatory monitoring	11. VIGILANCIA Y CONTROL "11.3 Para el arroz producido en el país se establecerá un sistema de control a nivel de producción, industrialización y distribución". There is no system in place now, but one will be put in place in the future.
21. If there are two or more government agencies involved in external monitoring, clarifies the roles and responsibilities between different government agencies in external monitoring	(N/A) Only one government agency involved	11.6 Ministry of Health

22. Allows for monitoring to be conducted often enough that problems can be identified and addressed on a timely basis; specifies a timeline for inspections (i.e. once every 6 months, increasing to once every 2 months if a discrepancy is found)	(0) Does not state		
23. If samples are to be taken, describes the sampling process: for example, number of samples, amount, frequency, individual vs. composite, where samples are taken in the process, and percent considered passing	(2) States that samples should be taken as part of external monitoring		11.4
24. States applicability of using qualitative testing to determine the presence or absence of a vitamin or mineral (spot tests, iChecks)	(0) Does not state		
25. States registration is required in order to use a logo/be licensed to produce fortified foods	(0) Does not state that registration or licensing is required		
**COMMERCIAL MONITORING (conducted by government)**			
26. Provides justification for commercial monitoring at retail stores	(2) Provides justification for commercial monitoring		11.4
27. Describes protocols and systems for commercial monitoring	(1) Does not explicitly describe, but references protocols and systems for regulatory monitoring	11. VIGILANCIA Y CONTROL "11.3 Para el arroz producido en el país se establecerá un sistema de control a nivel de producción, industrialización y distribución". There is no system in place now, but one will be put in place in the future.	
28. If there are two or more government agencies involved in commercial monitoring, clarifies the roles and responsibilities between different government agencies in commercial monitoring	(N/A) Only one government agency involved	11.6 Ministry of Health	
29. Allows for monitoring to be conducted often enough that problems at the production site or import companies can be identified and addressed on a timely basis; specifies a timeline for inspections (i.e. once every 6 months) or works with production companies to correct noncompliance	(0) Does not state		
30. If samples are to be taken, describes the sampling process: for example, number of samples, amount, frequency, individual vs. composite, where samples are taken in the process, and percent considered passing	(2) States that samples should be taken as part of commercial monitoring		11.4
**IMPORT MONITORING (conducted by government)**			
31. Provides justification for import monitoring at points of entry	(2) Provides justification for import monitoring	11.2	
32. Describes protocols and systems for import monitoring	(1) Does not explicitly state, but references protocols and systems for import monitoring	11. VIGILANCIA Y CONTROL "11.2"	
33. If there are two or more government agencies involved in import monitoring, clarifies the roles and responsibilities between different government agencies in import monitoring	(N/A) Only one government agency involved	11.6 Ministry of Health. Does not explicitly state MoH in charge of import monitoring but does state "and other locations".	
34. If samples are to be taken, describes the sampling process: for example, number of samples, amount, frequency, individual vs. composite, where samples are taken in the process, and percent considered passing	(1) States that samples should be taken (generally)		11.4
**ENFORCEMENT/PENALTIES**			
35. Indicates roles and responsibilities in enforcing the legislation	(2) States the role and responsibilities of government in enforcement	11.6 Ministry of Health	
36. States incentives to start fortification	(0) Does not state		
37. States incentives to continue fortification, including ensuring compliance	(0) Does not state		
38. States penalties to compel compliance	(2) States any penalties	11.5 & 13	
39. Penalties are objectively defined (e.g. first penalty=$100, second penalty=$300)	(0) Penalties are not objectively laid out	11.5 & 13	
40. States that enforcement is required to include feedback and support to improve performance and correct noncompliance	(0) Does not require		
**LABORATORY**			
41. References required analytical assays for nutrients (e.g. Liquid chromatography-mass spectrometry for folic acid, atomic absorption for iron and zinc.)	(2) References required assays	11.7 These are to be developed in the future.	
42. States recognition that laboratory results are subject to several sources of variation and do not provide conclusive evidence of compliance or noncompliance	(0) Does not state recognition		
43. Focuses on the quantitative analysis of "marker" micronutrients such as iron	(0) Does not state		
**REPORTING**			
44. States how government monitoring results are shared with stakeholders	(0) Does not state how results are shared		

Panama Rice

Element	Scoring (use drop-down menus)	Comments (open text)
**GENERAL**		
1. States that legislation applies to at least one food vehicle fit for human consumption (types/grades to be fortified)	(0) Does not state	"Artículo 1. Se crea el programa interinstitucional denominado Programa de Fortificación de Arroz en la República de Panamá, que consiste en fortificar el arroz blanco que se empaca en el país con ácido fólico, vitaminas B1, B6 y B12, niacina, hierro y zinc, en los siguientes niveles de adición:"
2. States the public health objective; purpose and scope of legislation	(0) Does not state	
3. References latest available science or accepted international norms and recommendations, particularly for items that may not be covered in the country's documents	(0) Does not state	
4. Provides definitions that includes terms that are specific to fortification (e.g. fortified food, premix, fortificant, food vehicle)	(2) States at least one term related to fortification	Artículo 2
5. Provides repeals (if there is at least one prior document about fortification)	(N/A) No prior documents about fortification	No prior documents about rice fortification.
6. Provides effective date or gives grace period for when fortification is to begin (e.g. effective 6 months from signing)	(2) States effective date or grace period for when fortification is to begin (e.g. effective 6 months from signing)	Artículo 3 "...Las empresas molineras empacadoras tendrán un plazo de dos meses después de instalados los dosificadores para tener su producto en el mercado con las especificaciones descritas en la presente Ley." Artículo 17
**MICRONUTRIENTS/PREMIX**		
7. States nutrients required	(2) States nutrients	Artículo 1
8. States fortificants (chemical compounds) to be used (including fortificants that are allowable as options)	(2) States fortificants for at least one nutrient	Artículo 1
9. States fortification levels	(1) States one number only	Artículo 1. Artículo 5 specifies that the average levels consider intrinsic plus fortification levels of nutrients.
10. States consideration of bioavailability/biological activity of fortificants	(2) States some consideration of bioavailability (mentions these or related terms)	"Artículo 4. Los micronutrientes serán incorporados al arroz por medio de la adición de una premezcla en una proporción de 1:200 (5kg por tonelada métrica). El tipo de hierro (pirofosfato férrico) podrá ser cambiado en el futuro por otro tipo de mejor biodisponibilidad y que mantenga las propiedades sensoriales del arroz, a un costo accesible. El Ministerio de Salud tomará la decisión de cambiar el tipo de hierro con base en resultados científicos y de factibilidad técnica."
11. States consideration of nutrient stability	(0) Does not state any consideration	
**COSTING**		
12. States that the cost of fortification is regulated through cost sharing schemes (between government, industry, consumers) or tax measures [to assist industry]	(2) States consideration of either cost regulation method	"Artículo 3. Para el desarrollo del Programa de Fortificación de Arroz, el Gobierno Nacional, a través del Ministerio de Desarrollo Agropecuario y del Instituto de Mercadeo Agropecuario, proporcionará a las empresas molineras empacadoras que operan en el territorio nacional la premezcla de granos fortificados, la cual deberán retirar de las bodegas que indique el Instituto de Mercadeo Agropecuario y agregar al arroz que se empaca en dichas empresas.<br>Las empresas molineras empacadoras deberán agregar la premezcla al arroz blanco, en proporción del medio por ciento (0.5%) del arroz que se empaca, a través de dosificadores que les serán instalados gradualmente durante un periodo no mayor de dieciocho meses por cuenta del Gobierno Nacional."
13. States consideration of the financial responsibility of monitoring and enforcing fortification (schedule of fees, budget) [on the government side]	(2) Shows consideration that monitoring costs money	Artículo 15
**LABELING**		
14. Includes some sort of statement/label/logo that makes it clear that the product is fortified	(2) Includes a statement, label, or logo	"Artículo 7. En el empaque del arroz blanco se deberá especificar que se trata de un alimento enriquecido o fortificado y llevará la leyenda "ARROZ ENRIQUECIDO" o "ARROZ<br>FORTIFICADO". Deberá indicar, además, el tipo y contenido total de micronutrientes, según lo dispuesto en el artículo 6."
15. Provides guidance on health claims that can be made for this product (specific to micronutrients added through fortification)	(0) Does not provide	
**INTERNAL MONITORING (conducted by industry)**		
16. If samples are to be taken, describes the sampling process: for example, number of samples, amount, frequency, individual vs. composite, where samples are taken in the process, and percent considered passing	(N/A) Does not describe the sampling process	
17. States that industry is required to follow quality assurance/quality control in regards to fortification	(0) Does not state requirement	

18. States applicability of using qualitative testing to determine the presence or absence of a vitamin or mineral (spot tests, iChecks)	(0) Does not state	
**EXTERNAL MONITORING (conducted by government)**		
19.States requirement for external monitoring at the production site to assure compliance with standards and regulations	(2) States requirement for external monitoring or the need for audits/inspections	"Artículo 9. El Departamento de Protección de Alimentos del Ministerio de Salud tendrá la responsabilidad de vigilar la fortificación del arroz en las empresas empacadoras y distribuidoras, así como en comercios y cualquier otro lugar que estime conveniente en el territorio nacional."
20. Describes protocols and systems for regulatory monitoring	(1) Does not explicitly describe, but references protocols and systems for regulatory monitoring	"Artículo 9. El Departamento de Protección de Alimentos del Ministerio de Salud tendrá la responsabilidad de vigilar la fortificación del arroz en las empresas empacadoras y distribuidoras, así como en comercios y cualquier otro lugar que estime conveniente en el territorio nacional."
21. If there are two or more government agencies involved in external monitoring, clarifies the roles and responsibilities between different government agencies in external monitoring	(2) Clarifies roles and responsibilities for more than one agency	Articulo 11, 12
22. Allows for monitoring to be conducted often enough that problems can be identified and addressed on a timely basis; specifies a timeline for inspections (i.e. once every 6 months, increasing to once every 2 months if a discrepancy is found)	(0) Does not state	
23. If samples are to be taken, describes the sampling process: for example, number of samples, amount, frequency, individual vs. composite, where samples are taken in the process, and percent considered passing	(N/A) Does not describe the sampling process	
24. States applicability of using qualitative testing to determine the presence or absence of a vitamin or mineral (spot tests, iChecks)	(0) Does not state	
25. States registration is required in order to use a logo/be licensed to produce fortified foods	(0) Does not state that registration or licensing is required	
**COMMERCIAL MONITORING (conducted by government)**		
26. Provides justification for commercial monitoring at retail stores	(2) Provides justification for commercial monitoring	"Artículo 9. El Departamento de Protección de Alimentos del Ministerio de Salud tendrá la responsabilidad de vigilar la fortificación del arroz en las empresas empacadoras y distribuidoras, así como en comercios y cualquier otro lugar que estime conveniente en el territorio nacional."
27. Describes protocols and systems for commercial monitoring	(1) Does not explicitly describe, but references protocols and systems for regulatory monitoring	Articulo 9, 10
28. If there are two or more government agencies involved in commercial monitoring, clarifies the roles and responsibilities between different government agencies in commercial monitoring	(2) Clarifies roles and responsibilities for more than one agency	Articulo 9, 12
29. Allows for monitoring to be conducted often enough that problems at the production site or import companies can be identified and addressed on a timely basis; specifies a timeline for inspections (i.e. once every 6 months) or works with production companies to correct noncompliance	(0) Does not state	
30. If samples are to be taken, describes the sampling process: for example, number of samples, amount, frequency, individual vs. composite, where samples are taken in the process, and percent considered passing	(N/A) Does not describe the sampling process	
**IMPORT MONITORING (conducted by government)**		
31. Provides justification for import monitoring at points of entry	(0) Does not provide justification for import monitoring	No import monitoring to be done as law applies to rice packaged in country.
32. Describes protocols and systems for import monitoring	(0) Does not state	
33. If there are two or more government agencies involved in import monitoring, clarifies the roles and responsibilities between different government agencies in import monitoring	(0) Clarifies roles and responsibilities for no agencies	
34. If samples are to be taken, describes the sampling process: for example, number of samples, amount, frequency, individual vs. composite, where samples are taken in the process, and percent considered passing	(N/A) Does not describe the sampling process	
**ENFORCEMENT/PENALTIES**		
35. Indicates roles and responsibilities in enforcing the legislation	(2) States the role and responsibilities of government in enforcement	"Artículo 9. El Departamento de Protección de Alimentos del Ministerio de Salud tendrá la responsabilidad de vigilar la fortificación del arroz en las empresas empacadoras y distribuidoras, así como en comercios y cualquier otro lugar que estime conveniente en el territorio nacional."

36. States incentives to start fortification	(2) States any incentives to encourage fortification initiation (e.g. tax incentives for new equipment or premix)	Articulo 3, 15
37. States incentives to continue fortification, including ensuring compliance	(2) States any incentives to encourage the continuation of fortification (e.g. transport priority, favorable tax or tariff treatment, or patent rights)	Articulo 3, 15
38. States penalties to compel compliance	(2) States any penalties	Artículo 16
39. Penalties are objectively defined (e.g. first penalty=$100, second penalty=$300)	(0) Penalties are not objectively laid out	Artículo 16
40. States that enforcement is required to include feedback and support to improve performance and correct noncompliance	(0) Does not require	
**LABORATORY**		
41. References required analytical assays for nutrients (e.g. Liquid chromatography-mass spectrometry for folic acid, atomic absorption for iron and zinc.)	(0) Does not state requirements	
42. States recognition that laboratory results are subject to several sources of variation and do not provide conclusive evidence of compliance or noncompliance	(0) Does not state recognition	
43. Focuses on the quantitative analysis of "marker" micronutrients such as iron	(0) Does not state	
**REPORTING**		
44. States how government monitoring results are shared with stakeholders	(0) Does not state how results are shared	

Papua New Guinea Rice

Element	Scoring (use drop-down menus)	Comments (open text)
**GENERAL**		
1. States that legislation applies to at least one food vehicle fit for human consumption (types/grades to be fortified)	(2) States at least one type fit for human consumption	White rice - Schedule 2, note after table 3A
2. States the public health objective; purpose and scope of legislation	(0) Does not state	
3. References latest available science or accepted international norms and recommendations, particularly for items that may not be covered in the country's documents	(0) Does not state	Do Australian standards count? They're just for analyses, not for the standards themselves?
4. Provides definitions that includes terms that are specific to fortification (e.g. fortified food, premix, fortificant, food vehicle)	(0) Does not state	
5. Provides repeals (if there is at least one prior document about fortification)	(0) Does not state	
6. Provides effective date or gives grace period for when fortification is to begin (e.g. effective 6 months from signing)	(2) States effective date or grace period for when fortification is to begin (e.g. effective 6 months from signing)	Schedule 8 - Implementation schedule
**MICRONUTRIENTS/PREMIX**		
7. States nutrients required	(2) States nutrients	Schedule 2, Table 3A Note - Thiamin, Niacin, Iron
8. States fortificants (chemical compounds) to be used (including fortificants that are allowable as options)	(2) States fortificants for at least one nutrient	Schedule 2, Table 3B lists permitted forms
9. States fortification levels	(1) States one number only	States min levels
10. States consideration of bioavailability/biological activity of fortificants	(0) Does not state any consideration	
11. States consideration of nutrient stability	(0) Does not state any consideration	
**COSTING**		
12. States that the cost of fortification is regulated through cost sharing schemes (between government, industry, consumers) or tax measures [to assist industry]	(0) Does not state any consideration	
13. States consideration of the financial responsibility of monitoring and enforcing fortification (schedule of fees, budget) [on the government side]	(2) Shows consideration that monitoring costs money	Page 15 - #19?, Page 20 - #29(12)?, page 25 - #38 license fees?
**LABELING**		
14. Includes some sort of statement/label/logo that makes it clear that the product is fortified	(0) Does not include statement, label, or logo	Page 9 - 12(f)quantity of each vitamin, but Page 12 - 13(a) not allowed to claim that the article is food or food additive for specific dietary use.
15. Provides guidance on health claims that can be made for this product (specific to micronutrients added through fortification)	(2) Provides guidance on health claims specific to micronutrients added through fortification	Schedule 2 - 4, 5, 6, 7 RE nutrition claims that can be made
**INTERNAL MONITORING (conducted by industry)**		
16. If samples are to be taken, describes the sampling process: for example, number of samples, amount, frequency, individual vs. composite, where samples are taken in the process, and percent considered passing	(N/A) Does not describe the sampling process	
17. States that industry is required to follow quality assurance/quality control in regards to fortification	(2) States requirement of QA/QC for fortification	States GMP
18. States applicability of using qualitative testing to determine the presence or absence of a vitamin or mineral (spot tests, iChecks)	(0) Does not state	
**EXTERNAL MONITORING (conducted by government)**		
19.States requirement for external monitoring at the production site to assure compliance with standards and regulations	(2) States requirement for external monitoring or the need for audits/inspections	
20. Describes protocols and systems for regulatory monitoring	(1) Does not explicitly describe, but references protocols and systems for regulatory monitoring	Page 14 - 17(2): how inspectors can inspect
21. If there are two or more government agencies involved in external monitoring, clarifies the roles and responsibilities between different government agencies in external monitoring	(0) Clarifies roles and responsibilities for no agencies	
22. Allows for monitoring to be conducted often enough that problems can be identified and addressed on a timely basis; specifies a timeline for inspections (i.e. once every 6 months, increasing to once every 2 months if a discrepancy is found)	(2) Describes frequency and how it is responsive to the needs of industry or the stage of fortification implementation	Schedule 6 - Audit Frequency for various classifications and how they change, likely based on need
23. If samples are to be taken, describes the sampling process: for example, number of samples, amount, frequency, individual vs. composite, where samples are taken in the process, and percent considered passing	(2) States that samples should be taken as part of external monitoring	Page 15 - #20 Sampling
24. States applicability of using qualitative testing to determine the presence or absence of a vitamin or mineral (spot tests, iChecks)	(0) Does not state	
25. States registration is required in order to use a logo/be licensed to produce fortified foods	(2) Describes some type of registration or licensing	Page 25 - #35(a)
**COMMERCIAL MONITORING (conducted by government)**		

26. Provides justification for commercial monitoring at retail stores	(2) Provides justification for commercial monitoring	
27. Describes protocols and systems for commercial monitoring	(1) Does not explicitly describe, but references protocols and systems for regulatory monitoring	Page 14 - 17(1) includes premises where foods are stored, displated for sale, handled, etc.
28. If there are two or more government agencies involved in commercial monitoring, clarifies the roles and responsibilities between different government agencies in commercial monitoring	(0) Clarifies roles and responsibilities for no agencies	
29. Allows for monitoring to be conducted often enough that problems at the production site or import companies can be identified and addressed on a timely basis; specifies a timeline for inspections (i.e. once every 6 months) or works with production companies to correct noncompliance	(2) Describes frequency and how it is responsive to the needs of industry or the stage of fortification implementation	Schedule 6 - Audit Frequency for various classifications and how they change, likely based on need
30. If samples are to be taken, describes the sampling process: for example, number of samples, amount, frequency, individual vs. composite, where samples are taken in the process, and percent considered passing	(2) States that samples should be taken as part of commercial monitoring	
**IMPORT MONITORING (conducted by government)**		
31. Provides justification for import monitoring at points of entry	(2) Provides justification for import monitoring	Page 19 - #29
32. Describes protocols and systems for import monitoring	(2) Includes checklists or detailed description of import monitoring procedures	Page 19 - #29 review of documents, determine extent of inspection and sampling required.
33. If there are two or more government agencies involved in import monitoring, clarifies the roles and responsibilities between different government agencies in import monitoring	(0) Clarifies roles and responsibilities for no agencies	
34. If samples are to be taken, describes the sampling process: for example, number of samples, amount, frequency, individual vs. composite, where samples are taken in the process, and percent considered passing	(2) States that samples should be taken as part of import monitoring	
**ENFORCEMENT/PENALTIES**		
35. Indicates roles and responsibilities in enforcing the legislation	(0) Does not state	
36. States incentives to start fortification	(0) Does not state	
37. States incentives to continue fortification, including ensuring compliance	(0) Does not state	
38. States penalties to compel compliance	(2) States any penalties	4.2.4 - fine not exceeding K10,000 or 50,000 (depending on type of offense), or imprisonment, or both
39. Penalties are objectively defined (e.g. first penalty=$100, second penalty=$300)	(2) Penalties are objectively laid out in the document (e.g. first penalty=$100, second penalty=$300)	
40. States that enforcement is required to include feedback and support to improve performance and correct noncompliance	(0) Does not require	
**LABORATORY**		
41. References required analytical assays for nutrients (e.g. Liquid chromatography-mass spectrometry for folic acid, atomic absorption for iron and zinc.)	(0) Does not state requirements	Page 16 - #23 states analysis info, but nothing specific to nutrients
42. States recognition that laboratory results are subject to several sources of variation and do not provide conclusive evidence of compliance or noncompliance	(0) Does not state recognition	
43. Focuses on the quantitative analysis of "marker" micronutrients such as iron	(0) Does not state	
**REPORTING**		
44. States how government monitoring results are shared with stakeholders	(0) Does not state how results are shared	

Philippines Rice

Element	Scoring (use drop-down menus)	Comments (open text)
**GENERAL**		
1. States that legislation applies to at least one food vehicle fit for human consumption (types/grades to be fortified)	(2) States at least one type fit for human consumption	Sec. 6. Mandatory Food Fortification. - (a) The fortification of staple foods based on standards set by the DOH through the BFAD is hereby made mandatory for the following: (1) Rice - with Iron
2. States the public health objective; purpose and scope of legislation	(2) States the public health objective or general purpose of legislation	SEC. 2. Declaration of Policies. - Section 15 of Article II of the Constitution provides that the State shall protect and promote the right to health of the people and instill health consciousness among them. The state recognizes that nutritional deficiency problems in the Philippines, based on nutrition surveys, include deficiencies in energy, iron, vitamin A, iodine, thiamin and riboflavin. To a minor extent, the Filipino diet is also deficient in ascorbic acid, calcium and folate. The State recognizes that food fortification is vital where there is a demonstrated need to increase the intake of an essential nutrient by one or more population groups, as manifested in dietary, biochemical or clinical evidences of deficiency. Food fortification is considered important in the promotion of optimal health and to compensate for the loss of nutrients due to processing and/or storage of food. Food fortification, therefore, shall be carried out to compensate for the inadequacies in Filipino diet, based on present-day needs as measured using the most recent Recommended Dietary Allowances (RDA).
3. References latest available science or accepted international norms and recommendations, particularly for items that may not be covered in the country's documents	(0) Does not state	The DOH guidelines on micronutrient fortification of processed foods or food products included in Administrative Order No. 4-A series of 1995 and such other necessary guidelines that may be issued by the DOH, shall serve as a basis for the addition of micronutrient(s) to processed foods or food products
4. Provides definitions that includes terms that are specific to fortification (e.g. fortified food, premix, fortificant, food vehicle)	(2) States at least one term related to fortification	Rule III Definition of terms. Section 1. For the purpose of this act, the following terms shall mean: fortification, fortificant, micronutrient, etc.
5. Provides repeals (if there is at least one prior document about fortification)	(2) States repeals	Sec. 14. Repealing Clause. -All laws, decrees, rule and regulations, executive orders inconsistent with the provisions of this Act are hereby repealed or modified accordingly.
6. Provides effective date or gives grace period for when fortification is to begin (e.g. effective 6 months from signing)	(2) States effective date or grace period for when fortification is to begin (e.g. effective 6 months from signing)	SECTION 2. The Implementation of the Mandatory Food Fortification for wheat flour, refined sugar, cooking oil and rice, including those milled and/or distributed by the Natioanl food Authority, shall commence after four years from the effectivity of Rep. Act NO. 8976, which was November 7, 2000, hence the Implementing Rules and Regulation for Mandatory Food Fortification shall take effect on November 7, 2004.
**MICRONUTRIENTS/PREMIX**		
7. States nutrients required	(2) States nutrients	Rule VI, Section 1.1 Table
8. States fortificants (chemical compounds) to be used (including fortificants that are allowable as options)	(2) States fortificants for at least one nutrient	Rule VI, Section 1.1 Table
9. States fortification levels	(2) States a range or number with +/-	Iron (ferrous sulfate) 60 mg Fe/kg raw rice to 90 mg Fe/kg raw rice
10. States consideration of bioavailability/biological activity of fortificants	(0) Does not state any consideration	
11. States consideration of nutrient stability	(2) States consideration of nutrient stability	1. Imposing quality control on the fortificant(s) to ensure that specification are met<br>1.1. require certificate of analysis for every delivery of the fortificant(s)<br>1.2. checking if the fortificant(s) used is still within the market shelf-life
**COSTING**		
12. States that the cost of fortification is regulated through cost sharing schemes (between government, industry, consumers) or tax measures [to assist industry]	(2) States consideration of either cost regulation method	Rule IX-Support to Affected Manufacturers
13. States consideration of the financial responsibility of monitoring and enforcing fortification (schedule of fees, budget) [on the government side]	(2) Shows consideration that monitoring costs money	SECTION 2. The agencies mentioned in Rule VIII and Rule IX mandated to implement and support the food fortification program shall allocate part of their budget for the implementation and support of this program.
**LABELING**		

14. Includes some sort of statement/label/logo that makes it clear that the product is fortified	(2) Includes a statement, label, or logo	Sangkap Pinoy Seal Program – a strategy to encourage manufacturers to fortify processed foods or food products with essential nutrients at levels approved by the DOH. The fundamental concept of the program is to authorize manufacturers to use the DOH seal of acceptance for processed foods or food products, after these products passed a set of defined criteria. The seal is a guide used by consumers in selecting nutritious foods.
15. Provides guidance on health claims that can be made for this product (specific to micronutrients added through fortification)	(0) Does not provide	
**INTERNAL MONITORING (conducted by industry)**		
16. If samples are to be taken, describes the sampling process: for example, number of samples, amount, frequency, individual vs. composite, where samples are taken in the process, and percent considered passing	(N/A) Does not describe the sampling process	
17. States that industry is required to follow quality assurance/quality control in regards to fortification	(2) States requirement of QA/QC for fortification	Quality Assurance. - The agencies charged with the implementation of this Act shall establish a quality assurance system. Likewise, the manufacturers and importers of processed foods or food products shall also establish their own quality assurance system in accordance with the quality assurance system of the implementing agencies.
18. States applicability of using qualitative testing to determine the presence or absence of a vitamin or mineral (spot tests, iChecks)	(0) Does not state	
**EXTERNAL MONITORING (conducted by government)**		
19.States requirement for external monitoring at the production site to assure compliance with standards and regulations	(0) Does not state requirement	
20. Describes protocols and systems for regulatory monitoring	(1) Does not explicitly describe, but references protocols and systems for regulatory monitoring	Rule VIII "SECTION 5. The BFAD shall formulate the standard operating procedures (SOP) for monitoring the implementation of this Act."
21. If there are two or more government agencies involved in external monitoring, clarifies the roles and responsibilities between different government agencies in external monitoring	(2) Clarifies roles and responsibilities for more than one agency	Rule VIII "SECTION 4. The DOH through BFAD, as the lead implementing agency shall be assisted in the monitoring and review of the program by the following:<br>a. Sugar Regulatory Administration (SRA) for sugar; b. National Food Authority (NFA) for rice; c. Philippine Coconut Authority (PCA); d. Bureau of Customs for imported products e. The other agencies enumerated in Rule No. IX.<br>The BFAD shall continue to monitor and review the fortification of wheat flour.<br>SECTION 5. The BFAD shall formulate the standard operating procedures (SOP) for monitoring the implementation of this Act."
22. Allows for monitoring to be conducted often enough that problems can be identified and addressed on a timely basis; specifies a timeline for inspections (i.e. once every 6 months, increasing to once every 2 months if a discrepancy is found)	(0) Does not state	
23. If samples are to be taken, describes the sampling process: for example, number of samples, amount, frequency, individual vs. composite, where samples are taken in the process, and percent considered passing	(N/A) Does not describe the sampling process	
24. States applicability of using qualitative testing to determine the presence or absence of a vitamin or mineral (spot tests, iChecks)	(0) Does not state	
25. States registration is required in order to use a logo/be licensed to produce fortified foods	(2) Describes some type of registration or licensing	SECTION 4. The above named business establishments enumerated in the preceding section except food service establishments shall be duly licensed with the concerned government agencies such as National Food Authority for rice … They shall likewise inform BFAD prior to the process of fortification and they shall register their products with BFAD prior to distribution.
**COMMERCIAL MONITORING (conducted by government)**		

26. Provides justification for commercial monitoring at retail stores	(2) Provides justification for commercial monitoring	SECTION 6. The Local Government Units (LGUs) shall assist in the monitoring of foods mandated to be fortified, in public markets retail stores, and food service establishments. It shall likewise check if the labels of fortified products contain nutrition facts stating the nutrient added and its quantity. The LGUs shall designate only one set from the following officials enumerated in Rep. Act No. 8976, Sec. 8 to conduct the monitoring or checking functions: Head officers or Agricultural officers or Nutritionist-dieticians or Sanitary Inspectors The LGUs shall inform BFAD which of the officers enumerated above has been assigned to monitor the fortified products. The LGUs shall submit reports on monitoring to the Bureau of Food and Drugs (BFAD) in the manner and form prescribed by the latter.
27. Describes protocols and systems for commercial monitoring	(0) Does not describe	
28. If there are two or more government agencies involved in commercial monitoring, clarifies the roles and responsibilities between different government agencies in commercial monitoring	(2) Clarifies roles and responsibilities for more than one agency	SECTION 6. The Local Government Units (LGUs) shall assist in the monitoring of foods mandated to be fortified, in public markets retail stores, and food service establishments. It shall likewise check if the labels of fortified products contain nutrition facts stating the nutrient added and its quantity. The LGUs shall designate only one set from the following officials enumerated in Rep. Act No. 8976, Sec. 8 to conduct the monitoring or checking functions: Head officers or Agricultural officers or Nutritionist-dieticians or Sanitary Inspectors The LGUs shall inform BFAD which of the officers enumerated above has been assigned to monitor the fortified products. The LGUs shall submit reports on monitoring to the Bureau of Food and Drugs (BFAD) in the manner and form prescribed by the latter.
29. Allows for monitoring to be conducted often enough that problems at the production site or import companies can be identified and addressed on a timely basis; specifies a timeline for inspections (i.e. once every 6 months) or works with production companies to correct noncompliance	(0) Does not state	
30. If samples are to be taken, describes the sampling process: for example, number of samples, amount, frequency, individual vs. composite, where samples are taken in the process, and percent considered passing	(N/A) Does not describe the sampling process	
**IMPORT MONITORING (conducted by government)**		
31. Provides justification for import monitoring at points of entry	(2) Provides justification for import monitoring	Imported rice, wheat flour, refined sugar, cooking oil and other processed foods or food products that may be identified later by the NNC, shall comply with the requirements of this Act on entry in the country, at the end of manufacturing process and/or at all points of sale or distribution
32. Describes protocols and systems for import monitoring	(2) Includes checklists or detailed description of import monitoring procedures	RULE VII. "SECTION 1. In accordance with the mandate of Sec. 7 of Rep. Act No. 8976, the agencies responsible for the implementation of this law shall establish a quality assurance system with respect to food fortification. However, manufacturers and importers of processed food or food products or repackers shall also establish their own quality assurance system, which shall conform to the quality assurance system of the implementing agency. Annex 1 is the General Quality Assurance system for Food Fortification to be used as a guide."
33. If there are two or more government agencies involved in import monitoring, clarifies the roles and responsibilities between different government agencies in import monitoring	(2) Clarifies roles and responsibilities for more than one agency	SECITON 8. The Bureau of Customs shall inform the DOH through BFAD of all imported rice, sugar, wheat flour and cooking oil, prior to their release, to enable BFAD to ascertain if such imported products are already fortified. BFAD shall undertake a final inspection that the products are really fortified and registered with BFAD as fortified products prior to sale and distribution
34. If samples are to be taken, describes the sampling process: for example, number of samples, amount, frequency, individual vs. composite, where samples are taken in the process, and percent considered passing	(N/A) Does not describe the sampling process	
**ENFORCEMENT/PENALTIES**		
35. Indicates roles and responsibilities in enforcing the legislation	(2) States the role and responsibilities of government in enforcement	The DOH through the BFAD, after notice and hearing, shall impose any or all of the following administrative sanctions in cases of noncompliance with the food fortification guidelines it has set (denial of registration, recall of food, fine)

36. States incentives to start fortification	(2) States any incentives to encourage fortification initiation (e.g. tax incentives for new equipment or premix)	Section 1. The affected manufacturers shall be supported by the following government agencies in the achievement of the purposes of this act
37. States incentives to continue fortification, including ensuring compliance	(2) States any incentives to encourage the continuation of fortification (e.g. transport priority, favorable tax or tariff treatment, or patent rights)	Section 1. The affected manufacturers shall be supported by the following government agencies in the achievement of the purposes of this act
38. States penalties to compel compliance	(2) States any penalties	Rule XI , Section 1. and Sec. 7. Any person found guilty of violating any provision of this Act shall be punished for each and every offense by an imprisonment of not less than thirty days and not more than six months or a fine of not less than two hundred pesos and not more than two thousand pesos or by both such fine and imprisonment in the discretion of the court.
39. Penalties are objectively defined (e.g. first penalty=$100, second penalty=$300)	(2) Penalties are objectively laid out in the document (e.g. first penalty=$100, second penalty=$300)	Rule XI , Section 1. and Sec. 7. Any person found guilty of violating any provision of this Act shall be punished for each and every offense by an imprisonment of not less than thirty days and not more than six months or a fine of not less than two hundred pesos and not more than two thousand pesos or by both such fine and imprisonment in the discretion of the court.
40. States that enforcement is required to include feedback and support to improve performance and correct noncompliance	(0) Does not require	
**LABORATORY**		
41. References required analytical assays for nutrients (e.g. Liquid chromatography-mass spectrometry for folic acid, atomic absorption for iron and zinc.)	(0) Does not state requirements	
42. States recognition that laboratory results are subject to several sources of variation and do not provide conclusive evidence of compliance or noncompliance	(0) Does not state recognition	
43. Focuses on the quantitative analysis of "marker" micronutrients such as iron	(0) Does not state	
**REPORTING**		
44. States how government monitoring results are shared with stakeholders	(0) Does not state how results are shared	The local food industries shall report on the production, marketing and distribution of fortified foods. They shall submit annual reports to the DOH, also indicating their industrial concerns and recommendations.

United States Rice

Element	Scoring (use drop-down menus)	Comments (open text)
**GENERAL**		
1. States that legislation applies to at least one food vehicle fit for human consumption (types/grades to be fortified)	(2) States at least one type fit for human consumption	(a) The foods for which definitions and standards of identity are prescribed by this section are forms of milled rice (except rice coated with talc and glucose and known as coated rice), to which nutrients have been added so that each pound of the rice contains:
2. States the public health objective; purpose and scope of legislation	(2) States the public health objective or general purpose of legislation	The achievement and maintenance of a desirable level of nutritional quality in the nation's food supply is an important public health objective.
3. References latest available science or accepted international norms and recommendations, particularly for items that may not be covered in the country's documents	(2) States the documents referenced	The Food and Drug Administration (FDA) is amending the food additive regulations to provide for the safe use of folic acid in foods that are the subject of a standard of identity that requires the addition of folic acid; to provide for its addition to breakfast cereals on a per serving basis; to permit its use in infant formulas, medical foods, and foods for special dietary use; and to incorporate specifications for folic acid consistent with those in the Food Chemicals Codex.
4. Provides definitions that includes terms that are specific to fortification (e.g. fortified food, premix, fortificant, food vehicle)	(2) States at least one term related to fortification	The term "essential nutrient" under the fortification policy refers to the vitamins and minerals assigned Reference Daily Intakes (RDIs) listed in 21 CFR 101.9(c)(8)(iv), as well as protein and potassium that have daily reference values (DRVs) (21 CFR 101.9(c)(9) or 21 CFR 104.20(d)(3)).
5. Provides repeals (if there is at least one prior document about fortification)	(2) States repeals	In the Federal Register of October 14, 1993 (58 FR 53312), FDA proposed to amend the regulation that establishes safe conditions of food use for folic acid, § 172.345 (21 CFR 172.345)).
6. Provides effective date or gives grace period for when fortification is to begin (e.g. effective 6 months from signing)	(2) States effective date or grace period for when fortification is to begin (e.g. effective 6 months from signing)	EFFECTIVE DATE: January 1, 1998. Many comments expressed concern over the statement in the standards of identity proposal that the final rule would become effective 1 year after publication.
**MICRONUTRIENTS/PREMIX**		
7. States nutrients required	(2) States nutrients	Not less than 2.0 milligrams and not more than 4.0 mg of thiamin, not less than 1.2 mg and not more than 2.4 mg of riboflavin, not less than 16 mg and not more than 32 mg of niacin or niacinamide, not less than 0.7 mg and not more than 1.4 mg of folic acid, and not less than 13 mg and not more than 26 mg of iron (Fe).
8. States fortificants (chemical compounds) to be used (including fortificants that are allowable as options)	(2) States fortificants for at least one nutrient	Not less than 2.0 milligrams and not more than 4.0 mg of thiamin, not less than 1.2 mg and not more than 2.4 mg of riboflavin, not less than 16 mg and not more than 32 mg of niacin or niacinamide, not less than 0.7 mg and not more than 1.4 mg of folic acid, and not less than 13 mg and not more than 26 mg of iron (Fe).
9. States fortification levels	(2) States a range or number with +/-	Not less than 2.0 milligrams and not more than 4.0 mg of thiamin, not less than 1.2 mg and not more than 2.4 mg of riboflavin, not less than 16 mg and not more than 32 mg of niacin or niacinamide, not less than 0.7 mg and not more than 1.4 mg of folic acid, and not less than 13 mg and not more than 26 mg of iron (Fe).
10. States consideration of bioavailability/biological activity of fortificants	(2) States some consideration of bioavailability (mentions these or related terms)	With respect to issues of bioavailability, FDA concluded that bioavailability cannot be meaningfully factored into fortification scenarios because issues of bioavailability are very complex, and no systematic data are available on many of the factors that affect bioavailability.

11. States consideration of nutrient stability	(2) States consideration of nutrient stability	(c) The requirement of paragraphs (a) and (b) of this section will be deemed to have been met if reasonable overages of the vitamins and minerals, within the limits of good manufacturing practice, are present to insure that the required levels of the vitamins and minerals are maintained throughout the expected shelf life of the food under customary conditions of distribution and storage. Review food additives and enrichment in storage to determine if allowed in standards (21 CFR 137). Calculate percentages or amounts in the finished product per CWT (hundred weight or 100 lbs.) of flour (remember to be consistent with the units utilized in the standards.). Enrichment held for a year or more, or held at abnormally high temperatures or humidity, may have deteriorated.
**COSTING**		
12. States that the cost of fortification is regulated through cost sharing schemes (between government, industry, consumers) or tax measures [to assist industry]	(2) States consideration of either cost regulation method	c. Reformulation costs. Reformulation costs associated with this option were estimated in the proposal to be $27 million for the first year. The cost of adding the required folic acid is approximately $4 million per year. The cost of testing was estimated to be about $2.5 million per year and the cost of the required label changes $20 million. FDA will use these costs for this final rule as no comments were received on this part of the analysis.
13. States consideration of the financial responsibility of monitoring and enforcing fortification (schedule of fees, budget) [on the government side]	(2) Shows consideration that monitoring costs money	c. Reformulation costs. Reformulation costs associated with this option were estimated in the proposal to be $27 million for the first year. The cost of adding the required folic acid is approximately $4 million per year. The cost of testing was estimated to be about $2.5 million per year and the cost of the required label changes $20 million. FDA will use these costs for this final rule as no comments were received on this part of the analysis.
**LABELING**		
14. Includes some sort of statement/label/logo that makes it clear that the product is fortified	(2) Includes a statement, label, or logo	(g) Label declaration. Each of the ingredients used in the food shall be declared on the label as required by the applicable sections of parts 101 and 130 of this chapter. but, if insufficient additional calcium is present to meet the 960 milligram level, no claim may be made on the label for calcium as a nutrient; (d) Model health claims. The following are examples of model health claims that may be used in food labeling to describe the relationship between folate and neural tube defects: (1) Examples 1 and 2. Model health claims appropriate for foods containing 100 percent or less of the DV for folate per serving or per unit (general population). The examples contain only the required elements:
15. Provides guidance on health claims that can be made for this product (specific to micronutrients added through fortification)	(2) Provides guidance on health claims specific to micronutrients added through fortification	(i) Healthful diets with adequate folate may reduce a woman's risk of having a child with
**INTERNAL MONITORING (conducted by industry)**		
16. If samples are to be taken, describes the sampling process: for example, number of samples, amount, frequency, individual vs. composite, where samples are taken in the process, and percent considered passing	(N/A) Does not describe the sampling process	
17. States that industry is required to follow quality assurance/quality control in regards to fortification	(0) Does not state requirement	
18. States applicability of using qualitative testing to determine the presence or absence of a vitamin or mineral (spot tests, iChecks)	(0) Does not state	
**EXTERNAL MONITORING (conducted by government)**		

19.States requirement for external monitoring at the production site to assure compliance with standards and regulations	(0) Does not state requirement	
20. Describes protocols and systems for regulatory monitoring	(0) Does not describe	
21. If there are two or more government agencies involved in external monitoring, clarifies the roles and responsibilities between different government agencies in external monitoring	(1) Clarifies roles and responsibilities for one agency	
22. Allows for monitoring to be conducted often enough that problems can be identified and addressed on a timely basis; specifies a timeline for inspections (i.e. once every 6 months, increasing to once every 2 months if a discrepancy is found)	(0) Does not state	
23. If samples are to be taken, describes the sampling process: for example, number of samples, amount, frequency, individual vs. composite, where samples are taken in the process, and percent considered passing	(1) States that samples should be taken (generally)	Vitamins and minerals - Samples will be collected on assignment basis or if inspectional conditions warrant.<br>1. Retail Packages 4.5kg (10 lbs or less) -Collect twelve intact packages in duplicate of the same code.<br>2. Bulk and Retail Packages 4.5kg (Larger than 10 lbs) - Collect 12 - 453 gm (1 lb) subs in duplicate from each of twelve different containers of the same code. Remove flour or other cereal product with a trier inserted the full length of the container. Prepare each sub in individual airtight containers and protect against sunlight by wrapping in opaque paper. Do not fumigate samples.
24. States applicability of using qualitative testing to determine the presence or absence of a vitamin or mineral (spot tests, iChecks)	(0) Does not state	
25. States registration is required in order to use a logo/be licensed to produce fortified foods	(0) Does not state that registration or licensing is required	
**COMMERCIAL MONITORING (conducted by government)**		
26. Provides justification for commercial monitoring at retail stores	(0) Does not provide justification for commercial monitoring	
27. Describes protocols and systems for commercial monitoring	(0) Does not describe	
28. If there are two or more government agencies involved in commercial monitoring, clarifies the roles and responsibilities between different government agencies in commercial monitoring	(0) Clarifies roles and responsibilities for no agencies	
29. Allows for monitoring to be conducted often enough that problems at the production site or import companies can be identified and addressed on a timely basis; specifies a timeline for inspections (i.e. once every 6 months) or works with production companies to correct noncompliance	(N/A) No commercial monitoring occurs	
30. If samples are to be taken, describes the sampling process: for example, number of samples, amount, frequency, individual vs. composite, where samples are taken in the process, and percent considered passing	(1) States that samples should be taken (generally)	Enrichment, Minerals and Nutrition Labeling - Bread, rolls, baked or unbaked product: Compliance samples should consist of 12 subsamples of at least 1 lb. each (454 gm) collected in duplicate. Collect consumer size retail packages (2 - 1 lb. subs from each of 12 cases) from separate cases of the same code. Protect enrichment samples from sun by wrapping in opaque paper. Determine and record by sub the gross weight.
**IMPORT MONITORING (conducted by government)**		
31. Provides justification for import monitoring at points of entry	(0) Does not provide justification for import monitoring	
32. Describes protocols and systems for import monitoring	(2) Includes checklists or detailed description of import monitoring procedures	
33. If there are two or more government agencies involved in import monitoring, clarifies the roles and responsibilities between different government agencies in import monitoring	(2) Clarifies roles and responsibilities for more than one agency	The purpose of this chapter is to provide an overview of import procedures for articles subject to the laws and regulations enforced by the Food and Drug Administration (FDA). The chapter also includes an overview of laws and regulations enforced by U.S. Customs and Border Protection (CBP), as they relate to importation of articles regulated by FDA.<br>Division of Authority section

34. If samples are to be taken, describes the sampling process: for example, number of samples, amount, frequency, individual vs. composite, where samples are taken in the process, and percent considered passing	(1) States that samples should be taken (generally)	additional documentation is made, FDA will take appropriate action. If FDA decides to collect a sample, it will provide the filer, importer, owner and/or consignee, where appropriate, with a Notice of Sampling and advise: (1) whether the entry is to be held intact for FDA examination or sampling; or, (2) specify only those items that need be held. Generally, when FDA wishes to sample, it will be acting upon its authority in the Customs regulations (19 CFR 151.4) to collect its own samples for examination. FDA may request an examination or sample of articles under its jurisdiction. If no examination or sample is requested, FDA will so notify CBP and the filer, who is responsible for notifying the importer or other designated parties. This is referred to as a "May Proceed Notice," and indicates that the shipment may proceed without further FDA examination. In the ACS/OASIS process this may occur as a result of the initial FDA/OASIS screening prior to the information being forwarded to the district office, or after the district performs an "On-Screen-Review" of the information provided. (Note: Should the article, at a later time, be found in violation of the law, FDA is not prevented from taking legal action (e.g., seizure, injunction) because it allowed admission of the article without examination at the time of importation.) If FDA requests an examination or sample, FDA will notify CBP and the broker or filer, importer, or other
**ENFORCEMENT/PENALTIES**		
35. Indicates roles and responsibilities in enforcing the legislation	(2) States the role and responsibilities of government in enforcement	The purpose of this chapter is to provide an overview of import procedures for articles subject to the laws and regulations enforced by the Food and Drug Administration (FDA). The chapter also includes an overview of laws and regulations enforced by U.S. Customs and Border Protection (CBP), as they relate to importation of articles regulated by FDA.
36. States incentives to start fortification	(0) Does not state	
37. States incentives to continue fortification, including ensuring compliance	(0) Does not state	
38. States penalties to compel compliance	(2) States any penalties	Consequently, FDA may issue a warning letter and take enforcement action if a marketed food bearing one or more of these nutrient content claims contains a nutrient addition that is inconsistent with the fortification policy as incorporated into the regulations noted above. Nevertheless, FDA strongly encourages you to follow these fortification guidelines regardless of whether any claims appear on the label or in labeling.
39. Penalties are objectively defined (e.g. first penalty=$100, second penalty=$300)	(N/A) No penalties are stated (Answered (0) to previous question)	
40. States that enforcement is required to include feedback and support to improve performance and correct noncompliance	(0) Does not require	
**LABORATORY**		
41. References required analytical assays for nutrients (e.g. Liquid chromatography-mass spectrometry for folic acid, atomic absorption for iron and zinc.)	(2) References required assays	The quantitative content of the following vitamins shall be calculated in terms of the following chemically identifiable reference forms: Vitamin Reference form Name Empirical formula Molecular weight Thiamine ... Thiamine chloride hydrochloride. $C_{12}H_{17}ClN_4OS{\cdot}HCl$ 337.28 Riboflavin .. Riboflavin .... $C_{17}H_{20}N_4O_6$ 376.37 Niacin .... Niacin .... $C_6H_5NO_2$ 123.11 (c) The method referred to in paragraph (a) of this section is the method prescribed in ‘‘Official Methods of Analysis of the Association of Official Analytical Chemists’’ (AOAC), 13th Ed. (1980), section 8.002, ‘‘Reagent (Displacement soln.),’’

42. States recognition that laboratory results are subject to several sources of variation and do not provide conclusive evidence of compliance or noncompliance	(0) Does not state recognition
43. Focuses on the quantitative analysis of "marker" micronutrients such as iron	(0) Does not state
**REPORTING**	
44. States how government monitoring results are shared with stakeholders	(0) Does not state how results are shared
