# Supplementary material for: Review of Grain Fortification Legislation, Standards, and Monitoring Documents
Source: Glob Health Sci Pract. 2018 Jun 27;6(2):356–71. doi: 10.9745/GHSP-D-17-00427 (PMC6024620; doi:10.9745/GHSP-D-17-00427)
Supplement: 17-00427-Marks-SupplementTable3.pdf [file 17-00427-Marks-SupplementTable3.pdf]

Marks KJ, Luthringer CL, Ruth LJ, et al. Review of grain fortification legislation, standards, and monitoring documents. *Glob Health Sci Pract*. 2018;6(2). https://doi.org/10.9745/GHSP-D-17-00427

SUPPLEMENT TABLE 3. Example Language for Each Item, Extracted From Document Reviews*,**

Item	Example Language	Country	Reference
General			
1. States that legislation applies to at least one food vehicle fit for human consumption (types/grades to be fortified)	This Technical Regulation applies to the mandatory fortification of wheat flour and corn flour with the iron and folic acid. Excluded from this regulation, due to technological processing, are the following products: tapioca flour or corn flour obtained by maceration; flakes; whole wheat flour and durum wheat flour.	Brazil	National Health Surveillance Agency (ANVISA). RDC Resolution No. 344. Approve the technical regulations for the wheat flour and corn flour fortification with iron and folic acid, found in the annex to this resolution. Brazil: Official Federal Gazette. 2002.
2. States the public health objective; purpose and scope of legislation	The State shall protect and promote the right to health of the people and instill health consciousness among them. The state recognizes that nutritional deficiency problems in the Philippines, based on nutrition surveys, include deficiencies in energy, iron, vitamin A, iodine, thiamin and riboflavin...The State recognizes that food fortification is vital where there is a demonstrated need to increase the intake of an essential nutrient by one or more population groups, as manifested in dietary, biochemical or clinical evidences of deficiency. Food fortification is considered important in the promotion of optimal health and to compensate for the loss of nutrients due to processing and/or storage of food. Food fortification, therefore, shall be carried out to compensate for the inadequacies in Filipino diet.	Philippines	Department of Health. Office of the Secretary. The implementing rules and regulations of republic act no. 8976. Entitled: "An Act Establishing the Philippine Food Fortification Program and for Other Purposes". Manila, Philippines: Department of Health. n.d.

Item	Example Language	Country	Reference
3. References latest available science or accepted international norms and recommendations, particularly for items that may not be covered in the country's documents	In establishing standards for food, the Ministries shall take into account fully the recommended international standards of the Codex Alimentarius Commission, including those related to fortification of foods.	Liberia	The National Fortification Alliance of Liberia (NFA). Guidelines of the National Fortification Alliance of Liberia. Monrovia, Liberia: NFA. n.d.
4. Provides definitions that includes terms that are specific to fortification (e.g. fortified food, premix, fortificant, food vehicle)	As used in this Regulation, the following terms shall be given the meanings described below: "Fortified food" or "enriched food" means any food to which one or more essential nutrients, such as vitamins, minerals, proteins, essential amino or fatty acids, or other nutritional substances have been added in order to increase the nutritive value of the food and which are absent from the food in its original state or which are lost during normal manufacturing, storage, or handling.	Liberia	The National Fortification Alliance of Liberia (NFA). Guidelines of the National Fortification Alliance of Liberia. Monrovia, Liberia: NFA. n.d.
5. Provides repeals (if there is at least one prior document about fortification)	"Within six months of the declaration of an East African Standard, the Partner States shall adopt, without deviation from the approved text of the standard, the East African Standard as a national standard and withdraw any existing national standard with similar scope and purpose."	East African Standard	Final Draft East African Standard, FDEAS 767:2012, Fortified wheat flour--specification
6. Provides effective date or gives grace period for when fortification is to begin (e.g. effective 6 months from signing)	The mandatory Standard provides industry with two years to implement requirements and is enforceable from 13 September 2009.	Australia	Food Standards Australia New Zealand (FSANZ). Australian user guide. Mandatory folic acid fortification. Implementing the requirements of the mandatory fortification with folic acid under Standard 2.1.1 – cereals and cereal products. (n.p.): FSANZ. 2009.
Micronutrients/premix			

Item	Example Language	Country	Reference
7. States nutrients required	Wheat flour for making bread must contain – (a) no less than 2 mg/kg and no more than 3 mg/kg of folic acid; and (b) no less than 6.4 mg/kg of thiamin.	Australia	Federal Register of Legislative Instruments F2014C01190. Standard 2.1.1 cereals and cereal products. Australia: Author. 2014.
8. States fortificants (chemical compounds) to be used (including fortificants that are allowable as options)	In the fortification of flour with iron and folic acid food grade ferrous sulphate should be used 60 ppm or 60 ppm ferrous fumarate or ethylene acid diamine tetra-acetic acid / sodium EDTA and iron (NaFeEDTA) in 40ppm folic acid and 2,6 ppm.	Cape Verde	The Republic of Cape Verde. Official Bulletin. Series I Number 46. Cape Verde: Cape Verdean National Press. SA. 2014.
9. States fortification levels	Wheat flour for making bread must contain – (a) no less than 2 mg/kg and no more than 3 mg/kg of folic acid; and (b) no less than 6.4 mg/kg of thiamin.	Australia	Federal Register of Legislative Instruments F2014C01190. Standard 2.1.1 cereals and cereal products. Australia: Author. 2014.
10. States consideration of bioavailability/biological activity of fortificants	Companies should ensure that the food grade iron compounds are bioavailable. Bio-availability- this is the proportion of the ingredient ingested that is available for the metabolic process and varies for each type of iron. Ferrous sulfate and fumarate have good bio-availability, however, elemental iron is considered lower.	Brazil	National Health Surveillance Agency (ANVISA). RDC Resolution No. 344. Approve the technical regulations for the wheat flour and corn flour fortification with iron and folic acid, found in the annex to this resolution. Brazil: Official Federal Gazette. 2002.
11. States consideration of nutrient stability	The Vitamin fortificants and premixes shall have storage stability such that no more than 20 % of its original activity will be lost when stored for 21 days at 45 °C in a well closed container at a level 2.5g per kg in wheat flour having moisture content in the range of 13.5 % - 14.5 %.	East African Standard	Final Draft East African Standard, FDEAS 767:2012, Fortified wheat flour--specification
Costing			

Item	Example Language	Country	Reference
12. States that the cost of fortification is regulated through cost sharing schemes (between government, industry, consumers) or tax measures [to assist industry]	Take measures on economic stimulus (provision of economic entities with privileges, that are envisaged by customs and tax legislation of the Kyrgyz Republic) and provide economic stimulus to legal entities and physical persons producing fortified flour	Kyrgyzstan	President of the Kyrgyz Republic. Law of the Kyrgyz Republic on fortification of baking flour. Kyrgyzstan: Author. 2009.
13. States consideration of the financial responsibility of monitoring and enforcing fortification (schedule of fees, budget) [on the government side]	Actions of the program shall be covered financially from allocations approved yearly in the national public budget and other sources...Expenses for examination, storage, transporrtation, use, or destruction of food that is non-compliant is paid by the food industry	Republic of Moldova	Decree on the approval of measures to reduce by 2017 disorders determined by iron and folic acid deficiency. No. 171. Chisinau, Republic of Moldova: n.p. 2012.
Labeling			
14. Includes some sort of statement/label/logo that makes it clear that the product is fortified	All products designated as fortified should bare the ENRICHED logo in accordance with the logo guidelines.	Liberia	The National Fortification Alliance of Liberia (NFA). Guidelines of the National Fortification Alliance of Liberia. Monrovia, Liberia: NFA. n.d.
15. Provides guidance on health claims that can be made for this product (specific to micronutrients added through fortification)	All food vehicles shall be labelled as follows:<br>(a) the claim “enriched with” or “enriched” may only be used in addition to the word “fortified” on one label in cases where a micronutrient other than the specified fortificants is added to a food vehicle or in cases where at least 15% more than the prescribed amounts of fortificants are added to a food vehicle;<br>(b) the claim “Fortified for better health” and the official fortification logo to that effect as indicated in Annexure VII are reserved only for food vehicles, that have been identified in these regulations and may be displayed on the label or in an advertising material	South Africa	Department of Health. No. R 7634. Foodstuffs, cosmetics, and disinfectants Act, 1972 (Act No. 54 of 1972). Regulations relating to the fortification of certain foodstuffs. (n.p.): Government notice. 2003.

Item	Example Language	Country	Reference
Internal monitoring (conducted by industry)			
16. If samples are to be taken, describes the sampling process: for example, number of samples, amount, frequency, individual vs. composite, where samples are taken in the process, and percent considered passing	Quality control 6.2.1. Sampling. All flour samples should be collected after they have been packaged. The random collection of at least 3 (three) packages (of 1 or 5kg) from each -sample is recommended. For 50kg sacks, a sample of about 1kg can be taken from 3 sacks, or directly from the mouth of the bagger, for non-automatic systems.	Brazil	Germani, R., Ascheri, J. L. R., Silva, F. T., Torrezan, R., Silva, K. L., Netto, A. G., & Nutti, M. R. Manual for fortification of wheat flour with iron. Documents 46. Rio de Janeiro, Brazil: Embrapa Agroindústria de Alimentos. 2001.
17. States that industry is required to follow quality assurance/quality control in regards to fortification	Manufacturer of a fortified food shall develop, maintain and routinely follow procedures for safety and quality assurance throughout the manufacturing process to ensure that the final product complies with these regulations and any other regulations made under the Act.	Tanzania	Tanzania Food and Drugs Authority (TFDA). Guidelines for conducting external monitoring food fortification (1st ed.). Dar es Salaam, Tanzania: TFDA. 2012.
18. States applicability of using qualitative testing to determine the presence or absence of a vitamin or mineral (spot tests, iChecks)	Qualitative tests are simple and rapid tests that can be done at the mill to determine if a flour sample has been fortified or not. The primary test used for this purpose is the Iron Spot Test. The iron spot test when properly done with known fortified flour samples afford a rough estimate on the level of fortification in an unknown sample. This is the most common test used by mills for quality control to assure that a correct amount of premix is being added to flour and a uniform product is being achieved.	Egypt	Global Alliance for Improved Nutrition, United Nations World Food Programme. Quality Assurance (QA) & Quality Control (QC). Rome: Global Alliance for Improved Nutrition, United Nations World Food Programme. 2010.
External monitoring (conducted by government)			

Item	Example Language	Country	Reference
19. States requirement for external monitoring at the production site to assure compliance with standards and regulations	Technical auditing and inspection activities are carried out at manufacturing facilities as part of the enforcement activities performed by the regulatory authority in order to ensure that fortified foods meets the nutrient quality and safety specifications established in regulations.	Tanzania	Tanzania Food and Drugs Authority (TFDA). Guidelines for conducting external monitoring food fortification (1st ed.). Dar es Salaam, Tanzania: TFDA. 2012.
20. Describes protocols and systems for regulatory monitoring	Procedure<br>During the inspection, inspector(s) shall follow the procedures stipulated here under:-<br>Conducting opening session<br>a) Inspectors shall conduct opening meeting with the factory management which includes General Manager, Factory or Production Manager, Quality Assurance and Control department manager and Laboratory Manager.<br>b) During the meeting inspectors shall explain briefly the purpose and approximate duration of the inspection and that this will be carried out through reviewing of written procedures, records, personnel interviews, observation of the fortification process and taking some samples.<br>c) Inspector shall inquire from factory management if there are problems that are associated with the implementation of food fortification.<br>d) Inspector shall record name of attendants to the session in a format prescribed in form F1/TFDA/DFS/FI&E/GL/001 of these guidelines<br>Conducting technical audit and inspection<br>The inspector (s) shall:-<br>a) Conduct technical audit and inspection with the aid of the checklist prescribed in form F2/TFDA/DFS/FI&E/GL/001 or F3/TFDA/DFS/FI&E/GL/001 of these guidelines. As the audit and inspection takes place, record any non-compliance.<br>b) Review the non-compliances found during the last	Tanzania	Tanzania Food and Drugs Authority (TFDA). Guidelines for conducting external monitoring food fortification (1st ed.). Dar es Salaam, Tanzania: TFDA. 2012.

Item	Example Language	Country	Reference
	inspection (if any) and recommendations made. Assess the corrective actions and record the findings.<br>Sampling<br>At the end of audit and inspection, inspector shall take samples by using procedures stipulated under section 3 of these guidelines.<br>Preparation of preliminary report<br>Inspectors shall plan to dedicate adequate time to prepare the preliminary report on the major findings during the audit and inspection inspection. In the report, provide comments regarding the performance of the quality assurance and control procedures, opportunities for improvement and non-compliance if any in a format prescribed in from F4/TFDA/DFS/FI&E/GL/001 of these guidelines.		

Item	Example Language	Country	Reference
21. If there are two or more government agencies involved in external monitoring, clarifies the roles and responsibilities between different government agencies in external monitoring	This manual was developed by the officers of the Directorate of Internal Trade in charge of the quality control of food products. It is an important work tool (logbook) for all employees in the quality control of fortified products. Thus, the central services of the DCI and the decentralized departments will appropriate this reference document to harmonize interventions on the one hand and improve the control effectiveness on the other...The quality control of oils and fortified flour is naturally in the food quality control provided by the Ministry for Trade through the Division of Consumer Affairs and Consumer Safety (DCSC) and decentralized Internal Trade services (regional and departmental services). In total, there are 14 regional services and 20 departmental services for Commerce across the country. It should be noted that this control is carried out in collaboration with other jurisdictions such as the Directorate of Plant Protection (DPV), the Directorate of Veterinary Services (DSV), the Customs Directorate (DGD), etc.	Senegal	Ministry of Commerce, Industry and Informal Sector, ITA, The Senegalese Committee for the Fortification of Food in Micronutrients (COSFAM), & Micronutrient Initiative (MI). Manual of Procedures. Quality control of fortified oils and flours. (n.p.): Author. 2012.
22. Allows for monitoring to be conducted often enough that problems can be identified and addressed on a timely basis; specifies a timeline for inspections (i.e. once every 6 months, increasing to once every 2 months if a discrepancy is found)	Authorized officers shall conduct inspections of food premises in line with the risk associated with the food being produced, processed, handled, stored, displayed, transported or sold and in accordance with the history of compliance or otherwise as outlined in the Twenty-eighth Schedule.	Fiji	Food Safety Regulations 2009. Legal Notice No. 20. Food and Safety Act 2003 (No. 10 of 2003). Fiji Islands: n.p. n.d.

23. If samples are to be taken, describes the sampling process: for example, number of samples, amount, frequency, individual vs. composite, where samples are taken in the process, and percent considered passing	Procedure for sampling at maize and wheat flour manufacturing facilities<br>Fortificant/ Premix<br>Take a 50g sample of the fortificant/premix that is being used for fortification at the factory during the time of inspection. Label it with the name of the mill, name of the manufacturer, micronutrient content indicated, and date of sample collection.<br>Daily composite samples<br>a) Before the inspection inspection ends, go to the laboratory and check that "daily composite samples" for the last 30 working days are appropriately stored.<br>b) Choose three daily composite samples at random. In form F6/TFDA/DFS/FI&E/GL/001 of these guidelines write down the production date, estimated iron level, and any other information provided on the sample identification.<br>Samples from production or storage warehouse<br>Take two more samples per type of flour being produced that day or from the storage warehouse.<br>Samples from production<br>a) In the packaging area, the inspector should take 500 g of flour from any bag before weighing and sealing.<br>b) Repeat step (a) every 10 minutes until 8 samples have been collected.<br>c) Ask personnel of the mill to help for the verification of the presence of iron in each sample, using the spot-test method.<br>d) Mix equal amounts of each of the 8 samples to produce a composite sample from production.<br>Samples from storage warehouse<br>a) Collect 8 samples of 500g each from stores warehouse by selecting bags at random.<br>b) Verify the presence of iron in each sample.<br>c) Combine and mix well the 8 samples to produce a composite sample from store.	Tanzania	Tanzania Food and Drugs Authority (TFDA). Guidelines for conducting external monitoring food fortification (1st ed.). Dar es Salaam, Tanzania: TFDA. 2012.

Item	Example Language	Country	Reference
24. States applicability of using qualitative testing to determine the presence or absence of a vitamin or mineral (spot tests, iChecks)	Take samples of approved fortified foods and conduct spot test for iron and where necessary, take samples for quantitative analysis at TFDA head quarters. Ask personnel of the mill to help for the verification of the presence of iron in each sample, using the spot-test method.	Tanzania	Tanzania Food and Drugs Authority (TFDA). Guidelines for conducting external monitoring food fortification (1st ed.). Dar es Salaam, Tanzania: TFDA. 2012.
25. States registration is required in order to use a logo/be licensed to produce fortified foods	State registration of foods and materials for food packaging (hereinafter - state registration) - a procedure which is carried out by the competent health authorities to determine the compliance of foods, materials for food packaging with the requirements of normative legal acts of Turkmenistan, as well as regulations. The aim is to prevent the state registration of production, circulation of food products and materials for food packaging that pose a threat to human life and health, his heredity.	Turkmenis tan	Law of Turkmenistan. On ensuring the safety and quality of food products. Ashgabat, Turkmenistan: n.p. 2014.

Item	Example Language	Country	Reference
Commercial monitoring (conducted by government)			
26. Provides justification for commercial monitoring at retail stores	Commercial inspection is the verification of legal compliance of fortified foods sold in retail supermarkets, markets, grocery stores, and wholesale stores. It also includes inspection at bakeries as a convenient sampling site for fortified foods namely salt, sugar, flour and cooking oil. This monitoring allows for the detection in the market of brands that are not approved by the Ministry of Health or do not comply with local fortification regulations. It also helps to confirm whether brands that have previously been inspected in factories and importation sites are indeed fulfilling the requirements as claimed by inspectors during the external monitoring process. Furthermore, commercial monitoring serves as an education tool since inspectors are able to inform the retailers about the existence of the fortification program, the benefits of fortification, their role as retailers, and their rights as consumers.	Liberia	The National Fortification Alliance of Liberia (NFA). Guidelines of the National Fortification Alliance of Liberia. Monrovia, Liberia: NFA. n.d.

27. Describes protocols and systems for commercial monitoring	Procedures<br>During the inspection, inspector(s) shall follow the procedures stipulated here under:-<br>a) When inspectors arrive in the villages, towns or cities, they should inspection the most popular food outlets where people buy their food supplies.<br>b) Inspectors should enter the store and show their credentials identifying them as inspectors of the Authority. They should follow on with a brief explanation about the purpose of the inspection.<br>c) Inspectors should be able to identify the approved fortified brands sold in the store by using format prescribed in form F5/TFDA/DFS/FI&E/GL/001 of these guidelines.<br>d) Take samples of approved fortified foods and conduct spot test for iron and where necessary, take samples for quantitative analysis at TFDA head quarters. They should fill in the form F5/TFDA/DFS/FI&E/GL/001 of these guidelines<br>e) Choose a sealed packaged of about 500gm or 500ml of each brand of each fortified food in the store. If the food is not available in such quantities, take the nearest larger retail-size presentation. If packages are much smaller, collect sufficient packages to make up the specified weight; (e.g. 2 packages of 250 gm).<br>f) If the food is sold by weight or volume from large sacks or a barrel, take approximately 500gm or 500ml sample from this food product.<br>g) Ensure that the sack or the barrel is new; otherwise there is no guarantee that the product inside corresponds to the factory name in the container.<br>h) Mark the samples appropriately, pack in a box and transport them to the local Food Control office, which in turn sends copies of report and the samples to the Authority on quarterly basis.	Tanzania	Tanzania Food and Drugs Authority (TFDA). Guidelines for conducting external monitoring food fortification (1st ed.). Dar es Salaam, Tanzania: TFDA. 2012.

Item	Example Language	Country	Reference
28. If there are two or more government agencies involved in commercial monitoring, clarifies the roles and responsibilities between different government agencies in commercial monitoring	The Food Control Authority inspectors or municipal/district health inspectors are responsible for checking compliance of packaging and labeling, and for taking samples of the foods for analysis. They should report on the results of their visits to their supervisor. The supervisor is then responsible of sending samples to the central office, and for consolidating the reports on the findings and reporting every month to the Head of the Food Control Authority. In turn, the Food Control Authority Should prepare consolidated reports every 6 months and send to other government and auxiliary bodies, such as the National Food Fortification Alliance, involved in the supervision of the food fortification programs.	Uganda	Ministry of Health, & Uganda National Bureau of Standards. Manual for regulatory monitoring of fortified foods. Kampala, Uganda: n.p. n.d.
29. Allows for monitoring to be conducted often enough that problems at the production site or import companies can be identified and addressed on a timely basis; specifies a timeline for inspections (i.e. once every 6 months) or works with production companies to correct noncompliance	Plan at least one visit a year to each region based on the total number of districts in the coutnry...Frequency and intensity of sampling depends on the population density, amount of food sold in the region, and risk factors such as location close to the borders with other countries where the food is not fortified...When a brand does not meet the minimum legal requirements (micronutrient content, labeling and packaging) depicted in the fortification regulations, a warning letter shall be sent to the factors, packaging plant or importer responsible for the brand. Sampling priority should be given to these brands in future visits. Extra visits to the factories might be considered within the external monitoring activities.	Uganda	Ministry of Health, & Uganda National Bureau of Standards. Manual for regulatory monitoring of fortified foods. Kampala, Uganda: n.p. n.d.

Item	Example Language	Country	Reference
30. If samples are to be taken, describes the sampling process: for example, number of samples, amount, frequency, individual vs. composite, where samples are taken in the process, and percent considered passing	In taking samples of wheat flour, the following things should be taken into account:<br>- Samplings are conducted for any wheat flour trade name in circulation; samples are randomly picked from retailers/ vendors/ agents/ distributors.<br>- Samples are taken at districts/ cities periodically or as programmed by stakeholder instrumentalities on either an individual or collective basis.<br>- During each stage, samplings are carried out for 2 (two) identified districts/ cities, and for each of the districts/ cities 2 (two) sub-districts are picked in a random fashion.<br>- In each selected sub-district, 1 (one) retailer/ vendor/ agent/ distributor is identified. The sub-district's largest marketplace should be picked as the sampling location.<br>- Samplings at retailers/ vendors/ agents/ distributors are conducted as follows:<br>From each identified market retailer/ vendor/ agent/ distributor, pick at least 2 (two) packages of which combined weight equals 4kg, one pack for laboratory examination	Indonesia	Food Sampling Guideline for 2013. n.p. n.d.
Import monitoring (conducted by government)			
31. Provides justification for import monitoring at points of entry	The inspection of food at border or Ports is part of the responsibilities of the Government of Liberia to ensure that foods entering the border are compliant with the technical regulations of the national food fortification program. This is to make sure that specified foods are not imported and distributed to consumers if they are not fortified appropriately.	Liberia	The National Fortification Alliance of Liberia (NFA). Guidelines of the National Fortification Alliance of Liberia. Monrovia, Liberia: NFA. n.d.
32. Describes protocols and systems for import monitoring	Procedure<br>Reviewing the Certificate of Conformity or Certificate of Analysis and Labeling<br>The food inspector shall perform the following duties:-	Tanzania	Tanzania Food and Drugs Authority (TFDA). Guidelines for conducting external monitoring food fortification (1st ed.). Dar

Item	Example Language	Country	Reference
	a) Review the documents that usually certify the safety (and sometimes quality) of the imported product. Examine the Certificate of Conformity or Analysis, issued by a government authority or an officially recognized body from the country of origin, which would declare –supported by laboratory analysis- that the food fulfills the regulations established in the importer country.<br>b) Examine the packaging and the labeling to make sure that it indicates the brand name, batch number, country of origin and name and address of manufacturer. The food must comply with labeling requirements established in the regulations for fortified foods. Inspectors should also look out for false health claims that may be contrary to set recognized standards. They should record data in form F7/TFDA/DFS/FI&E/GL/001 of these guidelines.<br>Confirming the presence of indicator micronutrients<br>a) From each consignment randomly collect three representative samples of imported fortified food. Divide each sample into three portions (500g or 100ml per sample).<br>b) Collect samples based on brand names and perform spot test and record results in form F8/TFDA/DFS/FI&E/GL/001 of these guidelines.<br>Taking decisions to authorize<br>a) All samples should test positive for the indicator micronutrient.<br>b) If samples fail the qualitative test or fail to comply in terms of proper documentation and labeling requirements, the affected brand should not be allowed to enter the country.<br>c) If documentation is correct, and samples show the presence of the key micronutrient, authorize importation.		es Salaam, Tanzania: TFDA. 2012.

Item	Example Language	Country	Reference
33. If there are two or more government agencies involved in import monitoring, clarifies the roles and responsibilities between different government agencies in import monitoring	The Uganda National Bureau of Standards (UNBS) is mandated among others to formulate and enforce national standards to assure the safety and quality of products, including food products, produced and sold on the Ugandan market…While executing its role, the UNBS collaborates with other government institutions as mentioned below: The Ministry of Health (MoH) is the parent ministry that monitors the implementation and enforcement of...food fortification...Health inspectors under the Ministry of Health and the Ministry of Local Government (MoLG) are mandated under the public health act...and the food and drugs act...to carry out inspection at factory, entry ports and markets for foods. Uganda Revenue Authority (URA) collaborates with the MoH, UNBS, and NDA in the area of import inspection to ensure that only products (fortified foods, fortificants and fortification mixes) compliant to the national standards and regulations are allowed into the country.	Uganda	Ministry of Health, & Uganda National Bureau of Standards. Manual for regulatory monitoring of fortified foods. Kampala, Uganda: n.p. n.d.
34. If samples are to be taken, describes the sampling process: for example, number of samples, amount, frequency, individual vs. composite, where samples are taken in the process, and percent considered passing	For imported Wheat Flour which does not have the Certificate of Analysis (CoA) document attached...a sample will have to be taken and tested in accordance with the parameters of SNI...by a testing laboratory	Indonesia	Minister of Industry of the Republic of Indonesia. Regulation of the Minister of Industry of the Republic of Indonesia No. 35/M-IND/PER/3/2011 on obligatory application of the Indonesian national standard (SNI) for wheat flour as a foodstuff. Jakarta, Indonesia: Author. 2011.

Item	Example Language	Country	Reference
Enforcement/penalties			
35. Indicates roles and responsibilities in enforcing the legislation	The Ministry of Health and Medical Industry, the Ministry of Trade and Consumers and State Standards "Turkmenstandardlary" should ensure packaging, production, distribution and storage of flour within their scopes of responsibilities. The Ministry of Health should monitor and supervise the implementation of the activities.	Turkmenistan	President of Turkmenistan. Decree of the President of Turkmenistan No.7855 on the fortification of wheat flour with iron and folic acid. n.p. 2006.
36. States incentives to start fortification	Article 4. Powers of the Government of the Kyrgyz Republic In the field of enrichment of flour and prevention of iron deficiency, the government of the Kyrgyz Republic:...take measures for the economic stimulation of legal and physical persons involved in the production of enriched flour (by disbursing funds for the acquisition of premix, and other means)	Kyrgyzstan	President of the Kyrgyz Republic. Law of the Kyrgyz Republic on the enriching of baking flour. Kyrgyzstan: Author. n.d.
37. States incentives to continue fortification, including ensuring compliance	Foods that are fortified in compliance with regulatory requirements shall enjoy priority over non-fortified foods of the same class and category with respect to transport, storage, and display, including retail shelf space; shall be entitled to carry a logo authorized by the Ministries; and shall be entitled to any other favored treatment established by the government.	Liberia	The National Fortification Alliance of Liberia (NFA). Guidelines of the National Fortification Alliance of Liberia. Monrovia, Liberia: NFA. n.d.
38. States penalties to compel compliance	Failure to fulfill the terms of this Resolution constitutes a health violation subject to the penalties set forth in Law No. 6437 of August 20, 1977 and other applicable provisions	Brazil	National Health Surveillance Agency (ANVISA). RDC Resolution No. 344. Approve the technical regulations for the wheat flour and corn flour fortification with iron and folic acid, found in the annex to this resolution. Brazil: Official Federal Gazette. 2002.

Item	Example Language	Country	Reference
39. Penalties are objectively defined (e.g. first penalty=$100, second penalty=$300)	Any person convicted of an offence under this Act shall, subject to the provisions of subsection (2), be liable- a. on a first conviction, to a fine not exceeding four hundred rand or to imprisonment for a period not exceeding six months or to both such fine and such imprisonment; b. on a second conviction, to a fine not exceeding eight hundred rand or to imprisonment for a period not exceeding twelve months or to both such fine and such imprisonment; c. on a third conviction, to a fine not exceeding two thousand rand or to imprisonment for a period not exceeding twenty-four months or to both such fine and such imprisonment	South Africa	Foodstuffs, cosmetics, and disinfectants act. No. 54 of 1972. n.p. n.d.
40. States that enforcement is required to include feedback and support to improve performance and correct noncompliance	An effective monitoring system requires a set of established procedures, methodologies, distribution of responsibilities, and reporting requirements to ensure continuous assessment of functionality, and an efficient feedback mechanism, which facilitates the establishment and implementation of corrective measures when operational problems arise.	Ghana	Food and Drugs Board. Regulatory monitoring of fortified wheat flour. Guidelines for millers & food control agencies. n.p. 2007.
Laboratory			
41. References required analytical assays for nutrients (e.g. Liquid chromatography-mass spectrometry for folic acid, atomic absorption for iron and zinc.)	Spectrophotometric method for determination of iron, AACC Method 40-41A adapted…Determination of folic acid by HPLC analysis (this method describes the procedure for the determination of folic acid in flour samples using the Dionex 500 Series HPLC)	Kosovo	Ministry of Agriculture, Forestry, and Rural Development. Administrative instruction MAFRD No. 07/2013. For flour enrichment standard, flour enrichment control and quality control. Pristina, Kosovo: Author. 2013.

Item	Example Language	Country	Reference
42. States recognition that laboratory results are subject to several sources of variation and do not provide conclusive evidence of compliance or noncompliance	Enforcement agencies should ensure any samples taken at a mill are reflective of product being produced and should include a composite sample that is representative of a 'lot' and should account for laboratory and analytical variability. Results of samples taken by enforcement agencies should be considered in conjunction with information available at the mill that demonstrates the correct amount of folic acid is being added e.g. by examining sampling records and processes and evidence the required range was being achieved to the appropriate level.	Australia	Federal Register of Legislative Instruments F2014C01180. Standard 1.2.5 date marking of food. Australia: Author. 2014.
43. Focuses on the quantitative analysis of "marker" micronutrients such as iron	The determination of iron and in some, at least one complex B vitamin or one micronutrient constituent in a premix will be used as a proxy for the analysis of all others. Due to the complexity and cost of quantitative analysis of vitamins, these may not be performed on a routine basis. Iron analysis is relatively simple and well known will always be done and the levels of the other micronutrients may be assumed from the iron content results, where conducting other analytical methods on a routine basis is not feasible.	Ghana	Food and Drugs Board. Regulatory monitoring of fortified wheat flour. Guidelines for millers & food control agencies. n.p. 2007.
Reporting			
44. States how government monitoring results are shared with stakeholders	The director for Food Inspection and Certification. Reports of examination results for food products that came back TMS are sent as hard copies by mail and as soft copies to xxxxxxxx@yahoo.com and xxxxxxxx@gmail.com in MS Excel format as attached in Annex 9.	Indonesia	Food Sampling Guideline for 2013. n.p. n.d.

Abbreviations: n.p. no information of publisher/place of publication found; n.d. no date of publication found.

Marks KJ, Luthringer CL, Ruth LJ, et al. Review of grain fortification legislation, standards, and monitoring documents. *Glob Health Sci Pract*. 2018;6(2). https://doi.org/10.9745/GHSP-D-17-00427

* Example language extracted from documents reviewed in English (originally or translated to English); excludes Spanish documents.

** There were many excellent examples for each item of the checklist from many different country-grain combinations; this table is limited to one example for each item as it was not feasible to present all of the quality examples.
